# Towards the development of supramolecular self-associating amphiphiles as antibiofilm agents against *Pseudomonas aeruginosa* and *Candida albicans* biofilms†

**DOI:** 10.1039/d5tb00653h

**Published:** 2025-06-16

**Authors:** Kira L. F. Hilton, Hendrik J. F. Steyn, Kusasalethu S. Luthuli, Matthew Rice, Bree R. Streather, Esther Sweeney, Lisa J. White, Findley R. Morgan, Jennifer Rankin, Jennifer Baker, Charlotte Bennett, Hollie B. Wilson, Perry A. Hailey, Michelle D. Garrett, Jose L. Ortega-Roldan, J. Mark Sutton, Charlotte K. Hind, Carolina H. Pohl, Jennifer R. Hiscock

**Affiliations:** a School of Natural Sciences, University of Kent Canterbury UK CT2 7NH J.R.Hiscock@Kent.ac.uk; b Department of Microbiology and Biochemistry, Faculty of Natural and Agricultural Sciences, University of the Free State Free State South Africa 9301 PohlCH@ufs.ac.za; c UKHSA, Science Group, Manor Farm Road Salisbury SP4 0JG UK Charlotte.Hind@UKHSA.gov.uk; d Cancer Research Horizons, Babraham Research Campus Cambridge CB22 3AT UK

## Abstract

The rise of antimicrobial resistant (AMR) infection represents a growing threat to the global population and to economic health. The majority of antimicrobial innovations are developed against planktonic microorganisms, however those same microorganisms contained within a biofilm can become over 1000 times more resistant to antimicrobial (including antibiotic) agents. Supramolecular self-associating amphiphiles (SSAs) are a class of amphiphilic salts and related compounds that have shown the potential for development into antibiofilm agents. Within the scope of this work we present five structurally diverse SSAs. We characterise the self-associative properties of these SSAs in the solid state and in solution, before analysing the interactions of these agents with model synthetic membranes and determining their antibiofilm activity against WHO high/critical priority pathogens, *Pseudomonas aeruginosa* and *Candida albicans*. We also combine SSAs as 1:1 co-formulations and confirm the combination of SSA to inform both SSA phospholipid membrane interaction events and biological activity. Finally, we undertake a series of *in vitro* and *in vivo* DMPK experiments to verify the drug-like properties for these structurally diverse SSAs.

## Abstract 2

The use of homogenous and heterogeneous co-formulations of supramolecular self-associating amphiphiles to prevent the growth of clinically relevant biofilms.

## Graphical abstract

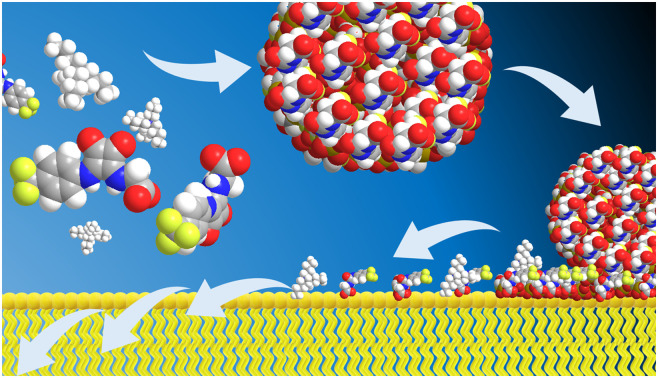

## Introduction

In 2019, the number of deaths directly attributed to antimicrobial resistant (AMR) infection were the same, if not greater than those directly attributed to HIV/AIDs or malaria,^1^ causing AMR to be termed by some as the ‘silent pandemic’.^2^ By 2050, it has been predicted that a greater number of people will die from the primary effects of AMR than currently die from cancer, unless significant interventions are implemented.^3^ Although the ongoing rise in AMR has traditionally been attributed to the misuse of antibiotics,^4–7^ there is evidence that the impacts of the COVID-19 pandemic could be a further contributing factor.^8,9^

Although, clinical resistance has now been identified to every antibiotic commonly prescribed, including colistin,^10,11^ 12 new antibiotics were approved for clinical use between 2017–2021, with a further 45 in development. Of these, 60% target WHO priority pathogens.^12^ Encouragingly, these numbers are predicted to rise through the launch of new initiatives to ensure the financial viability of the antibiotic development pipeline,^13^ previously absent.^14^ However, these novel antibiotics still do not fulfil the ever-increasing global need.^12,14,15^

The majority of AMR research is focused on inhibiting the growth of pathogenic planktonic cells, unintentionally ignoring biofilms,^16^ which is concerning when considering the significant economic impact that chronic and reoccurring biofilm based infections place on the global healthcare system.^17^ This is estimated at $281 billion (2017) for the treatment of wounds alone.^18^ When considered alongside the impact of biofilms within the food processing, marine, water supply and agricultural industries, the global economic impact associated with these microbial systems is estimated in the $trillions annually.^19^

Encasing microbes within a biofilm is known to reduce antibiotic efficacy by up to three orders of magnitude,^20^ while the polymicrobial nature of heterogenous biofilms is known to further drive AMR through both microbial synergy^21^ and plasmid exchange processes.^22^ The field of supramolecular chemistry has contributed a number of innovations towards the development of novel antimicrobial technologies,^23,24^ including synthetic ion transporters (ion carriers,^25,26^ ion channels^27^), polymeric materials^24^ and drug delivery vehicles.^28–31^ However, relatively few examples focus on the development of antibiofilm agents against heterogenous or homogenous biofilms. These examples include those by Nagarajan *et al.* who developed gluconamide based supramolecular gels,^32^ which act as antibiofilm agents against a range of Gram-positive and Gram-negative bacteria, including examples which appear on the 2024 WHO bacterial Priority Pathogen list, for which novel antimicrobial strategies are urgently needed^33^ (*e.g. Staphylococcus aureus*, *Listeria monocyogenes*, *Salmonella enterica* and *Pseudomonas aeruginosa*). In addition, Wang *et al.* have used eco-friendly β-cyclodextrin/stilbene-integrated supramolecular materials as antibiofilm agents against *Xanthomonas oryzae pv. oryzae*, which causes bacterial blight in rice.^34^ Finally, Zhou *et al.* have developed a series of supramolecular tripeptide based amphiphiles, which self-assembled into 1-D assemblies that could be reversibly disassembled due to environmental stimuli, such as light and competitive host–guest interactions, enabling control of antibiofilm activities against Gram-positive *S. aureus* and Gram-negative *P. aeruginosa*.^35^ Concerningly, even fewer examples (such as that provided by Yang and Hendrick *et al.*)^36^ exist where supramolecular innovations have been developed against biofilms containing fungal pathogens, specifically those detailed within the WHO priority fungal pathogens list, *e.g. Candida albicans* – one of the four fungal species to be considered a ‘critical threat’ to public health.^37^

**Fig. 1:**
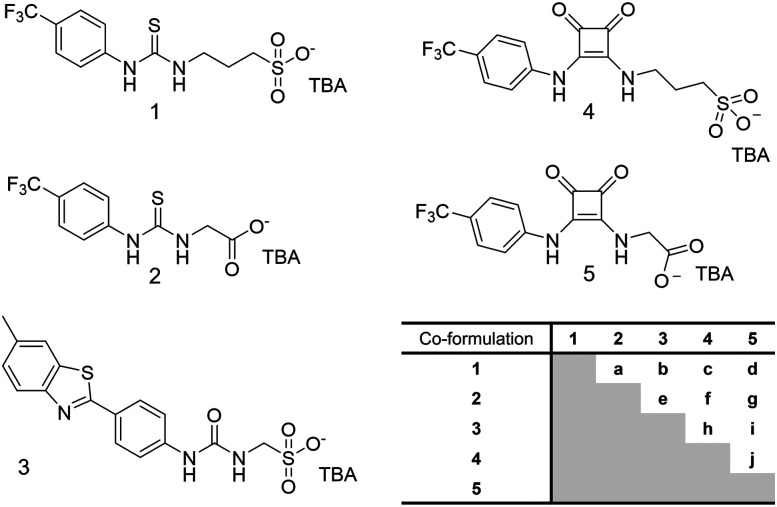
Chemical structures of 1–5 and a table showing the combination of SSAs use to make co-formulations a–j. TBA = tetrabutylammonium.

Within the scope of our own work, we have developed a class of supramolecular self-associating amphiphiles (SSAs), which exhibit drug-like properties and have been shown to act as both antimicrobial^38–40^ and antibiofilm agents,^30,41^ against WHO priority pathogens, both bacterial and fungal.^33,37^ SSAs, such as the examples detailed in Fig. 1, are a group of amphiphilic salts and related compounds, the majority of which contain a hydrophobic functionality, linked by a hydrogen bond donor/acceptor group, through an alkyl spacer, to an anionic moiety, that is charged balanced through the presence of a separate counter cation, typically tetrabutylammonium (TBA).^42,43^ These SSAs have been shown to adopt low order anionic dimeric species in polar organic solvent, spherical aggregates with a hydrodynamic diameter (*d*_H_) between ∼100–500 nm in 1 : 19 EtOH : H_2_O solutions and hydrogels upon the addition of inorganic salts such as NaCl.^30,42^

Within the scope of our preliminary work, a series of 11 structurally diverse SSAs were assessed for antibiofilm activity against biofilms of clinically relevant Gram-negative *P. aeruginosa* and fungal pathogen *C. albicans*.^41^ From these proof-of-principle studies, 2 demonstrated the greatest activity against *P. aeruginosa* biofilms, while 1 and 3 exhibited the greatest activity against *C. albicans* biofilms. When conducting preliminary structure activity relationship analysis, these data led us to hypothesise that increased SSA anion self-associative hydrogen bonding strength was a contributing factor to increasing antibiofilm efficacy. Building on the results from this proof-of-principle study we now explore the effects of: (i) increasing SSA anion hydrogen bond donor acidity through incorporation of the squaramide functionality (4 and 5) and, (ii) combining multiple SSAs (Fig. 1) on SSA self-association events and antibiofilm efficacy.

**Fig. 2:**
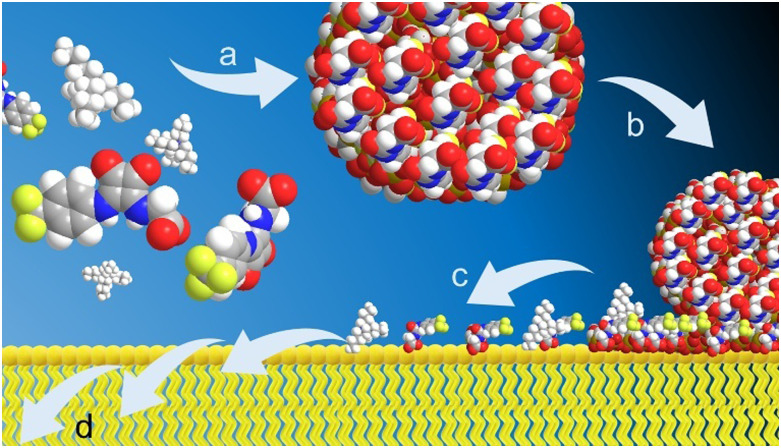
A cartoon summarising the proposed (a) SSA self-association processes under aqueous conditions, (b) and (c) phospholipid membrane interaction/adhesion events and (d) membrane permeation events.

As summarised in Fig. 2, following the synthesis of 1–5 we characterise the resultant self-associated SSA structures, we explore the interaction of these structures with biologically relevant synthetic phospholipid vesicles, before deriving the ability of the SSA systems to inhibit biofilm formation and alongside exemplar *in vitro* and *in vivo* DMPK profiles.

## Results and discussion

### Synthesis

SSAs 1–3 were synthesised through previously published methods.^12,13^ SSAs 4 and 5 were produced through the reaction of 4-trifluoromethyl aniline with dimethylsquarate in methanol, to produce an intermediate,^14^ that was subsequently reacted with the appropriate tetrabutylammonium (TBA) salt to give 4 and 5 as white solids in yields of 65% and 35% respectively.

### SSA self-association properties

**Fig. 3:**
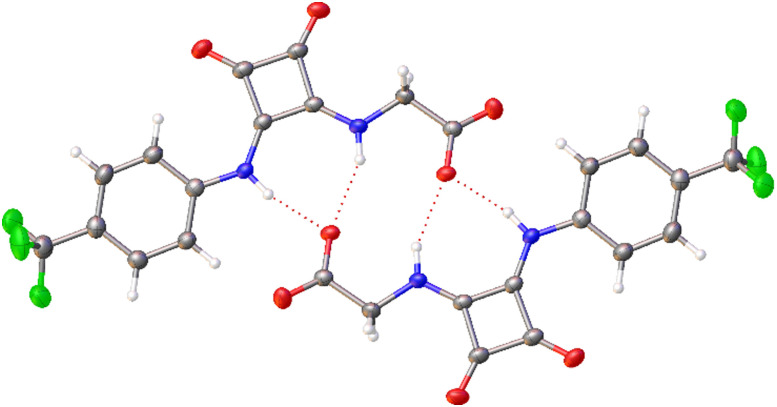
Single crystal X-ray structure obtained for 5 showing the anionic component of this amphiphilic salt forming a dimer, stabilised through the formation of four intermolecular hydrogen bonds. TBA counter-cations have been omitted for clarity. Grey = carbon, blue = nitrogen, red = oxygen, green = fluorine, white = hydrogen, red dashed lines = hydrogen bonds. Internal angle of dimerization = 180.0(3)°.‡‡A suitable crystal was selected and mounted on a Rigaku Oxford Diffraction Supernova diffractometer. Data were collected using Cu Kα radiation at 100 K. The structure was solved with the ShelXS^60^*via* Direct Methods and refined with ShelXL^61^ on least squares minimisation. Olex2^62^ was used as an interface to all ShelX programs. CCDC deposition number for the structure shown in Fig. 3 = CCDC 2387938.†

The self-associative properties of SSAs 1–5, and co-formulations a–j, were studied using previously published methodologies, the results of which are summarised in Table 1.^44,45^ Initially, we quantified the strength of homogeneous SSA anion self-association events, to confirm the effect of the squaramide – (thio)urea substitution. Quantitative ^1^H NMR spectroscopy experiments detected the presence of lower order self-association events in DMSO-d_6_ 1% DCM solutions (112 mM). Complimentary ^1^H NMR DOSY experiments conducted in DMSO-d_6_ 0.5% H_2_O solutions at 298 K confirmed the SSA anion and cation components to diffuse at different rates, providing supporting evidence towards the lack of any strong ion pairing interactions between the SSA anionic and cationic components under these experimental conditions. Calculating the hydrodynamic diameter (*d*_H_) of the SSA anion from these data confirmed the *d*_H_ < 1.7 nm for 1–5 (Section S8 and Fig. S32–37, ESI†), supporting the formation of lower order self-associated species, such as dimers. The formation of SSA anionic dimers has also been shown in the solid state through single crystal X-ray diffraction experiments.^42,43,46^ As shown in Fig. 3, the anionic component of squaramide incorporated SSA 5 was also shown to form a hydrogen bonded dimer in the solid state through single crystal X-ray diffraction studies. Here the SSA anion dimer is stabilised through the formation of four intermolecular squaramide-anion hydrogen bonds. Proton NMR spectroscopy dilution study data, obtained from a DMSO-d_6_ 0.5% H_2_O solution, were then fitted to both the EK (the equal K single component linear self-associative or dimerization model) and CoEK (the cooperative equal K single component linear self-associative or dimerization model) self-association models using Bindfit v0.5.^47,48^ These data were found to fit the EK over the CoEK model,^48^ which alongside the additional NMR spectroscopy data caused us to hypothesise the presence of SSA anion hydrogen bonded dimers present in a DMSO-d_6_ 0.5% H_2_O solution. Fitting these data to the EK binding isotherm enables the calculation of a dimerization constant (*K*_dim_, Table 1) for SSAs 1, 2, 3 and 5. The dimerization constants calculated confirm that substitution of the sulfonate ion (1 and 3) for the carboxylate ion (2) increase *K*_dim_ by approximately 50-fold. However, substituting the thiourea (2) for the squaramide (5) functionality increases the *K*_dim_ by a further 70-fold. Therefore, confirming our initial hypothesis, that incorporating the squaramide residue within the SSA anion structure would increase the strength of SSA anion self-association events.

**Table 1:** Physicochemical data produced to characterise SSA self-association events in a H_2_O : EtOH 19 : 1, D_2_O : EtOH 19 : 1 (quantitative ^1^H NMR spectroscopy only) or DMSO-d_6_/0.5% H_2_O (*K*_dim_ and ^1^H NMR DOSY only) solution. *K*_dim_ values were obtained from fitting ^1^H NMR spectroscopy dilution study data (change in chemical shift of the NH groups) to the appropriate binding isotherm model using Bindfit v0.5.^48^ Aggregate stability and *d*_H_ were obtained *via* zeta potential and DLS measurements respectively, at a concentration of 2.78 mM and a temperature of 298 K, following an annealing process unless otherwise stated. The *d*_H_ of the aggregates listed were obtained from intensity distribution peak maxima. CAC was derived at approximately 291 K from surface tension measurements. All quantitative ^1^H NMR spectroscopy experiments were conducted with a delay time (*d*_1_) of 60 s at 298 K and a concentration of 2.78 mM. Co-formulations a–j quantitative ^1^H NMR spectroscopy experiments represent the combined SSA anionic component. The values given in % represent the observed proportion of compound to become NMR silent. ST = surface tension. ZP = zeta potential

SSA	Quantitative ^1^H NMR (%)		*d*_H_ (nm)	CAC (mM)	ST at CAC (mN m^−1^)	ZP (mV)	*K*_dim_ (M^−1^)
	Anion	Cation					
1	33	31	184	5.6^43^	34^43^	−56	2.6
2	43	39	143	>10.0^b^	41^c^	−56	105
3	37	37	672	0.5^42^	47^42^	−86	2.7
4	44	39	162	>7.5^b^	42^c^	−79	d
5	33	37	173	>2.8^b^	43^c^	−73	7636

Co-formulation							
a	a	a	107	>10.0^b^	38^c^	−42	e
b	a	a	187	8.1	38	−72	e
c	a	a	107	>7.5^b^	39^c^	−68	e
d	a	a	99	>2.8^b^	45^c^	−54	e
e	a	a	189	2.8	46	−71	e
f	a	a	181	>7.5^b^	39^c^	−76	e
g	a	a	105	>2.8^b^	45^c^	−62	e
h	a	a	190	>7.5^b^	42^c^	−75	e
i	a	a	690	2.5	42	−69	e
j	a	a	84	>2.8^b^	44^c^	−82	e

a Evidence of higher order self-association events obtained, exact percentages could not be calculated.

b This represents the limit of solubility preventing determination of CAC.

c Surface tension at limit of solubility.

d Slow exchange processes prevented *K*_dim_ calculation.

e Multiple component system prevented data fitting.

Moving into aqueous solution, quantitative ^1^H NMR spectroscopy experiments were undertaken in a D_2_O : EtOH 19 : 1 solution (2.78 mM), to confirm the presence of higher order self-association events, leading to the formation of aggregates which exhibit solid-like properties, and thus appear silent by ^1^H NMR spectroscopy. All SSAs and co-formulations exhibited evidence of higher order self-association events under these experimental conditions, see Table 1. Interestingly, for homogeneous solutions of 1–5 there was not much variation in the proportion of SSA to be taken up into these higher-order aggregates under these experimental conditions. The *d*_H_ of these higher-order aggregates, subsequently determined by dynamic light scattering (DLS) for 1–5 (Table 1), showed those higher order aggregates produced to exhibit a similar size and size distribution profile (intensity peak maxima = 143–184 nm), with the exception of 3 (intensity peak maxima = 672 nm), the only SSA to contain the inclusion of a benzothiazole moiety.

The stability of the homogenous aggregates formed in H_2_O : EtOH 19 : 1 at 2.78 mM of 1–5 were then determined through zeta potential studies (Table 1). The thiourea based self-associated aggregates were found to be the least stable with zeta potential measurements of −56 mV. Substituting the thiourea for the squaramide residue increased the stability of those nanostructures formed, with zeta potential measurements of −79 mV and −73 mV reported for 4 and 5 respectively. We believe that this is due to the increased strength of SSA anion dimerization events (2*K*_dim_ = 105 M^−1^ and 5*K*_dim_ = 7636 M^−1^). Although 3 does not contain a squaramide residue and has a much lower self-associative binding constant of 2.7 M^−1^ in DMSO-d_6_ 0.5% H_2_O, the enhanced stability provided by the planar, hydrophobic benzothiazole functionality gives rise to aggregates with a zeta potential of −86 mV. Tensiometry experiments, the results of which are summarised in Table 1, confirmed 1–5 to exhibit surfactant properties in a H_2_O : EtOH 19 : 1 solution, as surface tension is lowered upon increasing concentrations of SSAs, however the critical aggregation concentration (CAC) could only be determined for 1 and 3 due to SSA solubility.

We then moved to characterize the self-associative properties of two-component heterogeneous SSA systems, co-formulated in 1 : 1 molecular ratio, giving rise to co-formulations a–j, listed in Fig. 1. These co-formulations were prepared in a H_2_O : EtOH 19 : 1 solution. Homogenous solutions of each SSA were combined at equal volumes. The heterogeneous solution subsequently underwent an annealing process, in which the solution was heated to 50 °C for 60 seconds, before cooling to room temperature. As summarised in Table 1, the properties of aggregates formed from co-formulations a–j differ quite considerably from the homogenous aggregates of 1–5, prepared under identical experimental conditions.

Quantitative ^1^H NMR spectroscopy confirmed the presence of higher-order aggregates with solid-like properties for co-formulations a–j at 2.78 mM, in a 19 : 1 D_2_O : EtOH solution. Here SSA co-formulation was found to result in the production of higher-order aggregated species which demonstrated increased variation in *d*_H_ (determined through DLS studies) and stability (determined through zeta potential studies) compared to the homogenous SSA aggregates produced under analogous experimental conditions. In general, the incorporation of 5 led to aggregates with a smaller *d*_H_ (99 nm, 105 nm, 84 nm), unless combined with 3, in which case this trend was reversed as this combination of SSAs led to the production of the largest heterogenous SSA aggregates identified (*d*_H_ = 690 nm). However, we believe that this size of aggregate may be produced through the aggregation of smaller species present in solution. Zeta potential measurements showed the presence of 3 (increased hydrophobicity and potential to stabilise intermolecular π–π stacking interactions) and 4 (a sulfonate squaramide based SSA) to increase the stability of the heterogenous aggregates to the greatest extent, with the most stable aggregates formed when combining 4 and 5, which we hypothesise to be due to enhanced intermolecular hydrogen bonding strength, provided through the presence of the squaramide moiety (Table 1). Tensiometry experiments showed that all SSA co-formulations retained the surfactant nature of the homogenous solutions, although solubility prevented calculation of accurate CAC values.

### Phospholipid membrane interaction studies

In previous studies we have shown spherical aggregates of 3 to firstly adhere to the surface of both Gram-negative and Gram-positive bacteria before forming a coating over the surface of the microbe and subsequently permeating through the cell membrane(s) into the cell interior.^38^ It is this interaction with the cell wall, specifically the interactions with the phospholipid bilayer that we hypothesise to contribute to the antimicrobial activity of SSAs.^49^

To gauge the activity and selectivity of 1–5 and a–j against microbial membranes, a series of synthetic model vesicle systems were prepared, containing a variety of common bacterial membrane phospholipid headgroups: (i) phosphatidylglycerol (PG) only; (ii) phosphatidylethanolamine (PE) : PG 3 : 1 and; (iii) PE : PG 1 : 1.^50^ Following our previous work in this area, we first quantified the ability of 1–5 and co-formulations a–j to lyse the aforementioned synthetic model phospholipid vesicle systems.^49^ A summary of these results are shown in Table 2, Figures and Tables present in the ESI† (Section S13 and S14). Here the fluorescent dye calcein was loaded into synthetic vesicles at a concentration where the dye self-quenches (70 mM). When the vesicle is lysed, the calcein is released to the extravesicular solution, effectively reducing calcein concentration to a point where the dye no longer undergoes self-quenching. Through comparison to a positive control experiment, in which 100% vesicle lysis was achieved through the addition of 2% X-100 Triton, the proportion of vesicles to undergo lysis in the presence or absence of SSA can be calculated. The data was then fitted to the Hill or linear equation to determine the effective concentration of SSA needed to lyse 50% of the vesicles present in solution (EC_50_) (Section S14, ESI†).^51^

**Table 2:** Summary of total molar concentration required to lyse 50% of the vesicles at specific timepoints (EC_50_ values) of 1 and co-formulations containing 1 (a–d) determined through fitting calcein loaded vesicle titration data primarily to the Hill equation, at 298 K in PBS buffer 5% EtOH. For all experimental data see Section S14†

SSA/co-formulation	EC_50_ (mM)				
	1 min	5 min	10 min	15 min	20 min
	PG				
1	0.0677	0.0320	0.0156	0.0062	0.0037
a	0.1324	0.0879	0.0410	0.0220	0.0114
b	0.1461	0.0590	0.0289	0.0203	0.0084
c	0.9913	0.0922	0.0521	0.0233	0.0123
d	0.1808	0.0644	0.0265	0.0140	0.0080

	PE : PG 3 : 1				
1	0.0302	0.0090	0.0040	0.0022	0.0017
a	0.5529	0.1167	0.0735	0.0888	0.1120
b	0.1258	0.0454	0.0232	0.0151	0.0108
c	0.1025	0.0183	0.0063	0.0037	0.0022
d	0.1292	0.0269	0.0139	0.0066	0.0045

	PE : PG 1 : 1				
1	0.0549	0.0095	0.0048	0.002	0.0015
a	0.9010	0.0302	0.0121	0.0041	0.0023
b	0.0842	0.0281	0.0215	0.0127	0.0144
c	0.0631	0.0246	0.0105	0.0067	0.0046
d	0.0971	0.0161	0.0066	0.0036	0.0027

All SSAs and SSA co-formulations exhibited vesicle lysis EC_50_ values > 0.1 mM at 20 min against the phospholipid vesicles of varying headgroup composition, apart from 1 and SSA co-formulations a–d, which all contain 1. In general, the fit of these data to the hill or linear equation increased in error where the EC_50_ was > 0.1 mM at 20 min, indicating the presence of complex processes than cannot be modelled through the fit of these data to these equations. The results for 1 and a–d, summarised in Table 2, show the vesicle lysis process to be time dependent, with the amount of SSA required to lyse 50% of the vesicles present decreasing over the 20-minute time frame of this experiment. SSA 1 was found to exhibit the greatest vesicle lysis properties across all synthetic microbial membranes tested, with a preference demonstrated towards the lysis of PE containing vesicle systems. However, co-formulation a (addition of a carboxylate, thiourea incorporated SSA) demonstrates the greatest degree of selectivity towards the lysis of PE : PG 1 : 1 membranes, while co-formulation c (addition of a sulfonate, squaramide incorporated SSA) demonstrates selectivity towards the lysis of PE : PG 3 : 1 membranes after 20 min incubation. Therefore, we provide evidence that SSA co-formulation enables us to tailor the phospholipid vesicle lysis properties of the individual SSA.

Next, we sought to understand the effects of 1, 2, 4 and 5 and a, c, d, f, g, j on synthetic vesicle phospholipid membrane fluidity using a fluorescence polarization (FP) assay.^52^ Here, the fluorescent reporter dye 1,6-diphenyl-1,3,5-hexatriene (DPH) is incorporated into the vesicles phospholipid bilayer. As membrane fluidity is increased, the DPH reporter dye moves more freely within the phospholipid bilayer environment, resulting in a decreased FP value. However, should the membrane become more rigid, the movement of the dye is decreased and the FP value increases. As 3 is intrinsically fluorescent and overlaps with the fluorescence excitation/emission properties of the DPH, this SSA and co-formulations incorporating this compound were not included.

When considering the effects of 1, 2, 4 and 5 on the membrane fluidity of the model synthetic vesicle systems, the squaramide based SSAs were shown to increase membrane rigidity to a far greater extent than the analogous thiourea based SSAs, 1 and 2 (Table 3). As the proportion of PG present within the synthetic vesicle systems was increased, so was the propensity of 1, 2, 4 and 5 to increase membrane rigidity. However, the presence of increasing proportions of PE led to increased membrane fluidity upon the addition of 1, 2, 4 and 5. Co-formulations c, d, f and g which contain both a thiourea based SSA (1, 2) and a squaramide based SSA (4, 5) increased the membrane rigidity of all synthetic vesicles tested, compared to the effects of adding 1 or 2 as single agents. Conversely, co-formulation a, which contained a combination of 1 and 2, caused a decrease in membrane rigidity for all synthetic membrane systems tested, compared to the effects of adding 1 or 2 as single agents. Therefore, through SSA co-formulation we have shown the ability to control the membrane fluidity properties for these synthetic vesicle systems.

**Table 3:** Average FP values (*n* = 3) obtained upon the addition of 1, 2, 4, 5, a, c, d, f, g and j (1.5 mM) to PG, PE : PG 3 : 1 or PE : PG 1 : 1 phospholipid vesicles incubated with DPH (10 μM), conducted in HEPES buffer (150 mM KCl, 10 mM HEPES, pH 7.4, EGTA 2.0 mM) at 298 K. Error = standard deviation of the mean

	PG	PE : PG 3 : 1	PE : PG 1 : 1		PG	PE : PG 3 : 1	PE : PG 1 : 1
1	29 ± 1	−9 ± 3	50 ± 1	c	124 ± 14	42 ± 5	111 ± 8
2	11 ± 8	−40 ± 0	−1 ± 1	d	105 ± 1	75 ± 1	70 ± 3
4	150 ± 8	73 ± 14	151 ± 5	f	94 ± 2	80 ± 3	75 ± 4
5	152 ± 6	106 ± 7	146 ± 4	g	112 ± 3	66 ± 13	41 ± 6
a	11 ± 1	−33 ± 3	17 ± 5	j	147 ± 4	83 ± 21	135 ± 3

Finally, in line with our previous work, we moved to characterise the membrane adhesion and permeation events of 1–5 and co-formulations a–j with PE : PG 1 : 1 synthetic phospholipid vesicles.^53^ This vesicle composition was prioritised for further analysis as this composition acts as a model for an AMR resistant strain of the ESKAPE pathogen *P. aeruginosa*,^54,55^ categorised as high priority by the WHO.^33^ As summarised in Table 4, membrane permeation (PF) and membrane adhesion factors (MAF) for the SSA anionic components were calculated through the use of ^1^H CPMG NMR spectroscopy experiments, using vesicles produced by extrusion through a 1000 nm polycarbonate membrane.

**Table 4:** PF and MAF determined for the SSA anion and anionic components of SSA co-formulations a–j against PE : PG 1 : 1 phospholipid vesicles. All PFs have an error of ± 0.1 unless otherwise stated. NMR spectroscopy samples were prepared containing: SSA (200 μM), 95% HEPES buffer (HEPES (10 mM), NaCl (10 mM)), 5% D_2_O, and sodium trimethylsilylpropanesulfonate – DSS (1 μM). Spectra were collected at 298 K. All MAFs have an error of ± 0.05 unless otherwise stated. The PF rank highlights the most phospholipid membrane permeable (1) to the least phospholipid membrane permeable (8) agent or co-formulation. The MAF rank highlights the most membrane adherent (1) to the least membrane adherent (8) SSA or SSA co-formulation. A PF value greater than one suggests an SSA or co-formulation is permeable and an MAF value less than one suggests that an SSA or co-formulation is adhering to the phospholipid membrane. SSA′ corresponds to the SSA anion with the lowest compound number in the co-formulation. SSA′′ corresponds to the SSA anion with the highest compound number in the co-formulation

SSA	PF	PF rank	MAF	MAF rank	SSA′ PF	SSA′ MAF	SSA′′ PF	SSA′′ MAF
1	1.01	4	0.94	8	N/A	N/A	N/A	N/A
2	0.85	5	0.88	6	N/A	N/A	N/A	N/A
3	0.00	8	0.00	1	N/A	N/A	N/A	N/A
4	1.48^a^	1	0.38	=2	N/A	N/A	N/A	N/A
5	0.67	7	0.38	=2	N/A	N/A	N/A	N/A

Co-formulation								
a	0.83	6	0.89	7	0.91	0.92	0.78	0.85
b	d	—	d	—	0.32	1.10^f^	e	e
c	d	—	d	—	0.99	0.88	e	e
d	1.03^b^	3	0.83	5	1.06	0.93	1.02	0.56
e	d	—	d	—	0.63	0.69	e	e
f	d	—	d	—	0.97	0.84	e	e
g	d	—	d	—	0.81	0.92	e	e
h	d	—	d	—	e	e	0.52	1.66^c^
i	d	—	d	—	e	e	0.87^b^	0.62
j	1.11^b^	2	0.56	4	1.11^b^	0.56	g	g

a Error = ±0.3.

b Error = ±0.2.

c Error = ±0.08.

d Could not be determined due to one or more of the molecular components adhering to the phospholipid membrane beyond the limit of detection. Within the limits of the experiment, all of the compound appears adhered to the phospholipid membrane.

e Compound adhered to the vesicle beyond the limit of detection. Within the limits of the experiment, all of the compound appears adhered to the phospholipid membrane.

f Where the MAF > 1 we hypothesise a synergistic effect between the components present.

g Independent system components could not be distinguished due to overlapping ^1^H NMR spectroscopy resonances.

**Fig. 4:**
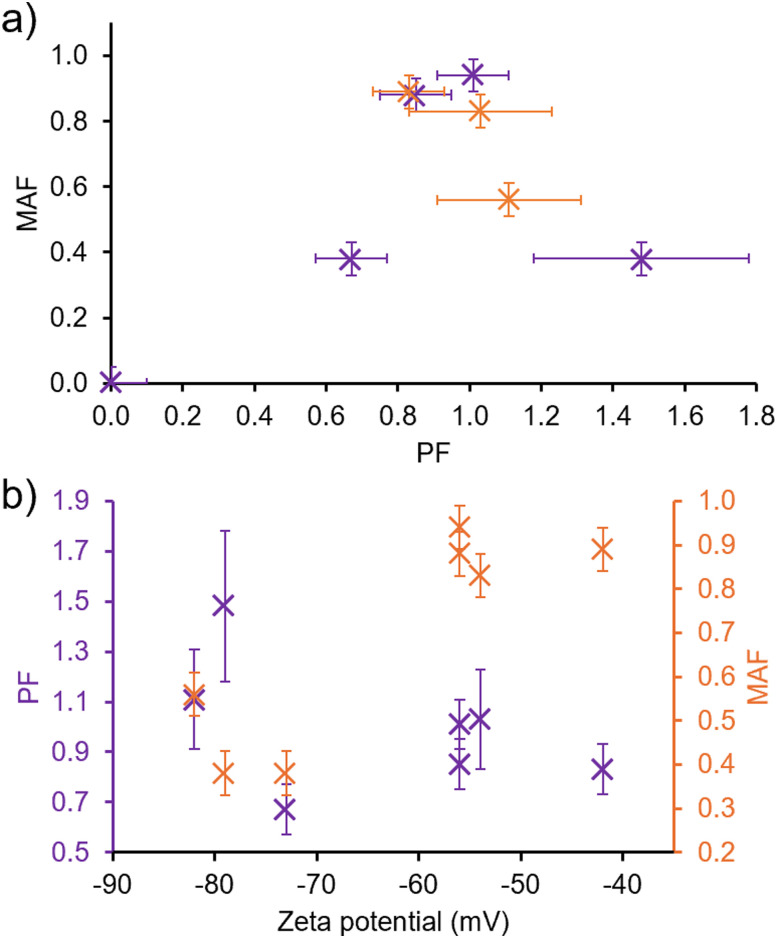
Scatter graphs illustrating the relationship between: (a) MAF and PF for 1–5 (purple) and co-formulations a, d and j (orange); (b) MAF (orange) or PF (purple) and zeta potential.

Here the lower the MAF, the stronger the adhesion of the molecule to the surface of the phospholipid membrane. A value of 0 indicates that there is no detectable concentration of SSA anion which remains free in solution, thus, all the detectable SSA anion appears bound to the phospholipid membrane. A PF value of less than 1 indicates that no detectible concentration of SSA has permeated the phospholipid bilayer and can usually be attributed to membrane adhesion events limiting the signal intensity. A PF value of 1 or above provides evidence of SSA permeation. The greater this value above 1, the greater the membrane permeation potential of a compound. A comparison of the MAF and PF for SSA and SSA co-formulations are provided in Fig. 4(a).

To increase the PF from 0 to 1, a decrease in the strength of SSA membrane association events are observed. However, for an SSA to demonstrate membrane permeation properties (PF ≥ 1), and for those properties to be further enhanced, a decrease in MAF is required which correlates to enhanced molecular membrane adhesion properties. We hypothesise that to achieve PF ≥ 1, the SSAs may have to form extended self-associated structures similar to the membrane pores or channels reported by Hou and *et al.*^27^ The increased strength of SSA membrane adhesion events leading to enhanced SSA membrane permeation properties supports the formation of stable membrane bound structures, that facilitate the flow of SSAs through the phospholipid bilayer, increasing the PF.

Next, we compared MAF and PF values obtained for 1–5 and co-formulations a, d and j with physicochemical data (Table 1), including zeta potential. As shown in Fig. 4(b), although there was no trend identified between permeation factor and zeta potential value, there is a trend between MAF and zeta potential. Here, to achieve a MAF < 0.5 the zeta potential value must be between −70 mV and −80 mV. In our previous work we have provided evidence towards a relationship between the enhanced stability of a SSA aggregate formed under these experimental conditions and the presence of hydrogen bonded intermolecular interactions,^42,43^ in addition to increased antimicrobial properties.^38^ We believe that this trend provides evidence that these same factors that drive SSA aggregate formation (SSA anion intermolecular interactions and lipophilicity) also contribute to enhanced membrane adhesion properties.

**Fig. 5:**
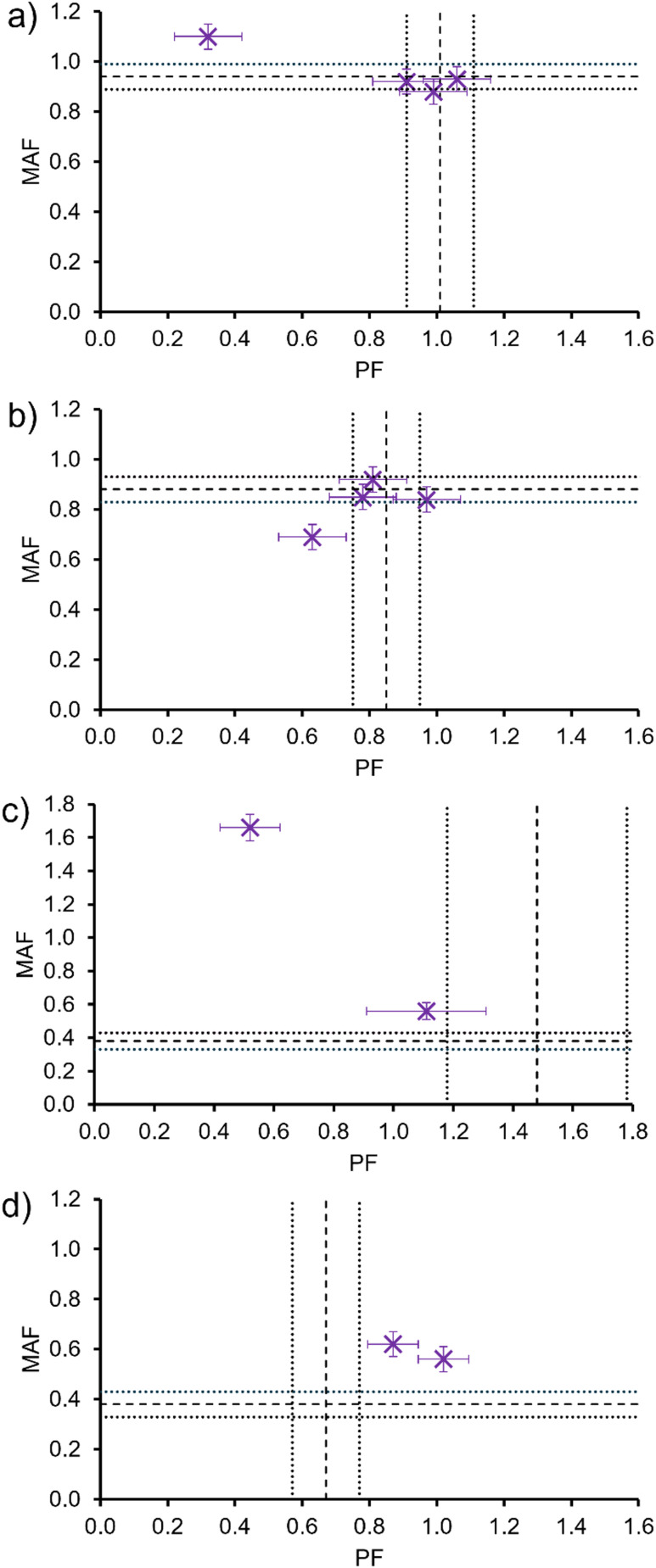
Graphs comparing the PF and MAF (long-dashed lines, error indicated by short-dashed lines) for homogenous solutions of (a) 1, (b) 2, (c) 4 and (d) 5 with the PF and MAF for the same SSA in a co-formulation (purple).

An advantage of this NMR methodology is the ability to elucidate the MAF and PF for the individual SSA components within the co-formulation, as long as the ^1^H NMR resonances do not overlap. Although there is no way to distinguish the ^1^H NMR resonances of 3 (and therefore the corresponding MAF and PF) in a co-formulation with the other SSAs, individual MAF and PF of SSAs 1, 2, 4 and 5 within SSA co-formulations could be determined and therefore compared with the MAF and PF of the homogenous solutions of 1–5. The results of these comparisons are shown in Fig. 5. Here it can be seen that although the MAF and PF of an individual SSA is retained within a co-formulation, there are examples for each SSA where the MAF and PF for an SSA is altered through co-formulation. This provides evidence that the SSAs may act synergistically in co-formulation to enable tailoring of the individual SSA membrane adhesion and permeation properties.

**Fig. 6:**
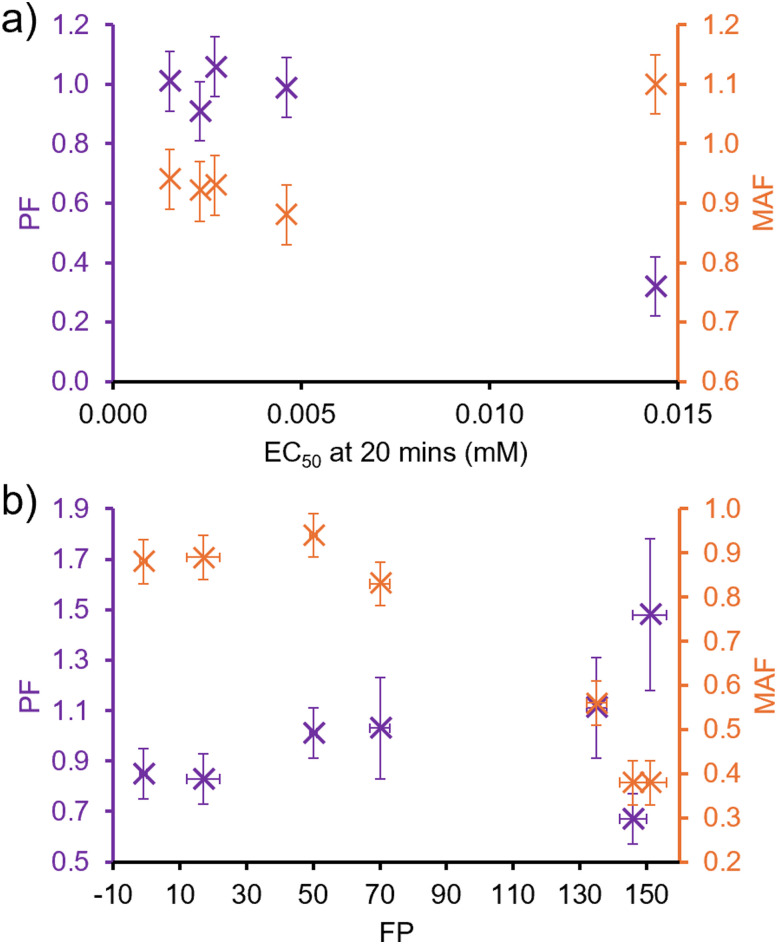
Scatter graphs illustrating the relationship between 1–5 and co-formulation a, d and j with MAF (orange) or PF (purple) and: (a) EC_50_ at 20 min (Table 2) for membrane lysis of 1 : 1 PE : PG vesicles; (b) FP values for 1 : 1 PE : PG vesicles at 1.5 mM (Table 3).

Further comparison of the MAF and PF for 1–5 and their co-formulations with 1 : 1 PE : PG vesicles and, the EC_50_ values of the same SSAs/co-formulation required to lyse vesicles of analogous phospholipid composition after 20 min (Table 2), indicated that to decrease vesicle lysis EC_50_ value, the SSA or co-formulation should exhibit a lower MAF and evidence of membrane permeation, PF ≈ 1 (Fig. 6(a)). Therefore, the SSAs which exhibit a greater strength of membrane adhesion and enhanced membrane permeation properties, also exhibit enhanced membrane lysis properties. In addition, when comparing MAFs and PFs to the corresponding FP values obtained from membrane fluidity experiments (Table 3), although there was no evidence towards the influence of PF on membrane fluidity, interestingly, as shown in Fig. 6(b), there is evidence for a relationship between decreasing MAF and decreasing FP value. Therefore, this data leads us to hypothesise that the enhanced strength of SSA membrane adhesion results in decreased membrane fluidity.

### Antibiofilm and antimicrobial activity

Moving away from synthetic vesicle systems, the activity of SSAs and their co-formulations was next established against biofilms of pathogenic microorganisms listed on WHO prioritised pathogens lists.^33,37^ These biofilms are highly complex when compared with synthetic vesicle systems. The cell membrane is comprised not only of phospholipids, but also of proteins and carbohydrates, which combined with the presence of the cell wall and extracellular matrix (ECM), adds to the chemical complexity of the cell surface.^56^

**Fig. 7:**
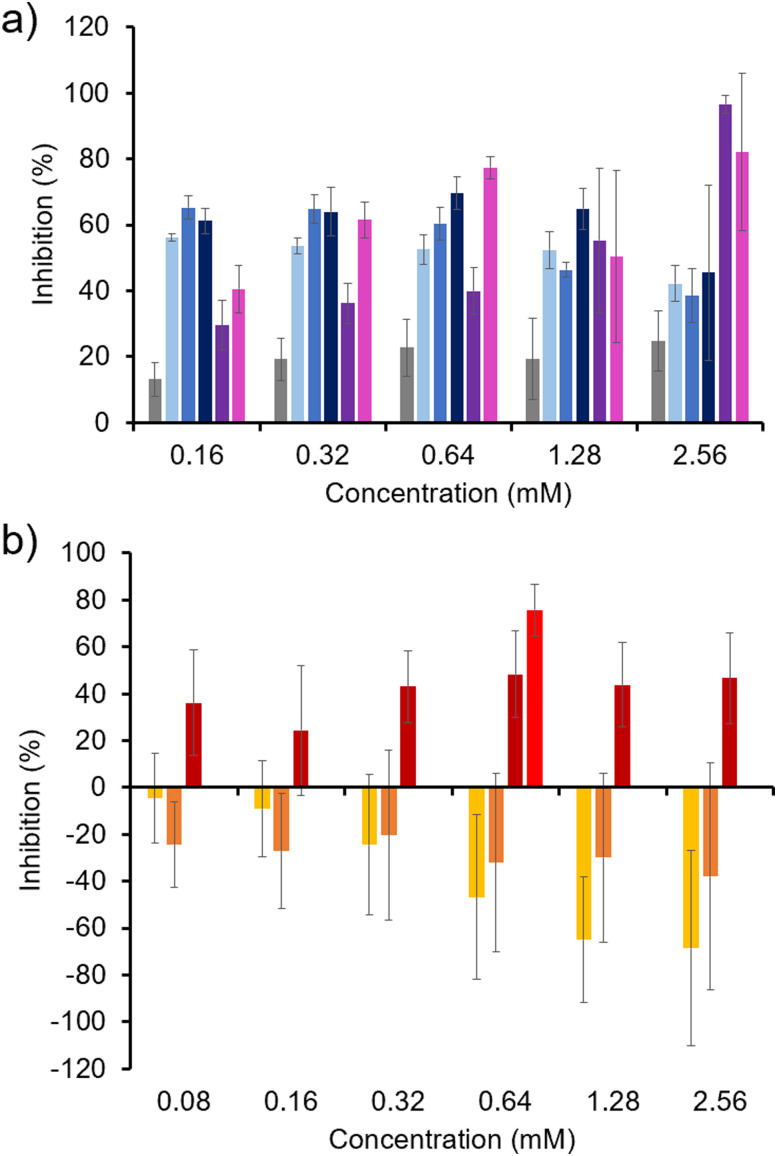
Percentage inhibition of *P. aeruginosa* (PAO1) biofilms by (a) 1–5 (*n* = 3) and, (b) co-formulations a, b, e and j (*n* ≥ 3) determined through XTT assay. Grey = TBA chloride (control), light blue = 1, medium blue = 2, dark blue = 3, purple = 4, pink = 5, yellow = a, orange = b, dark red = e, bright red = j. Error = ± 1 SD. Data relating to the antibiofilm activity of 1–3 has been previously published and is included to enable comparison of 4 and 5, alongside co-formulations a, b, e and j.^41^

Initially, the ability of SSAs 1–5 to inhibit biofilm formation was determined against *P. aeruginosa* (PAO1) biofilms through an XTT metabolic assay, used to determine the viability of cells within a biofilm (Section S4, ESI†).^57^ As shown in Fig. 7(a), 4 and 5 exhibited an enhanced antibiofilm activity over 1 and 2 at the highest SSA concentration tested (2.56 mM), with 4 also showing a greater activity over 3. At this concentration 4 and 5 were shown to completely inhibit biofilm formation. However, at lower concentrations (0.16 mM), 1–3 showed enhanced antibiofilm activity over 4 and 5 while still maintaining biofilm inhibition >55%. In all cases 1–5 exhibited an enhanced antibiofilm activity over the control TBACl. We believe that this may indicate that SSAs which contain a squaramide residue may experience an additional barrier to eliciting an antimicrobial effect that is concentration dependent. As these SSAs exhibit enhanced hydrogen bond donor properties, this may include enhanced interactions with the ECM, preventing preferential interactions of these SSAs with the bacteria.

To investigate the effects of SSA co-formulation on *P. aeruginosa* (PAO1) antibiofilm activity, the antibiofilm efficacy of a, b and e, which incorporate those SSAs that show the greatest efficacy against this biofilm as single agents at lower concentrations (0.16 mM) were established (Fig. 7(b)). In addition, a proof-of-principle experiment was also conducted with j which contains 4 and 5 to establish the potential for any enhancement in activity against a *P. aeruginosa* (PAO1) biofilm at lower concentrations (0.64 mM). Co-formulations e and j exhibited similar antibiofilm activities to homogenous SSA solutions, however a and b showed a loss of single agent antibiofilm activity, while higher concentrations of a were also shown to elicit a positive effect on bacterial growth.

SSAs have previously been hypothesised to elicit their mechanism of antibacterial action through selective phospholipid membrane adhesion and permeation events.^38,49^ Analysis of PF and MAF data (Table 4 and Fig. 5) suggested that co-formulation of the SSAs could either maintain or alter phospholipid membrane affinity and permeability properties of the SSA. These findings would seem to correlate with the results of these antibiofilm studies, with confirmation of the SSAs either causing the individual agent's activity to be maintained or minimised.

**Fig. 8:**
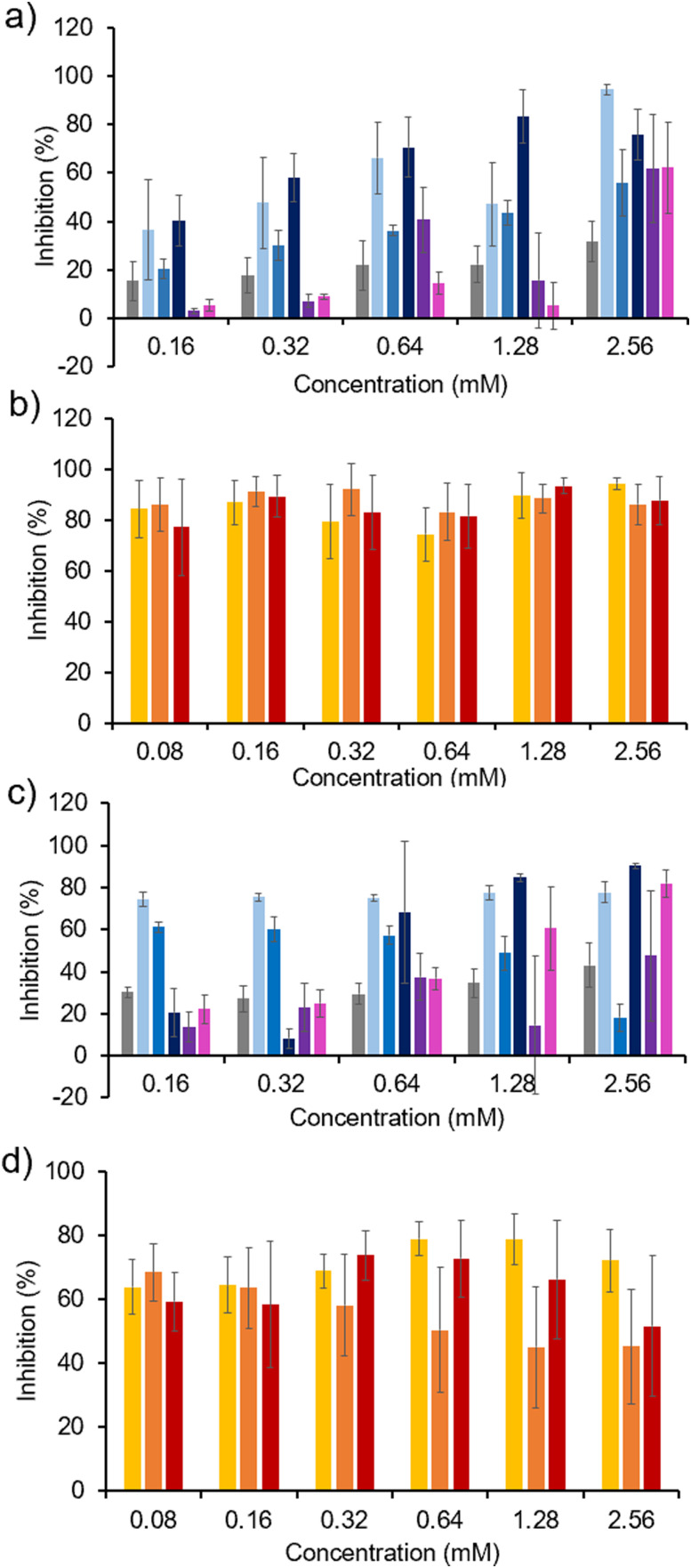
Percentage inhibition of (a) and (b) *C. albicans* (SC5314) biofilms and (c) and (d) polymicrobial biofilms of *C. albicans* (SC5314) and *P. aeruginosa* (PAO1) by (a) and (c) 1–5 (*n* = 3) and, (b) and (d) co-formulations a, b and e (*n* ≥ 3) determined through XTT assay. Grey = TBA chloride (control), light blue = 1, medium blue = 2, dark blue = 3, purple = 4, pink = 5, yellow = a, orange = b, red = e. Error = ± 1 SD. Data relating to the antibiofilm activity of 1–3 has been previously published and is included to enable comparison of 4 and 5, alongside co-formulations a, b and e.^41^

Next the activity of 1–5 against biofilms of *C. albicans* (SC5314) and polymicrobial biofilms of both species were established using the same XTT metabolic assay in line with previous work (Fig. 8).^41^ Against biofilms of *C. albicans* (SC5314), 1 and 3 were found to exhibit the greatest antibiofilm activity, competitive with 4 and 5 even at higher concentrations, as shown in Fig. 8(a). Again, there is a distinctive decrease in antibiofilm activity with decreasing SSA concentration, as previously observed with *P. aeruginosa* (PAO1) biofilms (Fig. 7(a)), supporting our previous hypothesis that enhanced adhesion of the SSA to the ECM may result in decreased antibiofilm activity at lower concentrations, causing 4 and 5 to exhibit a lower antibiofilm activity than the control compound TBACl at 0.32 and 0.16 mM. In general, the order of SSA activity against *C. albicans* (SC5314) biofilms follows the trend: (thio)urea sulfonate SSA (1, 3) > (thio)urea carboxylate SSA (2) > squaramide (4 and 5). This trend is the inverse of the SSA *K*_dim_ (Table 1), the lower the *K*_dim_ the greater the antibiofilm activity. Co-formulations a, b and e which contain combinations of the most active single antibiofilm SSAs at lower concentrations (1, 2 and 3) were subsequently investigated for antibiofilm activity, Fig. 8(b). Unlike the *P. aeruginosa* (PAO1) biofilms (Fig. 7(b)), these co-formulations demonstrated enhanced activity against *C. albicans* (SC5314) biofilms (Fig. 8(b)), at 0.08 mM these co-formulations still demonstrate almost complete biofilm inhibition. Again, these results show that the co-formulation of SSAs changes the antibiofilm properties of the individual SSAs, which we hypothesise to be a direct result of SSA co-formulation processes modifying the interactions of the SSA with biological membranes (Tables 2–4 and Fig. 5).

Finally, the activity of 1–5 against polymicrobial *C. albicans* (SC5314) and *P. aeruginosa* (PAO1) biofilms was examined. Here, the noticeable difference in SSA activity against the heterogenous as opposed to the homogenous biofilms is the decreased activity of 3 at lower concentrations <0.64 mM, as shown in Fig. 8(c). We hypothesise that this may be due to the presence of the hydrophobic, planar benzothiazole functionality interacting negatively with the biofilm environment, preventing preferential antimicrobial interactions. However, the antibiofilm activity of 1 and 2 is maintained at lower concentrations (0.16 mM) between ∼60–80%. Again, co-formulations a, b and e, containing combinations of the most active antibiofilm SSAs (1, 2 and 3) against this heterogenous biofilm were then investigated for their antibiofilm activity (Fig. 8(d)). Here the co-formulations of 1 and 2 conserve the antibiofilm activity of the individual SSAs, however, in this instance the co-formulations of 3, which exhibited a lower antimicrobial activity at lower concentrations in comparison to 1 and 2, showed an enhanced antibiofilm activity when co-formulated with 1 and 2, providing evidence of a synergistic relationship between 1 and 3 (b), and 2 and 3 (e) towards increased antibiofilm activity. Interestingly, the minimum concentration of compound required to inhibit 100% of growth, (minimum inhibitory concentration, MIC) determined for 1–5 and a, b, e and j against a panel of Gram-positive and Gram-negative bacteria, showed these SSAs and their co-formulations to be more effective against microbes contained in biofilms (Fig. 7 and 8) compared to the planktonic cells (Section S18, ESI†). However, it should be noted that an MIC is a different measure of antimicrobial activity, requiring complete inhibition of growth, while antibiofilm activity in this instance is measuring a percentage inhibition of biofilm formation.

**Fig. 9:**
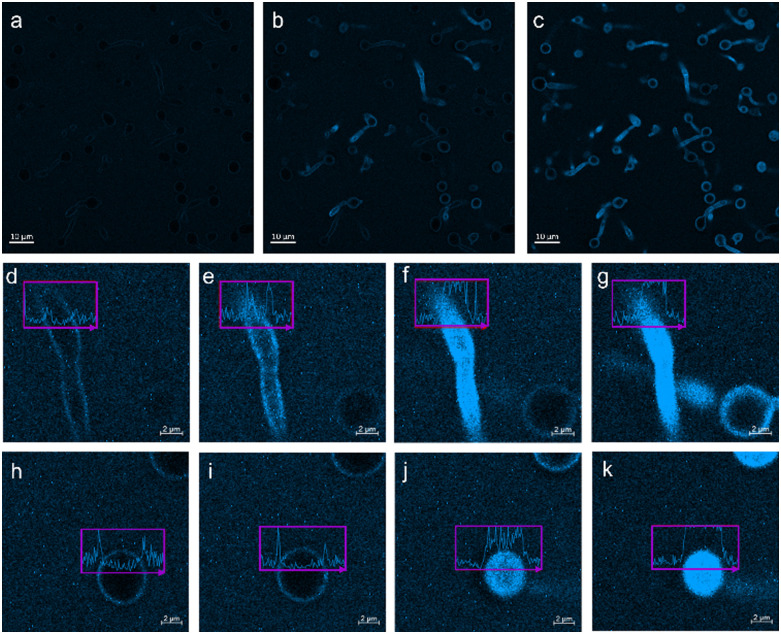
(a)–(c) Fluorescent microscopy images of 3 taken at different time intervals in the presence of *C. albicans*. (d)–(g) Distribution of 3 within the (pseudo)hyphae of *C. albicans* at differing time intervals. (h)–(k) Distribution of 3 along the *C. albicans* cell body at differing time intervals. Time intervals: d and h = 2 second, e and i = 180 seconds, f and j = 360 seconds, g and k = 600 seconds. Scale bar a–c = 10 μm. Scale bar d–k = 2 μm. Excitation/emission wavelength = 365/461 nm.

We have previously used a combination of fluorescence and transmitted light microscopy to confirm the adhesion and permeation of intrinsically fluorescent 3 through both Gram-positive and Gram-negative cell membranes,^38^ however we have yet to confirm this mechanism of action for live *C. albicans.*Fig. 9 presents the results of complementary studies of 3 with *C. albicans* (SC5314). Here, *C. albicans* biofilms were cultivated on a glass coverslip, 3 was added and a live recording of the fluorescence microscopy experiment initiated. Fig. 9(a)–(c) show stills collected from this time lapse video included within the ESI.† SSA 3 is shown to adhere to the surface of the microbial cell before internalising, demonstrating a preference for the (pseudo)hyphae. These regions are the ‘tail-like’ protrusions projecting out of the spherical cell body and are involved in pathogenesis.^58,59^ The comparative concentration of SSA present at the cell surface and within *C. albicans* was also determined with respect to time, Fig. 9(d)–(k). Upon the addition of 3, a measure of background fluorescence intensity was determined (Fig. 9(d) and (h)), any increase in fluorescence intensity, indicating an increased concentration of 3 could then be confirmed. At 180 seconds into the video (360 seconds in real time) (Fig. 9(e) and (i)) the external surface of the (pseudo)hyphae increases in fluorescence intensity (Fig. 9(e)), compared to the yeast cell body (Fig. 9(i)). It takes double the time for the yeast cell body to increase to a comparative fluorescence intensity (360 seconds into the video, Fig. 9(j)), at which point, 3 has completely permeated the (pseudo)hyphae (Fig. 9(f)). In summary, as shown by the fluorescence intensity graphs provided in Fig. 9(d)–(k), the SSA initially interacts with cell membrane, demonstrating a preference for adhesion to the surface of (pseudo)hype over the cell body, before permeating through the cell membrane (leaving the cell surface unstained) to become internalised within the cell. However, the results from these fluorescence microscopy experiments did not show any evidence of cell membrane lysis events.

### *In vitro* and *in vivo* drug metabolism and pharmacokinetics (DMPK)

A TBA–benzothiazole–urea–sulfonate SSA with a similar structure to that of 3 has already been shown to exhibit a drug-like profile through initial *in vitro* assays consisting of plasma stability, mouse, rat and human microsomal stability, plasma protein binding and Caco-2 permeability. This was followed by *in vivo* pharmacokinetic analysis in mice. Here the TBA–benzothiazole–urea–sulfonate SSA was administered intravenously and distribution of the TBA–benzothiazole–urea–sulfonate SSA was measured in the lung, muscle and liver tissues.^49^

Due to the presence of a squaramide in 4 and 5, we also chose to investigate the DMPK properties of 5 to understand the implication of squaramide and carboxylate functionalities on the drug-like properties of SSAs. For experimental details and data, please see Section S5 (ESI†). These data show that the introduction of the carboxylate and squaramide functionalities into the SSA structure does not impact on the *in vitro* drug-like properties of the SSAs. However, these structural modifications do impact the SSA *in vivo* profile, increasing systemic clearance and volume of distribution *versus* a TBA–benzothiazole–urea–sulfonate SSA when the SSA is administered intravenously into the mouse.

## Conclusions

We have investigated the self-associative properties of five structurally diverse, but related SSAs, 1–5. We have shown that the introduction of the squaramide functionality increases the strength of SSA anion hydrogen bonded self-association events by an order of magnitude or more in comparison to the thio(urea) analogues within a DMSO-d_6_ 0.5% H_2_O solution. We have also co-formulated 1–5 in a 1 : 1 ratio to produce a–j. We have shown that changing the structure of these SSAs as well as co-formulation can be used to modulate the self-associating properties of these systems, and the properties of any aggregate formed in a 19 : 1 H_2_O : EtOH solution.

SSAs 1–5 exhibit concentration and molecular structure dependent phospholipid membrane lysis and effect on phospholipid membrane fluidity properties, for a range of synthetic model microbial membranes. These phospholipid membrane lysis properties and effects on membrane fluidity were found to be further tuned through SSA co-formulation. Determination of MAF and PF for the homogeneous and heterogenous 1 : 1 SSA co-formulations against PE : PG 1 : 1 phospholipid membranes showed that, depending on the particular SSA co-formulation, the individual components of that co-formulation either retained their MAF and/or PF, of the individual SSAs, or could be modulated.

The antibiofilm activity of SSAs was established for 1–5 against homogenous and heterogeneous biofilms containing *C. albicans* (SC5314) and/or *P. aeruginosa* (PAO1). For all biofilms, near complete inhibition of biofilm formation was observed for at least one condition tested, with 1, 2 and 3 retained as lead antibiofilm SSAs. These SSAs were subsequently supplied as a 1 : 1 co-formulation to the biofilms, resulting in enhanced biofilm inhibition compared to the individual SSAs against *C. albicans* (SC5314), and to enhance the activity of 3 against heterogenous *C. albicans* (SC5314) and *P. aeruginosa* (PAO1) biofilm formation. Previous data had already led us to hypothesise that the SSA mechanism of antimicrobial activity included selective adhesion to, and subsequent permeation through the cellular membranes. However, we now confirm this also for *C. albicans* (SC5314), where 3 is shown to preferentially target the (pseudo) hyphae. With this hypothesised mechanism of action, we believe that these data show that through co-formulation, we can modify SSA membrane adhesion and permeation properties selectively and as a result increase antibiofilm activity against target biofilms of interest. Finally, we performed a range of DMPK studies with 5, that show that the substitution of the (thio)urea functionality with the squaramide does not vastly impact the *in vitro* drug-like properties of the SSAs, however does impact the intravenous *in vivo* profile as observed with an increase in clearance and volume of distribution.

## Author contributions

KLFH: investigation; validation; writing – original draft, review & editing. HJFS, KSL, MR, BRS, LJW, ES, JR, JB, CB, HBW: investigation; validation. JLOR, CKH, CHP, JMS: supervision; validation; writing – review & editing; funding acquisition. JRH: conceptualization; funding acquisition; project administration; supervision; writing – original draft, review & editing.

## Conflicts of interest

There are no conflicts to declare.

## Supplementary Material

TB-013-D5TB00653H-s001

TB-013-D5TB00653H-s002

## Data Availability

The data supporting this article have been included as part of the ESI.†
