# Supplementary material for: Towards the development of supramolecular self-associating amphiphiles as antibiofilm agents against Pseudomonas aeruginosa and Candida albicans biofilms
Source: J Mater Chem B. 2025 Jun 16;13(27):8239–51. doi: 10.1039/d5tb00653h (PMC12169104; doi:10.1039/d5tb00653h)
Supplement: TB-013-D5TB00653H-s001 [file TB-013-D5TB00653H-s001.pdf]

Supplementary Information (SI) for Journal of Materials Chemistry B.
This journal is © The Royal Society of Chemistry 2025

Towards the development of supramolecular self-associating amphiphiles as antibiofilm agents against *Pseudomonas aeruginosa* and *Candida albicans* biofilms

Kira L. F. Hilton, Hendrik J. F. Steyn, Kusasalethu S. Luthuli, Matthew Rice, Bree R. Streather, Esther Sweeney, Lisa J. White, Findley R. Morgan, Jennifer Rankin, Jennifer Baker, Charlotte Bennett, Hollie B. Wilson, Perry A. Hailey, Michelle D. Garrett, Jose L. Ortega-Roldan, J. Mark Sutton,* Charlotte K. Hind,* Carolina H. Pohl* and Jennifer R. Hiscock*

Contents

Section S1: Chemical structures 3
Section S2: Summary data tables for SSA physicochemical data 4
Section S3: General methods 7
Section S4: Microbial materials and methods 10
Section S5: *In vitro* and *in vivo* studies 11
- *In vitro* Absorption, Distribution and Metabolism studies methods: 11
- *In vitro* Absorption, Distribution and Metabolism results 13
- *In vivo* Pharmacokinetic study methods 13
- *In vivo* Pharmacokinetic study results 14

Section S6: Chemical synthesis 15
Section S7: Single Crystal XRD data 17
Section S8: NMR spectroscopy data 18
- Molecular characterisation data 18
- ^{1}H qNMR spectroscopy data 22
- ^{1}H NMR spectroscopy dilution study data 31
- ^{1}H DOSY data 33

Section S9: DLS data 39
- Overview 69

Section S10: Zeta potential data 70
Section S11: Critical aggregation concentration (CAC) determination data 77
Section S12: Low level computational modelling data 79
Section S13: Vesicle leakage assay data 81
- PG lipid titrations 81
- PE:PG 3:1 lipid titrations 89
- PE:PG 1:1 lipid titrations 97

Section S14: Determination of vesicle lysis EC_{50} values 105

PG vesicle lysis titrations 106
PE:PG 3:1 vesicle lysis titrations 145
PE:PG 1:1 vesicle lysis titrations 184
Summary 223
Section S15: FP membrane fluidity data 225
PG FP titrations 225
PE:PG 3:1 FP titrations 230
PE:PG 1:1 FP titrations 235
Comparison of membrane fluidity FP data 240
Section S16: Vesicle adhesion data 245
PG FP titrations 245
PE:PG 3:1 FP titrations 247
PE:PG 1:1 FP titrations 250
Comparison of membrane adhesion FP data 252
Section S17: MAF and PF determination from NMR spectroscopy data 255
Section S18: Antimicrobial data 263
Section S19: Mass spectrometry data 264
Section S20: References 265

Section S1: Chemical structures

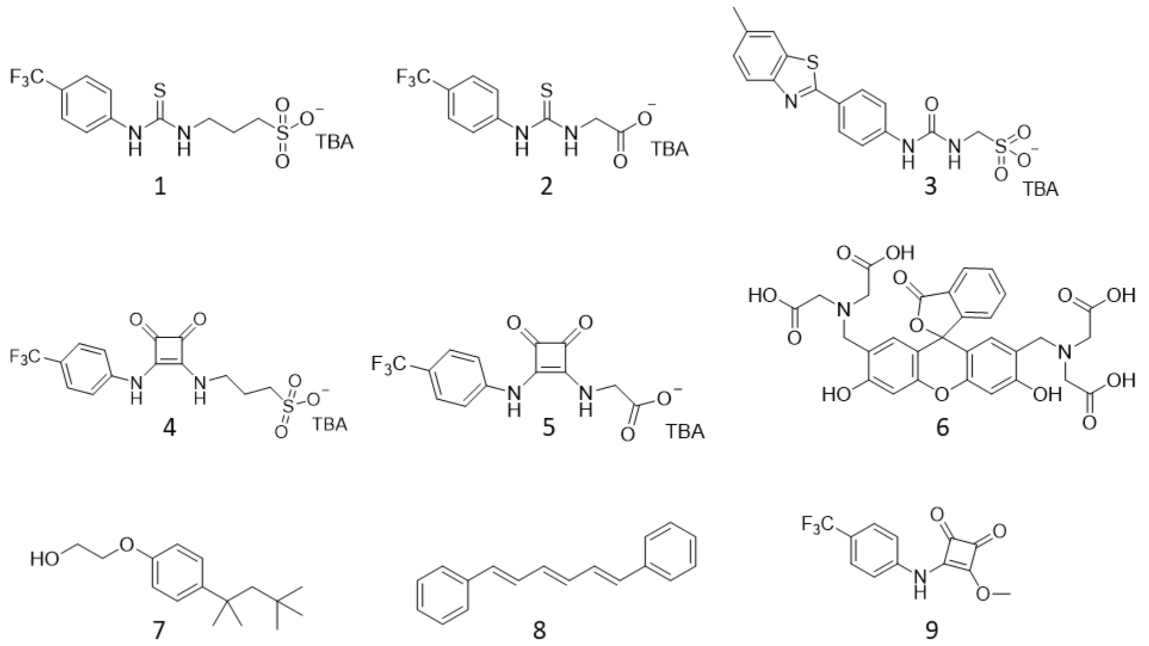

Figure S1 – Chemical structures of SSAs **1** – **5**, calcein **6**, Triton X-100 **7**, 1,6-diphenyl-hexa-1,3,5-triene (DPH) **8**, squaramide intermediate **9**.

Section S2: Summary data tables for SSA physicochemical data

Table S1 - Overview of solution state studies of single component homogenous SSAs. Physicochemical data produced to characterise SSA self-association events in a H_2O:EtOH 19:1, D_2O:EtOH 19:1 (quantitative ^{1}H NMR spectroscopy only) or DMSO-d_6/0.5 % H_2O (K_{dim} and ^{1}H NMR DOSY only) solution. K_{dim} values were obtained from fitting ^{1}H NMR spectroscopy dilution study data (change in chemical shift of the NH groups) to the appropriate binding isotherm model using Bindfit v0.5. Aggregate stability and d_H were obtained via zeta potential and DLS measurements respectively, at a concentration of 2.78 mM and a temperature of 298 K, following an annealing process unless otherwise stated. The d_H of the aggregates listed were obtained from intensity distribution peak maxima. CAC was derived at approximately 291 K from surface tension measurements. All quantitative ^{1}H NMR spectroscopy experiments were conducted with a delay time (d_1) of 60 s at 298 K and a concentration of 2.78 mM. The values given in % represent the observed proportion of compound to become NMR silent. ST = surface tension. ZP = zeta potential. PDI = polydispersity index relating to the d_H calculated from DLS studies.

SSA	DMSO-d_6/0.5 % H_2O		H_2O:EtOH 1:19				
	d_H (nm)	K_{dim} (M^{-1})	CAC (mM)	ST at CAC (mN/M)	Zeta potential (mV)	d_H (nm)	PDI
**1**	1.50	3	5.61	33.59	-55.70	184.36	0.0315
**2**	1.42	105	*b*	*b*	-55.50	142	0.0350
**3**	1.64	3	0.5	46.5	-86.33	672.27	0.0658
**4**	1.65	*a*	<7.5	*b*	-79.31	162.46	0.0388
**5**	1.61	7636	<2.78*b*	*b*	-73.44	174	0.0614

a – Compound is in slow exchange.

b – CAC is above the limit of solubility.

Table S2 – Overview of solution state studies of co-formulated heterogenous 1:1 mixtures of SSAs. Where a concentration is given, this concentration represents the total molecular concentration. Physicochemical data produced to characterise SSA self-association events in a H_2O:EtOH 19:1 or D_2O:EtOH 19:1 (quantitative ^1H NMR spectroscopy only) solution. Aggregate stability and d_H were obtained via zeta potential and DLS measurements respectively, at a concentration of 2.78 mM and a temperature of 298 K, following an annealing process unless otherwise stated. The d_H of the aggregates listed were obtained from intensity distribution peak maxima. CAC was derived at approximately 291 K from surface tension measurements. All quantitative ^1H NMR spectroscopy experiments were conducted with a delay time (d_1) of 60 s at 298 K and a concentration of 2.78 mM. The values given in % represent the observed proportion of compound to become NMR silent. ST = surface tension. ZP = zeta potential. PDI = polydispersity index relating to the d_H calculated from DLS studies.

**Co-formulation**	**SSA components**	**H_2O:EtOH 1:19**				
		**CAC (mM)**	**ST at CAC (mN/M)**	**Zeta potential (mV)**	**d_H (nm)**	**PDI**
**a**	**1** + **2**	<10*a*	*a*	0.99	107.1	0.0745
**b**	**1** + **3**	8.06	37.51	-96.99	187.24	0.0600
**c**	**1** + **4**	<7.5*a*	*a*	-68.2	107.1	0.0688
**d**	**1** + **5**	<2.78a	*a*	-53.9	98.7	0.0485
**e**	**2** + **3**	2.83	46.38	-71.13	188.8	0.0596
**f**	**2** + **4**	<7.5*a*	*a*	-76.06	181.4	0.0374
**g**	**2** + **5**	<2.78*a*	*a*	-62.2	105.3	0.0103
**h**	**3** + **4**	<7.5*a*	*a*	-74.9	189.8	0.0686
**i**	**3** + **5**	2.45	42.14	-69.4	689.8	0.0809
**j**	**4** + **5**	<2.78*a*	*a*	-82.05	84	0.0580

a – CAC is above the limit of solubility.

Table S3 – Overview of results from quantitative ^{1}H NMR spectroscopy studies in D_2O:EtOH 19:1. Values given in % represent the observed proportion of compound that has been apparently 'lost'. Anion (combined) is the average of the two anionic species for each co-formulation. ^{1}H NMR spectroscopy experiments carried out at 298 K, at a total molar concentration of 2.78 mM. T_1 increased to 60s. Anion 1 represents the SSA with the lowest compound number (Figure S1) within the 1:1 co-formulation and Anion 2 represents the SSA with the highest compound number (Figure S1) within the 1:1 co-formulation.

**Co-formulation**	**Anion 1**	**Anion 2**	**Anion (combined)**	**Cation**
**a**	52	64	58	64
**b**	66	70	68	69
**c**	*a*	*a*	59	65
**d**	52	56	54	67
**e**	77	73	75	71
**f**	71	64	68	70
**g**	71	73	72	70
**h**	60	49	54	66
**i**	58	57	58	59
**j**	33	41	37	49

a – Anionic components could not be separated through ^{1}H NMR spectroscopy.

Section S3: General methods

General remarks: A positive pressure of nitrogen and oven dried glassware were used for all reactions. All solvents and starting materials were purchased from known chemical suppliers or available stores and used without any further purification unless specifically stipulated. All NMR spectra were obtained using a Bruker AV2 400 MHz or AVNEO 400 MHz spectrometer. The data was processed using Topspin software. NMR Chemical shift values are reported in parts per million (ppm) and calibrated to the centre of the residual solvent peak set (s = singlet, br = broad, d = doublet, t = triplet, q = quartet, m = multiplet). Tensiometry measurements were undertaken using the Biolin Scientific Theta Attension optical tensiometer. The data was processed using Biolin OneAttension software. A Hamilton (309) syringe was used for these measurements. High resolution mass spectrometry was performed using a Waters Acquity H-Class (Quaternary Solvent Manager(QSM) and Flow Through Needle(FTN)), configured to allow direct injection into a Waters Vion. DLS and Zeta Potential studies were carried out using an Anton Paar Litesizer^{TM} 500 and processed using Kalliope TM professional. Rheological measurements were recorded on an Anton Parr modular compact rheometer (MCR302) using a cylinder probe ST10-4V-8.8/97.5. UV-Vis absorbance and optical density measurements were conducted and analysed using a Clariostar plate reader and MARS data analysis software. Fluorescence and fluorescence polarisation measurements were conducted using a Clariostar plate reader and analysed using MARS data analysis software.

Formation of the heterogenous SSA co-formulation: Homogenous solutions of each SSA (2.78 mM) were combined at equal volumes. The heterogeneous solution then underwent an annealing process in which the solution was heated to 50 °C for 60 seconds before being allowed to cool to room temperature.

Self-association constant calculation: Self-association constants were determined using Bindfit v0.5 (http://app.supramolecular.org/bindfit/).[1] All data can be accessed online using the hyperlinks provided.

Tensiometry Studies: All samples were prepared in an EtOH:H_2O (1:19) solution. All samples underwent an annealing process in which the various solutions were heated to approximately 313 K before being allowed to cool to room temperature, allowing each sample to reach a thermodynamic minimum. All samples were prepared through serial dilution of the most concentrated sample. Three surface tension measurements were obtained for each sample at a given concentration, using the pendant drop method. The average of the three values were plotted to calculate the critical aggregation concentration (CAC).

Mass spectrometry: Ammonium Acetate (10 mM, Supleco Lichropur, Lot AM 1890034413) was prepared in 95:5 H_20 (Fischer Chemicals, Lot 2361141) :Methanol (Sigma-Aldrich, Lot STBK7695). The QSM was configured to deliver 0.02ml/min using direct injection. The FTN delivered 10ul per injection and samples were analysed in triplicate. The parameters for the mass spectrometry are as follows: Source Temperature = 121 °C; Desolvation Temperature = 350 °C; Cone Gas = 49 L/h; Desolvation Gas = 597 L/h; Capillary = 2.54 kV; Mode = negative mode, High Definition MSE; Scan Time = 1.000 secs.

Mass Spectrometry Standards: Lock mass spray consisted of a Leucine Enkephalin (ex. Waters, Lot W27072310) (100 pg/µL) and was prepared according to Waters procedure but adjusted to 100 pg/µL; Mass spec calibration performed using Major Mix (ex. Waters, Lot W07022424) and prepared according to Waters procedure 715005131. Rev A.

Sample Preparation: Approximately 1 mg of each compound was dissolved in 1 mL of methanol (Sigma-Aldrich, Lot STBK7695). This solution was further diluted 10 µL in 1000 µL in 95:5 H_20 (Fischer

Chemicals, Lot 2361141) :methanol (Sigma-Aldrich, Lot STBK7695). Samples directly injected into a flow of 10 mM ammonium acetate in 95:5 (H_20:methanol) at 0.2 mL/min.

DLS studies: All vials used for preparing the samples were clean and dry. All solvents used were filtered to remove any particulates that may interfere with the results obtained. Samples of differing concentrations were obtained through serial dilution of a concentrated solution. All samples underwent an annealing process, in which they were heated to 313 K before being allowed to cool to 298 K to allow each sample to reach a thermodynamic minimum. A series of 9 or 10 runs were recorded at 298 K.

Zeta potential studies: All vials used for preparing the samples were clean and dry. All solvents used were filtered to remove any particulates that may interfere with the results obtained. All samples underwent an annealing process, in which they were heated to 313 K before being allowed to cool to room temperature, allowing each sample to reach a thermodynamic minimum. The final zeta potential value given is an average of the number of experiments conducted at 298 K.

Preparation of vesicles: All lipids were purchased as powder stocks from Avanti Polar Lipids. Lipid vesicles were prepared according to standard procedures. Each phospholipid (~ 20 mg) was dissolved in chloroform: methanol 3:1 (~ 10 mL) and the solvent was removed under reduced pressure. The thin film of dried lipid was further dried in vacuo overnight. Lipid films were resuspended in the desired buffer and subjected to 10 freeze-thaw cycles in liquid nitrogen and extruded 19 times through a 200 nm polycarbonate membrane.

Preparation of calcein loaded vesicles:[2] Lipid films were resuspended in calcein dye (70 mg calcein, 865 µL deionized H_2O, 135 µL NaOH (2 M), with NaOH added dropwise until calcein dissolved) and subjected to 10 freeze-thaw cycles in liquid nitrogen and extruded 19 times through a 200 nm polycarbonate membrane. The suspension was run down a size exclusion column packed with sephadex G-50 using 1 X PBS. The hydrodynamic dimeter of the lipids was monitored using an Anton Paar Litesizer^{TM} 500 to ensure the separation of free and entrapped calcein. Phospholipid concentration was adjusted to 60 µM for vesicle leakage experiments.

Vesicle leakage assay: Black bottom 96-well plates were prepared by serially diluting desired SSA in a EtOH: H_2O 1:19 solution across the plate, the appropriate lipid solution (100 µL, 60 µM) was added to each well to give a total well volume of 200 µL. A solution of 2 % Triton X-100 was used for a 100 % lysis control and a EtOH: H_2O 1:19 solution was used as a 0 % lysis control. Fluorescent measurements were taken at 25 °C using an excitation value of 495 nm and an optimised gain of 800. Data were acquired in endpoint mode. All experiments were repeated in triplicate to ensure experimental reproducibility.

Preparation of DPH fluorescent labelled vesicles: Lipid films were resuspended in buffer (150 mM KCl, 10 mM HEPES, pH 7.4, 2 mM EGTA) and subjected to 10 freeze-thaw cycles in liquid nitrogen and extruded 20 times through a 200 nm polycarbonate membrane. For fluorescent labelling, the desired vesicles were pre-incubated with DPH (10 µM) at 60 °C for 1 hour.

Membrane fluidity assay: Black bottom 96-well plates were prepared by serially diluting desired SSA in a EtOH: H_2O 1:19 solution across the plate, the appropriate DPH labelled vesicles (100 µL, 60 µM) were added to each well to give a total well volume of 200 µL. FP measurements were taken at 25 °C using a 355 nm filter for excitation and a 430 m for emission. The DPH labelled vesicles were set to a FP value of 100 mP. Data were acquired in endpoint mode. All experiments were repeated in triplicate to ensure experimental reproducibility.

Fluorescence polarisation: Lipid films were resuspended in Buffer (150 mM KCl, 10 mM HEPES, pH 7.4, 2 mM EGTA) and subjected to 10 freeze-thaw cycles in liquid nitrogen and extruded 19 times through a 200 nm polycarbonate membrane. Black bottom 96-well plates were prepared by serially diluting desired vesicle solution (2 mM) across the plate, the appropriate SSA solution (100 µL, 0.3 mM) was added to each well to give a total well volume of 200 µL. Fluorescent SSA solutions were set to a FP value of 100 mP. Data were acquired in endpoint mode. All experiments were repeated in triplicate to ensure experimental reproducibility.

Membrane permeability and adhesion assay, determining membrane adhesion factors (MAF) and permeation factors (PF): Lipid vesicles of DOPE and PG, in a 1:1 ratio by weight were prepared as described in 'preparation of vesicles' with one alteration. Here vesicles were extruded 21 times through a 1000 nm polycarbonate membrane. NMR samples were prepared containing: SSA (200 µM), 95% HEPES buffer (HEPES (10 mM), NaCl (10 mM)) and 5% D_2O, and sodium trimethylsilylpropanesulfonate - DSS (1 µM). In order to calculate the PF and MAF the paramagnetic relaxation enhancement agent (PRE), gadodiamide (1 mM) was added to the NMR mixture. Where lipid vesicles were present in the NMR mixture, vesicles were added at a concentration of 0.1 mg/mL. All ^{1}H CPMG NMR spectra were collected with a spin-lock time of 150 mS on a Bruker Avance III spectrometer equipped with a cold probe at a proton frequency of 600 MHz and recorded at 298 K. Peak intensities were measured using MestReNova (Mestrelab Research). The MAF and PF were determined using previously published method.[3] For individual SSAs, MAF and PF were calculated using all aromatic CH resonances. For SSA co-formulations an average MAF and PF were calculated using all aromatic CH resonances. For individual components of the SSA co-formulations, the resonances for the different SSA anions were treated independently of one another.

Section S4: Microbial materials and methods

General remarks: The reference strains, *Pseudomonas aeruginosa* PAO1 and *Candida albicans* SC5314, were used in this study. The strains were stored at −80 °C in nutrient broth (NB) (1 gL^{-1} meat extract, 2 gL^{-1} yeast extract, 5 gL^{-1} peptone, and 8 gL^{-1} sodium chloride) supplemented with 25 and 15 % (v/v) glycerol, respectively. Before each assay, the *P. aeruginosa* and *C. albicans* strains were cultured from the frozen stock onto nutrient agar (NA) (1 gL^{-1} malt extract, 2 gL^{-1} yeast extract, 5 gL^{-1} peptone, 8 gL^{-1} sodium chloride, 20 gL^{-1} agar) and yeast malt extract (YM) agar (3 gL^{-1} malt extract, 3 gL^{-1} yeast extract, 5 gL^{-1} peptone, 10 gL^{-1} glucose). For every experiment, a fresh culture (pre-inoculum) of *P. aeruginosa* was prepared by transferring a single colony from the maintained plates into 5 mL of NB and incubating at 37 °C with shaking (150 rpm (RPM)) for 24 hours. Similarly, a pre-inoculum of *C. albicans* was prepared into 10 mL of YM and incubated at 30 °C for 24 hours. For all assays, filter-sterilized (0.22 µm nitrocellulose filter, ABLUO, GVS, USA) RPMI 1640 medium with l-glutamine and sodium bicarbonate (Sigma-Aldrich, UK) at pH 7.0, was used.

Metabolic assay: The pre-inoculum was washed three times with phosphate-buffered saline (PBS) (0.2 gL^{-1} potassium chloride, 0.2 gL^{-1} potassium dihydrogen phosphate, 1.15 gL^{-1} disodium hydrogen phosphate, 8 gL^{-1} sodium chloride) (Oxoid, UK) at pH 7.3 and standardized to an optical density (OD) OD_{600} of 0.5 for *P. aeruginosa* or 1 × 10^6 cells mL^{-1} for *C. albicans* as described previously.[4–6] The standardized cell suspension was dispensed into a 96-well flat-bottom culture (microtiter) plate (Greiner Bio-One, Germany) (250 µL total volume per well) together with a twofold dilution series of each SSA to achieve a final concentration range of 0.08–2.56 mM. The microtiter plates were incubated for 48 hours at 37 °C and OD measurement (at a wavelength of 595 nm, OD_{595}) (EZ Read 800 Research, Biochrom, England) was performed. To cultivate polymicrobial biofilms, the methods described for the monomicrobial biofilm models were used. However, when standardizing the cell solutions needed for the inoculation of a microtiter plate, *P. aeruginosa* and *C. albicans* cell suspensions were adjusted to an OD_{600} of 0.1 and 2 × 10^6 cells mL^{-1}, respectively. After incubation, an indirect and semiquantitative measure of biofilm formation was performed, using a 2,3-bis(2-methoxy-4-nitro-5-sulfo-phenyl)-2*H*-tetrazolium-5-carboxanilide (XTT) colorimetric reduction assay as described using 1 gL^{-1} of filter-sterilized (0.22 µm nitrocellulose filter) XTT salt (Sigma-Aldrich, UK), dissolved in PBS, and supplemented with 1 mM menadione in acetone. The supernatant from the 96-well plates was discarded, the wells washed twice with 200 µL of PBS before 50 µL of XTT-menadione solution was introduced to each well. The plates were incubated for 3 hours at 37 °C in the dark and the absorbance measured at 492 nm using a microtiter plate reader (EZ Read 800 Research, Biochrom, England). Furthermore, when considering the antimicrobial potential of quaternary ammonium compounds, such as TBA, the counterion of the selected SSAs, the antibiofilm activity of TBA chloride was also evaluated. This was repeated five times. Additionally, appropriate controls were included for known antimicrobials, colistin, and fluconazole, respectively.

Confocal Laser Scanning Microscopy (CLSM): *C. albicans* was cultivated on a glass coverslip in a six-well microtiter plate (Greiner Bio-One, Germany) for 1 hour at 37 °C. After incubation the microscope slide was placed in the confocal microscope (ZEISS LSM 900 with an AiryScan 2 confocal laser scanning microscope (ZEISS, Germany)) and **3** added (2.56 mM). Visualisation occurred for 10 minutes, at an excitation/emission wavelength of 365/461 nm (using the DAPI filter setting)with frames taken every 30 seconds. This was possible due to the inherent fluorescent properties of **3**.

Statistical Analyses: For all quantitative experiments, the averages and standard deviations were calculated. Data produced were analysed using a Student *t* test to establish statistically significant differences between data sets. A *p*-value of ≤0.05 was considered significant.

Section S5: *In vitro* and *in vivo* studies

These studies were performed by Contract Research Organisation (CRO) Pharmaron. The methodology provided within the ESI is published within the express permission of this organisation. The percentage of a compound recovered during the analysis process should be 100 %; any deviation from this value indicates experimental limitations such as unintended potential non-specific binding events to experimental equipment, solubility issues, etc.

In vitro Absorption, Distribution and Metabolism studies methods:

Protein Binding measurements in human plasma by using equilibrium dialysis:

The fraction unbound in human plasma (P-KB, Ltd People's hospital of Shandong) was measured using 96-well Equilibrium Dialysis Plate (HTDialysis LLC, Gales Ferry, CT). Human plasma containing 5 µM test compound was dialysed against dialysis buffer (PBS, pH 7.4) with initial samples taken to allow for assessment of plasma stability and recovery before the plate was incubated for 6 hours at 37°C at 5% CO_2. Samples were removed at 6 hours, and all samples were matrix matched and quenched with acetonitrile containing internal standard. Samples were centrifuged at 3,220g for 30 minutes to precipitate protein and enable sampling of supernatant prior to appropriate dilution for liquid chromatography mass-spectrometry (LC-MS/MS) analysis.

Samples were analysed using a Shimadzu Nexera LC Binary Gradient LC-30AD (Shimadzu Inc, Kyoto), coupled to a Sciex 6500+ (AB Inc., Illinois). Separation was achieved using a XSelect HSS T3 (2.5µm, 2.1 x 50 mm) (Waters Ltd., Wilmslow) column at a temperature of 40°C and a binary mobile phase gradient at a flow rate of 0.8 mL/min. Initial LC conditions comprised of 95% solvent A (0.1% formic acid in water), 5% solvent B (0.1% formic acid in acetonitrile); this was ramped to 98% B at 0.5 min, held for 0.4 min and then immediately returned to initial conditions. Sample analysis was by electrospray ionisation in combined with multiple reaction monitoring in positive ionisation mode. The ionisation voltage was +5500 V.

Kinetic Solubility Determination in PBS pH 7.4:

The stock solutions of test and control compounds were prepared in DMSO at 10 mM. From this a 300 µM solution was prepared in duplicate in 100 mM Phosphate Buffered Saline (PBS, pH 7.4 ± 0.1). These solutions were then shaken at 1100rpm for 2 hours at 25°C. Following incubation, the samples were filtered using a vacuum manifold. The filtrate was analysed and quantified against a standard of known concentration using liquid chromatography mass-spectrometry (LC-MS/MS).

Samples were analysed using a Shimadzu Nexera LC Binary Gradient LC-30AD (Shimadzu Inc, Kyoto), coupled to a Sciex 4500 mass spectrometer (AB Inc., Illinois) Separation of compounds was achieved using an XSelect HSS T3 column (2.5µm, 50 x 2.1 mm) (Waters Ltd., Wilmslow) at a temperature of 40°C and a binary mobile phase gradient at a flow rate of 0.8 mL/min. Initial LC conditions comprised of 95% solvent A (0.1% formic acid in water) and 5% solvent B (0.1% formic acid in acetonitrile). This was ramped to 98% solvent B at 0.5 minutes, held for 0.4 minutes and then immediately returned to initial conditions. Sample analysis was by electrospray ionisation combined with multiple reaction monitoring in positive ion mode. The ionspray voltage was 5500V.

Metabolic Stability in Rat, Mouse and Human Liver Microsomes:

The metabolic stability of compounds at a concentration of 1 µM, in rat, mouse and human liver microsomes (BD Gentest, Franklin Lakes NJ) was measured following incubation with hepatic microsomes (0.5 mg of protein / mL) in the presence and absence of cofactor (NADPH) at 37 °C. Aliquots of each incubation were removed at 0.5, 5, 15, 30 and 60 minutes. The reaction was stopped by the addition of cold acetonitrile containing analytical internal standard (IS).

Samples were then centrifuged at 3,220g for 40 minutes. The supernatant was aliquoted and added to ultra-pure H_2O in a 1:1 ratio in preparation for LC-MS/MS analysis. Incubations were conducted in duplicate; control incubations (substituting PBS for NADPH) were conducted simultaneously. All control incubations were within acceptance criteria in mouse, rat, and human microsomes confirming stability was acceptable over 60 minutes in the absence of co-factor.

Samples were analysed using a Shimadzu Nexera LC Binary Gradient LC-30AD (Shimadzu Inc, Kyoto), coupled to a Sciex 5500 mass spectrometer (AB Inc., Illinois) for rat and mouse microsomes and a Sciex 6500+ for human microsomes. Separation achieved using an XSelect HSS T3 column (2.5µm, 50 x 2.1 mm) (Waters Ltd., Wilmslow) at a temperature of 40°C and a binary mobile phase gradient at a flow rate of 0.7 mL/min. Initial LC conditions comprised of 95% solvent A (0.1% formic acid in water) and 5% solvent B (0.1% formic acid in acetonitrile). This was ramped to 95% solvent B at 0.7 minutes, held for 0.5 minutes and then immediately returned to initial conditions. Sample analysis was by electrospray ionisation combined with multiple reaction monitoring in positive ion mode. The ionspray voltage was 5500V.

Bi-directional Permeability in Caco-2 Cell Line:

The bi-directional permeability in Caco-2 cell lines was determined by seeding cells onto an HTS Transwell plate. Cells were cultivated for 14-18 days in a cell culture incubator at 37°C, 5% CO_2, 95% relative humidity. Cell culture medium was replaced every other day. To assess monolayer integrity the transepithelial electrical resistance (TEER) across the monolayer was measured using Millicell Epithelial volt-ohm meter prior to assay and Lucifer yellow (LY) fluorescence measurement post-assay.

Test compound was prepared in DMSO and diluted in Hank's Balanced Saline Solution (HBSS, pH 7.4) to a final concentration of 5 µM. Compound was added to the donor compartment of the transwell plate for both apical to basolateral (A>B) and basolateral to apical (B>A) measurements, with blank HBSS being added to the receiver compartment. The transwell plate was then incubated at 37°C, 5% CO_2 for 2 hours.

Samples were shaken at T=0 and T=2 hours to determine the concentration of initial donor sample, and the concentration of receiver and donor wells post-incubation, respectively. The samples were quenched with solvent containing internal standard (IS). The supernatant was mixed with an appropriate volume of ultra-pure water before LC-MS/MS analysis.

Samples were analysed using a Shimadzu Nexera LC Binary Gradient LC-30AD (Shimadzu Inc, Kyoto), coupled to a Sciex 5500+ (AB Inc., Illinois). Separation of compounds was achieved using an XSelect HSS T3 XP (2.5µm,2.1x50mm) (Waters Ltd., Wilmslow) column at a temperature of 40°C. A binary mobile phase gradient at a flow rate of 0.7 mL/min was used. Initial LC conditions comprised of 95% solvent A (0.1% formic acid in water) and 5% solvent B (0.1% formic acid in acetonitrile). This was ramped to 95% solvent B at 1.2 minutes, held for 0.4 minutes and then immediately returned to initial conditions. Sample analysis was by electrospray ionisation combined with multiple reaction monitoring in positive ion mode. The ionspray voltage was 5500V.

In vitro Absorption, Distribution and Metabolism results

The summary of *in vitro* results is shown in Table S4-Table S7.

Table S4 - Result from solubility assays performed in PBS pH 7.4 for SSA 5.

ID	Solubility (μM)
SSA 5	273

Table S5 - Summary of results from Caco-2 permeability assays for SSA 5. P_{app} is given as x10^{-6} cm/s. Low P_{app} = < 5 x10^{-6} cm/s; Moderate P_{app} = 5-10 x10^{-6} cm/s; High P_{app} = > 10 x10^{-6} cm/s. A = apical; B = basolateral. Recovery = % SSA recovered from the assay after 2 hours.

ID	P_{app} (A-B)	% Recovery	P_{app} (B-A)	% Recovery	Efflux Ratio
SSA 5	< 0.06	< 98.4	4.24	82.6	> 73.9

Table S6 - Summary of results from human plasma protein binding assays for SSA 5. % Bound = % of SSA bound to the plasma proteins after 6 hours incubation at 37 °C. Low % Bound = < 80 %; Moderate % Bound = 80-95 %; High % Bound = 95-99 %; Very high % Bound = > 99 %. % Recovery = % SSA recovered from the assay after 6 hours.

ID	% Bound	% Recovery	% Stability
SSA 5	92.4	101.6	99.5

Table S7 - Summary of results from mouse, rat and human microsomal stability assays for SSA 5. Low ER = < 0.3; Moderate ER = 0.3-0.7; High ER = > 0.7.

	Mouse		Rat		Human	
ID	In vitro $T_{1/2}$ (min)	Hepatic Extraction Ratio (ER)	In vitro $T_{1/2}$ (min)	Hepatic Extraction Ratio (ER)	In vitro $T_{1/2}$ (min)	Hepatic Extraction Ratio (ER)
SSA 5	> 185	< 0.26	> 185	< 0.21	> 185	< 0.27

In vivo Pharmacokinetic study methods

SSA **5** was dosed at 1 mg/kg to female CD-1 mice (n=3) via an intravenous tail vein bolus administration, dose formulation was a solution in 2% DMSO/98% Water. Blood was sampled from the dorsal metatarsal vein at each time point (n=8) and added to tubes containing anticoagulant K2-EDTA. Blood samples were extracted by protein precipitation. Blood concentrations were calculated through UPLC-MS/MS techniques, with internal standard. The lower limit of quantitation was 6.4 nM. PK parameters were calculated using Phoenix WinNonlin version 8.3.

In vivo Pharmacokinetic study results

Table S8 – Summary of *in vivo* pharmacokinetic study results.

**Route**	IV
**Formulation**	DMSO:H_2O 2:98
**Dose**	1 mg/kg
**C_{max} (nM)**	1196 ± 208
**T_{max} (h)**	0.083
**AUC_{last} (h*nM)**	327 ± 57
**AUC_{INF} (h*nM)**	335 ± 60
**Cl Blood (mL/min/kg)**	162 ± 28 (>100 % LBF)
**$t_{1/2}$ (h)**	0.73 ± 0.61
**V_{ss} (L/kg)**	3.1 ± 1.7

SSA **5** is a very high blood cleared compound (162 ± 28 mL/min/kg, >100% LBF) with a moderate volume of distribution (3.1 ± 1.7 L/kg) following IV administration to female CD-1 mice formulated in DMSO:H_2O 2:98.

IV administration of **5** at 1 mg/kg gave a mean blood C_{max} of *ca.* 1196 ± 208 nM at 5 minutes post-dose and levels of **5** were detected up to 2 hours following dosing (8.1 nM). The blood AUC_{INF} was 335 ± 60 h*nM and the terminal half-life following intravenous administration was short at 0.73 ± 0.61 h.

Section S6: Chemical synthesis

Compound 1: Aminopropansulfonic acid (0.278 g, 2.00 mmol) was dissolved in tetrabutylammonium in methanol (2.00 mL, 2.00 mmol) with excess methanol (15 mL) to aid dissolving before being taken to complete dryness. Tetrabutylammonium aminopropansulfonate (2.00 mmol) was then dissolved in ethyl acetate (20 mL) and 1-isothiocyanato-4-(trifluoromethyl)benzene (0.406 g, 2.00 mmol) was added and stirred overnight at room temperature. The crude precipitate was filtered and rinsed with ethyl acetate (5 mL) resulting in the pure product as a white solid (0.728 g, 1.25 mmol, 63.3 %); ^{1}H NMR (400 MHz, 298 K, DMSO-d_6): 0.93 (t, J = 7.28 Hz, 12H), 1.26 – 1.35 (m, 8H), 1.53 – 1.60 (m, 8H), 1.81 – 1.88 (m, 2H), 2.48 (s, 2H), 3.14 – 3.18 (m, 8H), 3.56 (d, J = 3.92 Hz, 2H), 7.62 (d, J = 8.52 Hz, 2H), 7.76 (d, J = 7.32 Hz, 2H), 8.19 (s, 1H), 9.89 (s, 1H).[7]

Compound 2: Glycine (0.150 g, 2.00 mmol) was dissolved in dH_2O (5 mL) and tetrabutylammonium hydroxide (2.00 mL, 2.00 mmol) was added. The resultant mixture was taken to dryness, with the aid of toluene (10 mL). Glcyinate tetrabutylammonium was then dissolved in ethyl acetate (20 mL) and 1-isothiocyanato-4-(trifluoromethyl)benzene (0.406 g, 2.00 mmol) was added and stirred overnight at room temperature. The crude precipitate was filtered and rinsed with ethyl acetate (5 mL) resulting in the pure product as a white solid (0.670 g, 1.29 mmol, 64.5 %); ^{1}H NMR (400 MHz, 298 K, DMSO-d_6): 0.92 (t, J = 7.28 Hz, 12H), 1.25 – 1.35 (m, 8H), 1.52 – 1.59 (m, 8H), 3.13 – 3.17 (m, 8H), 3.66 (s, 2H), 7.57 (d, J = 8.56 Hz, 2H), 8.20 (d, 8.28 Hz, 2H), 8.49 (s, 1H), 11.66 (s, 1H).[7]

Compound 3: Aminomethansulfonic acid (0.222 g, 2.00 mmol) was dissolved in tetrabutylammonium in methanol (2.00 mL, 2 mmol) with excess methanol (15 mL) to aid dissolving before being taken to complete dryness. Tetrabutylammonium aminomethansulfonate (2.00 mmol) was then dissolved in ethyl acetate (20 mL), 4-(6-methylbenzo[d]thiazol-2-yl)aniline (0.480 g, 2 mmol) was added and the mixture was stirred at room temperature for 24 hours. The crude precipitate was filtered and rinsed with ethyl acetate (5 mL), then dissolved in chloroform (15 mL) and washed with water (15 mL x 3), and taken to dryness. Ethyl acetate (20 mL) was then added to the organic oil and sonicated for 30 minutes, resulting in a precipitate which was filtered and washed with ethyl acetate (5 mL) resulting in the pure product as a white solid (0.974 g, 1.57 mmol, 78.6 %). ^{1}H NMR (400 MHz, 298 K, DMSO-d_6): 0.93 (t, J = 7.28 Hz, 12H), 1.25 – 1.35 (m, 8H), 1.52 – 1.59 (m, 8H), 2.44 (s, 3H), 3.13 – 3.17 (m, 8H), 3.92 (d, J = 5.80 Hz, 2H), 6.75 (s, 1H), 7.31 (d, J = 8.36 Hz, 1H), 7.56 (d, J = 8.72 Hz, 2H), 7.85 – 7.92 (m, 4H), 9.18 (s, 1H).[8]

Compound 4: Aminopropansulfonic acid (0.278 g, 2.00 mmol) was dissolved in tetrabutylammonium in methanol (2.00 mL, 2 mmol) with excess methanol (15 mL) to aid dissolving before being taken to complete dryness. Tetrabutylammonium aminopropansulfonate (2.00 mmol) was then dissolved in ethyl acetate (20 mL), **9** (0.271 g, 2.00 mmol) was added and the mixture was stirred at room temperature for 24 hours. The crude precipitate was filtered and rinsed with ethyl acetate (5 mL) resulting in the pure product as a white solid (0.906 g, 1.46 mmol, 87 %); melting point: 161 °C; ^{1}H NMR (400 MHz, 298 K, DMSO-d_6): 0.92 (t, 12H, J = 7.40 Hz), 1.25 – 1.34 (m, 8H), 1.52 – 1.60 (m, 8H), 1.94 (t, 2H, J = 6.88 Hz), 2.66 (t, 2H, 7.64 Hz), 3.13 – 3.18 (m, 8H), 3.72 (s, 2H), 7.63 – 7.72 (m, 4H), 8.14 (s, 0.8H), 8.71 (s, 0.1H), 10.23 (s, 1H); ^{13}C{^{1}H} NMR (400 MHz, 298 K, DMSO-d_6): 13.3 (CH_3), 19.1 (CH_2), 22.9 (CH_2), 26.7 (CH_2), 42.6 (CH_2), 48.0 (CH_2), 57.4 (CH_2), 117.9 (ArCH), 122.1 (q, J_1 = 128.0 Hz, ArC), 124.4 (q, J_1 = 1096.0 Hz, CF_3), 126.3 (q, J_1 = 12.0 Hz, ArCH), 142.8 (ArC), 163.0 (ArC), 169.6 (ArC), 179.9 C=O, 184.5 C=O; IR (film): ν = 1788 (C=O stretch), 1690, 1612, 1589, 1420, 1034; HRMS for the carboxylate-urea ion ($C_{14}H_{16}F_3N_2O_3^-$); HRMS for the anionic component of **4** ($C_{14}H_{12}F_3N_2O_5S^-$) (ESI$^-$): m/z: measured = 377.0416 $[M]^-$, predicted = 377.0425 $[M]^-$.

Compound 5: Glycine (0.150 g, 2.00 mmol) was dissolved in dH_2O (5 mL) and tetrabutylammonium hydroxide (2.00 mL, 2.00 mmol) was added. The resultant mixture was taken to dryness, with the aid of toluene (10 mL). Glcyinate tetrabutylammonium was then dissolved in ethyl acetate (20 mL) and **9** (0.271 g, 2.00 mmol) was added and the mixture was stirred at room temperature for 24 hours. The crude precipitate was filtered and rinsed with ethyl acetate (5 mL) resulting in the pure product as a white solid. (0.523 g, 0.942 mmol, 47 %); melting point: <200 °C; ^{1}H NMR (400 MHz, 298 K, DMSO-d_6): 0.91 (t, *J* = 7.28 Hz, 12H), 1.24 – 1.33 (m, 8H), 1.51 – 1.59 (m, 8H), 3.12 – 3.17 (m, 8H), 4.13 (s, 2H), 7.58 (d, *J* = 8.72 Hz, 2H), 8.28 (d, *J* = 8.44 Hz, 2H), 8.96 (s, 1H), 13.0 (s, 1H); ^{13}C{^{1}H} NMR (400 MHz, 298 K, DMSO-d_6): 13.4 (CH_3), 19.2 (CH_2), 23.0 (CH_2), 48.1 (CH_2), 57.5 (CH_2), 118.8 (ArCH), 121.5 (q, J_1 = 128.3 Hz, ArC), 124.7 (q, J_1 = 1077.2 Hz, CF_3), 126.0 (q, J_1 = 14.6 Hz, ArCH), 144.2 (ArC), 163.5 (ArC), 168.8 (ArC), 170.5 (C=O), 180.4 (C=O), 185.1 (C=O); IR (film): ν = 3219, 1788, (C=O stretch), 1680, 1636, 1589, 1437, 1107; HRMS for the anionic component of **5** ($C_{13}H_8F_3N_2O_4^-$) (ESI$^-$): m/z: measured = 313.0441 [M]$^-$, predicted = 313.0442 [M]$^-$.

Compound 9: A mixture of 4-(trifluoromethyl)analine (0.88 mL, 7.00 mmol) and 3,4-dimethoxycyclobut-3-ene-1,2-dione was stirred at room temperature in MeOH (15 mL) for 48 hours. The pure product was isolated by filtration as a pale yellow solid (1.24 g, 5.25 mmol, 75.2 %). ^{1}H NMR (400 MHz, 298 K, DMSO-d_6): 4.40 (s, 3H), 7.55 (d, *J* = 8.44 Hz, 2H), 7.71 (d, *J* = 8.52 Hz, 2H) 11.01 (s, 1H).[9]

Section S7: Single Crystal XRD data

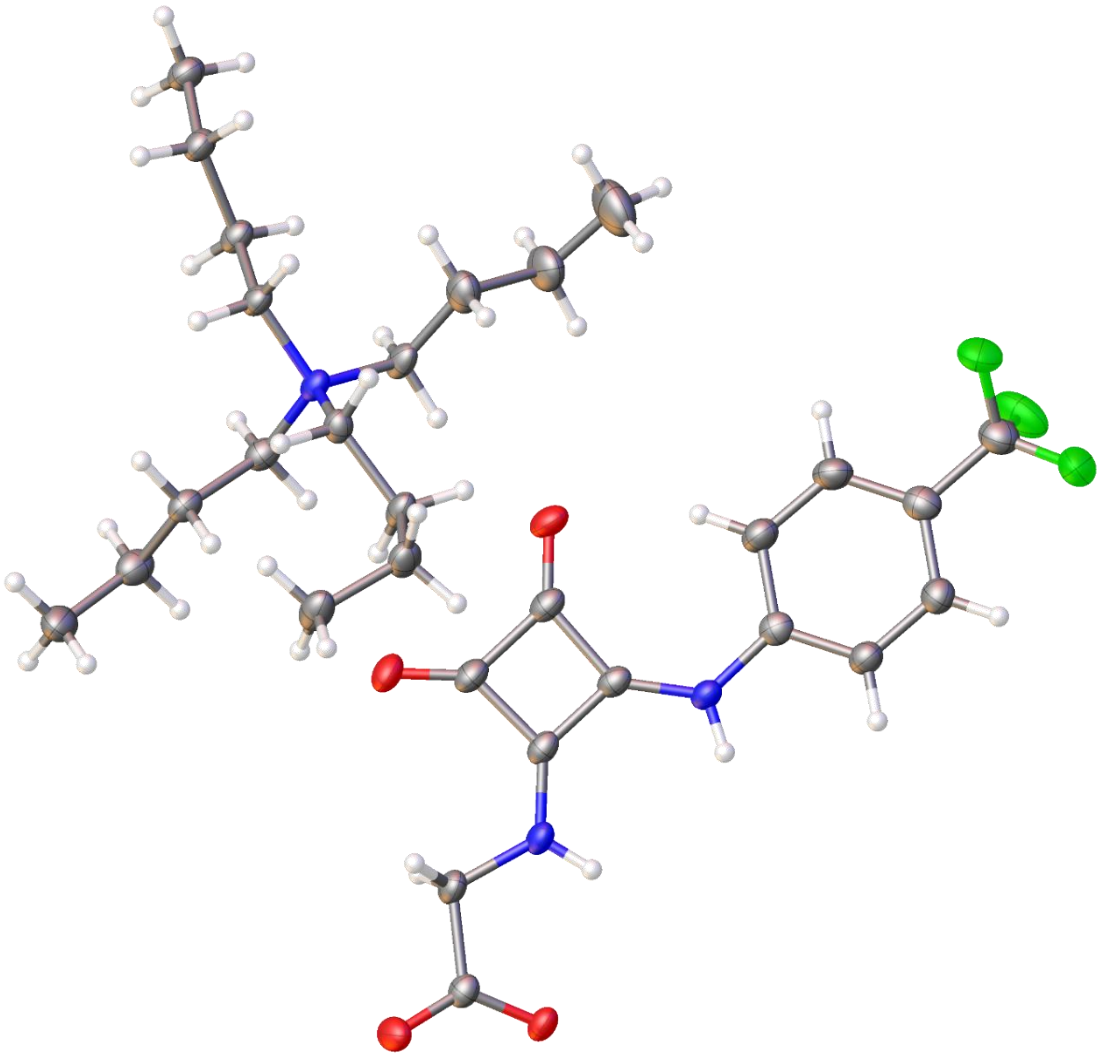

Figure S2 – Single crystal X-ray structure of **5**: red = oxygen; green = fluorine; blue = nitrogen; white = hydrogen; grey = carbon. CCDC 2387938, $C_{29}H_{44}F_3N_3O_4$ (M = 555.67): triclinic, space group P -1, a = 8.7142(4) Å, b = 10.4256(4) Å, c = 16.5504(7) Å, α = 82.895(3)°, β = 85.060(3)°, γ = 89.453(3)°, V = 1486.53(11) Å^3, Z = 2, T = 150(1) K, CuK\α = 1.5418 Å, Dcalc = 1.241 g/cm^3, 9752 reflections measured (8.548 ≤ 2Θ ≤ 133.188), 5247 unique (R_{int} = 0.0401, R_{sigma} = 0.0498) which were used in all calculations. The final R_1 was 0.0497 (I > 2σ(I)) and wR_2 was 0.1388 (all data). Internal angel of dimerization = 180.0(3)°.

Table S9 – Hydrogen bond distances and angles observed for **5**, calculated from the single crystal X-ray structure shown in Figure S2.

Hydrogen bond donor	Hydrogen atom	Hydrogen bond acceptor	Hydrogen bond length (D•••A) (Å)	Hydrogen bond angle (D-H•••A) (°)
N1	H1	O2	2.893(2)	161.42(9)
N2	H2	O2	2.779(2)	165.20(11)

Section S8: NMR spectroscopy data

Molecular characterisation data

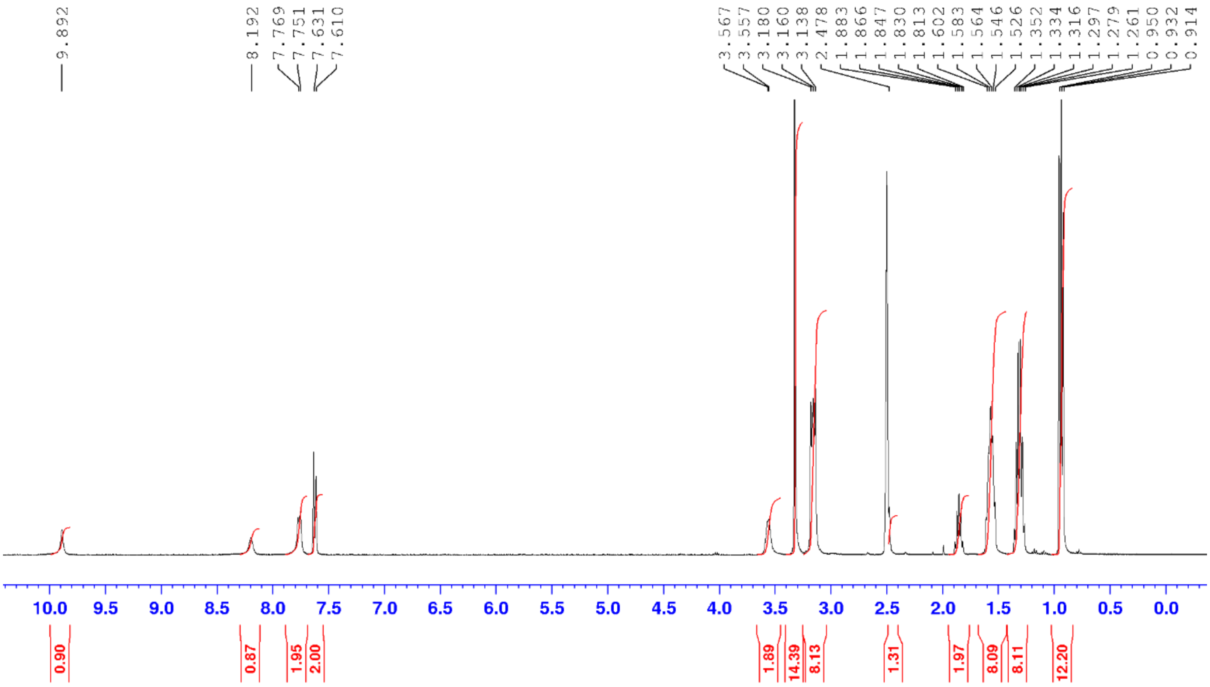

Figure S3 – ^{1}H NMR spectrum of **1** in DMSO-d_6 conducted at 298 K.

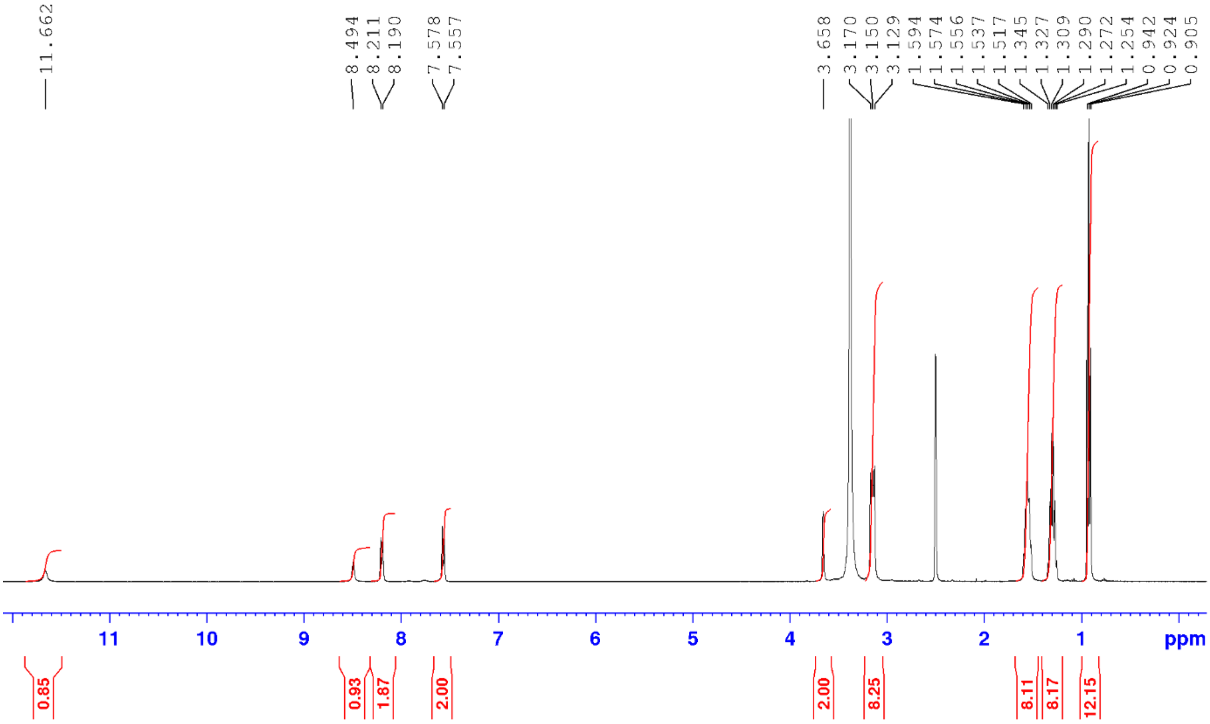

Figure S4 – ^{1}H NMR spectrum of **2** in DMSO-d_6 conducted at 298 K.

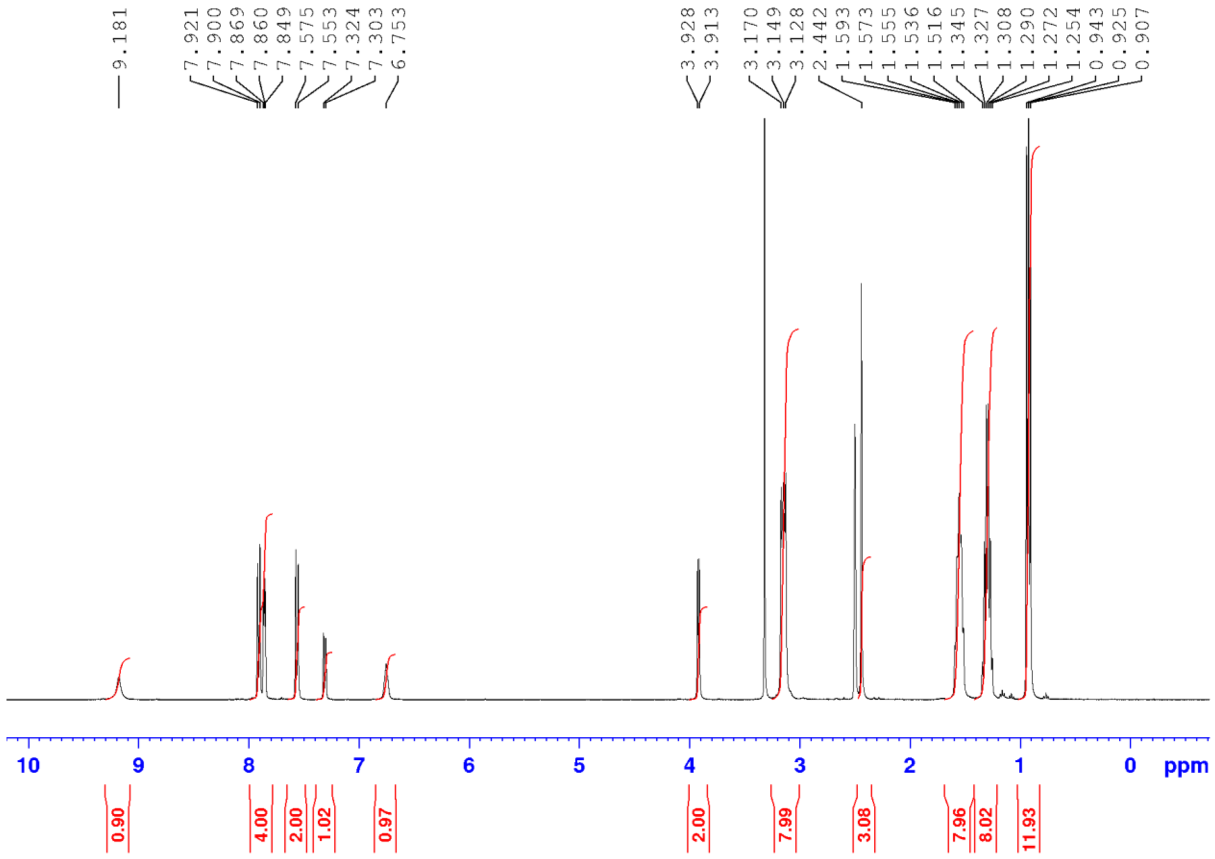

Figure S5 – ^{1}H NMR spectrum of **3** in DMSO-d_6 conducted at 298 K.

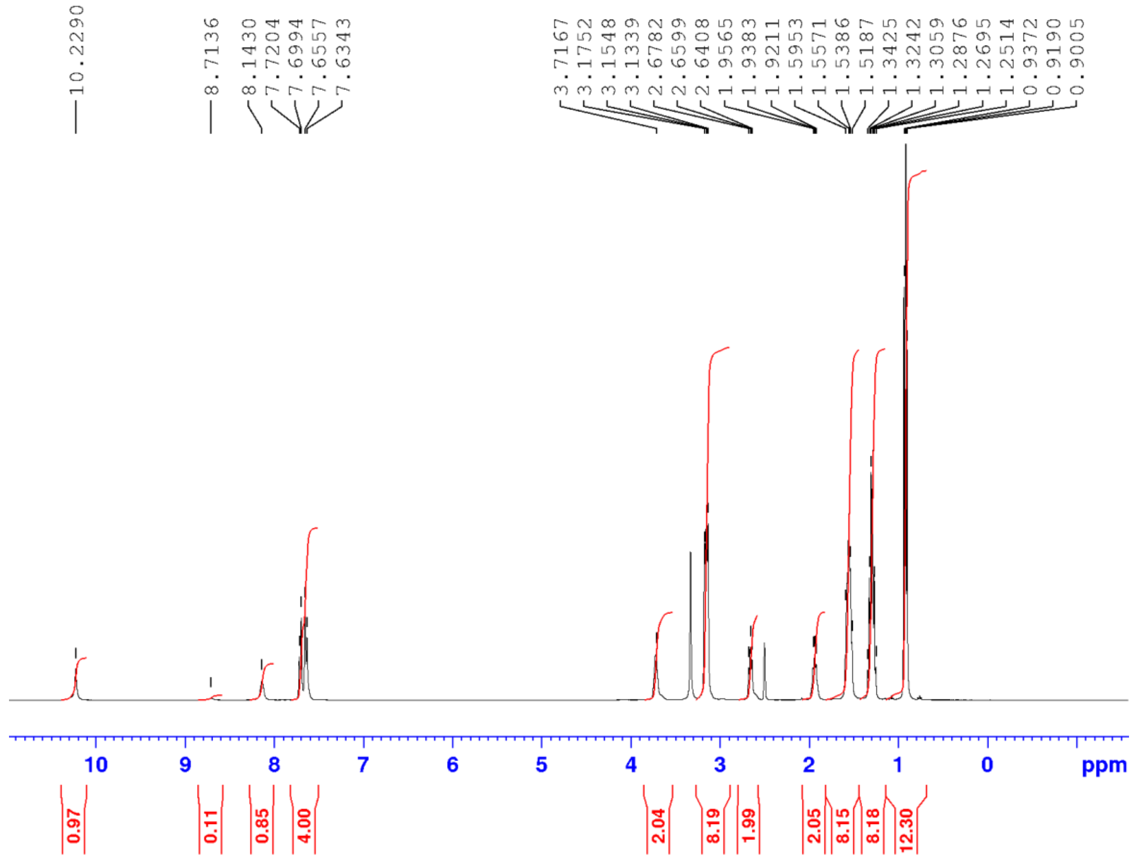

Figure S6 – ^{1}H NMR spectrum of **4** in DMSO-d_6 conducted at 298 K.

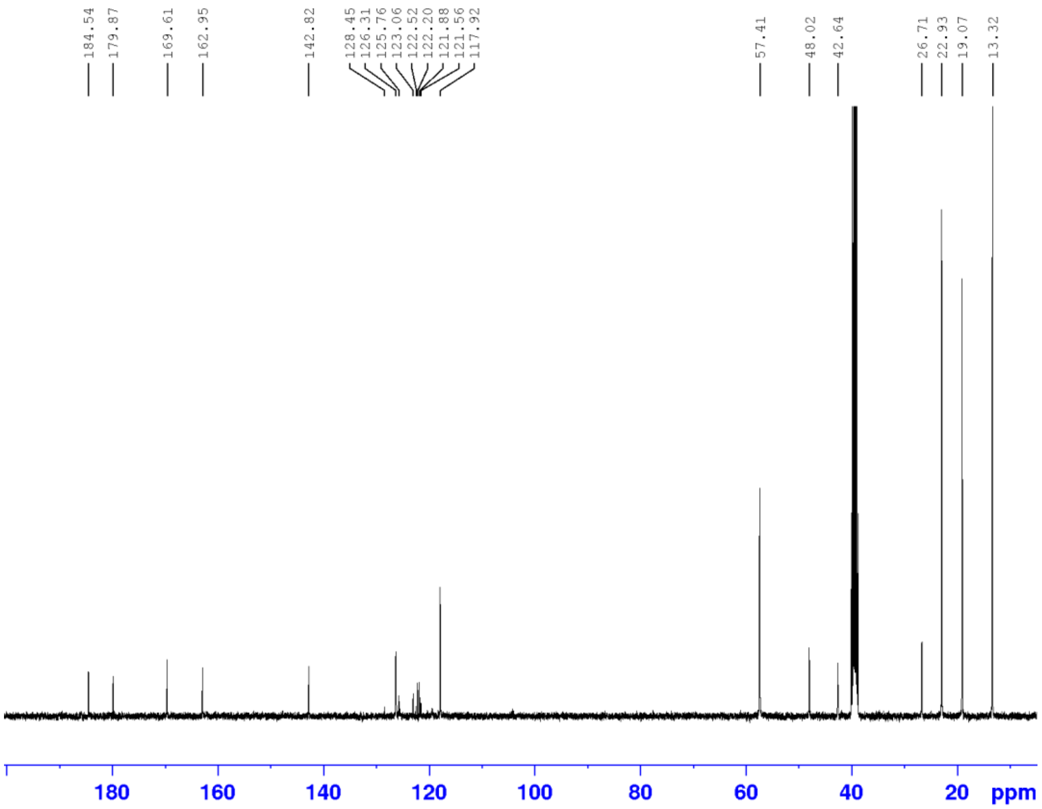

Figure S7 – $^{13}C\{^{1}H\}$ NMR spectrum of **4** in DMSO-d_6 conducted at 298 K.

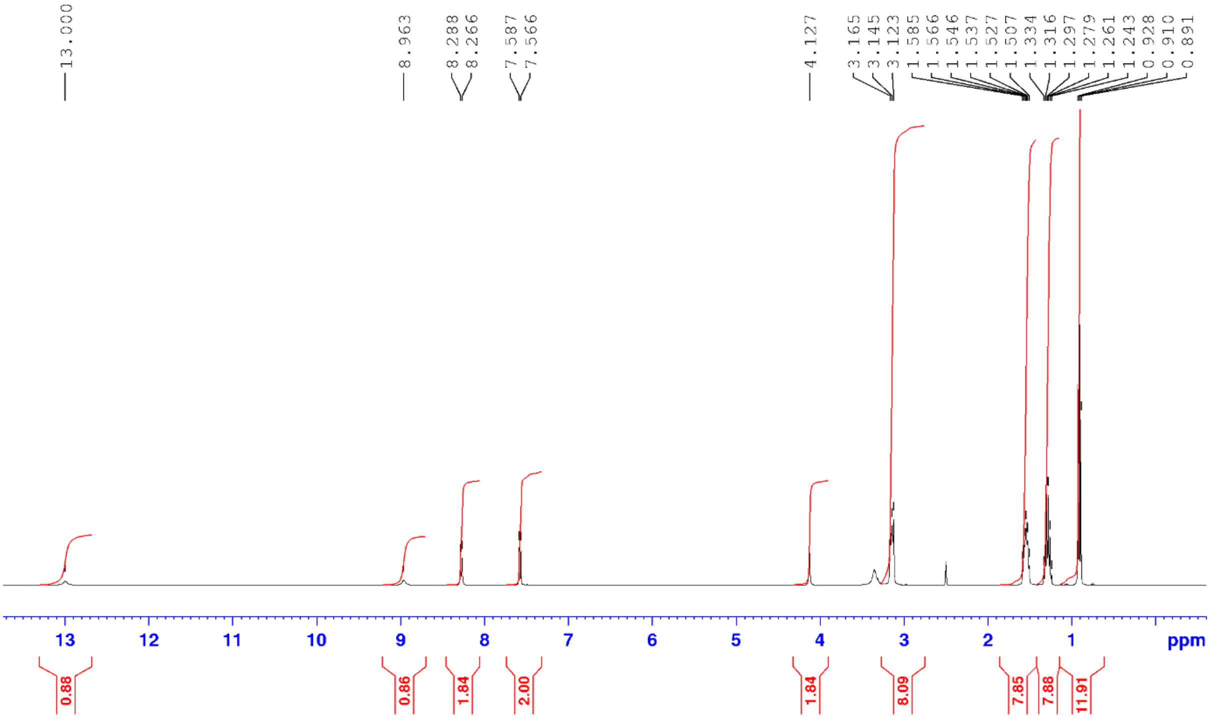

Figure S8 – ^{1}H NMR spectrum of **5** in DMSO-d_6 conducted at 298 K.

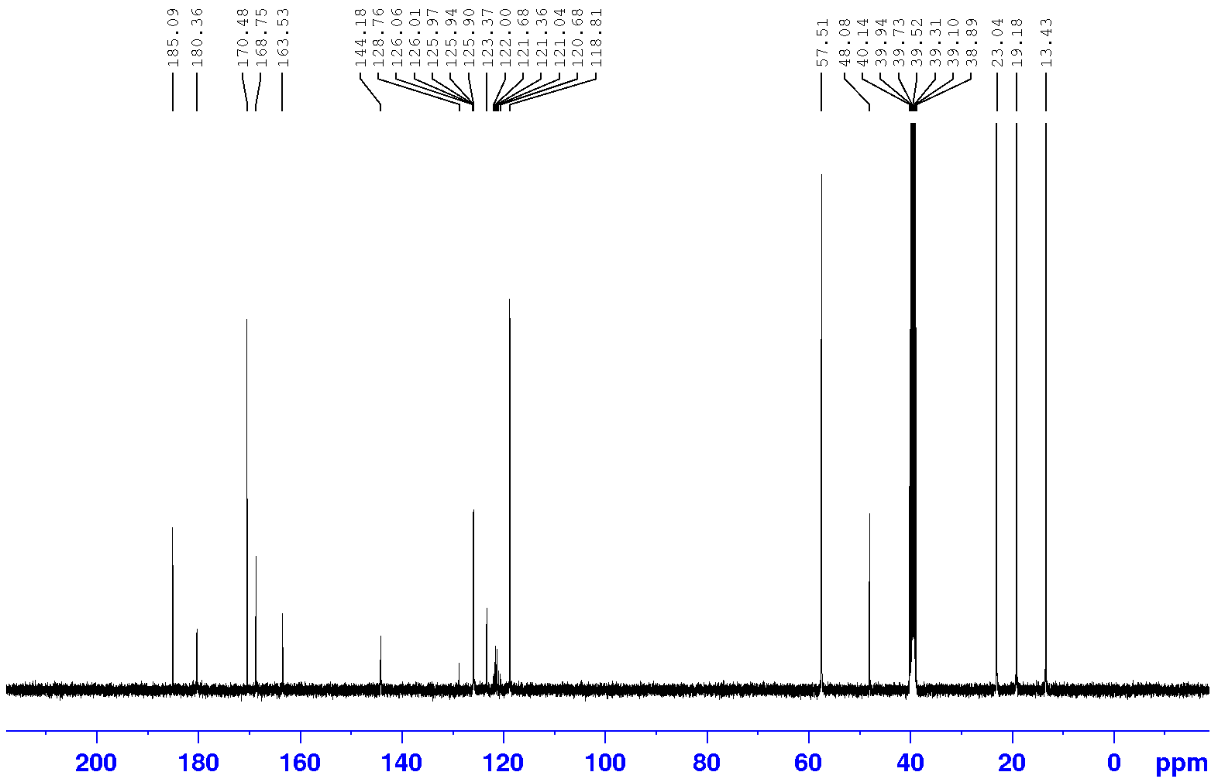

Figure S9 – $^{13}C\{^{1}H\}$ NMR spectrum of **5** in DMSO-d_6 conducted at 298 K.

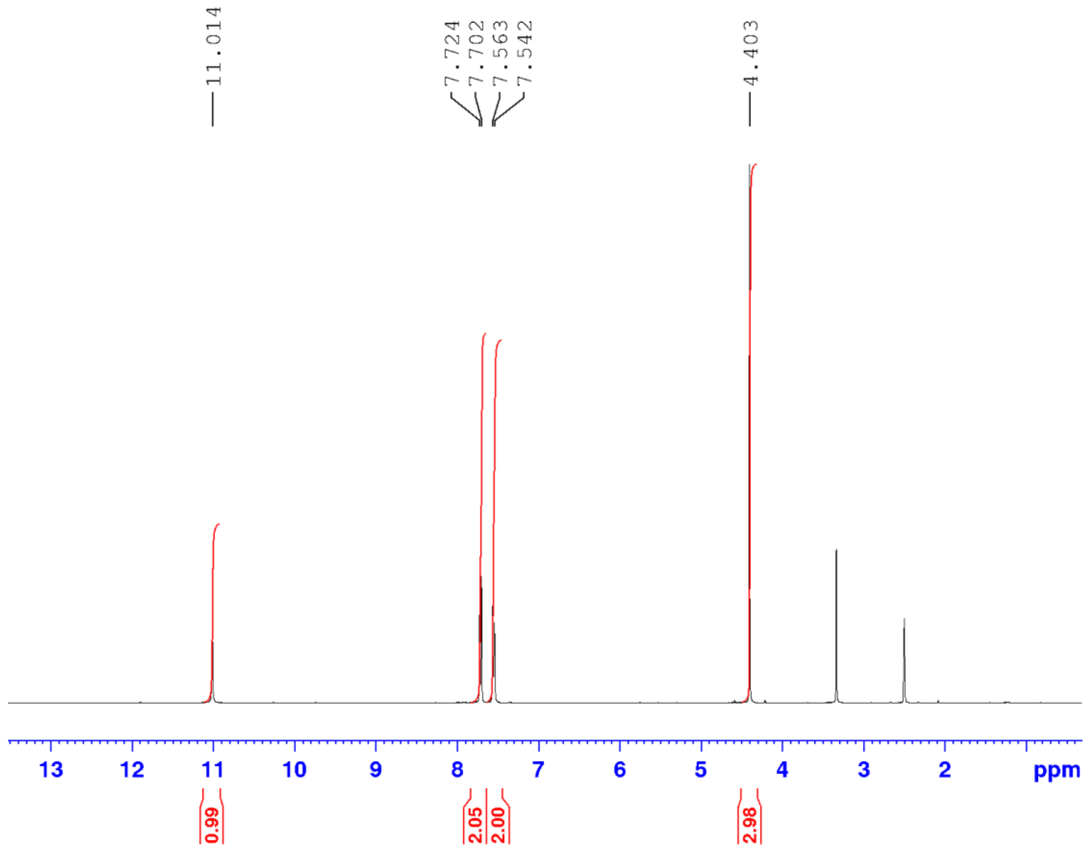

Figure S10 – ^{1}H NMR spectrum of **9** in DMSO-d_6 conducted at 298 K.

^{1}H qNMR spectroscopy data

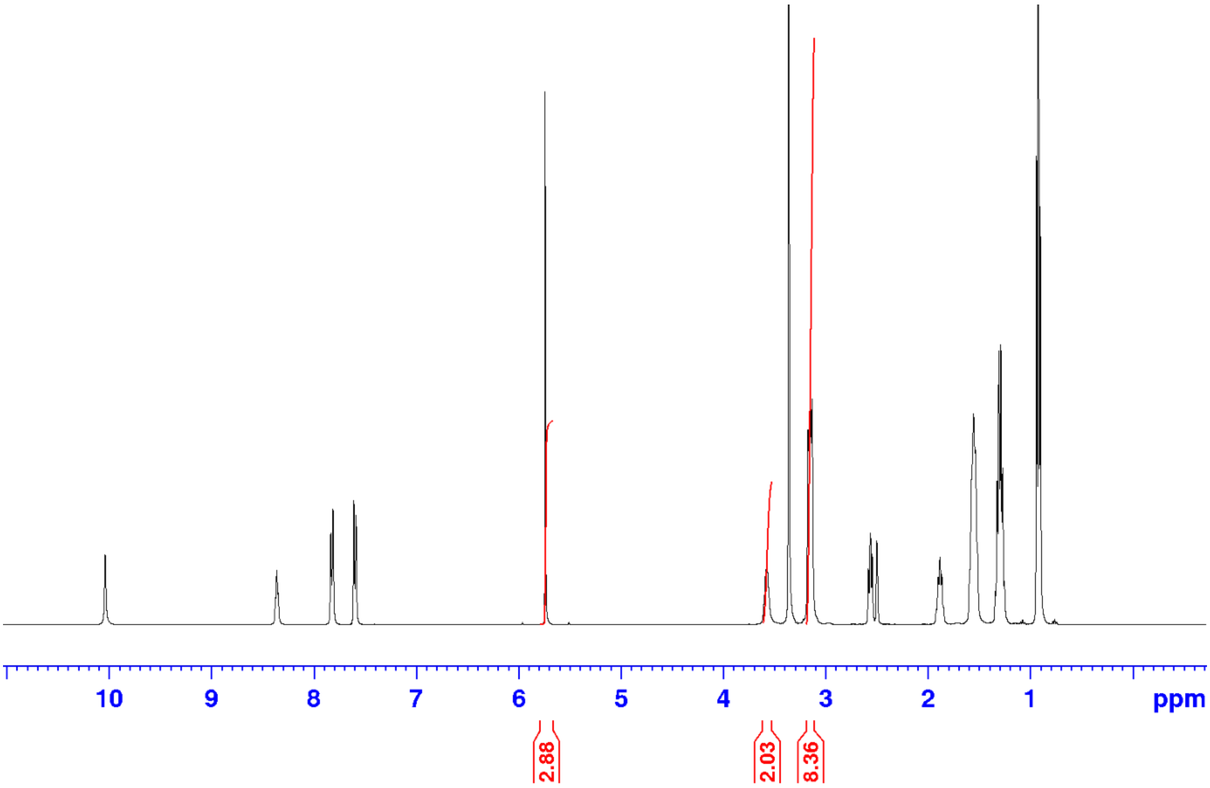

Figure S11 – ^{1}H NMR spectrum (d_1 = 60 s) of **1** (112.12 mM) in DMSO-d_6/1.0 % DCM. Comparative integration indicates 0 % of the anionic and cationic component of **1** has become NMR silent.

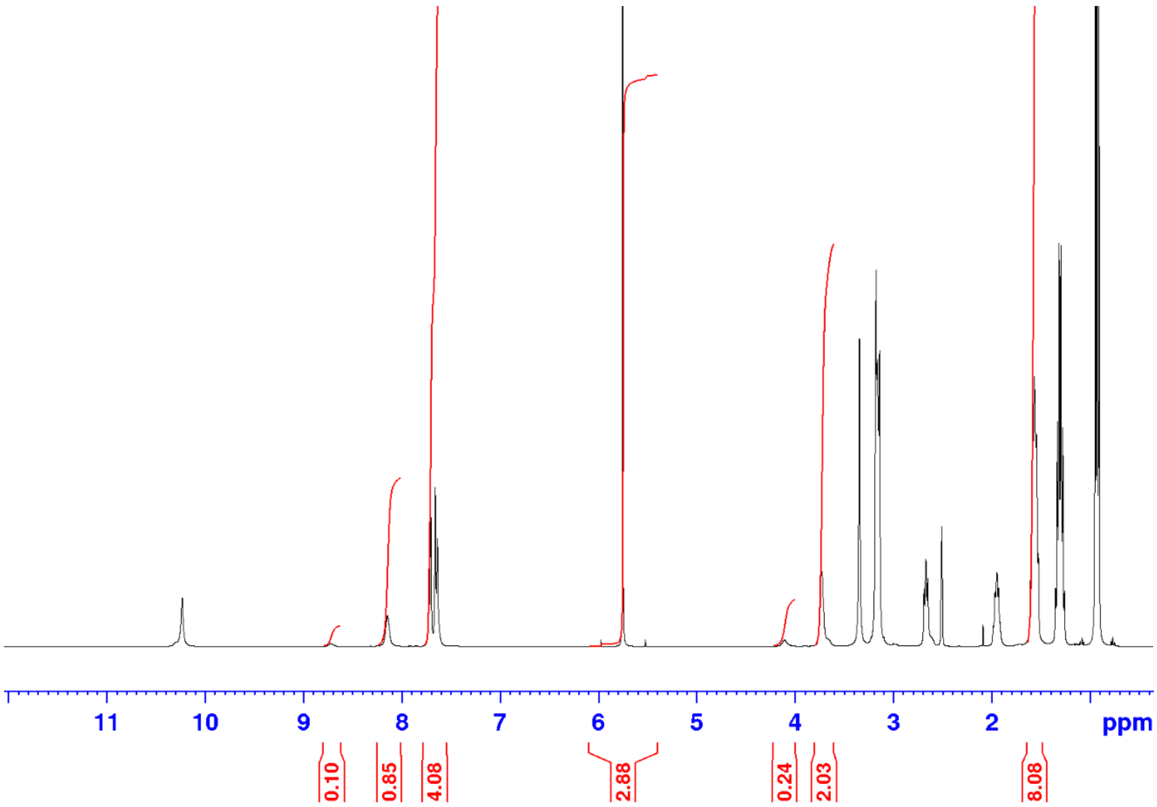

Figure S12 – ^{1}H NMR spectrum (d_1 = 60 s) of **4** (112.12 mM) in DMSO-d_6/1.0 % DCM. Comparative integration indicates 0 % of the anionic and cationic component of **4** has become NMR silent.

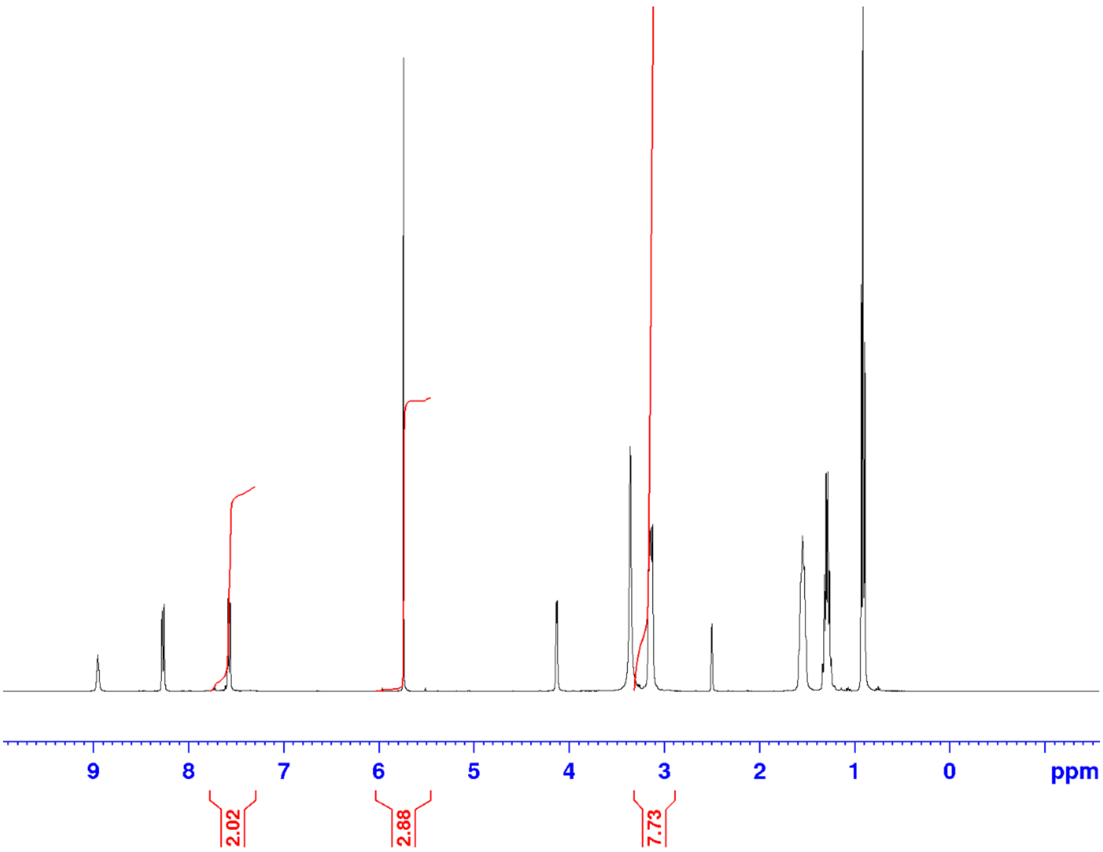

Figure S13 – ^{1}H NMR spectrum (d_1 = 60 s) of **5** (112.12 mM) in DMSO-d_6/1.0 % DCM. Comparative integration indicates 0 % of the anionic and cationic component of **5** has become NMR silent.

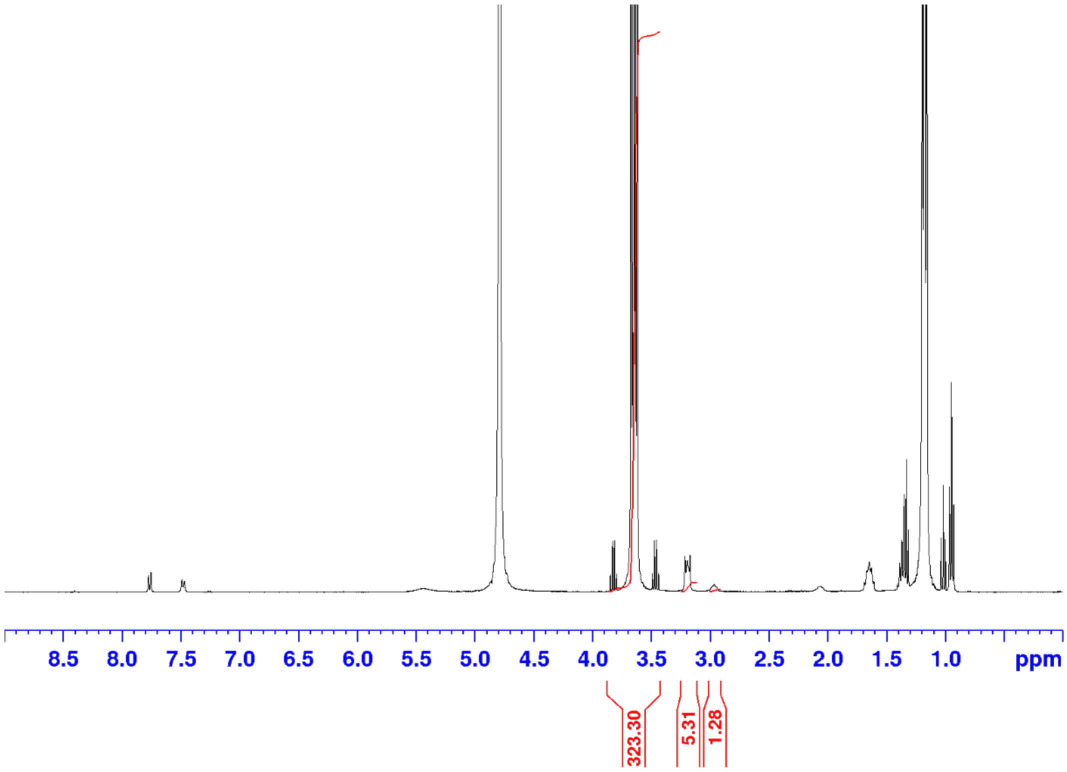

Figure S14 – ^{1}H NMR spectrum (d_1 = 60 s) of **1** (2.78 mM) in D_2O/5.0 % EtOH. Comparative integration indicates 33 % of the anionic and 31 % of the cationic component of **1** has become NMR silent.

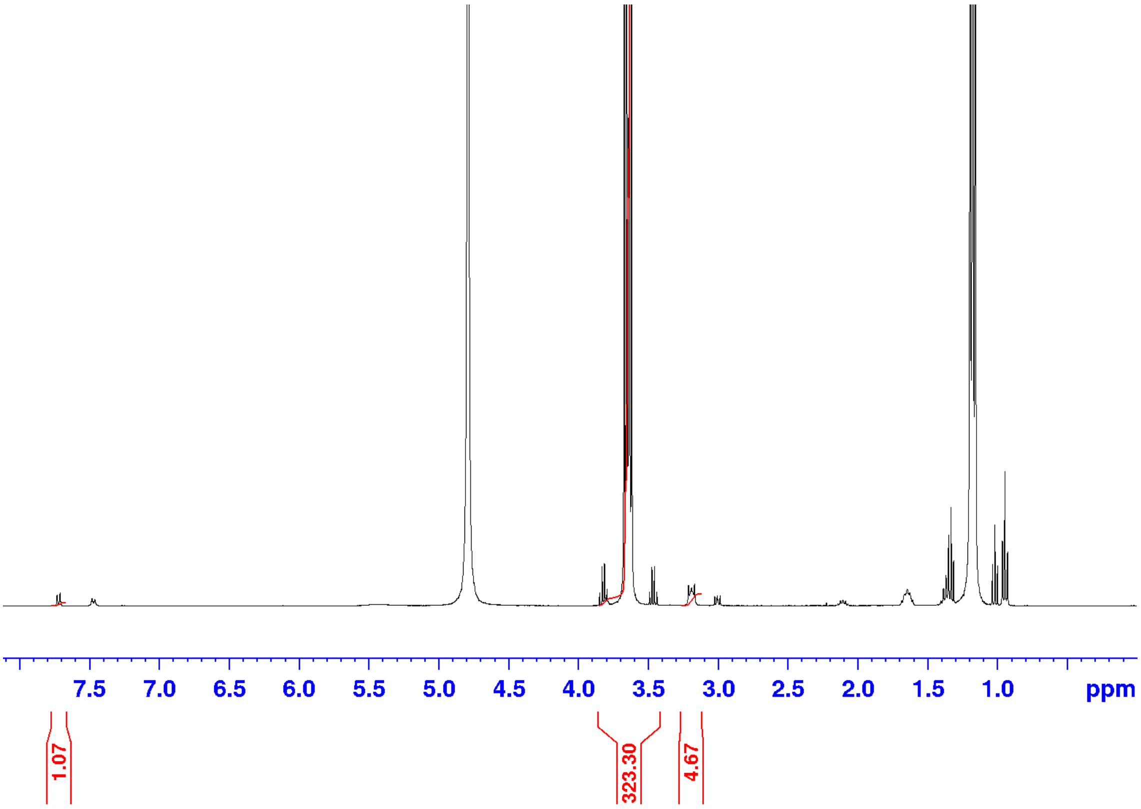

Figure S15 – ^{1}H NMR spectrum (d_1 = 60 s) of **2** (2.78 mM) in D_2O/5.0 % EtOH. Comparative integration indicates 43 % of the anionic and 39 % of the cationic component of **2** has become NMR silent.

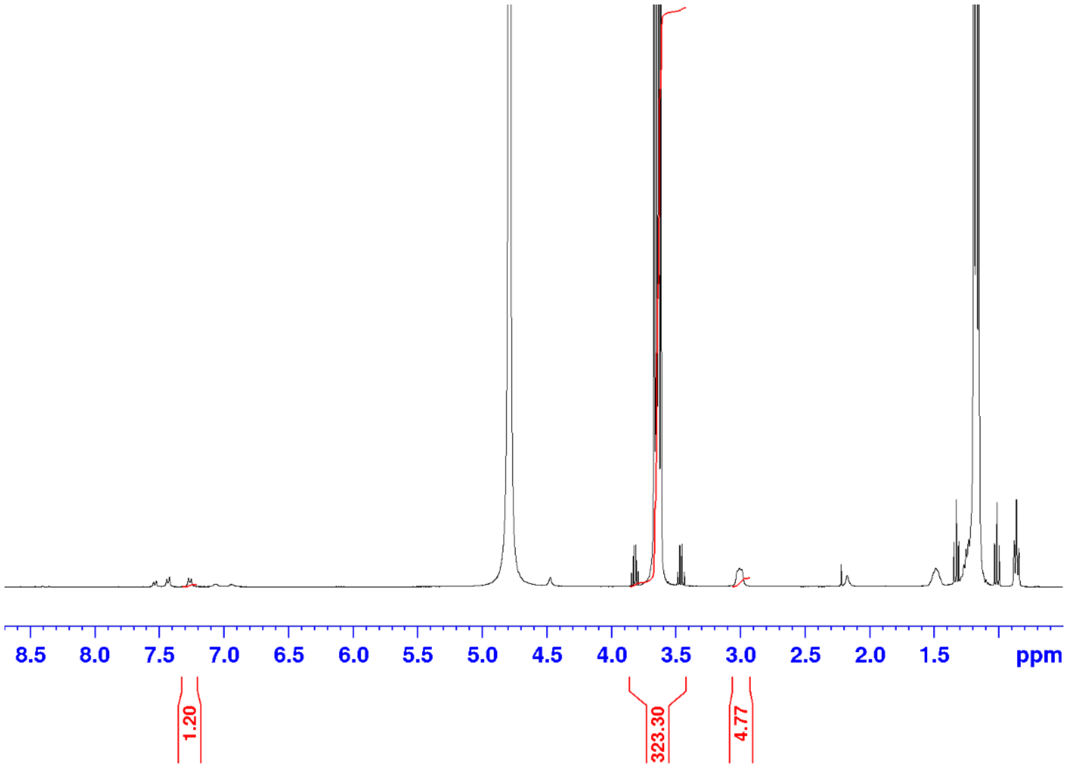

Figure S16 – ^{1}H NMR spectrum (d_1 = 60 s) of **3** (2.78 mM) in D_2O/5.0 % EtOH. Comparative integration indicates 37 % of the anionic and 37 % of the cationic component of **3** has become NMR silent.

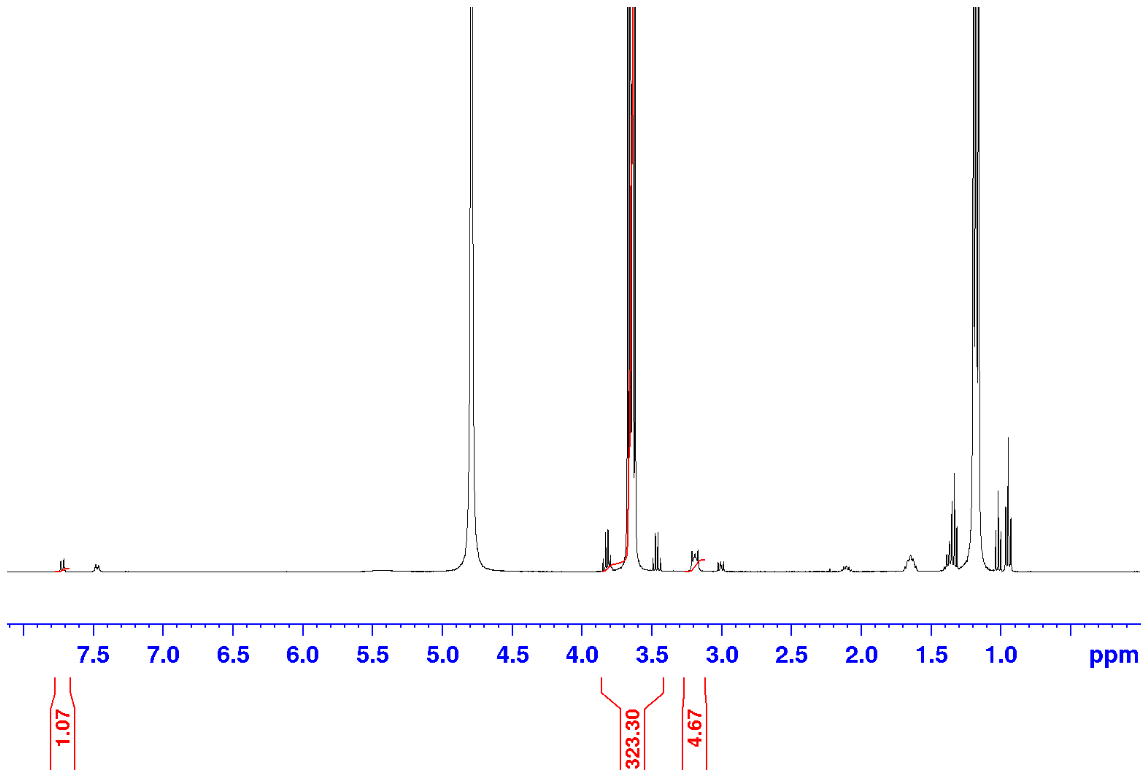

Figure S17 – ^{1}H NMR spectrum (d_1 = 60 s) of **4** (2.78 mM) in D_2O/5.0 % EtOH. Comparative integration indicates 44 % of the anionic and 39 % of the cationic component of **4** has become NMR silent.

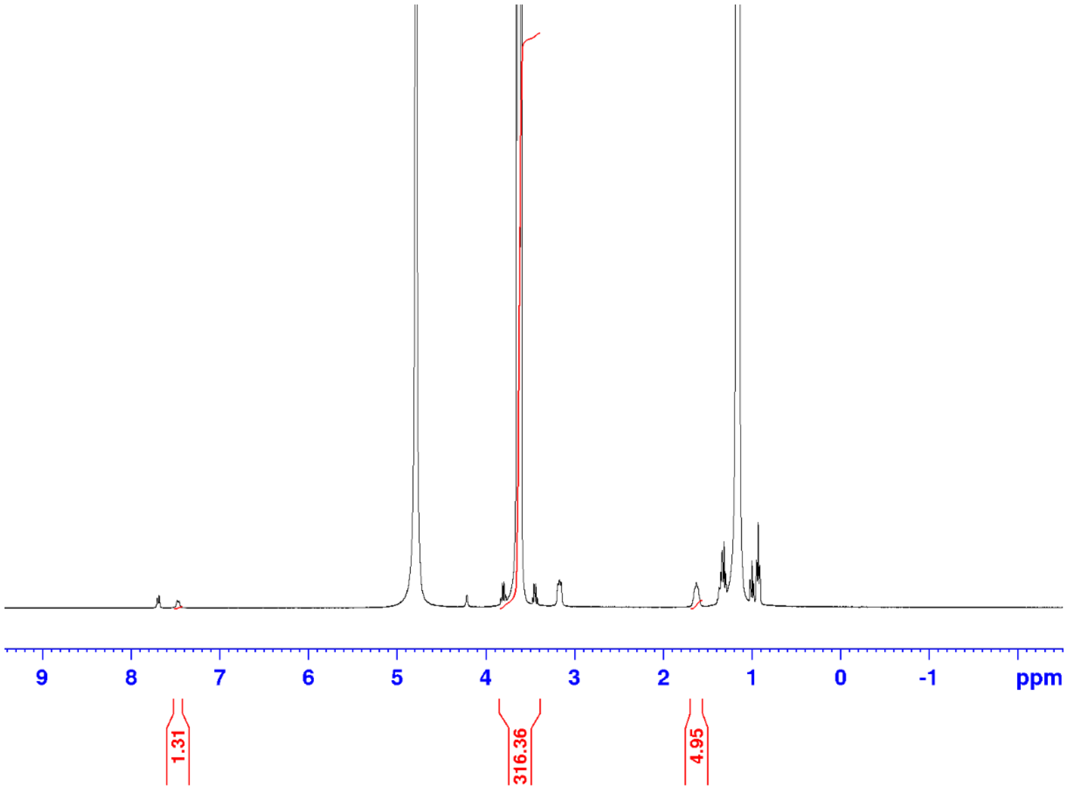

Figure S18 – ^{1}H NMR spectrum (d_1 = 60 s) of **5** (2.78 mM) in D_2O/5.0 % EtOH. Comparative integration indicates 33 % of the anionic and 37 % of the cationic component of **5** has become NMR silent.

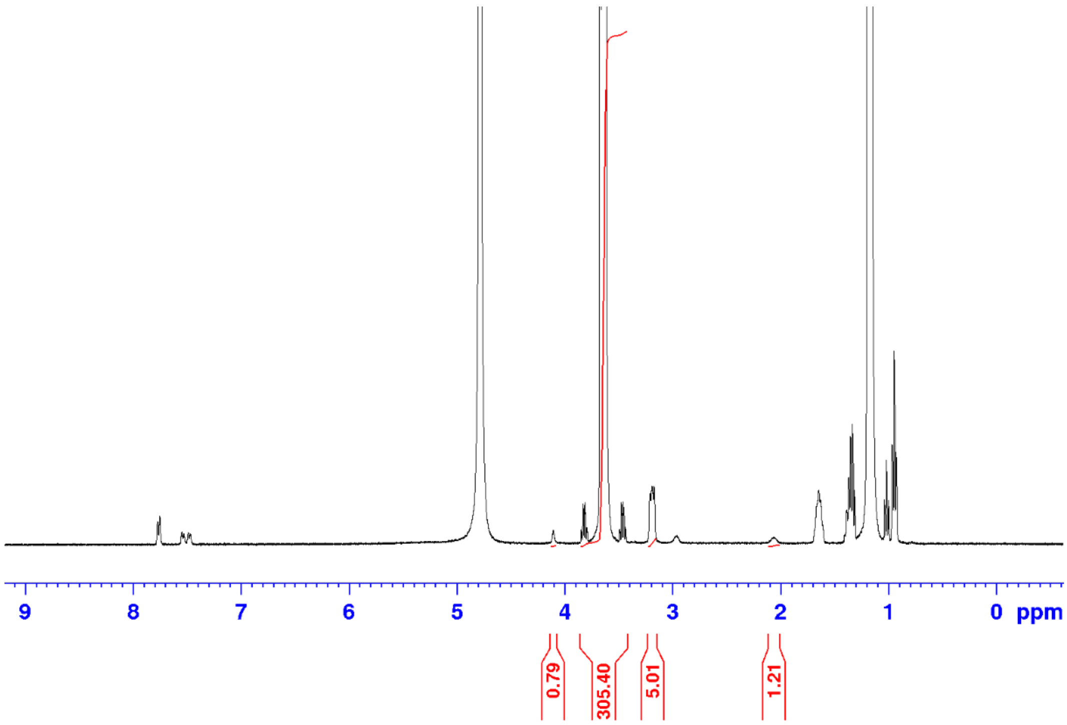

Figure S19 – ^{1}H NMR spectrum (d_1 = 60 s) of co-formulation **a** (2.78 mM) in D_2O/5.0 % EtOH. Comparative integration indicates evidence of higher order aggregate formation.

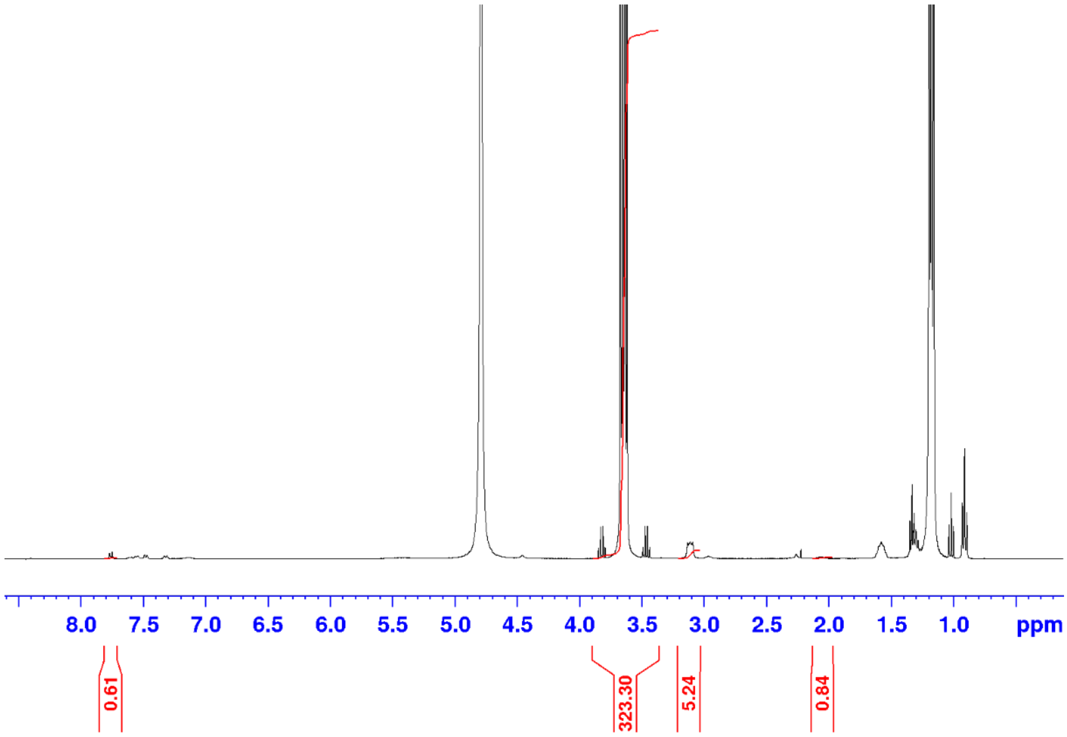

Figure S20 – ^{1}H NMR spectrum (d_1 = 60 s) of co-formulation **b** (2.78 mM) in D_2O/5.0 % EtOH. Comparative integration indicates evidence of higher order aggregate formation.

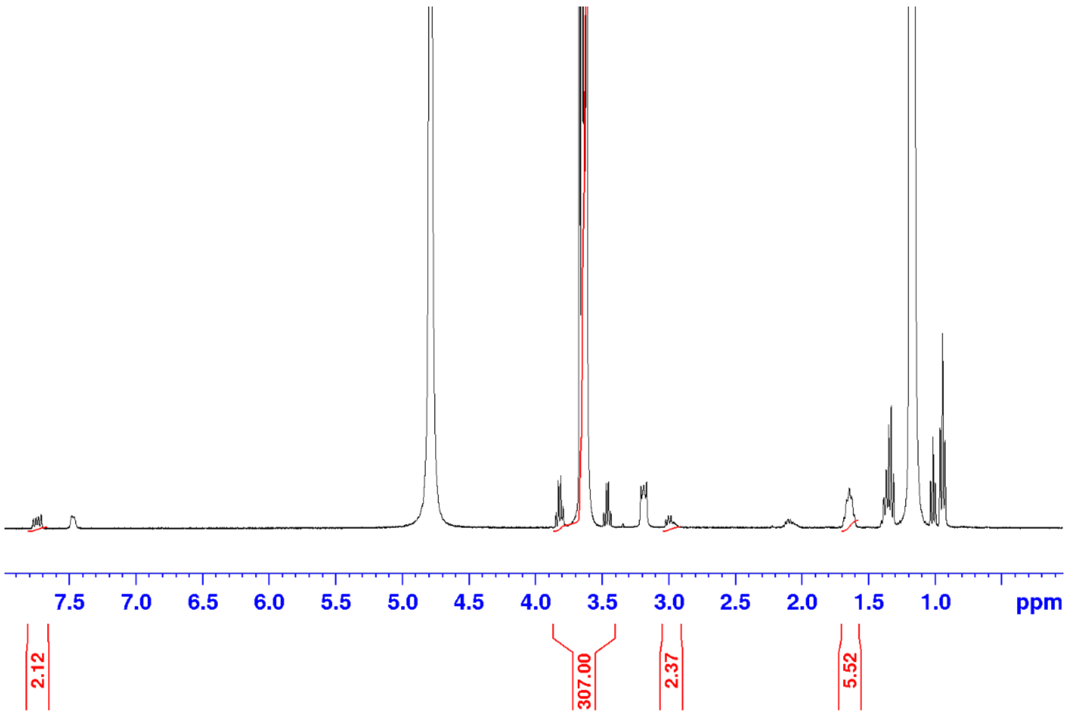

Figure S21 – ^{1}H NMR spectrum (d_1 = 60 s) of co-formulation **c** (2.78 mM) in D_2O/5.0 % EtOH. Comparative integration indicates evidence of higher order aggregate formation.

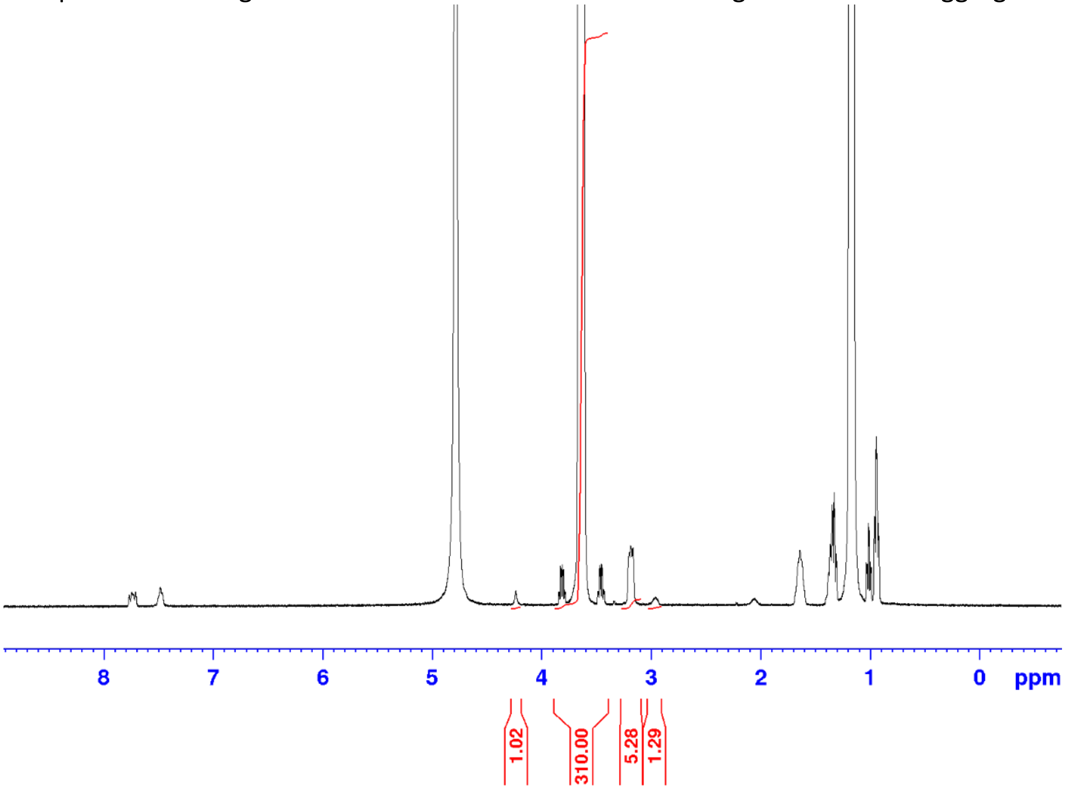

Figure S22 – ^{1}H NMR spectrum (d_1 = 60 s) of co-formulation **d** (2.78 mM) in D_2O/5.0 % EtOH. Comparative integration indicates evidence of higher order aggregate formation.

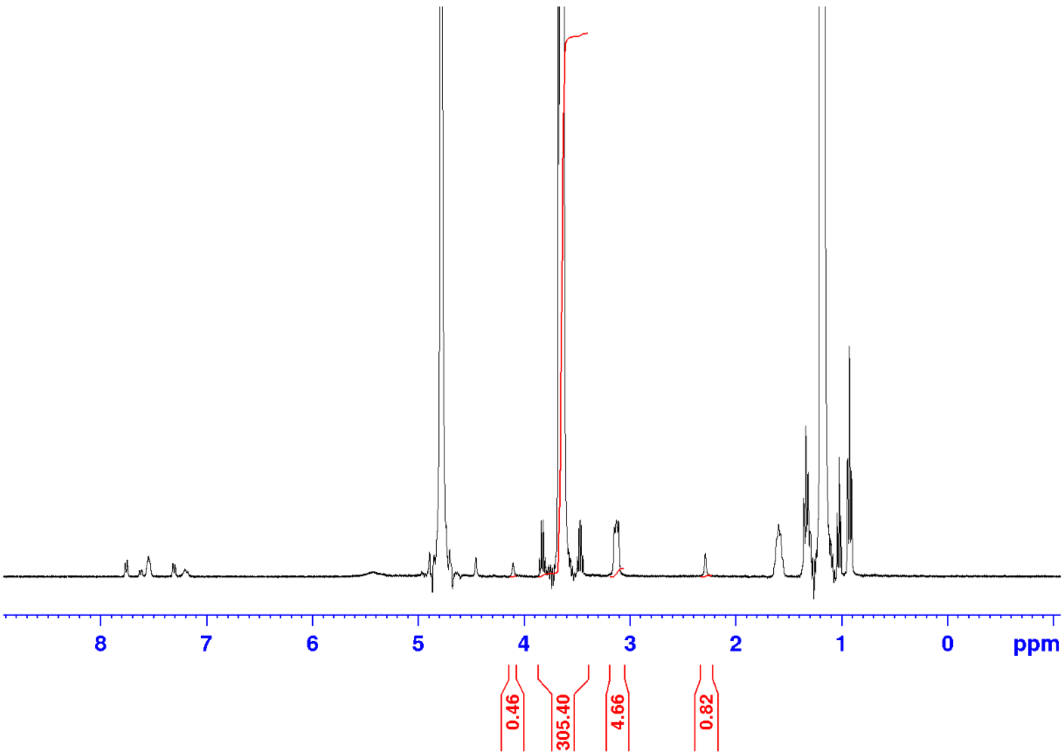

Figure S23 – ^{1}H NMR spectrum (d_1 = 60 s) of co-formulation **e** (2.78 mM) in D_2O/5.0 % EtOH. Comparative integration indicates evidence of higher order aggregate formation.

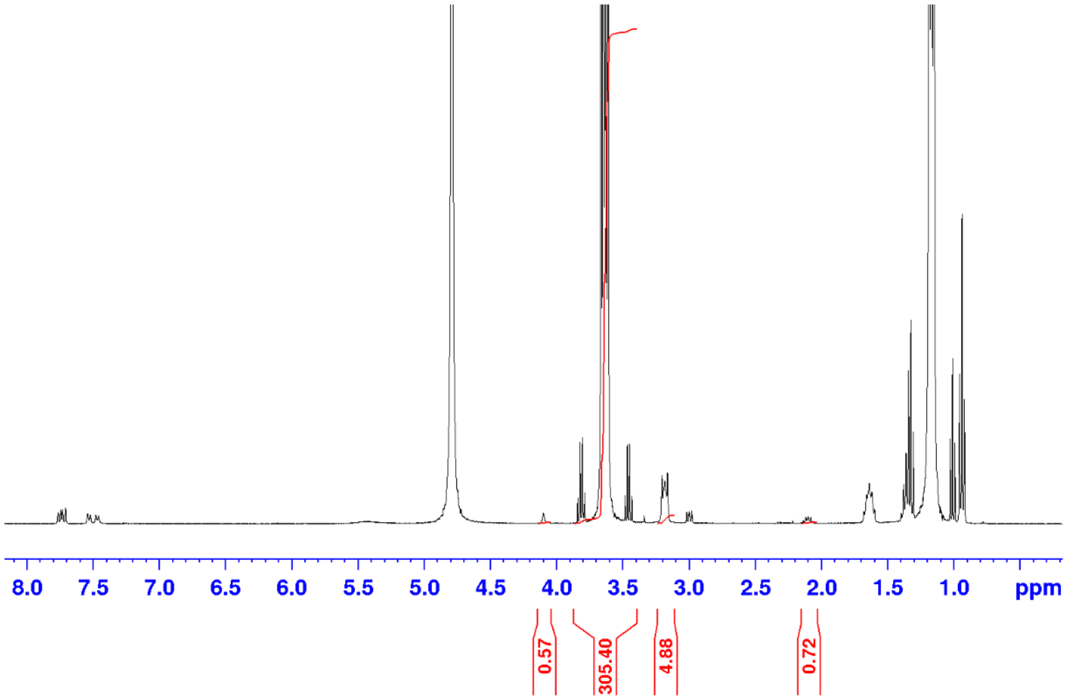

Figure S24 – ^{1}H NMR spectrum (d_1 = 60 s) of co-formulation **f** (2.78 mM) in D_2O/5.0 % EtOH. Comparative integration indicates evidence of higher order aggregate formation.

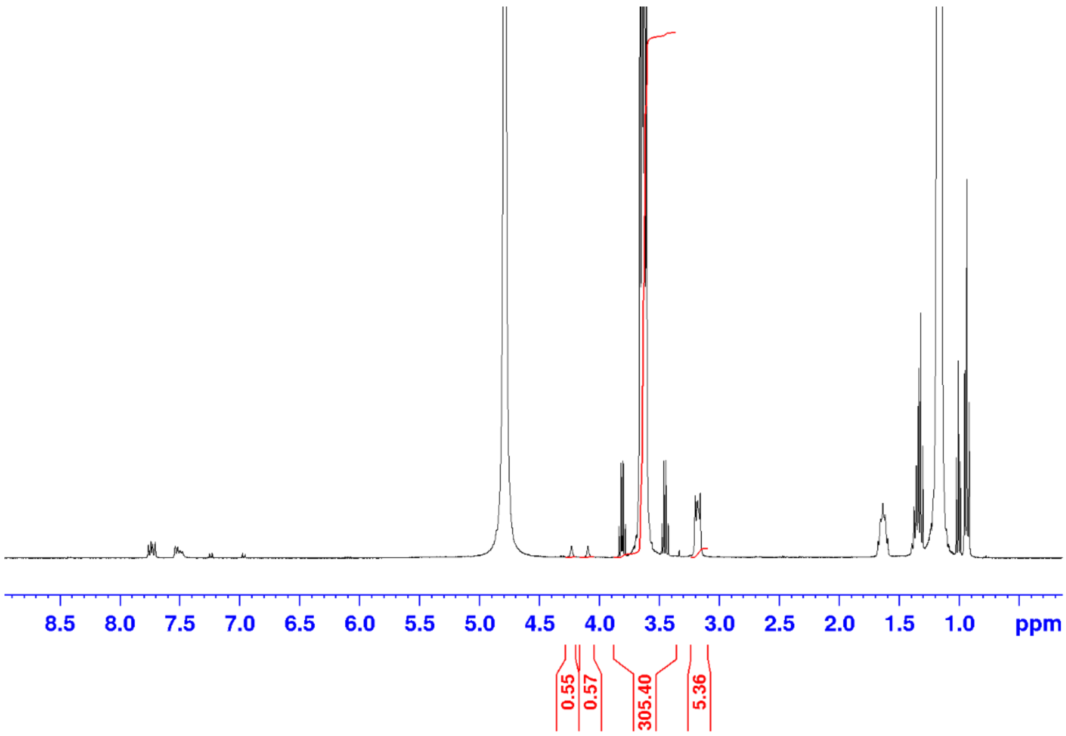

Figure S25 – ^{1}H NMR spectrum (d_1 = 60 s) of co-formulation **g** (2.78 mM) in D_2O/5.0 % EtOH. Comparative integration indicates evidence of higher order aggregate formation.

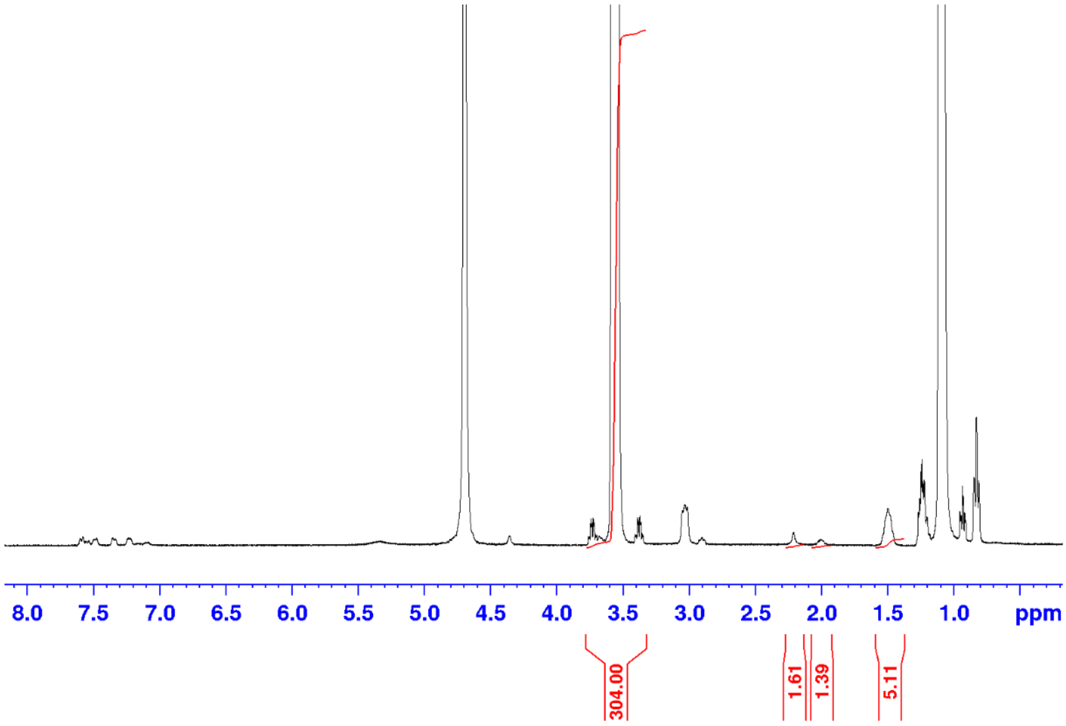

Figure S26 – ^{1}H NMR spectrum (d_1 = 60 s) of co-formulation **h** (2.78 mM) in D_2O/5.0 % EtOH. Comparative integration indicates evidence of higher order aggregate formation.

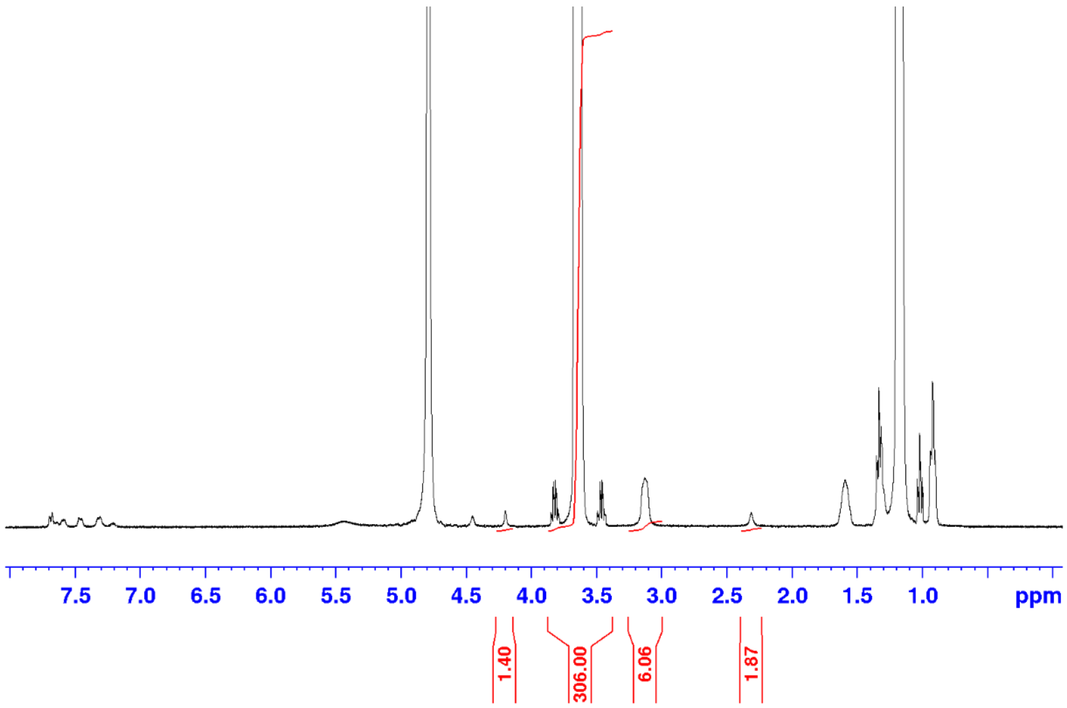

Figure S27 – ^{1}H NMR spectrum (d_1 = 60 s) of co-formulation **i** (2.78 mM) in D_2O/5.0 % EtOH. Comparative integration indicates evidence of higher order aggregate formation.

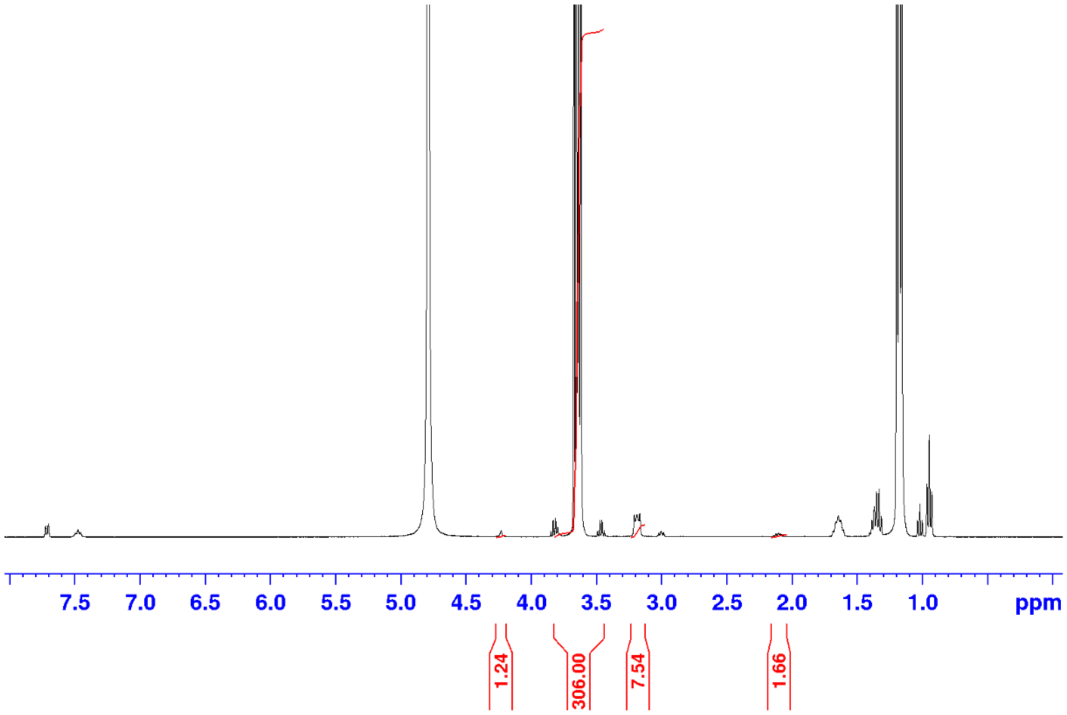

Figure S28 – ^{1}H NMR spectrum (d_1 = 60 s) of co-formulation **j** (2.78 mM) in D_2O/5.0 % EtOH. Comparative integration indicates evidence of higher order aggregate formation.

^1H NMR spectroscopy dilution study data

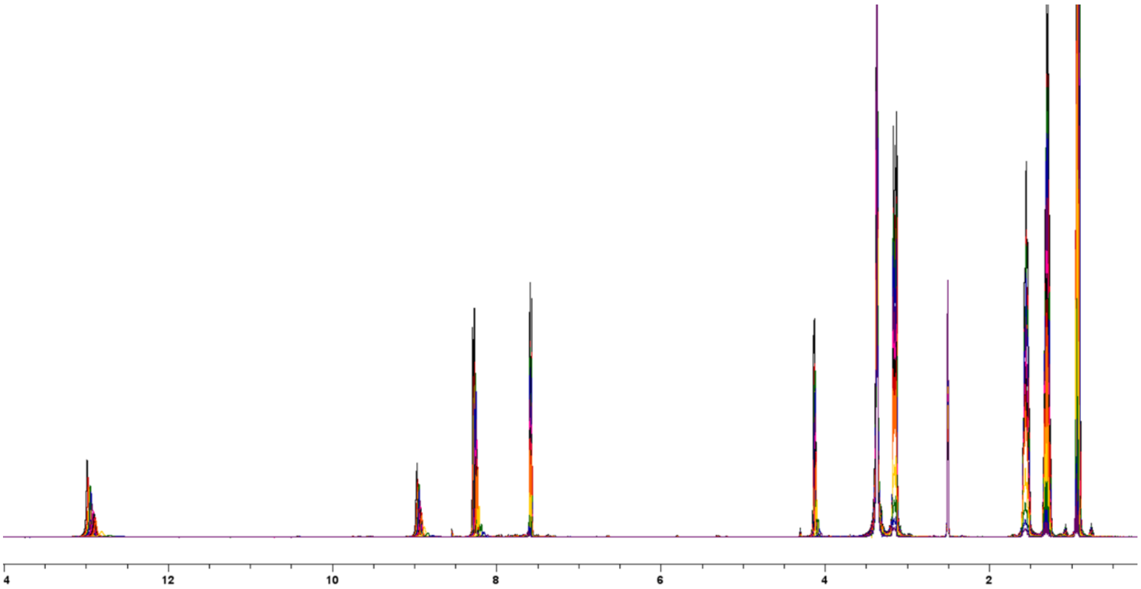

Figure S29 – ^1H NMR stack plot of **4** in a DMSO-d_6 0.5 % H_2O solution. Samples were prepared in series with an aliquot of the most concentrated solution undergoing serial dilution.

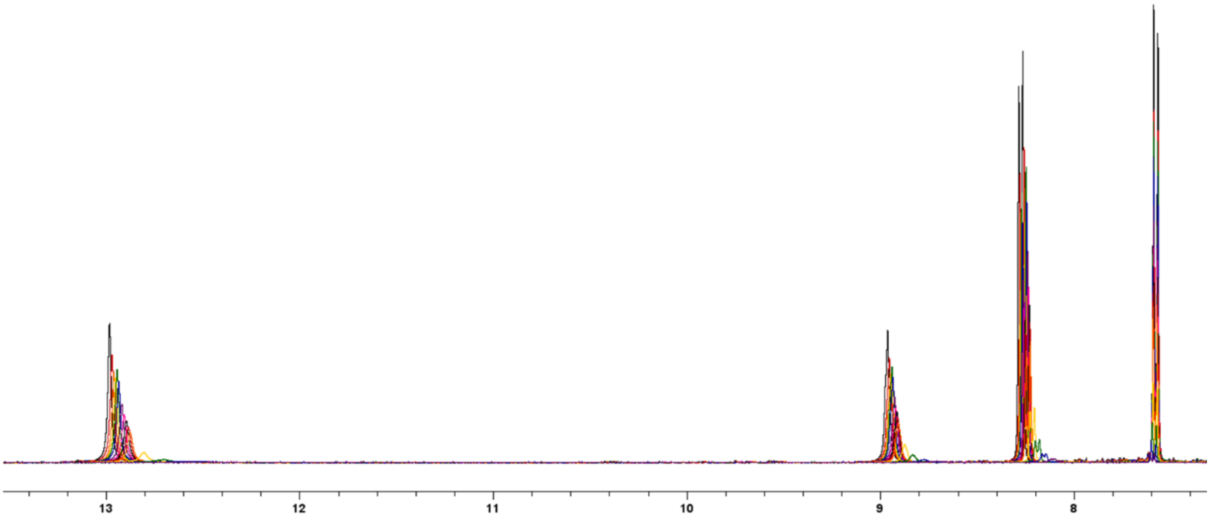

Figure S30 – Enlarged ^1H NMR stack plot of **4** in a DMSO-d_6 0.5 % H_2O solution. Samples were prepared in series with an aliquot of the most concentrated solution undergoing serial dilution.

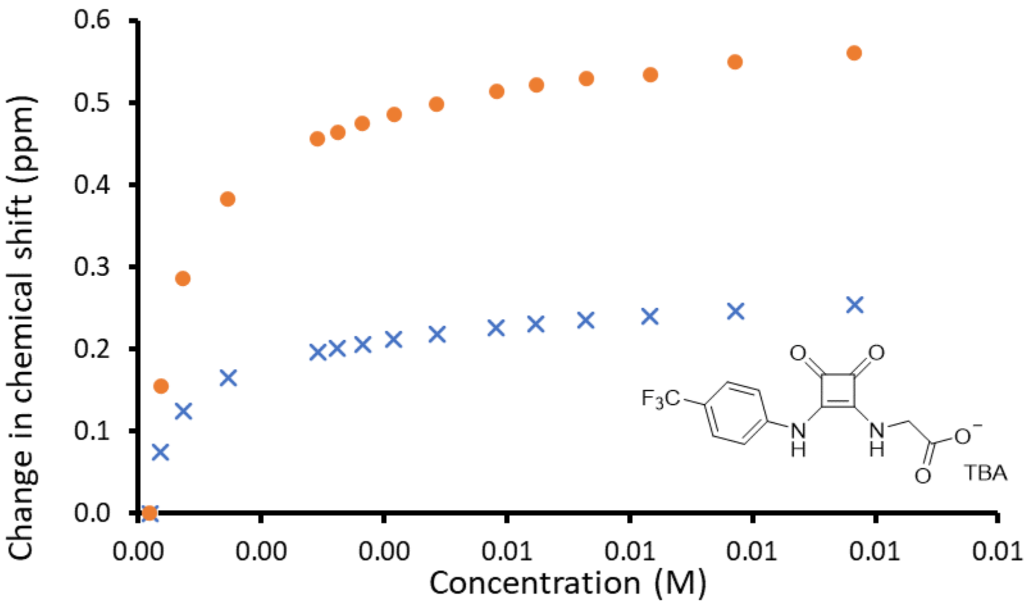

Figure S31 – Graph illustrating the ^{1}H NMR spectroscopy down-field change in chemical shift of urea NH resonances with increasing concentrations of **4** in DMSO-d_6 0.5 % H_2O solution (298 K).

Self-association constant calculation

4 – Dilution study in DMSO-d_6 0.5 % H_2O. Values calculated from data gathered from both NH 1 and 2.

Equal K/Dimerisation mode

K_e = 15265 M^{-1} ± 1.63 % K_{dim} = 7632 M^{-1} ± 0.82 %

http://app.supramolecular.org/bindfit/view/ee86deb0-09a1-4edf-a599-741ce48499e7

CoEK model

K_e = 55964 M^{-1} ± 7.23 % K_{dim} = 27982 M^{-1} ± 3.61 % ρ = 1.73 ± 9.91 %

http://app.supramolecular.org/bindfit/view/588200aa-c98d-4425-8500-7ac5dc1bdb0e

[1]H DOSY data

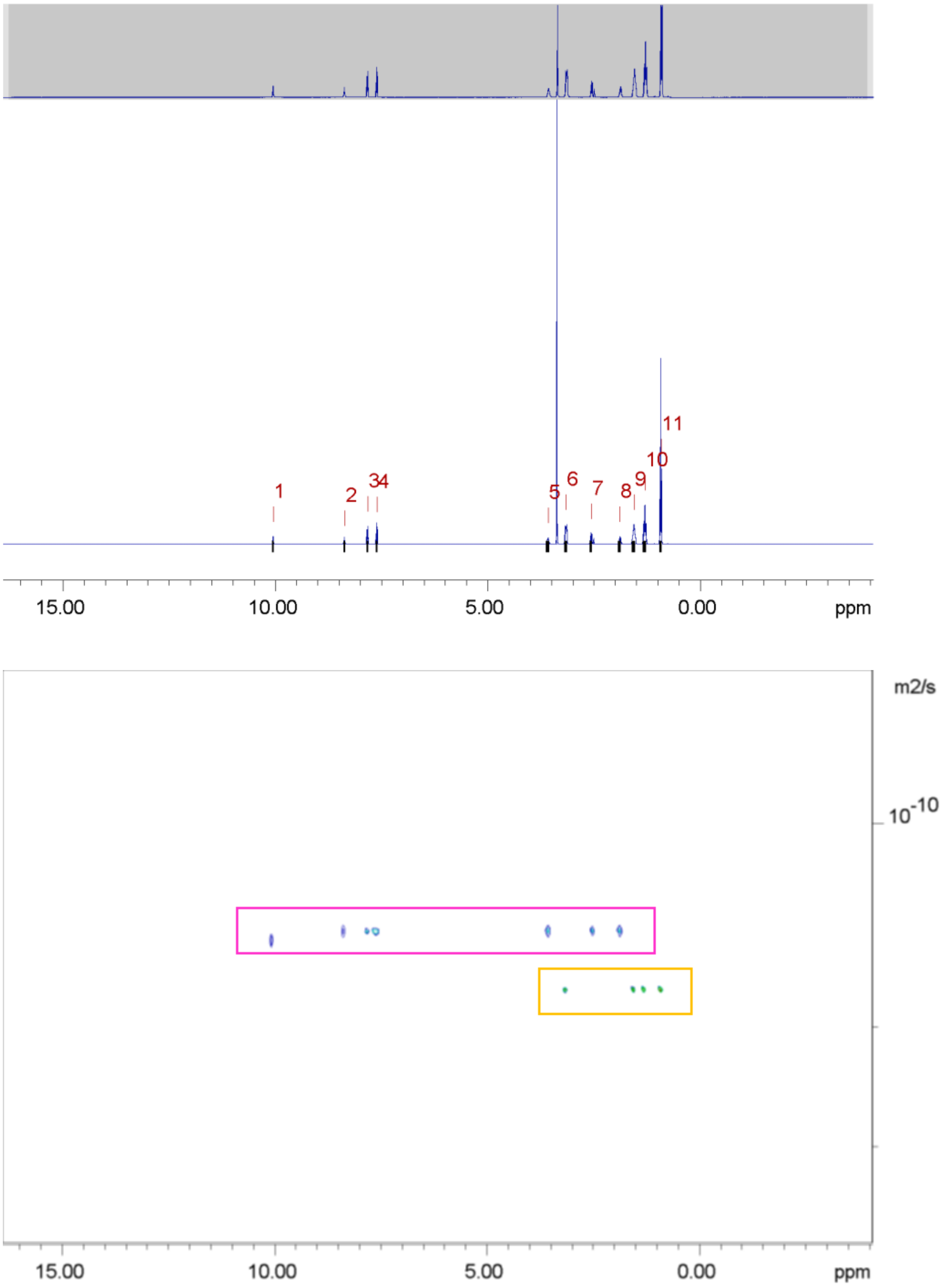

Figure S32 – ^{1}H DOSY NMR of **1** (112.12 mM) in DMSO-d_6 conducted at 298 K. Anionic component highlighted in purple, TBA counter cation highlighted in yellow.

Peak name	F2 [ppm]	Io	error	D [m2/s]	error	fitInfo
1	10.054	2.84e+08	1.431e+04	1.49e-10	1.672e-14	Done
2	8.378	2.82e+08	1.315e+04	1.45e-10	1.509e-14	Done
3	7.832	7.34e+08	1.314e+04	1.45e-10	5.781e-15	Done
4	7.616	7.37e+08	1.314e+04	1.45e-10	5.769e-15	Done
5	3.586	5.76e+08	1.831e+04	1.45e-10	1.028e-14	Done
6	3.165	1.81e+09	1.805e+04	1.75e-10	3.831e-15	Done
7	2.571	6.05e+08	1.380e+04	1.45e-10	7.377e-15	Done
8	1.899	5.69e+08	1.712e+04	1.45e-10	9.708e-15	Done
9	1.570	2.01e+09	1.989e+04	1.75e-10	3.794e-15	Done
10	1.310	2.61e+09	1.989e+04	1.75e-10	2.919e-15	Done
11	0.929	4.37e+09	1.529e+04	1.76e-10	1.343e-15	Done

Figure S33 – ^{1}H DOSY NMR spectrum of **1** (112.12 mM) in DMSO-d_6 at 298 K and a table reporting the diffusion constants calculated for each peak used to determine the hydrodynamic diameter of the anionic component of **1** (d_H = 1.50 nm). Peaks 1 – 5, 7 and 8 correspond to the anionic component of **1** while peaks 6, and 9 – 11 correspond to the cationic component of **1**.

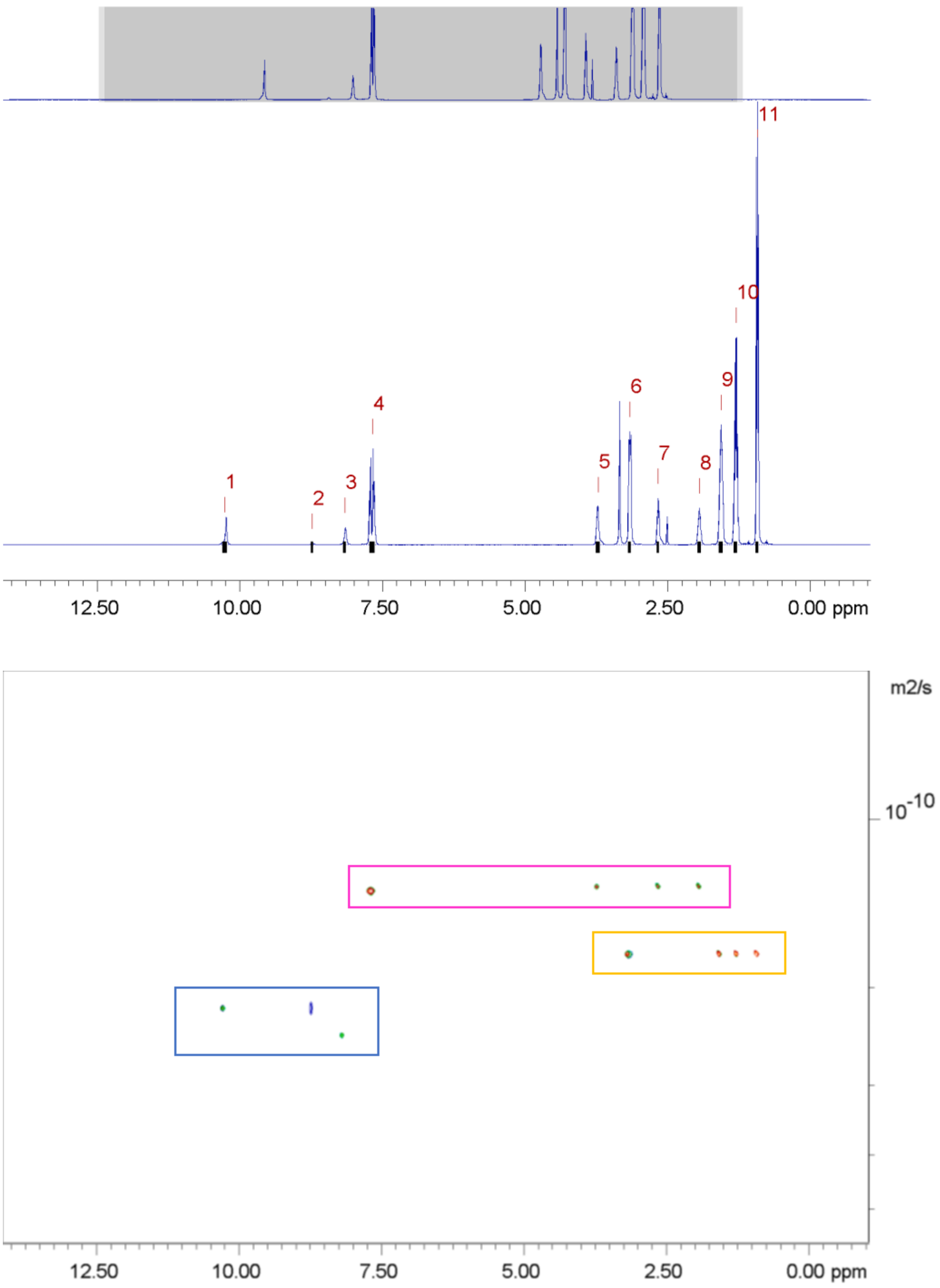

Figure S34 – ^{1}H DOSY NMR of **4** (112.12 mM) in DMSO-d_6 conducted at 298 K. Anionic component highlighted in purple, aromatic NH's highlighted in blue, TBA counter cation highlighted in yellow.

Peak name	F2 [ppm]	Io	error	D [m2/s]	error	fitInfo
1	10.262	9.59e+08	8.080e+04	2.18e-10	3.988e-14	Done
2	8.731	6.47e+07	6.201e+04	2.18e-10	4.538e-13	Done
3	8.158	6.91e+08	6.713e+04	2.43e-10	5.095e-14	Done
4	7.680	5.08e+09	7.649e+04	1.33e-10	4.536e-15	Done
5	3.718	1.98e+09	6.532e+04	1.33e-10	9.929e-15	Done
6	3.167	5.08e+09	5.478e+04	1.74e-10	4.129e-15	Done
7	2.670	1.75e+09	5.283e+04	1.32e-10	9.058e-15	Done
8	1.946	1.88e+09	6.084e+04	1.33e-10	9.744e-15	Done
9	1.567	6.92e+09	6.844e+04	1.74e-10	3.776e-15	Done
10	1.306	9.00e+09	6.693e+04	1.74e-10	2.834e-15	Done
11	0.931	1.53e+10	5.838e+04	1.73e-10	1.452e-15	Done

Figure S35 – ^{1}H DOSY NMR spectrum of **4** (112.12 mM) in DMSO-d_6 at 298 K and a table reporting the diffusion constants calculated for each peak used to determine the hydrodynamic diameter of the anionic component of **4** (d_H = 1.65 nm). Peaks 1 – 3 correspond to the NH's of the anionic component of **4**, peaks 4,5, 7 and 8 correspond to the anionic component of **4** while peaks 6, and 9 – 11 correspond to the cationic component of **4**.

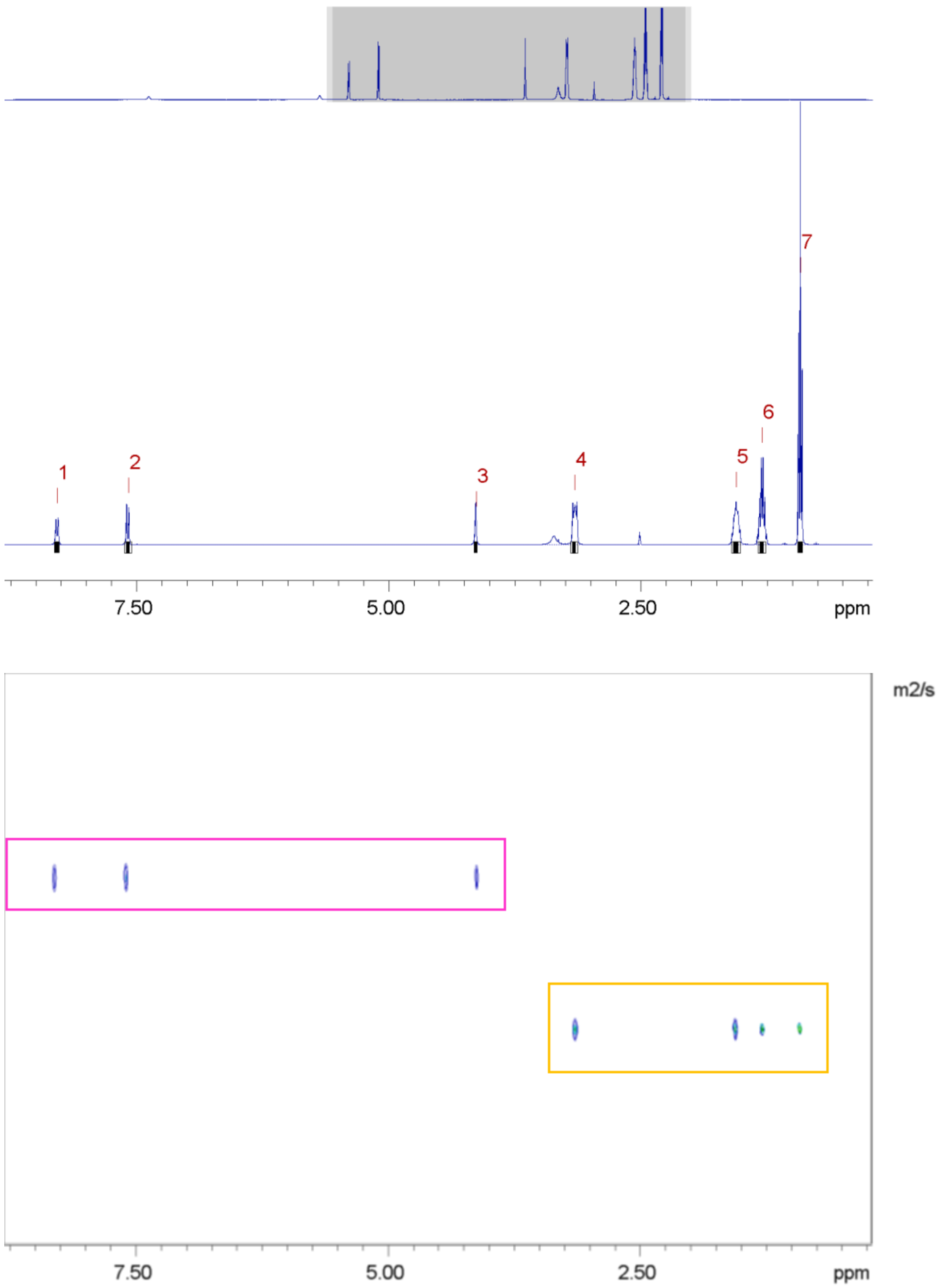

Figure S36 – ^{1}H DOSY NMR of **5** (112.12 mM) in DMSO-d_6 conducted at 298 K. Anionic component highlighted in purple, TBA counter cation highlighted in yellow.

Peak name	F2 [ppm]	Io	error	D [m2/s]	error	fitInfo
1	8.283	2.44e+09	5.557e+04	1.36e-10	6.847e-15	Done
2	7.580	2.78e+09	6.491e+04	1.36e-10	7.021e-15	Done
3	4.132	2.19e+09	5.020e+04	1.36e-10	6.880e-15	Done
4	3.156	7.05e+09	7.242e+04	1.81e-10	3.973e-15	Done
5	1.555	7.81e+09	7.880e+04	1.80e-10	3.894e-15	Done
6	1.297	9.77e+09	7.447e+04	1.80e-10	2.944e-15	Done
7	0.915	1.66e+10	6.004e+04	1.80e-10	1.395e-15	Done

Figure S37 – ^{1}H DOSY NMR spectrum of **5** (112.12 mM) in DMSO-d_6 at 298 K and a table reporting the diffusion constants calculated for each peak used to determine the hydrodynamic diameter of the anionic component of **5** (d_H = 1.61 nm). Peaks 1, 2 and 3 correspond to the anionic component of **5** while peaks 4 – 7 correspond to the cationic component of **5**.

Section S9: DLS data

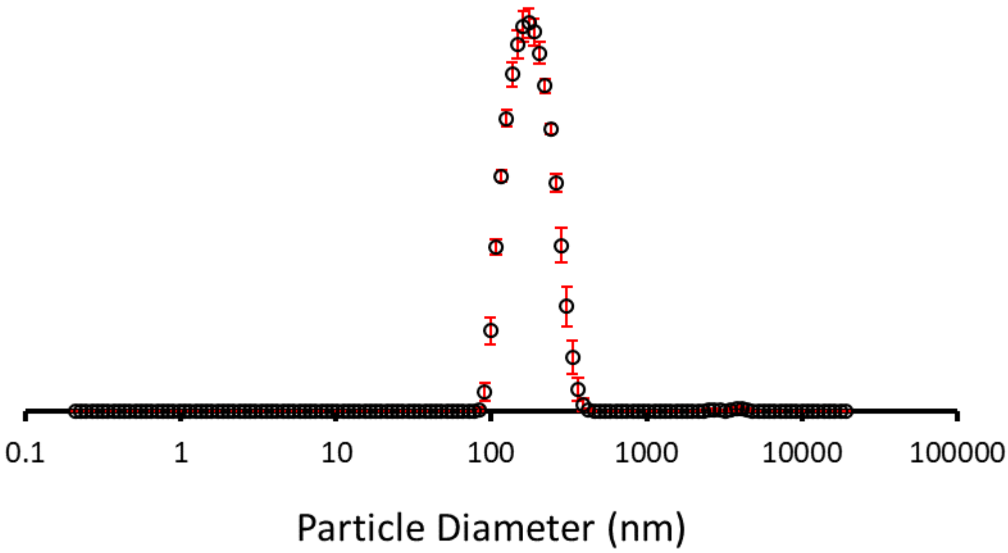

Figure S38 – The average intensity particle size distribution calculated (Peak maxima = 184 nm) using 10 DLS runs for **1** (2.78 mM) in an EtOH:H_2O (1:19) solution at 298 K.

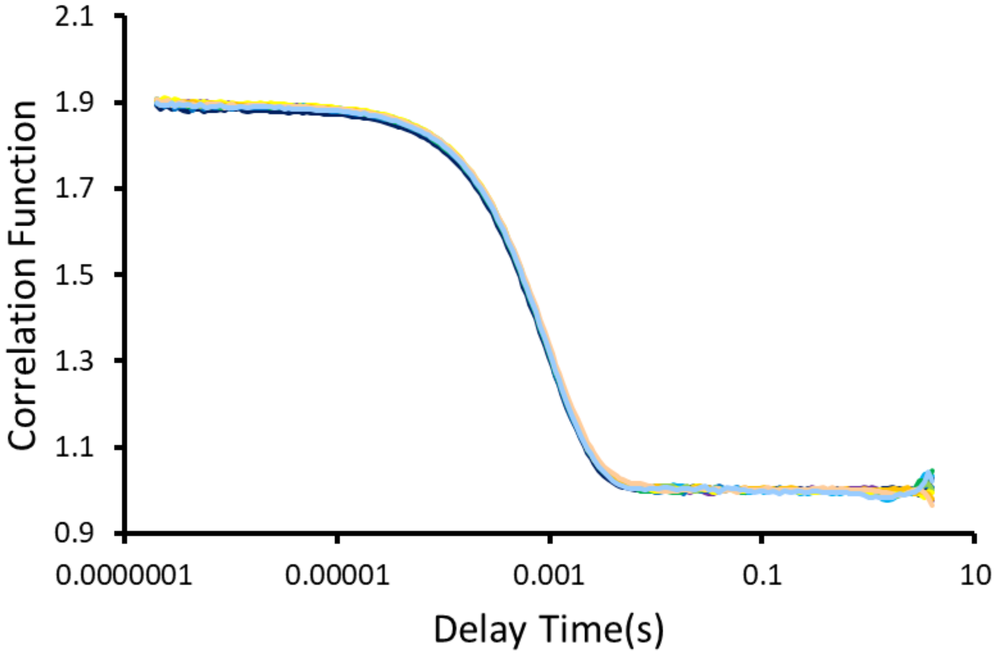

Figure S39 – Correlation function data for 10 DLS runs of **1** (2.78 mM) in an EtOH:H_2O (1:19) solution at 298 K.

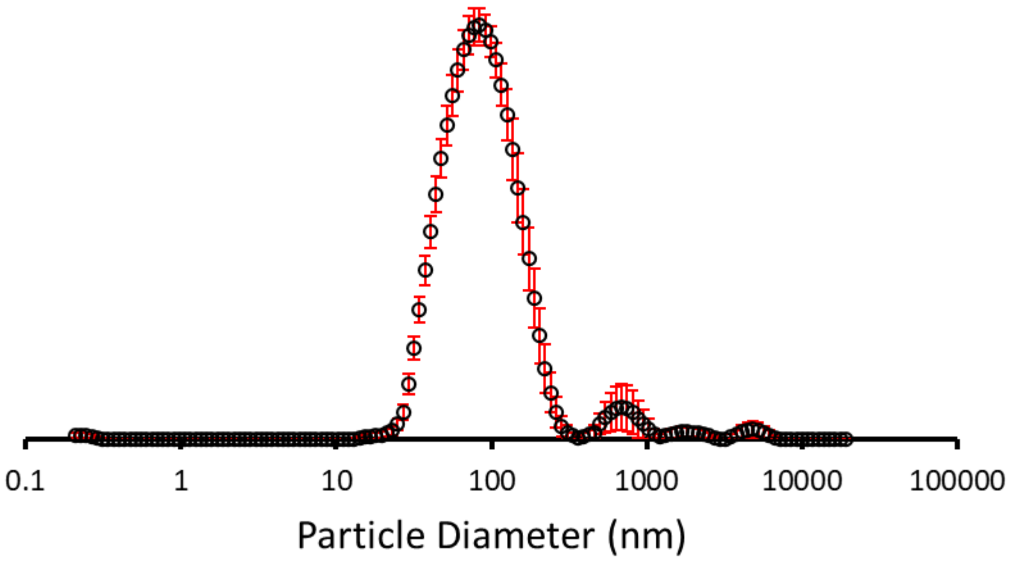

Figure S40 – The average intensity particle size distribution calculated (Peak maxima = 100 nm) using 10 DLS runs for **1** (0.28 mM) in an EtOH:H_2O (1:19) solution at 298 K.

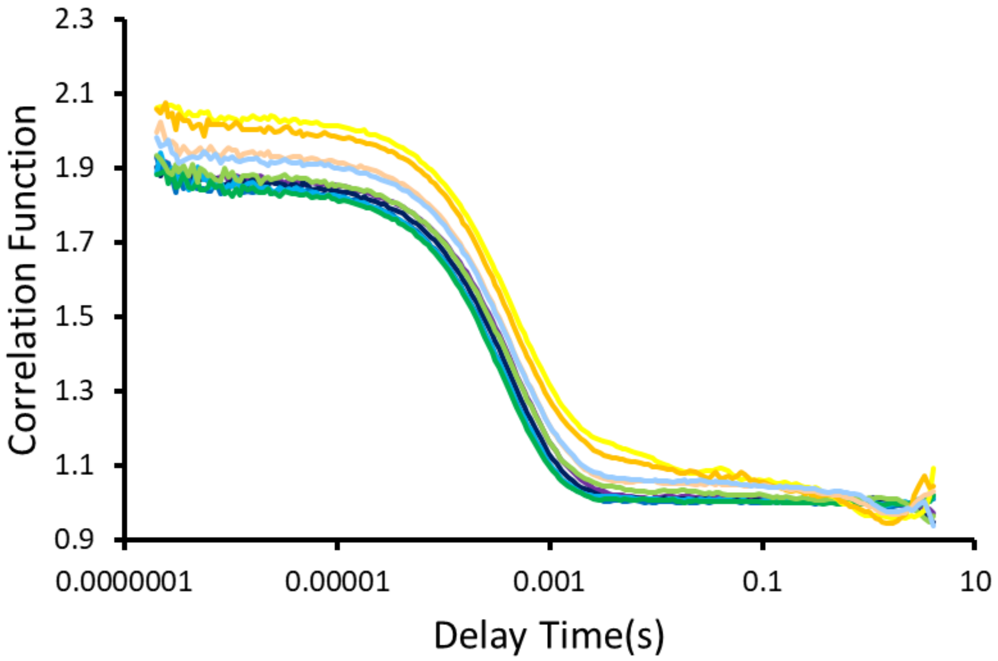

Figure S41– Correlation function data for 10 DLS runs of **1** (0.28 mM) in an EtOH:H_2O (1:19) solution at 298 K.

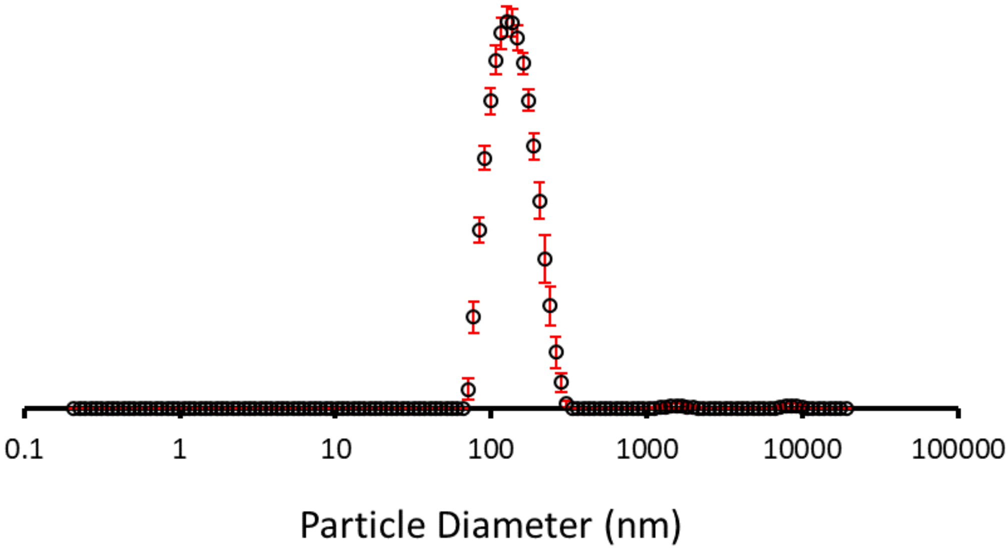

Figure S42 – The average intensity particle size distribution calculated (Peak maxima = 143 nm) using 10 DLS runs for **2** (2.78 mM) in an EtOH:H_2O (1:19) solution at 298 K.

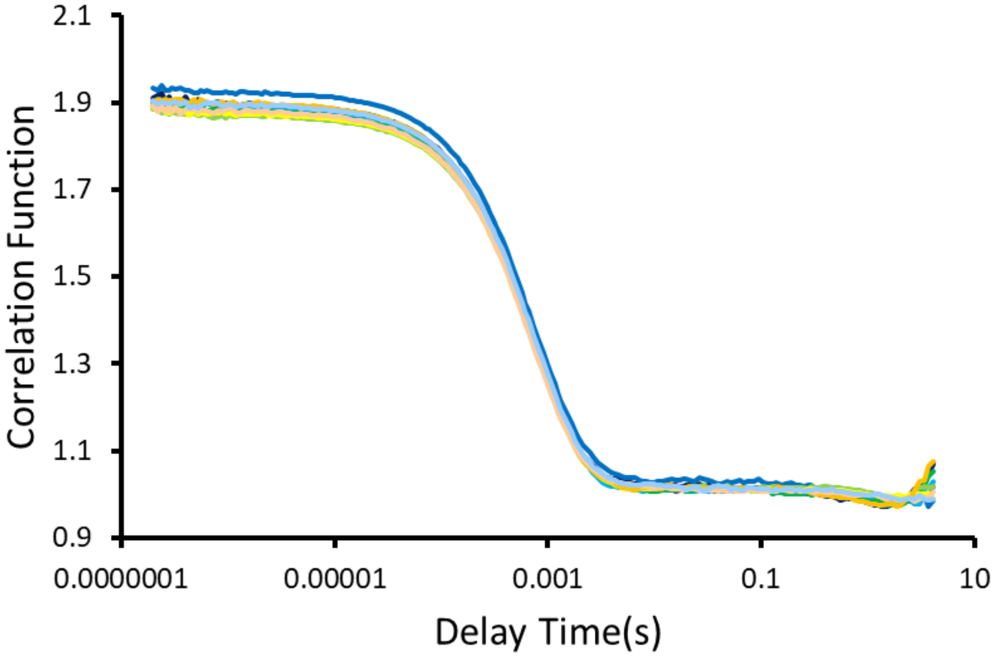

Figure S43 – Correlation function data for 10 DLS runs of **2** (2.78 mM) in an EtOH:H_2O (1:19) solution at 298 K.

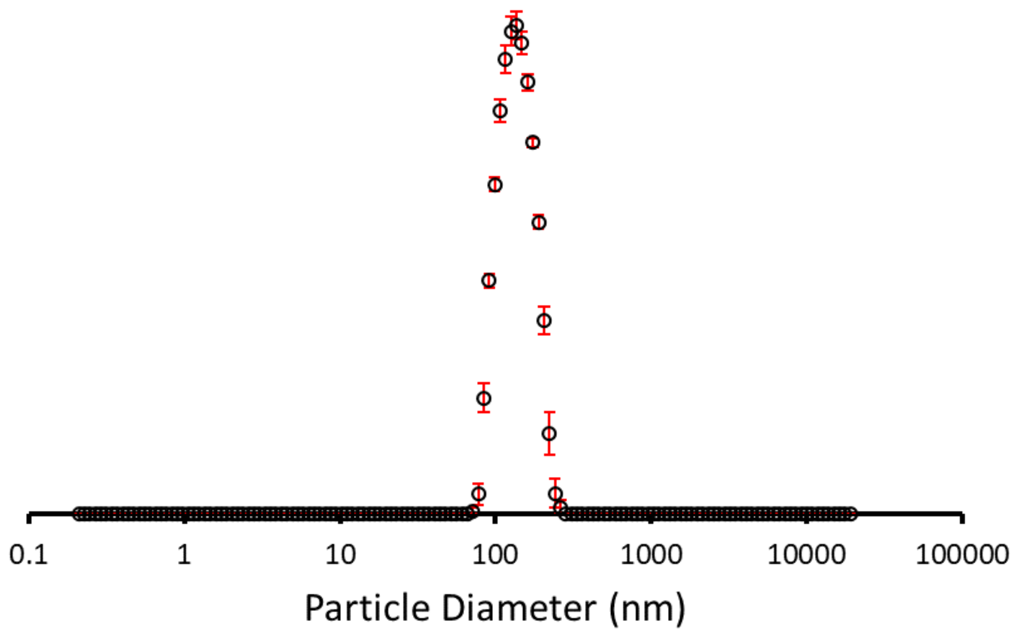

Figure S44 – The average intensity particle size distribution calculated (Peak maxima = 139 nm) using 10 DLS runs for **2** (0.28 mM) in an EtOH:H_2O (1:19) solution at 298 K.

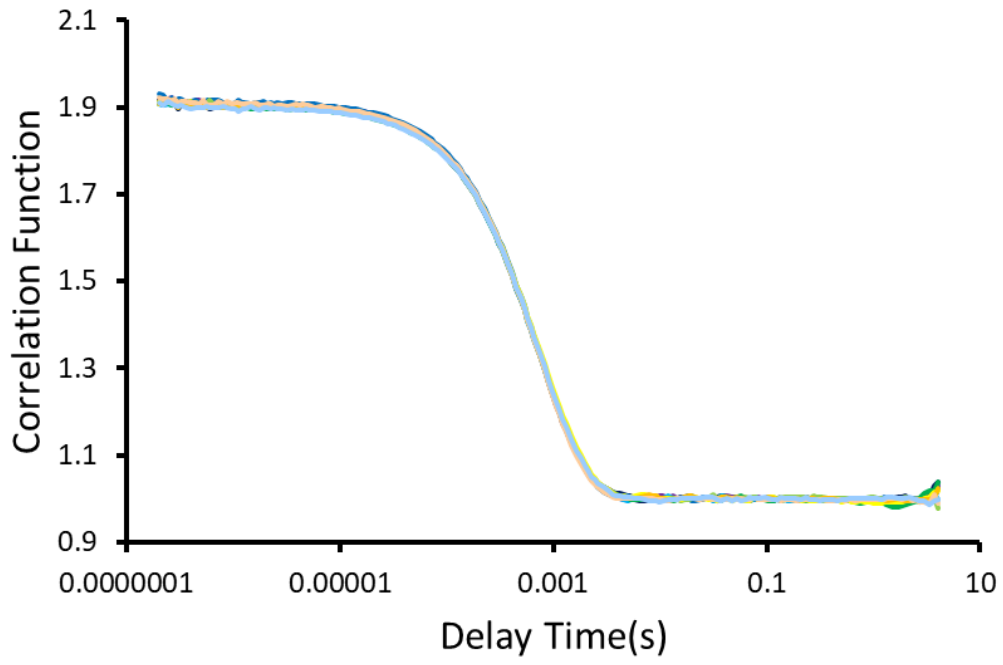

Figure S45 – Correlation function data for 10 DLS runs of **2** (0.28 mM) in an EtOH:H_2O (1:19) solution at 298 K.

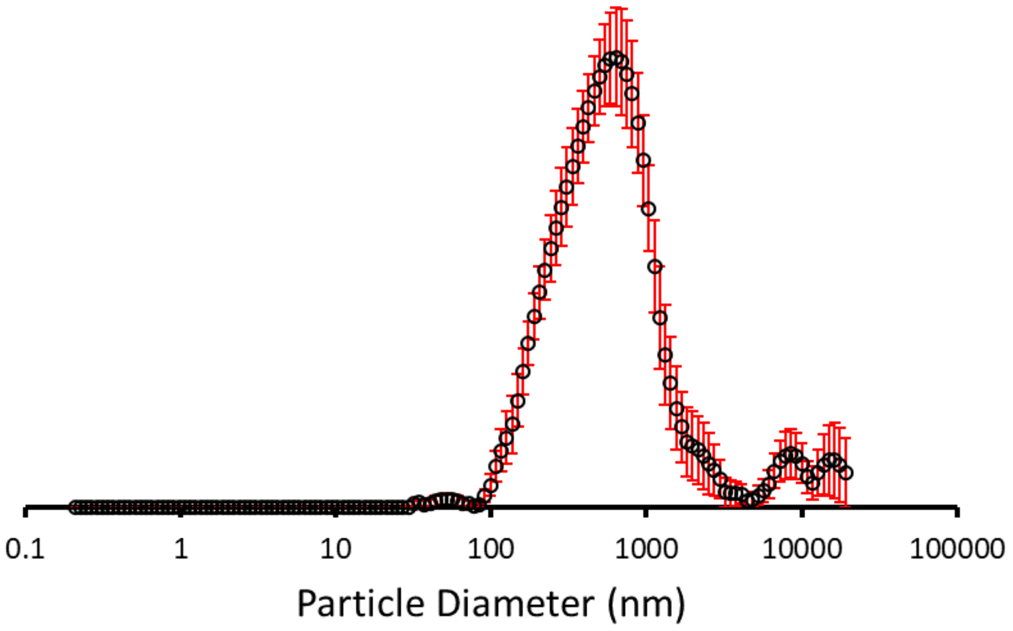

Figure S46 – The average intensity particle size distribution calculated (Peak maxima = 672 nm) using 10 DLS runs for **3** (2.78 mM) in an EtOH:H_2O (1:19) solution at 298 K.

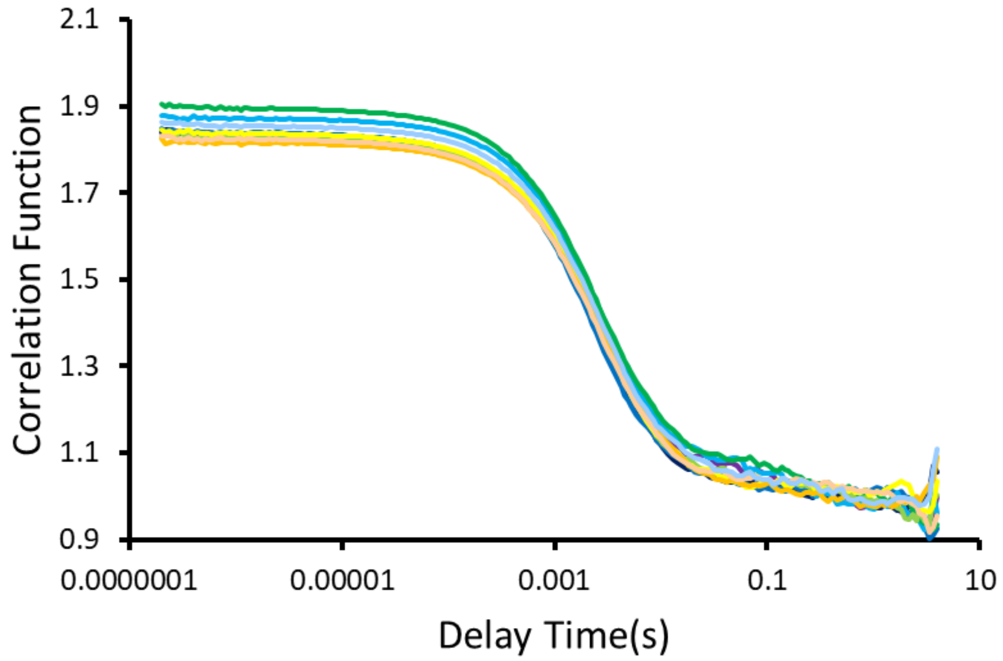

Figure S47 – Correlation function data for 10 DLS runs of **3** (2.78 mM) in an EtOH:H_2O (1:19) solution at 298 K.

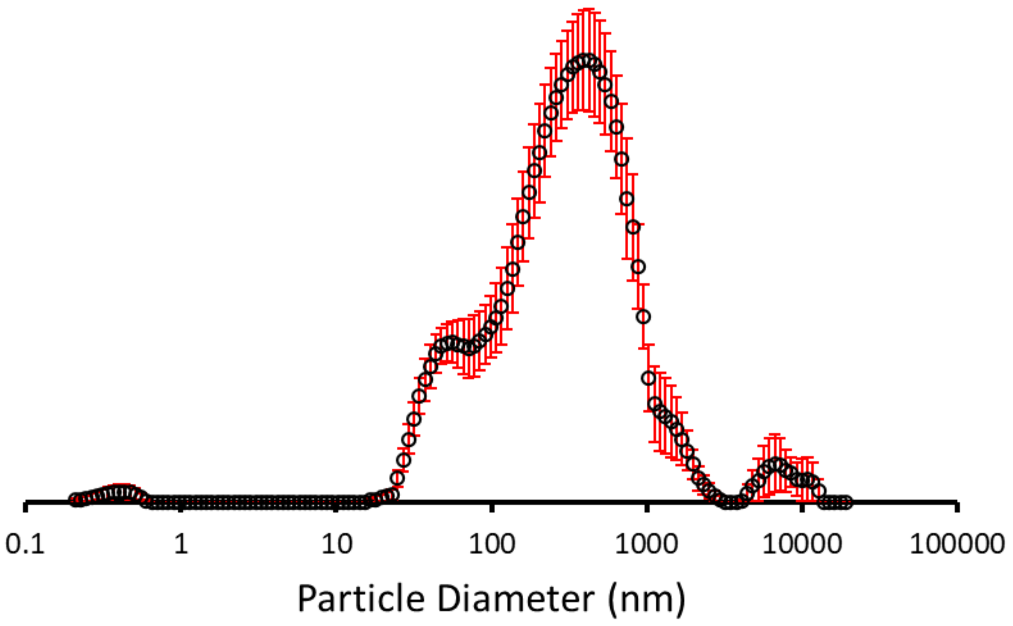

Figure S48 – The average intensity particle size distribution calculated (Peak maxima = 462 nm) using 9 DLS runs for **3** (0.28 mM) in an EtOH:H_2O (1:19) solution at 298 K.

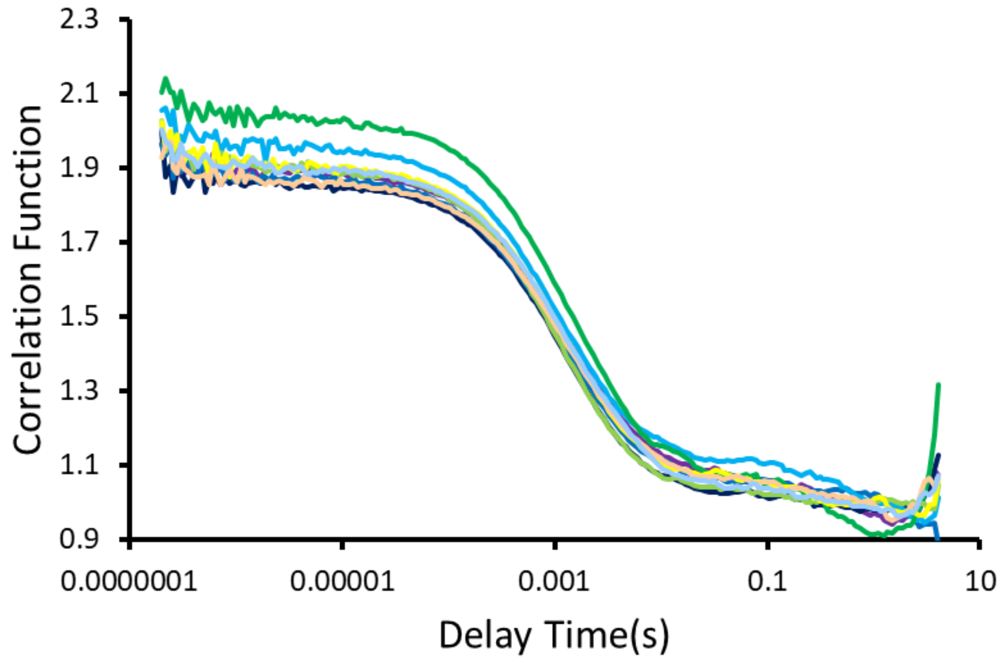

Figure S49 – Correlation function data for 9 DLS runs of **3** (0.28 mM) in an EtOH:H_2O (1:19) solution at 298 K.

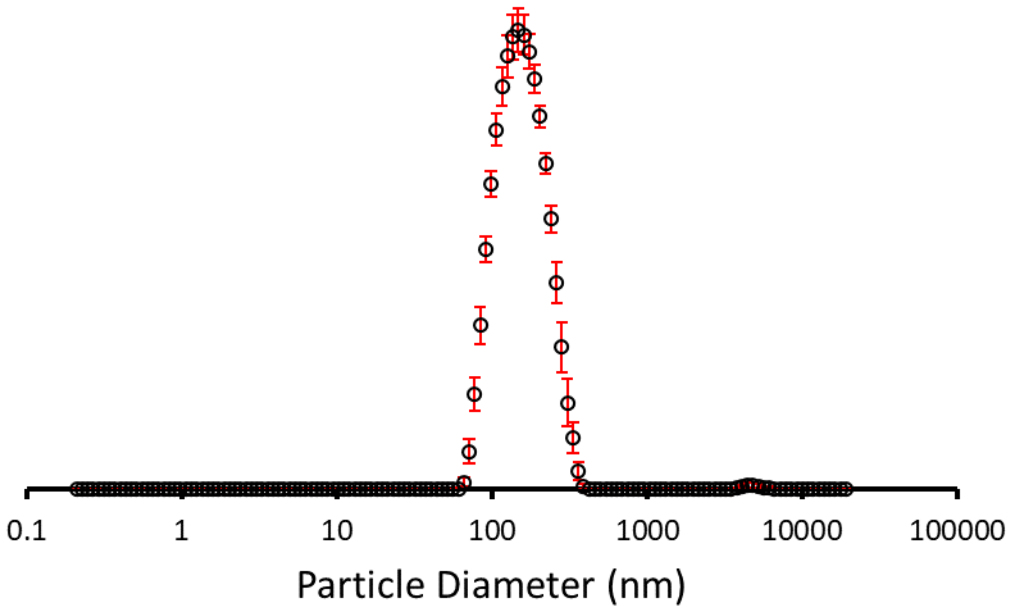

Figure S50 – The average intensity particle size distribution calculated (Peak maxima = 162 nm) using 10 DLS runs for **4** (2.78 mM) in an EtOH:H_2O (1:19) solution at 298 K.

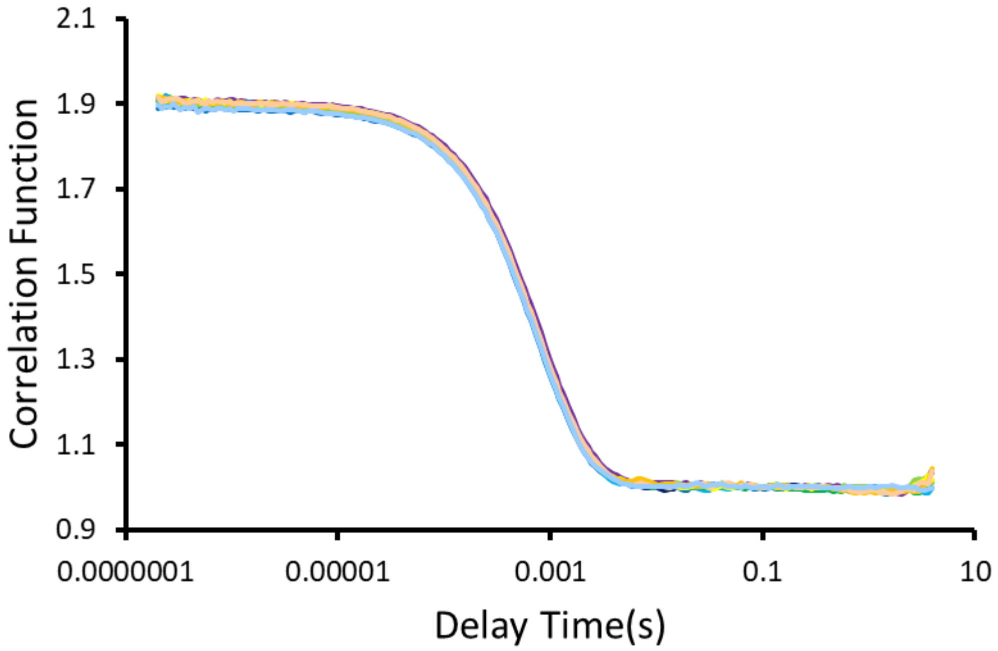

Figure S51 – Correlation function data for 10 DLS runs of **4** (2.78 mM) in an EtOH:H_2O (1:19) solution at 298 K.

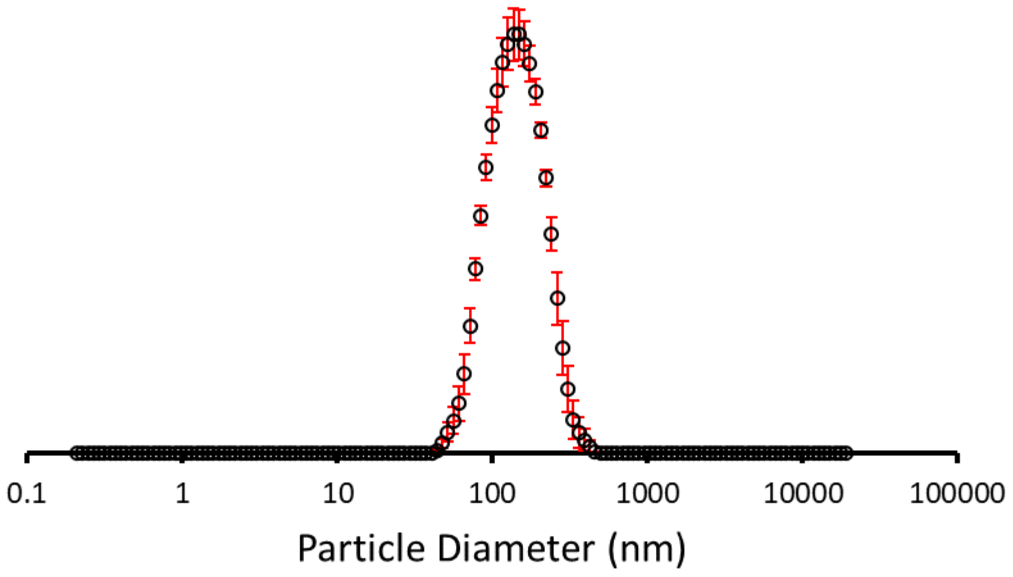

Figure S52– The average intensity particle size distribution calculated (Peak maxima = 151 nm) using 10 DLS runs for **4** (0.28 mM) in an EtOH:H_2O (1:19) solution at 298 K.

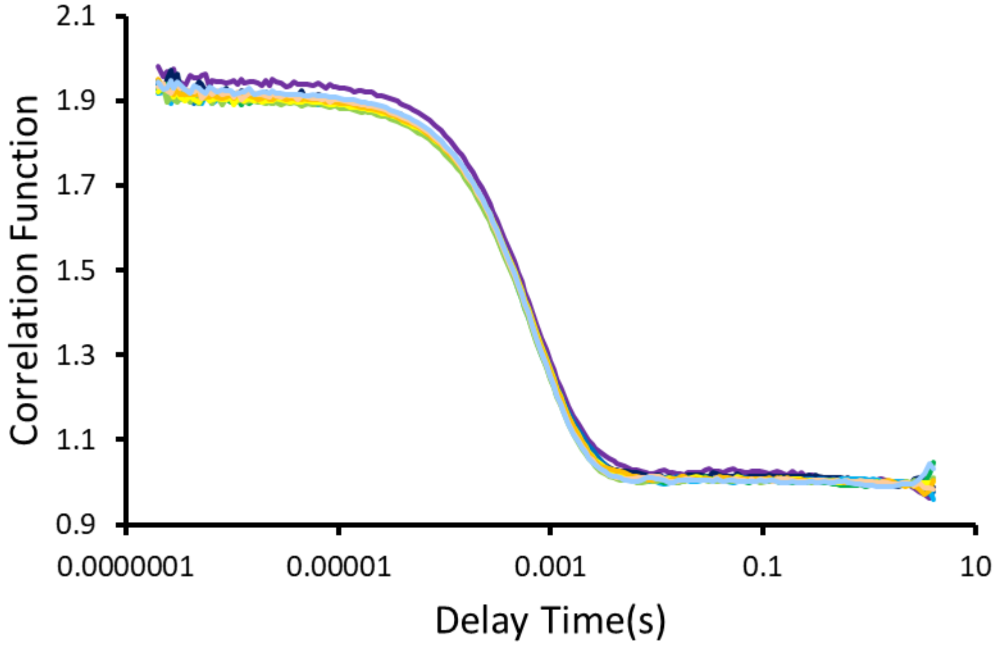

Figure S53 – Correlation function data for 10 DLS runs of **4** (0.28 mM) in an EtOH:H_2O (1:19) solution at 298 K.

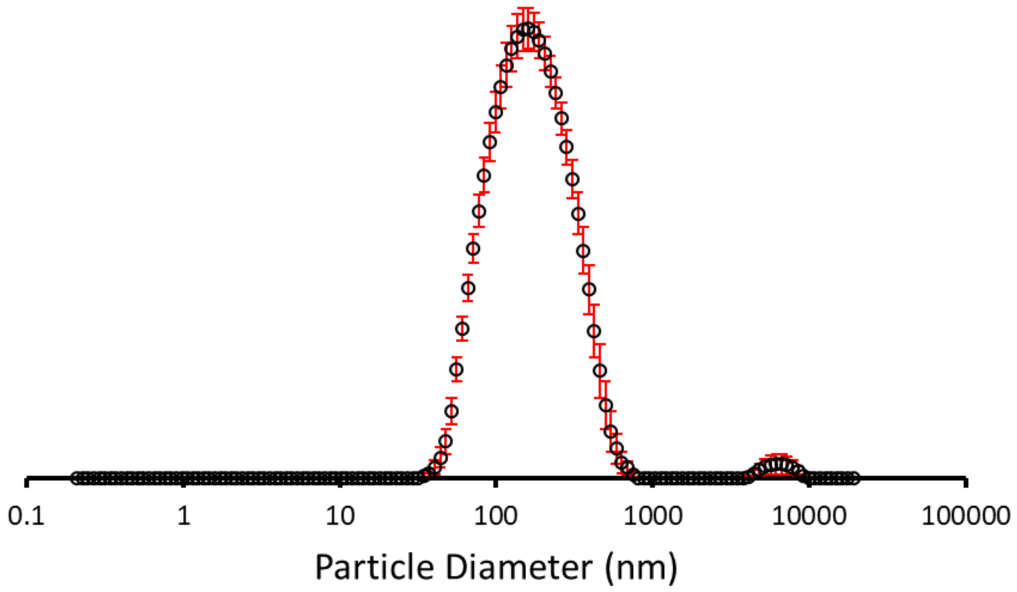

Figure S54 – The average intensity particle size distribution calculated (Peak maxima = 174 nm) using 10 DLS runs for **5** (2.78 mM) in an EtOH:H_2O (1:19) solution at 298 K.

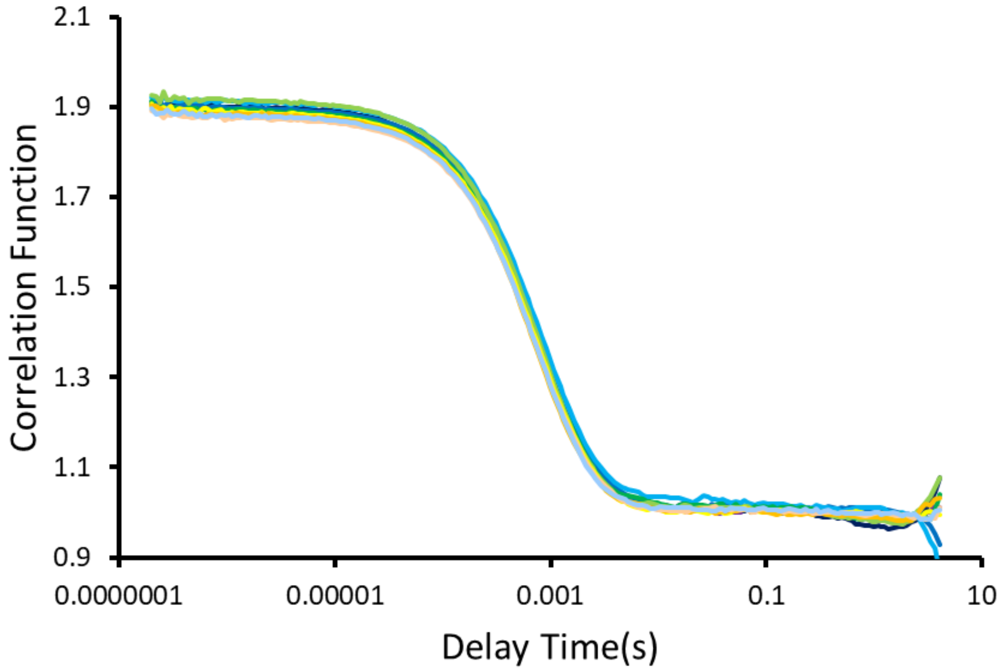

Figure S55 – Correlation function data for 10 DLS runs of **5** (2.78 mM) in an EtOH:H_2O (1:19) solution at 298 K.

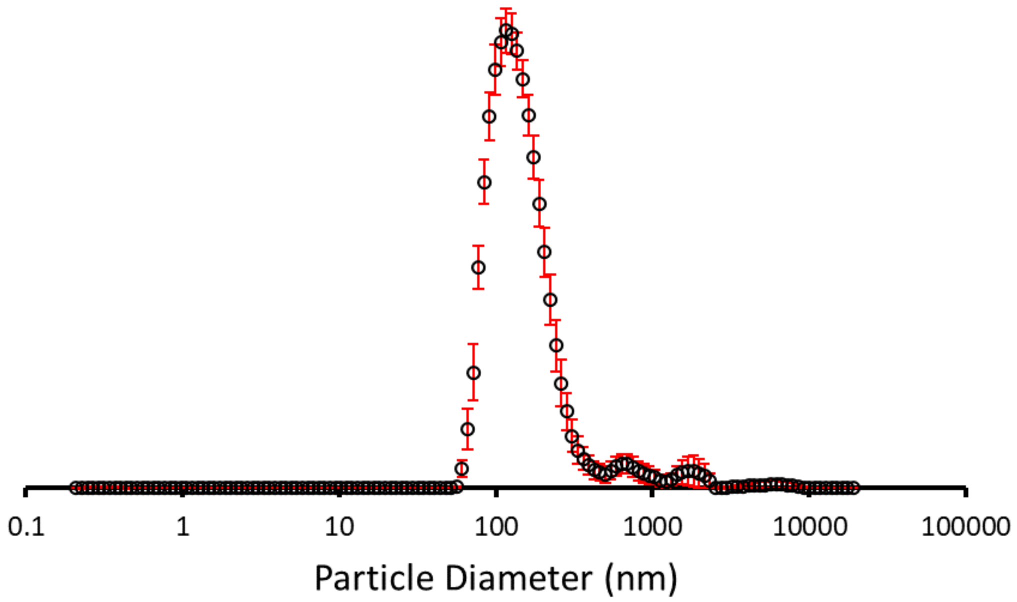

Figure S56 – The average intensity particle size distribution calculated (Peak maxima = 116 nm) using 10 DLS runs for **5** (0.28 mM) in an EtOH:H_2O (1:19) solution at 298 K.

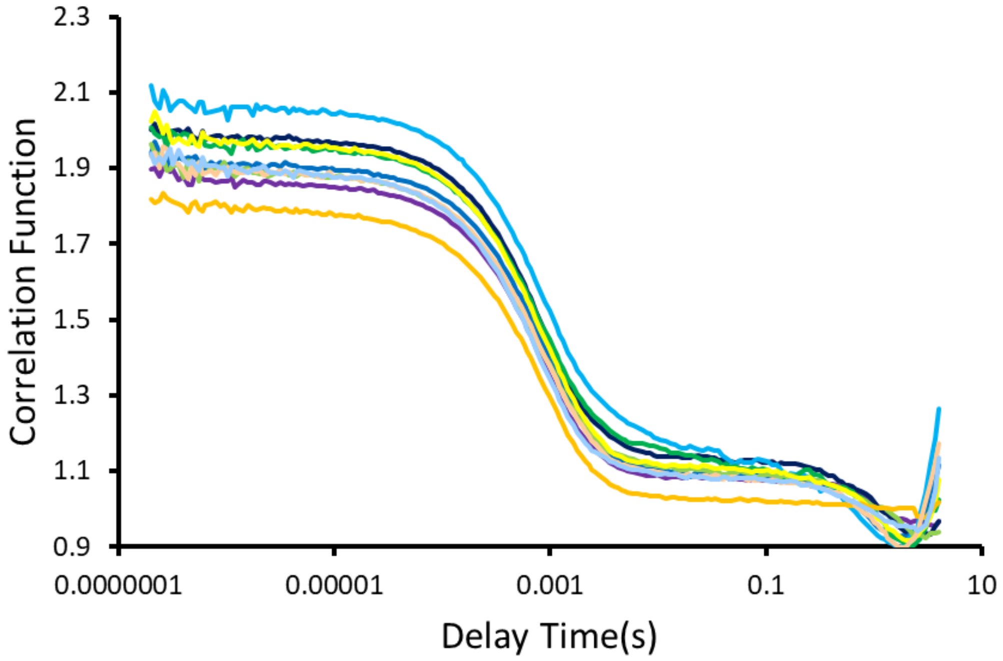

Figure S57 – Correlation function data for 10 DLS runs of **5** (0.28 mM) in an EtOH:H_2O (1:19) solution at 298 K.

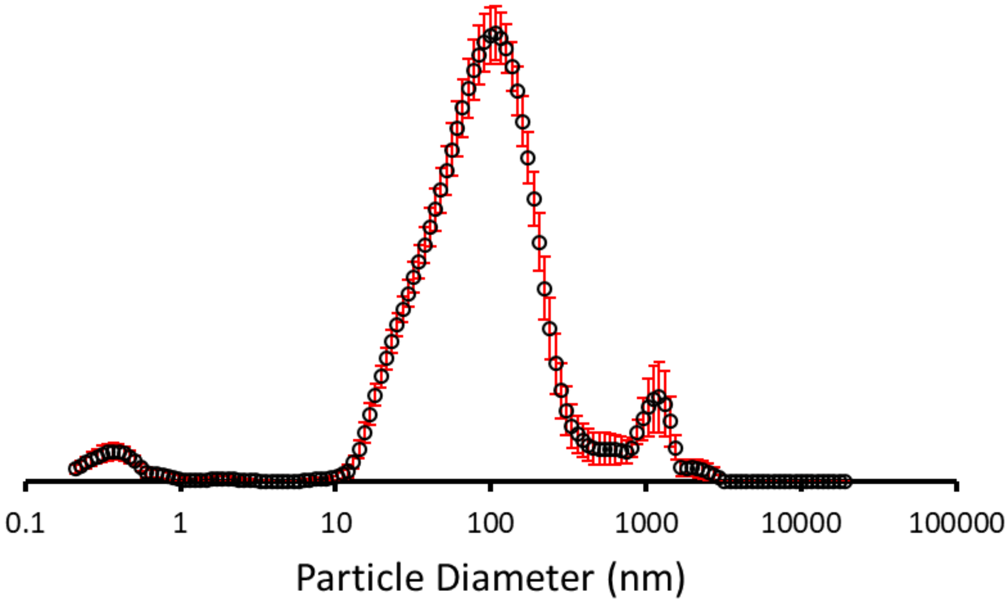

Figure S58 – The average intensity particle size distribution calculated (Peak maxima = 107 nm) using 10 DLS runs for co-formulation **a** (2.78 mM) in an EtOH:H_2O (1:19) solution at 298 K.

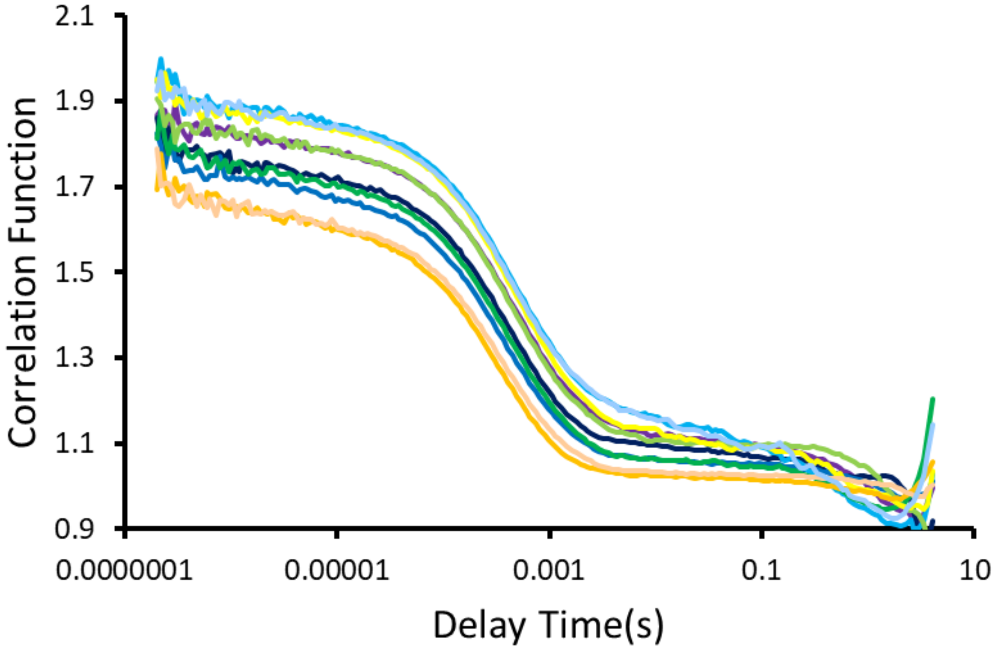

Figure S59 – Correlation function data for 10 DLS runs of co-formulation **a** (2.78 mM) in an EtOH:H_2O (1:19) solution at 298 K.

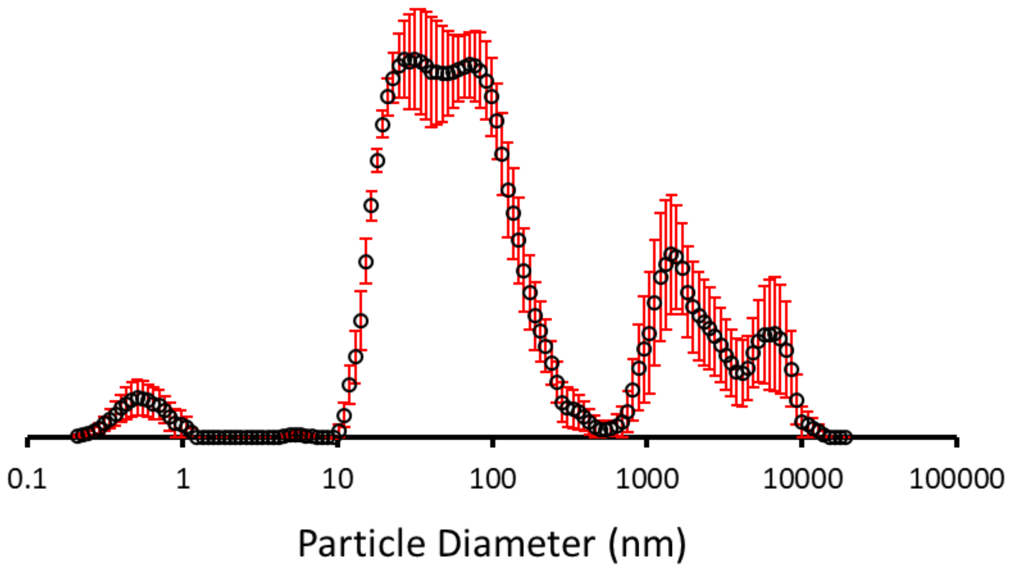

Figure S60 – The average intensity particle size distribution calculated (Peak maxima = 69 nm) using 10 DLS runs for co-formulation **a** (0.28 mM) in an EtOH:H_2O (1:19) solution at 298 K.

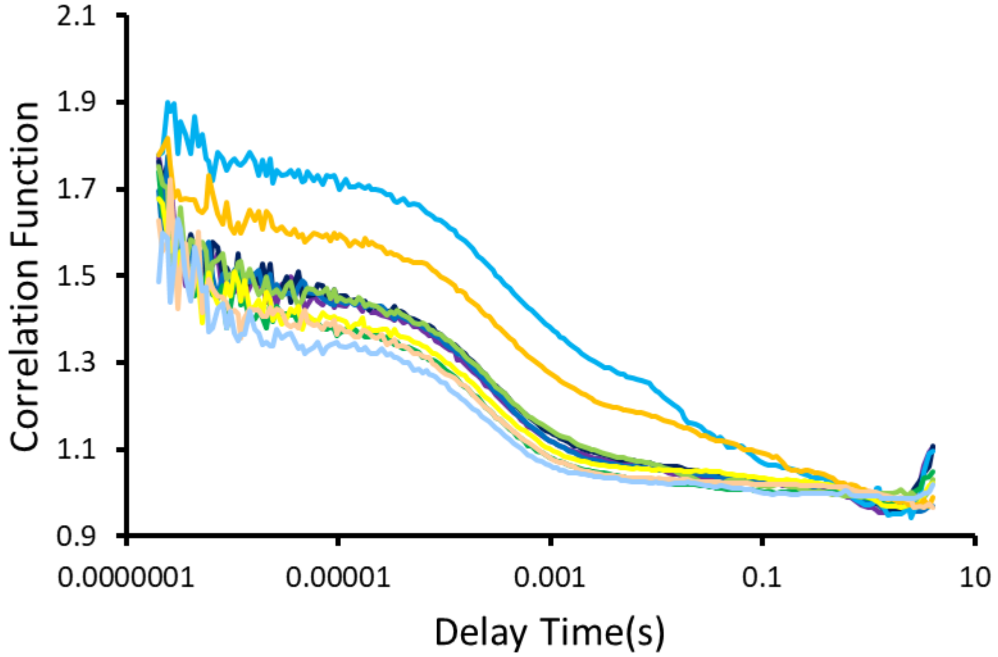

Figure S61 – Correlation function data for 10 DLS runs of co-formulation **a** (0.28 mM) in an EtOH:H_2O (1:19) solution at 298 K.

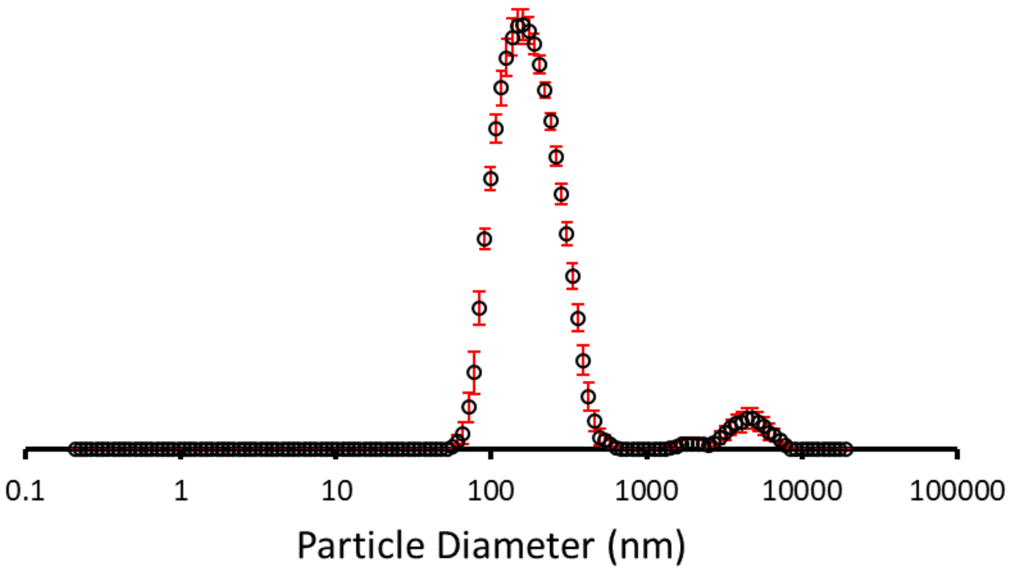

Figure S62 – The average intensity particle size distribution calculated (Peak maxima = 187.24 nm) using 10 DLS runs for co-formulation **b** (2.78 mM) in an EtOH:H_2O (1:19) solution at 298 K.

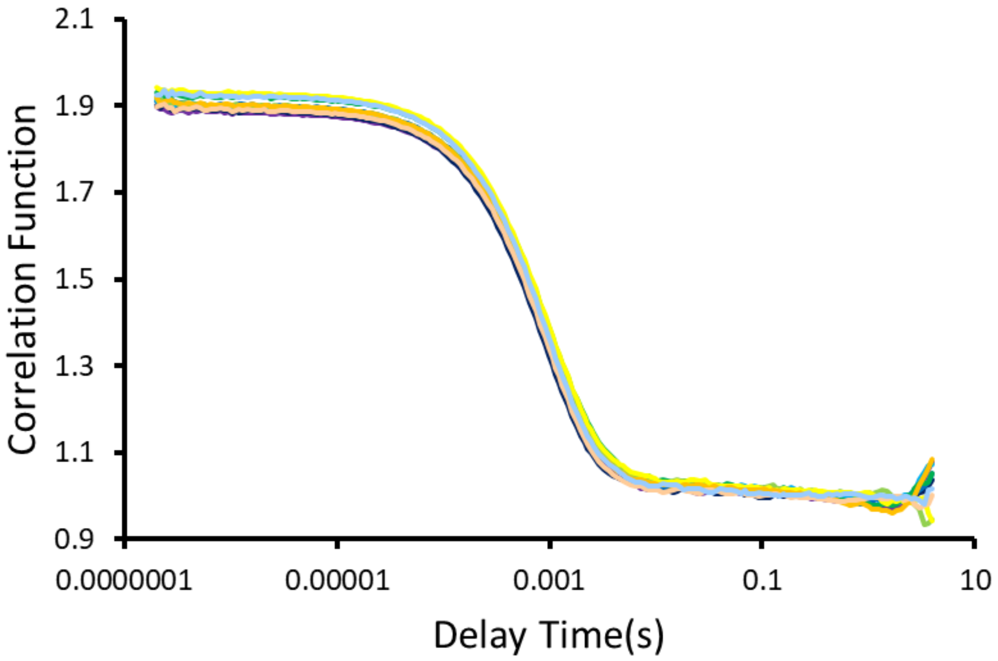

Figure S63 – Correlation function data for 10 DLS runs of co-formulation **b** (2.78 mM) in an EtOH:H_2O (1:19) solution at 298 K.

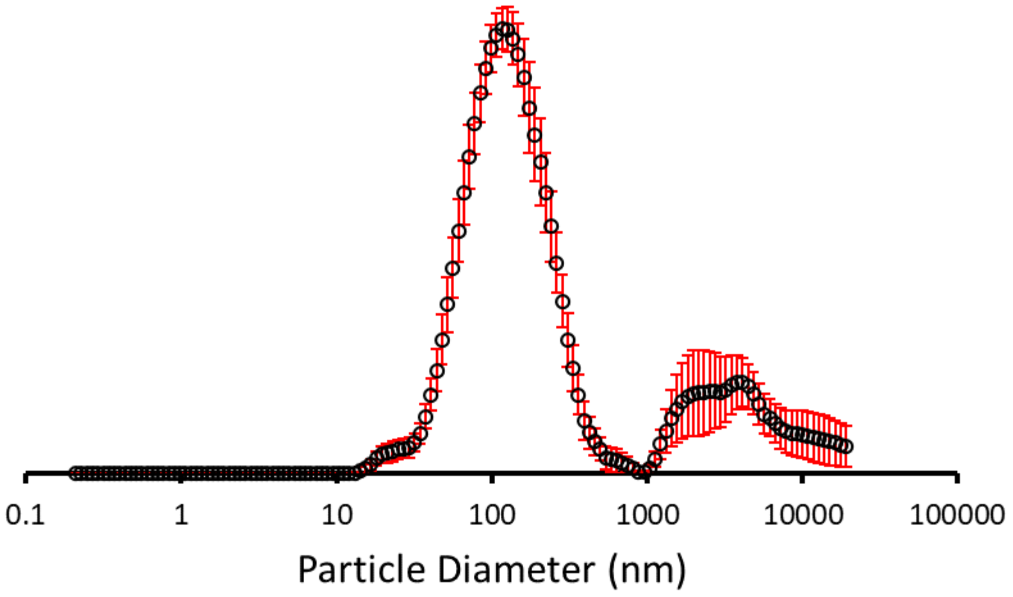

Figure S64 – The average intensity particle size distribution calculated (Peak maxima = 307 nm) using 10 DLS runs for co-formulation **b** (0.28 mM) in an EtOH:H_2O (1:19) solution at 298 K.

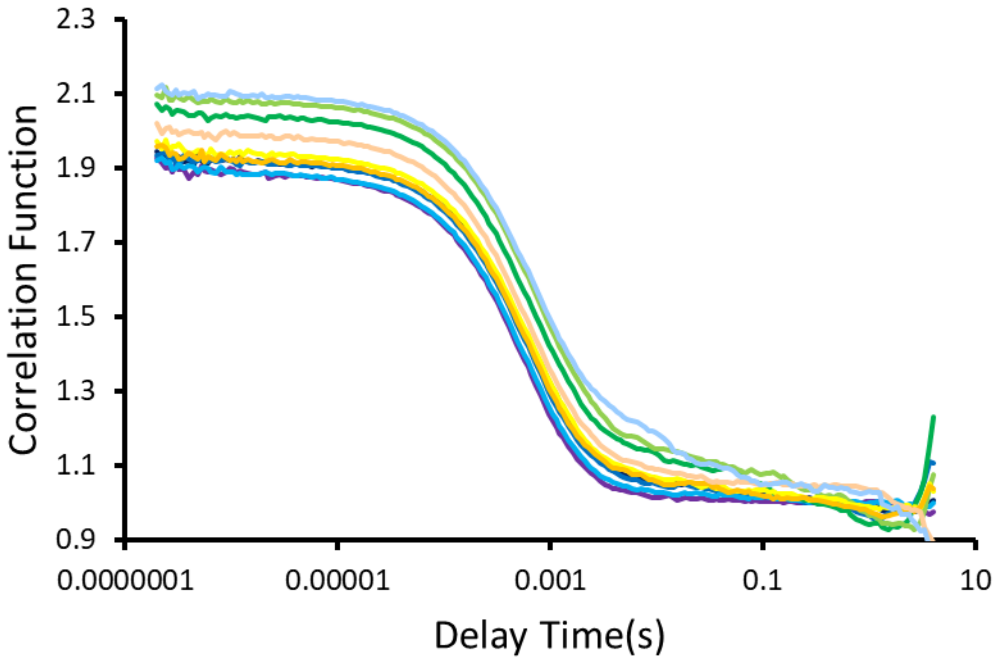

Figure S65 – Correlation function data for 10 DLS runs of co-formulation **b** (0.56 mM) in an EtOH:H_2O (1:19) solution at 298 K.

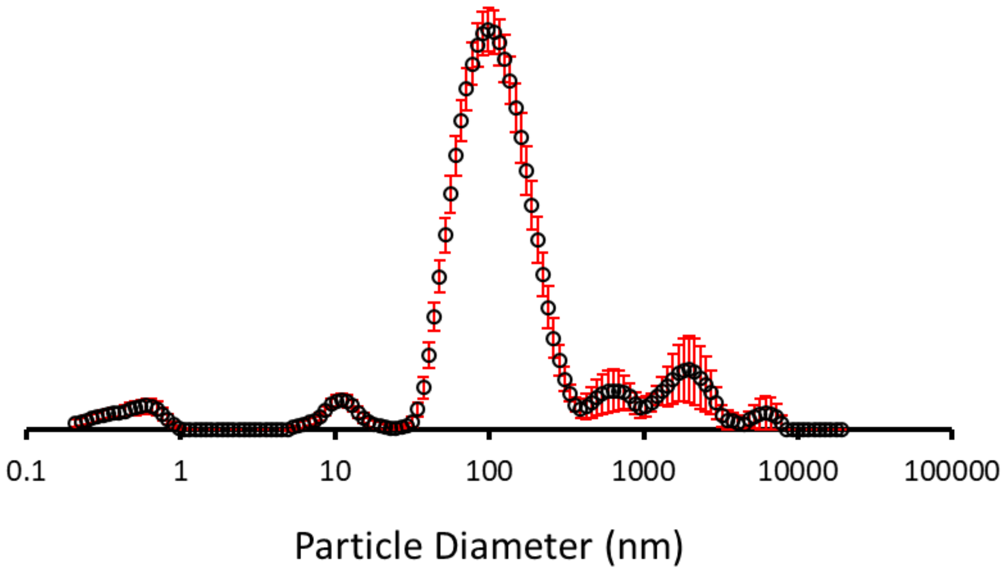

Figure S66 – The average intensity particle size distribution calculated (Peak maxima = 107 nm) using 10 DLS runs for co-formulation **c** (2.78 mM) in an EtOH:H_2O (1:19) solution at 298 K.

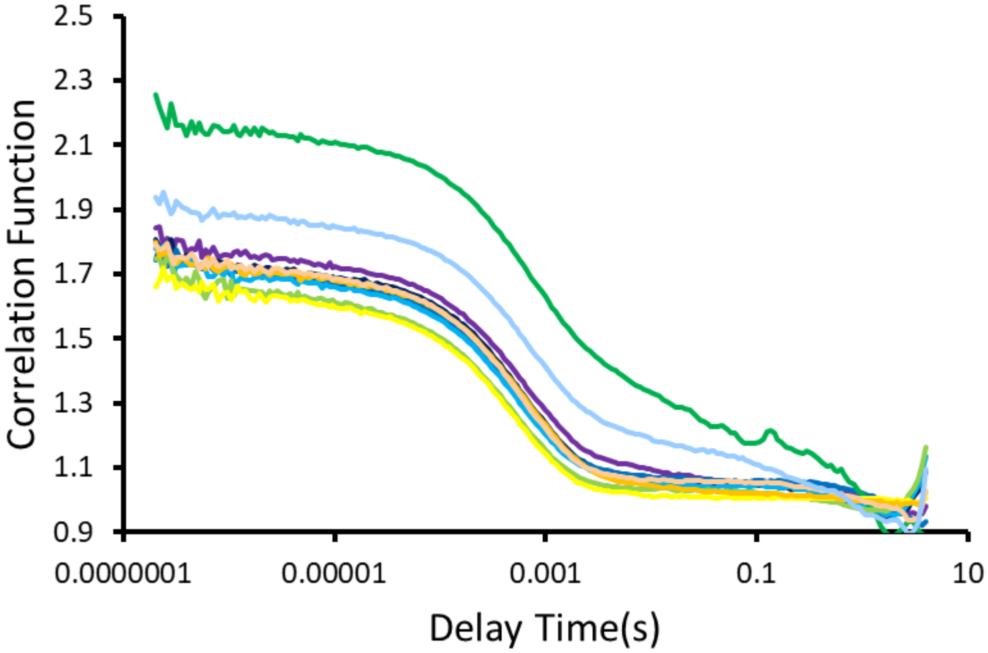

Figure S67 – Correlation function data for 10 DLS runs of co-formulation **c** (2.78 mM) in an EtOH:H_2O (1:19) solution at 298 K.

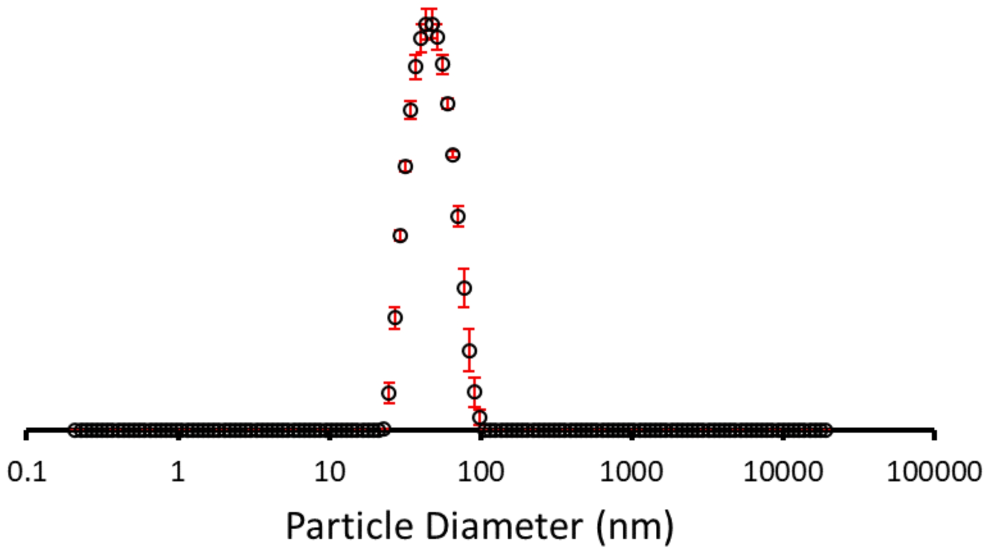

Figure S68 – The average intensity particle size distribution calculated (Peak maxima = 49 nm) using 10 DLS runs for co-formulation **c** (0.28 mM) in an EtOH:H_2O (1:19) solution at 298 K.

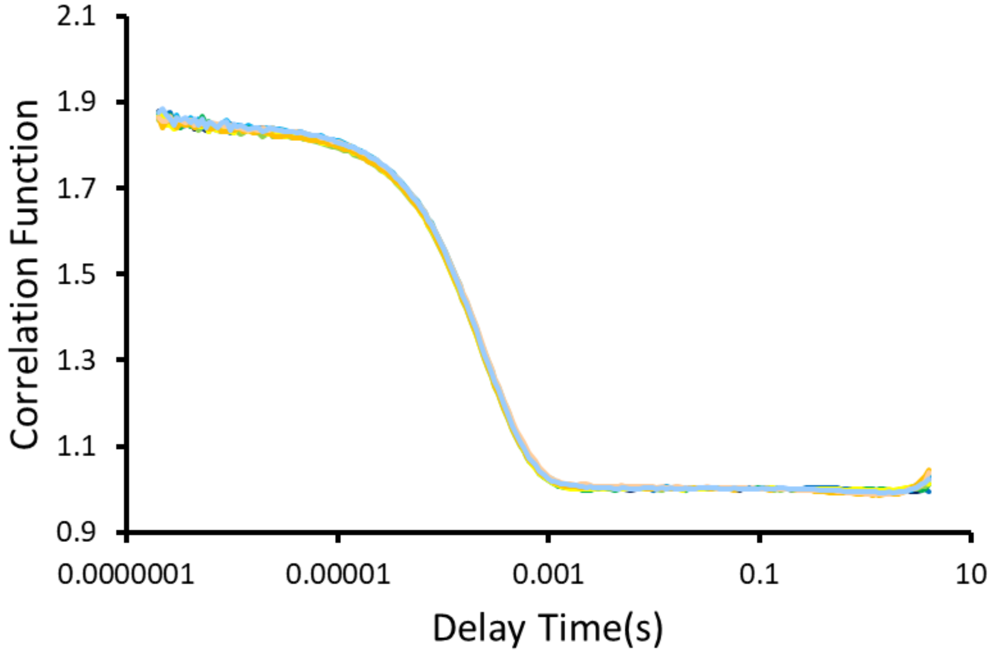

Figure S69 – Correlation function data for 10 DLS runs of co-formulation **c** (0.28 mM) in an EtOH:H_2O (1:19) solution at 298 K.

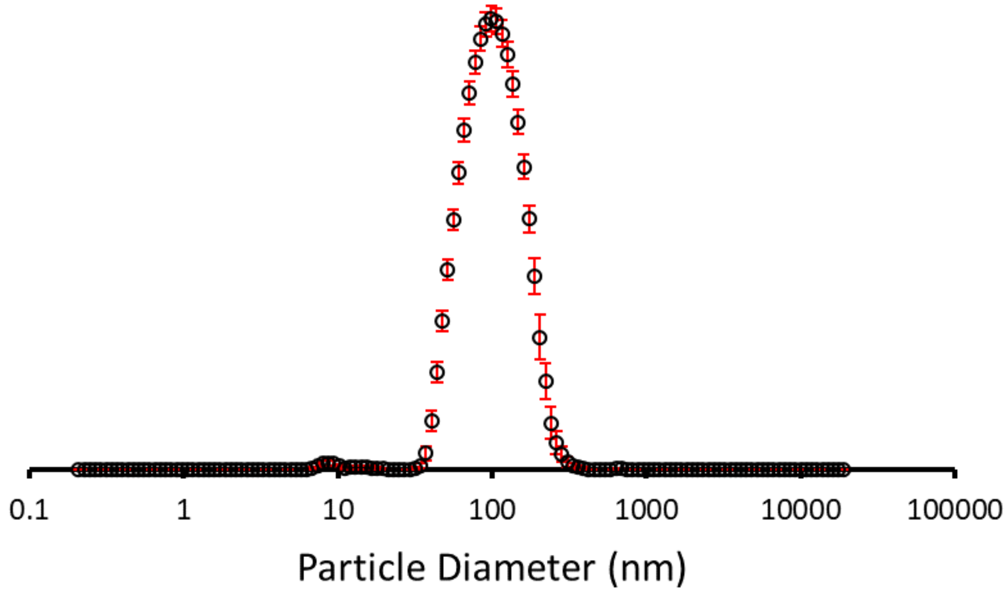

Figure S70 – The average intensity particle size distribution calculated (Peak maxima = 98 nm) using 10 DLS runs for co-formulation **d** (2.78 mM) in an EtOH:H_2O (1:19) solution at 298 K.

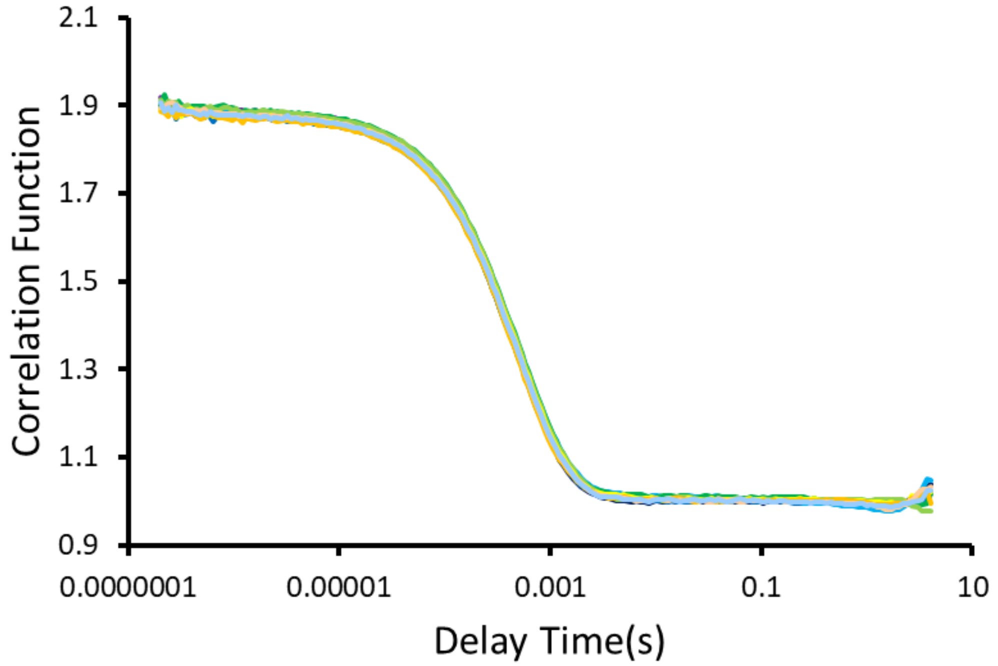

Figure S71 – Correlation function data for 10 DLS runs of co-formulation **d** (2.78 mM) in an EtOH:H_2O (1:19) solution at 298 K.

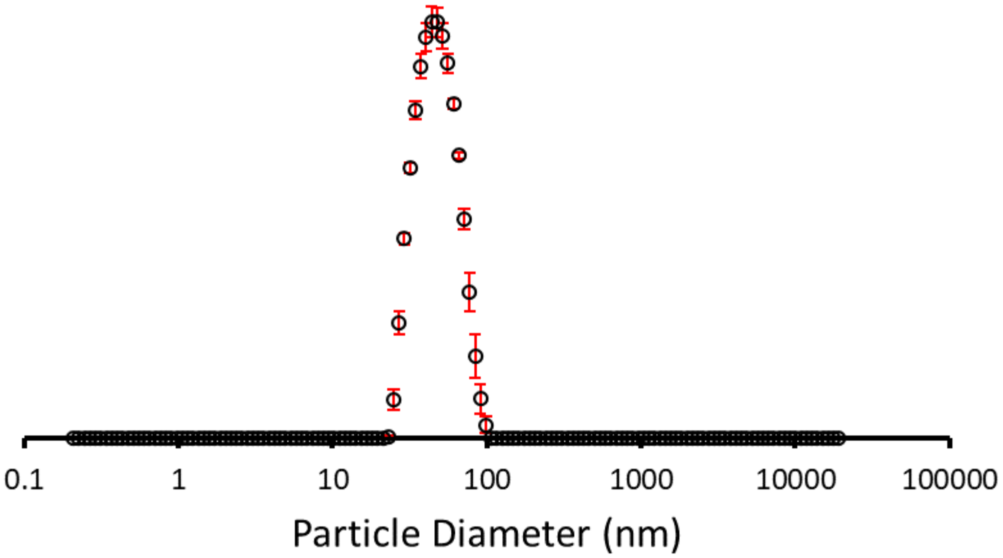

Figure S72 – The average intensity particle size distribution calculated (Peak maxima = 49 nm) using 10 DLS runs for co-formulation **d** (0.28 mM) in an EtOH:H_2O (1:19) solution at 298 K.

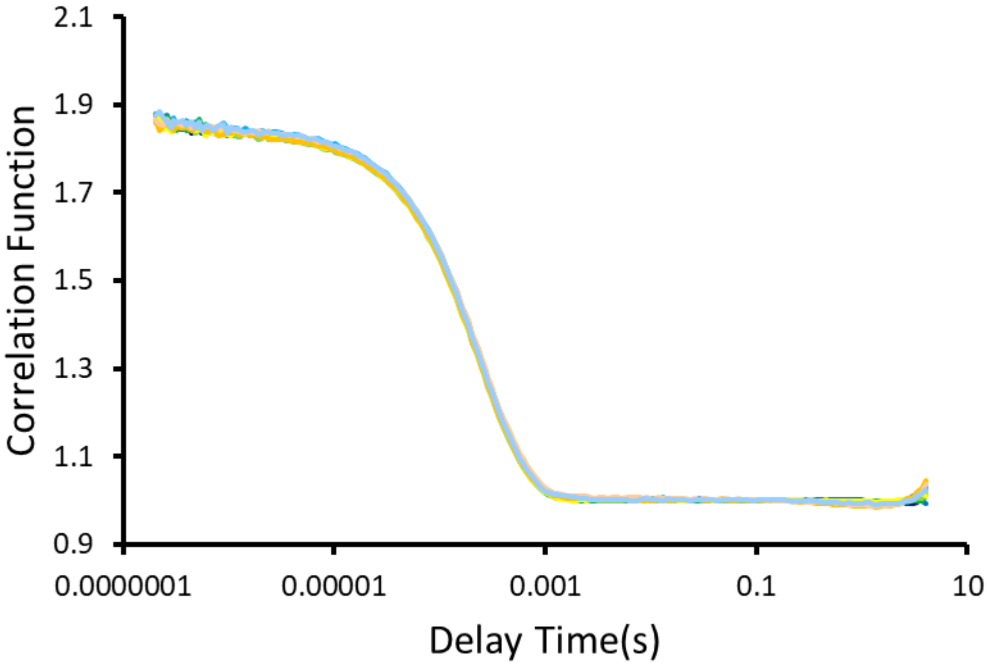

Figure S73 – Correlation function data for 10 DLS runs of co-formulation **d** (0.28 mM) in an EtOH:H_2O (1:19) solution at 298 K.

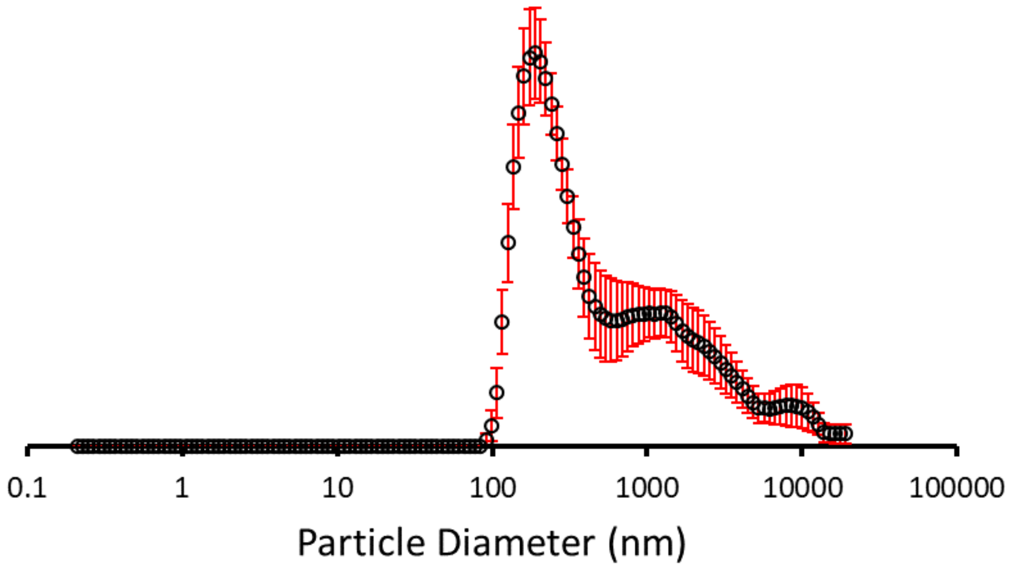

Figure S74 – The average intensity particle size distribution calculated (Peak maxima = 189 nm) using 10 DLS runs for co-formulation **e** (2.78 mM) in an EtOH:H_2O (1:19) solution at 298 K.

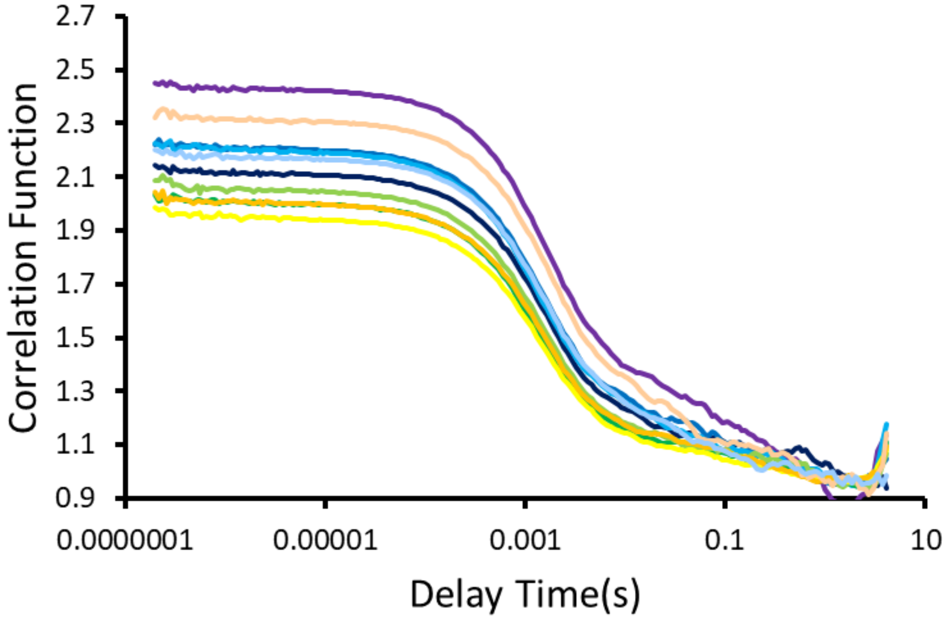

Figure S75 – Correlation function data for 10 DLS runs of co-formulation **e** (2.78 mM) in an EtOH:H_2O (1:19) solution at 298 K.

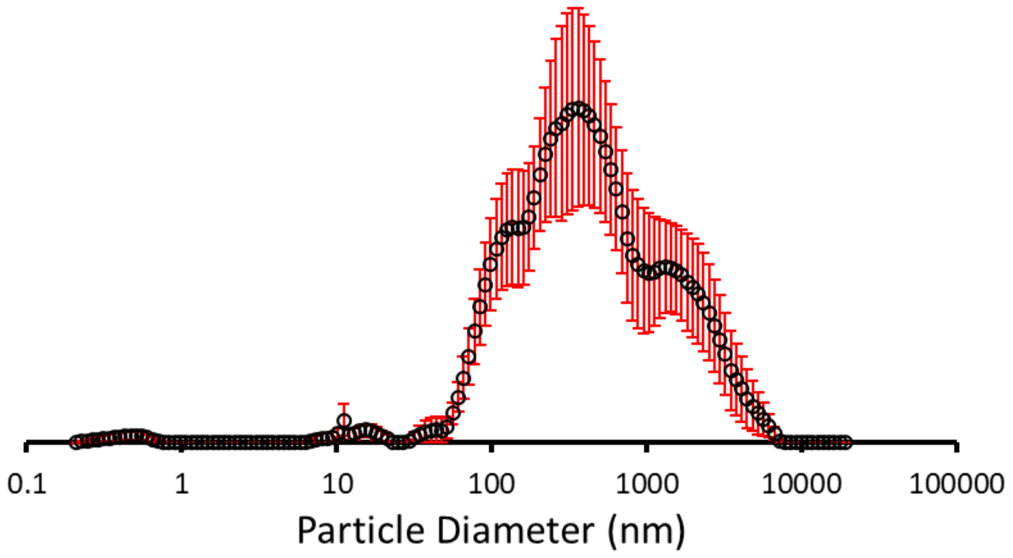

Figure S76 – The average intensity particle size distribution calculated (Peak maxima = 391 nm) using 10 DLS runs for co-formulation **e** (0.28 mM) in an EtOH:H_2O (1:19) solution at 298 K.

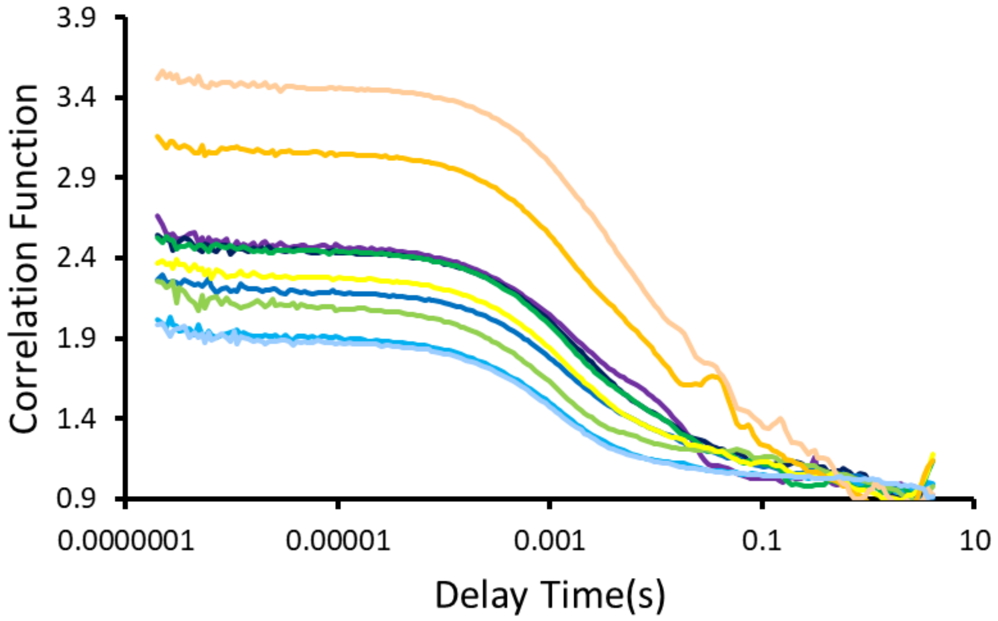

Figure S77 – Correlation function data for 10 DLS runs of co-formulation **e** (0.28 mM) in an EtOH:H_2O (1:19) solution at 298 K.

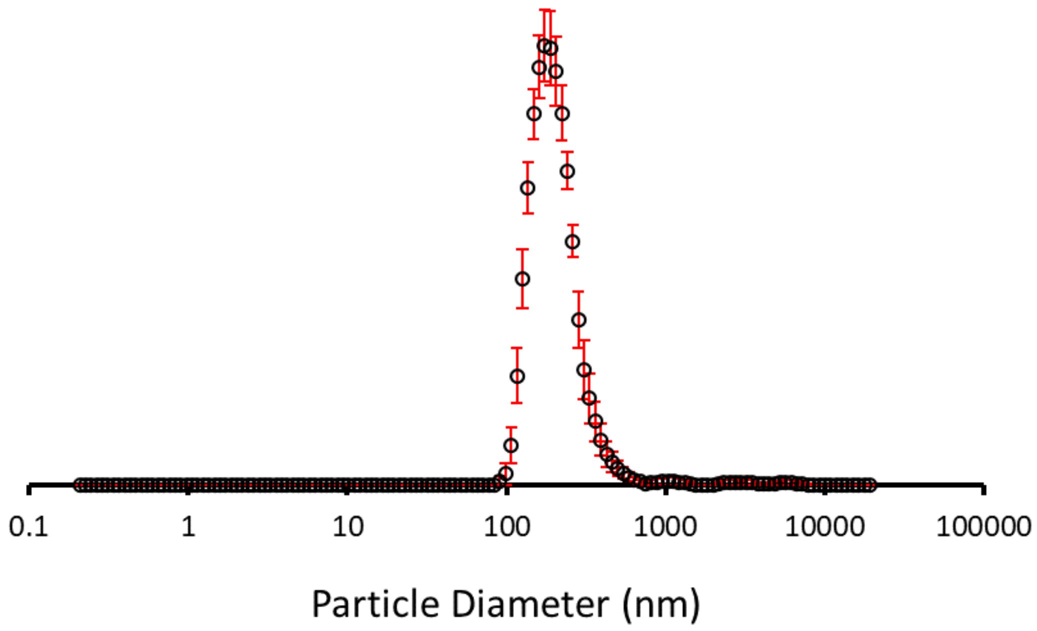

Figure S78 – The average intensity particle size distribution calculated (Peak maxima = 181 nm) using 10 DLS runs for co-formulation **f** (2.78 mM) in an EtOH:H_2O (1:19) solution at 298 K.

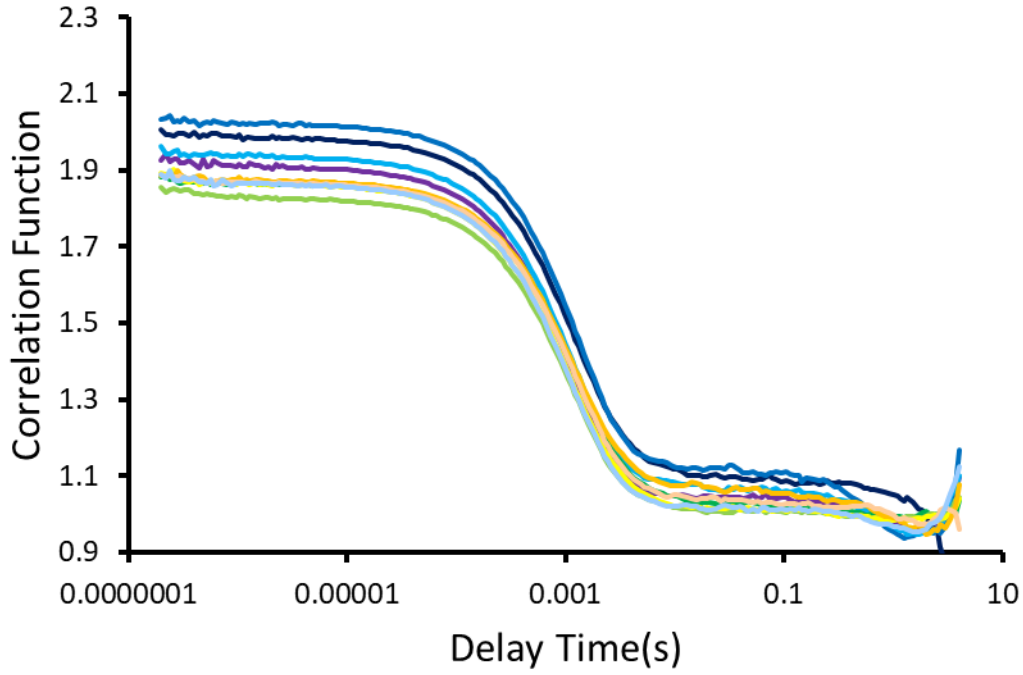

Figure S79 – Correlation function data for 10 DLS runs of co-formulation **f** (2.78 mM) in an EtOH:H_2O (1:19) solution at 298 K.

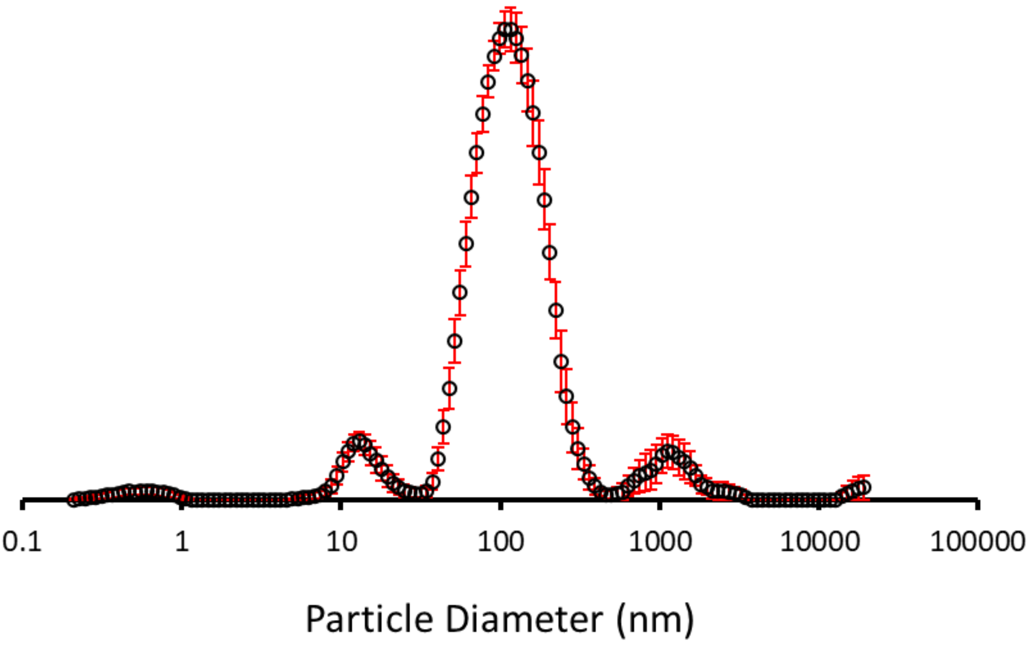

Figure S80 – The average intensity particle size distribution calculated (Peak maxima = 261 nm) using 10 DLS runs for co-formulation **f** (0.28 mM) in an EtOH:H_2O (1:19) solution at 298 K.

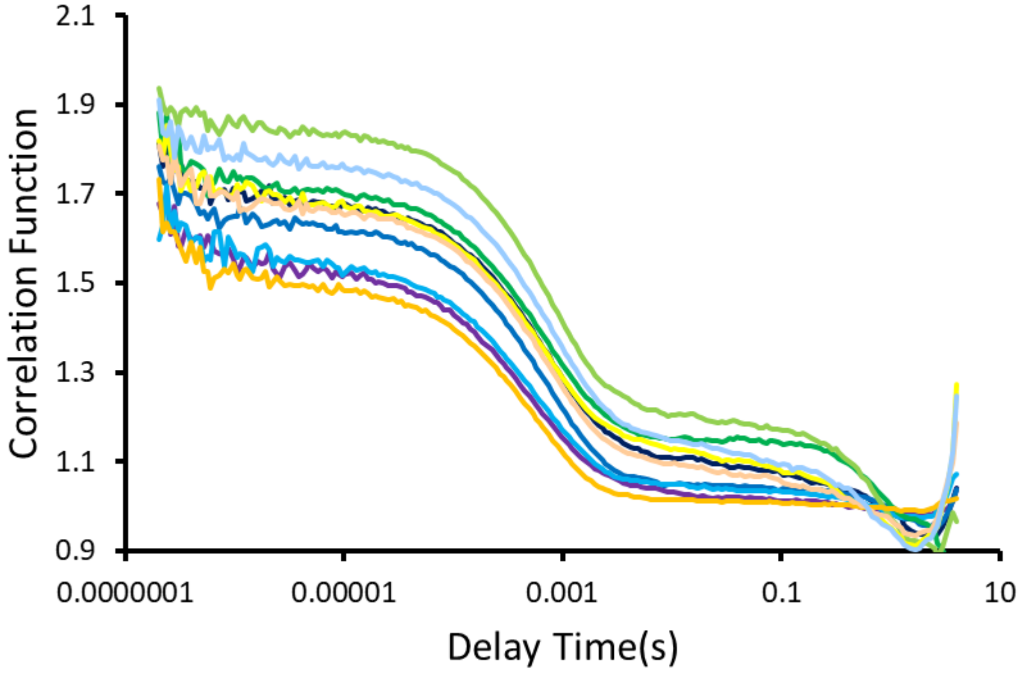

Figure S81 – Correlation function data for 10 DLS runs of co-formulation **f** (0.28 mM) in an EtOH:H_2O (1:19) solution at 298 K.

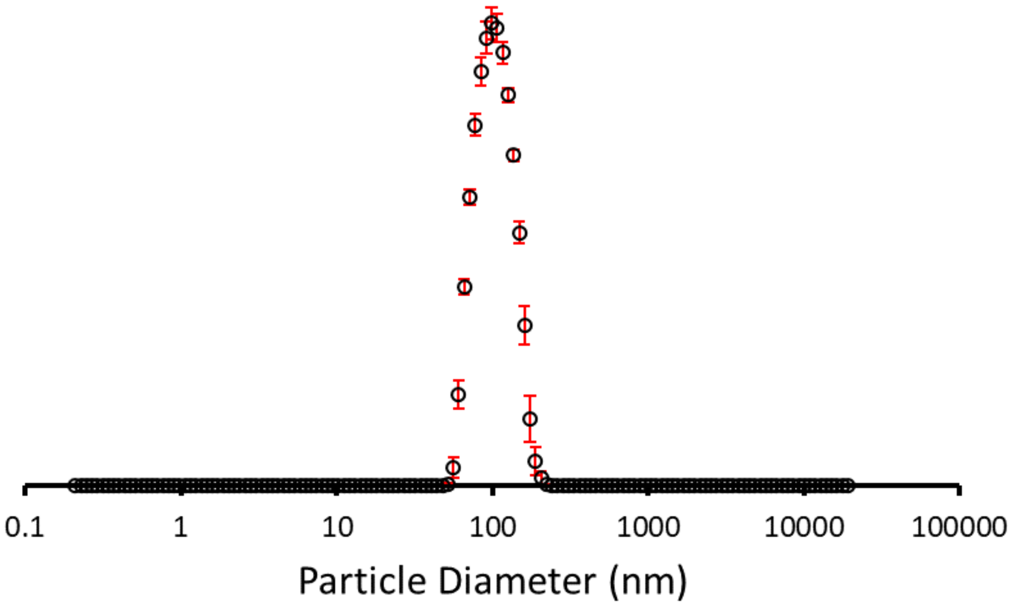

Figure S82 – The average intensity particle size distribution calculated (Peak maxima = 105 nm) using 10 DLS runs for co-formulation **g** (2.78 mM) in an EtOH:H_2O (1:19) solution at 298 K.

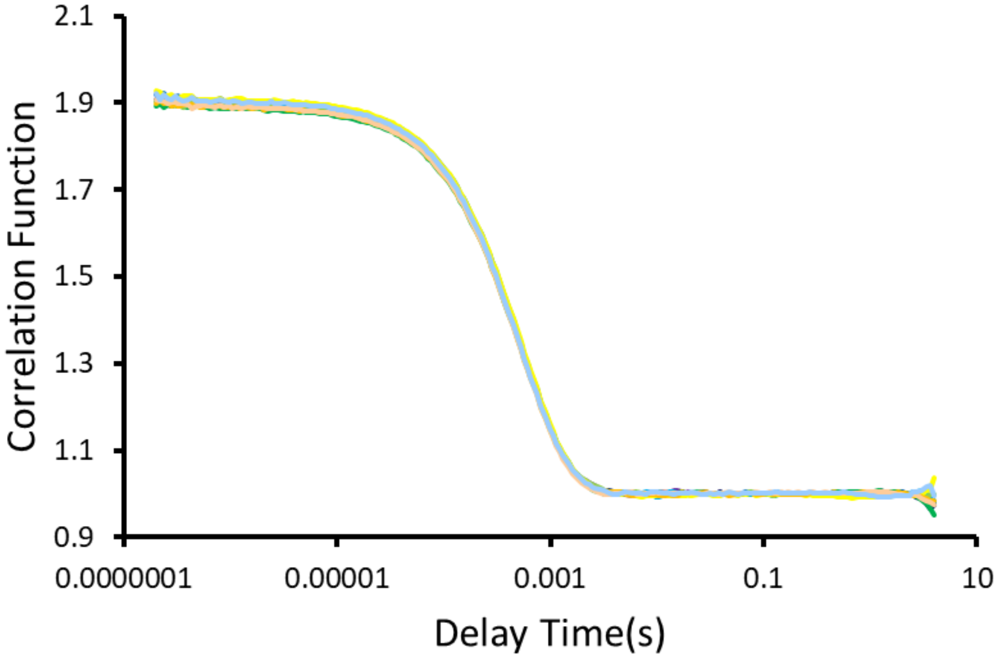

Figure S83 – Correlation function data for 10 DLS runs of co-formulation **g** (2.78 mM) in an EtOH:H_2O (1:19) solution at 298 K.

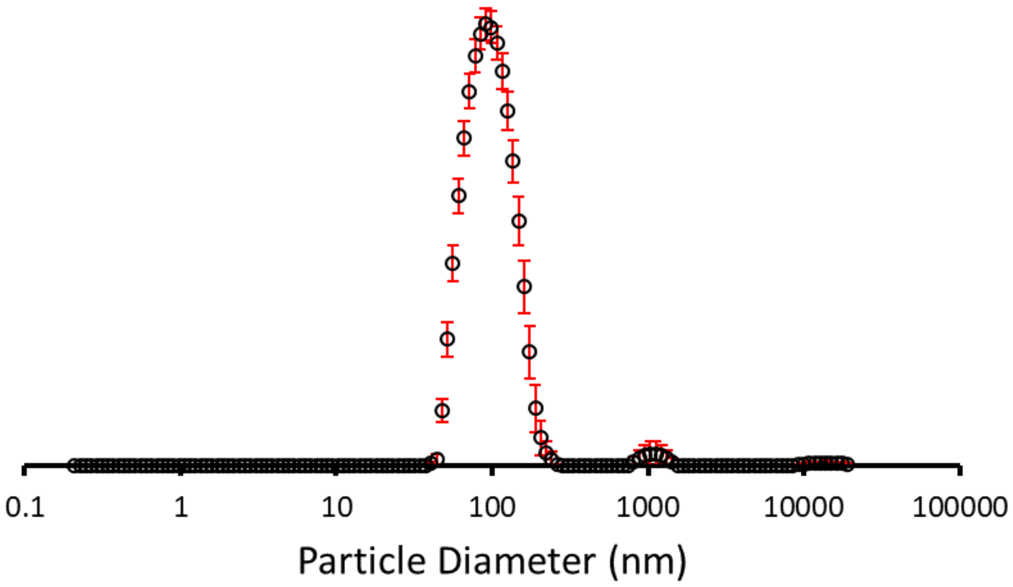

Figure S84 – The average intensity particle size distribution calculated (Peak maxima = 100 nm) using 10 DLS runs for co-formulation **g** (0.28 mM) in an EtOH:H_2O (1:19) solution at 298 K.

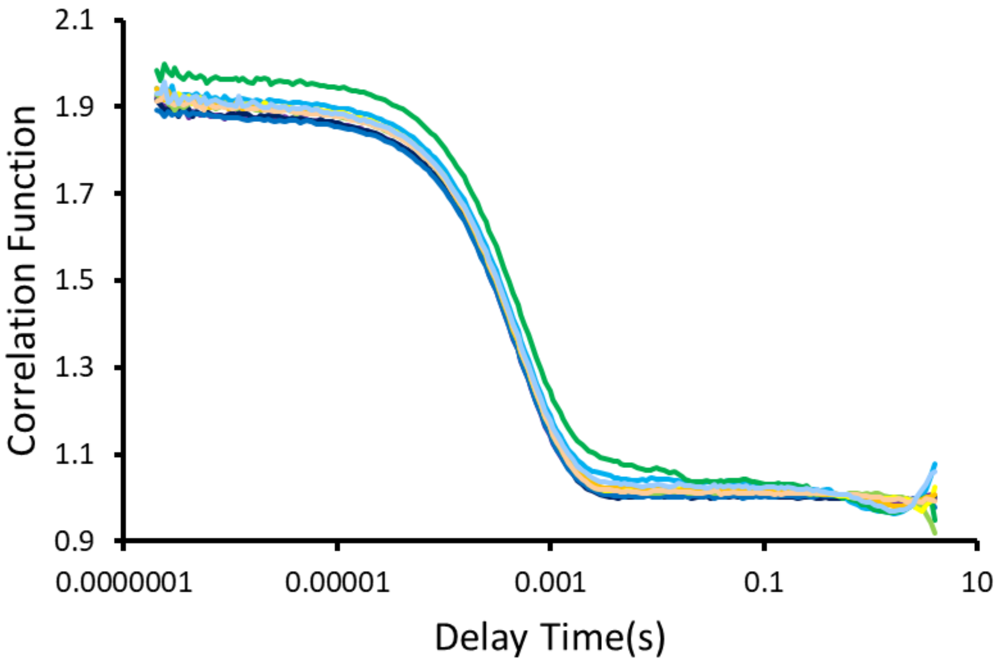

Figure S85 – Correlation function data for 10 DLS runs of co-formulation **g** (0.28 mM) in an EtOH:H_2O (1:19) solution at 298 K.

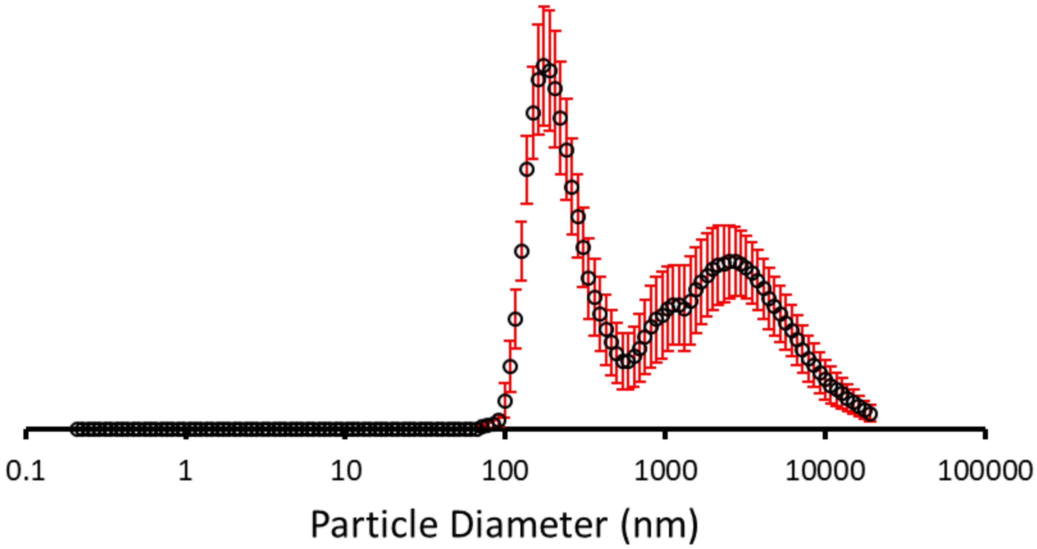

Figure S86 – The average intensity particle size distribution calculated (Peak maxima = 190 nm) using 10 DLS runs for co-formulation **h** (2.78 mM) in an EtOH:H_2O (1:19) solution at 298 K.

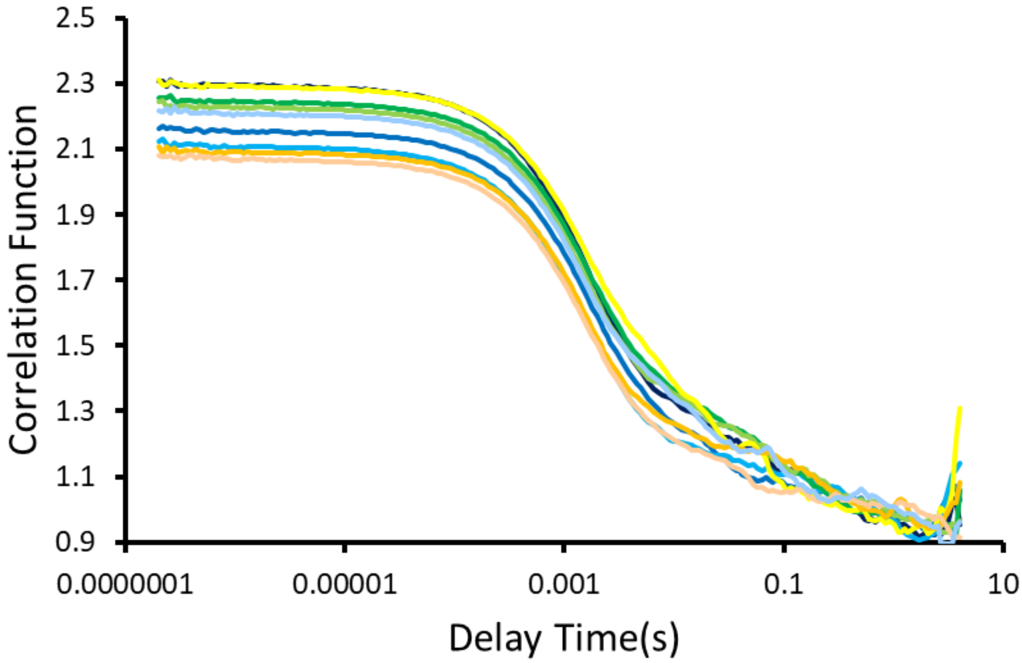

Figure S87 – Correlation function data for 10 DLS runs of co-formulation **h** (2.78 mM) in an EtOH:H_2O (1:19) solution at 298 K.

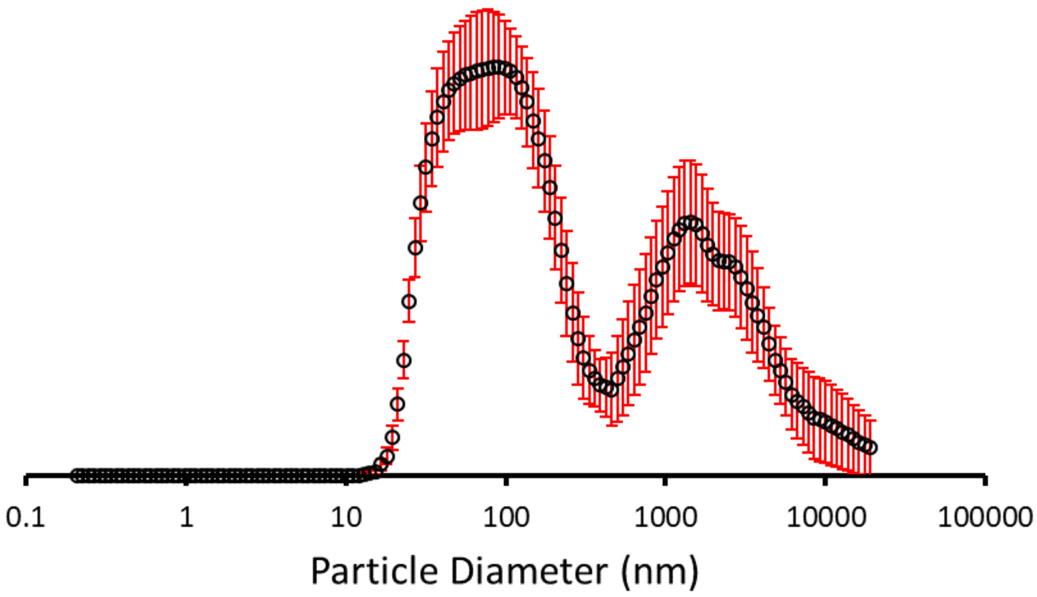

Figure S88 – The average intensity particle size distribution calculated Peak maxima = (84 nm) using 10 DLS runs for co-formulation **h** (0.28 mM) in an EtOH:H_2O (1:19) solution at 298 K.

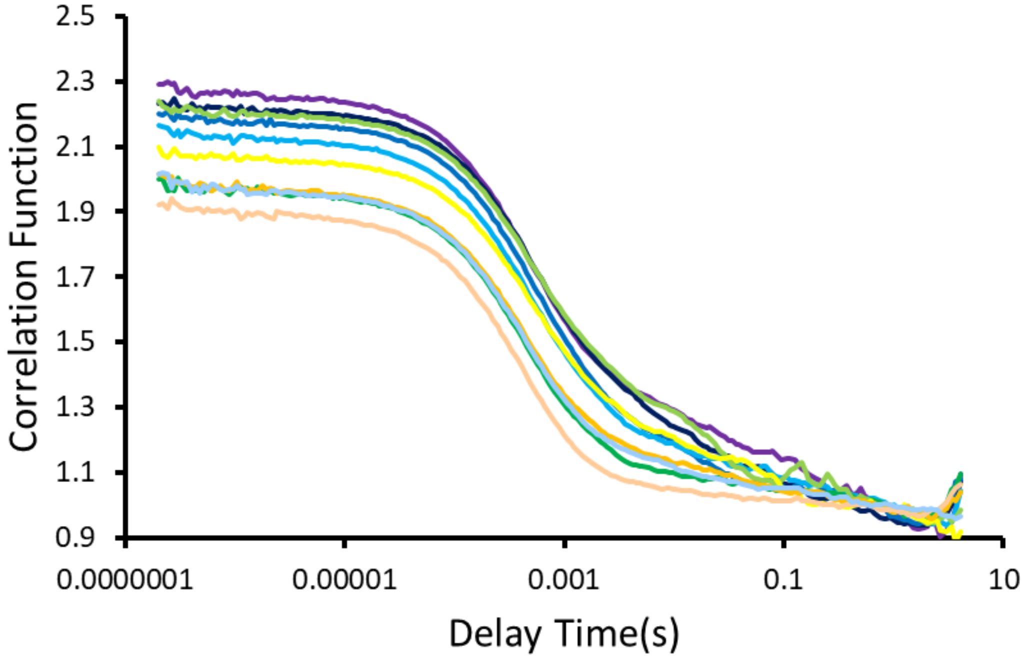

Figure S89 – Correlation function data for 10 DLS runs of co-formulation **h** (0.28 mM) in an EtOH:H_2O (1:19) solution at 298 K.

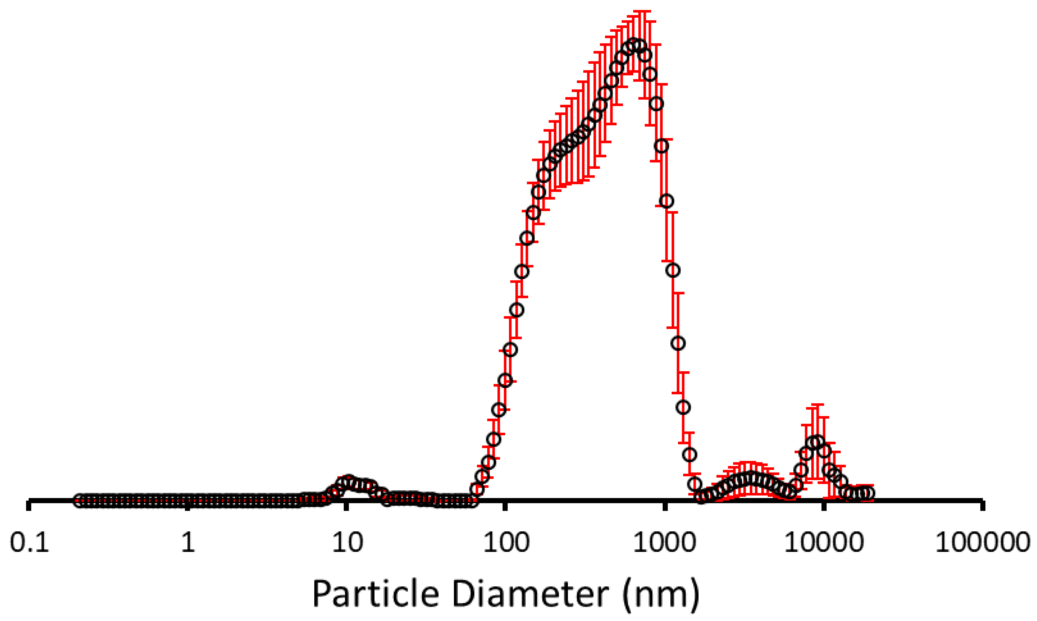

Figure S90 – The average intensity particle size distribution calculated (Peak maxima = 690 nm) using 10 DLS runs for co-formulation **i** (2.78 mM) in an EtOH:H_2O (1:19) solution at 298 K.

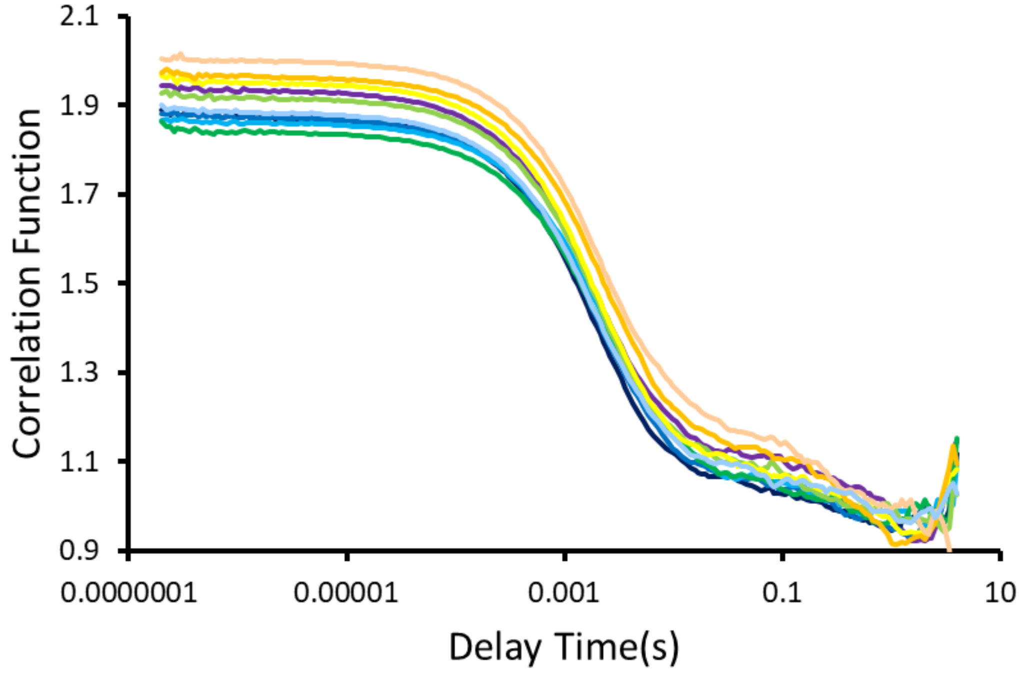

Figure S91 – Correlation function data for 10 DLS runs of co-formulation **i** (2.78 mM) in an EtOH:H_2O (1:19) solution at 298 K.

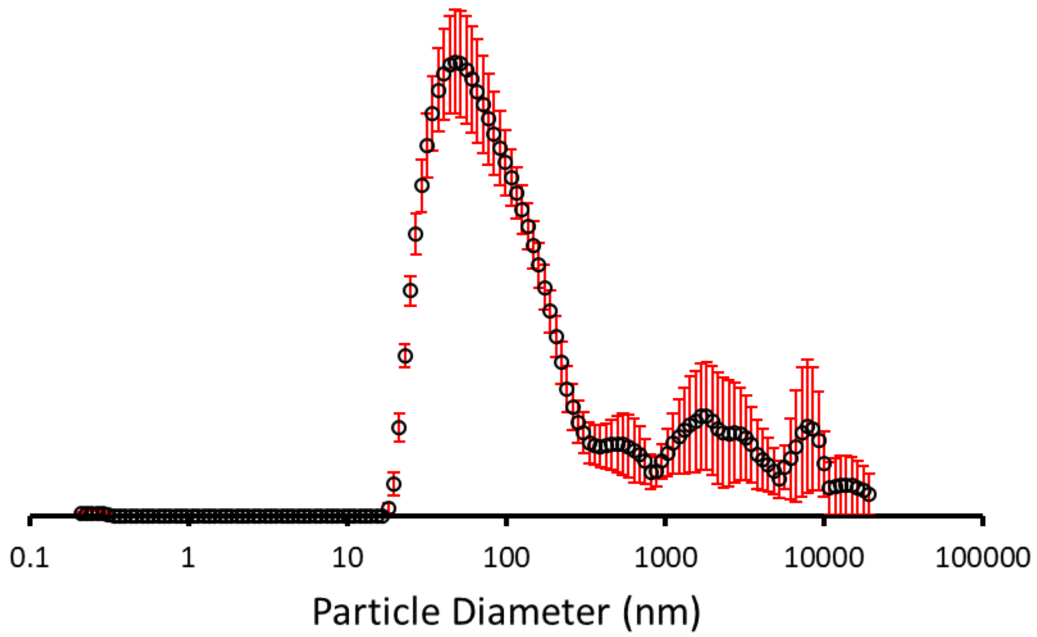

Figure S92 – The average intensity particle size distribution calculated (Peak maxima = 53 nm) using 10 DLS runs for co-formulation **i** (0.28 mM) in an EtOH:H_2O (1:19) solution at 298 K.

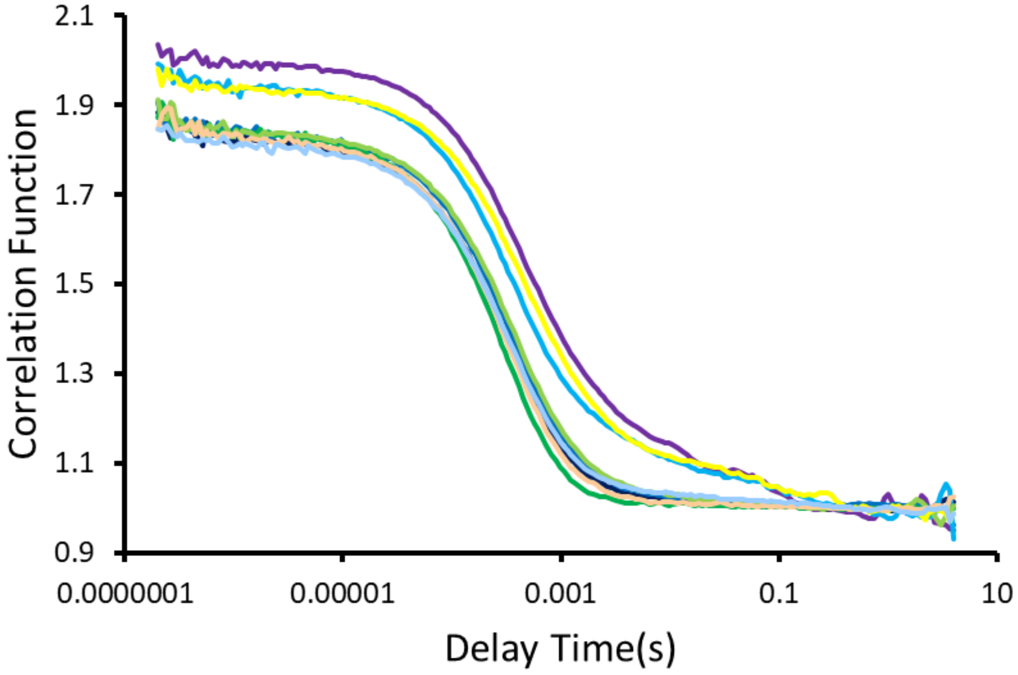

Figure S93 – Correlation function data for 10 DLS runs of co-formulation **i** (0.28 mM) in an EtOH:H_2O (1:19) solution at 298 K.

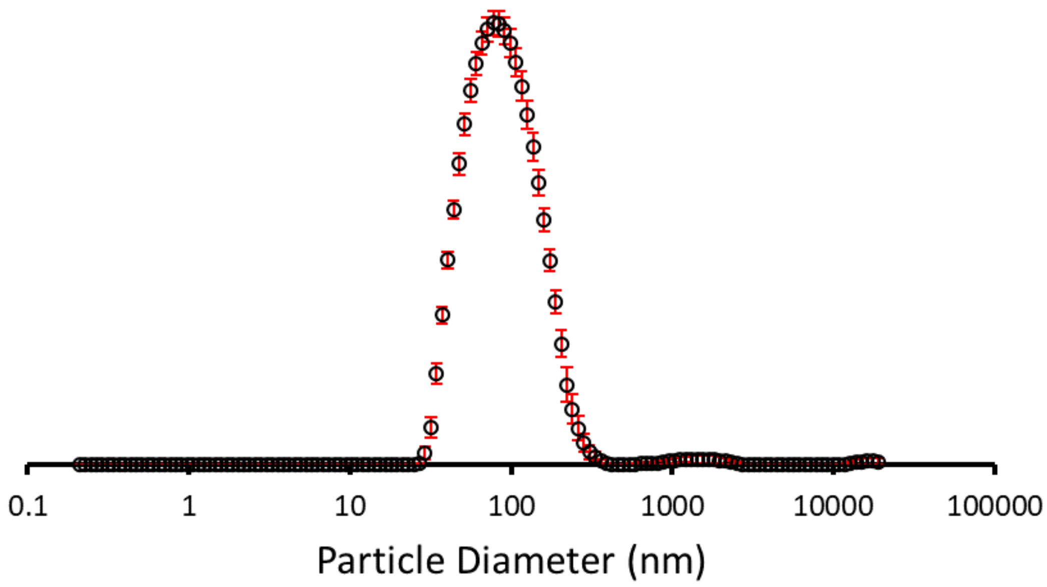

Figure S94 – The average intensity particle size distribution calculated (Peak maxima = 84 nm) using 10 DLS runs for co-formulation **j** (2.78 mM) in an EtOH:H_2O (1:19) solution at 298 K.

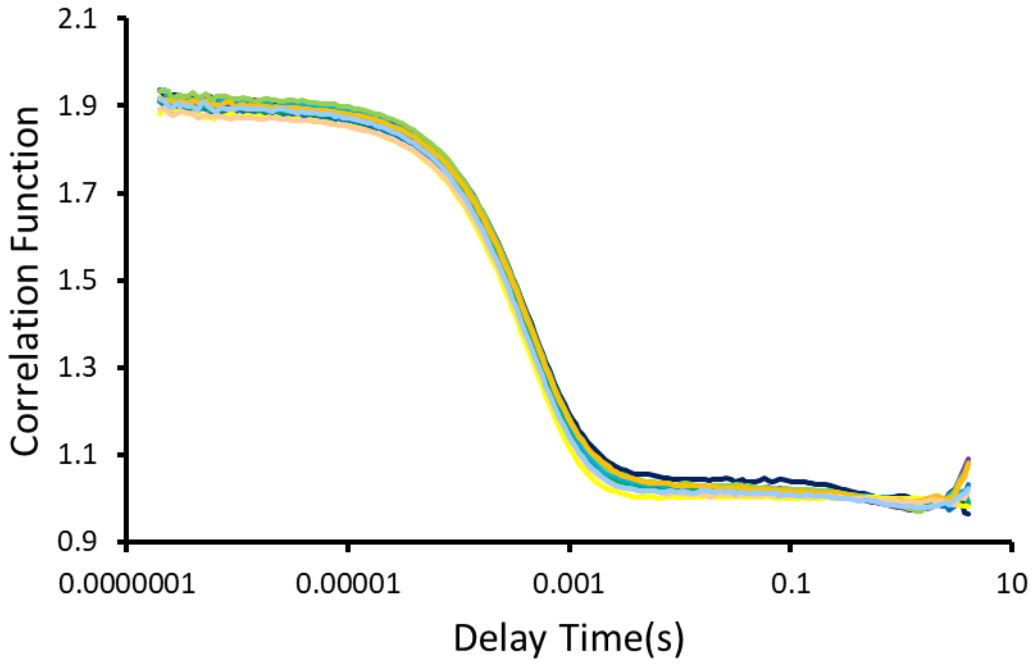

Figure S95 – Correlation function data for 10 DLS runs of co-formulation **j** (2.78 mM) in an EtOH:H_2O (1:19) solution at 298 K.

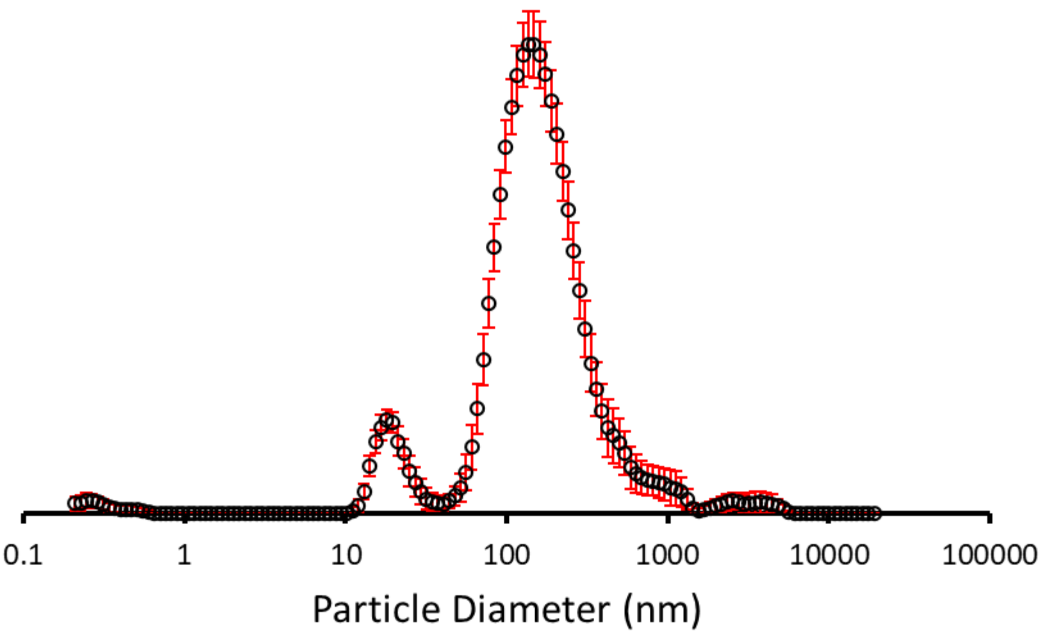

Figure S96 – The average intensity particle size distribution calculated (Peak maxima = 148 nm) using 10 DLS runs for co-formulation **j** (0.28 mM) in an EtOH:H_2O (1:19) solution at 298 K.

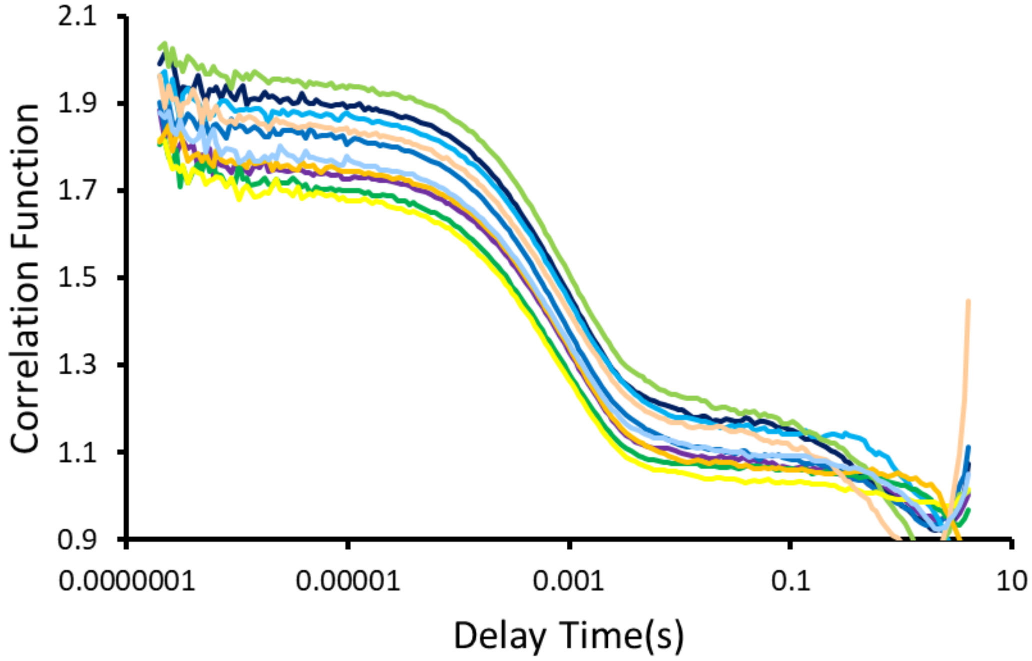

Figure S97 – Correlation function data for 10 DLS runs of co-formulation **j** (0.28 mM) in an EtOH:H_2O (1:19) solution at 298 K.

Overview

Table S10 – Summary of average intensity particle size distribution data determined via DLS in EtOH:H_2O 1:19 solution.

SSA/co-form	Concentration (mM)	Peak 1 (nm)	Peak 2 (nm)	Peak 3 (nm)	Peak 4 (nm)	PDI
**1**	**2.78**	-	184.36	-	-	0.0315
	**0.28**	0.23	100.61	747.99	-	0.0602
**2**	**2.78**	-	142.73	-	-	0.0350
	**0.28**	-	139.08	-	-	0.0160
**3**	**2.78**	51.65	672.27	-	-	0.0658
	**0.28**	0.43	56.01	462.06	-	0.0863
**4**	**2.78**	-	162.46	-	-	0.0388
	**0.28**	-	151/10	-	-	0.0452
**5**	**2.78**	-	160.53		-	0.0614
	**0.28**	-	125.90	747.97	-	0.0547
**a**	**2.78**	0.40	116.10	-	-	0.0745
	**0.28**	0.55	68.50	-	-	0.0767
**b**	**2.78**	-	187.24	-	-	0.0600
	**0.28**	21.19	221.32	-	-	0.0734
**c**	**2.78**	0.65	12.02	107.10	689.79	0.0688
	**0.28**	-	49.00	-	-	0.0217
**d**	**2.78**	8.69	98.74	-	-	0.0485
	**0.28**	-	51.75	-	-	0.0247
**e**	**2.78**	-	188.76	-	-	0.0596
	**0.28**	0.47	15.34	319.27	-	0.0712
**f**	**2.78**	-	181.42	-	-	0.0374
	**0.28**	0.55	13.03	126.10	-	0.0632
**g**	**2.78**	-	105.29	-	-	0.0103
	**0.28**	-	99.72	-	-	0.0245
**h**	**2.78**	-	189.76	-	-	0.0686
	**0.28**	-	83.97	-	-	0.0724
**i**	**2.78**	11.08	391.30	689.79	-	0.0809
	**0.28**	-	51.65	540.99	-	0.0758
**j**	**2.78**	-	83.97	-	-	0.0580
	**0.28**	0.27	19.54	148.04	-	0.0755

Section S10: Zeta potential data

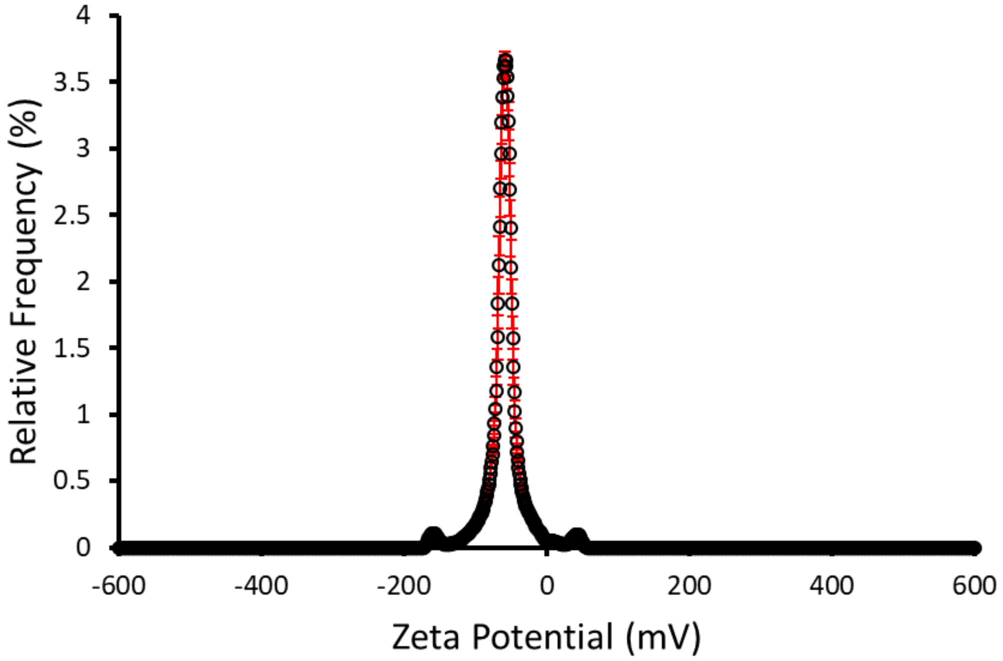

Figure S98 – The average zeta potential distribution calculated using 10 runs for **1** (2.78 mM) in EtOH:H_2O (1:19) solution at 298 K. Average measurement value -55.7 mV.

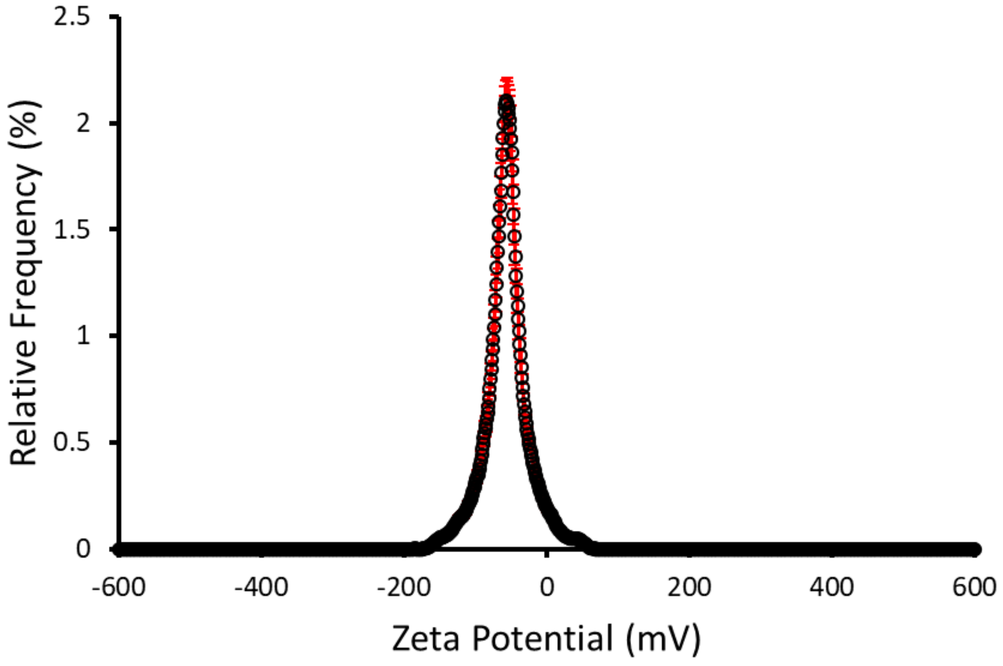

Figure S99 – The average zeta potential distribution calculated using 10 runs for **2** (2.78 mM) in EtOH:H_2O (1:19) solution at 298 K. Average measurement value -55.5 mV.

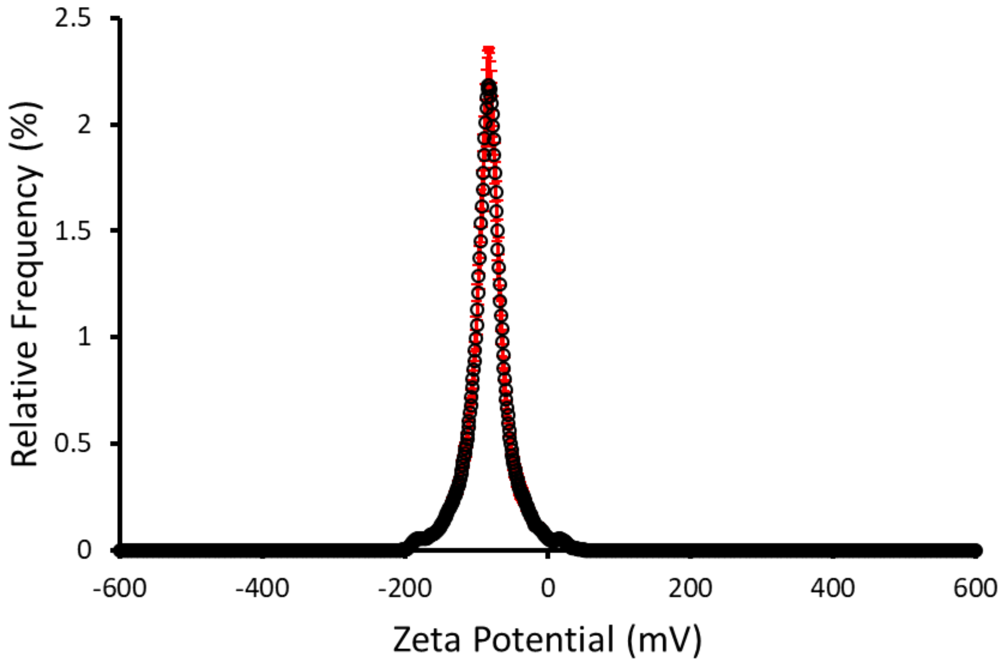

Figure S100 – The average zeta potential distribution calculated using 10 runs for **3** (2.78 mM) in EtOH:H_2O (1:19) solution at 298 K. Average measurement value -86.3 mV.

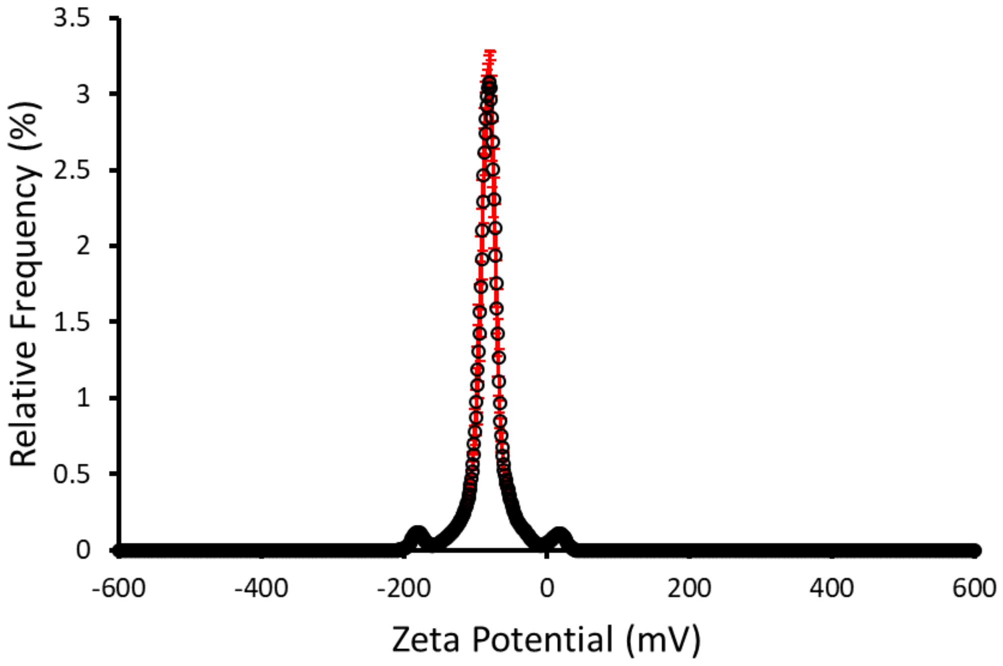

Figure S101 – The average zeta potential distribution calculated using 10 runs for **4** (2.78 mM) in EtOH:H_2O (1:19) solution at 298 K. Average measurement value -79.3 mV.

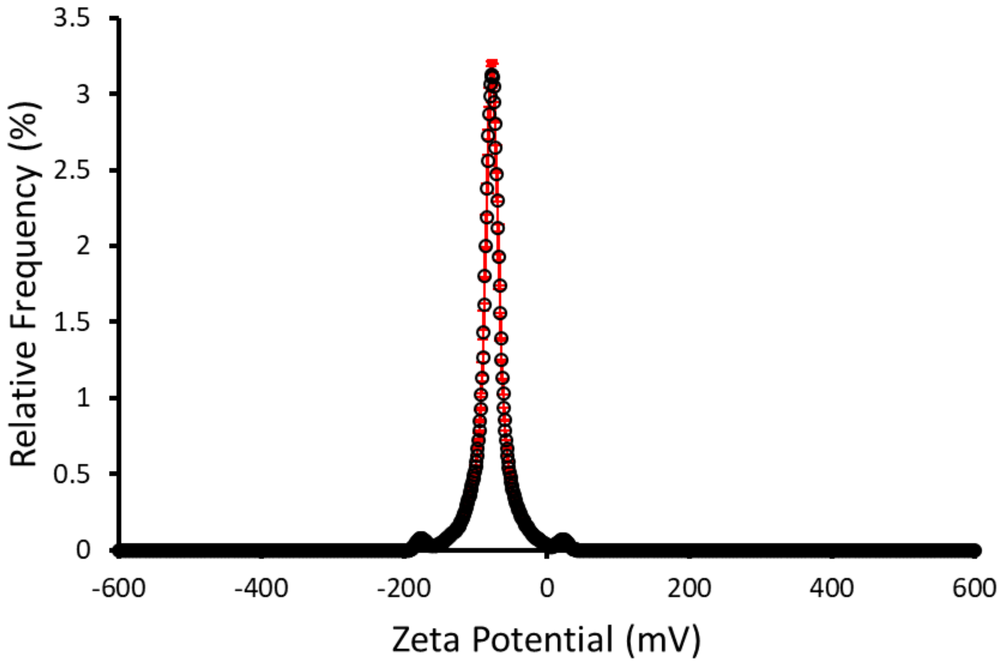

Figure S102 – The average zeta potential distribution calculated using 10 runs for **5** (2.78 mM) in EtOH:H_2O (1:19) solution at 298 K. Average measurement value -73.4 mV.

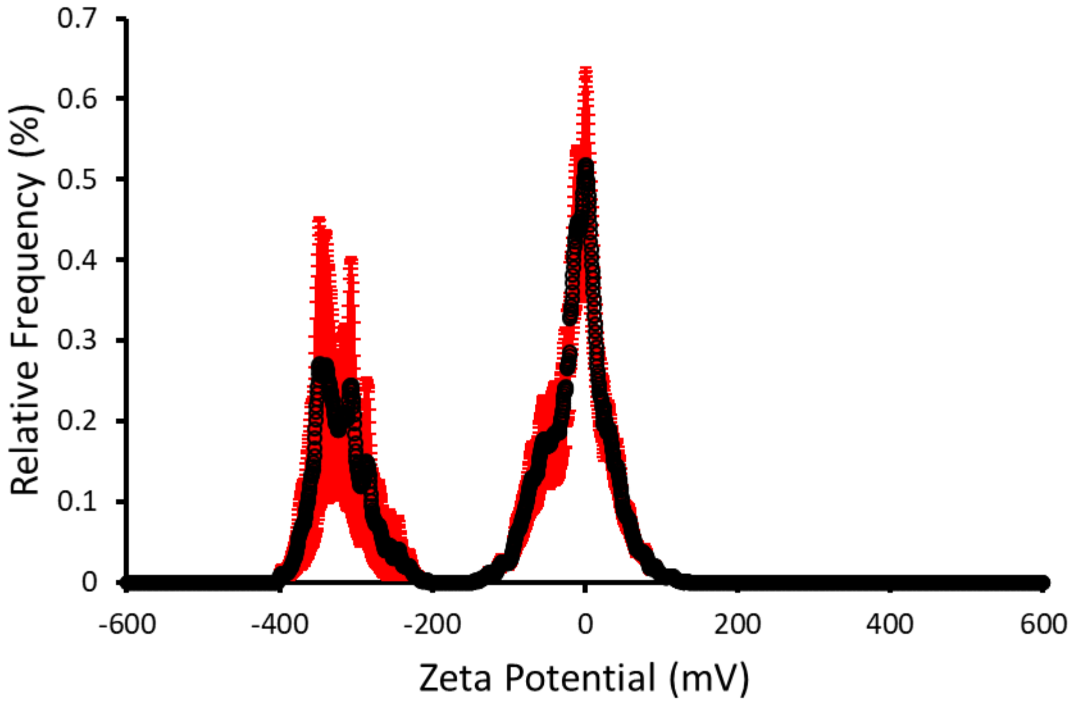

Figure S103 – The average zeta potential distribution calculated using 10 runs for co-formulation **a** (2.78 mM) in EtOH:H_2O (1:19) solution at 298 K. Average measurement value -0.99 mV.

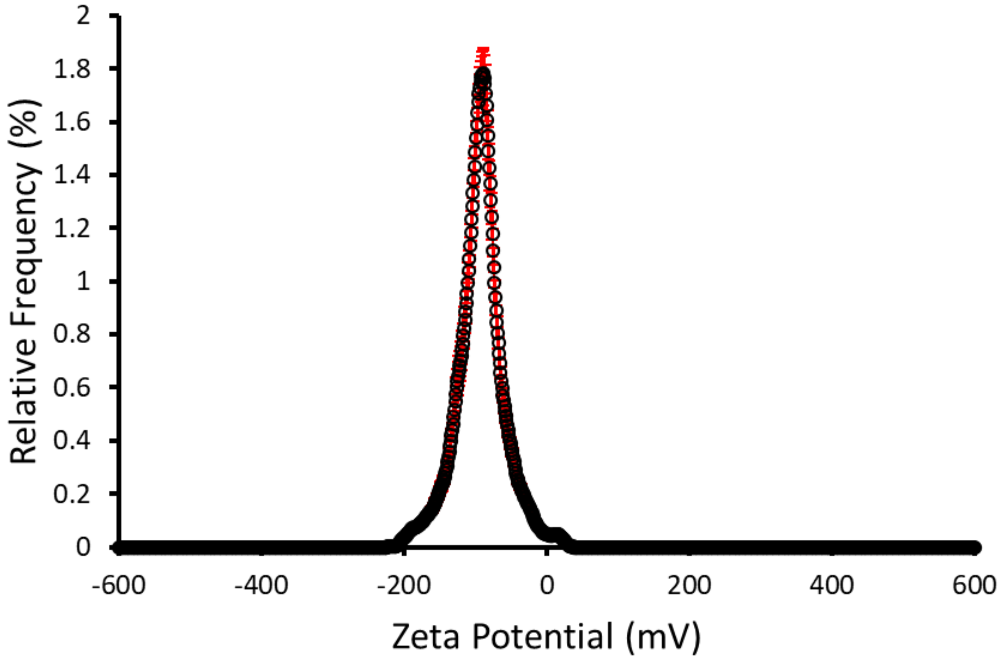

Figure S104 – The average zeta potential distribution calculated using 10 runs for co-formulation **b** (2.78 mM) in EtOH:H_2O (1:19) solution at 298 K. Average measurement value -71.6 mV.

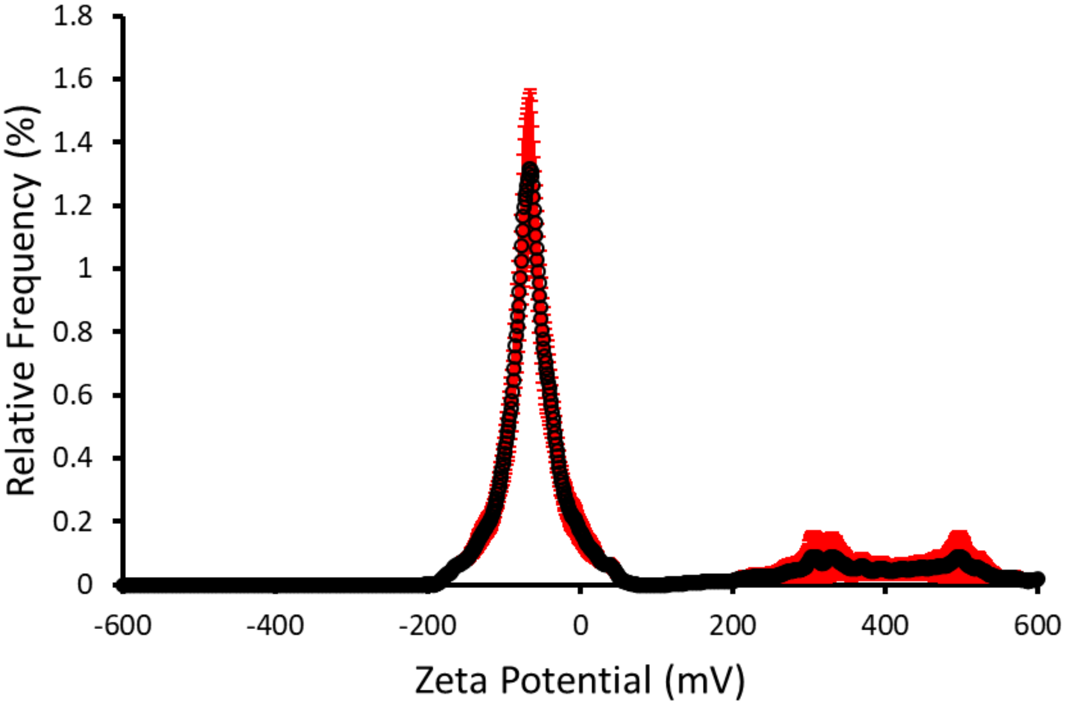

Figure S105 – The average zeta potential distribution calculated using 10 runs for co-formulation **c** (2.78 mM) in EtOH:H_2O (1:19) solution at 298 K. Average measurement value -68.2 mV.

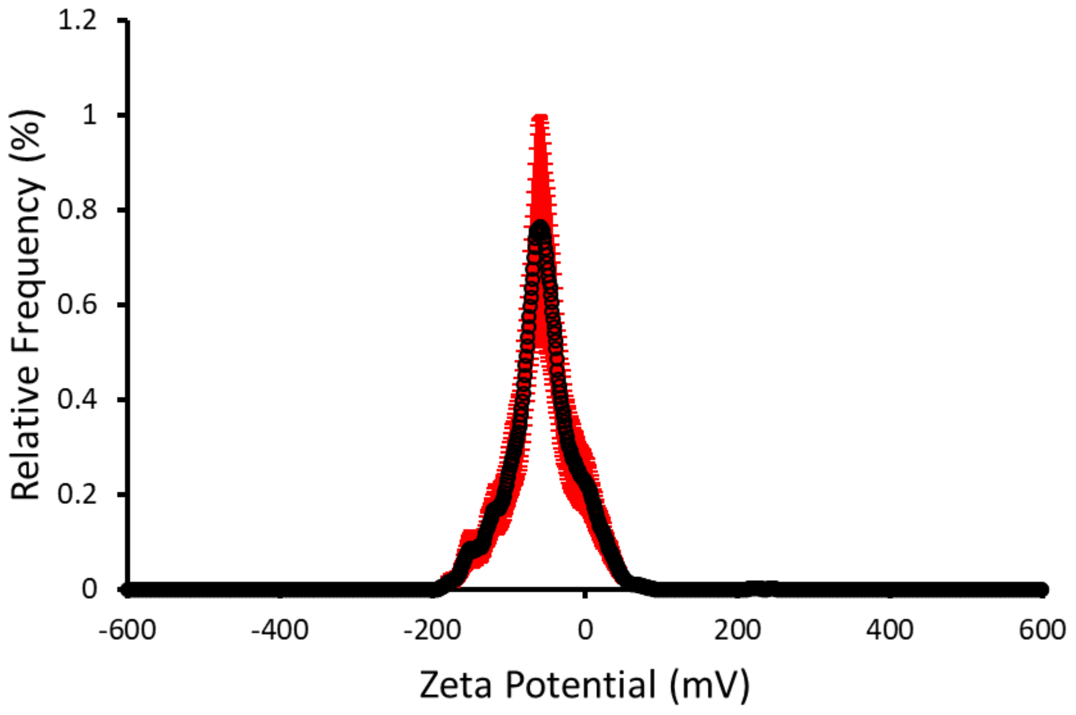

Figure S106 – The average zeta potential distribution calculated using 10 runs for co-formulation **d** (2.78 mM) in EtOH:H_2O (1:19) solution at 298 K. Average measurement value -53.9 mV.

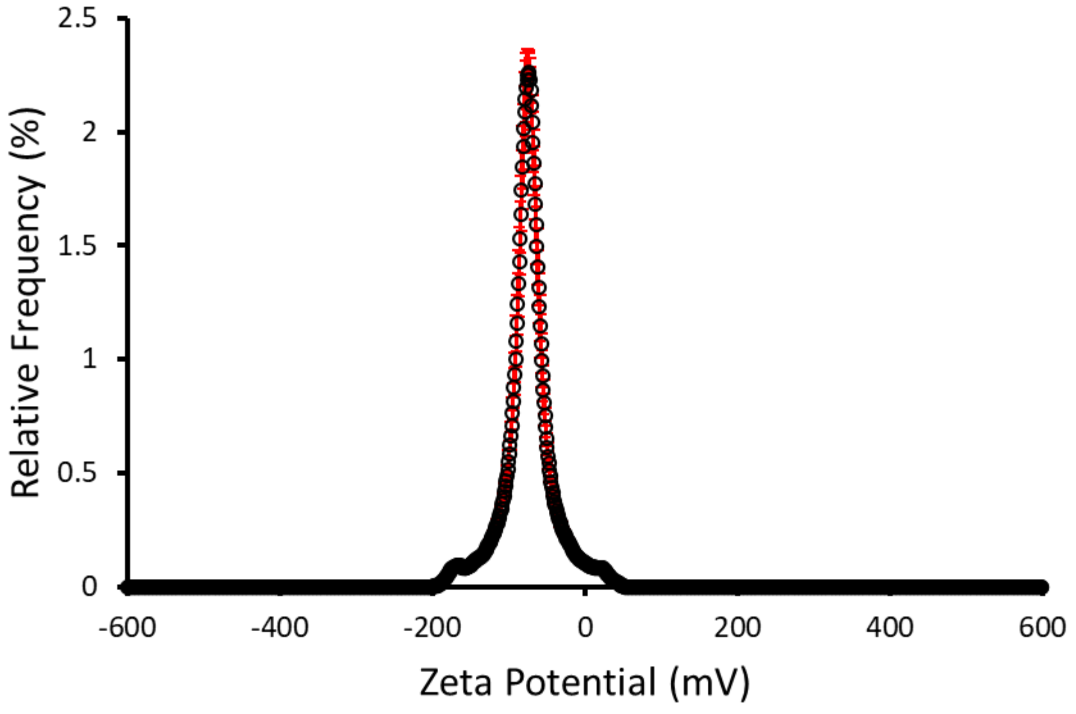

Figure S107 – The average zeta potential distribution calculated using 10 runs for co-formulation **e** (2.78 mM) in EtOH:H_2O (1:19) solution at 298 K. Average measurement value -71.1 mV.

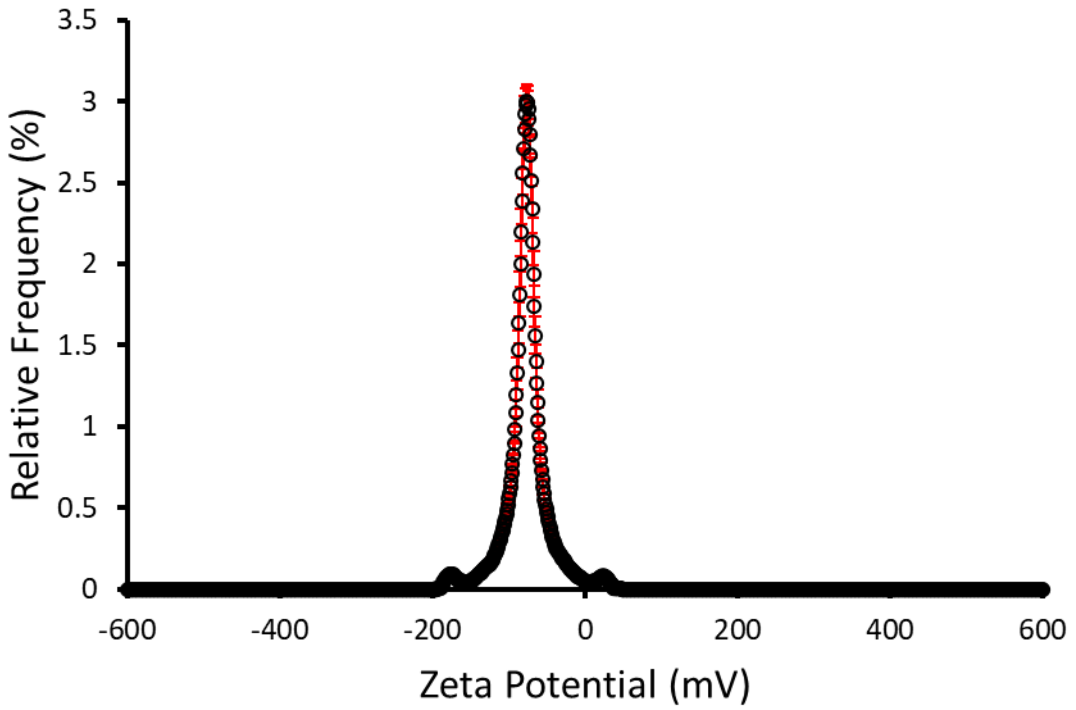

Figure S108 – The average zeta potential distribution calculated using 10 runs for co-formulation **f** (2.78 mM) in EtOH:H_2O (1:19) solution at 298 K. Average measurement value -76.1 mV.

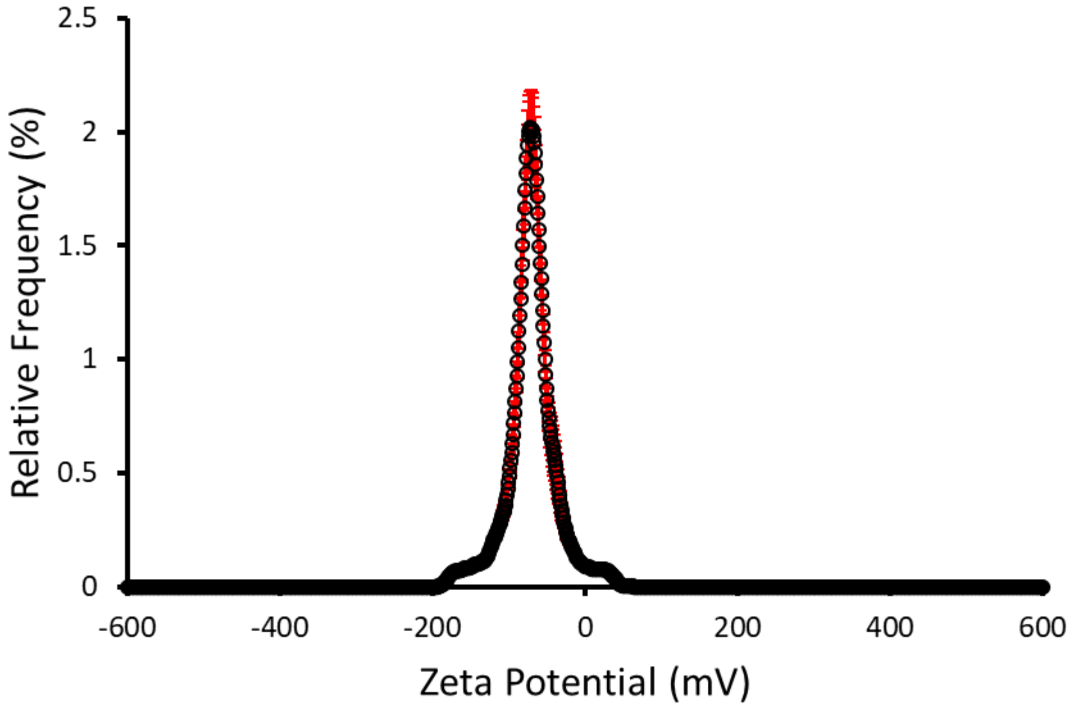

Figure S109 – The average zeta potential distribution calculated using 10 runs for co-formulation **g** (2.78 mM) in EtOH:H_2O (1:19) solution at 298 K. Average measurement value -62.2 mV.

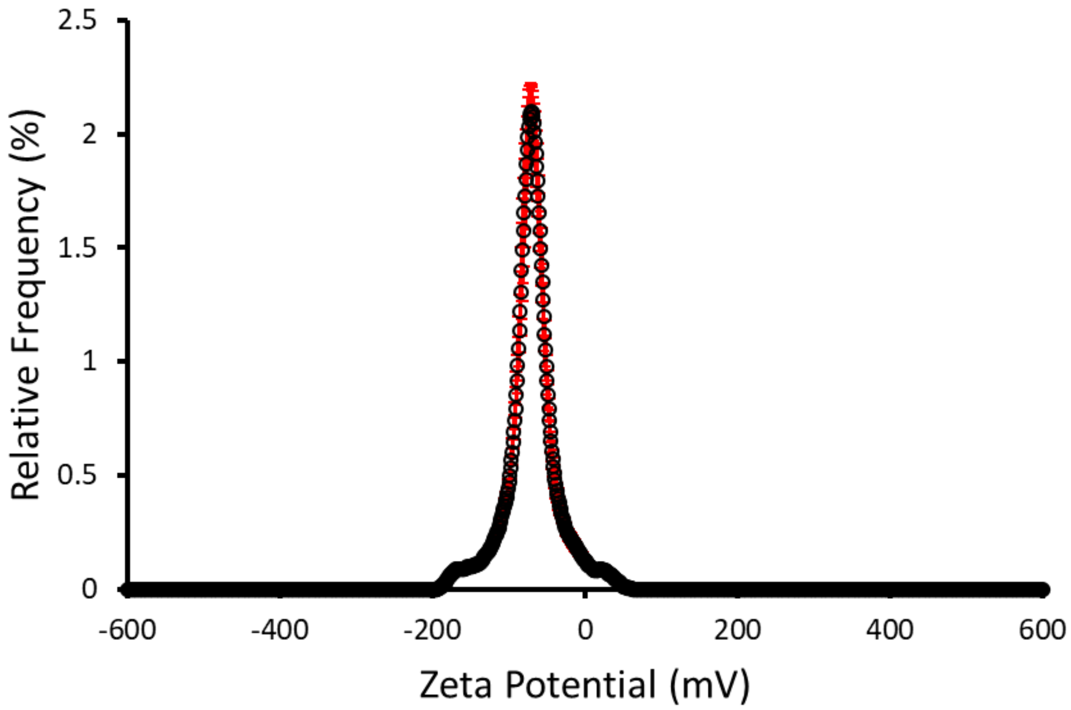

Figure S110 – The average zeta potential distribution calculated using 10 runs for co-formulation **h** (2.78 mM) in EtOH:H_2O (1:19) solution at 298 K. Average measurement value -74.9 mV.

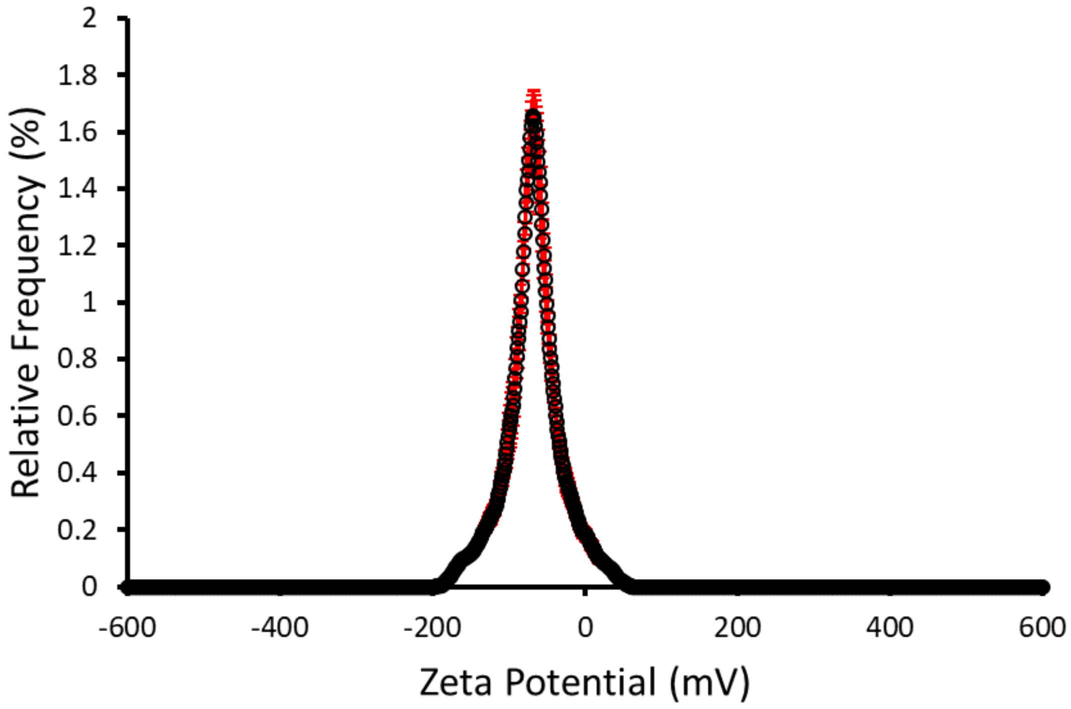

Figure S111 – The average zeta potential distribution calculated using 10 runs for co-formulation **i** (2.78 mM) in EtOH:H_2O (1:19) solution at 298 K. Average measurement value -69.3 mV.

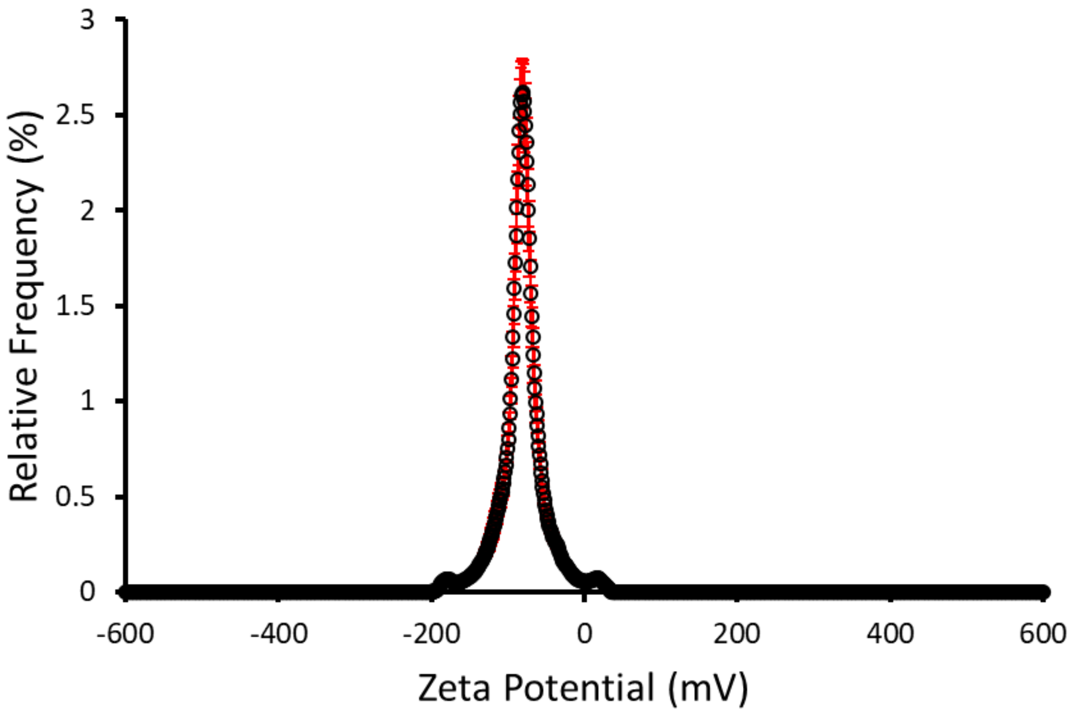

Figure S112 – The average zeta potential distribution calculated using 10 runs for co-formulation **j** (2.78 mM) in EtOH:H_2O (1:19) solution at 298 K. Average measurement value -82.1 mV.

Section S11: Critical aggregation concentration (CAC) determination data

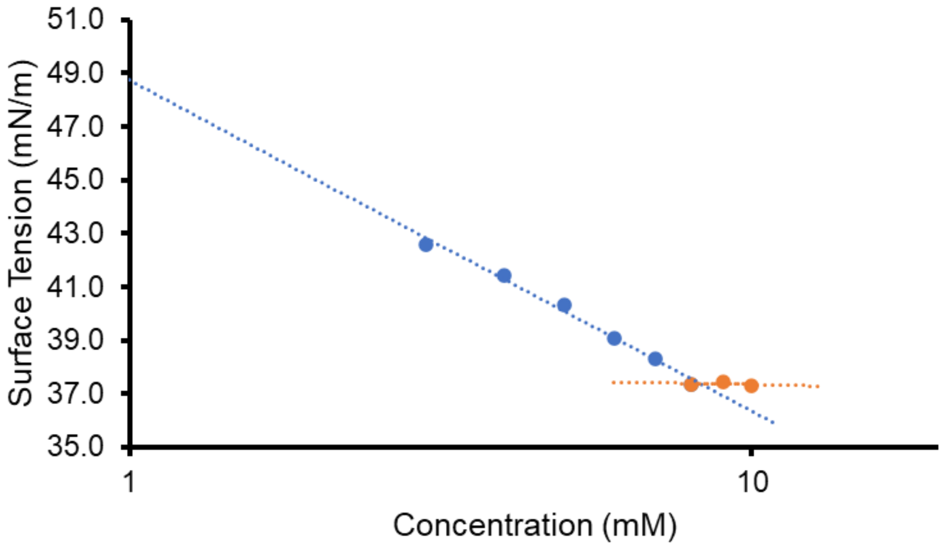

Figure S113 – Calculation of CAC (8.05 mM) for co-formulation **b** in an EtOH:H_2O 1:19 mixture using surface tension measurements.

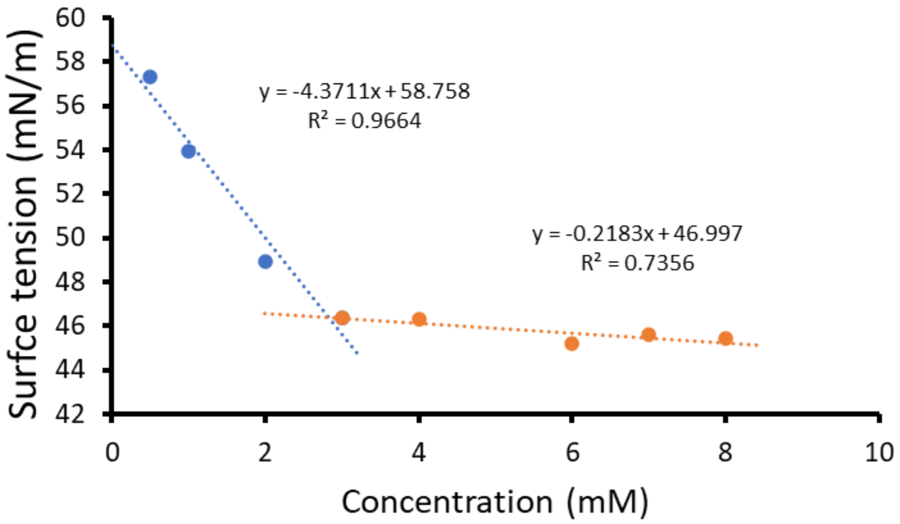

Figure S114 – Calculation of CAC (2.83 mM) for co-formulation **e** in an EtOH:H_2O 1:19 mixture using surface tension measurements.

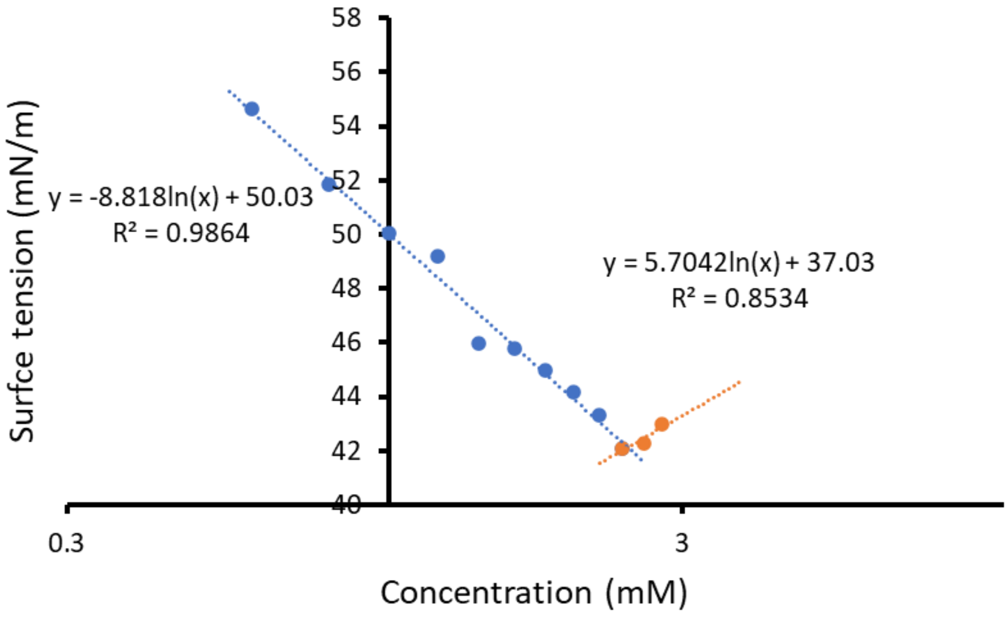

Figure S115 – Calculation of CAC (2.45 mM) for co-formulation **i** in an EtOH:H_2O 1:19 mixture using surface tension measurements.

Section S12: Low level computational modelling data

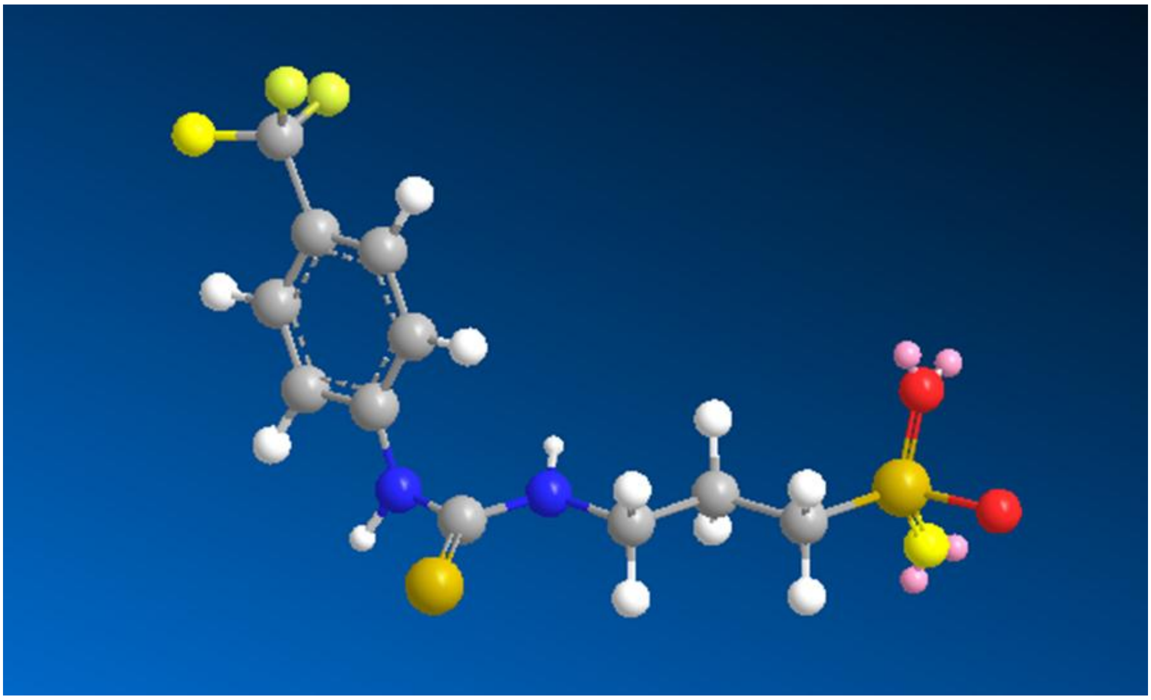

Figure S116 – MM2 model of **1**, with minimised energy. Modelled using Chem3D.

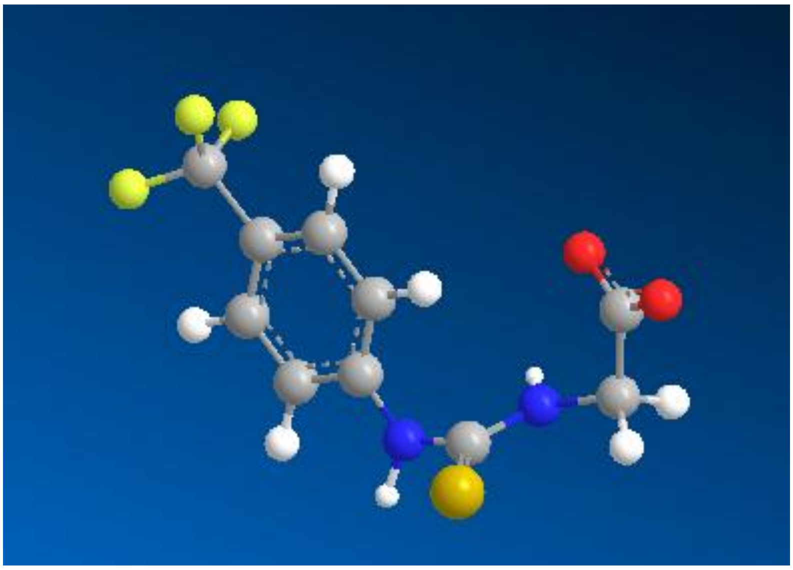

Figure S117 – MM2 model of **2**, with minimised energy. Modelled using Chem3D.

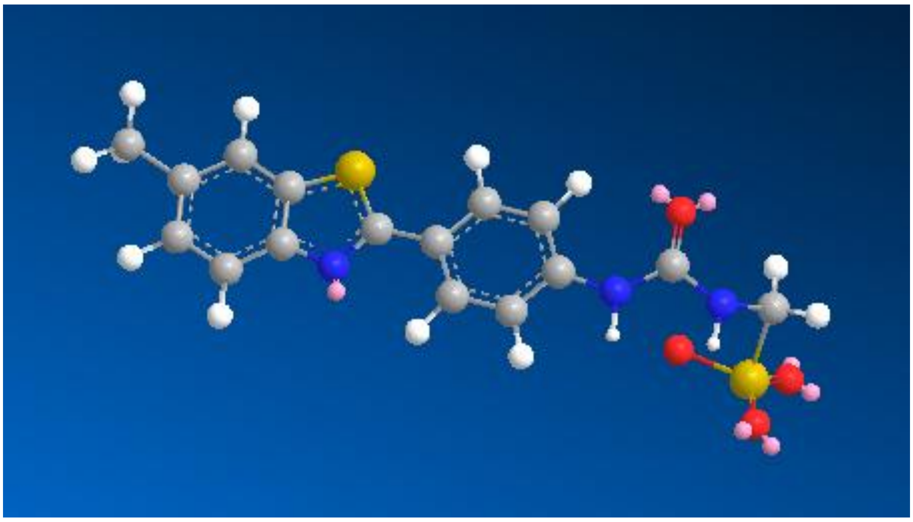

Figure S118 – MM2 model of **3**, with minimised energy. Modelled using Chem3D.

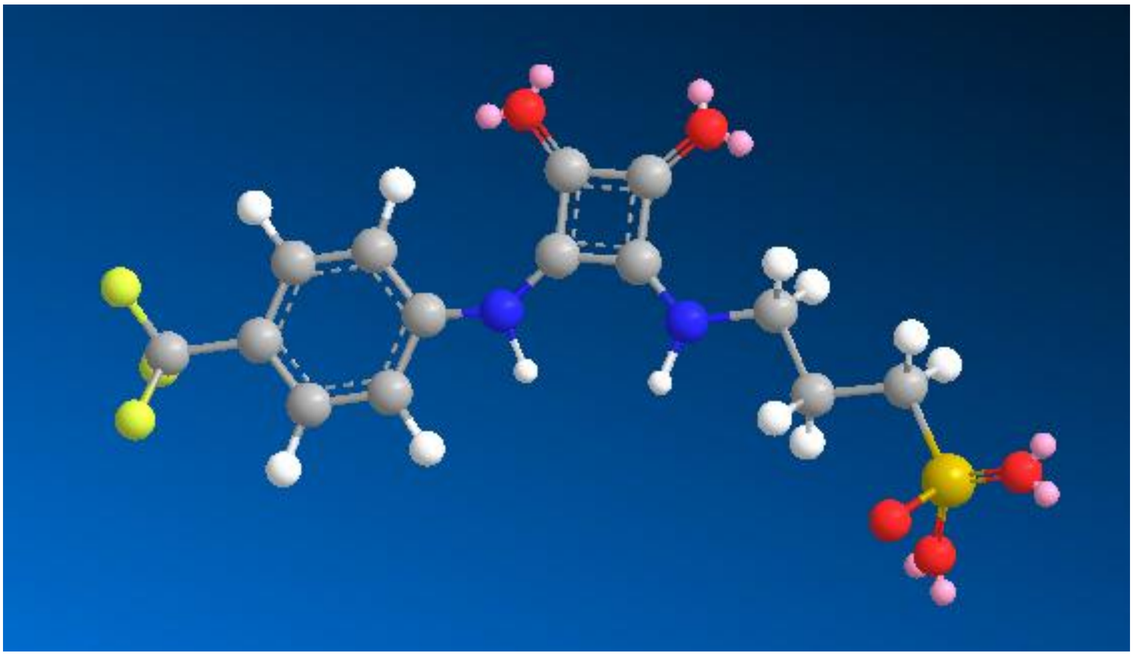

Figure S119 – MM2 model of **4**, with minimised energy. Modelled using Chem3D.

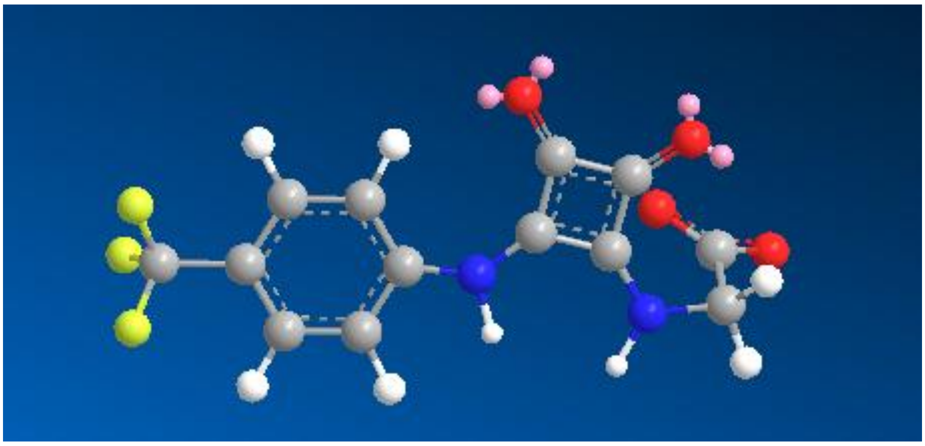

Figure S120 – MM2 model of **5**, with minimised energy. Modelled using Chem3D.

Table S11 – Summary of data generated form MM2 modelling of **1** – **5** using Chem3D.

	Length (nm)	Dipole/dipole energy (C.m)	Total energy (kcal/mol)
**1**	1.321	8.000	184.184
**2**	0.945	-0.610	-4.652
**3**	1.630	-0.176	167.357
**4**	1.536	28.654	263.624
**5**	1.184	18.395	77.704

Section S13: Vesicle leakage assay data

PG lipid titrations

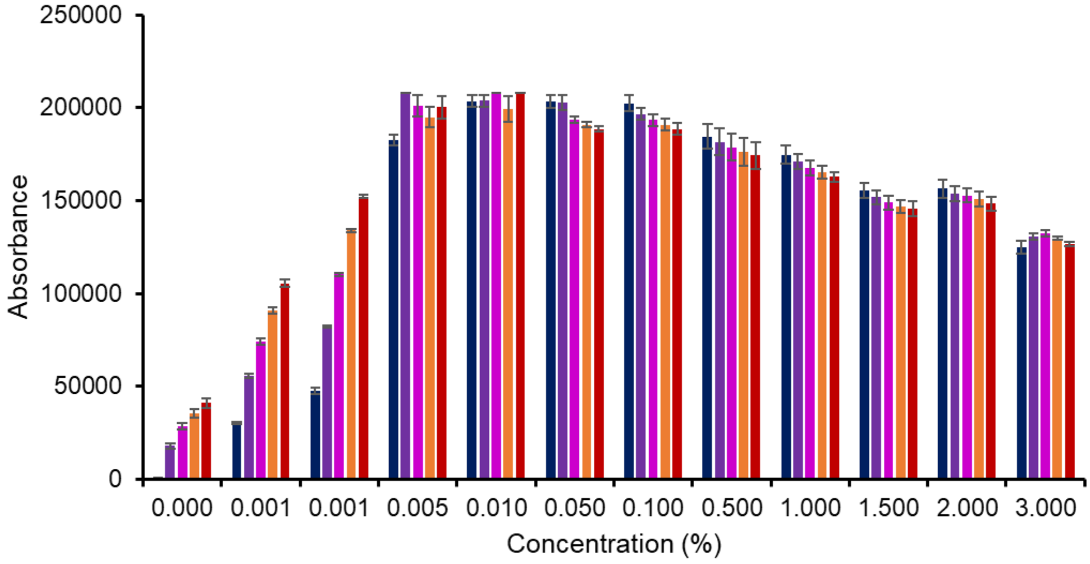

Figure S121 – Average (n = 3) absorbance readings from calcein loaded PG lipid vesicles (λ_{em} = 515 nm) following the addition of triton at varying concentrations. Blue = 1 minute, purple = 5 minutes, pink = 10 minutes, orange = 15 minutes, red = 20 minutes. Error = standard deviation of the mean.

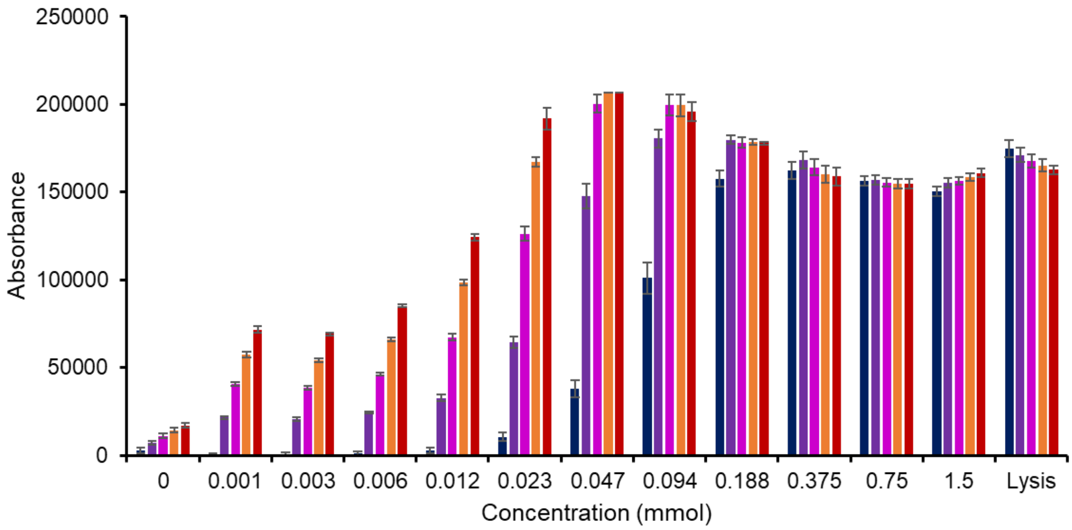

Figure S122 – Average (n = 3) absorbance readings from calcein loaded PG lipid vesicles (λ_{em} = 515 nm) following the addition of **1** at varying concentrations. Lysis value is triton (1 %). Blue = 1 minute, purple = 5 minutes, pink = 10 minutes, orange = 15 minutes, red = 20 minutes. Error = standard deviation of the mean.

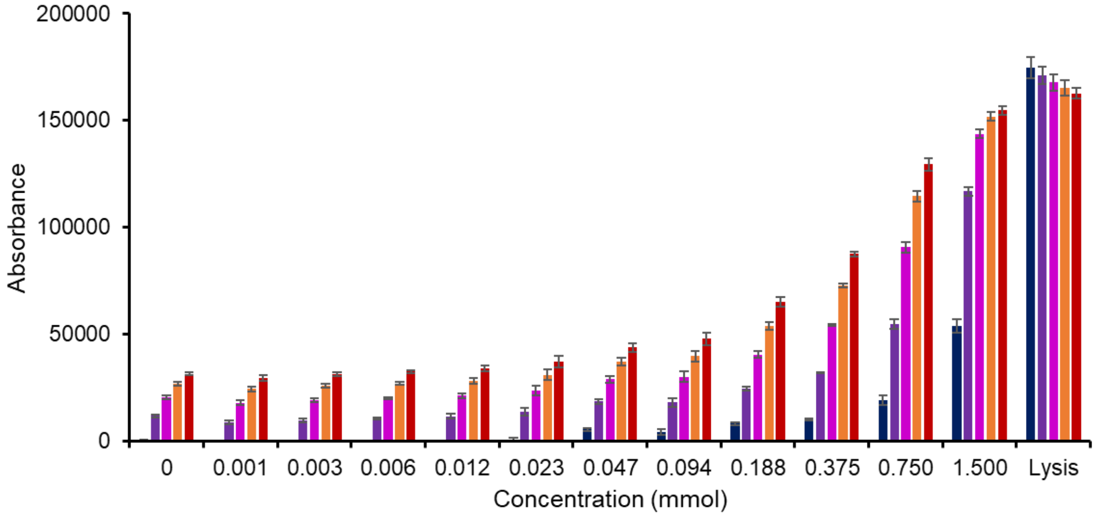

Figure S123 – Average (n = 3) absorbance readings from calcein loaded PG lipid vesicles (λ_{em} = 515 nm) following the addition of **2** at varying concentrations. Lysis value is triton (1 %). Blue = 1 minute, purple = 5 minutes, pink = 10 minutes, orange = 15 minutes, red = 20 minutes. Error = standard deviation of the mean.

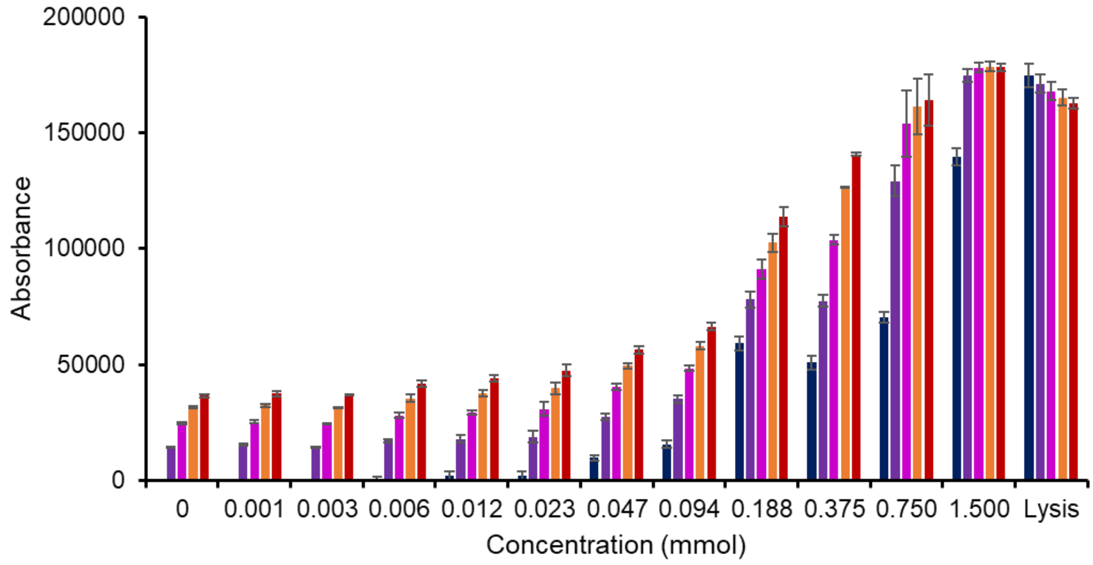

Figure S124 – Average (n = 3) absorbance readings from calcein loaded PG lipid vesicles (λ_{em} = 515 nm) following the addition of **3** at varying concentrations. Lysis value is triton (1 %). triton. Blue = 1 minute, purple = 5 minutes, pink = 10 minutes, orange = 15 minutes, red = 20 minutes. Error = standard deviation of the mean.

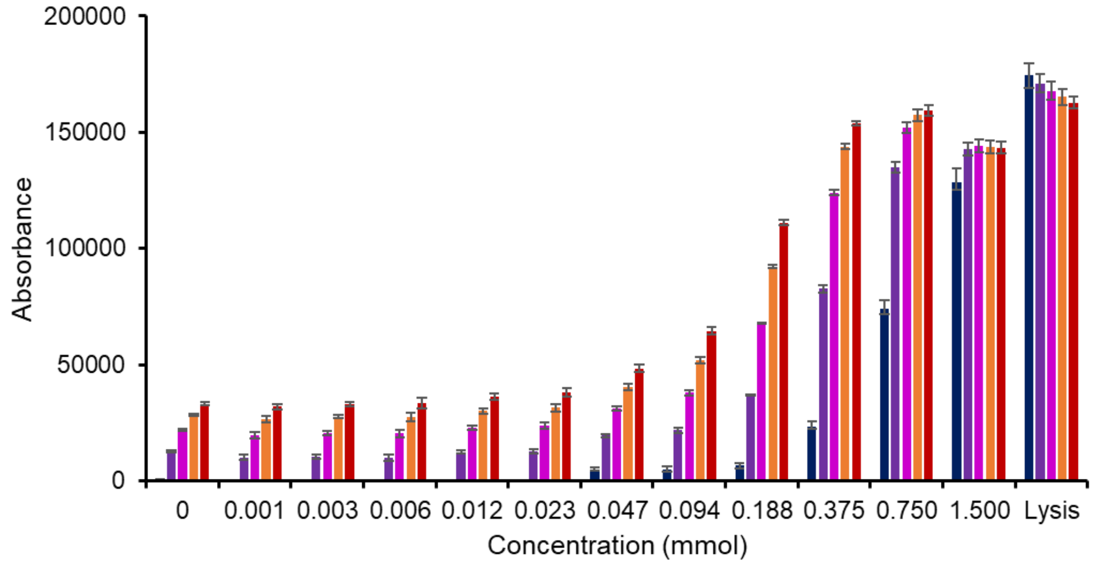

Figure S125 – Average (n = 3) absorbance readings from calcein loaded PG lipid vesicles (λ_{em} = 515 nm) following the addition of **4** at varying concentrations. Lysis value is triton (1 %). Blue = 1 minute, purple = 5 minutes, pink = 10 minutes, orange = 15 minutes, red = 20 minutes. Error = standard deviation of the mean.

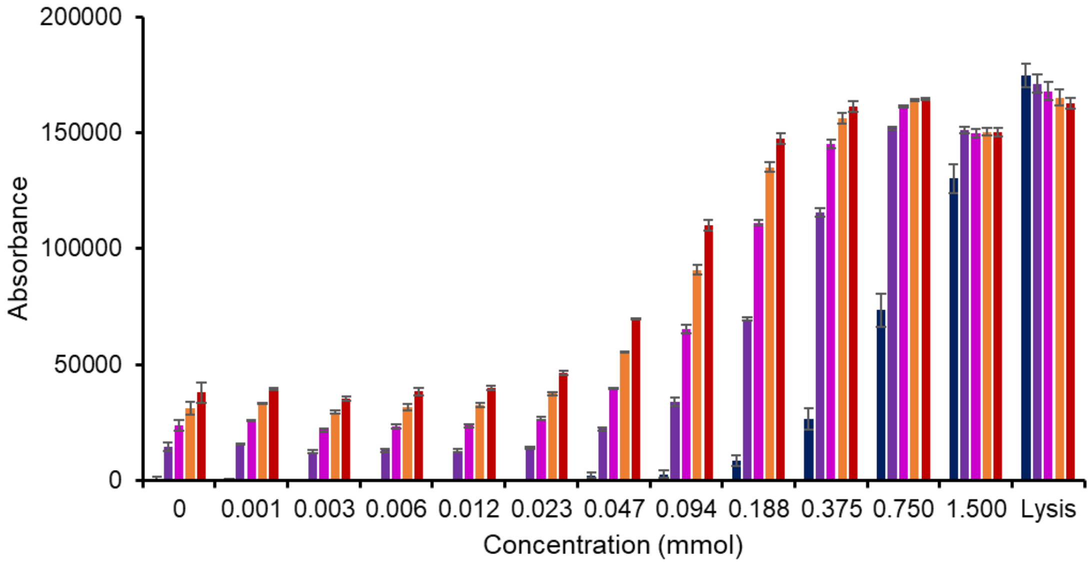

Figure S126 – Average (n = 3) absorbance readings from calcein loaded PG lipid vesicles (λ_{em} = 515 nm) following the addition of **5** at varying concentrations. Lysis value is triton (1 %). Blue = 1 minute, purple = 5 minutes, pink = 10 minutes, orange = 15 minutes, red = 20 minutes. Error = standard deviation of the mean.

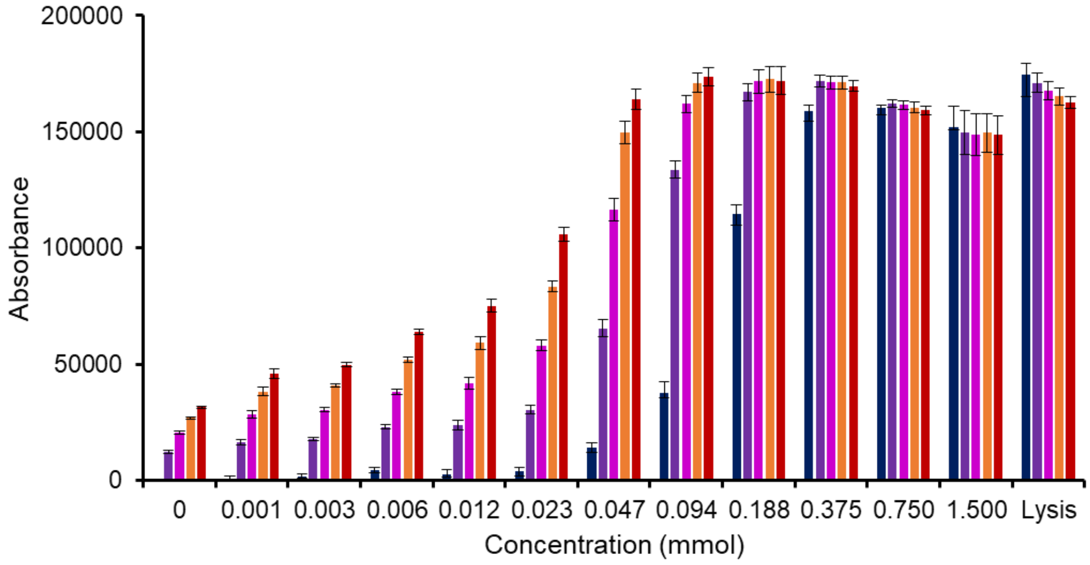

Figure S127 – Average (n = 3) absorbance readings from calcein loaded PG lipid vesicles (λ_{em} = 515 nm) following the addition of **a** at varying concentrations. Lysis value is triton (1 %). Blue = 1 minute, purple = 5 minutes, pink = 10 minutes, orange = 15 minutes, red = 20 minutes. Error = standard deviation of the mean.

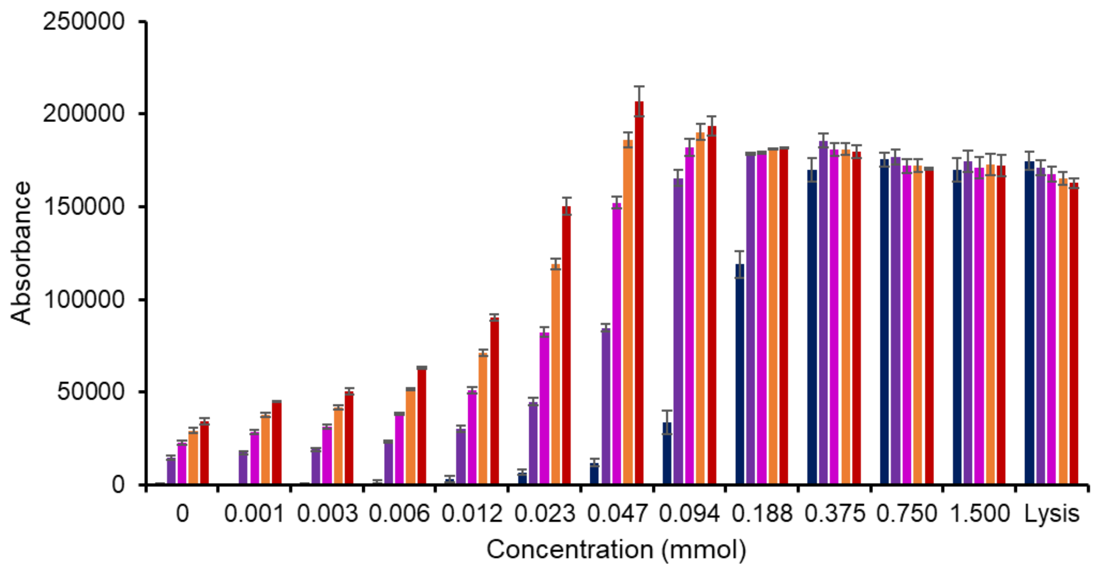

Figure S128 – Average (n = 3) absorbance readings from calcein loaded PG lipid vesicles (λ_{em} = 515 nm) following the addition of **b** at varying concentrations. Lysis value is triton (1 %). Blue = 1 minute, purple = 5 minutes, pink = 10 minutes, orange = 15 minutes, red = 20 minutes. Error = standard deviation of the mean.

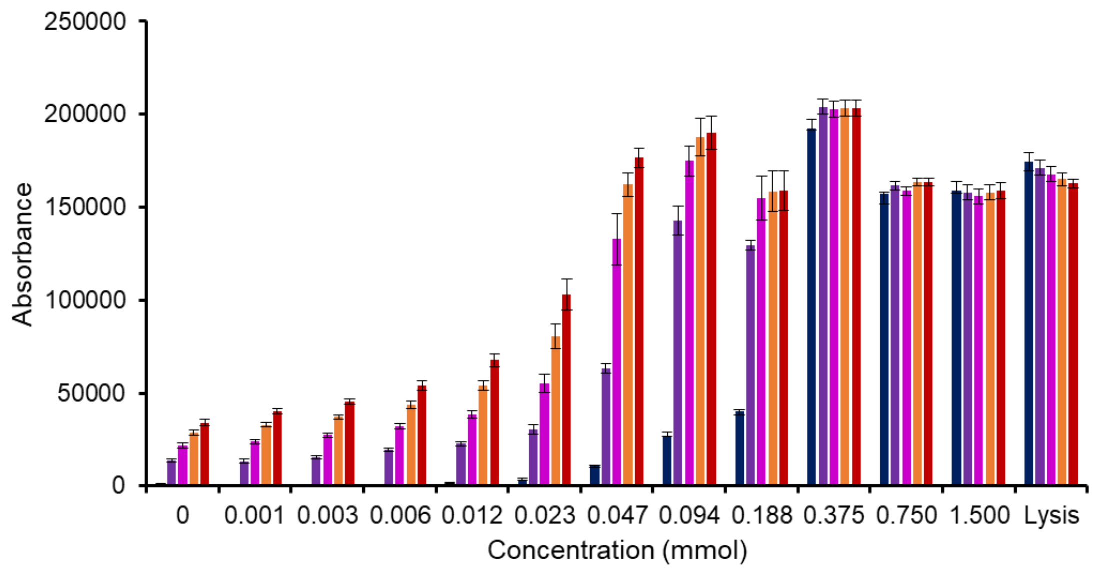

Figure S129 – Average (n = 3) absorbance readings from calcein loaded PG lipid vesicles (λ_{em} = 515 nm) following the addition of **c** at varying concentrations. Lysis value is triton (1 %). Blue = 1 minute, purple = 5 minutes, pink = 10 minutes, orange = 15 minutes, red = 20 minutes. Error = standard deviation of the mean.

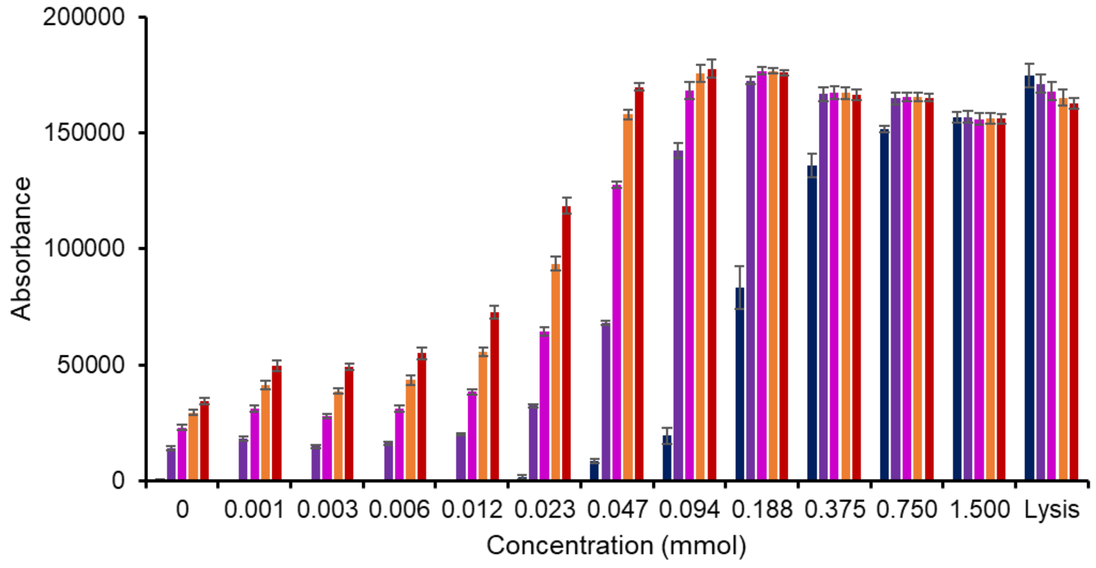

Figure S130 – Average (n = 3) absorbance readings from calcein loaded PG lipid vesicles (λ_{em} = 515 nm) following the addition of **d** at varying concentrations. Lysis value is triton (1 %). Blue = 1 minute, purple = 5 minutes, pink = 10 minutes, orange = 15 minutes, red = 20 minutes. Error = standard deviation of the mean.

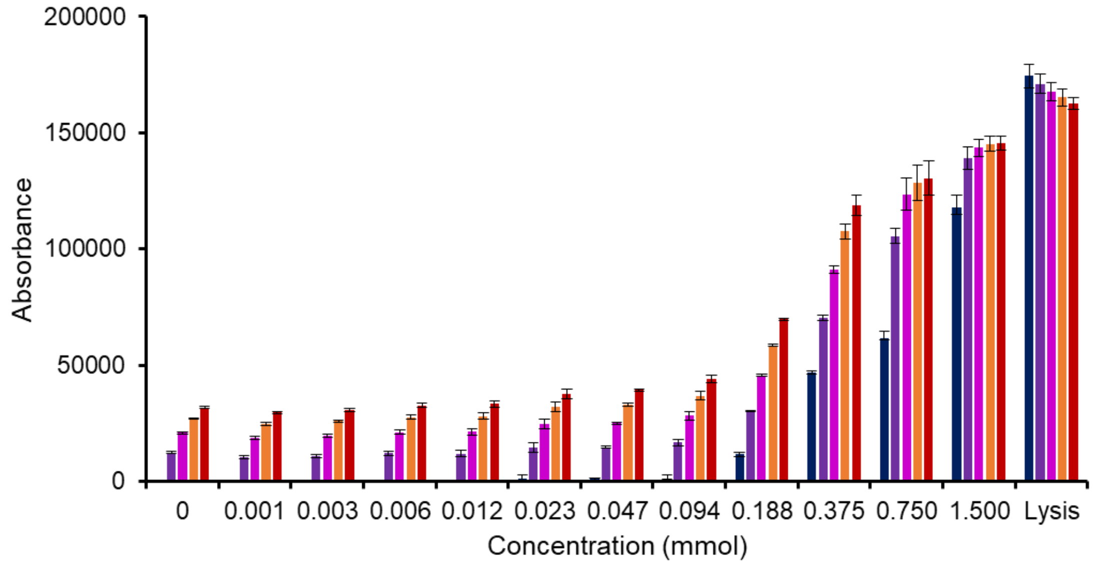

Figure S131 – Average (n = 3) absorbance readings from calcein loaded PG lipid vesicles (λ_{em} = 515 nm) following the addition of **e** at varying concentrations. Lysis value is triton (1 %). Blue = 1 minute, purple = 5 minutes, pink = 10 minutes, orange = 15 minutes, red = 20 minutes. Error = standard deviation of the mean.

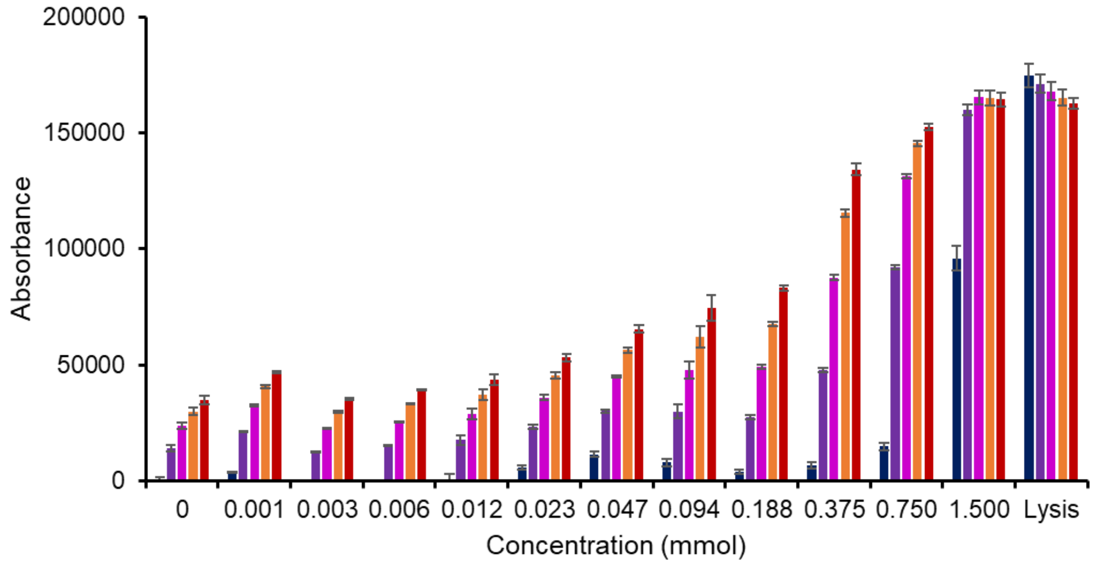

Figure S132 – Average (n = 3) absorbance readings from calcein loaded PG lipid vesicles (λ_{em} = 515 nm) following the addition of **f** at varying concentrations. Lysis value is triton (1 %). Blue = 1 minute, purple = 5 minutes, pink = 10 minutes, orange = 15 minutes, red = 20 minutes. Error = standard deviation of the mean.

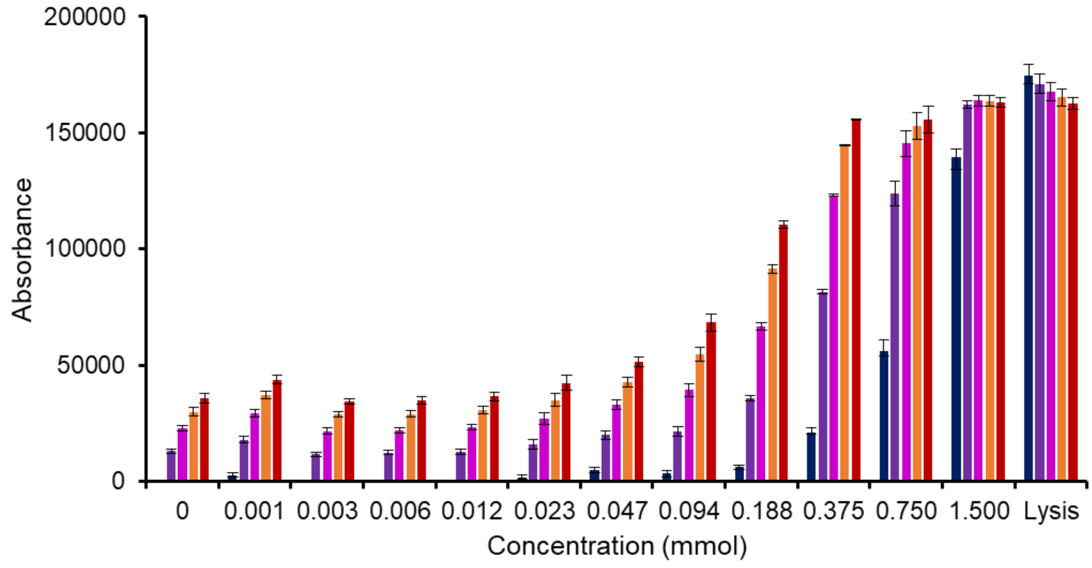

Figure S133 – Average (n = 3) absorbance readings from calcein loaded PG lipid vesicles (λ_{em} = 515 nm) following the addition of **g** at varying concentrations. Lysis value is triton (1 %). Blue = 1 minute, purple = 5 minutes, pink = 10 minutes, orange = 15 minutes, red = 20 minutes. Error = standard deviation of the mean.

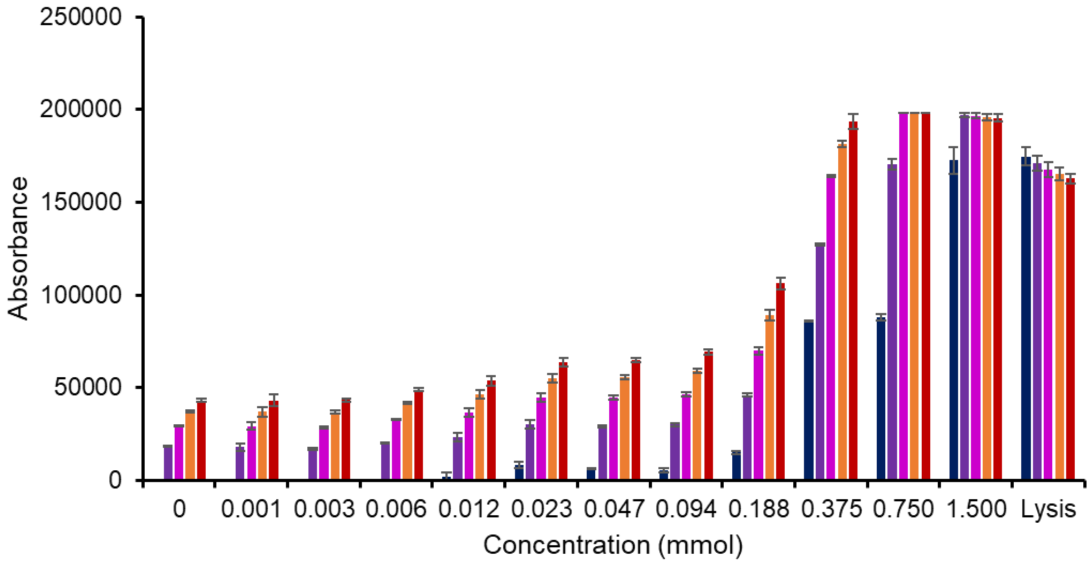

Figure S134 – Average (n = 3) absorbance readings from calcein loaded PG lipid vesicles (λ_{em} = 515 nm) following the addition of **h** at varying concentrations. Lysis value is triton (1 %). Blue = 1 minute, purple = 5 minutes, pink = 10 minutes, orange = 15 minutes, red = 20 minutes. Error = standard deviation of the mean.

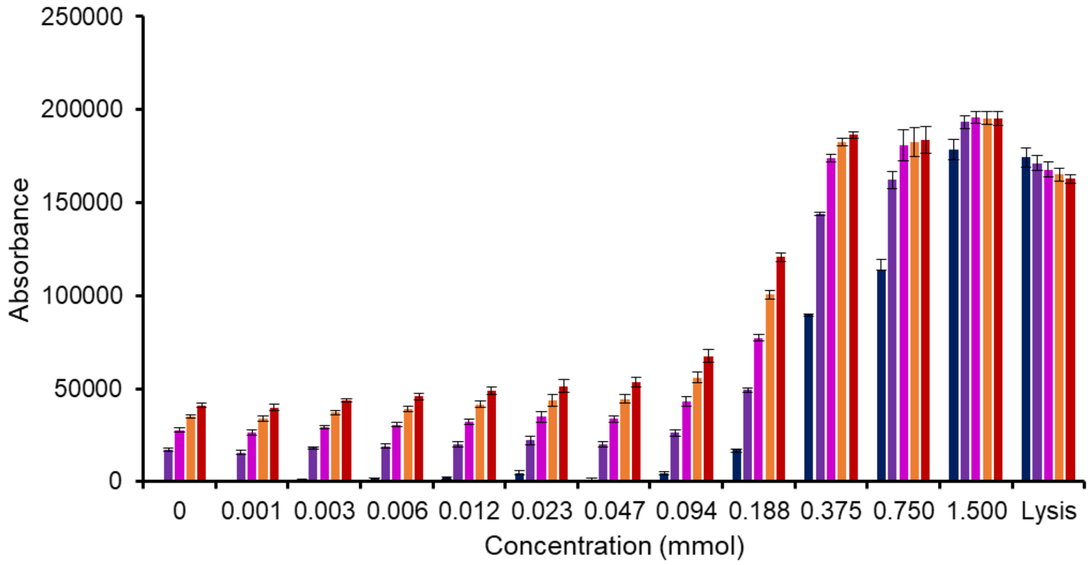

Figure S135 – Average (n = 3) absorbance readings from calcein loaded PG lipid vesicles (λ_{em} = 515 nm) following the addition of **i** at varying concentrations. Lysis value is triton (1 %). Blue = 1 minute, purple = 5 minutes, pink = 10 minutes, orange = 15 minutes, red = 20 minutes. Error = standard deviation of the mean.

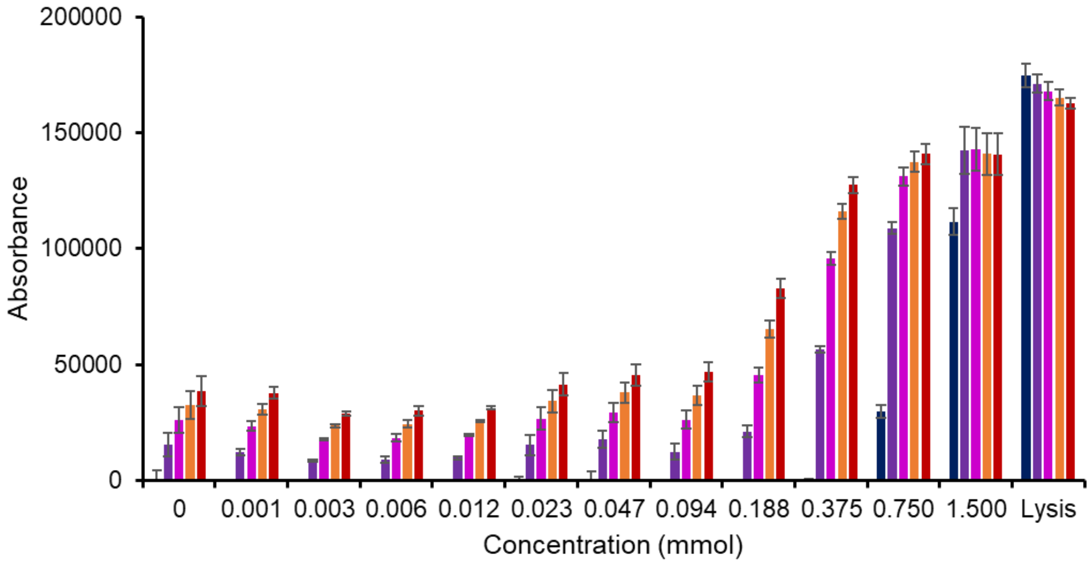

Figure S136 – Average (n = 3) absorbance readings from calcein loaded PG lipid vesicles (λ_{em} = 515 nm) following the addition of **j** at varying concentrations. Lysis value is triton (1 %). Blue = 1 minute, purple = 5 minutes, pink = 10 minutes, orange = 15 minutes, red = 20 minutes. Error = standard deviation of the mean.

PE:PG 3:1 lipid titrations

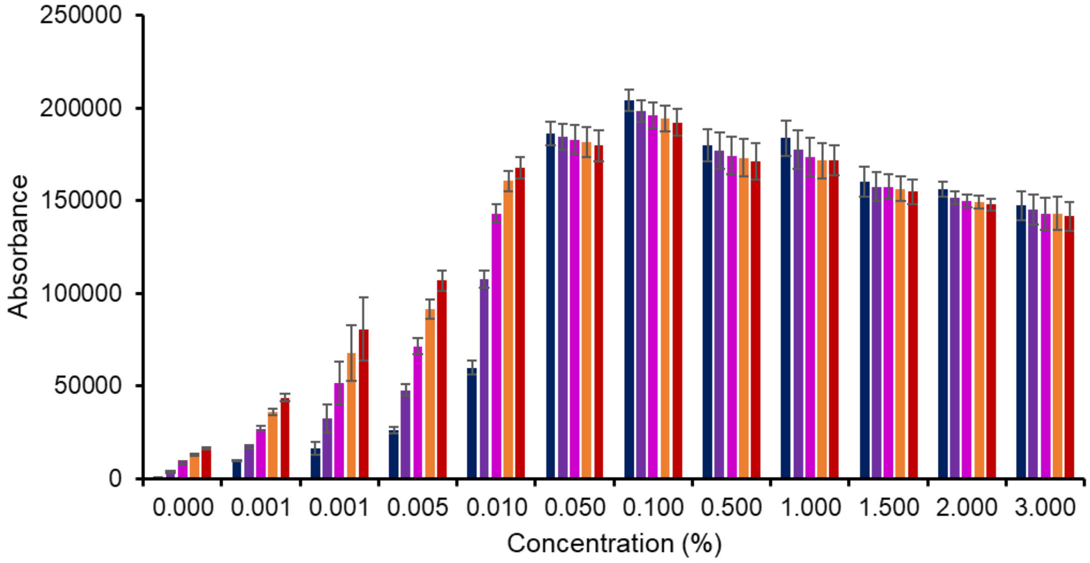

Figure S137 – Average (n = 3) absorbance readings from calcein loaded PE:PG 3:1 lipid vesicles (λ_{em} = 515 nm) following the addition of triton at varying concentrations. Blue = 1 minute, purple = 5 minutes, pink = 10 minutes, orange = 15 minutes, red = 20 minutes. Error = standard deviation of the mean.

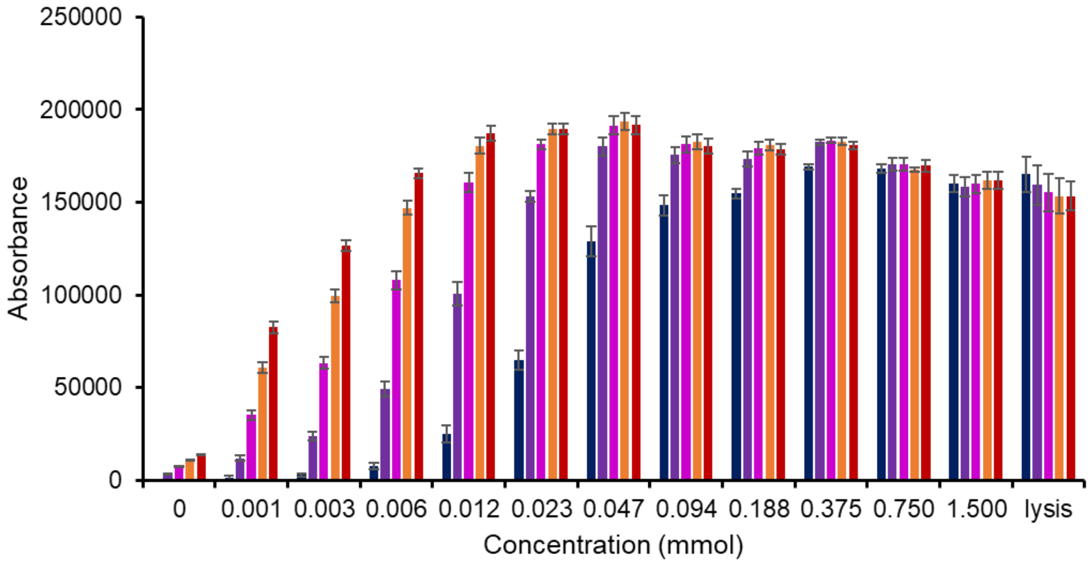

Figure S138 – Average (n = 3) absorbance readings from calcein loaded PE:PG 3:1 lipid vesicles (λ_{em} = 515 nm) following the addition of **1** at varying concentrations. Lysis value is triton (1 %). Blue = 1 minute, purple = 5 minutes, pink = 10 minutes, orange = 15 minutes, red = 20 minutes. Error = standard deviation of the mean.

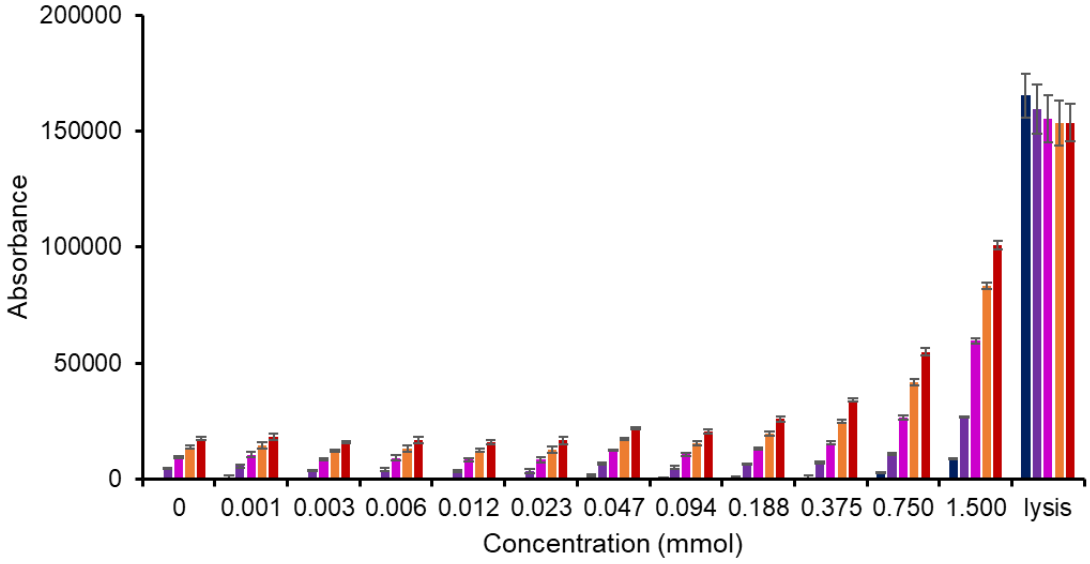

Figure S139 – Average (n = 3) absorbance readings from calcein loaded PE:PG 3:1 lipid vesicles (λ_{em} = 515 nm) following the addition of **2** at varying concentrations. Lysis value is triton (1 %). Blue = 1 minute, purple = 5 minutes, pink = 10 minutes, orange = 15 minutes, red = 20 minutes. Error = standard deviation of the mean.

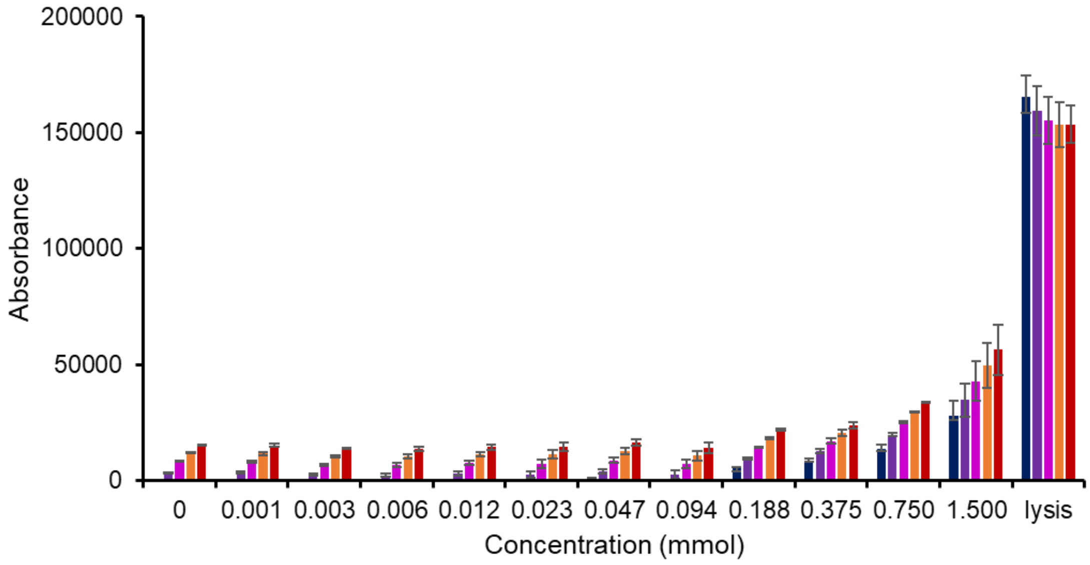

Figure S140 – Average (n = 3) absorbance readings from calcein loaded PE:PG 3:1 lipid vesicles (λ_{em} = 515 nm) following the addition of **3** at varying concentrations. Lysis value is triton (1 %). Blue = 1 minute, purple = 5 minutes, pink = 10 minutes, orange = 15 minutes, red = 20 minutes. Error = standard deviation of the mean.

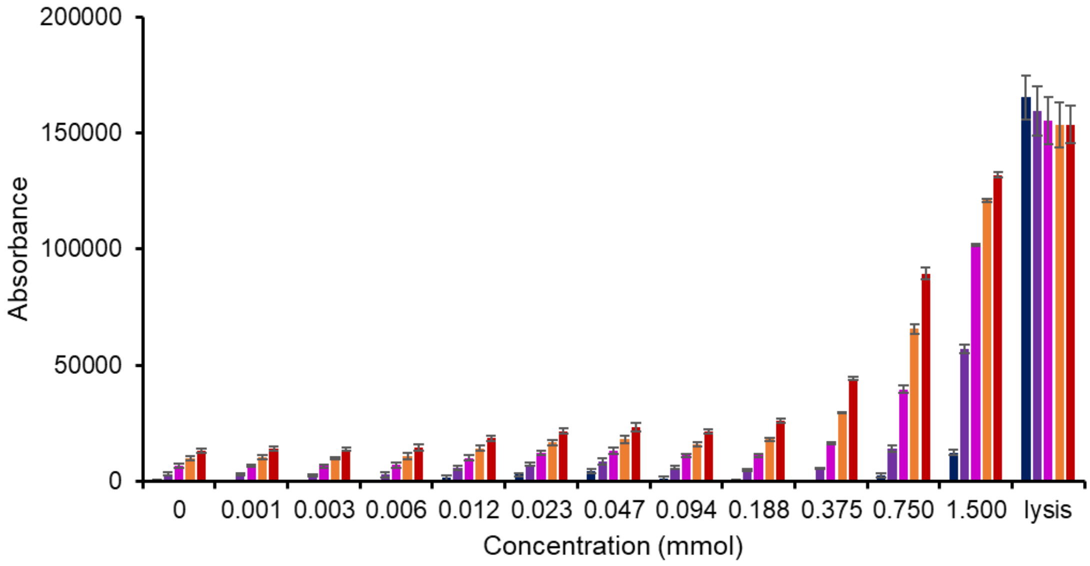

Figure S141 – Average (n = 3) absorbance readings from calcein loaded PE:PG 3:1 lipid vesicles (λ_{em} = 515 nm) following the addition of **4** at varying concentrations. Lysis value is triton (1 %). Blue = 1 minute, purple = 5 minutes, pink = 10 minutes, orange = 15 minutes, red = 20 minutes. Error = standard deviation of the mean.

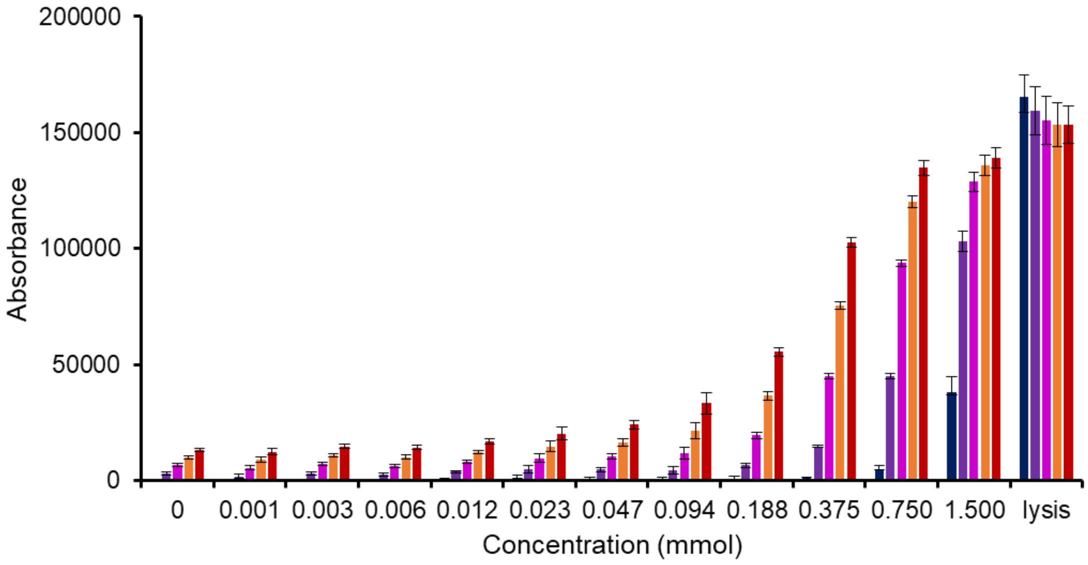

Figure S142 – Average (n = 3) absorbance readings from calcein loaded PE:PG 3:1 lipid vesicles (λ_{em} = 515 nm) following the addition of **5** at varying concentrations. Lysis value is triton (1 %). Blue = 1 minute, purple = 5 minutes, pink = 10 minutes, orange = 15 minutes, red = 20 minutes. Error = standard deviation of the mean.

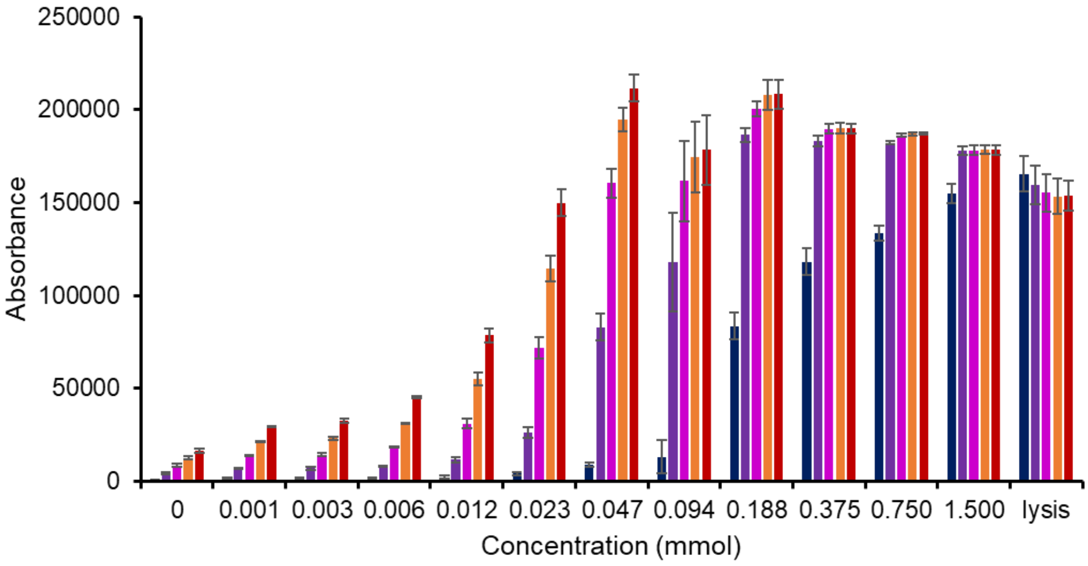

Figure S143 – Average (n = 3) absorbance readings from calcein loaded PE:PG 3:1 lipid vesicles (λ_{em} = 515 nm) following the addition of **a** at varying concentrations. Lysis value is triton (1 %). Blue = 1 minute, purple = 5 minutes, pink = 10 minutes, orange = 15 minutes, red = 20 minutes. Error = standard deviation of the mean.

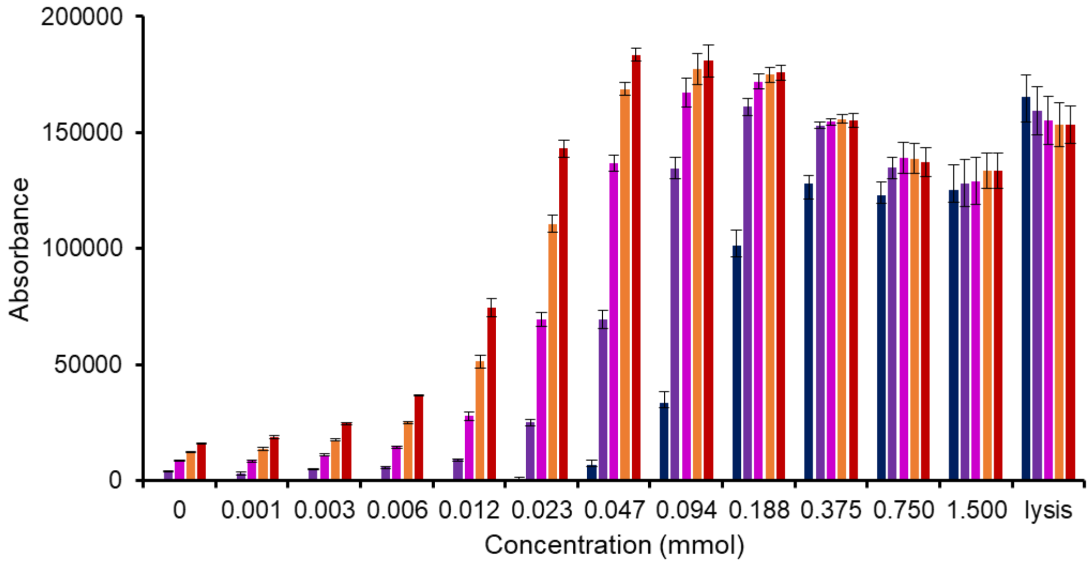

Figure S144 – Average (n = 3) absorbance readings from calcein loaded PE:PG 3:1 lipid vesicles (λ_{em} = 515 nm) following the addition of **b** at varying concentrations. Lysis value is triton (1 %). Blue = 1 minute, purple = 5 minutes, pink = 10 minutes, orange = 15 minutes, red = 20 minutes. Error = standard deviation of the mean.

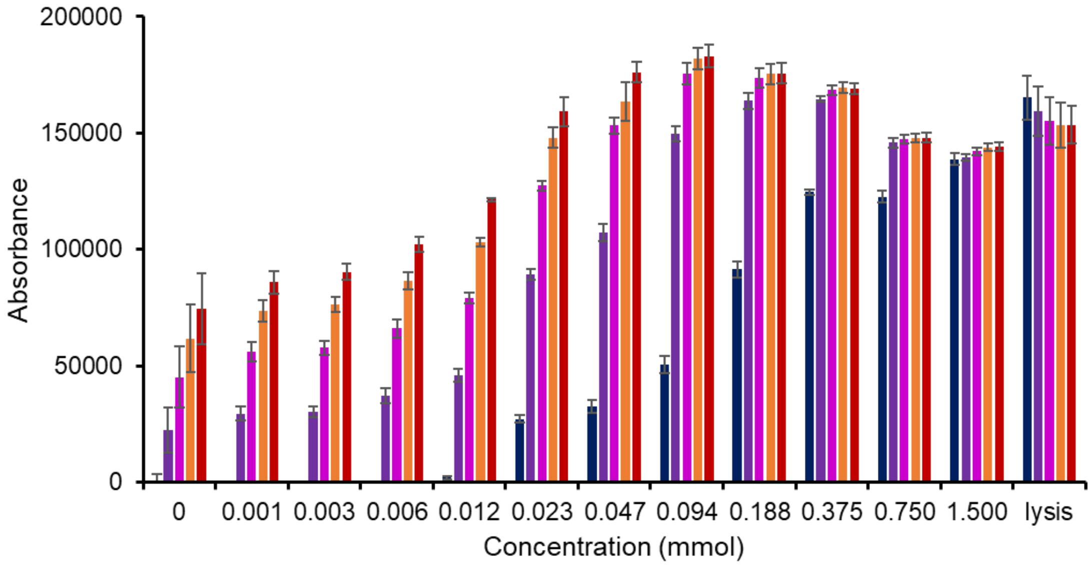

Figure S145 – Average (n = 3) absorbance readings from calcein loaded PE:PG 3:1 lipid vesicles (λ_{em} = 515 nm) following the addition of **c** at varying concentrations. Lysis value is triton (1 %). Blue = 1 minute, purple = 5 minutes, pink = 10 minutes, orange = 15 minutes, red = 20 minutes. Error = standard deviation of the mean.

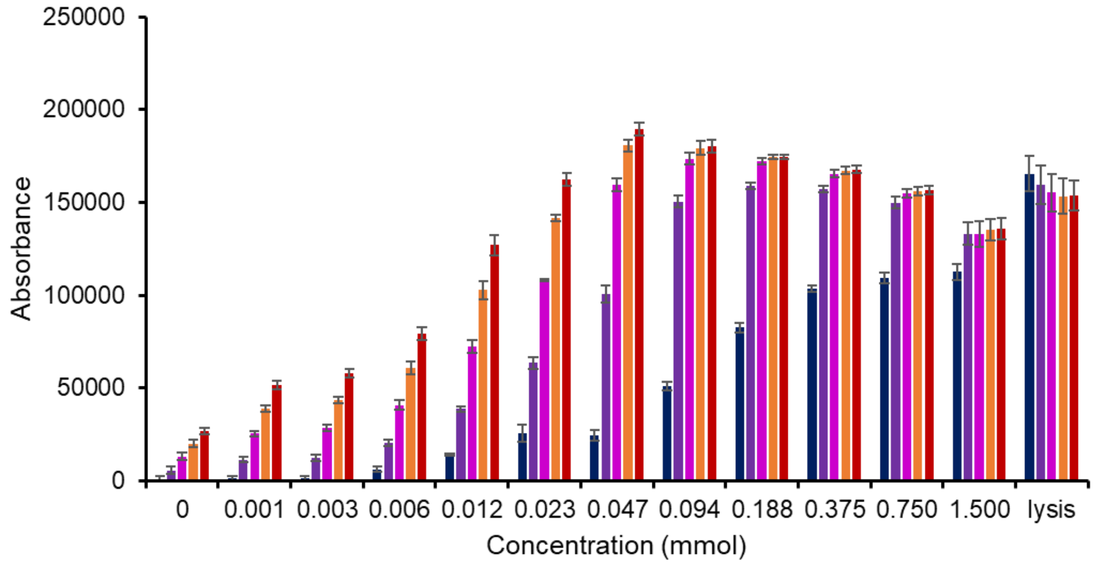

Figure S146 – Average (n = 3) absorbance readings from calcein loaded PE:PG 3:1 lipid vesicles (λ_{em} = 515 nm) following the addition of **d** at varying concentrations. Lysis value is triton (1 %). Blue = 1 minute, purple = 5 minutes, pink = 10 minutes, orange = 15 minutes, red = 20 minutes. Error = standard deviation of the mean.

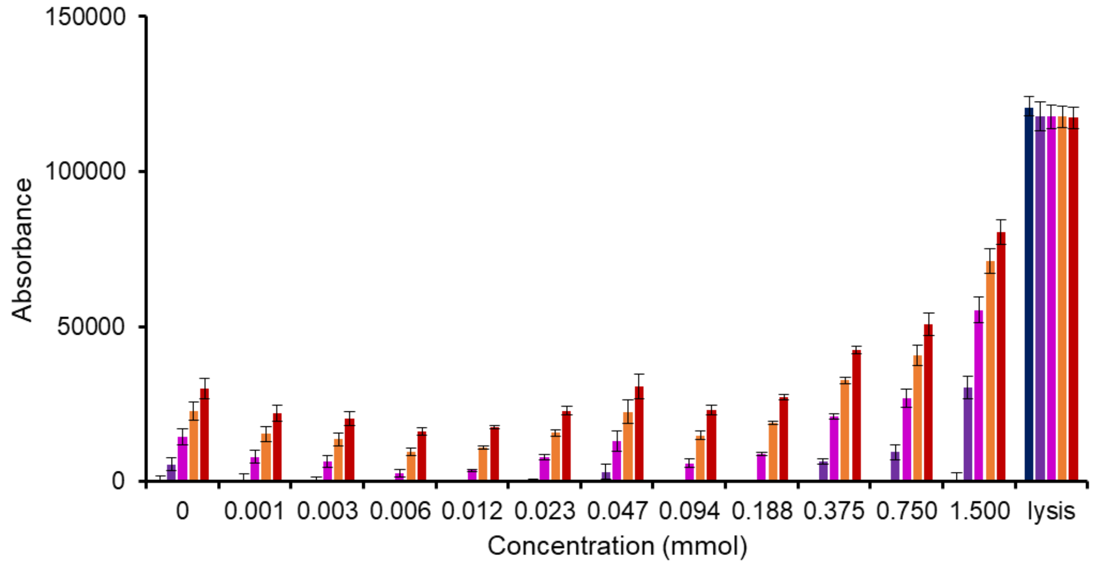

Figure S147 – Average (n = 3) absorbance readings from calcein loaded PE:PG 3:1 lipid vesicles (λ_{em} = 515 nm) following the addition of **e** at varying concentrations. Lysis value is triton (1 %). Blue = 1 minute, purple = 5 minutes, pink = 10 minutes, orange = 15 minutes, red = 20 minutes. Error = standard deviation of the mean.

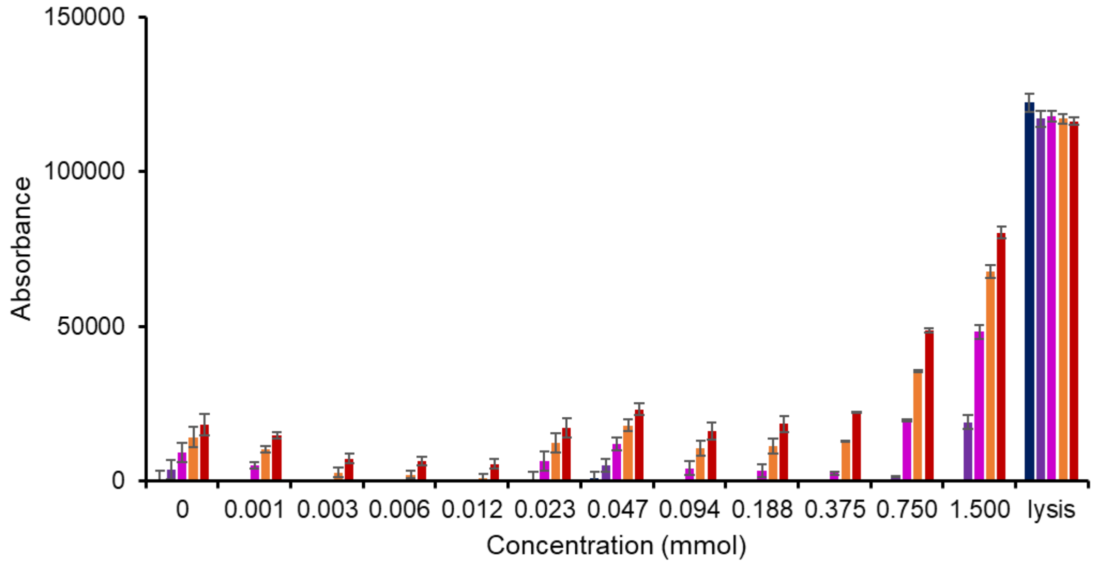

Figure S148 – Average (n = 3) absorbance readings from calcein loaded PE:PG 3:1 lipid vesicles (λ_{em} = 515 nm) following the addition of **f** at varying concentrations. Lysis value is triton (1 %). Blue = 1 minute, purple = 5 minutes, pink = 10 minutes, orange = 15 minutes, red = 20 minutes. Error = standard deviation of the mean.

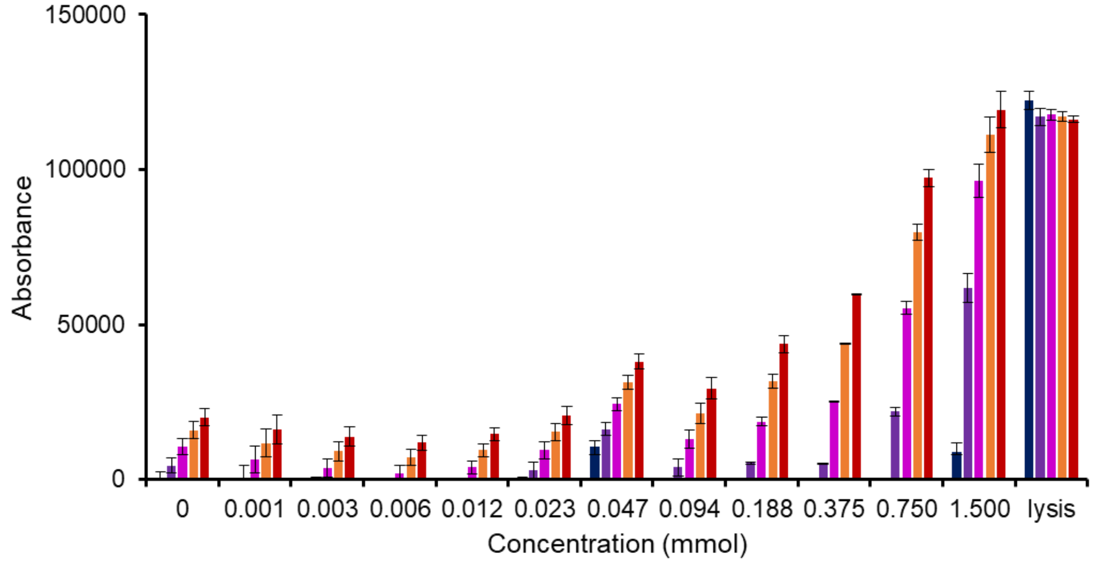

Figure S149 – Average (n = 3) absorbance readings from calcein loaded PE:PG 3:1 lipid vesicles (λ_{em} = 515 nm) following the addition of **g** at varying concentrations. Lysis value is triton (1 %). Blue = 1 minute, purple = 5 minutes, pink = 10 minutes, orange = 15 minutes, red = 20 minutes. Error = standard deviation of the mean.

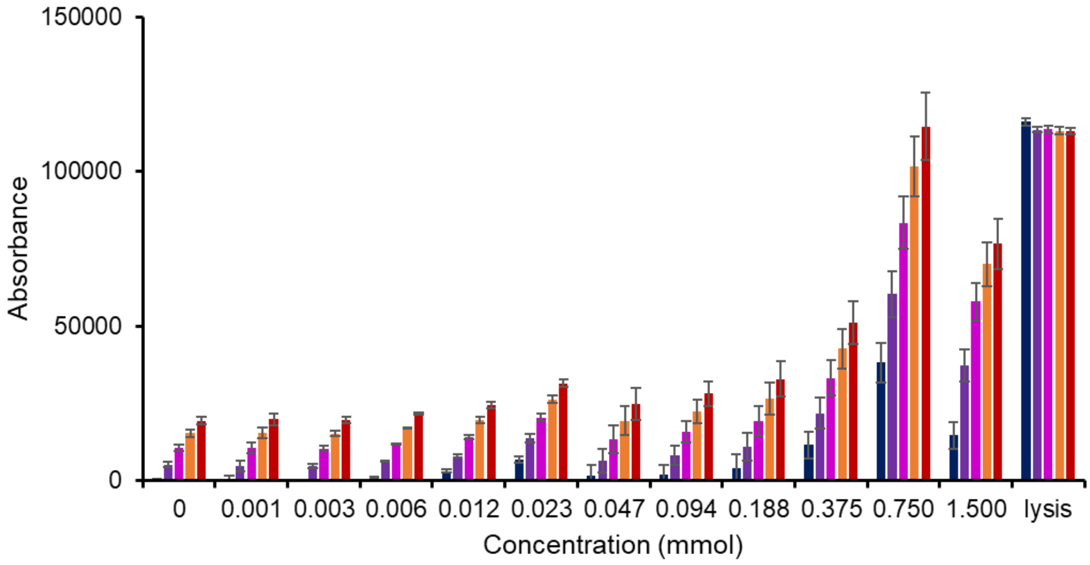

Figure S150 – Average (n = 3) absorbance readings from calcein loaded PE:PG 3:1 lipid vesicles (λ_{em} = 515 nm) following the addition of **h** at varying concentrations. Lysis value is triton (1 %). Blue = 1 minute, purple = 5 minutes, pink = 10 minutes, orange = 15 minutes, red = 20 minutes. Error = standard deviation of the mean.

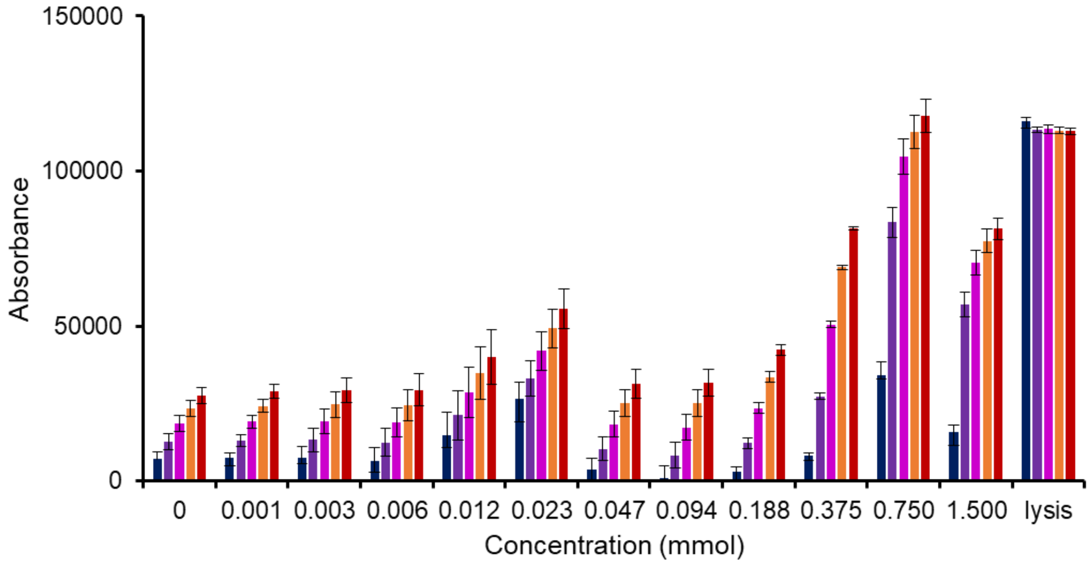

Figure S151 – Average (n = 3) absorbance readings from calcein loaded PE:PG 3:1 lipid vesicles (λ_{em} = 515 nm) following the addition of **i** at varying concentrations. Lysis value is triton (1 %). Blue = 1 minute, purple = 5 minutes, pink = 10 minutes, orange = 15 minutes, red = 20 minutes. Error = standard deviation of the mean.

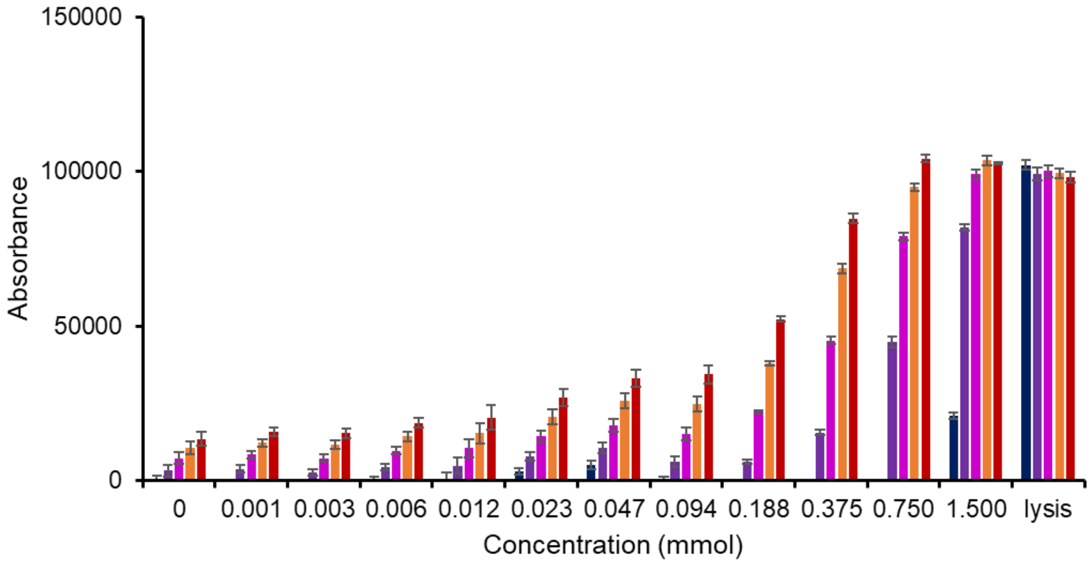

Figure S152 – Average (n = 3) absorbance readings from calcein loaded PE:PG 3:1 lipid vesicles (λ_{em} = 515 nm) following the addition of **j** at varying concentrations. Lysis value is triton (1 %). Blue = 1 minute, purple = 5 minutes, pink = 10 minutes, orange = 15 minutes, red = 20 minutes. Error = standard deviation of the mean.

PE:PG 1:1 lipid titrations

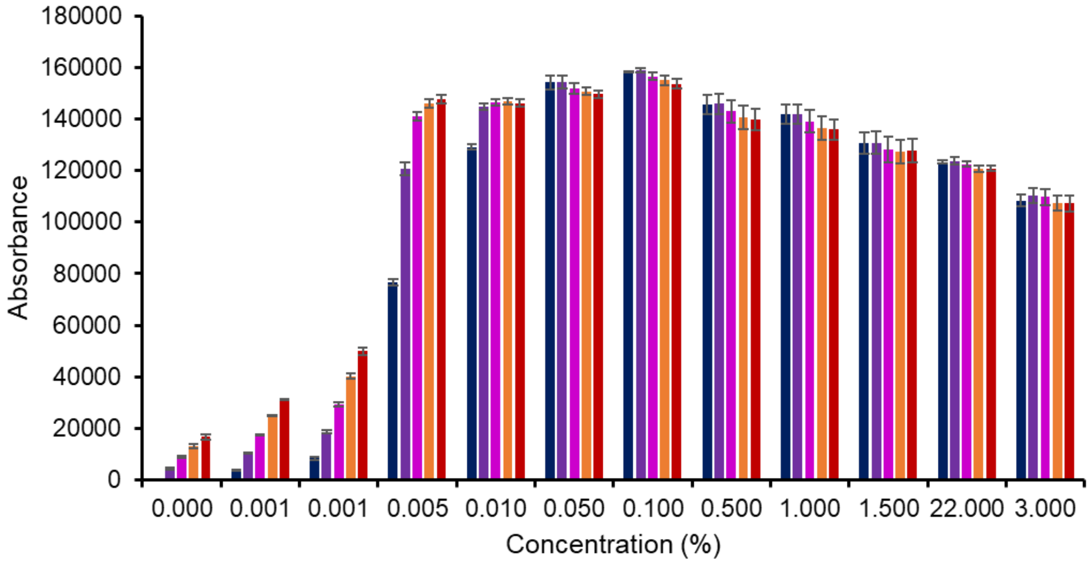

Figure S153 – Average (n = 3) absorbance readings from calcein loaded PE:PG 1:1 lipid vesicles (λ_{em} = 515 nm) following the addition of triton at varying concentrations. Blue = 1 minute, purple = 5 minutes, pink = 10 minutes, orange = 15 minutes, red = 20 minutes. Error = standard deviation of the mean.

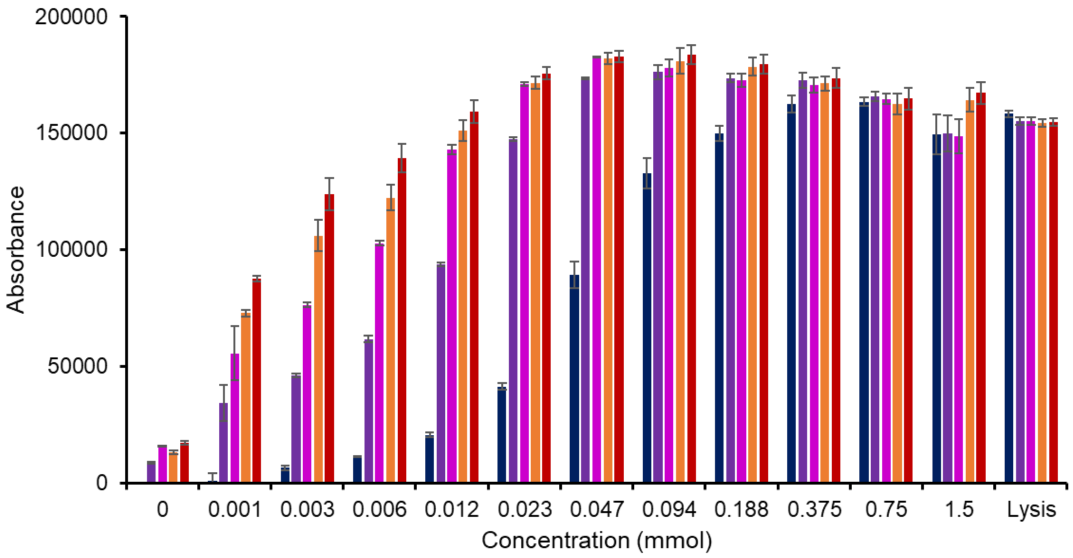

Figure S154 – Average (n = 3) absorbance readings from calcein loaded PE:PG 3:1 lipid vesicles (λ_{em} = 515 nm) following the addition of **1** at varying concentrations. Lysis value is triton (1 %). Blue = 1 minute, purple = 5 minutes, pink = 10 minutes, orange = 15 minutes, red = 20 minutes. Error = standard deviation of the mean.

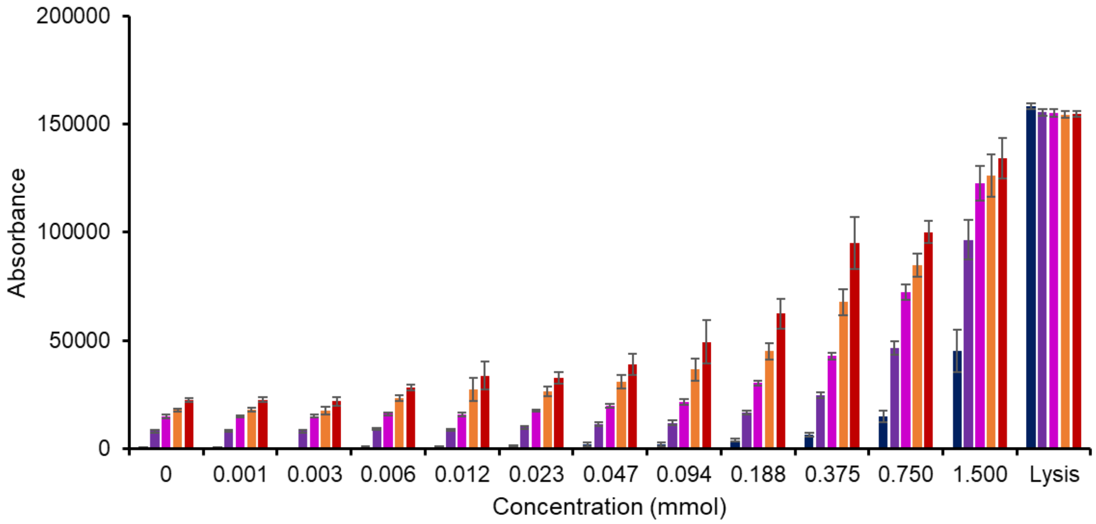

Figure S155 – Average (n = 3) absorbance readings from calcein loaded PE:PG 3:1 lipid vesicles (λ_{em} = 515 nm) following the addition of **2** at varying concentrations. Lysis value is triton (1 %). Blue = 1 minute, purple = 5 minutes, pink = 10 minutes, orange = 15 minutes, red = 20 minutes. Error = standard deviation of the mean.

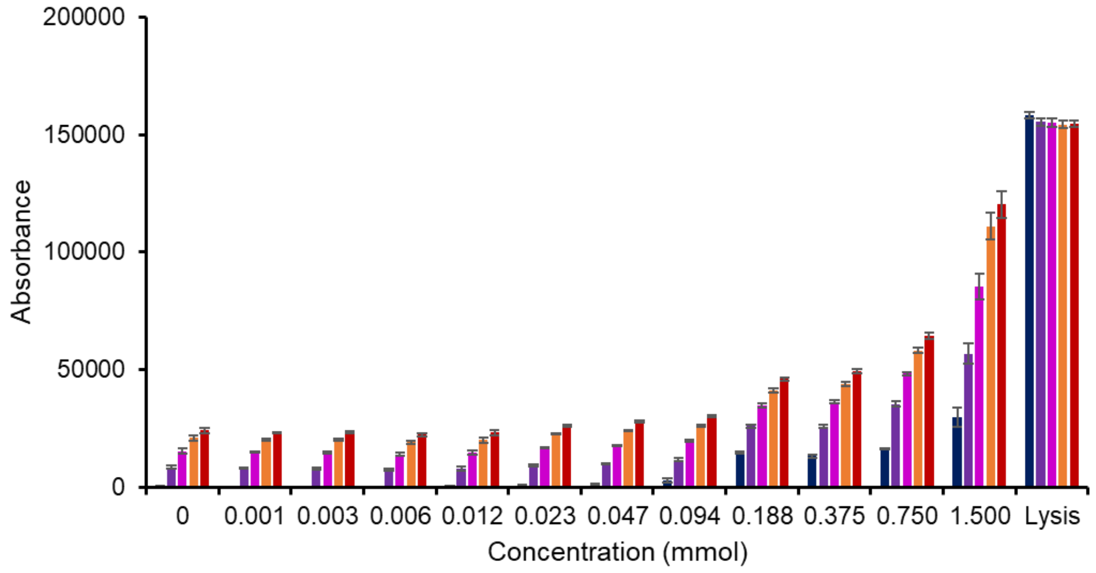

Figure S156 – Average (n = 3) absorbance readings from calcein loaded PE:PG 3:1 lipid vesicles (λ_{em} = 515 nm) following the addition of **3** at varying concentrations. Lysis value is triton (1 %). Blue = 1 minute, purple = 5 minutes, pink = 10 minutes, orange = 15 minutes, red = 20 minutes. Error = standard deviation of the mean.

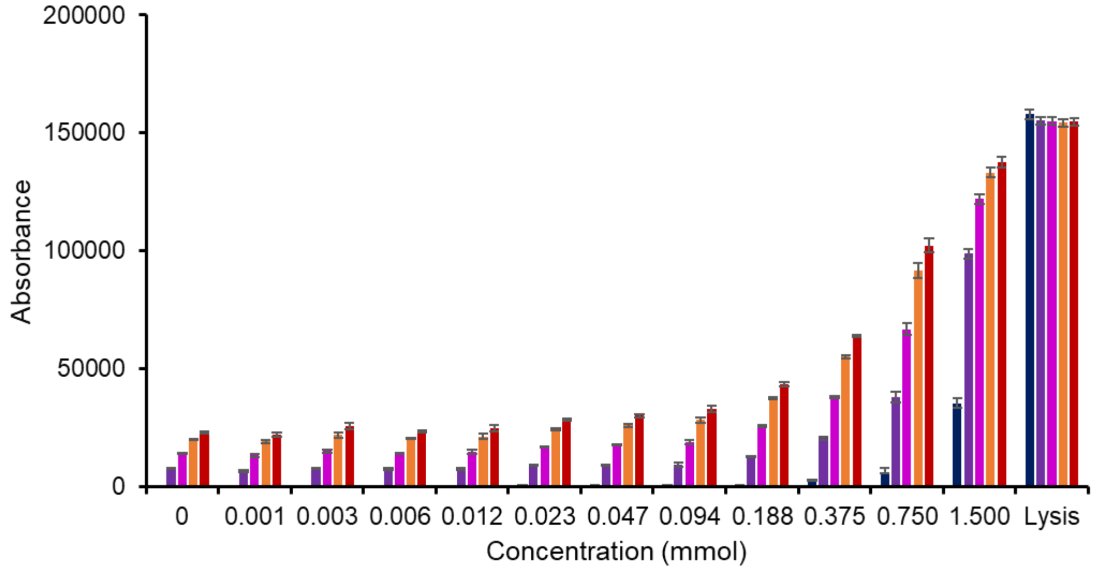

Figure S157 – Average (n = 3) absorbance readings from calcein loaded PE:PG 3:1 lipid vesicles (λ_{em} = 515 nm) following the addition of **4** at varying concentrations. Lysis value is triton (1 %). Blue = 1 minute, purple = 5 minutes, pink = 10 minutes, orange = 15 minutes, red = 20 minutes. Error = standard deviation of the mean.

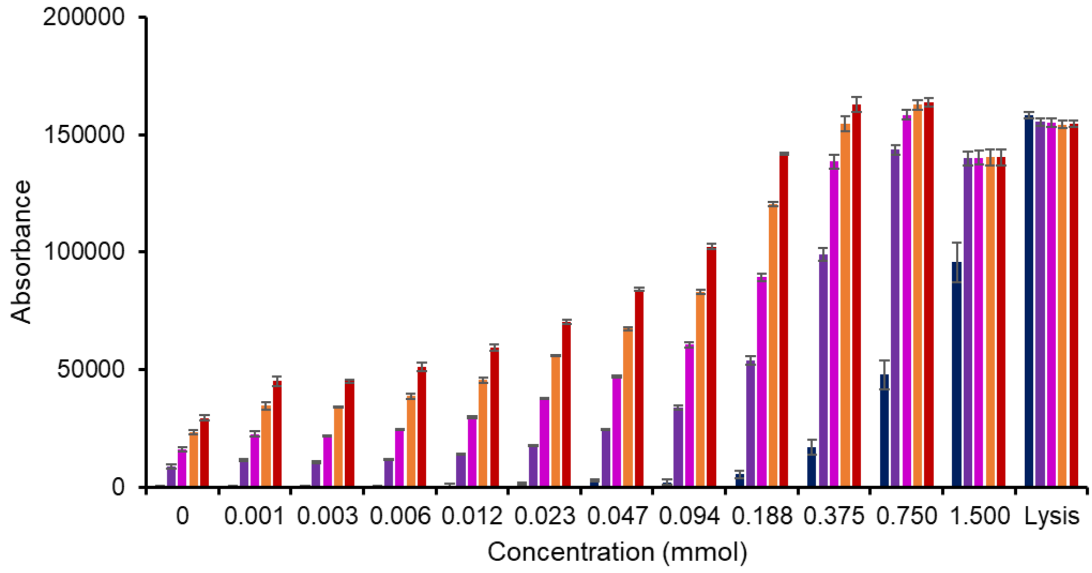

Figure S158 – Average (n = 3) absorbance readings from calcein loaded PE:PG 3:1 lipid vesicles (λ_{em} = 515 nm) following the addition of **5** at varying concentrations. Lysis value is triton (1 %). Blue = 1 minute, purple = 5 minutes, pink = 10 minutes, orange = 15 minutes, red = 20 minutes. Error = standard deviation of the mean.

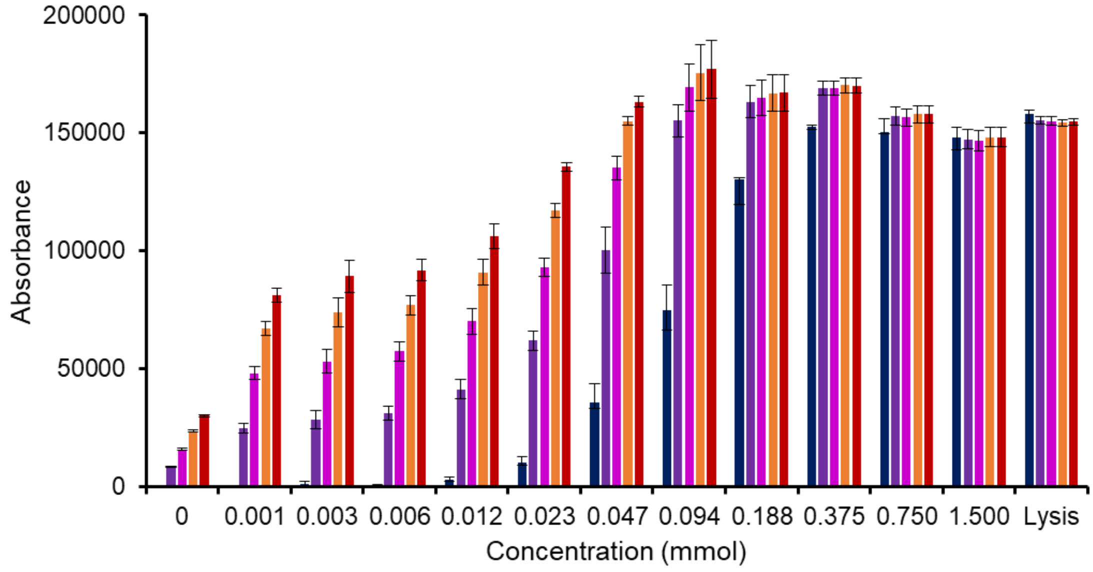

Figure S159 – Average (n = 3) absorbance readings from calcein loaded PE:PG 3:1 lipid vesicles (λ_{em} = 515 nm) following the addition of **a** at varying concentrations. Lysis value is triton (1 %). Blue = 1 minute, purple = 5 minutes, pink = 10 minutes, orange = 15 minutes, red = 20 minutes. Error = standard deviation of the mean.

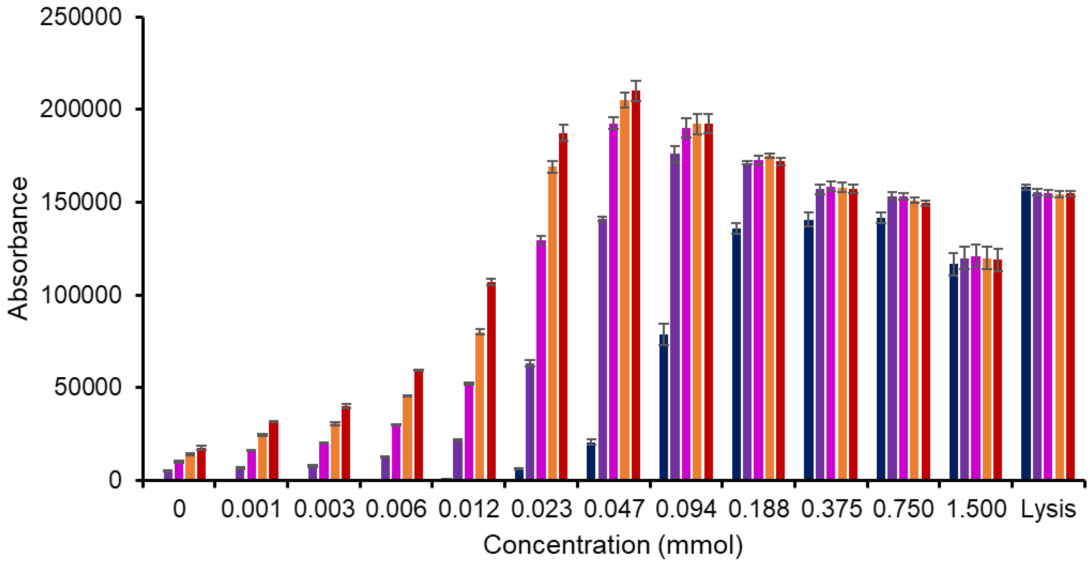

Figure S160 – Average (n = 3) absorbance readings from calcein loaded PE:PG 3:1 lipid vesicles (λ_{em} = 515 nm) following the addition of **b** at varying concentrations. Lysis value is triton (1 %). Blue = 1 minute, purple = 5 minutes, pink = 10 minutes, orange = 15 minutes, red = 20 minutes. Error = standard deviation of the mean.

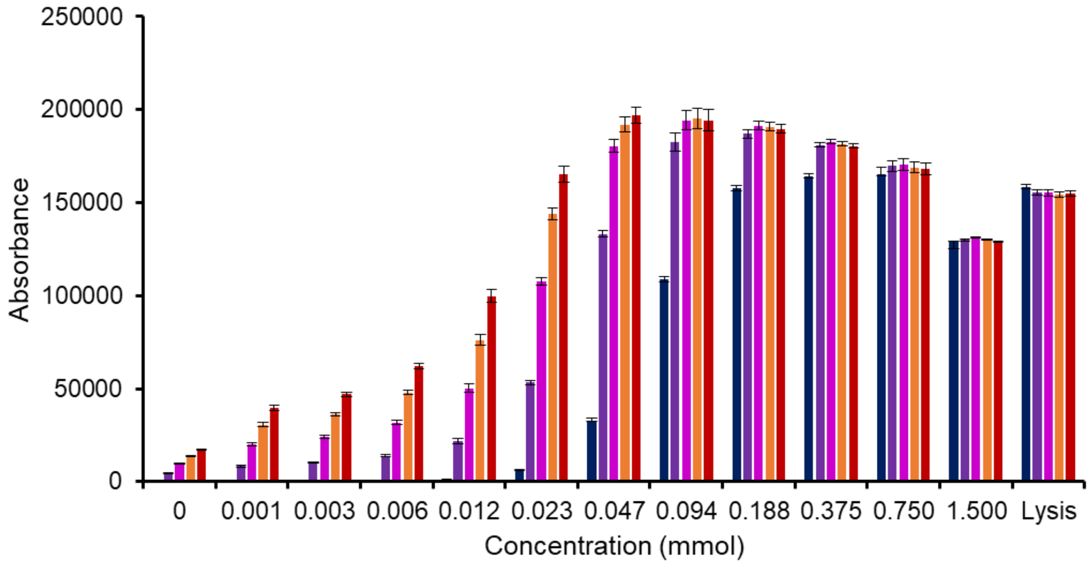

Figure S161 – Average (n = 3) absorbance readings from calcein loaded PE:PG 3:1 lipid vesicles (λ_{em} = 515 nm) following the addition of **c** at varying concentrations. Lysis value is triton (1 %). Blue = 1 minute, purple = 5 minutes, pink = 10 minutes, orange = 15 minutes, red = 20 minutes. Error = standard deviation of the mean.

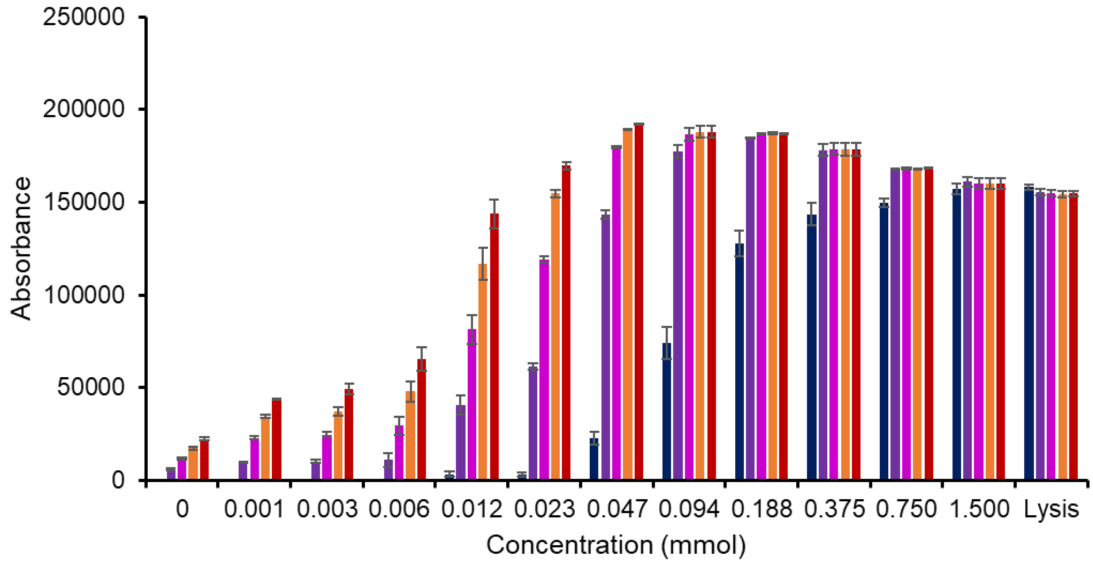

Figure S162 – Average (n = 3) absorbance readings from calcein loaded PE:PG 3:1 lipid vesicles (λ_{em} = 515 nm) following the addition of **d** at varying concentrations. Lysis value is triton (1 %). Blue = 1 minute, purple = 5 minutes, pink = 10 minutes, orange = 15 minutes, red = 20 minutes. Error = standard deviation of the mean.

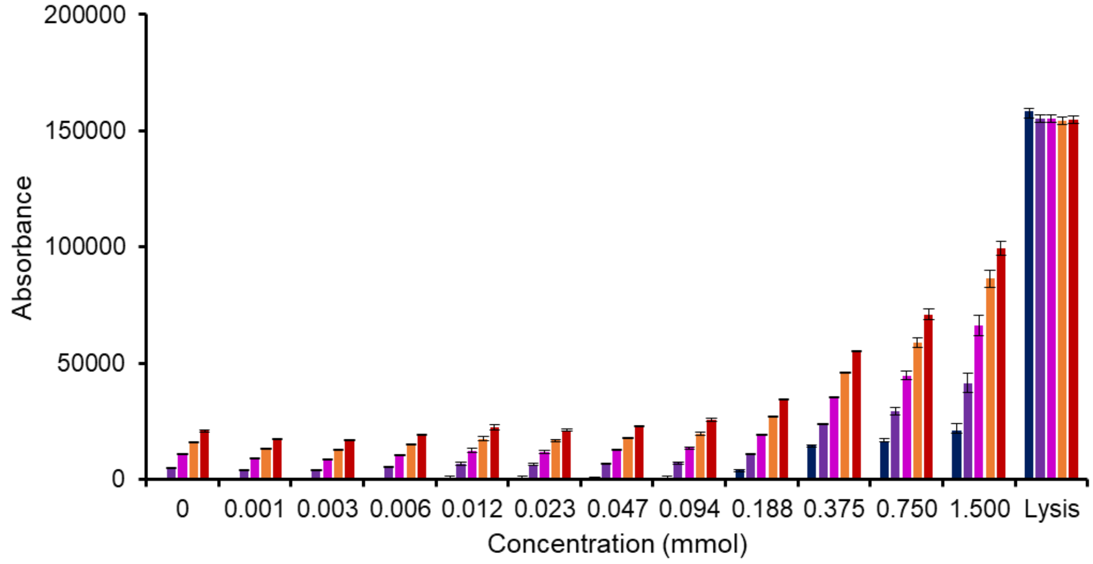

Figure S163 – Average (n = 3) absorbance readings from calcein loaded PE:PG 3:1 lipid vesicles (λ_{em} = 515 nm) following the addition of **e** at varying concentrations. Lysis value is triton (1 %). Blue = 1 minute, purple = 5 minutes, pink = 10 minutes, orange = 15 minutes, red = 20 minutes. Error = standard deviation of the mean.

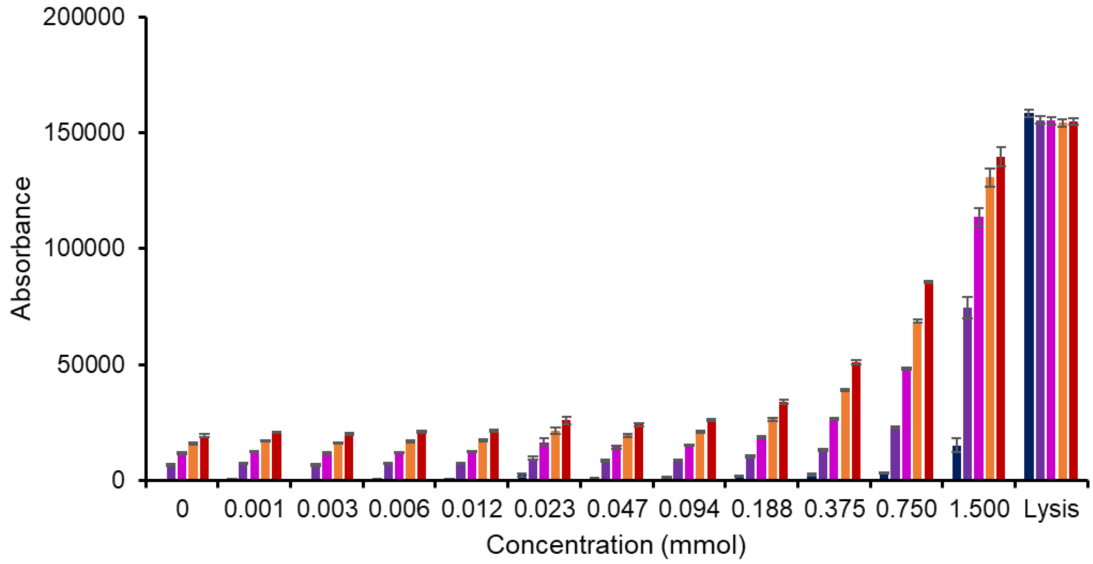

Figure S164 – Average (n = 3) absorbance readings from calcein loaded PE:PG 3:1 lipid vesicles (λ_{em} = 515 nm) following the addition of **f** at varying concentrations. Lysis value is triton (1 %). Blue = 1 minute, purple = 5 minutes, pink = 10 minutes, orange = 15 minutes, red = 20 minutes. Error = standard deviation of the mean.

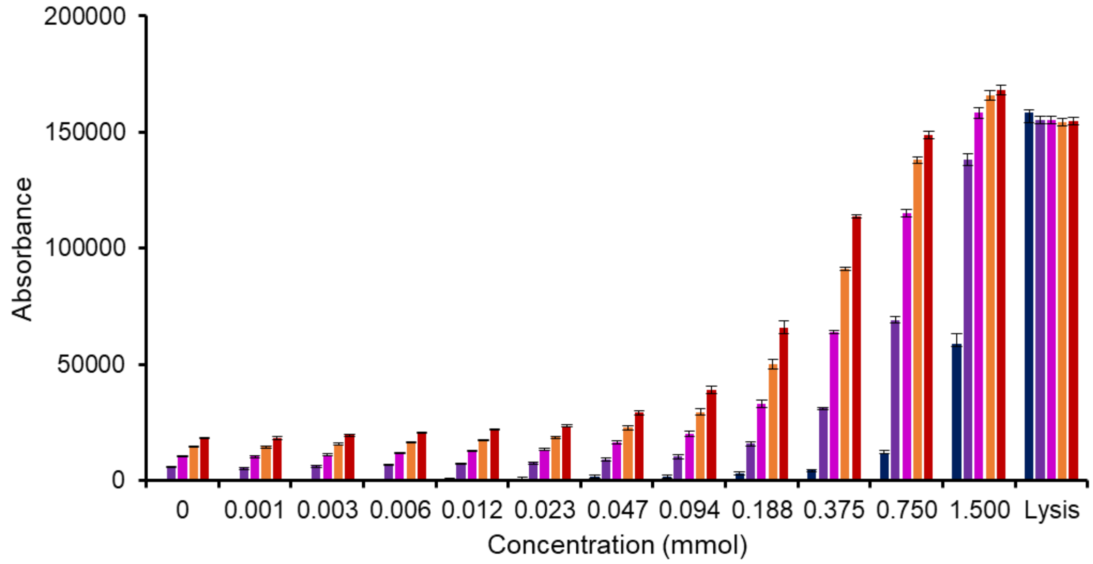

Figure S165 – Average (n = 3) absorbance readings from calcein loaded PE:PG 3:1 lipid vesicles (λ_{em} = 515 nm) following the addition of **g** at varying concentrations. Lysis value is triton (1 %). Blue = 1 minute, purple = 5 minutes, pink = 10 minutes, orange = 15 minutes, red = 20 minutes. Error = standard deviation of the mean.

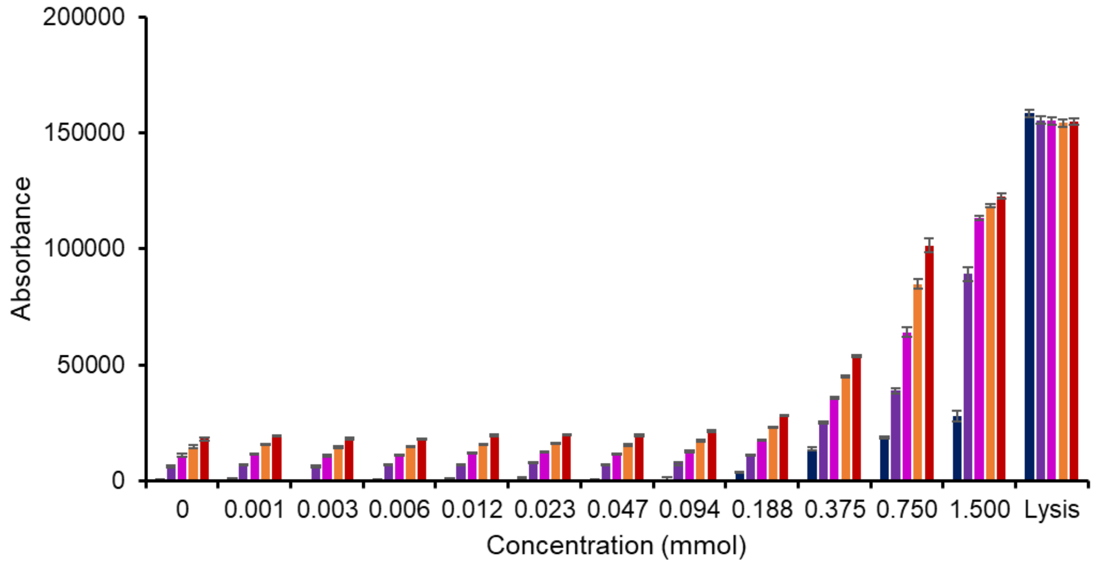

Figure S166 – Average (n = 3) absorbance readings from calcein loaded PE:PG 3:1 lipid vesicles (λ_{em} = 515 nm) following the addition of **h** at varying concentrations. Lysis value is triton (1 %). Blue = 1 minute, purple = 5 minutes, pink = 10 minutes, orange = 15 minutes, red = 20 minutes. Error = standard deviation of the mean.

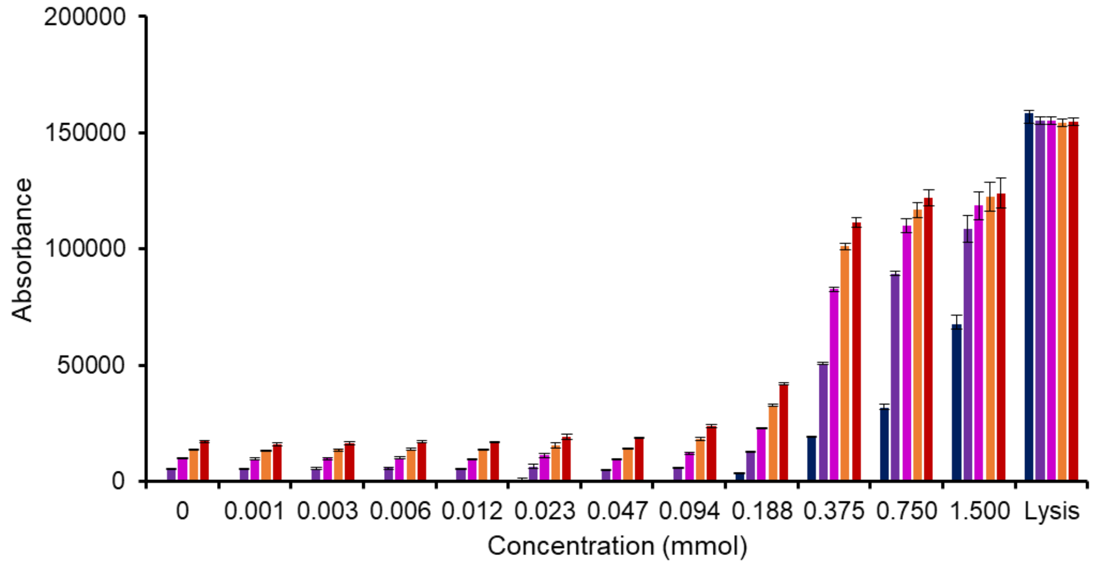

Figure S167 – Average (n = 3) absorbance readings from calcein loaded PE:PG 3:1 lipid vesicles (λ_{em} = 515 nm) following the addition of **i** at varying concentrations. Lysis value is triton (1 %). Blue = 1 minute, purple = 5 minutes, pink = 10 minutes, orange = 15 minutes, red = 20 minutes. Error = standard deviation of the mean.

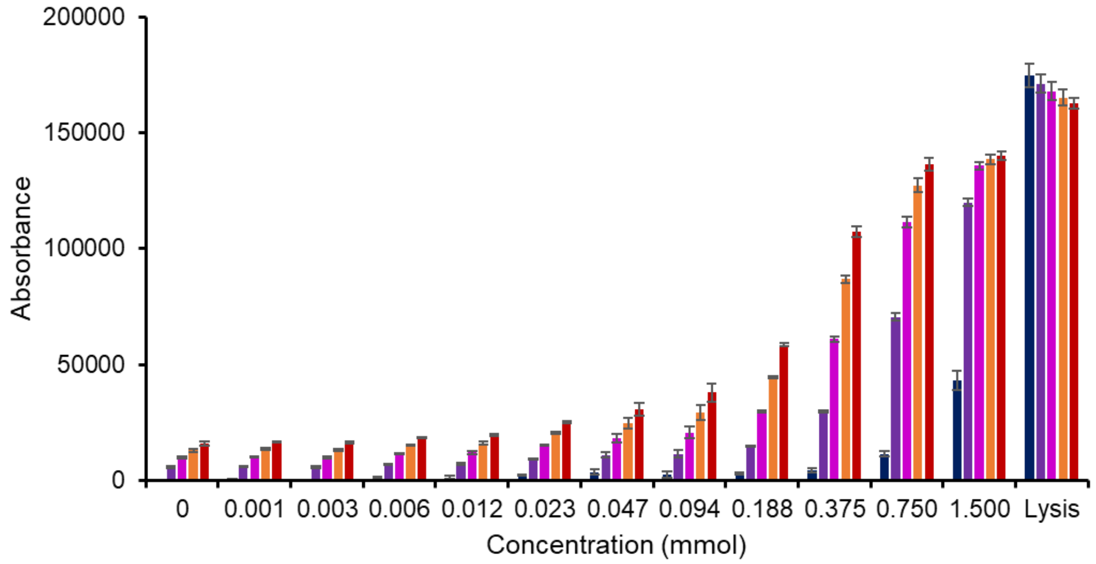

Figure S168 – Average (n=3) absorbance readings of calcein loaded PE:PG 3:1 lipid vesicles (λ_{em} = 515 nm) following the addition of **j** at varying concentrations. Lysis value is triton (1 %). Blue = 1 minute, purple = 5 minutes, pink = 10 minutes, orange = 15 minutes, red = 20 minutes. Error = standard deviation of the mean.

Section S14: Determination of vesicle lysis EC_{50} values

Data was originally fitted to the Hill equation using origin software. If the fitting of these vesicle lysis data to the Hill equation failed then the data were fitted to a linear line of best fit to estimate EC_{50} values, this tended to occur where the compounds were less active and the EC_{50} values were clearly above 0.1 mM. Where linear fitting failed, the data remained unfitted.

PG vesicle lysis titrations

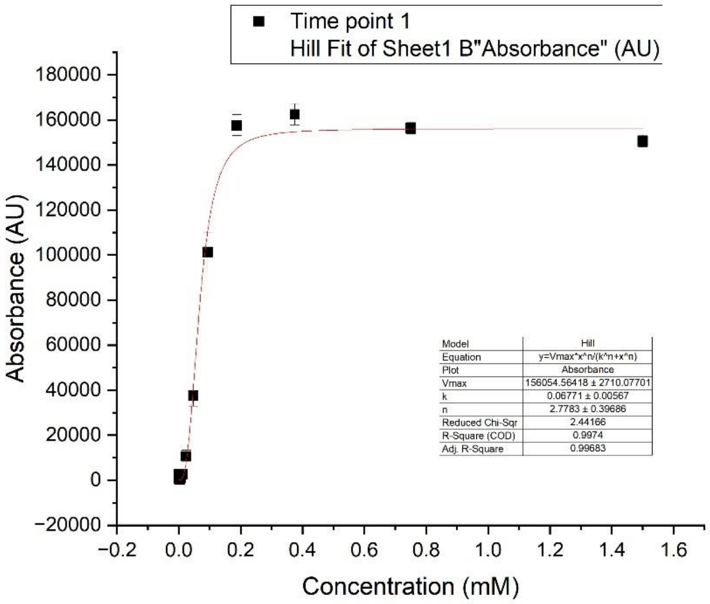

Figure S169 – Graph showing the lysis of calcein loaded PG vesicles at 1 minute, with respect to increasing concentration of **1**. Data was then fitted to the Hill equation. Average of n= 3, error = one standard deviation of the mean.

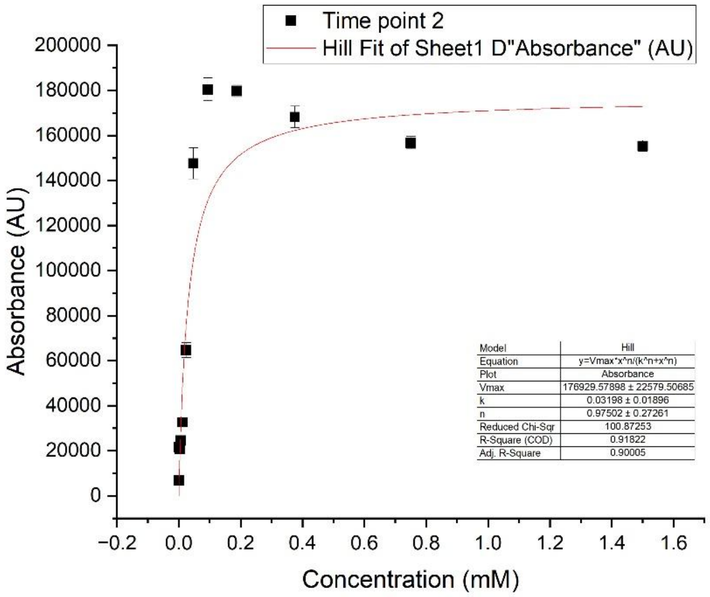

Figure S170 – Graph showing the lysis of calcein loaded PG vesicles at 5 minutes, with respect to increasing concentration of **1**. Data was then fitted to the Hill equation. Average of n= 3, error = one standard deviation of the mean.

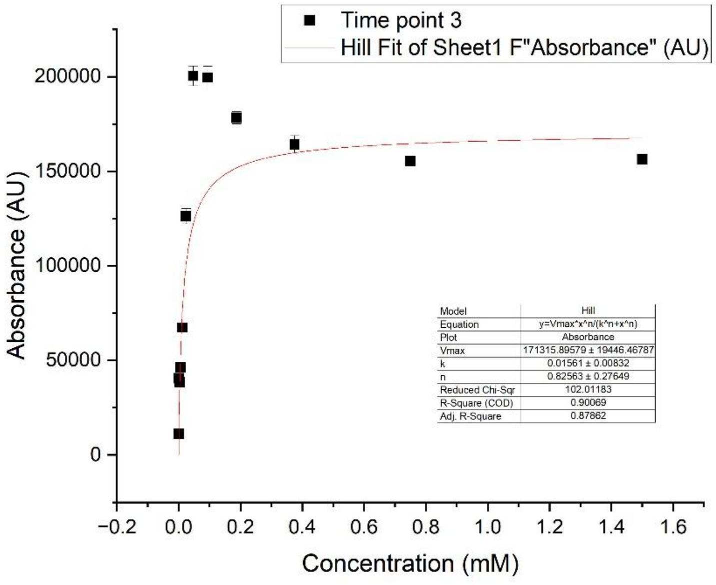

Figure S171 – Graph showing the lysis of calcein loaded PG vesicles at 10 minutes, with respect to increasing concentration of **1**. Data was then fitted to the Hill equation. Average of n= 3, error = one standard deviation of the mean.

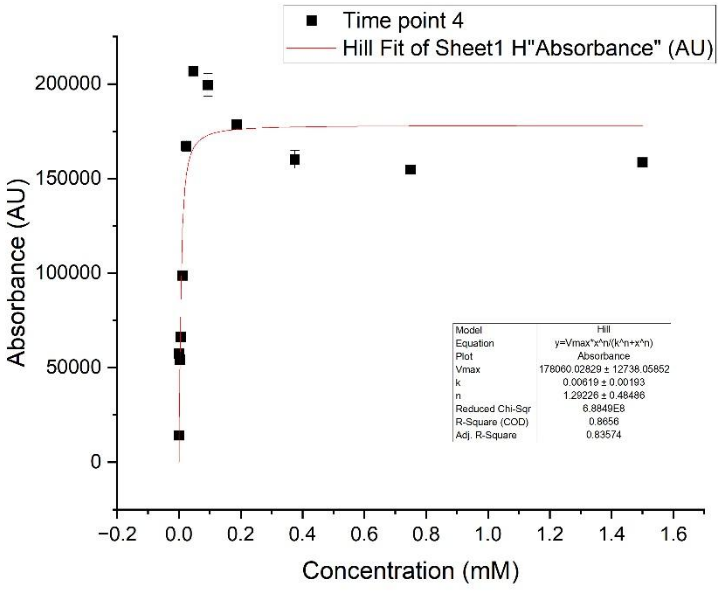

Figure S172 – Graph showing the lysis of calcein loaded PG vesicles at 15 minutes, with respect to increasing concentration of **1**. Data was then fitted to the Hill equation. Average of n= 3, error = one standard deviation of the mean.

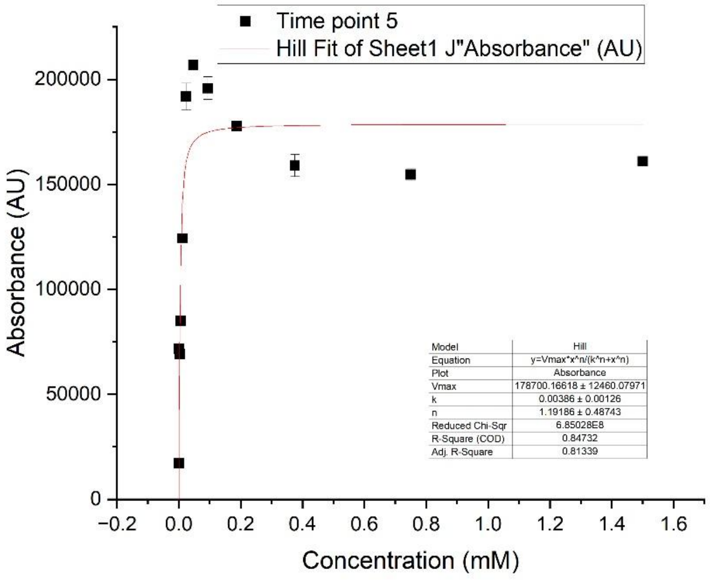

Figure S173 – Graph showing the lysis of calcein loaded PG vesicles at 20 minutes, with respect to increasing concentration of **1**. Data was then fitted to the Hill equation. Average of n= 3, error = one standard deviation of the mean.

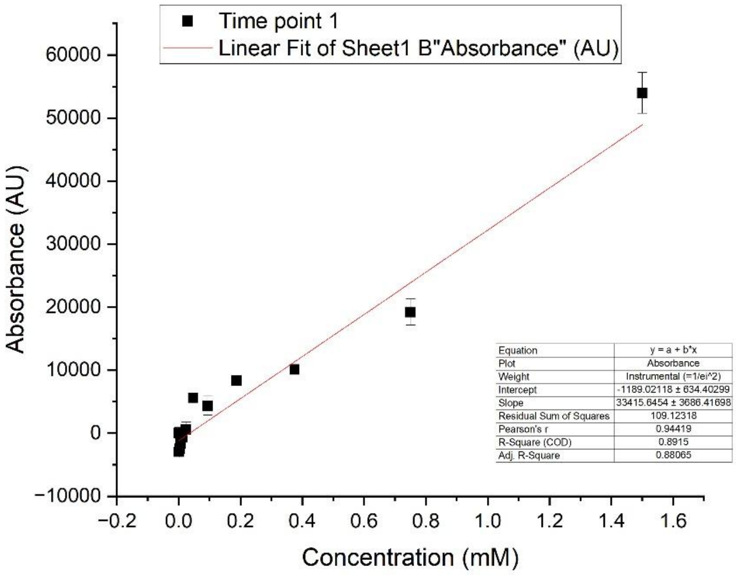

Figure S174 – Graph showing the lysis of calcein loaded PG vesicles at 1 minute, with respect to increasing concentration of **2**. Data was then fitted to a linear line of best fit. Average of n= 3, error = one standard deviation of the mean.

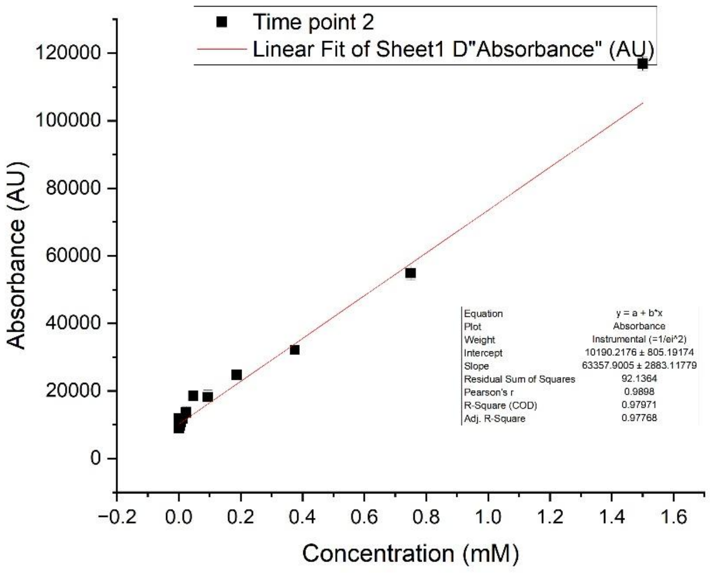

Figure S175 – Graph showing the lysis of calcein loaded PG vesicles at 5 minutes, with respect to increasing concentration of **2**. Data was then fitted to a linear line of best fit. Average of n= 3, error = one standard deviation of the mean.

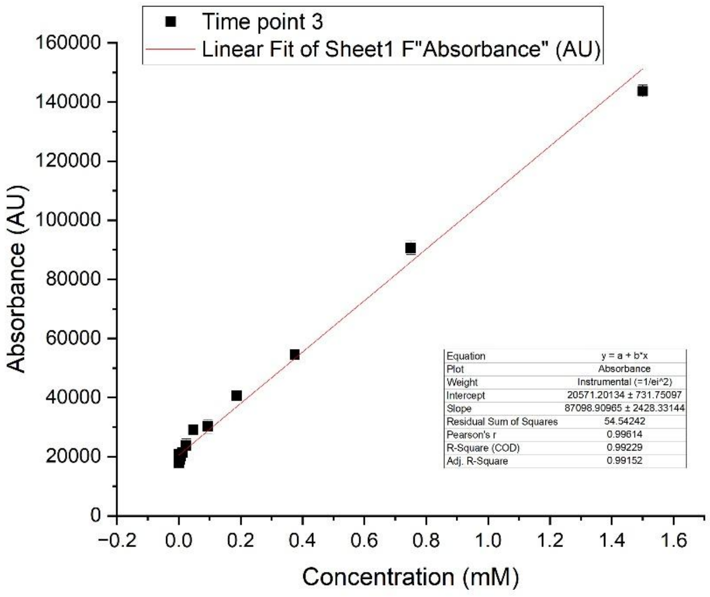

Figure S176 – Graph showing the lysis of calcein loaded PG vesicles at 10 minutes, with respect to increasing concentration of **2**. Data was then fitted to a linear line of best fit. Average of n= 3, error = one standard deviation of the mean.

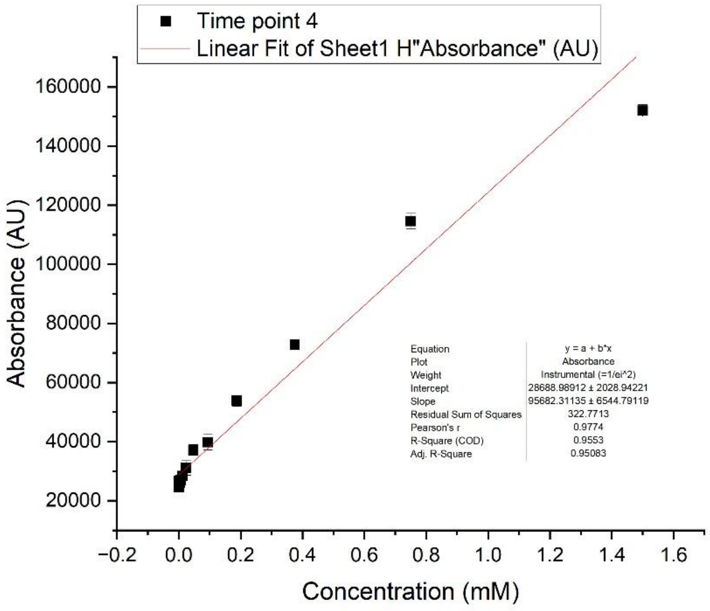

Figure S177 – Graph showing the lysis of calcein loaded PG vesicles at 15 minutes, with respect to increasing concentration of **2**. Data was then fitted to a linear line of best fit. Average of n= 3, error = one standard deviation of the mean.

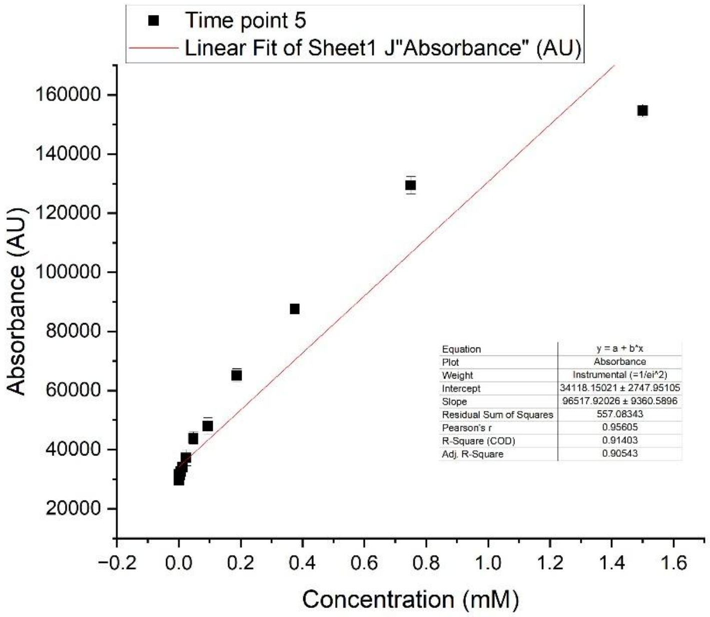

Figure S178 – Graph showing the lysis of calcein loaded PG vesicles at 20 minutes, with respect to increasing concentration of **2**. Data was then fitted to a linear line of best fit. Average of n= 3, error = one standard deviation of the mean.

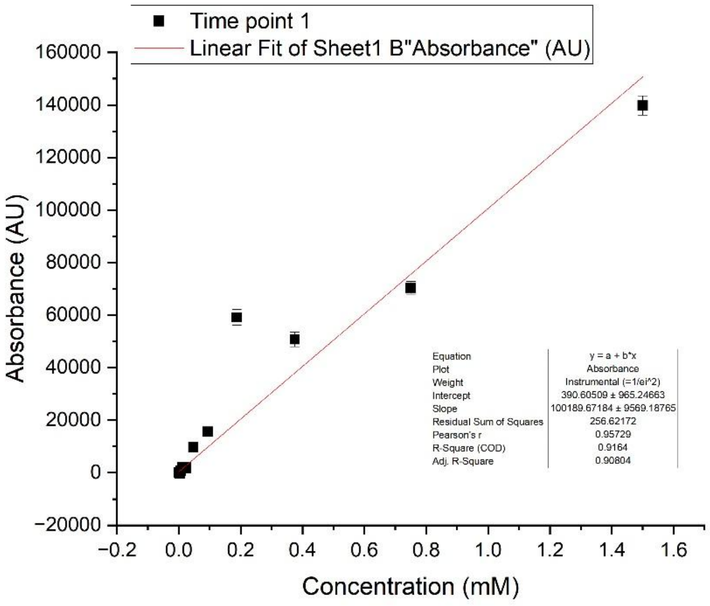

Figure S179 – Graph showing the lysis of calcein loaded PG vesicles at 1 minute, with respect to increasing concentration of **3**. Data was then fitted to a linear line of best fit. Average of n= 3, error = one standard deviation of the mean.

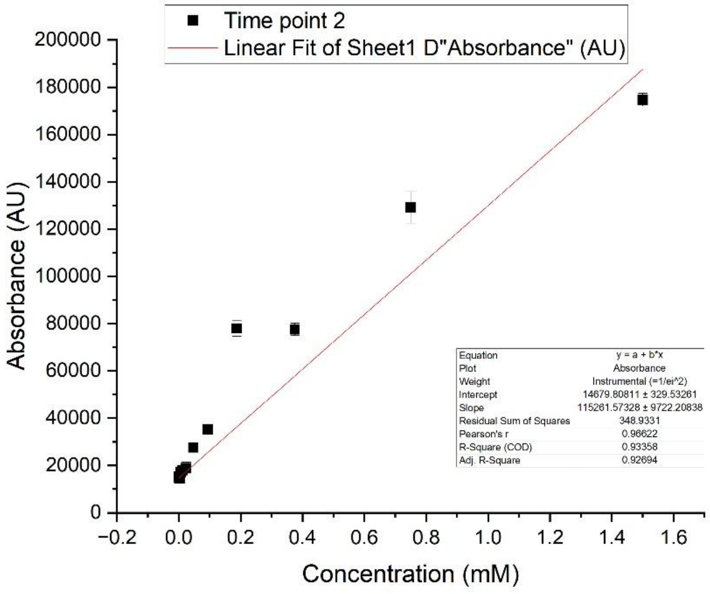

Figure S180 – Graph showing the lysis of calcein loaded PG vesicles at 5 minutes, with respect to increasing concentration of **3**. Data was then fitted to a linear line of best fit. Average of n= 3, error = one standard deviation of the mean.

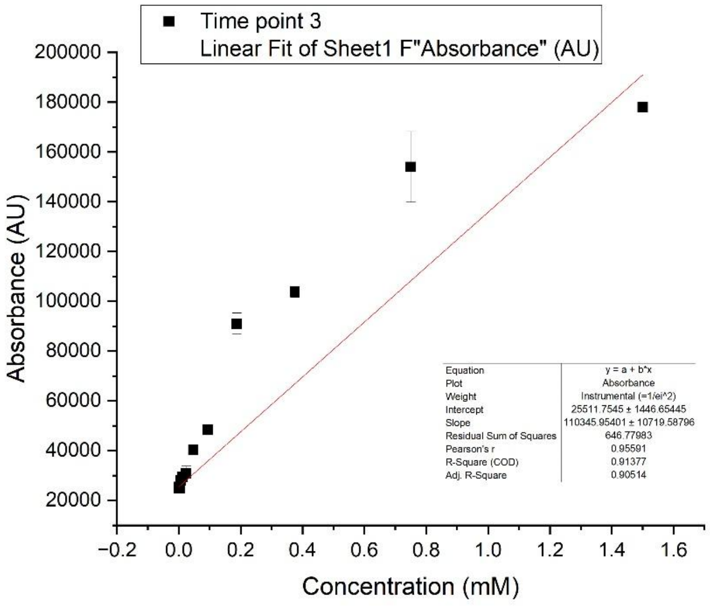

Figure S181 – Graph showing the lysis of calcein loaded PG vesicles at 10 minutes, with respect to increasing concentration of **3**. Data was then fitted to a linear line of best fit. Average of n= 3, error = one standard deviation of the mean.

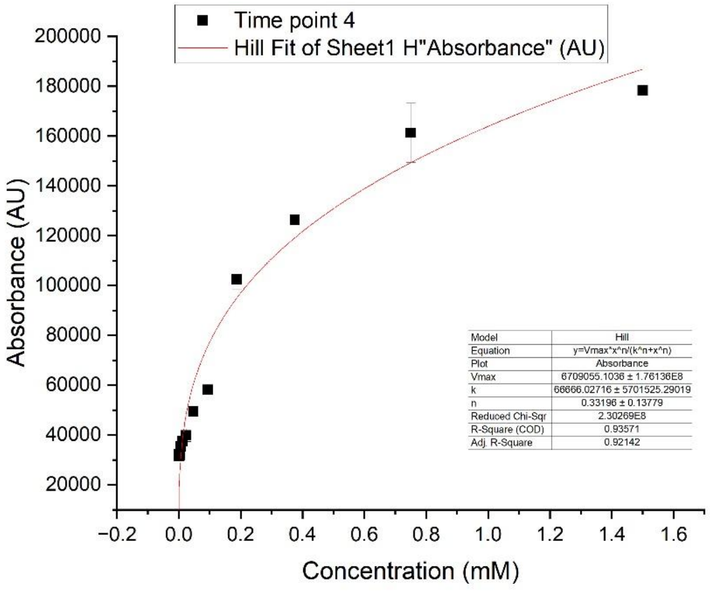

Figure S182 – Graph showing the lysis of calcein loaded PG vesicles at 15 minutes, with respect to increasing concentration of **3**. Data was then fitted to the Hill equation. Average of n= 3, error = one standard deviation of the mean.

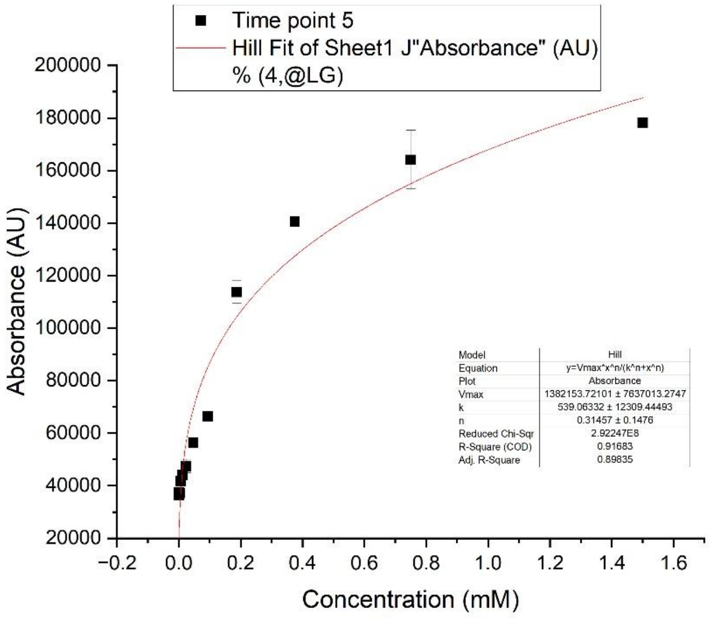

Figure S183 – Graph showing the lysis of calcein loaded PG vesicles at 20 minutes, with respect to increasing concentration of **3**. Data was then fitted to the Hill equation. Average of n= 3, error = one standard deviation of the mean.

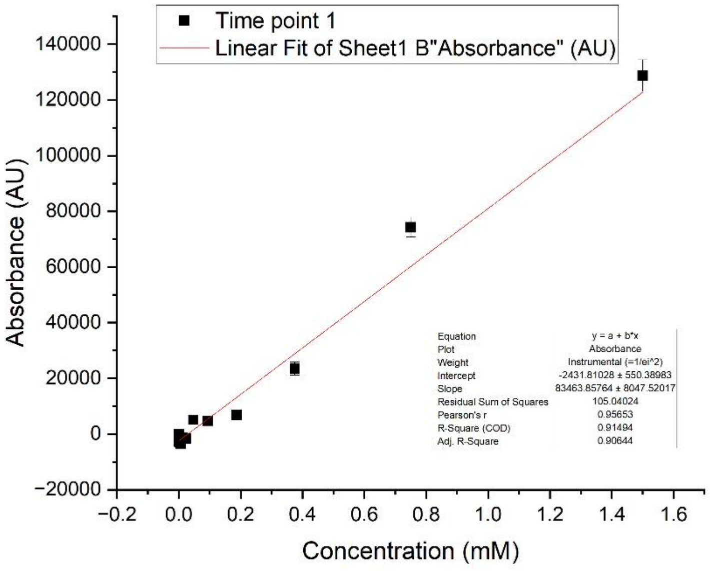

Figure S184 – Graph showing the lysis of calcein loaded PG vesicles at 1 minute, with respect to increasing concentration of **4**. Data was then fitted to a linear line of best fit. Average of n= 3, error = one standard deviation of the mean.

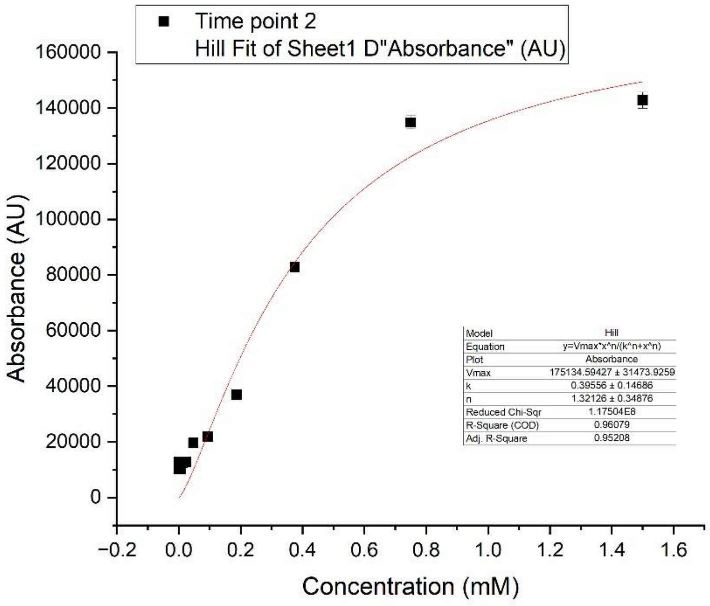

Figure S185 – Graph showing the lysis of calcein loaded PG vesicles at 5 minutes, with respect to increasing concentration of **4**. Data was then fitted to the Hill equation. Average of n= 3, error = one standard deviation of the mean.

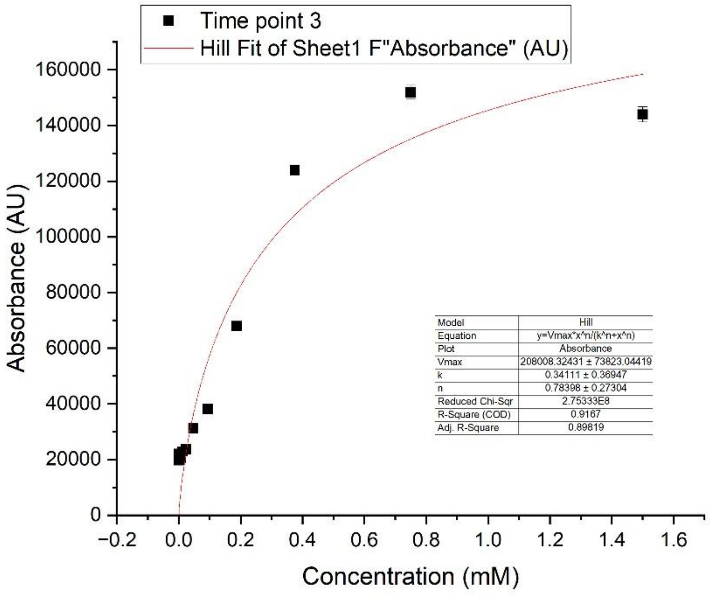

Figure S186 – Graph showing the lysis of calcein loaded PG vesicles at 10 minutes, with respect to increasing concentration of **4**. Data was then fitted to the Hill equation. Average of n= 3, error = one standard deviation of the mean.

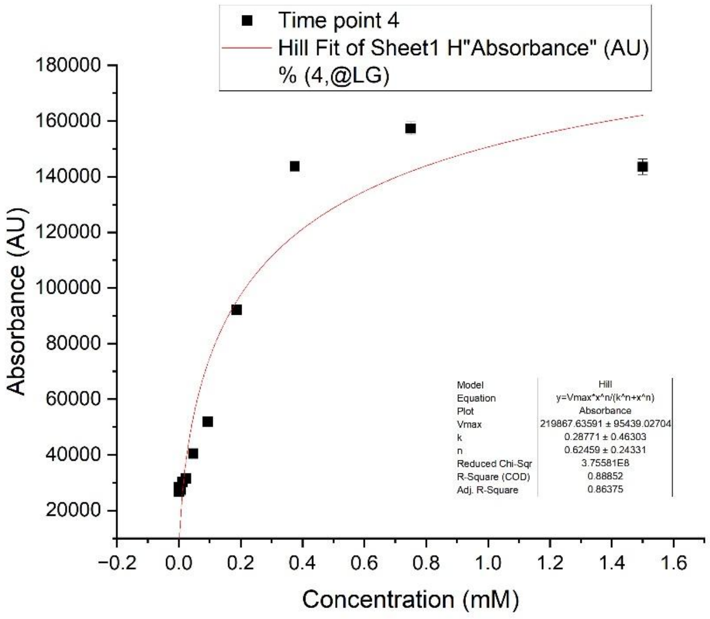

Figure S187 – Graph showing the lysis of calcein loaded PG vesicles at 15 minutes, with respect to increasing concentration of **4**. Data was then fitted to the Hill equation. Average of n= 3, error = one standard deviation of the mean.

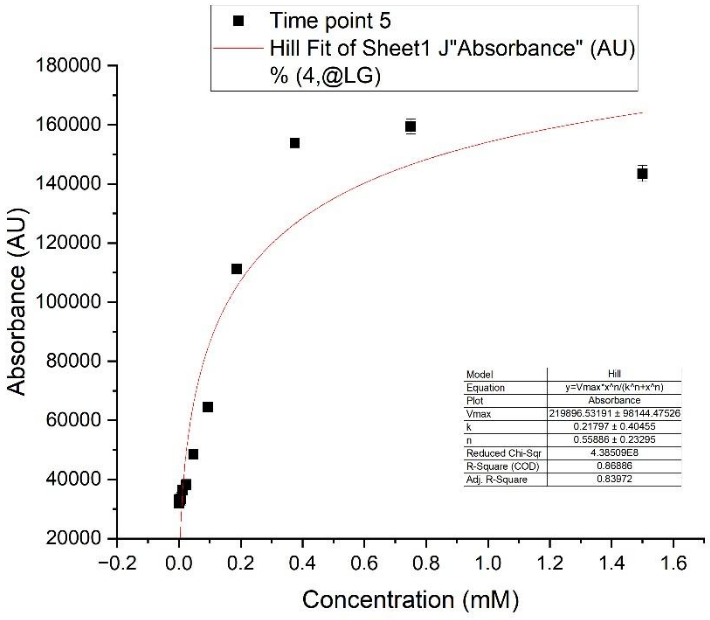

Figure S188 – Graph showing the lysis of calcein loaded PG vesicles at 20 minutes, with respect to increasing concentration of **4**. Data was then fitted to the Hill equation. Average of n= 3, error = one standard deviation of the mean.

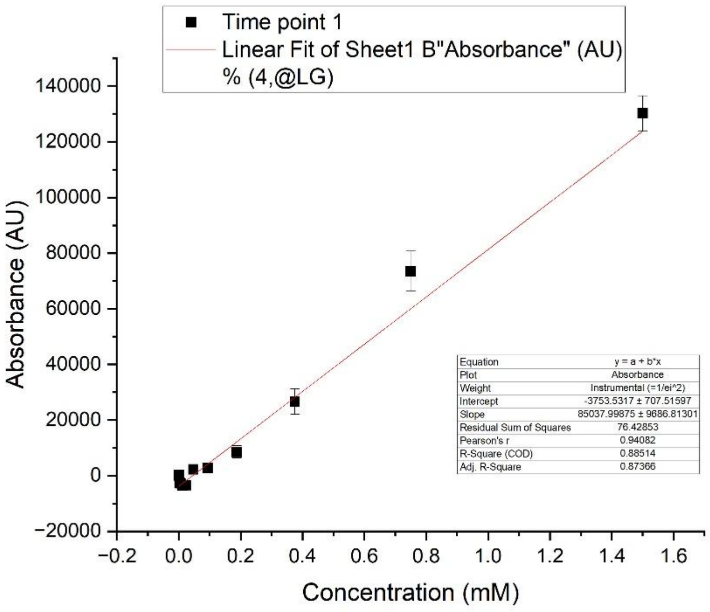

Figure S189 – Graph showing the lysis of calcein loaded PG vesicles at 1 minute, with respect to increasing concentration of **5**. Data was then fitted to a linear line of best fit. Average of n= 3, error = one standard deviation of the mean.

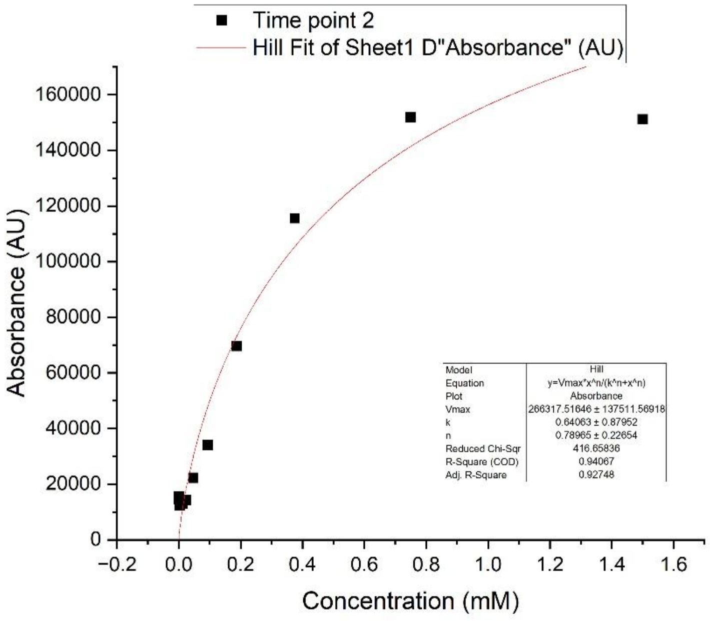

Figure S190 – Graph showing the lysis of calcein loaded PG vesicles at 5 minutes, with respect to increasing concentration of **5**. Data was then fitted to the Hill equation. Average of n= 3, error = one standard deviation of the mean.

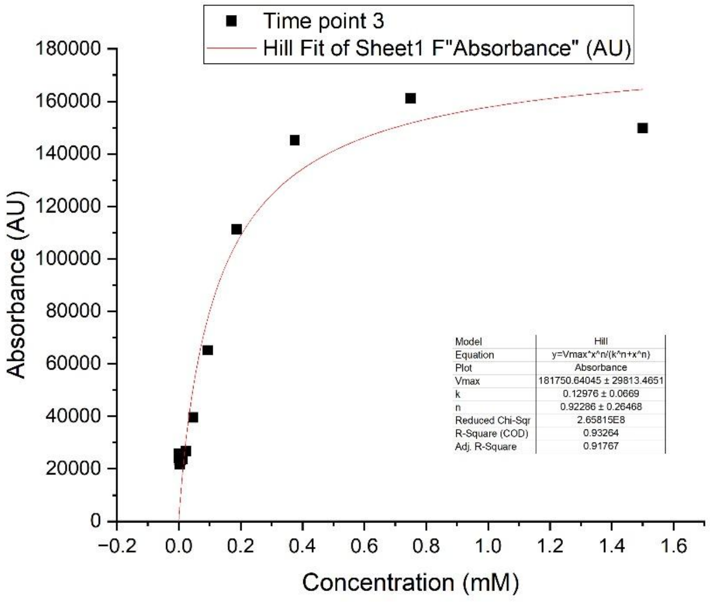

Figure S191 – Graph showing the lysis of calcein loaded PG vesicles at 10 minutes, with respect to increasing concentration of **5**. Data was then fitted to the Hill equation. Average of n= 3, error = one standard deviation of the mean.

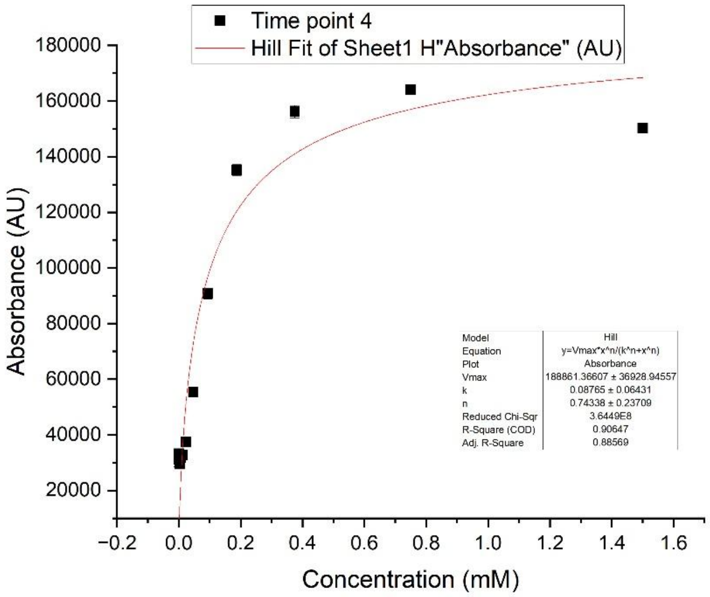

Figure S192 – Graph showing the lysis of calcein loaded PG vesicles at 15 minutes, with respect to increasing concentration of **5**. Data was then fitted to the Hill equation. Average of n= 3, error = one standard deviation of the mean.

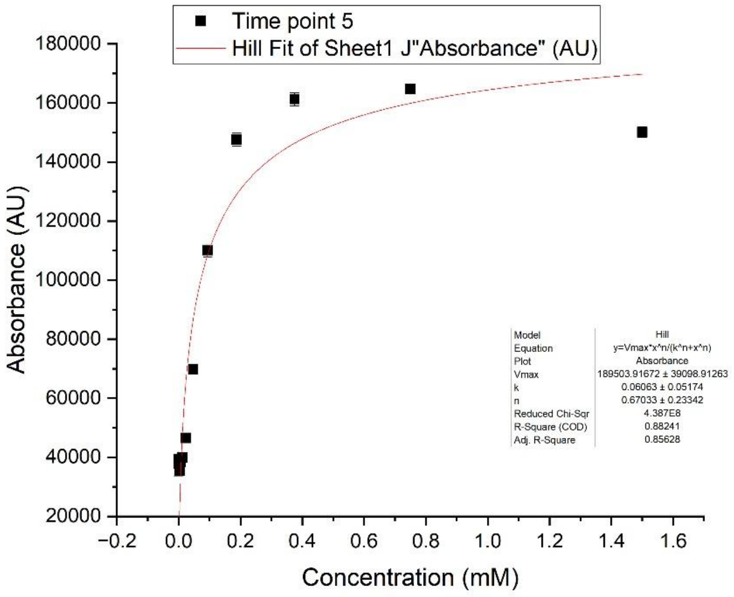

Figure S193 – Graph showing the lysis of calcein loaded PG vesicles at 20 minutes, with respect to increasing concentration of **5**. Data was then fitted to the Hill equation. Average of n= 3, error = one standard deviation of the mean.

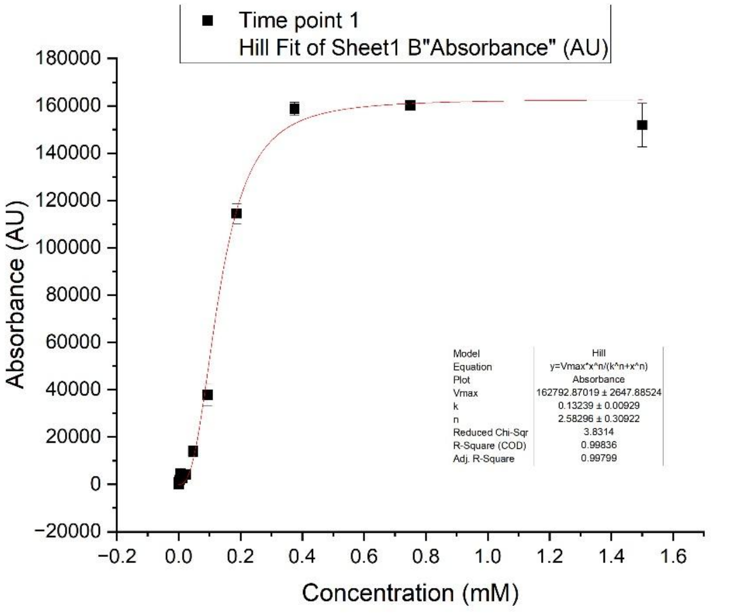

Figure S194 – Graph showing the lysis of calcein loaded PG vesicles at 1 minute, with respect to increasing concentration of **a**. Data was then fitted to the Hill equation. Average of n= 3, error = one standard deviation of the mean.

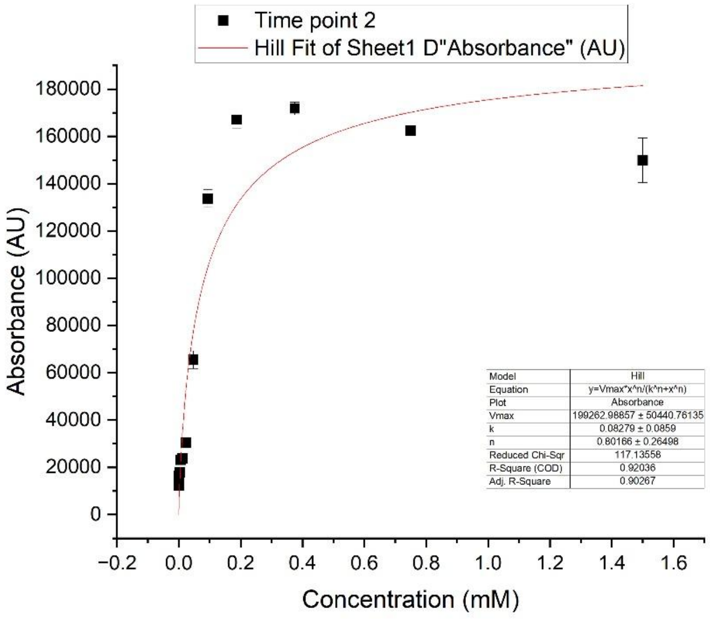

Figure S195 – Graph showing the lysis of calcein loaded PG vesicles at 5 minutes, with respect to increasing concentration of **a**. Data was then fitted to the Hill equation. Average of n= 3, error = one standard deviation of the mean.

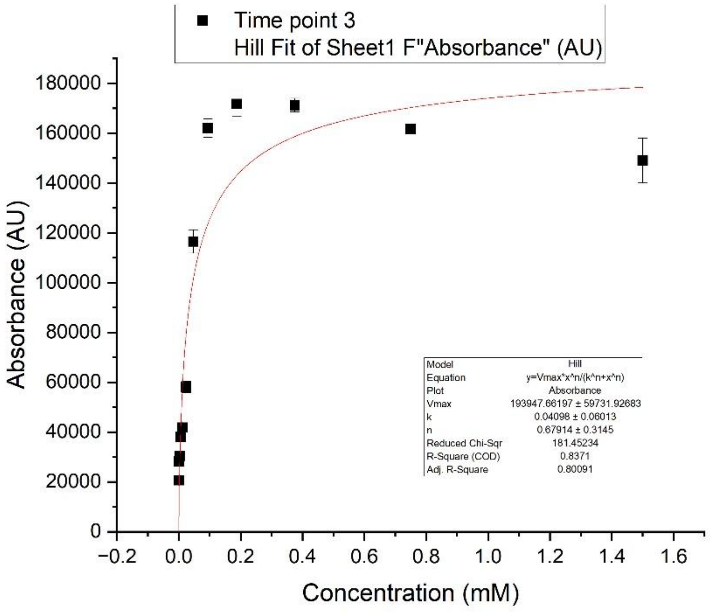

Figure S196 – Graph showing the lysis of calcein loaded PG vesicles at 10 minutes, with respect to increasing concentration of **a**. Data was then fitted to the Hill equation. Average of n= 3, error = one standard deviation of the mean.

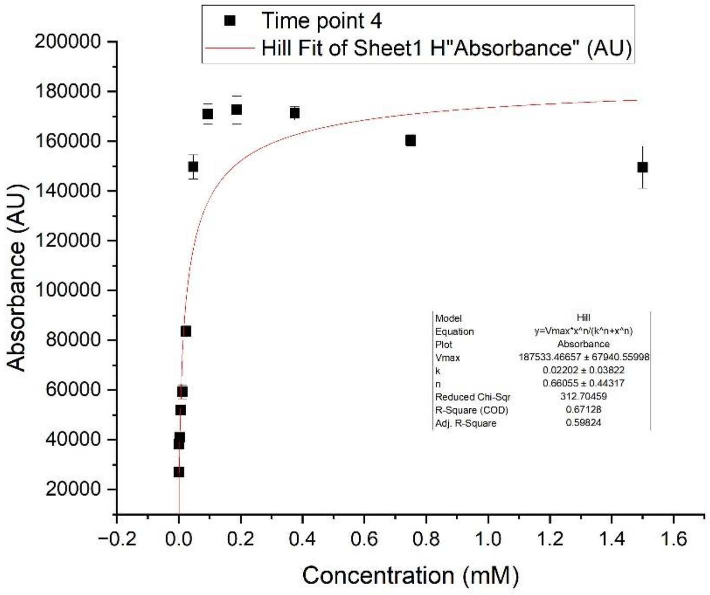

Figure S197 – Graph showing the lysis of calcein loaded PG vesicles at 15 minutes, with respect to increasing concentration of **a**. Data was then fitted to the Hill equation. Average of n= 3, error = one standard deviation of the mean.

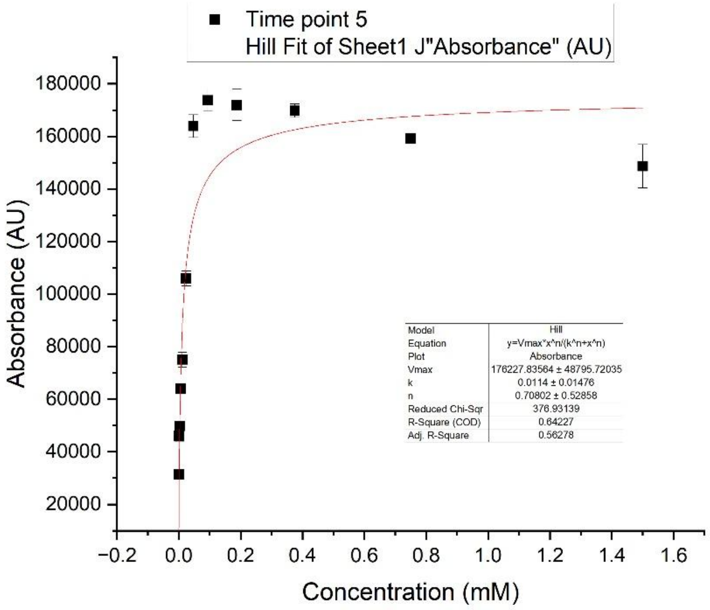

Figure S198 – Graph showing the lysis of calcein loaded PG vesicles at 20 minutes, with respect to increasing concentration of **a**. Data was then fitted to the Hill equation. Average of n= 3, error = one standard deviation of the mean.

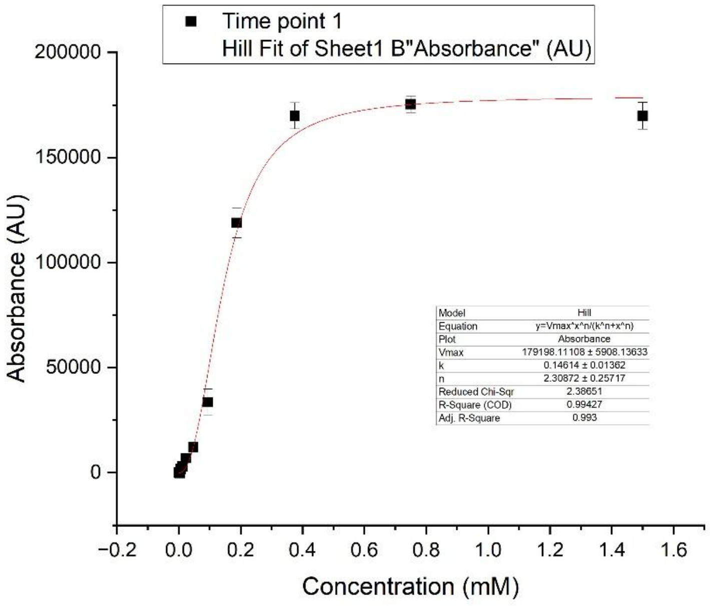

Figure S199 – Graph showing the lysis of calcein loaded PG vesicles at 1 minute, with respect to increasing concentration of **b**. Data was then fitted to the Hill equation. Average of n= 3, error = one standard deviation of the mean.

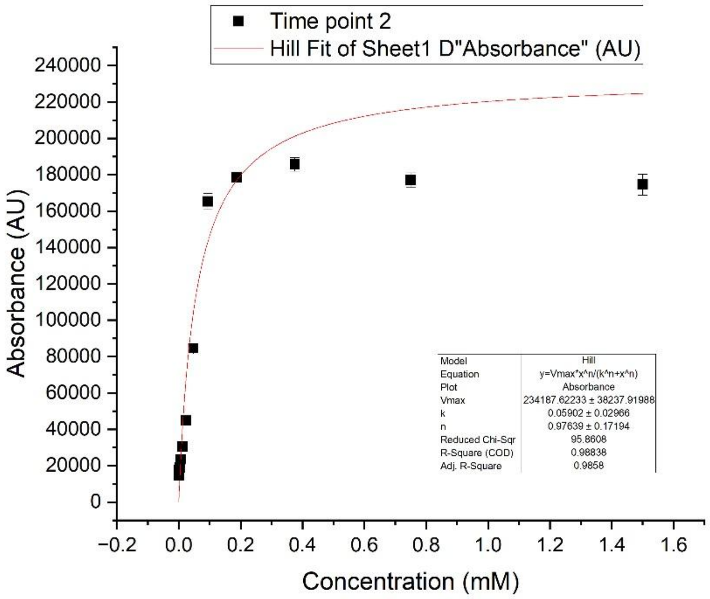

Figure S200 – Graph showing the lysis of calcein loaded PG vesicles at 5 minutes, with respect to increasing concentration of **b**. Data was then fitted to the Hill equation. Average of n= 3, error = one standard deviation of the mean.

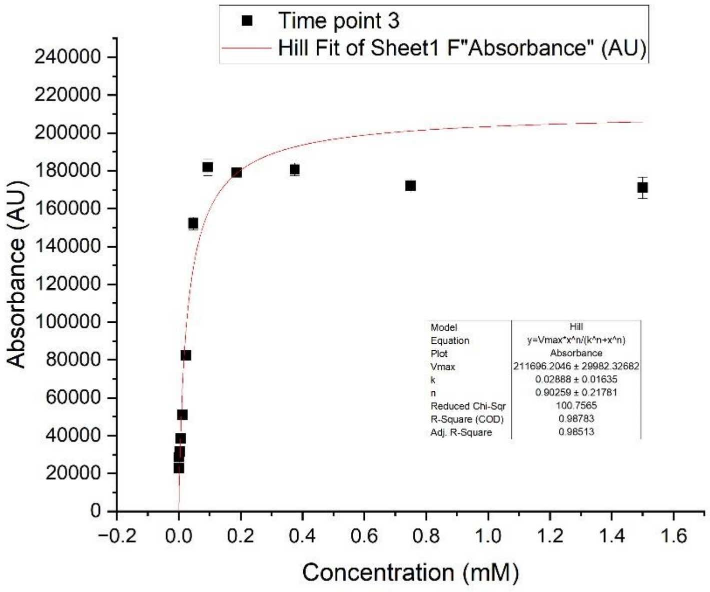

Figure S201 – Graph showing the lysis of calcein loaded PG vesicles at 10 minutes, with respect to increasing concentration of **b**. Data was then fitted to the Hill equation. Average of n= 3, error = one standard deviation of the mean.

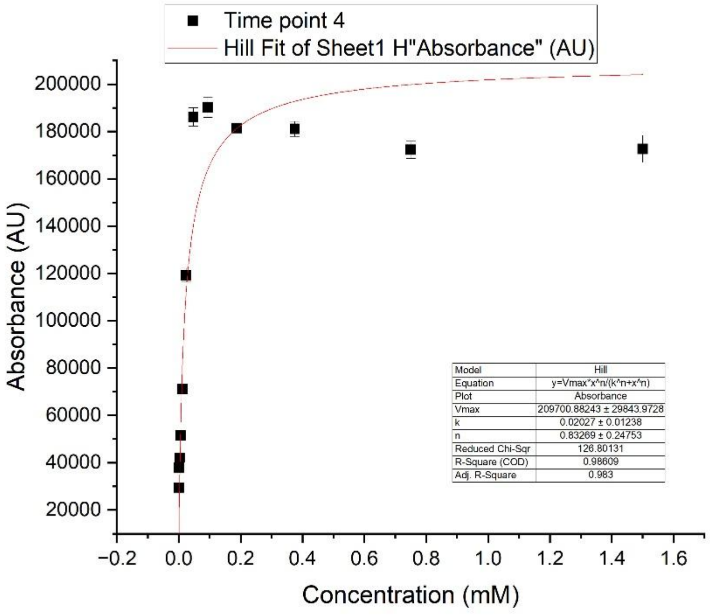

Figure S202 – Graph showing the lysis of calcein loaded PG vesicles at 15 minutes, with respect to increasing concentration of **b**. Data was then fitted to the Hill equation. Average of n= 3, error = one standard deviation of the mean.

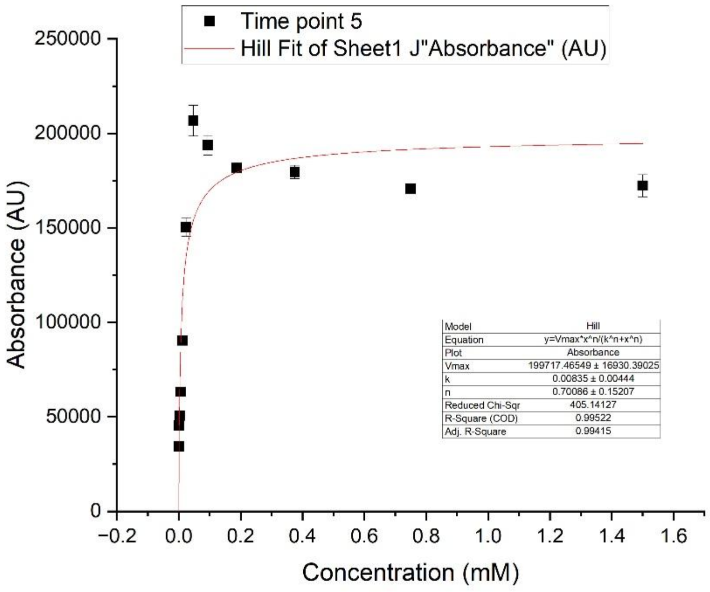

Figure S203 – Graph showing the lysis of calcein loaded PG vesicles at 20 minutes, with respect to increasing concentration of **b**. Data was then fitted to the Hill equation. Average of n= 3, error = one standard deviation of the mean.

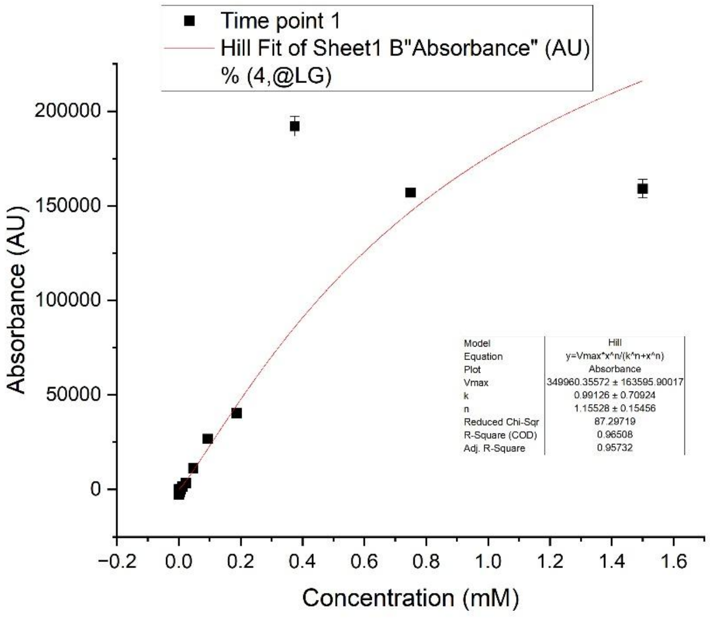

Figure S204 – Graph showing the lysis of calcein loaded PG vesicles at 1 minute, with respect to increasing concentration of **c**. Data was then fitted to the Hill equation. Average of n= 3, error = one standard deviation of the mean.

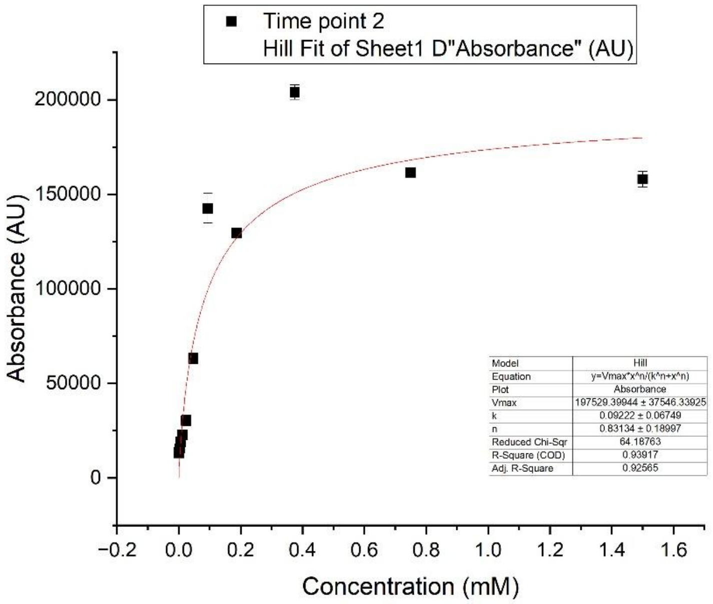

Figure S205 – Graph showing the lysis of calcein loaded PG vesicles at 5 minutes, with respect to increasing concentration of **c**. Data was then fitted to the Hill equation. Average of n= 3, error = one standard deviation of the mean.

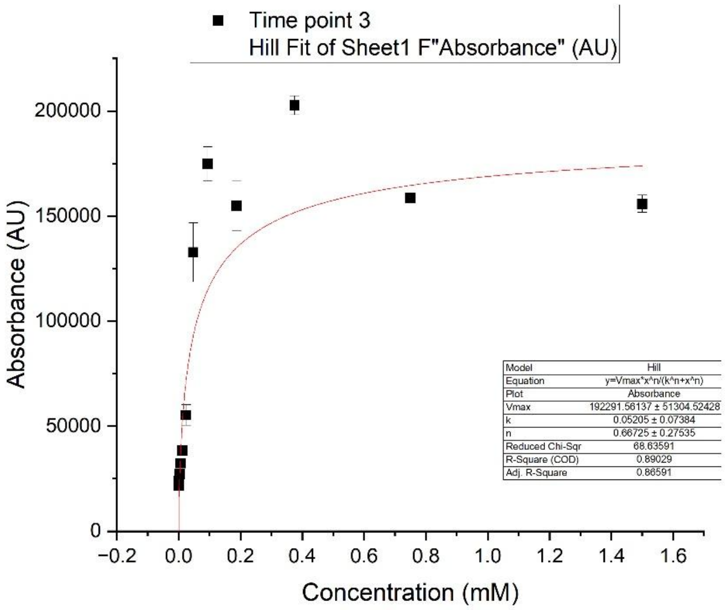

Figure S206 – Graph showing the lysis of calcein loaded PG vesicles at 10 minutes, with respect to increasing concentration of **c**. Data was then fitted to the Hill equation. Average of n= 3, error = one standard deviation of the mean.

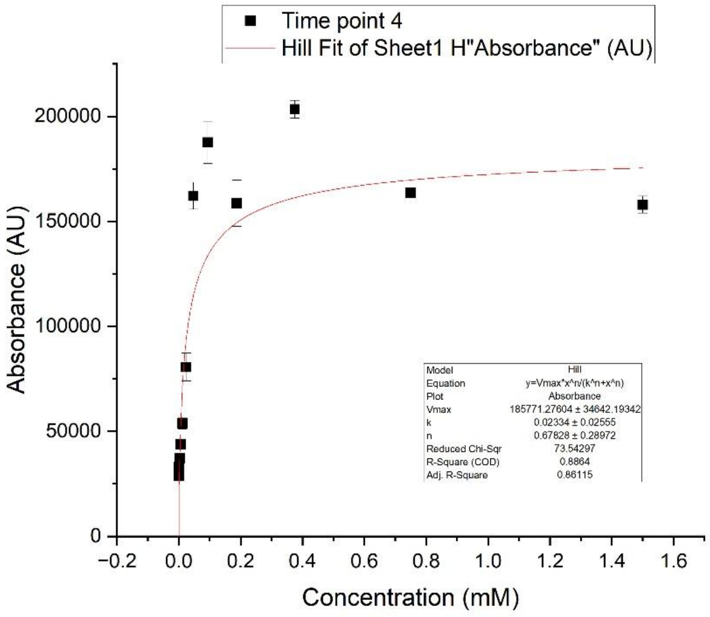

Figure S207 – Graph showing the lysis of calcein loaded PG vesicles at 15 minutes, with respect to increasing concentration of **c**. Data was then fitted to the Hill equation. Average of n= 3, error = one standard deviation of the mean.

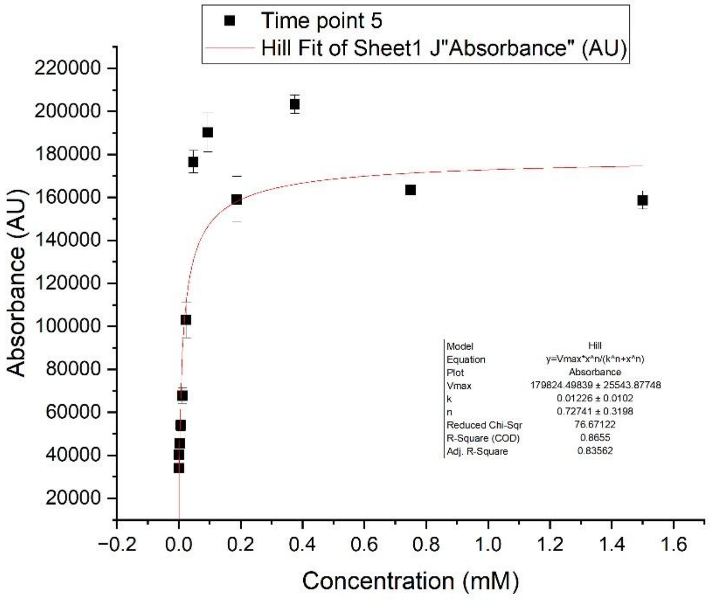

Figure S208 – Graph showing the lysis of calcein loaded PG vesicles at 20 minutes, with respect to increasing concentration of **c**. Data was then fitted to the Hill equation. Average of n= 3, error = one standard deviation of the mean.

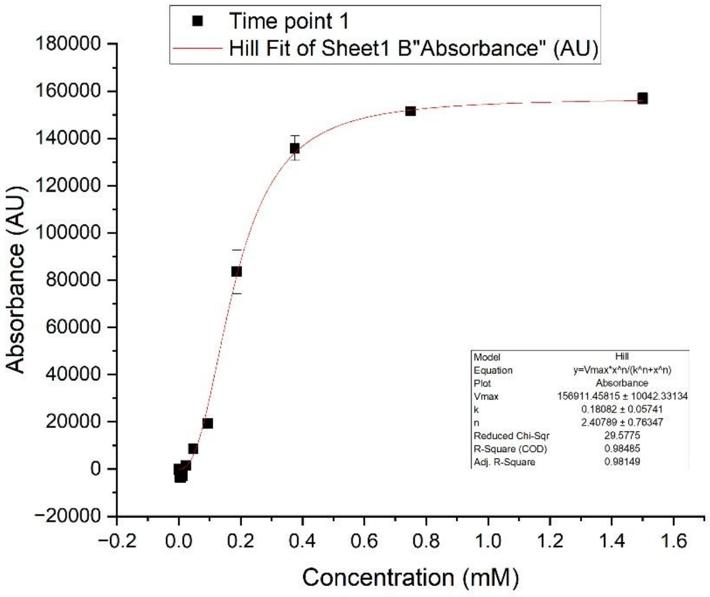

Figure S209 – Graph showing the lysis of calcein loaded PG vesicles at 1 minute, with respect to increasing concentration of **d**. Data was then fitted to the Hill equation. Average of n= 3, error = one standard deviation of the mean.

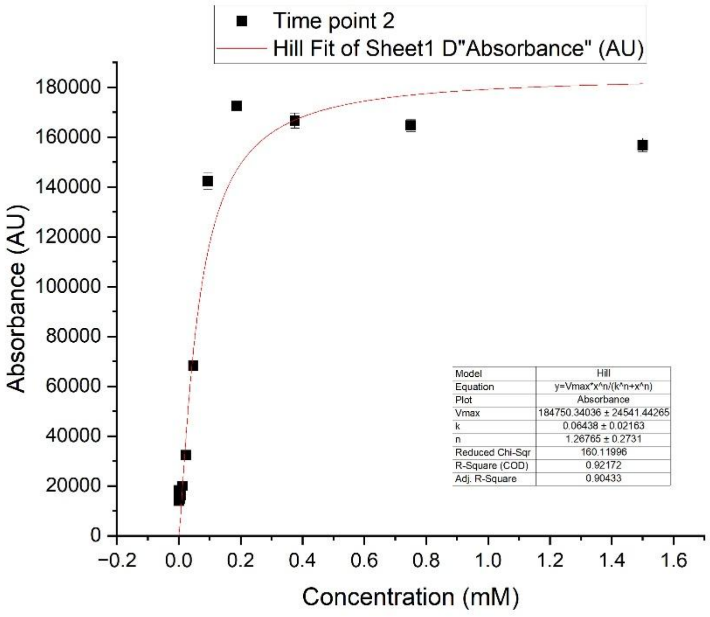

Figure S210 – Graph showing the lysis of calcein loaded PG vesicles at 5 minutes, with respect to increasing concentration of **d**. Data was then fitted to the Hill equation. Average of n= 3, error = one standard deviation of the mean.

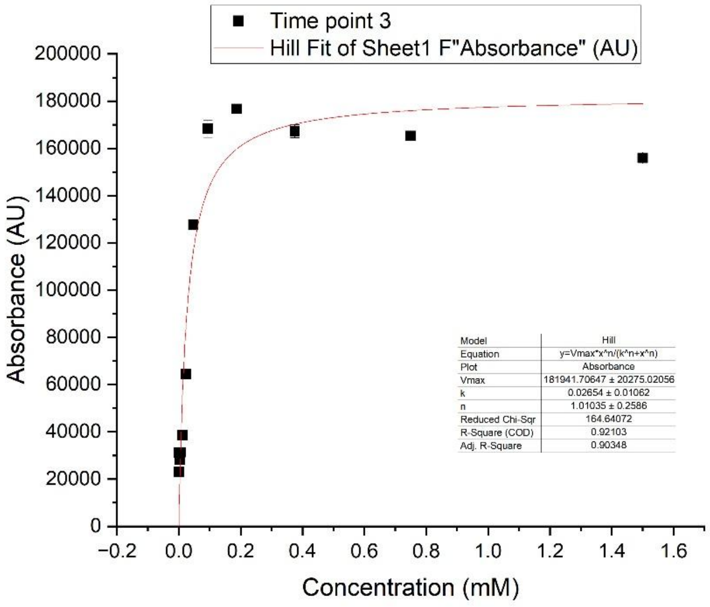

Figure S211 – Graph showing the lysis of calcein loaded PG vesicles at 10 minutes, with respect to increasing concentration of **d**. Data was then fitted to the Hill equation. Average of n= 3, error = one standard deviation of the mean.

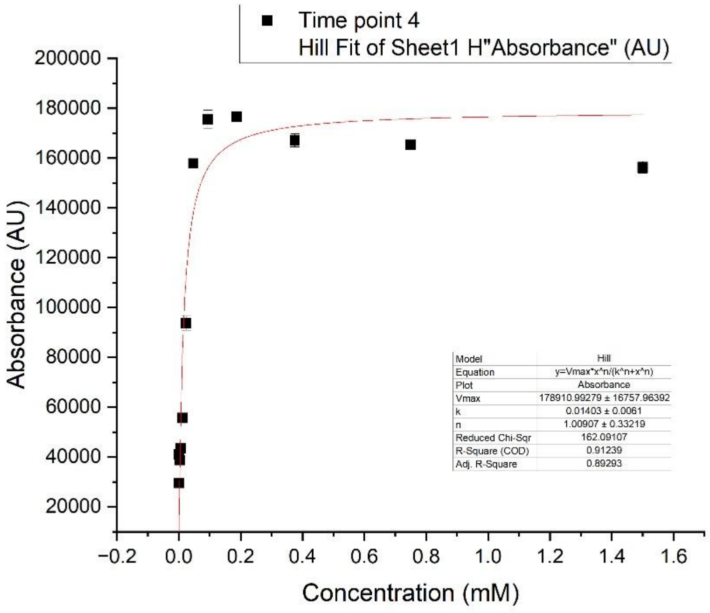

Figure S212 – Graph showing the lysis of calcein loaded PG vesicles at 15 minutes, with respect to increasing concentration of **d**. Data was then fitted to the Hill equation. Average of n= 3, error = one standard deviation of the mean.

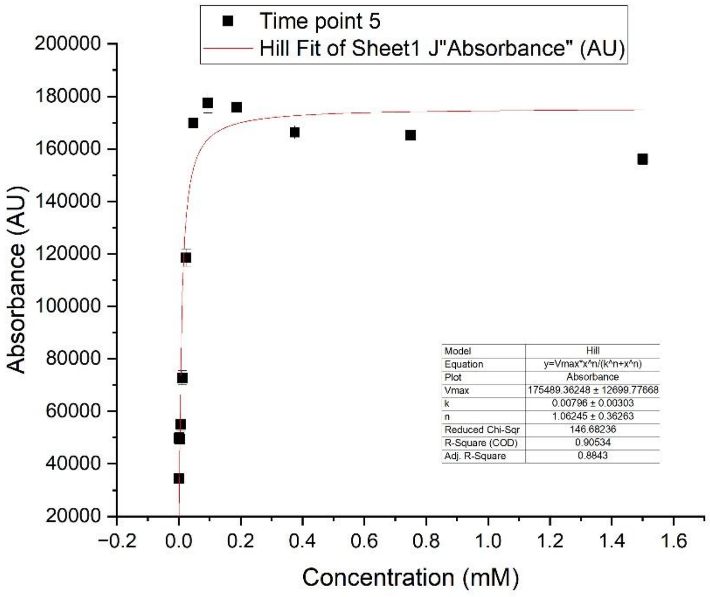

Figure S213 – Graph showing the lysis of calcein loaded PG vesicles at 20 minutes, with respect to increasing concentration of **d**. Data was then fitted to the Hill equation. Average of n= 3, error = one standard deviation of the mean.

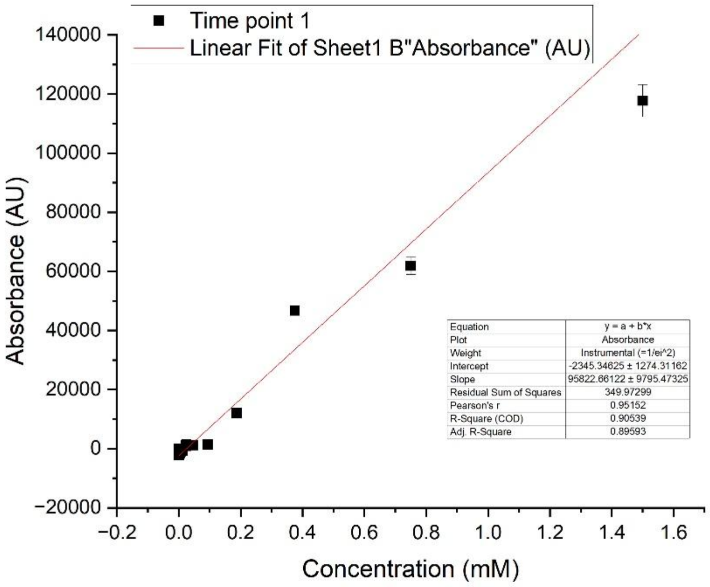

Figure S214 – Graph showing the lysis of calcein loaded PG vesicles at 1 minute, with respect to increasing concentration of **e**. Data was then fitted to a linear line of best fit. Average of n= 3, error = one standard deviation of the mean.

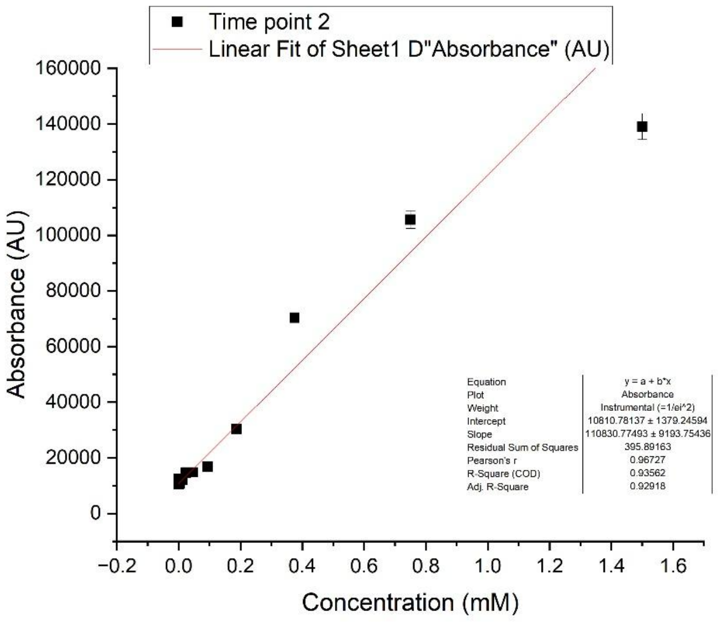

Figure S215 – Graph showing the lysis of calcein loaded PG vesicles at 5 minutes, with respect to increasing concentration of **e**. Data was then fitted to a linear line of best fit. Average of n= 3, error = one standard deviation of the mean.

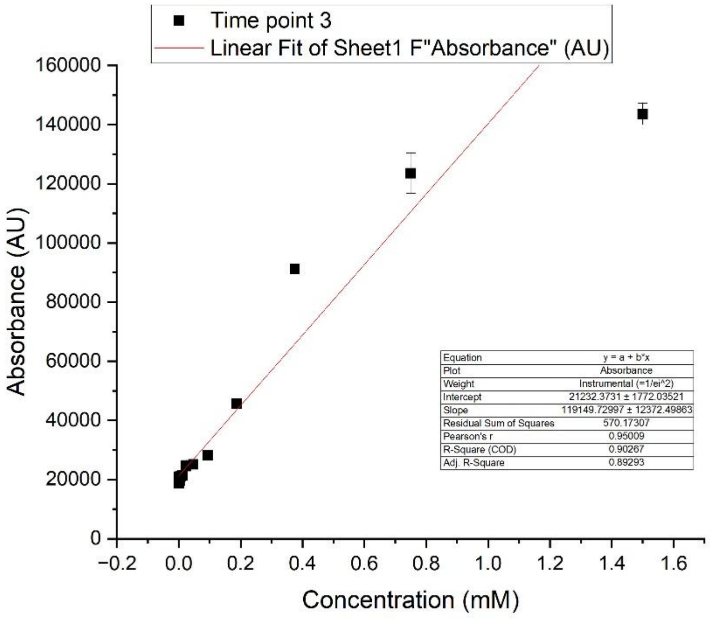

Figure S216 – Graph showing the lysis of calcein loaded PG vesicles at 5 minutes, with respect to increasing concentration of **e**. Data was then fitted to a linear line of best fit. Average of n= 3, error = one standard deviation of the mean.

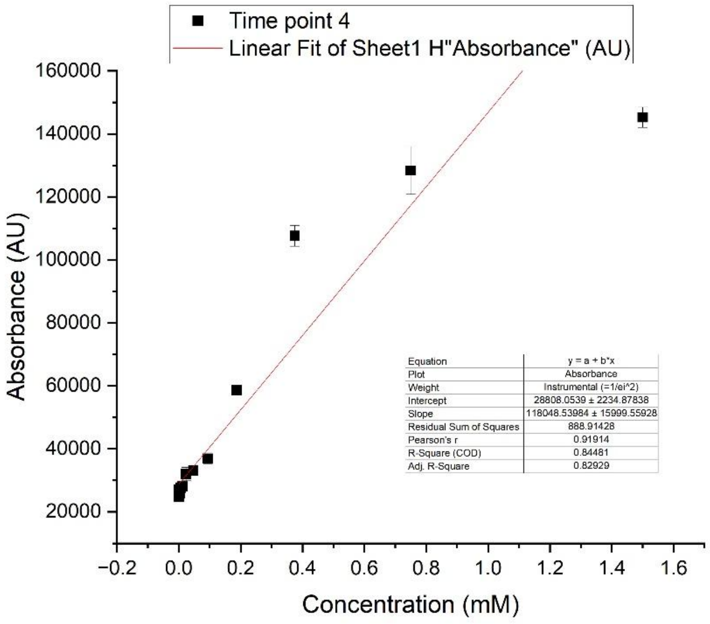

Figure S217 – Graph showing the lysis of calcein loaded PG vesicles at 15 minutes, with respect to increasing concentration of **e**. Data was then fitted to a linear line of best fit. Average of n= 3, error = one standard deviation of the mean.

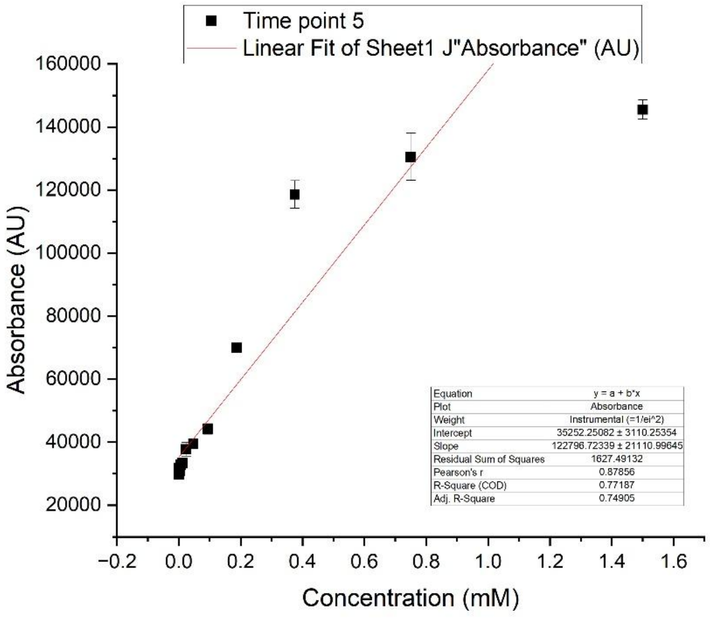

Figure S218 – Graph showing the lysis of calcein loaded PG vesicles at 20 minutes, with respect to increasing concentration of **e**. Data was then fitted to a linear line of best fit. Average of n= 3, error = one standard deviation of the mean.

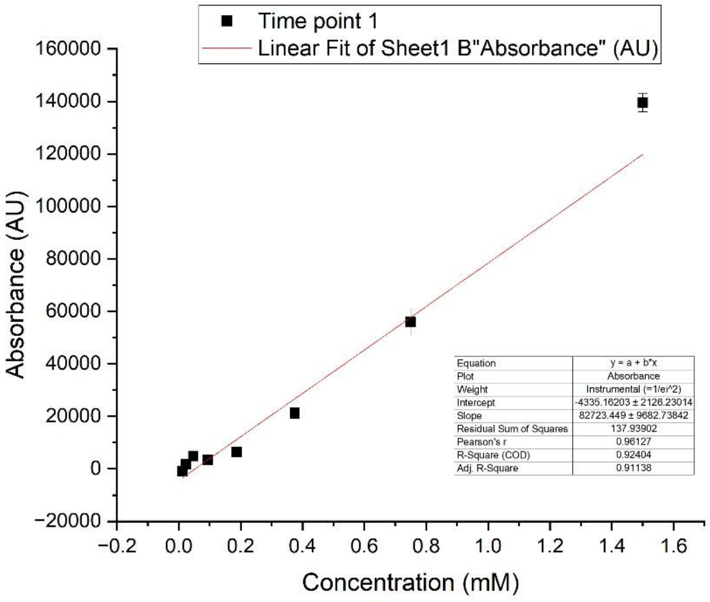

Figure S219 – Graph showing the lysis of calcein loaded PG vesicles at 1 minute, with respect to increasing concentration of **f**. Data was then fitted to a linear line of best fit. Average of n= 3, error = one standard deviation of the mean.

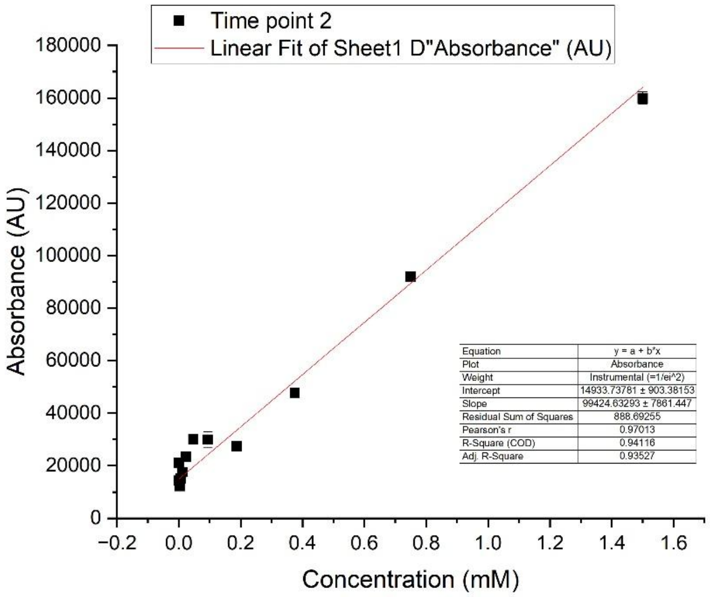

Figure S220 – Graph showing the lysis of calcein loaded PG vesicles at 5 minutes, with respect to increasing concentration of **f**. Data was then fitted to a linear line of best fit. Average of n= 3, error = one standard deviation of the mean.

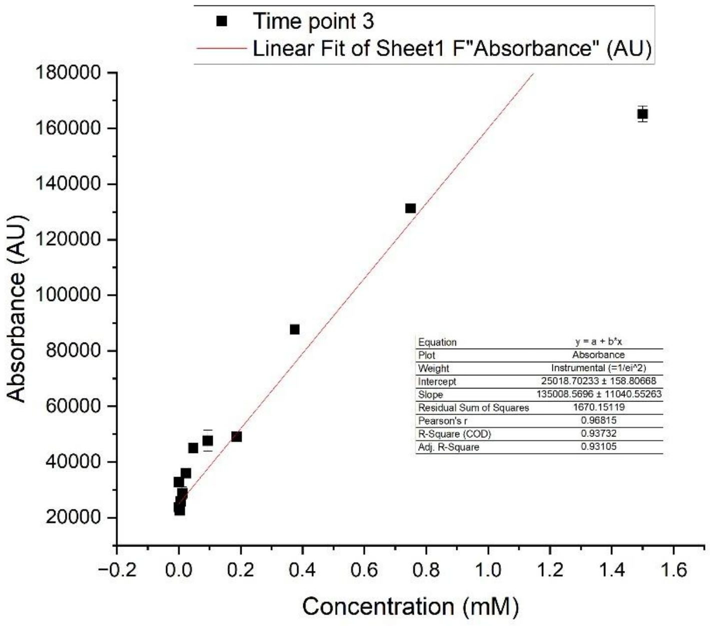

Figure S221 – Graph showing the lysis of calcein loaded PG vesicles at 10 minutes, with respect to increasing concentration of **f**. Data was then fitted to a linear line of best fit. Average of n= 3, error = one standard deviation of the mean.

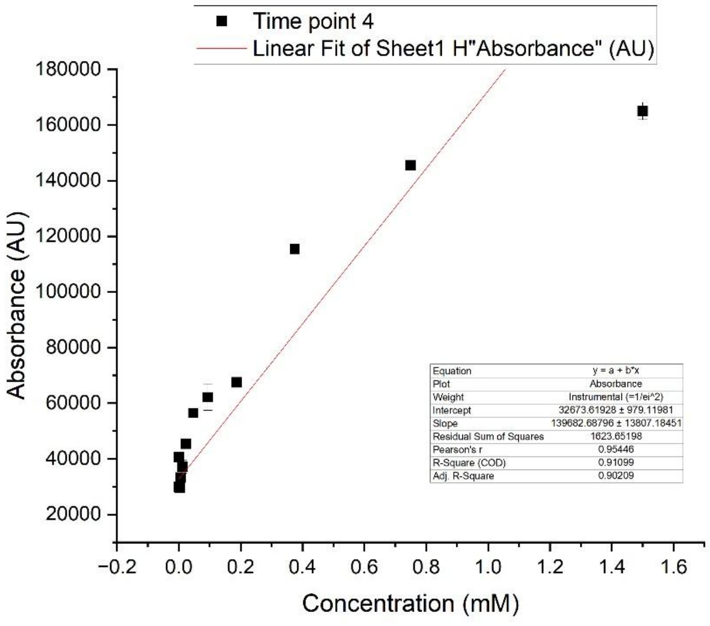

Figure S222 – Graph showing the lysis of calcein loaded PG vesicles at 15 minutes, with respect to increasing concentration of **f**. Data was then fitted to a linear line of best fit. Average of n= 3, error = one standard deviation of the mean.

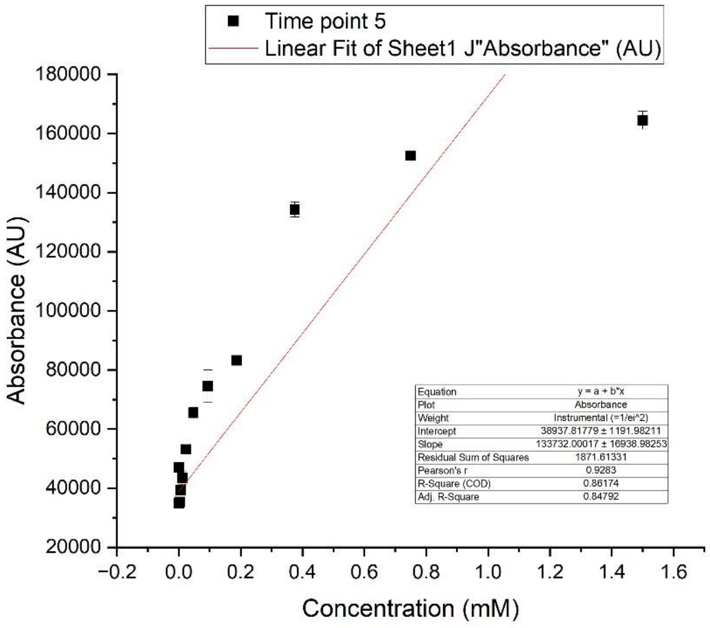

Figure S223 – Graph showing the lysis of calcein loaded PG vesicles at 20 minutes, with respect to increasing concentration of **f**. Data was then fitted to a linear line of best fit. Average of n= 3, error = one standard deviation of the mean.

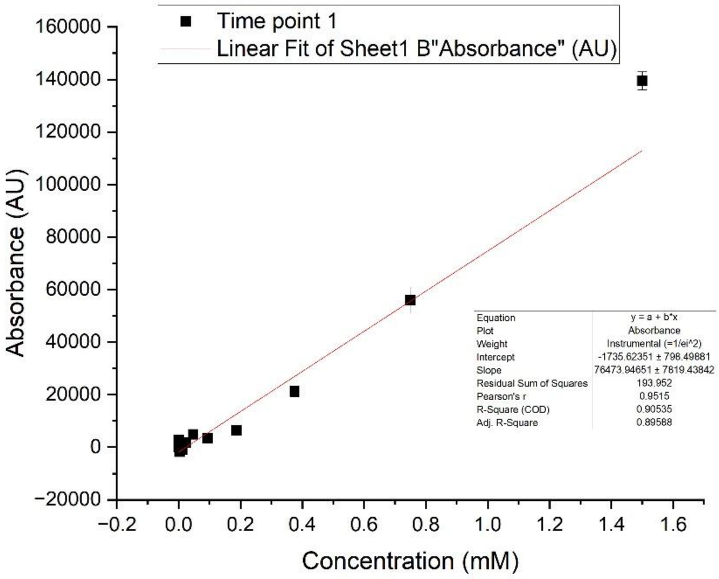

Figure S224 – Graph showing the lysis of calcein loaded PG vesicles at 1 minute, with respect to increasing concentration of **g**. Data was then fitted to a linear line of best fit. Average of n= 3, error = one standard deviation of the mean.

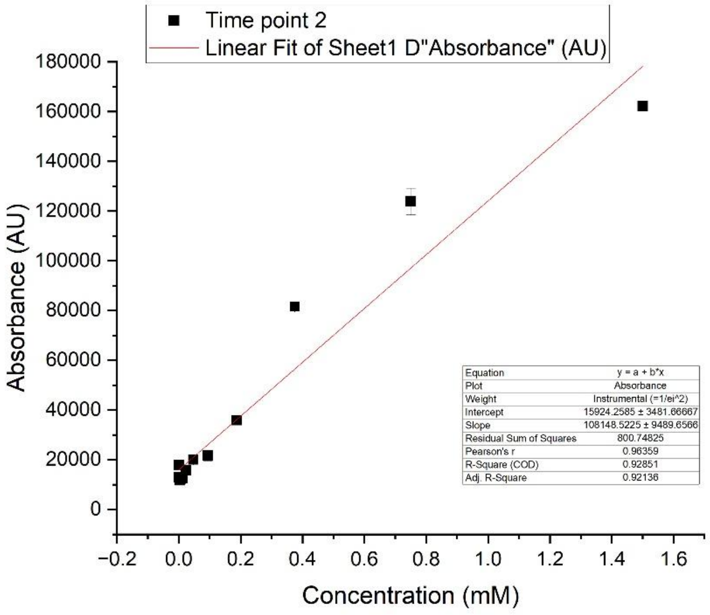

Figure S225 – Graph showing the lysis of calcein loaded PG vesicles at 5 minutes, with respect to increasing concentration of **g**. Data was then fitted to a linear line of best fit. Average of n= 3, error = one standard deviation of the mean.

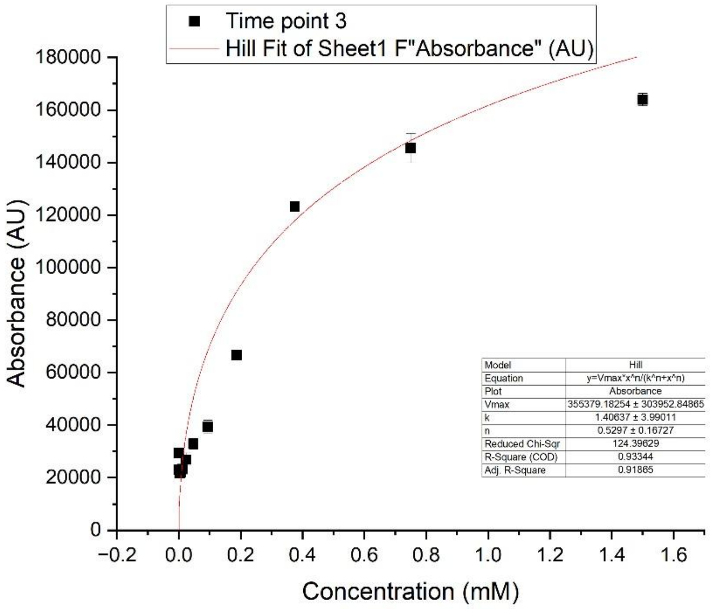

Figure S226 – Graph showing the lysis of calcein loaded PG vesicles at 10 minutes, with respect to increasing concentration of **g**. Data was then fitted to the Hill equation. Average of n= 3, error = one standard deviation of the mean.

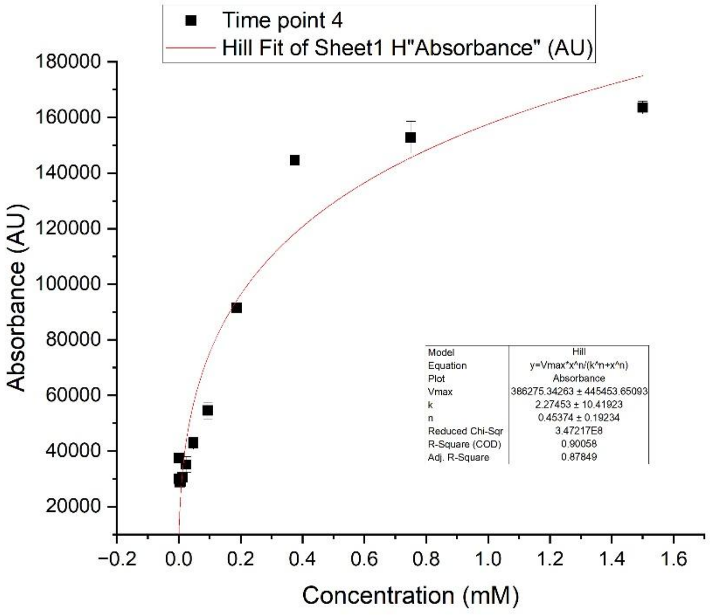

Figure S227 – Graph showing the lysis of calcein loaded PG vesicles at 15 minutes, with respect to increasing concentration of **g**. Data was then fitted to the Hill equation. Average of n= 3, error = one standard deviation of the mean.

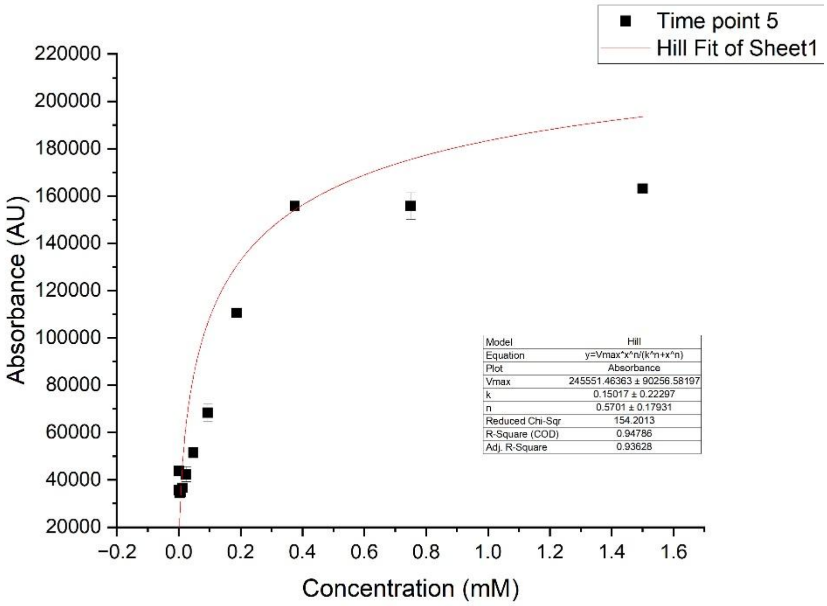

Figure S228 – Graph showing the lysis of calcein loaded PG vesicles at 20 minutes, with respect to increasing concentration of **g**. Data was then fitted to the Hill equation. Average of n= 3, error = one standard deviation of the mean.

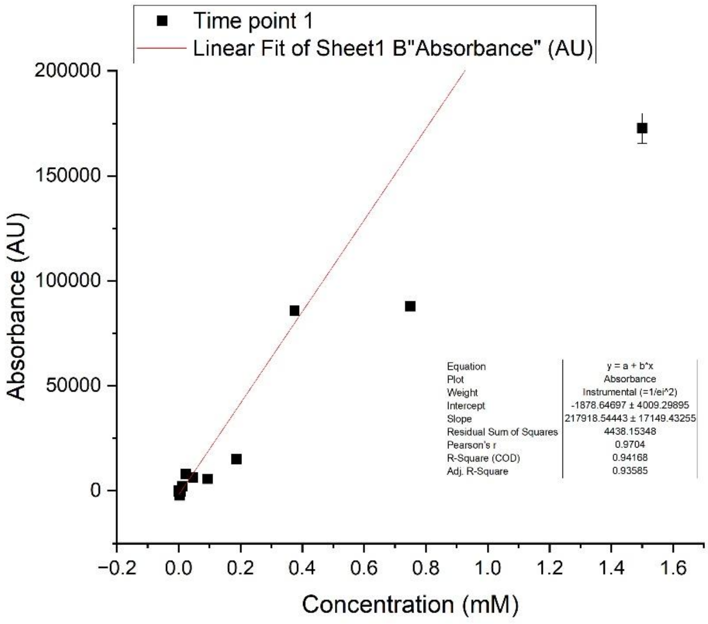

Figure S229 – Graph showing the lysis of calcein loaded PG vesicles at 1 minute, with respect to increasing concentration of **h**. Data was then fitted to a linear line of best fit. Average of n= 3, error = one standard deviation of the mean.

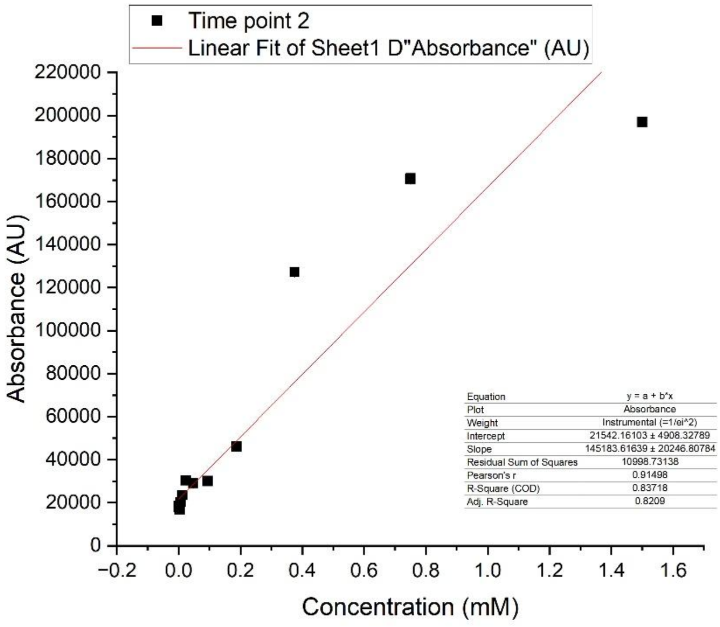

Figure S230 – Graph showing the lysis of calcein loaded PG vesicles at 5 minutes, with respect to increasing concentration of **h**. Data was then fitted to a linear line of best fit. Average of n= 3, error = one standard deviation of the mean.

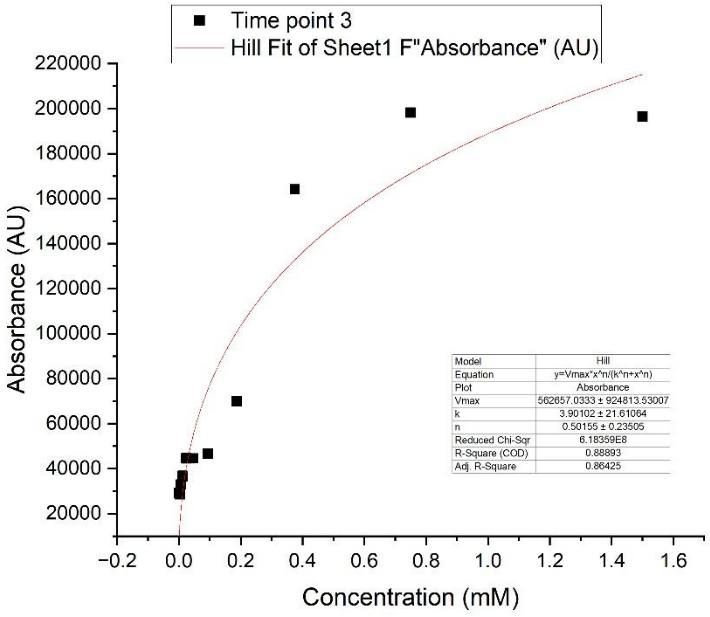

Figure S231 – Graph showing the lysis of calcein loaded PG vesicles at 10 minutes, with respect to increasing concentration of **h**. Data was then fitted to the Hill equation. Average of n= 3, error = one standard deviation of the mean.

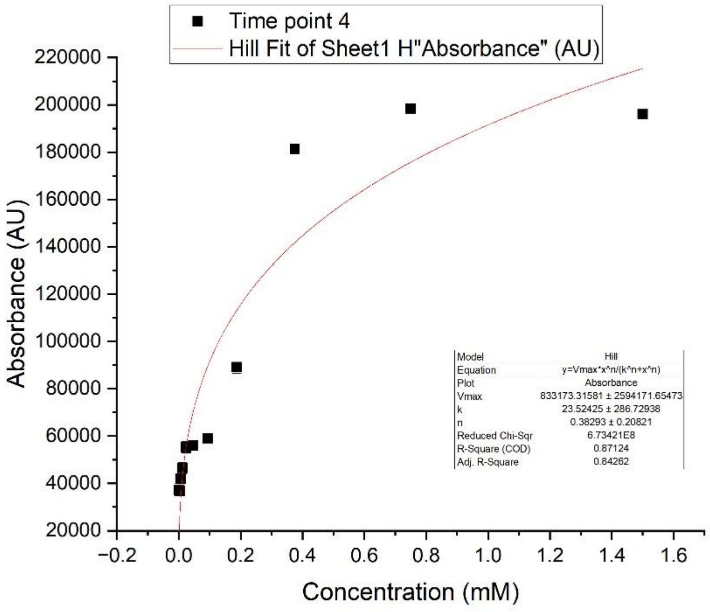

Figure S232 – Graph showing the lysis of calcein loaded PG vesicles at 15 minutes, with respect to increasing concentration of **h**. Data was then fitted to the Hill equation. Average of n= 3, error = one standard deviation of the mean.

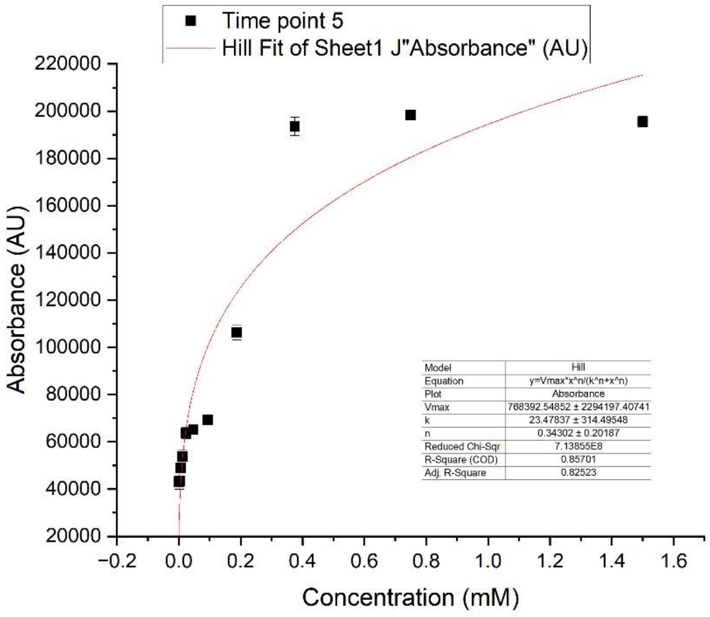

Figure S233 – Graph showing the lysis of calcein loaded PG vesicles at 20 minutes, with respect to increasing concentration of **h**. Data was then fitted to the Hill equation. Average of n= 3, error = one standard deviation of the mean.

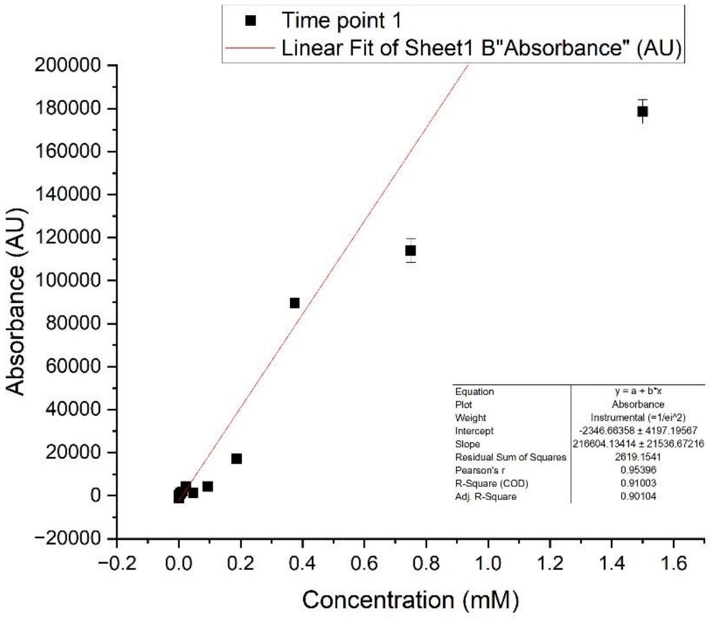

Figure S234 – Graph showing the lysis of calcein loaded PG vesicles at 1 minute, with respect to increasing concentration of **i**. Data was then fitted to a linear line of best fit. Average of n= 3, error = one standard deviation of the mean.

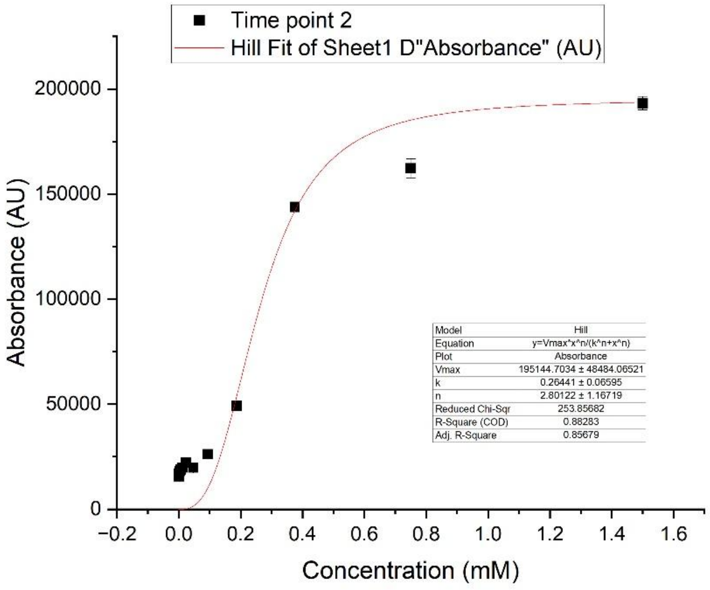

Figure S235 – Graph showing the lysis of calcein loaded PG vesicles at 5 minutes, with respect to increasing concentration of **i**. Data was then fitted to the Hill equation. Average of n= 3, error = one standard deviation of the mean.

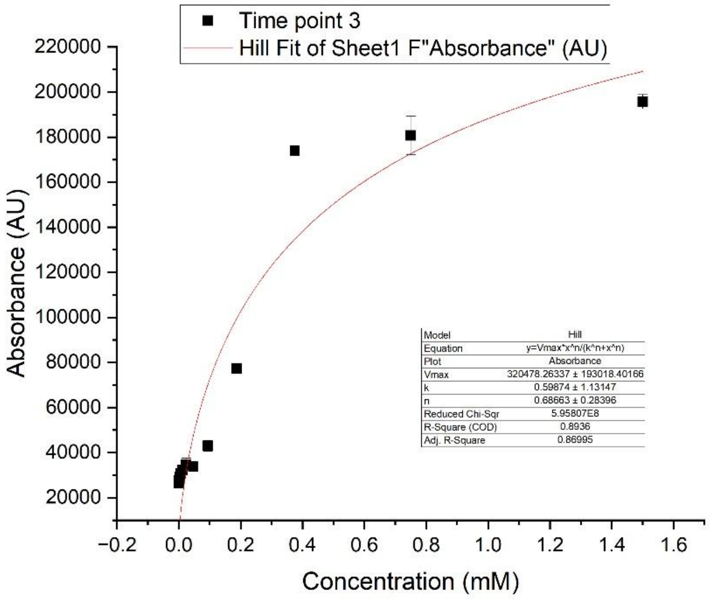

Figure S236 – Graph showing the lysis of calcein loaded PG vesicles at 10 minutes, with respect to increasing concentration of **i**. Data was then fitted to the Hill equation. Average of n= 3, error = one standard deviation of the mean.

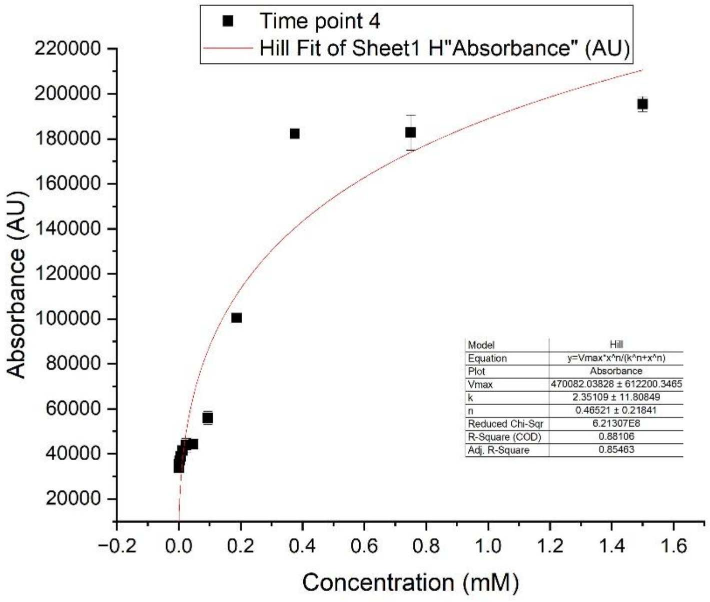

Figure S237 – Graph showing the lysis of calcein loaded PG vesicles at 15 minutes, with respect to increasing concentration of **i**. Data was then fitted to the Hill equation. Average of n= 3, error = one standard deviation of the mean.

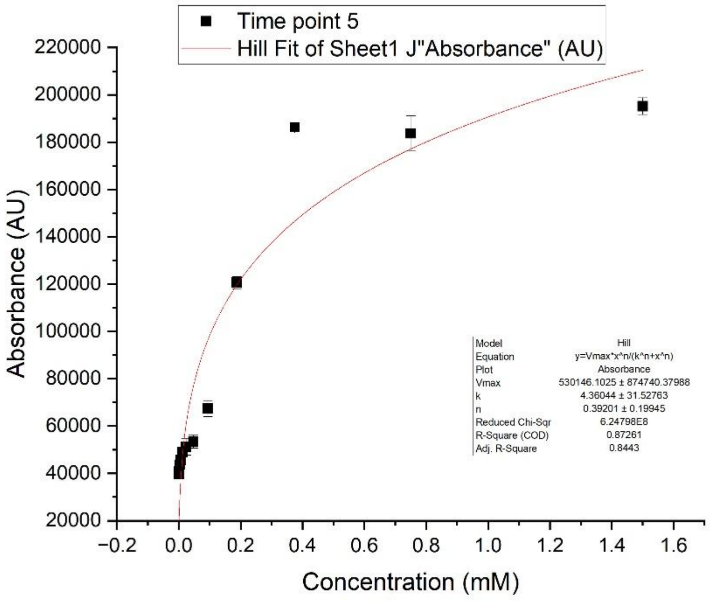

Figure S238 – Graph showing the lysis of calcein loaded PG vesicles at 20 minutes, with respect to increasing concentration of **i**. Data was then fitted to the Hill equation. Average of n= 3, error = one standard deviation of the mean.

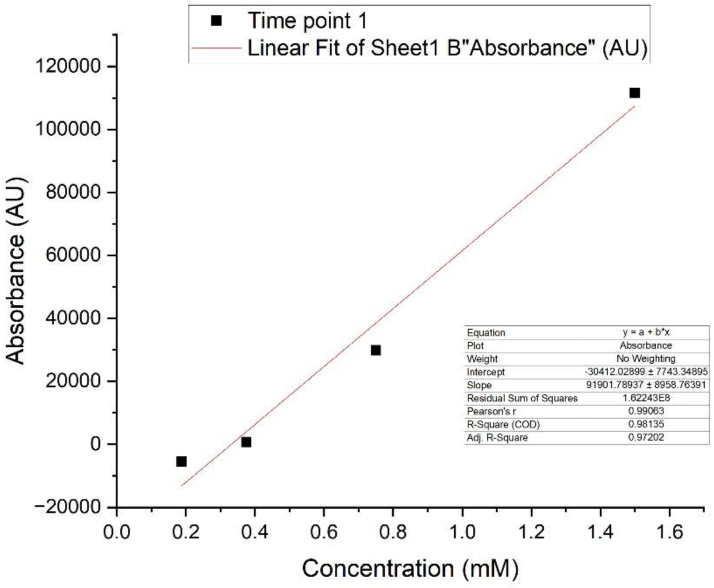

Figure S239 – Graph showing the lysis of calcein loaded PG vesicles at 1 minute, with respect to increasing concentration of **j**. Data was then fitted to a linear line of best fit. Average of n= 3, error = one standard deviation of the mean.

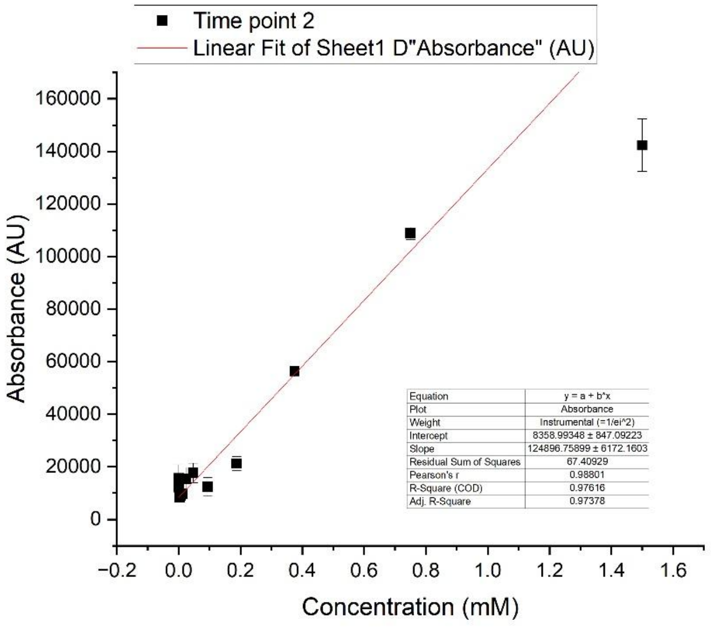

Figure S240 – Graph showing the lysis of calcein loaded PG vesicles at 5 minutes, with respect to increasing concentration of **j**. Data was then fitted to a linear line of best fit. Average of n= 3, error = one standard deviation of the mean.

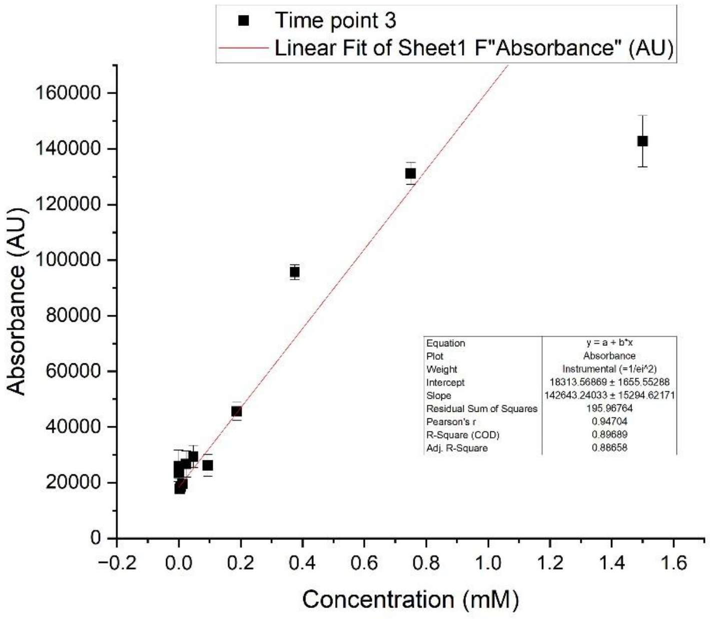

Figure S241 – Graph showing the lysis of calcein loaded PG vesicles at 10 minutes, with respect to increasing concentration of **j**. Data was then fitted to a linear line of best fit. Average of n= 3, error = one standard deviation of the mean.

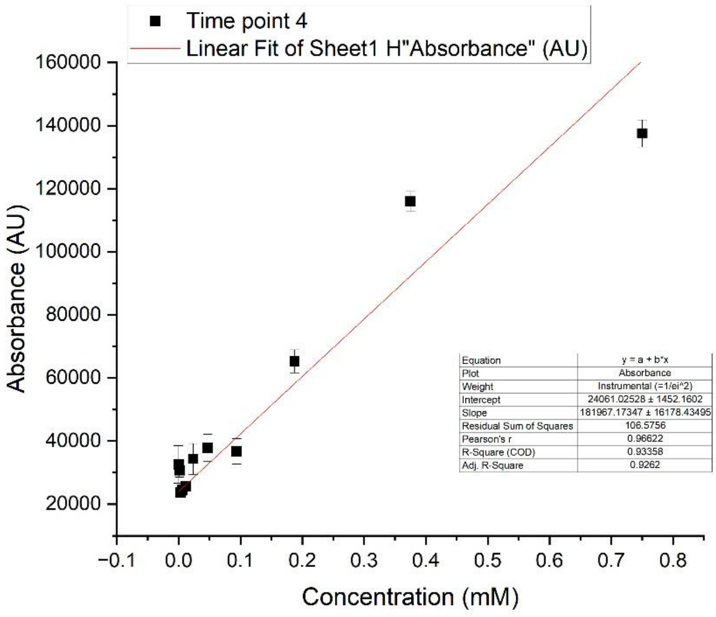

Figure S242 – Graph showing the lysis of calcein loaded PG vesicles at 15 minutes, with respect to increasing concentration of **j**. Data was then fitted to a linear line of best fit. Average of n= 3, error = one standard deviation of the mean.

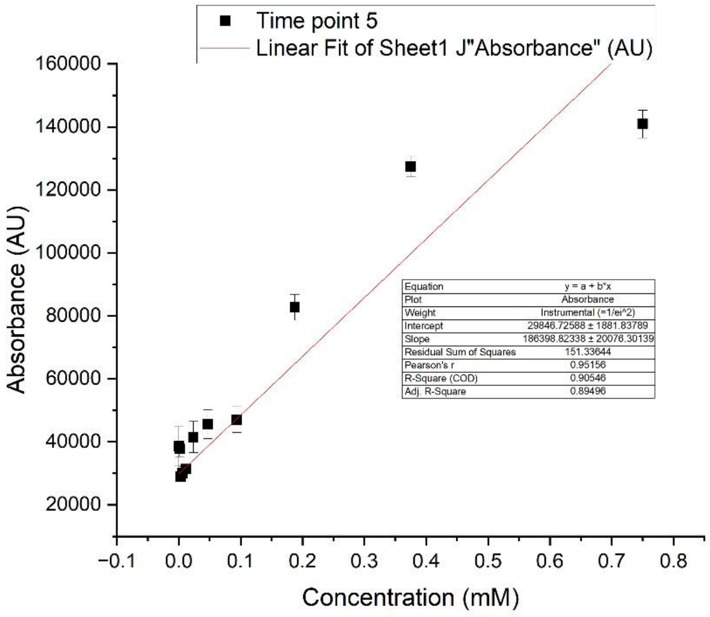

Figure S243 – Graph showing the lysis of calcein loaded PG vesicles at 20 minutes, with respect to increasing concentration of **j**. Data was then fitted to a linear line of best fit. Average of n= 3, error = one standard deviation of the mean.

PG vesicle lysis titration summary

Table S12 – Summary of EC_{50}, R^2 and *n* values determined through fitting calcein filled PG vesicles to the Hill equation. If a fit to the Hill equation could not be obtained these data were fitted to a linear line of best fit to estimate a EC_{50} value. Where '-' is shown, data was fitted to a linear line of best fit.

Compound	Timepoint	EC_{50} (mM)	R^2	*n*	Compound	Timepoint	EC_{50} (mM)	R^2	*n*
	1	0.0677	0.997	2.778		1	0.181	0.985	2.408
	2	0.0320	0.918	0.975		2	0.064	0.922	1.268
**1**	3	0.0156	0.901	0.826	**d**	3	0.027	0.921	1.01
	4	0.0062	0.866	1.292		4	0.014	0.912	1.009
	5	0.0037	0.847	1.192		5	0.008	0.905	1.062
	1	> 1.5	0.892	-		1	0.936	0.905	-
	2	1.136	0.98	-		2	0.674	0.936	-
**2**	3	0.727	0.992	-	**e**	3	0.526	0.903	-
	4	0.563	0.955	-		4	0.456	0.845	-
	5	0.49	0.914	-		5	0.376	0.772	-
	1	0.867	0.916	-		1	1.108	0.924	-
	2	0.615	0.933	-		2	0.710	0.941	-
**3**	3	0.529	0.914	-	**f**	3	0.436	0.937	-
	4	0.150	0.936	0.332		4	0.357	0.911	-
	5	0.170	0.917	0.315		5	0.364	0.862	-
	1	1.017	0.915			1	1.164	0.905	-
	2	0.396	0.961	1.321		2	0.644	0.929	-
**4**	3	0.341	0.917	0.784	**g**	3	0.19	0.933	0.53
	4	0.288	0.889	0.625		4	0.20	0.901	0.454
	5	0.218	0.869	0.559		5	0.15	0.948	0.57
	1	> 1.5	0.885	-		1	0.409	0.942	-
	2	0.641	0.941	0.79		2	0.441	0.837	-
**5**	3	0.130	0.933	0.923	**h**	3	0.15	0.889	0.502
	4	0.088	0.906	0.743		4	0.13	0.871	0.383
	5	0.061	0.882	0.67		5	0.05	0.857	0.343
	1	0.132	0.998	2.583		1	0.414	0.91	-
	2	0.083	0.92	0.802		2	0.264	0.883	2.801
**a**	3	0.041	0.837	0.679	**i**	3	0.15	0.894	0.687
	4	0.022	0.598	0.661		4	0.14	0.881	0.465
	5	0.011	0.642	0.708		5	0.05	0.873	0.392
	1	0.146	0.994	2.301		1	1.281	0.981	-
	2	0.059	0.988	0.976		2	0.618	0.976	-
**b**	3	0.029	0.988	0.903	**j**	3	0.460	0.897	-
	4	0.020	0.986	0.833		4	0.322	0.934	-
	5	0.008	0.995	0.701		5	0.276	0.905	-
	1	0.991	0.965	1.155					
	2	0.092	0.939	0.831					
**c**	3	0.052	0.89	0.667					
	4	0.023	0.886	0.678					
	5	0.012	0.866	0.727					

PE:PG 3:1 vesicle lysis titrations

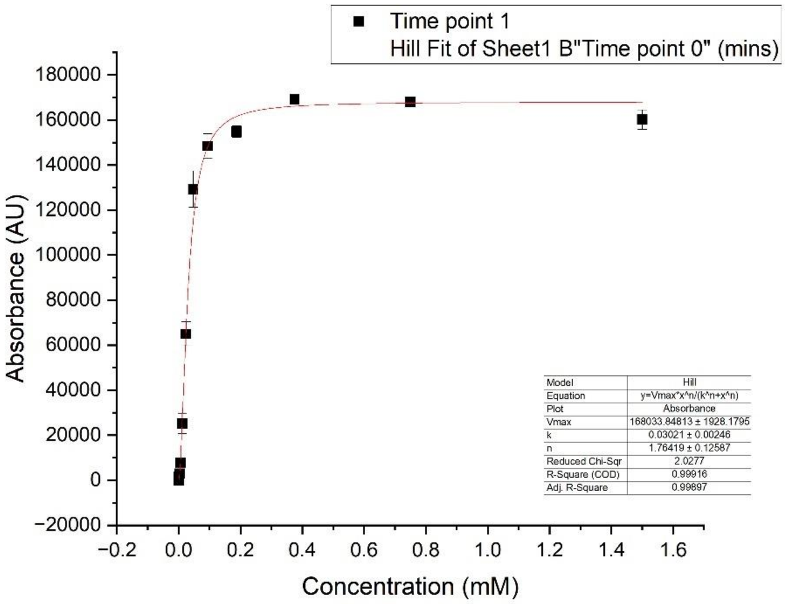

Figure S244 – Graph showing the lysis of calcein loaded PE:PG 3:1 vesicles at 1 minute, with respect to increasing concentration of **1**. Data was then fitted to the Hill equation. Average of n= 3, error = one standard deviation of the mean.

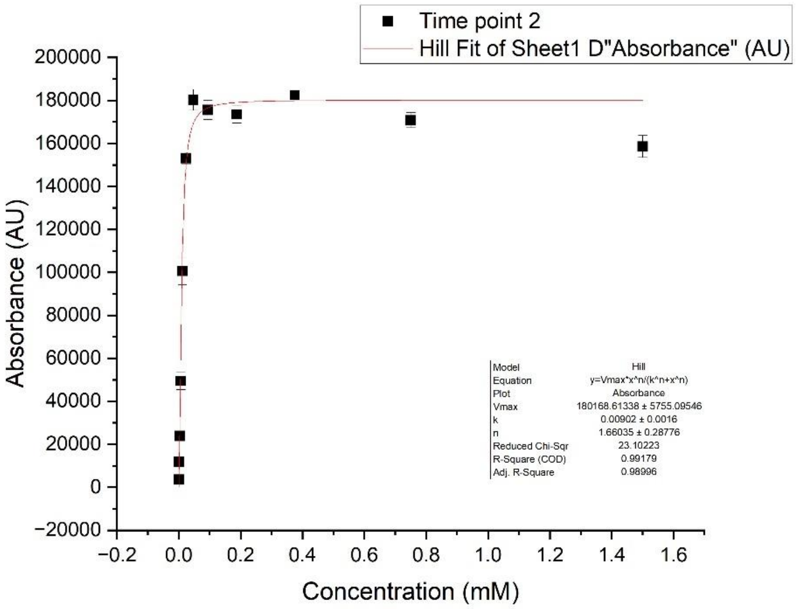

Figure S245 – Graph showing the lysis of calcein loaded PE:PG 3:1 vesicles at 5 minutes, with respect to increasing concentration of **1**. Data was then fitted to the Hill equation. Average of n= 3, error = one standard deviation of the mean.

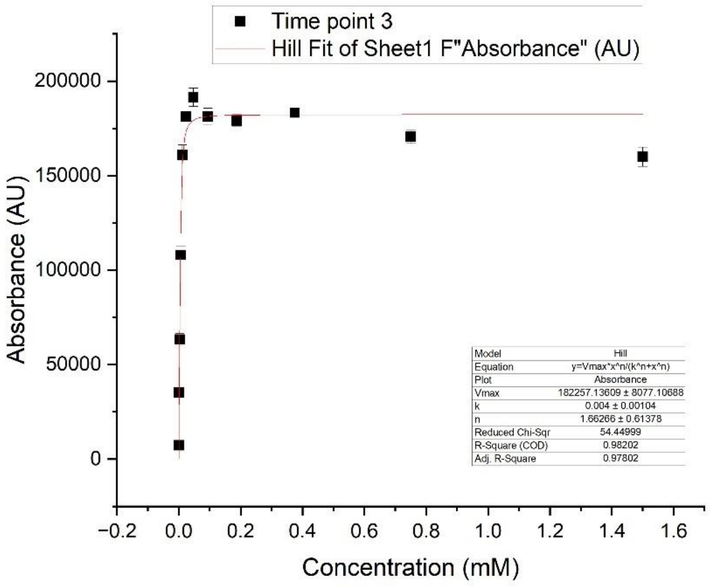

Figure S246 – Graph showing the lysis of calcein loaded PE:PG 3:1 vesicles at 10 minutes, with respect to increasing concentration of **1**. Data was then fitted to the Hill equation. Average of n= 3, error = one standard deviation of the mean.

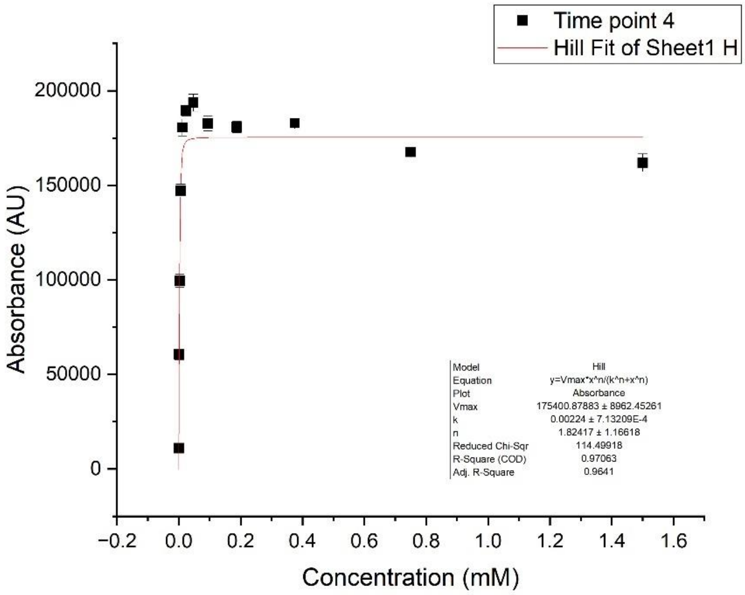

Figure S247 – Graph showing the lysis of calcein loaded PE:PG 3:1 vesicles at 15 minutes, with respect to increasing concentration of **1**. Data was then fitted to the Hill equation. Average of n= 3, error = one standard deviation of the mean.

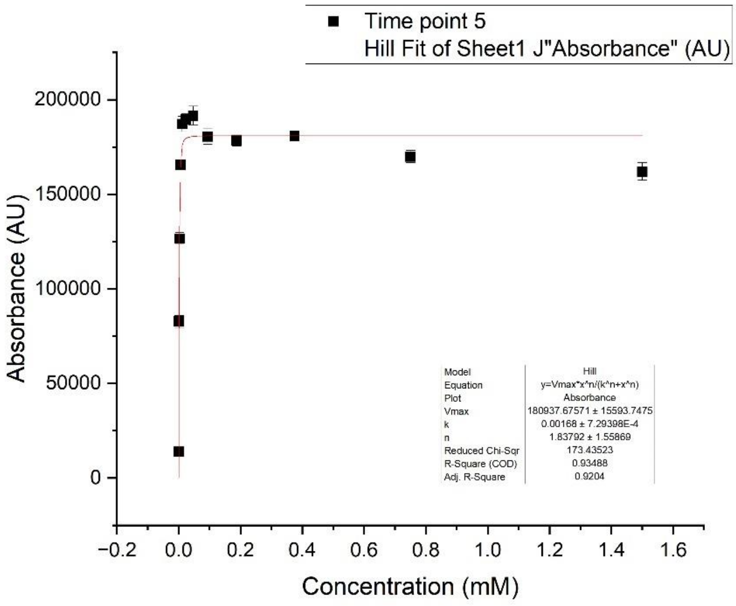

Figure S248 – Graph showing the lysis of calcein loaded PE:PG 3:1 vesicles at 20 minutes, with respect to increasing concentration of **1**. Data was then fitted to the Hill equation. Average of n= 3, error = one standard deviation of the mean.

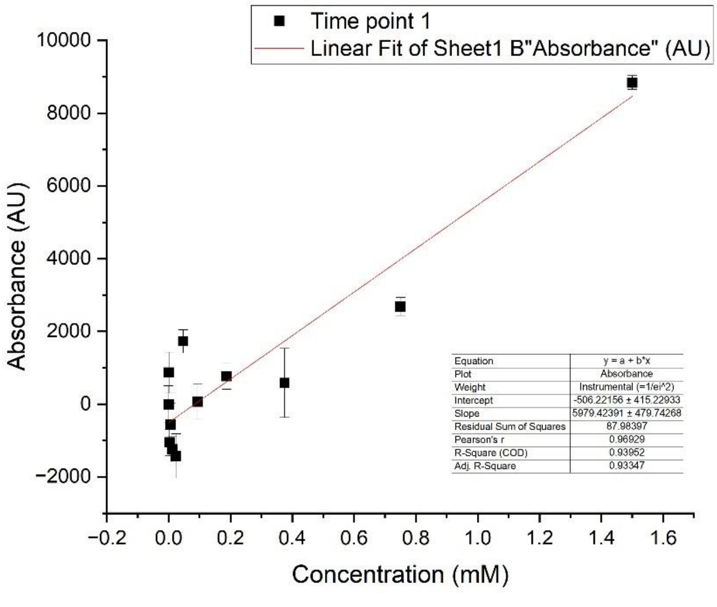

Figure S249 – Graph showing the lysis of calcein loaded PE:PG 3:1 vesicles at 1 minute, with respect to increasing concentration of **2**. Data was then fitted to a linear line of best fit. Average of n= 3, error = one standard deviation of the mean.

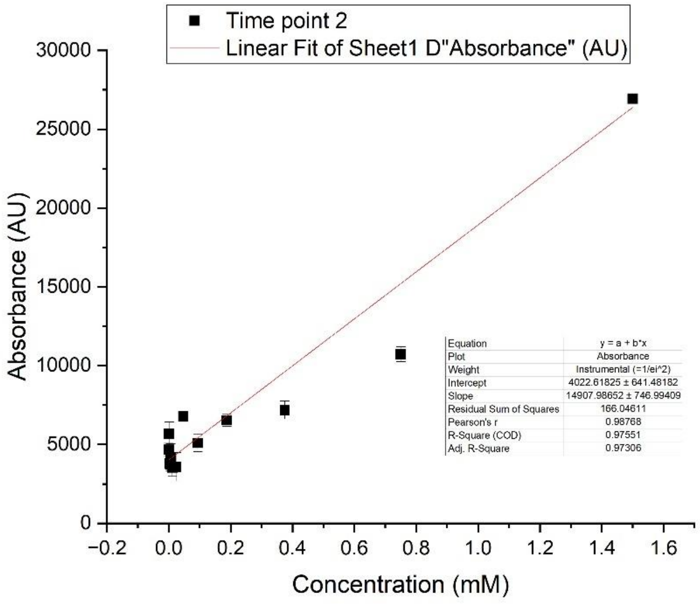

Figure S250 – Graph showing the lysis of calcein loaded PE:PG 3:1 vesicles at 5 minutes, with respect to increasing concentration of **2**. Data was then fitted to a linear line of best fit. Average of n= 3, error = one standard deviation of the mean.

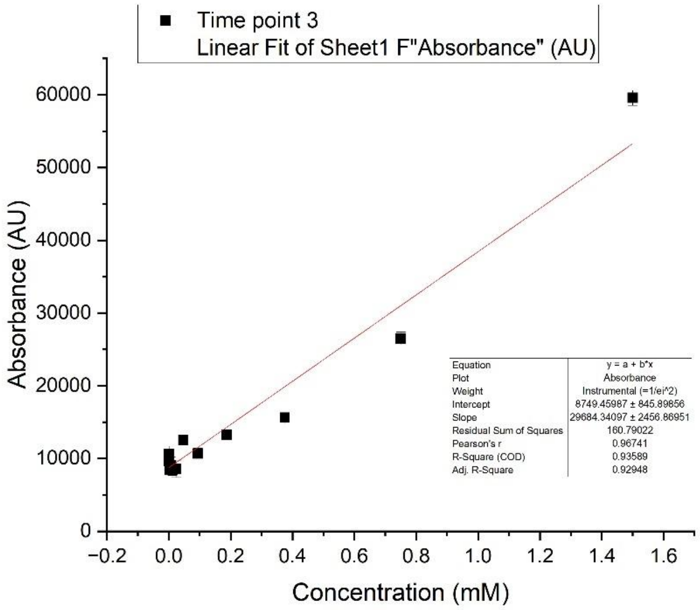

Figure S251 – Graph showing the lysis of calcein loaded PE:PG 3:1 vesicles at 10 minutes, with respect to increasing concentration of **2**. Data was then fitted to a linear line of best fit. Average of n= 3, error = one standard deviation of the mean.

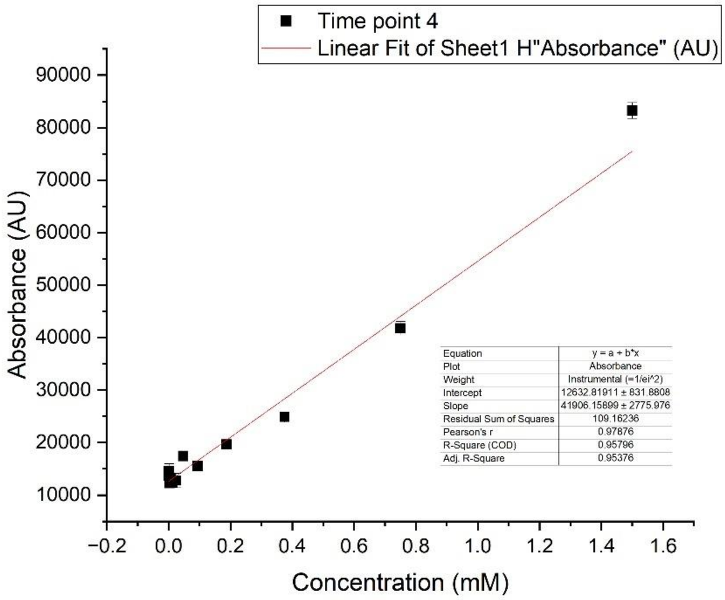

Figure S252 – Graph showing the lysis of calcein loaded PE:PG 3:1 vesicles at 15 minutes, with respect to increasing concentration of **2**. Data was then fitted to a linear line of best fit. Average of n= 3, error = one standard deviation of the mean.

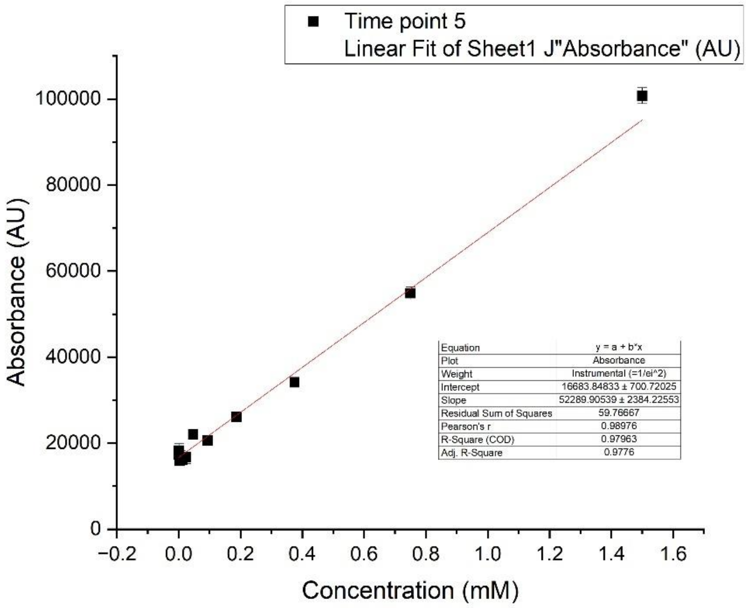

Figure S253 – Graph showing the lysis of calcein loaded PE:PG 3:1 vesicles at 20 minutes, with respect to increasing concentration of **2**. Data was then fitted to a linear line of best fit. Average of n= 3, error = one standard deviation of the mean.

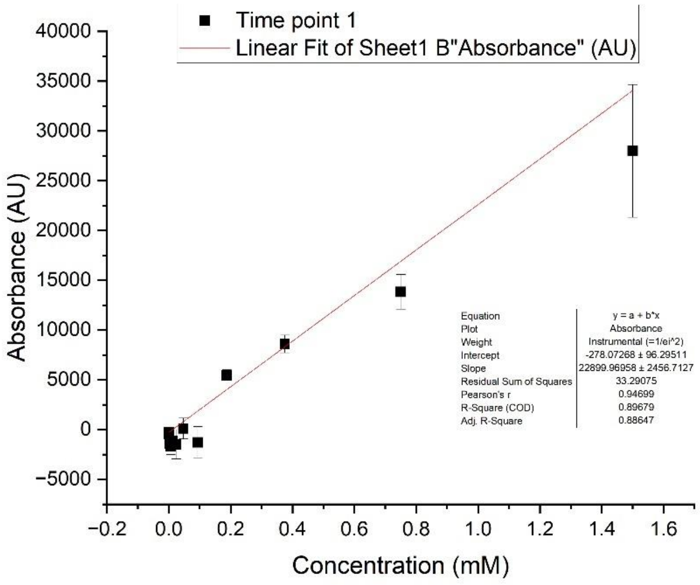

Figure S254 – Graph showing the lysis of calcein loaded PE:PG 3:1 vesicles at 1 minute, with respect to increasing concentration of **3**. Data was then fitted to a linear line of best fit. Average of n= 3, error = one standard deviation of the mean.

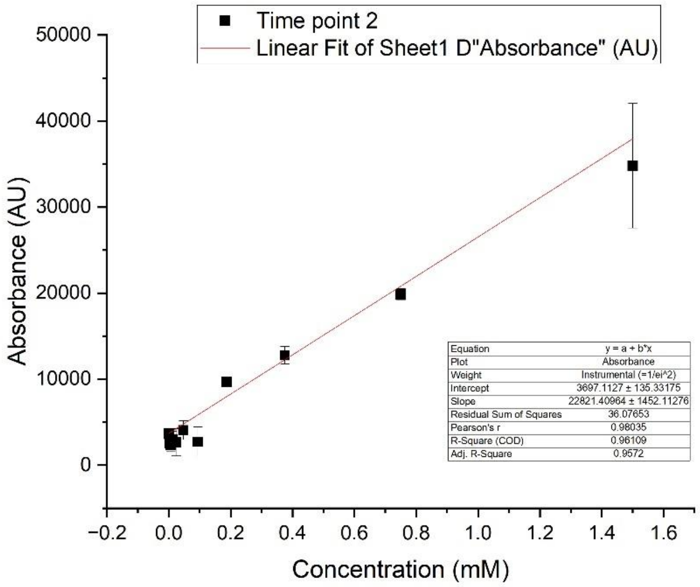

Figure S255 – Graph showing the lysis of calcein loaded PE:PG 3:1 vesicles at 5 minutes, with respect to increasing concentration of **3**. Data was then fitted to a linear line of best fit. Average of n= 3, error = one standard deviation of the mean.

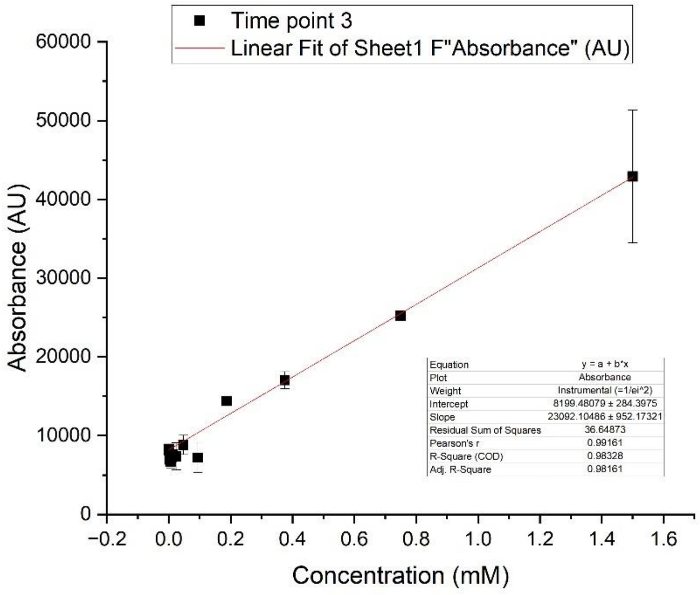

Figure S256 – Graph showing the lysis of calcein loaded PE:PG 3:1 vesicles at 10 minutes, with respect to increasing concentration of **3**. Data was then fitted to a linear line of best fit. Average of n= 3, error = one standard deviation of the mean.

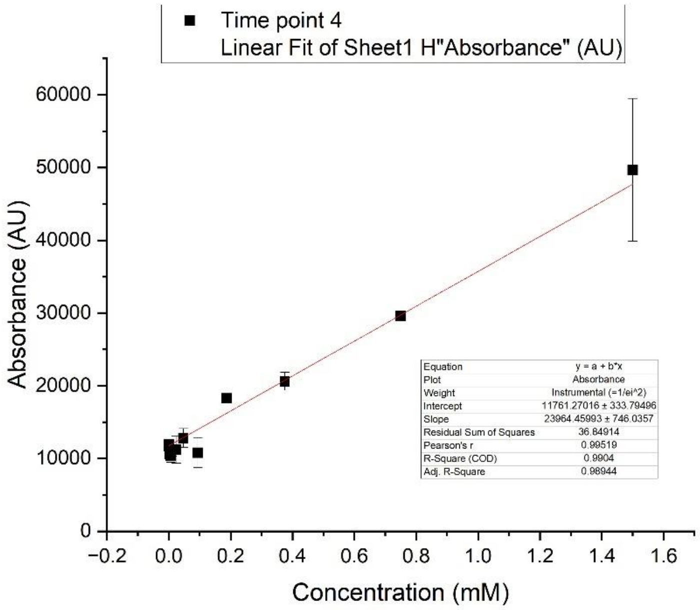

Figure S257 – Graph showing the lysis of calcein loaded PE:PG 3:1 vesicles at 15 minutes, with respect to increasing concentration of **3**. Data was then fitted to a linear line of best fit. Average of n= 3, error = one standard deviation of the mean.

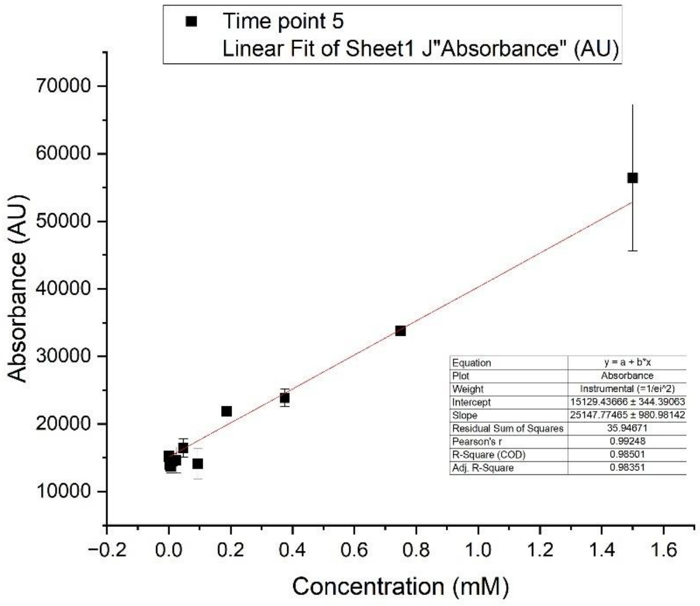

Figure S258 – Graph showing the lysis of calcein loaded PE:PG 3:1 vesicles at 20 minutes, with respect to increasing concentration of **3**. Data was then fitted to a linear line of best fit. Average of n= 3, error = one standard deviation of the mean.

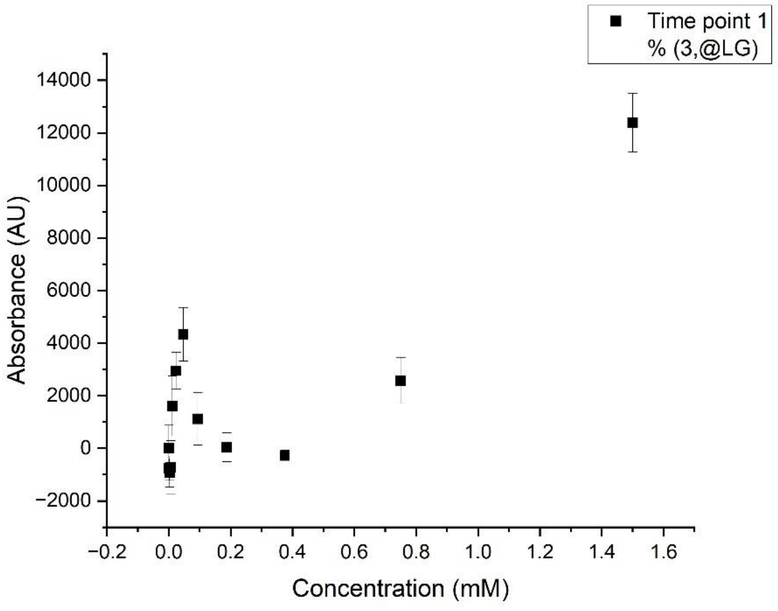

Figure S259 – Graph showing the lysis of calcein loaded PE:PG 3:1 vesicles at 1 minute, with respect to increasing concentration of **4**. Data could not be fitted. Average of n= 3, error = one standard deviation of the mean.

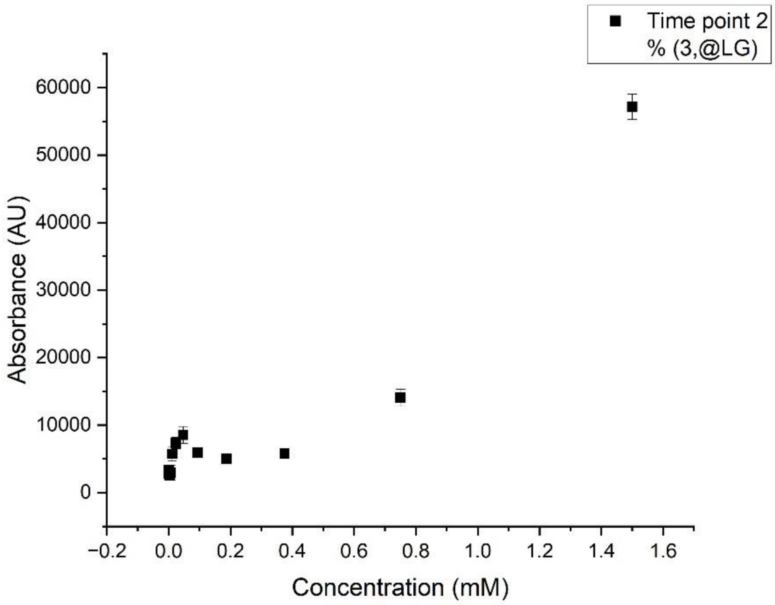

Figure S260 – Graph showing the lysis of calcein loaded PE:PG 3:1 vesicles at 5 minutes, with respect to increasing concentration of **4**. Data could not be fitted. Average of n= 3, error = one standard deviation of the mean.

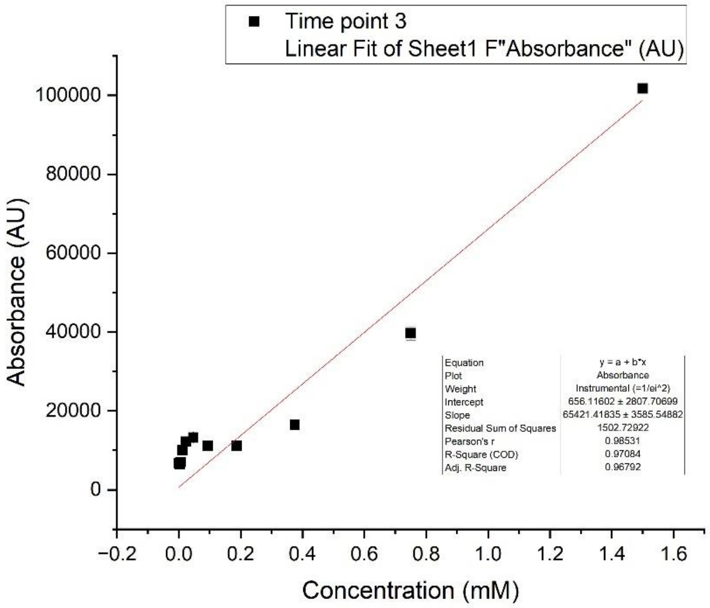

Figure S261 – Graph showing the lysis of calcein loaded PE:PG 3:1 vesicles at 10 minutes, with respect to increasing concentration of **4**. Data was then fitted to a linear line of best fit. Average of n= 3, error = one standard deviation of the mean.

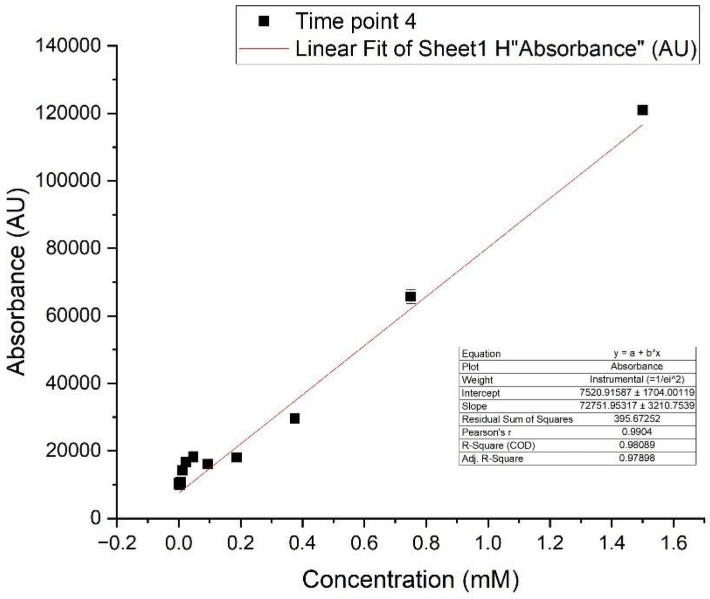

Figure S262 – Graph showing the lysis of calcein loaded PE:PG 3:1 vesicles at 15 minutes, with respect to increasing concentration of **4**. Data was then fitted to a linear line of best fit. Average of n= 3, error = one standard deviation of the mean.

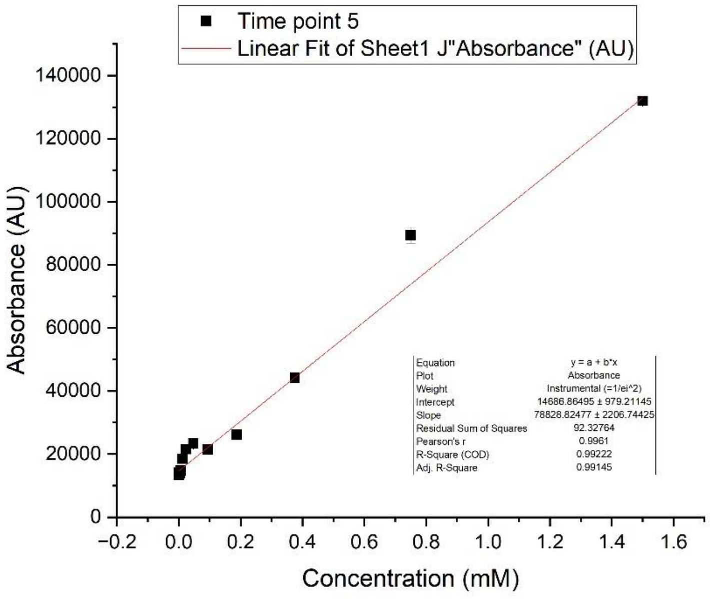

Figure S263 – Graph showing the lysis of calcein loaded PE:PG 3:1 vesicles at 20 minutes, with respect to increasing concentration of **4**. Data was then fitted to a linear line of best fit. Average of n= 3, error = one standard deviation of the mean.

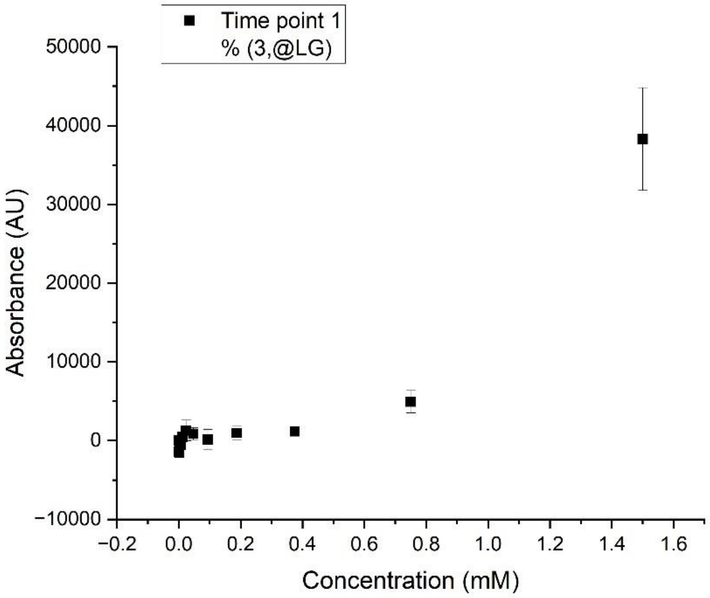

Figure S264 – Graph showing the lysis of calcein loaded PE:PG 3:1 vesicles at 1 minute, with respect to increasing concentration of **5**. Data could not be fitted. Average of n= 3, error = one standard deviation of the mean.

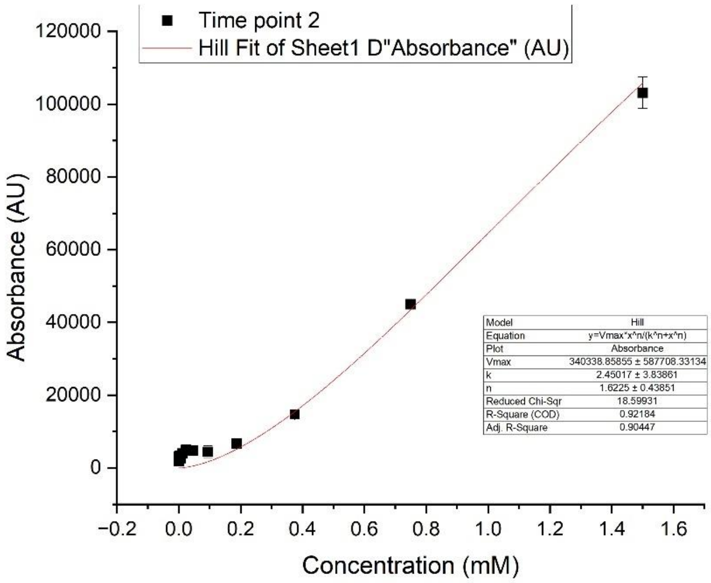

Figure S265 – Graph showing the lysis of calcein loaded PE:PG 3:1 vesicles at 5 minutes, with respect to increasing concentration of **5**. Data was then fitted to the Hill equation. Average of n= 3, error = one standard deviation of the mean.

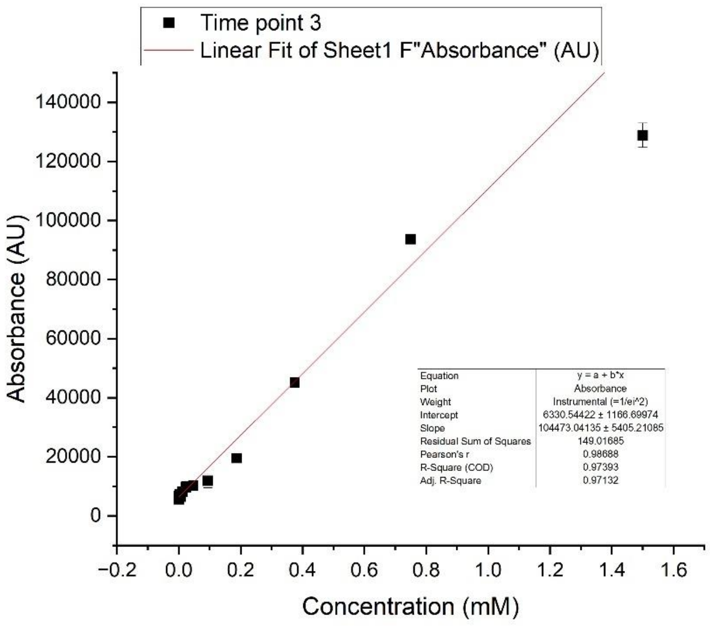

Figure S266 – Graph showing the lysis of calcein loaded PE:PG 3:1 vesicles at 10 minutes, with respect to increasing concentration of **5**. Data was then fitted to a linear line of best fit. Average of n= 3, error = one standard deviation of the mean.

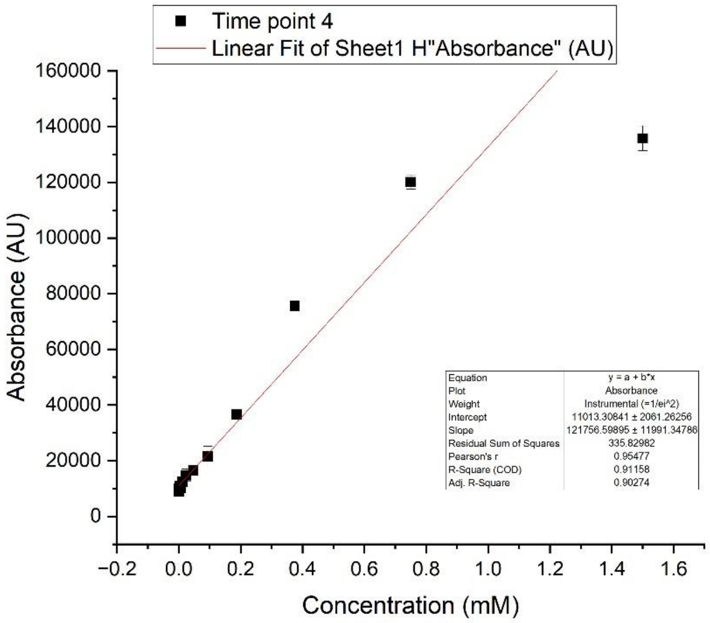

Figure S267 – Graph showing the lysis of calcein loaded PE:PG 3:1 vesicles at 15 minutes, with respect to increasing concentration of **5**. Data was then fitted to a linear line of best fit. Average of n= 3, error = one standard deviation of the mean.

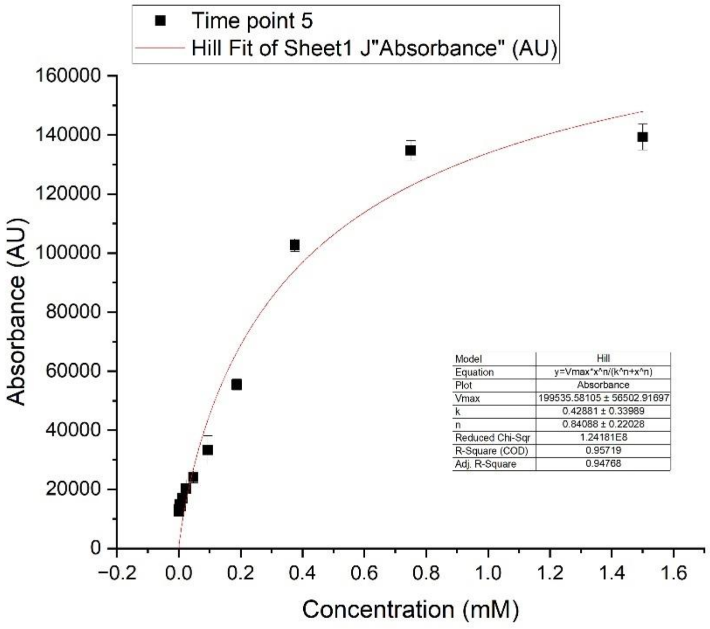

Figure S268 – Graph showing the lysis of calcein loaded PE:PG 3:1 vesicles at 20 minutes, with respect to increasing concentration of **5**. Data was then fitted to the Hill equation. Average of n= 3, error = one standard deviation of the mean.

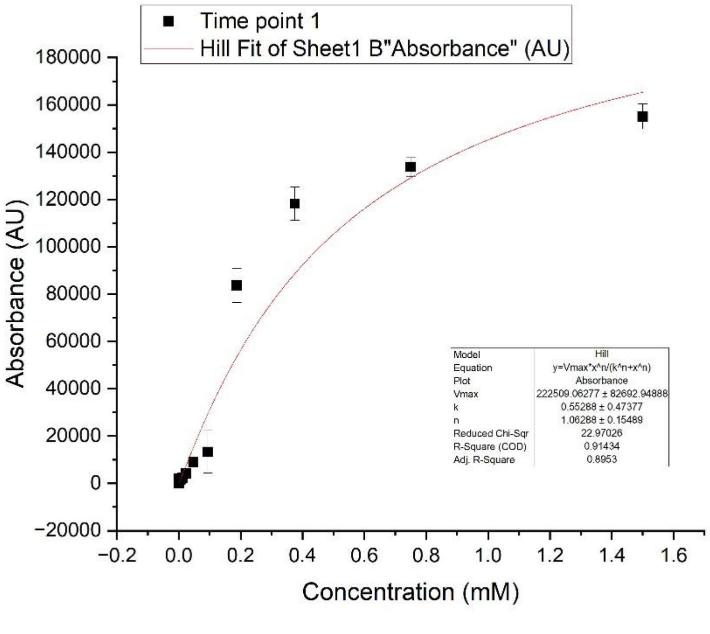

Figure S269 – Graph showing the lysis of calcein loaded PE:PG 3:1 vesicles at 1 minute, with respect to increasing concentration of **a**. Data was then fitted to the Hill equation. Average of n= 3, error = one standard deviation of the mean.

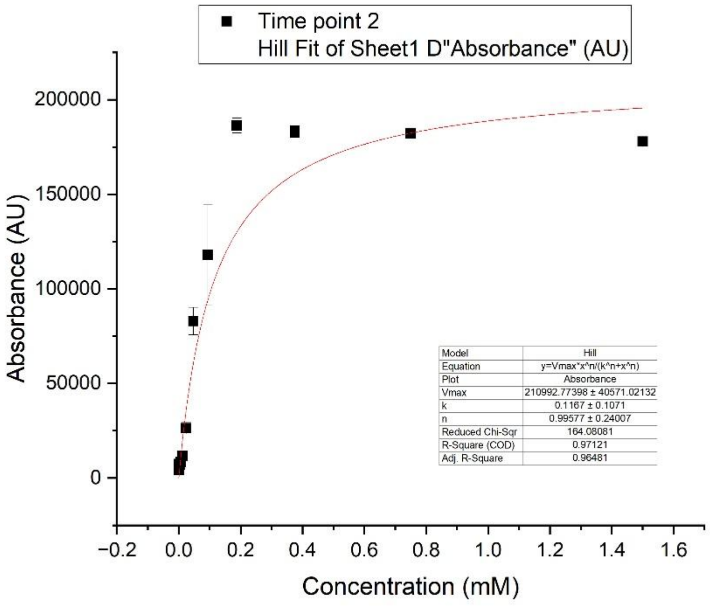

Figure S270 – Graph showing the lysis of calcein loaded PE:PG 3:1 vesicles at 5 minutes, with respect to increasing concentration of **a**. Data was then fitted to the Hill equation. Average of n= 3, error = one standard deviation of the mean.

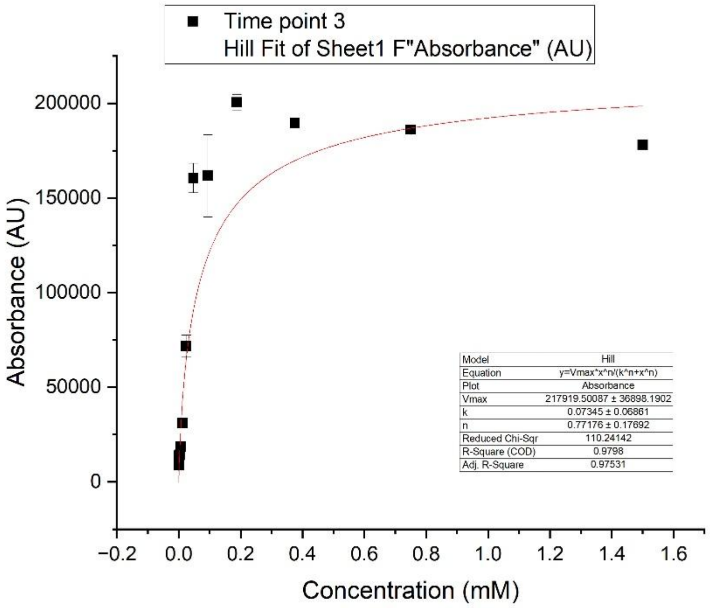

Figure S271 – Graph showing the lysis of calcein loaded PE:PG 3:1 vesicles at 10 minutes, with respect to increasing concentration of **a**. Data was then fitted to the Hill equation. Average of n= 3, error = one standard deviation of the mean.

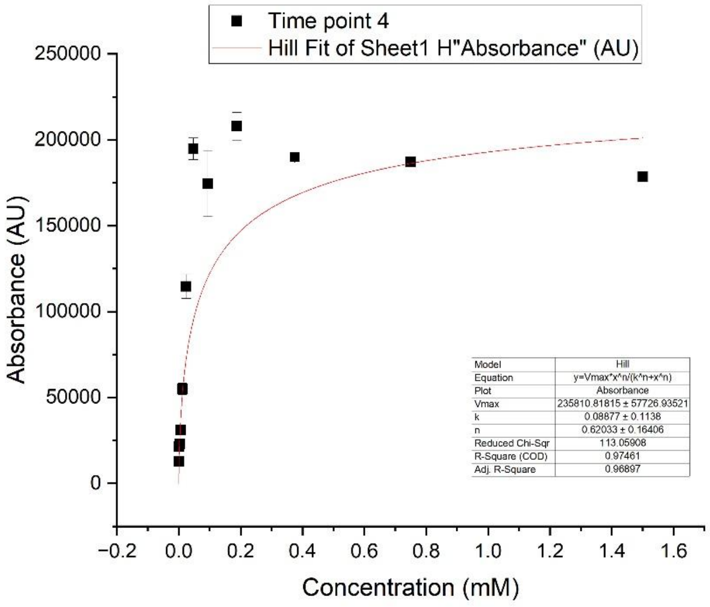

Figure S272 – Graph showing the lysis of calcein loaded PE:PG 3:1 vesicles at 15 minutes, with respect to increasing concentration of **a**. Data was then fitted to the Hill equation. Average of n= 3, error = one standard deviation of the mean.

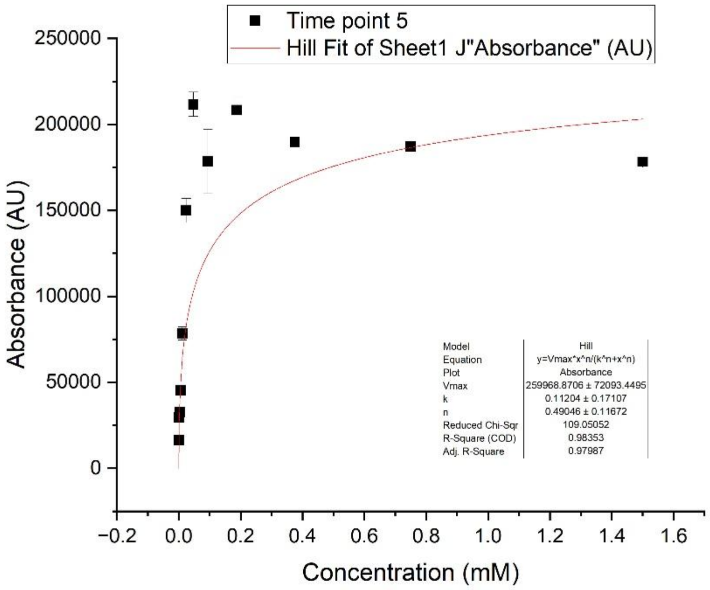

Figure S273 – Graph showing the lysis of calcein loaded PE:PG 3:1 vesicles at 20 minutes, with respect to increasing concentration of **a**. Data was then fitted to the Hill equation. Average of n= 3, error = one standard deviation of the mean.

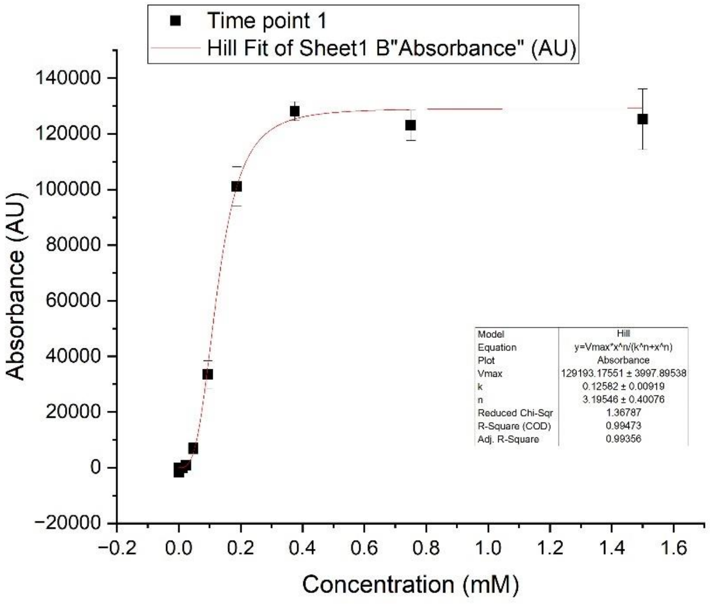

Figure S274 – Graph showing the lysis of calcein loaded PE:PG 3:1 vesicles at 1 minute, with respect to increasing concentration of **b**. Data was then fitted to the Hill equation. Average of n= 3, error = one standard deviation of the mean.

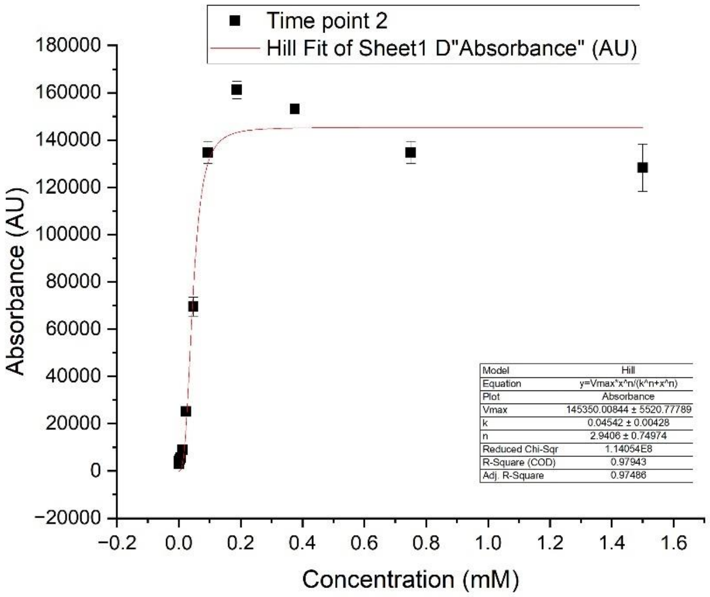

Figure S275 – Graph showing the lysis of calcein loaded PE:PG 3:1 vesicles at 5 minutes, with respect to increasing concentration of **b**. Data was then fitted to the Hill equation. Average of n= 3, error = one standard deviation of the mean.

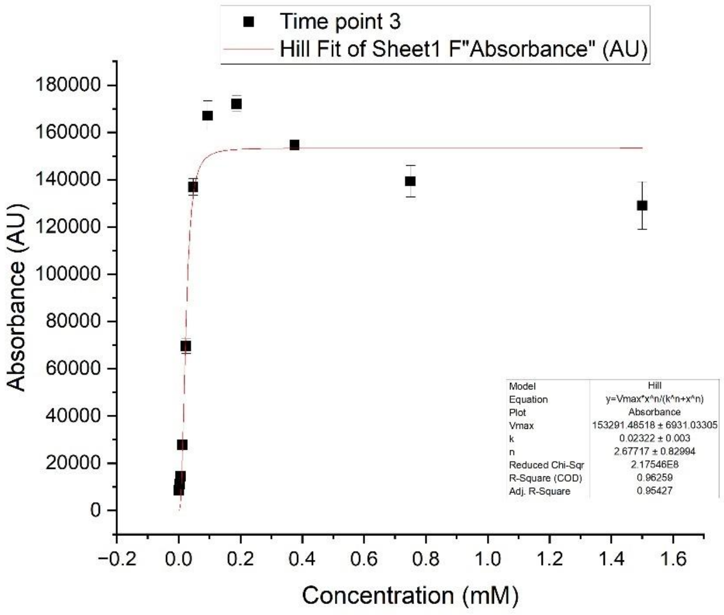

Figure S276 – Graph showing the lysis of calcein loaded PE:PG 3:1 vesicles at 10 minutes, with respect to increasing concentration of **b**. Data was then fitted to the Hill equation. Average of n= 3, error = one standard deviation of the mean.

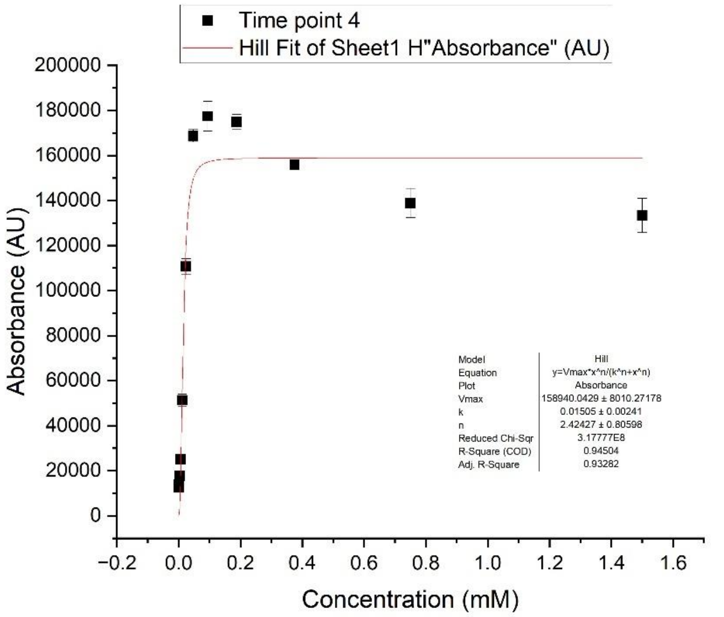

Figure S277 – Graph showing the lysis of calcein loaded PE:PG 3:1 vesicles at 15 minutes, with respect to increasing concentration of **b**. Data was then fitted to the Hill equation. Average of n= 3, error = one standard deviation of the mean.

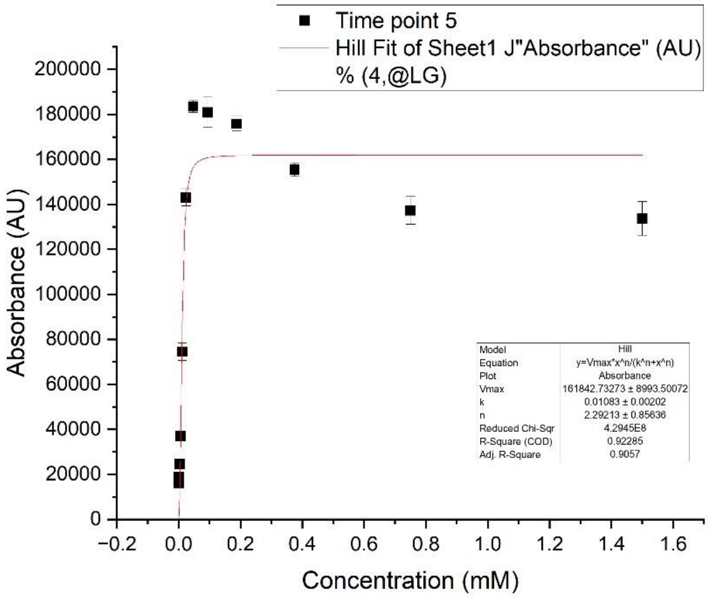

Figure S278 – Graph showing the lysis of calcein loaded PE:PG 3:1 vesicles at 20 minutes, with respect to increasing concentration of **b**. Data was then fitted to the Hill equation. Average of n= 3, error = one standard deviation of the mean.

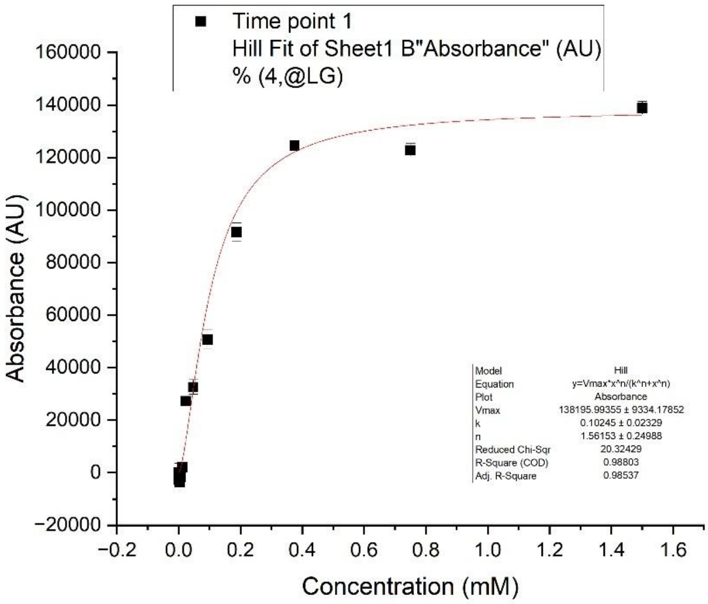

Figure S279 – Graph showing the lysis of calcein loaded PE:PG 3:1 vesicles at 1 minute, with respect to increasing concentration of **c**. Data was then fitted to the Hill equation. Average of n= 3, error = one standard deviation of the mean.

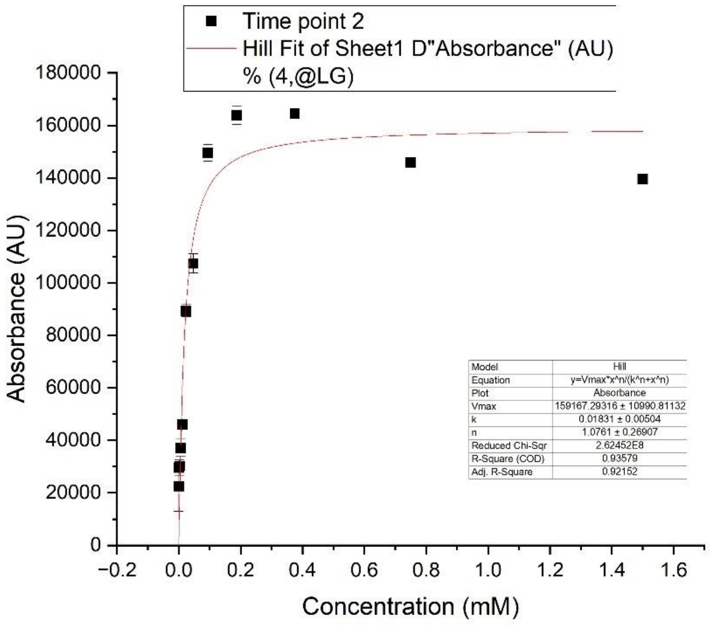

Figure S280 – Graph showing the lysis of calcein loaded PE:PG 3:1 vesicles at 5 minutes, with respect to increasing concentration of **c**. Data was then fitted to the Hill equation. Average of n= 3, error = one standard deviation of the mean.

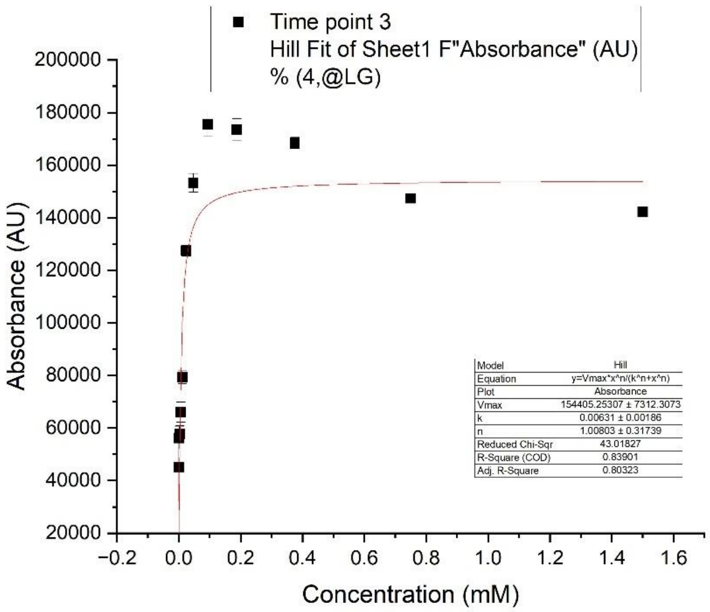

Figure S281 – Graph showing the lysis of calcein loaded PE:PG 3:1 vesicles at 10 minutes, with respect to increasing concentration of **c**. Data was then fitted to the Hill equation. Average of n= 3, error = one standard deviation of the mean.

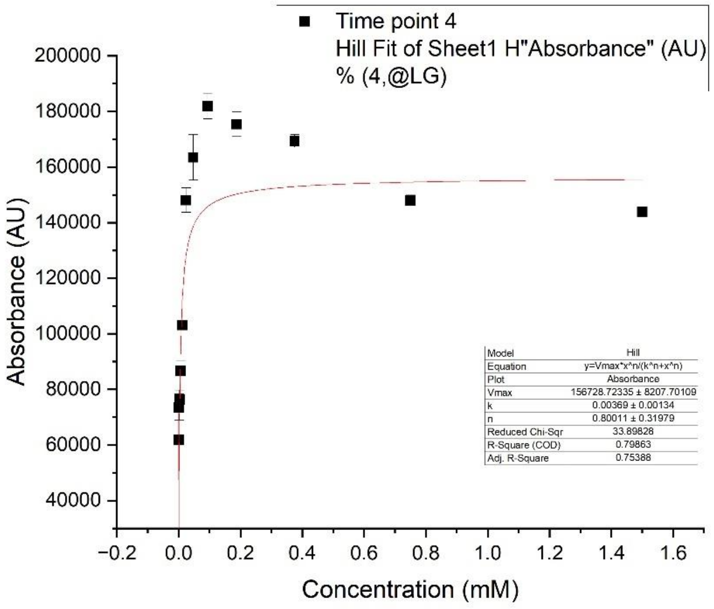

Figure S282 – Graph showing the lysis of calcein loaded PE:PG 3:1 vesicles at 15 minutes, with respect to increasing concentration of **c**. Data was then fitted to the Hill equation. Average of n= 3, error = one standard deviation of the mean.

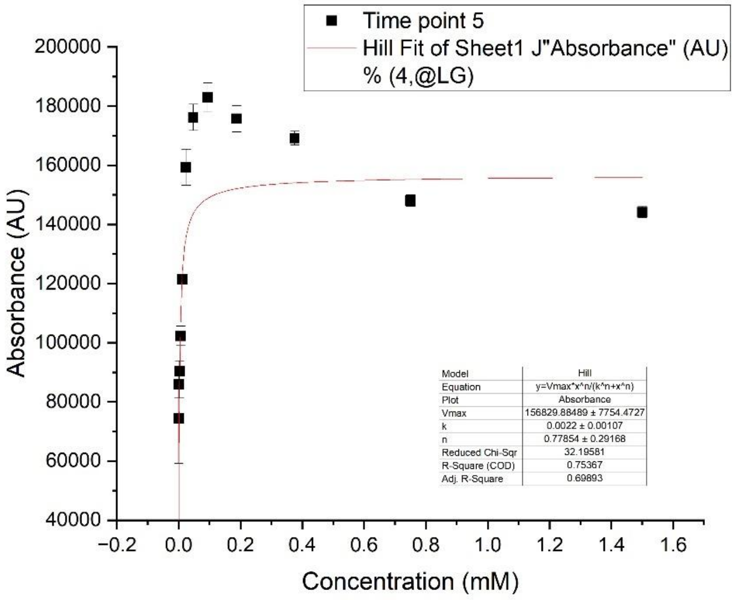

Figure S283 – Graph showing the lysis of calcein loaded PE:PG 3:1 vesicles at 20 minutes, with respect to increasing concentration of **c**. Data was then fitted to the Hill equation. Average of n= 3, error = one standard deviation of the mean.

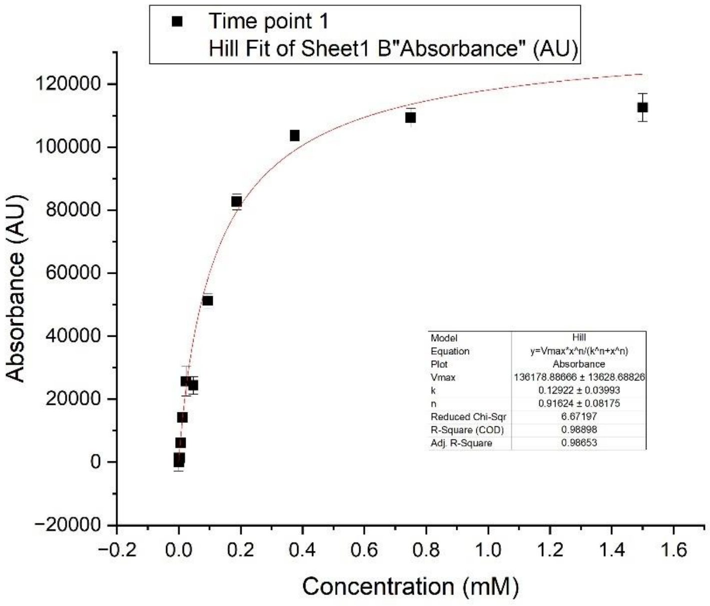

Figure S284 – Graph showing the lysis of calcein loaded PE:PG 3:1 vesicles at 1 minute, with respect to increasing concentration of **d**. Data was then fitted to the Hill equation. Average of n= 3, error = one standard deviation of the mean.

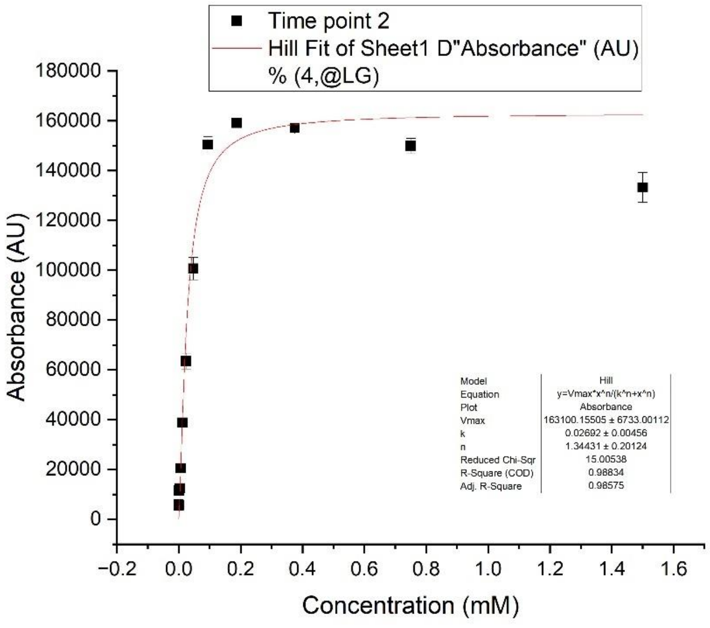

Figure S285 – Graph showing the lysis of calcein loaded PE:PG 3:1 vesicles at 5 minutes, with respect to increasing concentration of **d**. Data was then fitted to the Hill equation. Average of n= 3, error = one standard deviation of the mean.

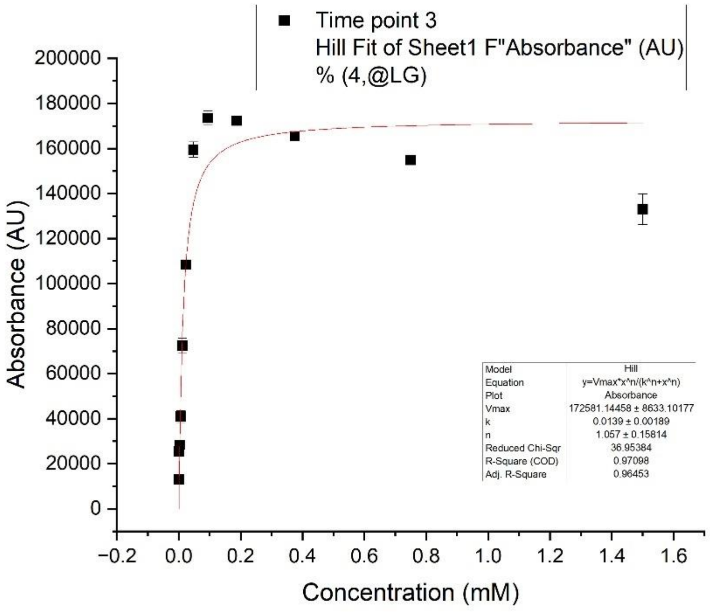

Figure S286 – Graph showing the lysis of calcein loaded PE:PG 3:1 vesicles at 10 minutes, with respect to increasing concentration of **d**. Data was then fitted to the Hill equation. Average of n= 3, error = one standard deviation of the mean.

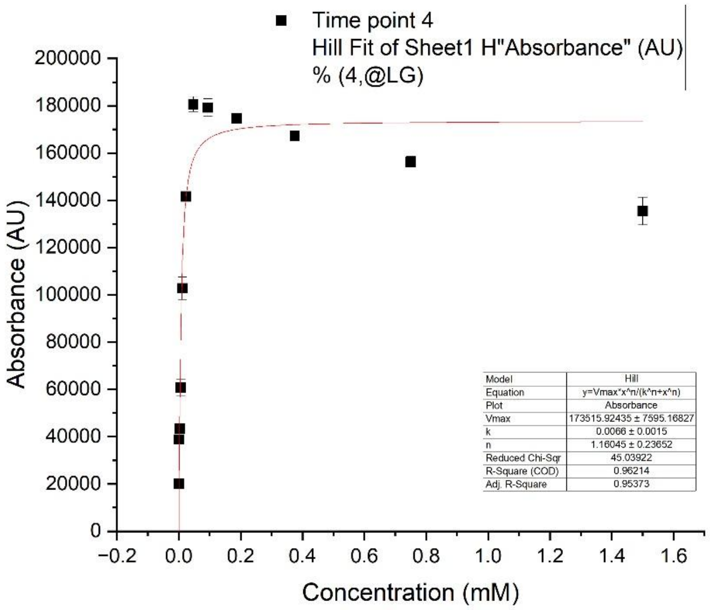

Figure S287 – Graph showing the lysis of calcein loaded PE:PG 3:1 vesicles at 15 minutes, with respect to increasing concentration of **d**. Data was then fitted to the Hill equation. Average of n= 3, error = one standard deviation of the mean.

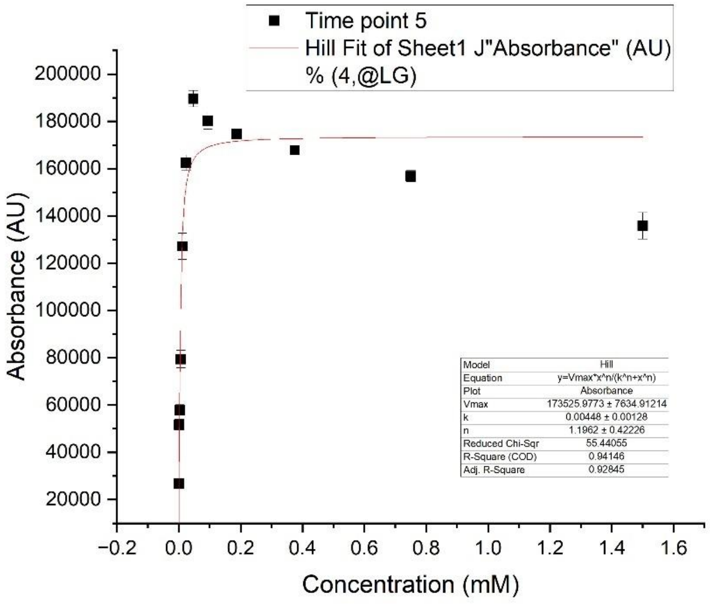

Figure S288 – Graph showing the lysis of calcein loaded PE:PG 3:1 vesicles at 20 minutes, with respect to increasing concentration of **d**. Data was then fitted to the Hill equation. Average of n= 3, error = one standard deviation of the mean.

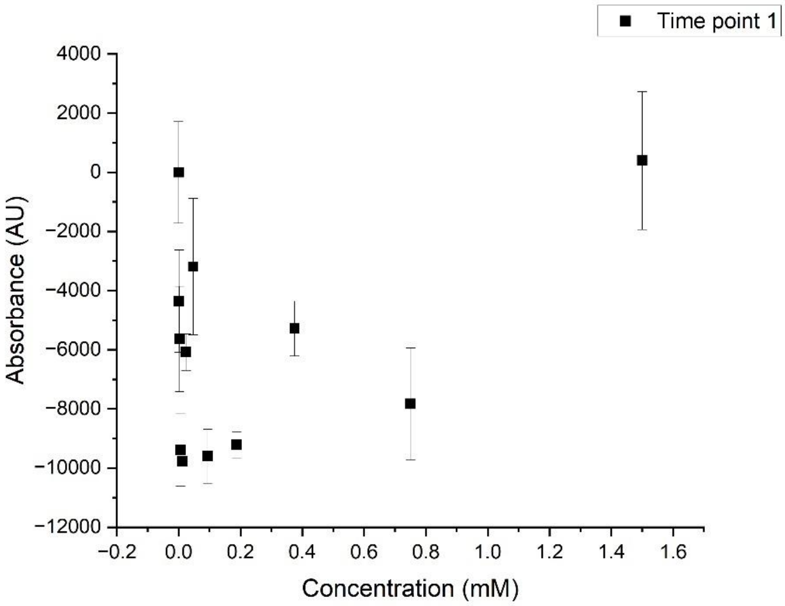

Figure S289 – Graph showing the lysis of calcein loaded PE:PG 3:1 vesicles at 1 minute, with respect to increasing concentration of **e**. Data could not be fitted. Average of n= 3, error = one standard deviation of the mean.

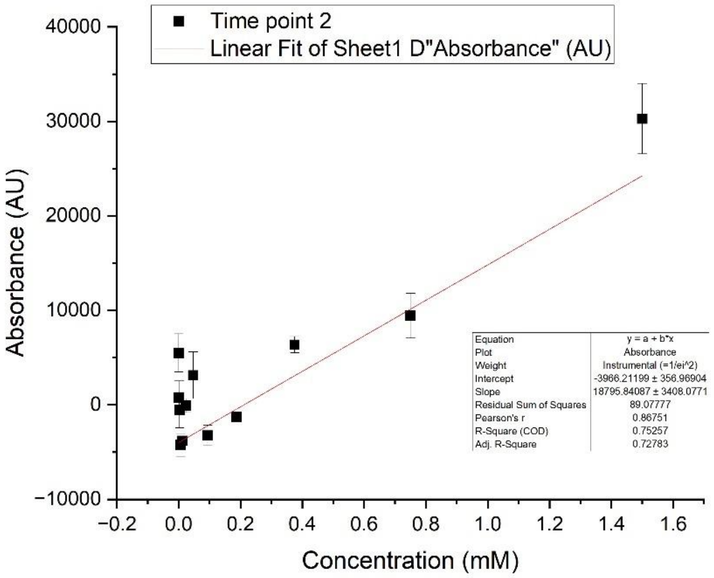

Figure S290 – Graph showing the lysis of calcein loaded PE:PG 3:1 vesicles at 5 minutes, with respect to increasing concentration of **e**. Data was then fitted to a linear line of best fit. Average of n= 3, error = one standard deviation of the mean.

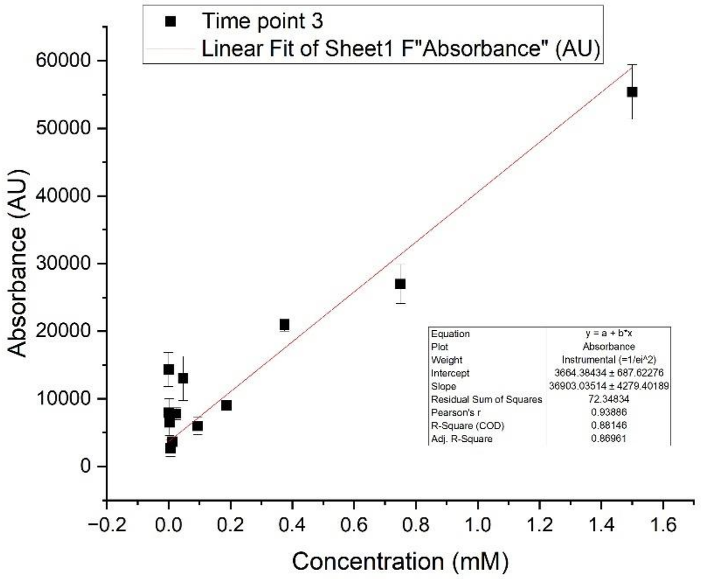

Figure S291 – Graph showing the lysis of calcein loaded PE:PG 3:1 vesicles at 10 minutes, with respect to increasing concentration of **e**. Data was then fitted to a linear line of best fit. Average of n= 3, error = one standard deviation of the mean.

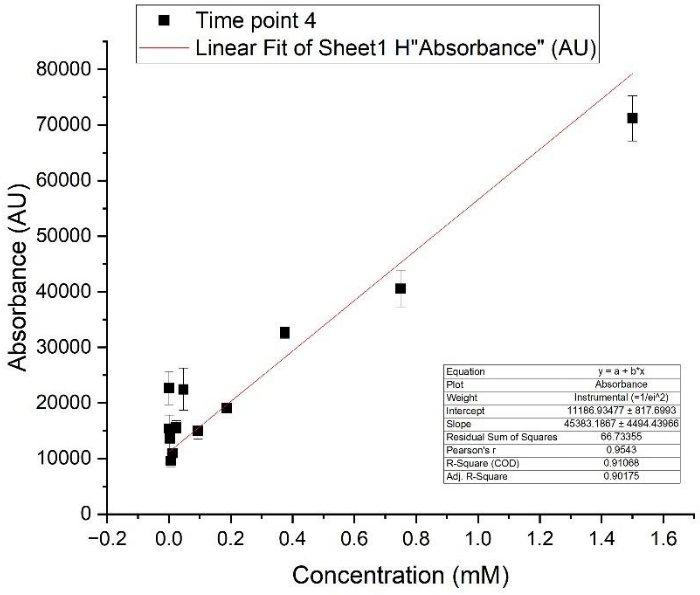

Figure S292 – Graph showing the lysis of calcein loaded PE:PG 3:1 vesicles at 15 minutes, with respect to increasing concentration of **e**. Data was then fitted to a linear line of best fit. Average of n= 3, error = one standard deviation of the mean.

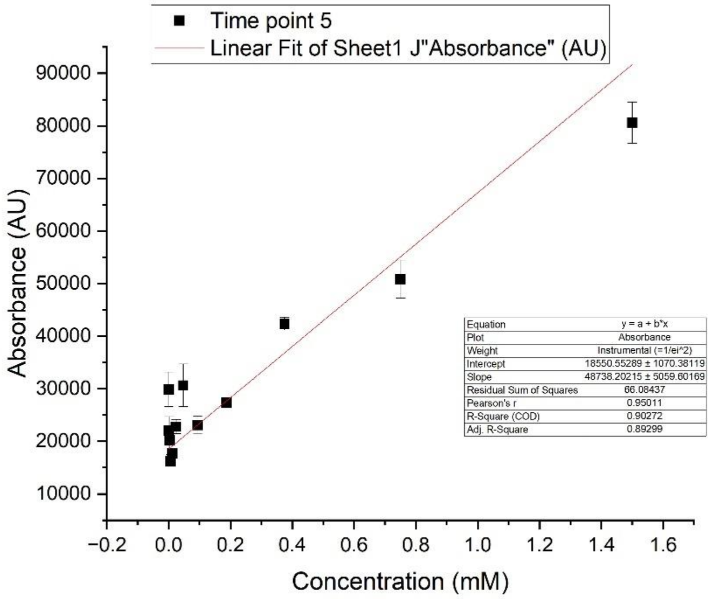

Figure S293 – Graph showing the lysis of calcein loaded PE:PG 3:1 vesicles at 20 minutes, with respect to increasing concentration of **e**. Data was then fitted to a linear line of best fit. Average of n= 3, error = one standard deviation of the mean.

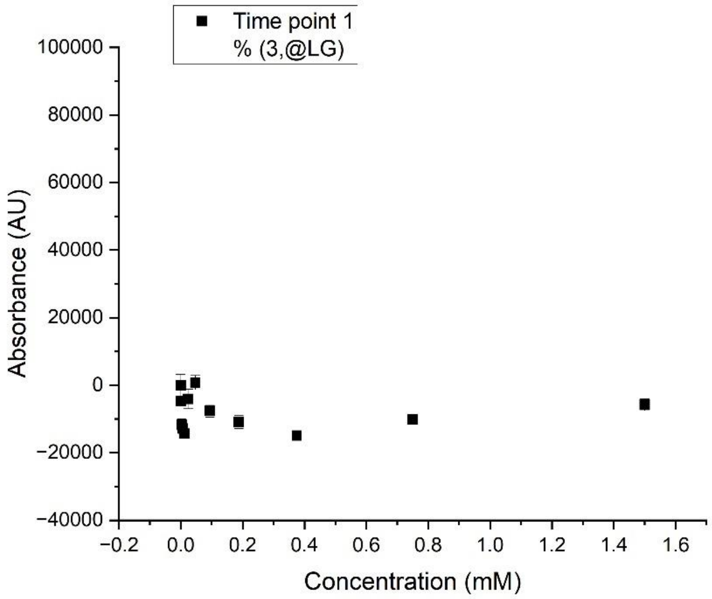

Figure S294 – Graph showing the lysis of calcein loaded PE:PG 3:1 vesicles at 1 minute, with respect to increasing concentration of **f**. Data could not be fitted. Average of n= 3, error = one standard deviation of the mean.

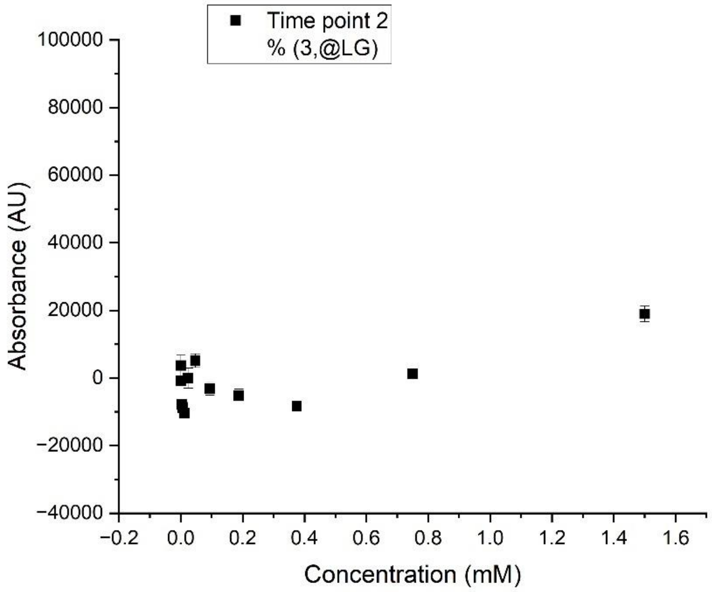

Figure S295 – Graph showing the lysis of calcein loaded PE:PG 3:1 vesicles at 5 minutes, with respect to increasing concentration of **f**. Data could not be fitted. Average of n= 3, error = one standard deviation of the mean.

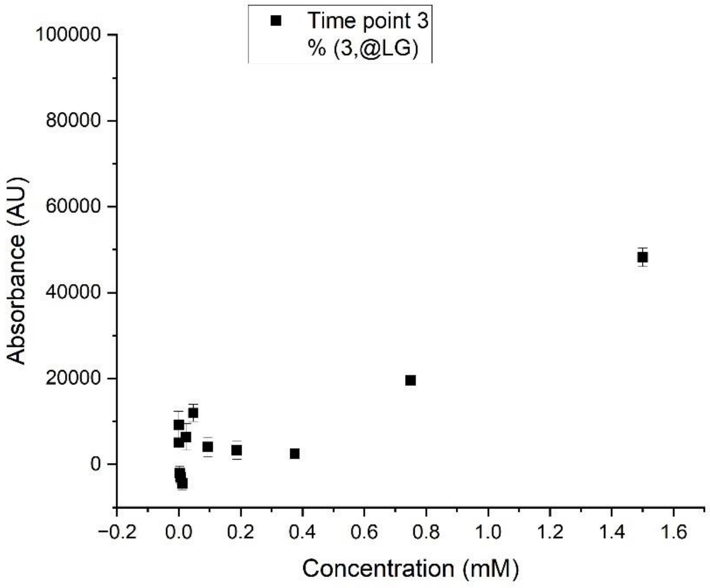

Figure S296 – Graph showing the lysis of calcein loaded PE:PG 3:1 vesicles at 10 minutes, with respect to increasing concentration of **f**. Data could not be fitted. Average of n= 3, error = one standard deviation of the mean.

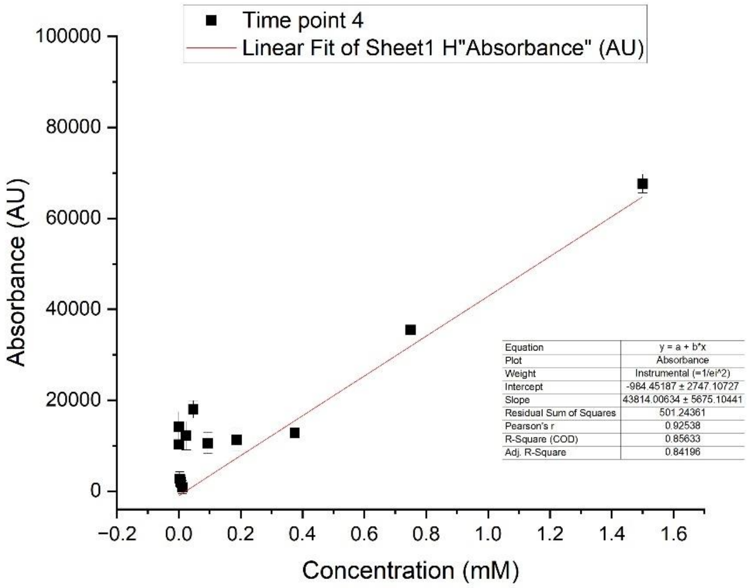

Figure S297 – Graph showing the lysis of calcein loaded PE:PG 3:1 vesicles at 15 minutes, with respect to increasing concentration of **f**. Data was then fitted to a linear line of best fit. Average of n= 3, error = one standard deviation of the mean.

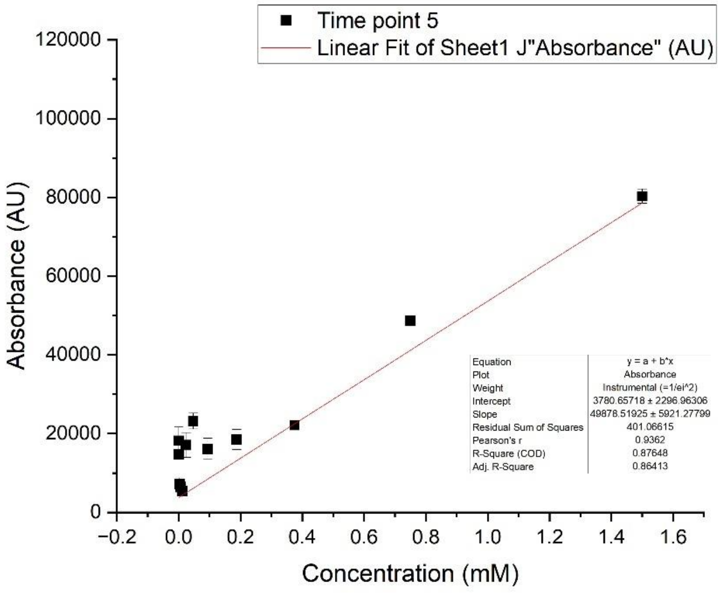

Figure S298 – Graph showing the lysis of calcein loaded PE:PG 3:1 vesicles at 20 minutes, with respect to increasing concentration of **f**. Data was then fitted to a linear line of best fit. Average of n= 3, error = one standard deviation of the mean.

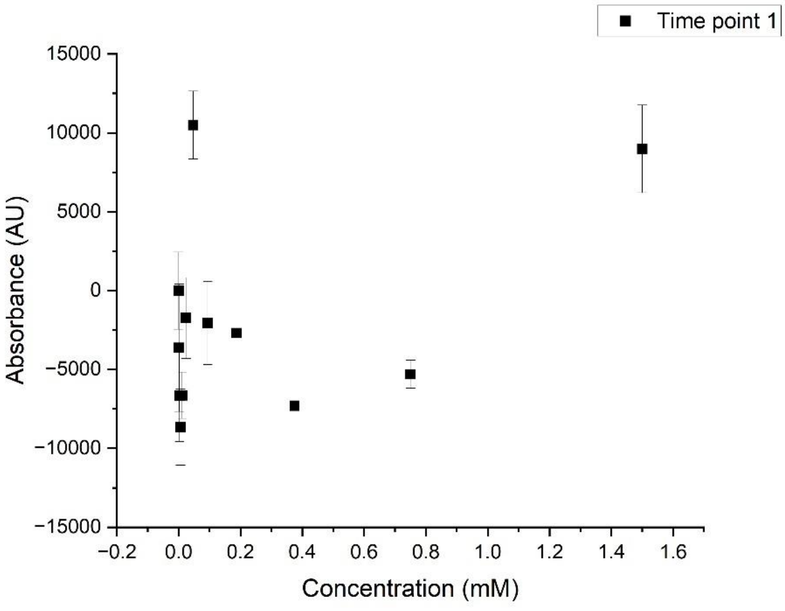

Figure S299 – Graph showing the lysis of calcein loaded PE:PG 3:1 vesicles at 1 minute, with respect to increasing concentration of **g**. Data could not be fitted. Average of n= 3, error = one standard deviation of the mean.

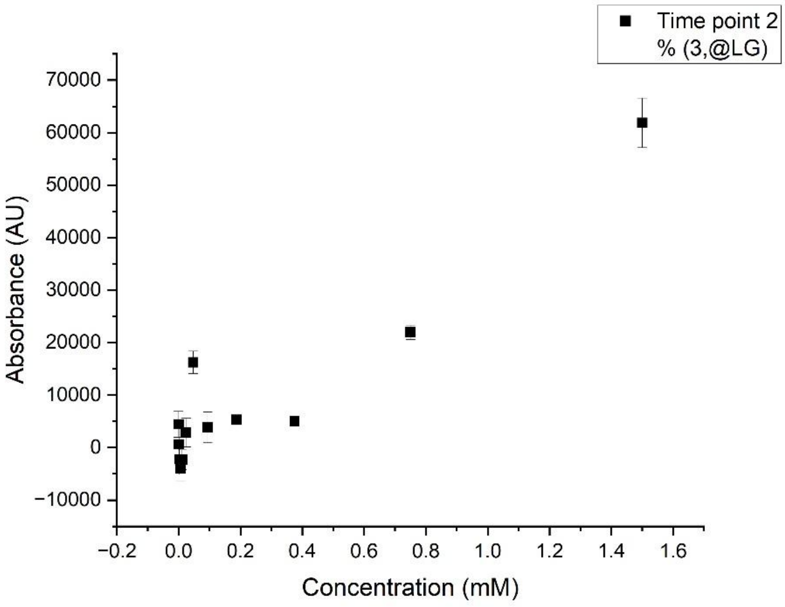

Figure S300 – Graph showing the lysis of calcein loaded PE:PG 3:1 vesicles at 5 minutes, with respect to increasing concentration of **g**. Data could not be fitted. Average of n= 3, error = one standard deviation of the mean.

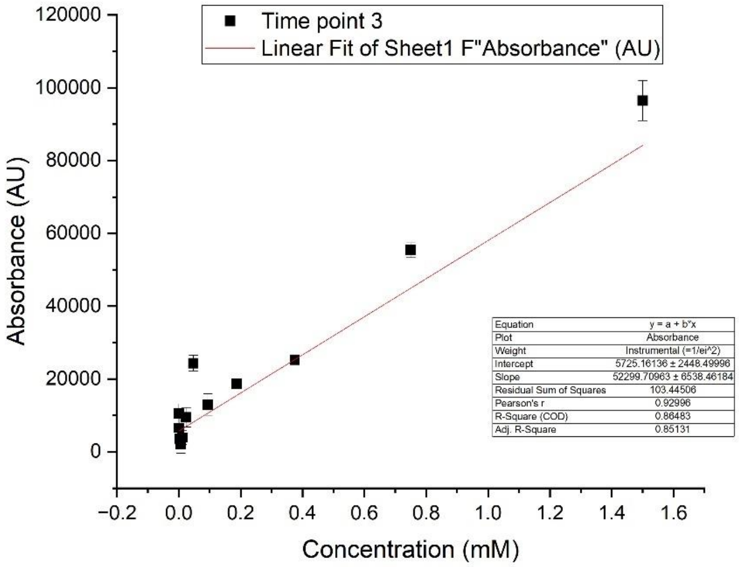

Figure S301 – Graph showing the lysis of calcein loaded PE:PG 3:1 vesicles at 10 minutes, with respect to increasing concentration of **g**. Data was then fitted to a linear line of best fit. Average of n= 3, error = one standard deviation of the mean.

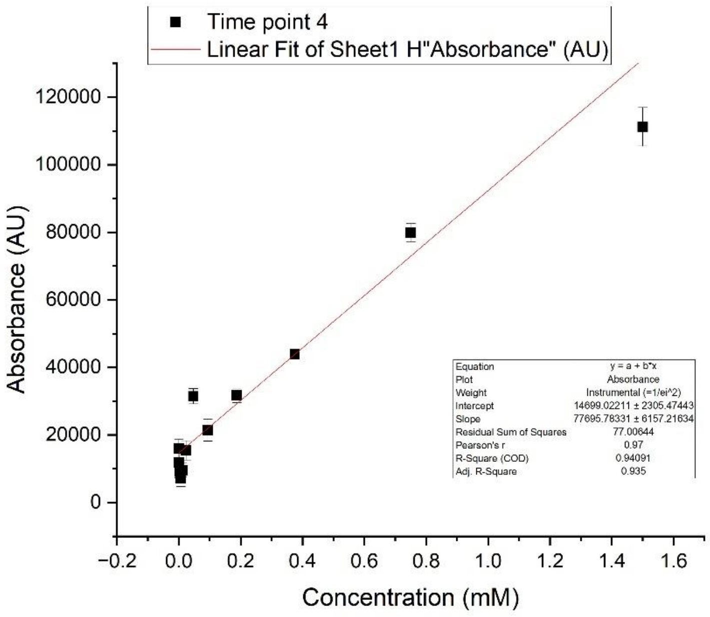

Figure S302 – Graph showing the lysis of calcein loaded PE:PG 3:1 vesicles at 15 minutes, with respect to increasing concentration of **g**. Data was then fitted to a linear line of best fit. Average of n= 3, error = one standard deviation of the mean.

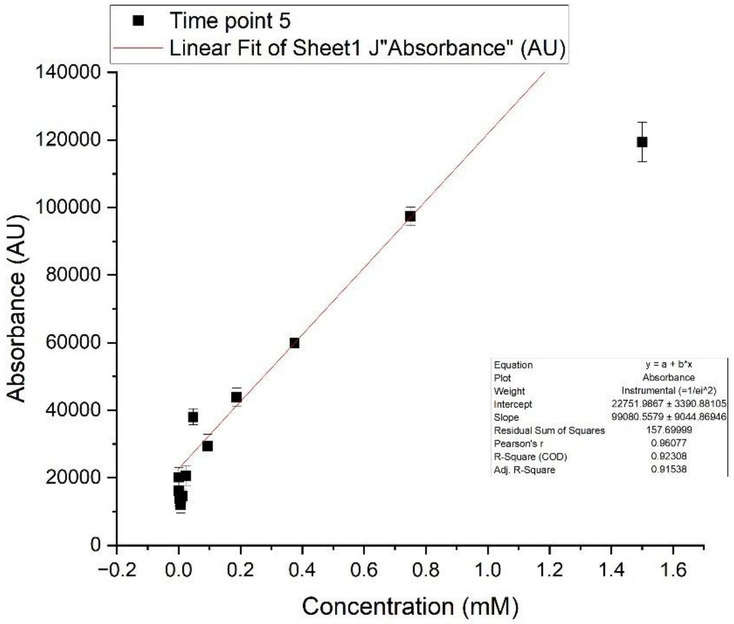

Figure S303 – Graph showing the lysis of calcein loaded PE:PG 3:1 vesicles at 20 minutes, with respect to increasing concentration of **g**. Data was then fitted to a linear line of best fit. Average of n= 3, error = one standard deviation of the mean.

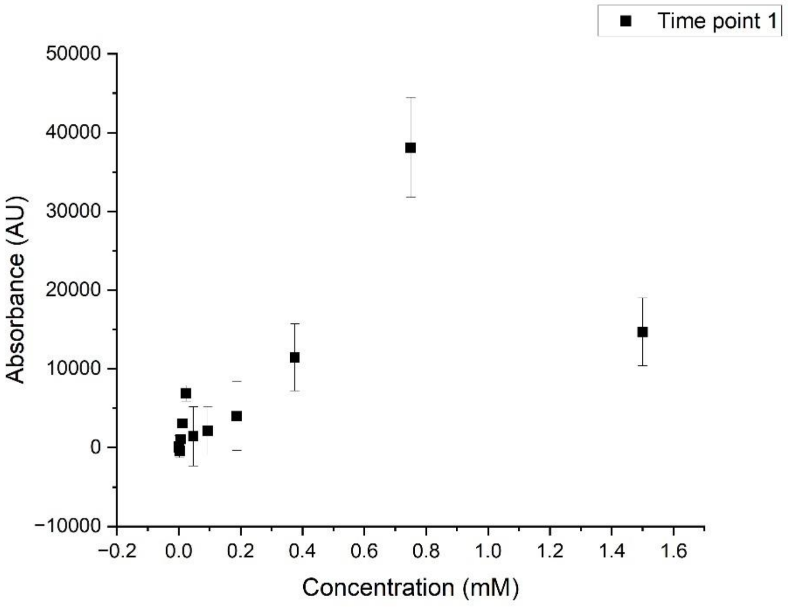

Figure S304 – Graph showing the lysis of calcein loaded PE:PG 3:1 vesicles at 1 minute, with respect to increasing concentration of **h**. Data could not be fitted. Average of n= 3, error = one standard deviation of the mean.

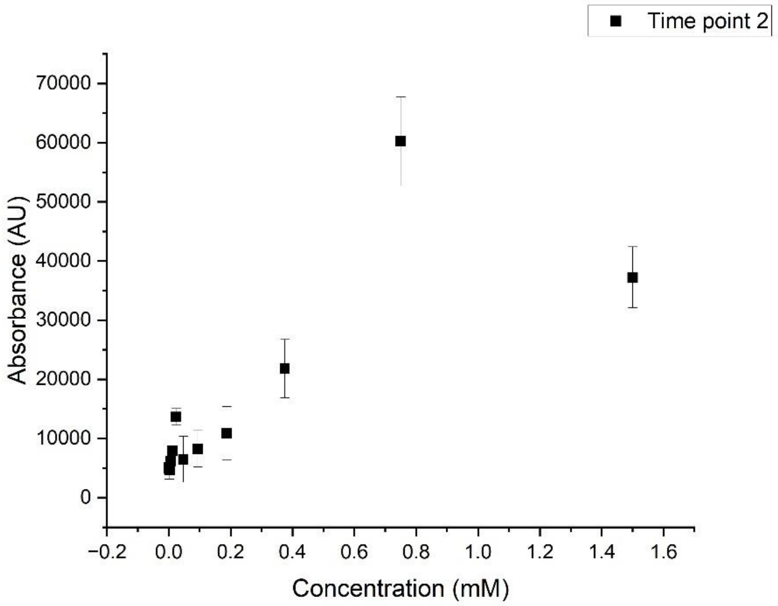

Figure S305 – Graph showing the lysis of calcein loaded PE:PG 3:1 vesicles at 5 minutes, with respect to increasing concentration of **h**. Data could not be fitted. Average of n= 3, error = one standard deviation of the mean.

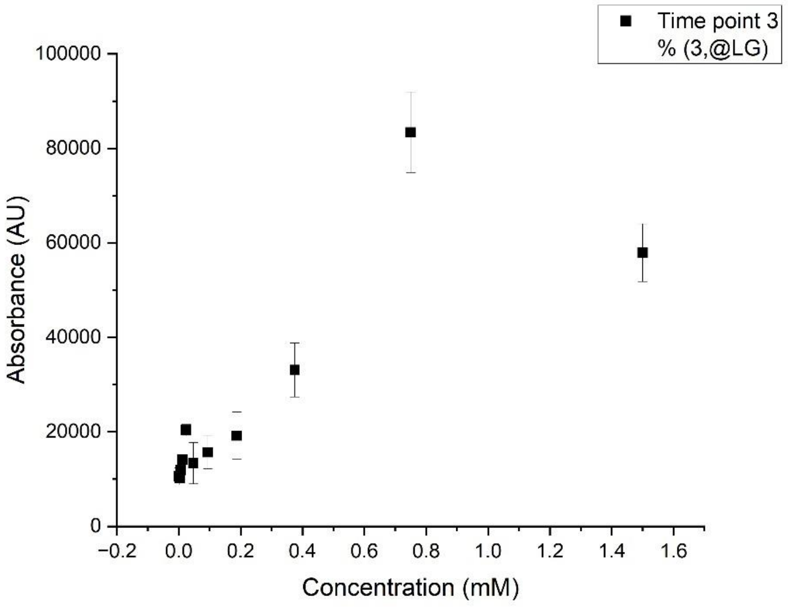

Figure S306 – Graph showing the lysis of calcein loaded PE:PG 3:1 vesicles at 10 minutes, with respect to increasing concentration of **h**. Data could not be fitted. Average of n= 3, error = one standard deviation of the mean.

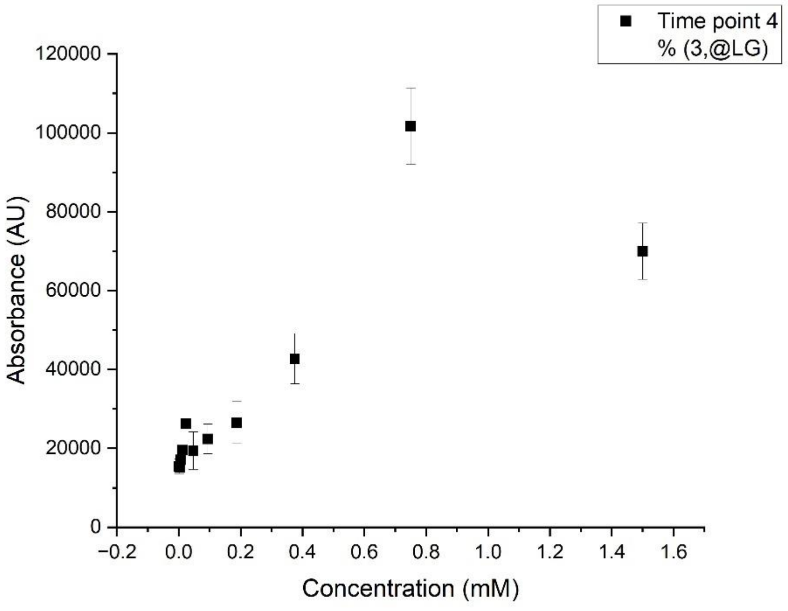

Figure S307 – Graph showing the lysis of calcein loaded PE:PG 3:1 vesicles at 15 minutes, with respect to increasing concentration of **h**. Data could not be fitted. Average of n= 3, error = one standard deviation of the mean.

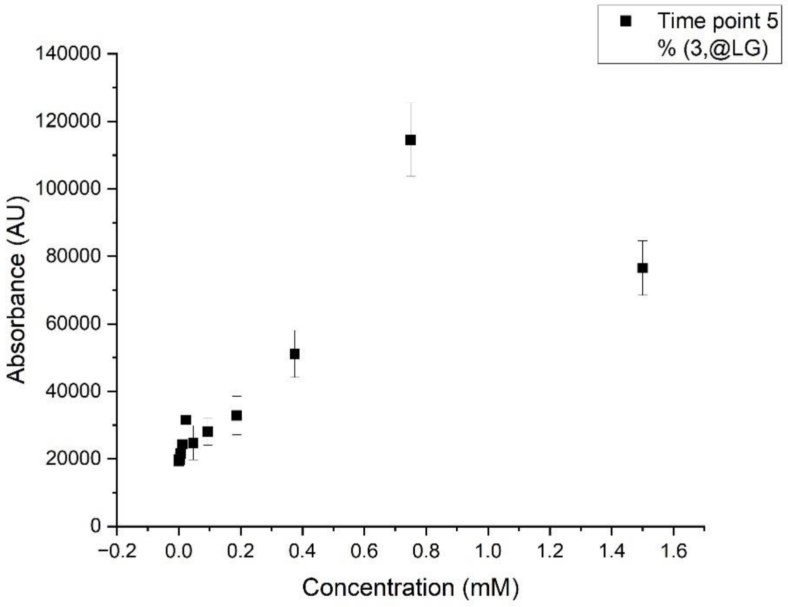

Figure S308 – Graph showing the lysis of calcein loaded PE:PG 3:1 vesicles at 20 minutes, with respect to increasing concentration of **h**. Data could not be fitted. Average of n= 3, error = one standard deviation of the mean.

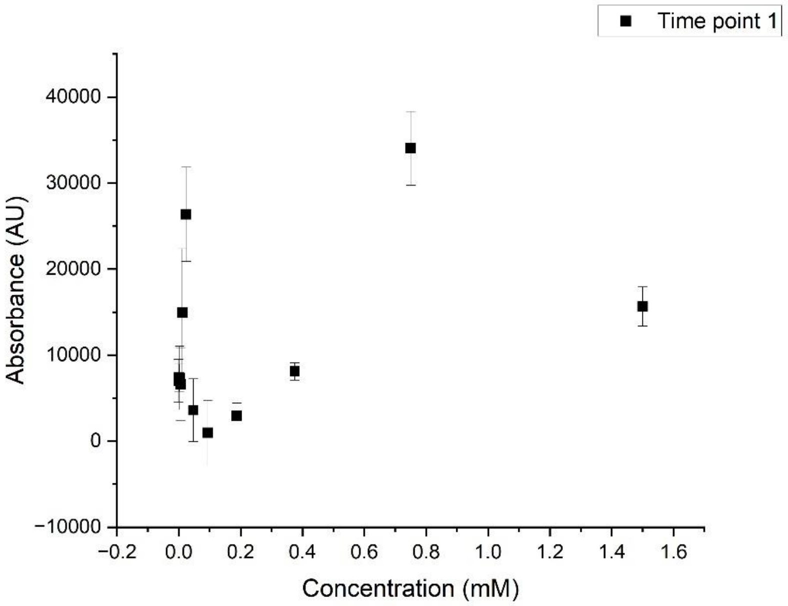

Figure S309 – Graph showing the lysis of calcein loaded PE:PG 3:1 vesicles at 1 minute, with respect to increasing concentration of **i**. Data was could not be fitted. Average of n= 3, error = one standard deviation of the mean.

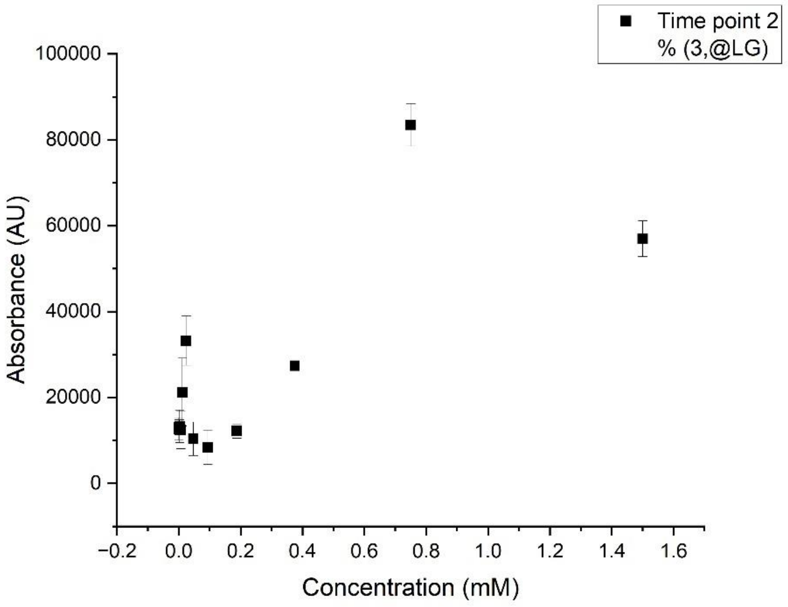

Figure S310 – Graph showing the lysis of calcein loaded PE:PG 3:1 vesicles at 5 minutes, with respect to increasing concentration of **i**. Data could not be fitted. Average of n= 3, error = one standard deviation of the mean.

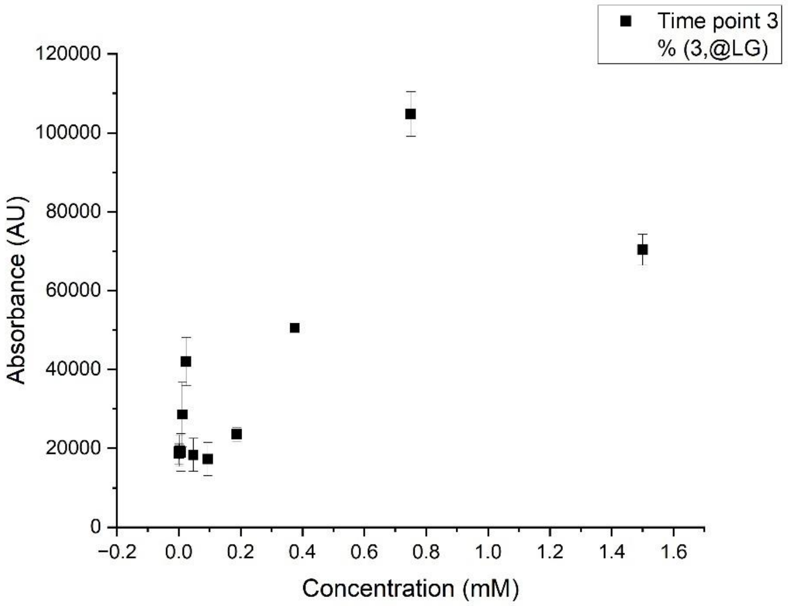

Figure S311 – Graph showing the lysis of calcein loaded PE:PG 3:1 vesicles at 10 minutes, with respect to increasing concentration of **i**. Data could not be fitted. Average of n= 3, error = one standard deviation of the mean.

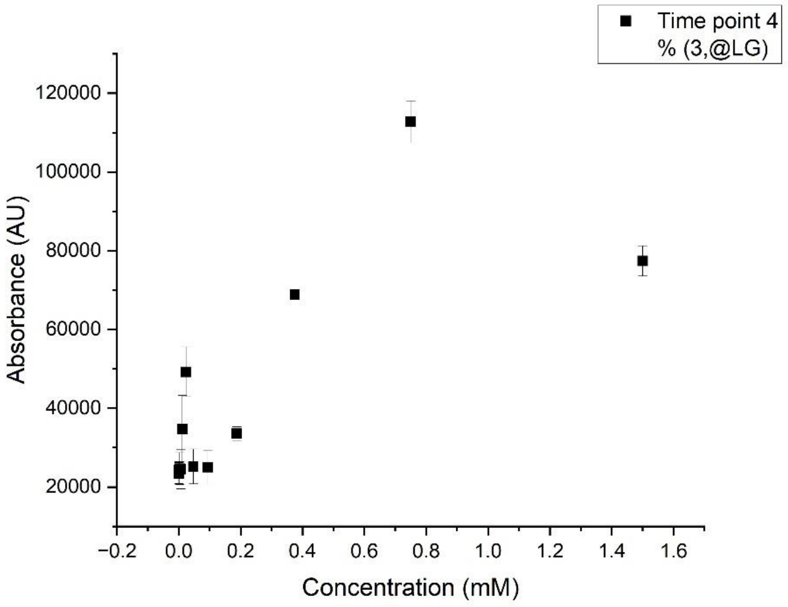

Figure S312 – Graph showing the lysis of calcein loaded PE:PG 3:1 vesicles at 15 minutes, with respect to increasing concentration of **i**. Data could not be fitted. Average of n= 3, error = one standard deviation of the mean.

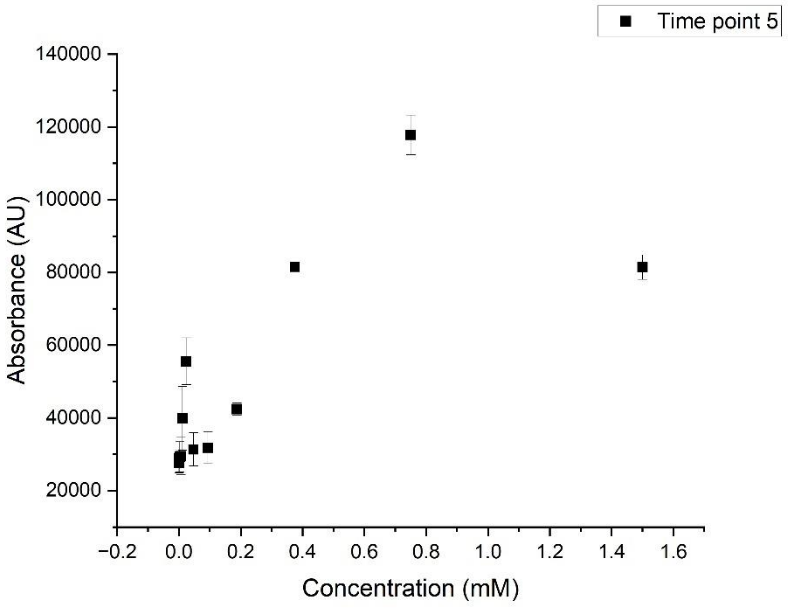

Figure S313 – Graph showing the lysis of calcein loaded PE:PG 3:1 vesicles at 20 minutes, with respect to increasing concentration of **i**. Data could not be fitted. Average of n= 3, error = one standard deviation of the mean.

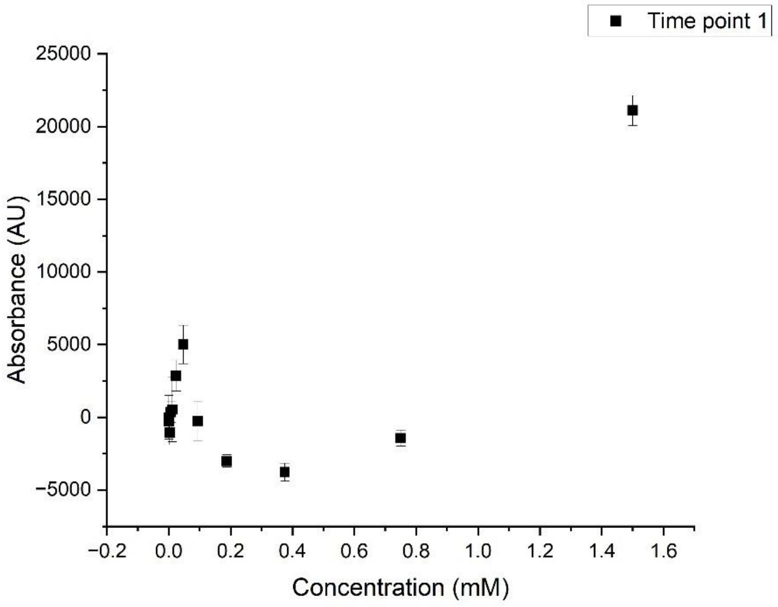

Figure S314 – Graph showing the lysis of calcein loaded PE:PG 3:1 vesicles at 1 minute, with respect to increasing concentration of **j**. Data could not be fitted. Average of n= 3, error = one standard deviation of the mean.

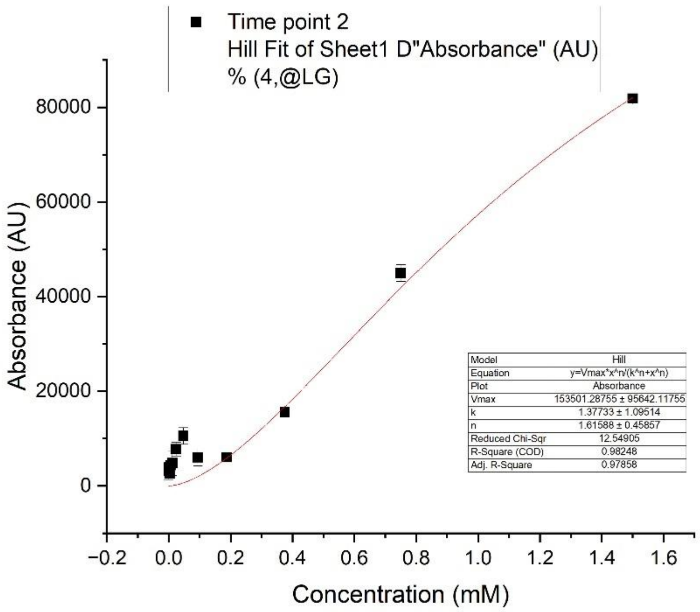

Figure S315 – Graph showing the lysis of calcein loaded PE:PG 3:1 vesicles at 5 minutes, with respect to increasing concentration of **j**. Data was then fitted to the Hill equation. Average of n= 3, error = one standard deviation of the mean.

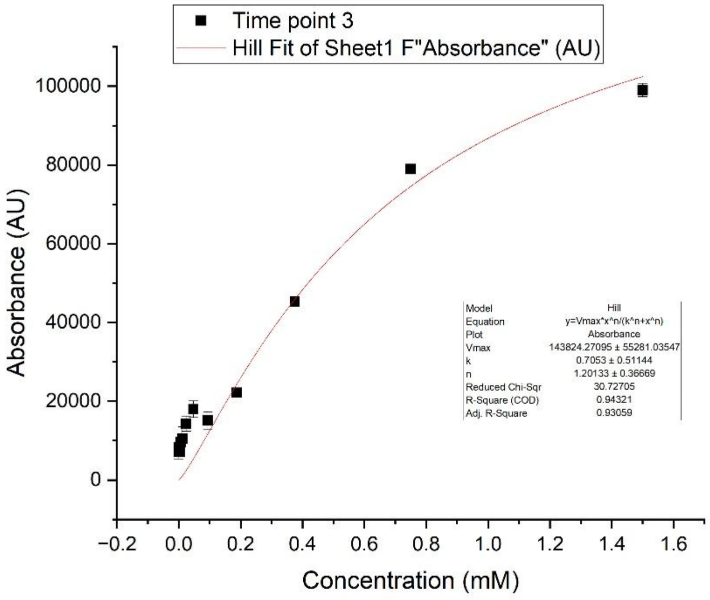

Figure S316 – Graph showing the lysis of calcein loaded PE:PG 3:1 vesicles at 10 minutes, with respect to increasing concentration of **j**. Data was then fitted to the Hill equation. Average of n= 3, error = one standard deviation of the mean.

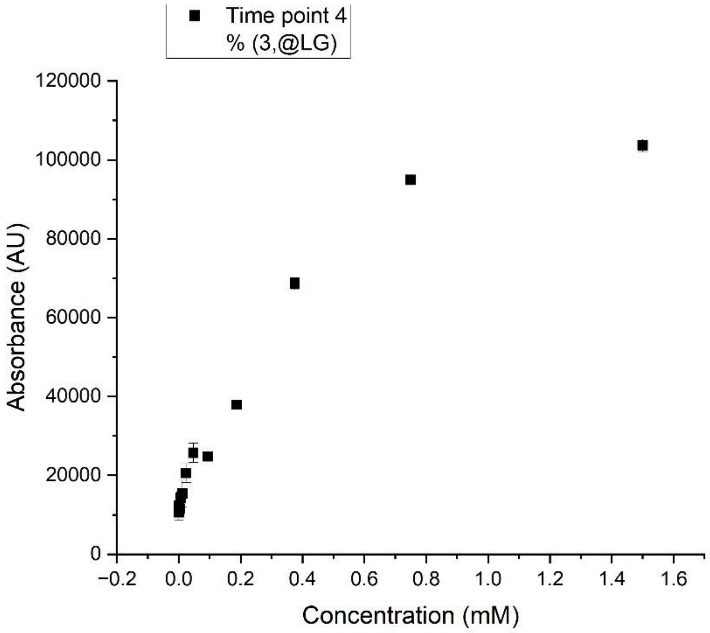

Figure S317 – Graph showing the lysis of calcein loaded PE:PG 3:1 vesicles at 15 minutes, with respect to increasing concentration of **j**. Data could not be fitted. Average of n= 3, error = one standard deviation of the mean.

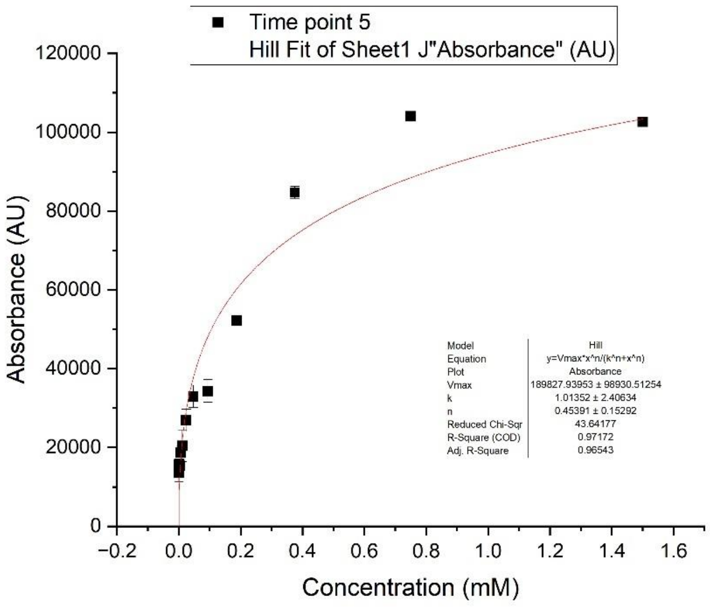

Figure S318 – Graph showing the lysis of calcein loaded PE:PG 3:1 vesicles at 20 minutes, with respect to increasing concentration of **j**. Data was then fitted to the Hill equation. Average of n= 3, error = one standard deviation of the mean.

PE:PG 3:1 vesicle lysis titration summary

Table S13 – Summary of EC_{50}, R^2 and *n* values determined through fitting calcein filled PG:PE 3:1 vesicles to the Hill equation. If a fit to the Hill equation could not be obtained these data were fitted to a linear line of best fit to estimate a EC_{50} value. Where '-' is shown, the data was fitted to a linear line of best fit. *a* – Data not fitted as R^2 values were >0.7.

Compound	Timepoint	EC_{50} (mM)	R^2	*n*	Compound	Timepoint	EC_{50} (mM)	R^2	*n*
	1	0.0302	0.991	1.764		1	0.129	0.989	0.946
	2	0.009	0.992	1.66		2	0.027	0.988	1.344
**1**	3	0.004	0.982	1.663	**d**	3	0.014	0.971	1.057
	4	0.002	0.971	1.824		4	0.007	0.962	1.16
	5	0.002	0.935	1.838		5	0.004	0.941	1.196
	1	> 1.5	0.94	-		1	> 1.5	*a*	-
	2	> 1.5	0.976	-		2	> 1.5	0.753	-
**2**	3	> 1.5	0.934	-	**e**	3	> 1.5	0.881	-
	4	> 1.5	0.958	-		4	> 1.5	0.911	-
	5	1.149	0.978	-		5	1.190	0.903	-
	1	> 1.5	0.897	-		1	> 1.5	*a*	-
	2	> 1.5	0.961	-		2	> 1.5	*a*	-
**3**	3	> 1.5	0.983	-	**f**	3	> 1.5	*a*	-
	4	> 1.5	0.99	-		4	> 1.5	0.856	-
	5	> 1.5	0.985	-		5	0.810	0.876	-
	1	> 1.5	*a*	-		1	> 1.5	*a*	-
	2	> 1.5	*a*	-		2	> 1.5	*a*	-
**4**	3	1.180	0.971	-	**g**	3	1.375	0.864	-
	4	0.950	0.981	-		4	0.798	0.941	-
	5	0.910	0.992	-		5	0.545	0.923	-
	1	> 1.5	*a*	-		1	> 1.5	*a*	-
	2	> 1.5	0.922	1.623		2	> 1.5	*a*	-
**5**	3	0.683	0.987	-	**h**	3	> 1.5	*a*	-
	4	0.540	0.912	-		4	> 1.5	*a*	-
	5	0.429	0.957	0.841		5	> 1.5	*a*	-
	1	0.553	0.914	1.063		1	> 1.5	*a*	-
	2	0.117	0.971	0.996		2	> 1.5	*a*	-
**a**	3	0.073	0.98	0.772	**i**	3	> 1.5	*a*	-
	4	0.089	0.975	0.620		4	> 1.5	*a*	-
	5	0.112	0.984	0.490		5	> 1.5	*a*	-
	1	0.126	0.995	3.195		1	> 1.5	*a*	-
	2	0.045	0.979	2.941		2	1.377	0.982	1.616
**b**	3	0.023	0.966	2.677	**j**	3	0.705	0.943	1.201
	4	0.045	0.945	2.424		4	> 1.5	*a*	-
	5	0.011	0.923	2.292		5	1.014	0.972	0.454
	1	0.102	0.988	1.562					
	2	0.018	0.936	1.076					
**c**	3	0.006	0.839	1.008					
	4	0.004	0.799	0.800					
	5	0.002	0.754	0.779					

PE:PG 1:1 vesicle lysis titrations

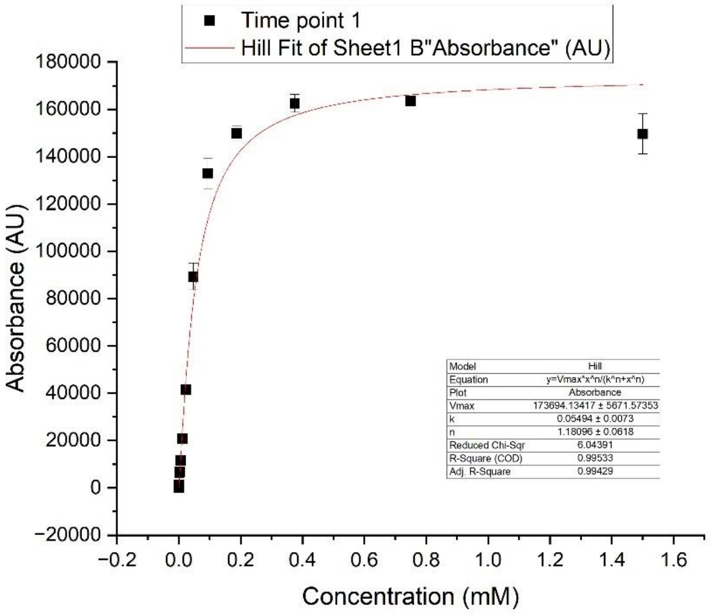

Figure S319 – Graph showing the lysis of calcein loaded PE:PG 1:1 vesicles at 1 minute, with respect to increasing concentration of **1**. Data was then fitted to the Hill equation. Average of n= 3, error = one standard deviation of the mean.

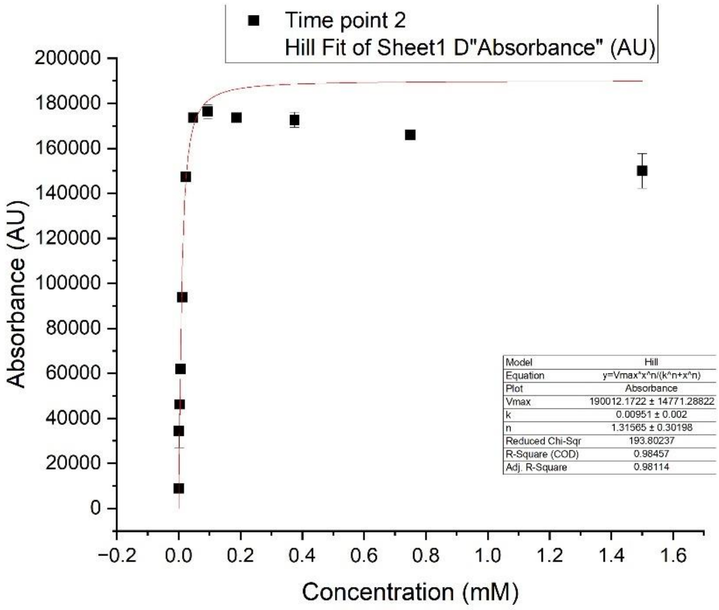

Figure S320 – Graph showing the lysis of calcein loaded PE:PG 1:1 vesicles at 5 minutes, with respect to increasing concentration of **1**. Data was then fitted to the Hill equation. Average of n= 3, error = one standard deviation of the mean.

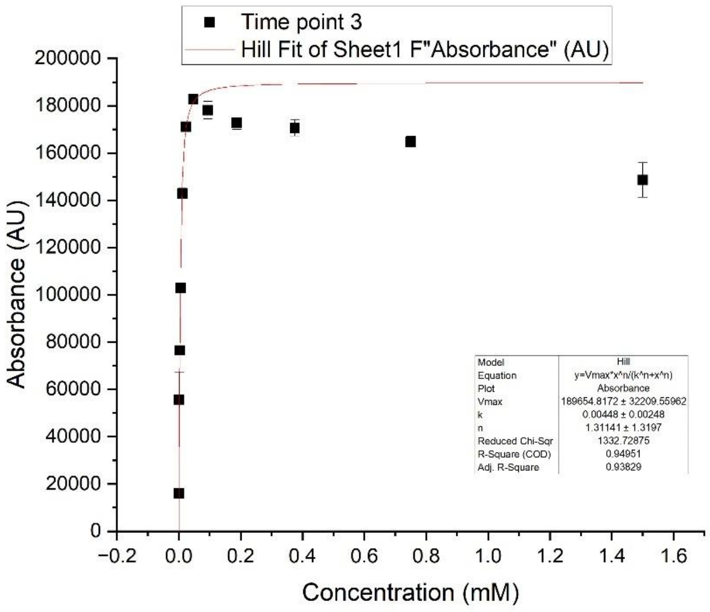

Figure S321 – Graph showing the lysis of calcein loaded PE:PG 1:1 vesicles at 10 minutes, with respect to increasing concentration of **1**. Data was then fitted to the Hill equation. Average of n= 3, error = one standard deviation of the mean.

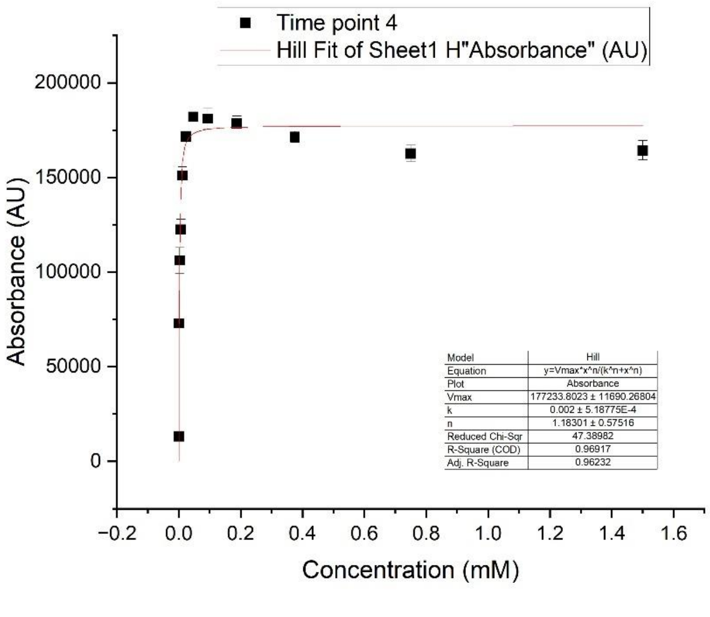

Figure S322 – Graph showing the lysis of calcein loaded PE:PG 1:1 vesicles at 15 minutes, with respect to increasing concentration of **1**. Data was then fitted to the Hill equation. Average of n= 3, error = one standard deviation of the mean.

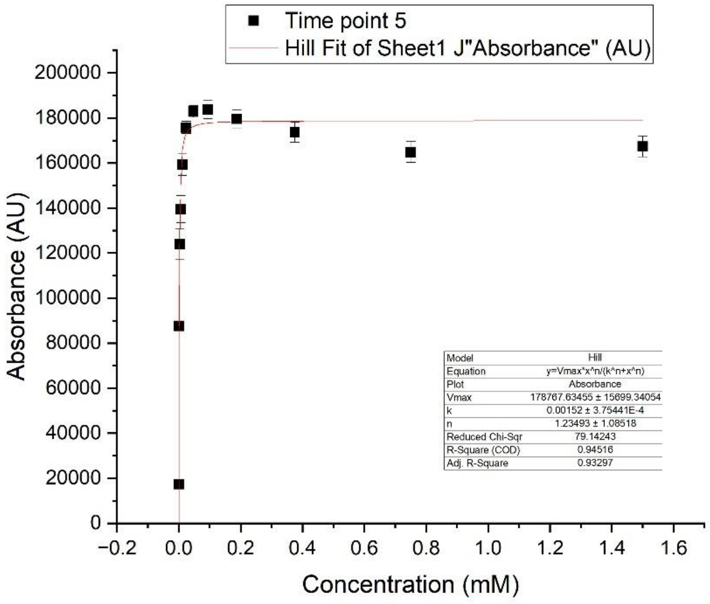

Figure S323 – Graph showing the lysis of calcein loaded PE:PG 1:1 vesicles at 20 minutes, with respect to increasing concentration of **1**. Data was then fitted to the Hill equation. Average of n= 3, error = one standard deviation of the mean.

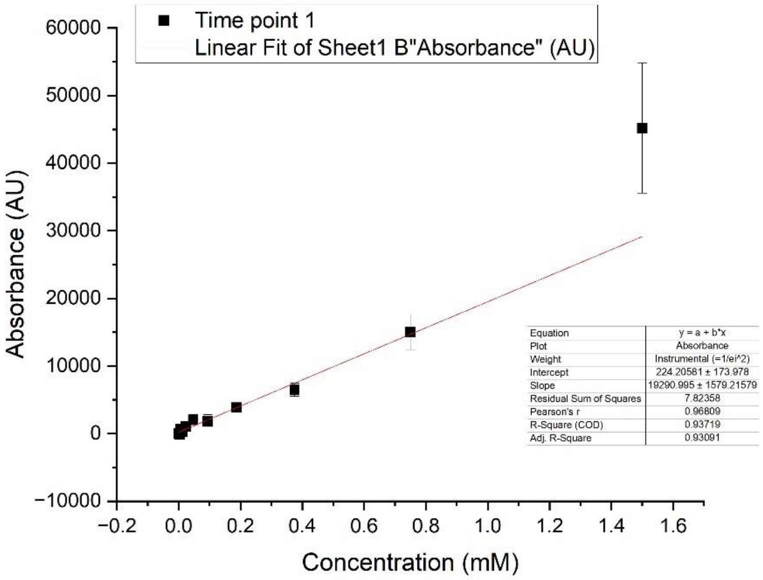

Figure S324 – Graph showing the lysis of calcein loaded PE:PG 1:1 vesicles at 1 minute, with increasing concentration of **2**. Data was then fitted to a linear line of best fit. Average of n= 3, error = one stand

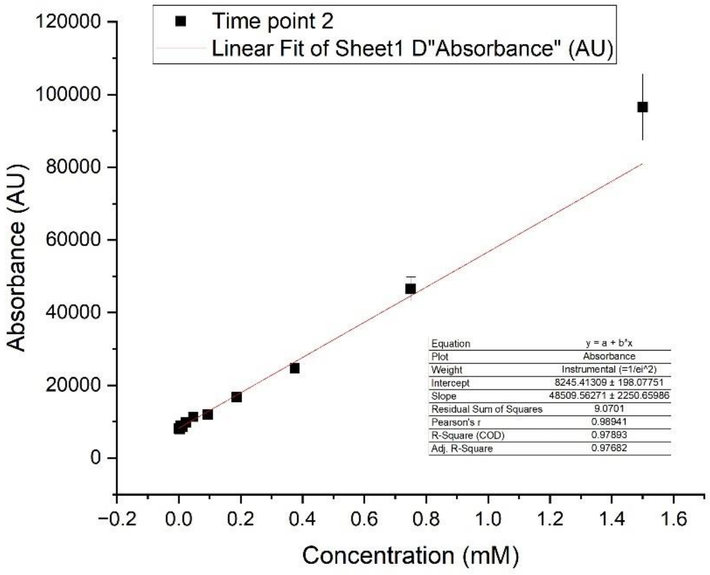

Figure S325 – Graph showing the lysis of calcein loaded PE:PG 1:1 vesicles at 5 minutes, with respect to increasing concentration of **2**. Data was then fitted to a linear line of best fit. Average of n= 3, error = one standard deviation of the mean.

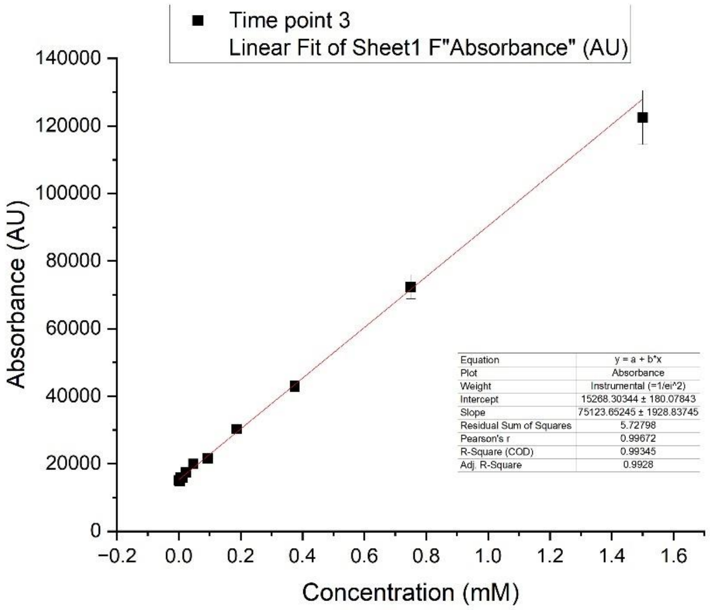

Figure S326 – Graph showing the lysis of calcein loaded PE:PG 1:1 vesicles at 10 minutes, with respect to increasing concentration of **2**. Data was then fitted to a linear line of best fit. Average of n= 3, error = one standard deviation of the mean.

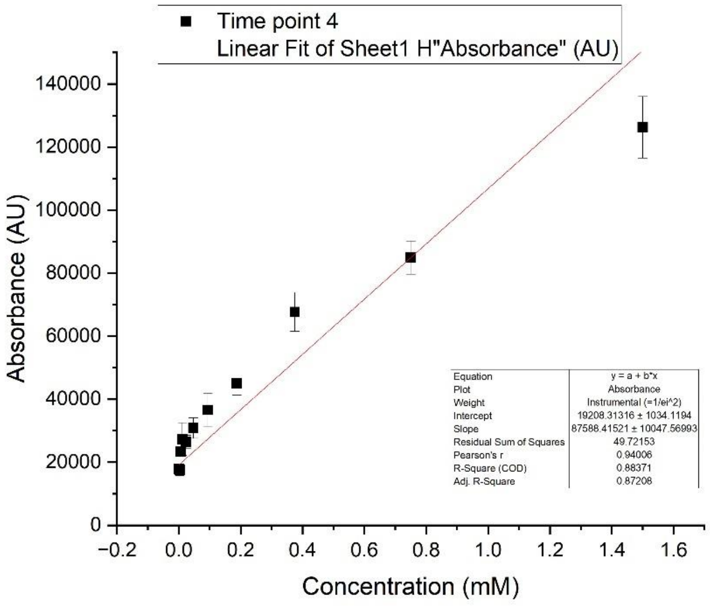

Figure S327 – Graph showing the lysis of calcein loaded PE:PG 1:1 vesicles at 15 minutes, with respect to increasing concentration of **2**. Data was then fitted to a linear line of best fit. Average of n= 3, error = one standard deviation of the mean.

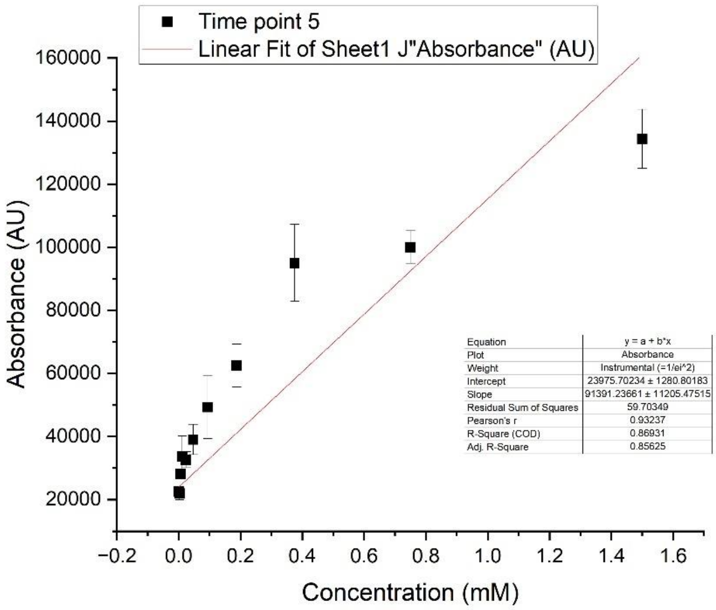

Figure S328 – Graph showing the lysis of calcein loaded PE:PG 1:1 vesicles at 20 minutes, with respect to increasing concentration of **2**. Data was then fitted to a linear line of best fit. Average of n= 3, error = one standard deviation of the mean.

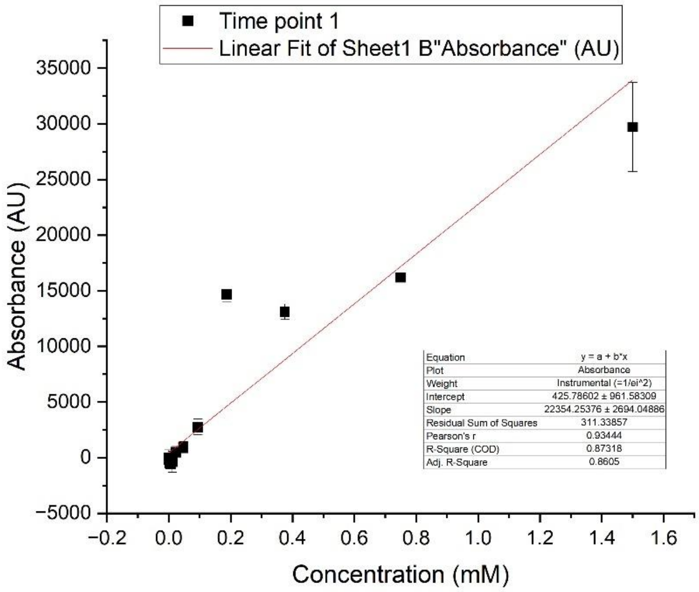

Figure S329 – Graph showing the lysis of calcein loaded PE:PG 1:1 vesicles at 1 minute, with respect to increasing concentration of **3**. Data was then fitted to a linear line of best fit. Average of n= 3, error = one standard deviation of the mean.

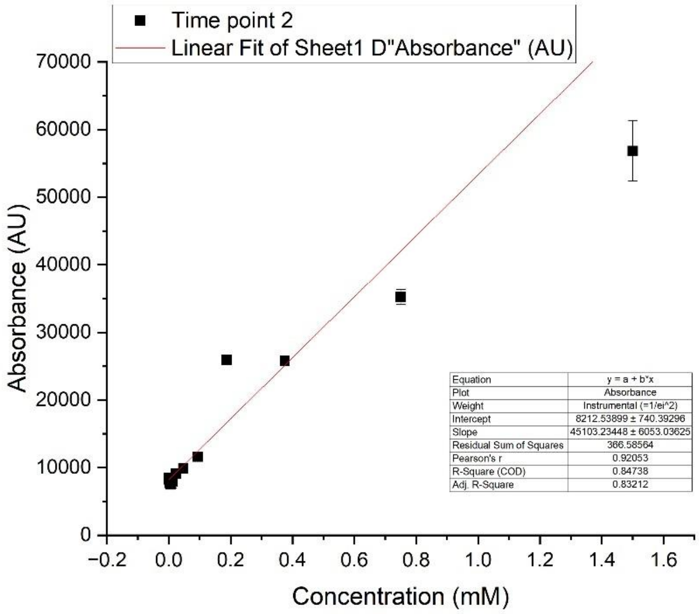

Figure S330 – Graph showing the lysis of calcein loaded PE:PG 1:1 vesicles at 5 minutes, with respect to increasing concentration of **3**. Data was then fitted to a linear line of best fit. Average of n= 3, error = one standard deviation of the mean.

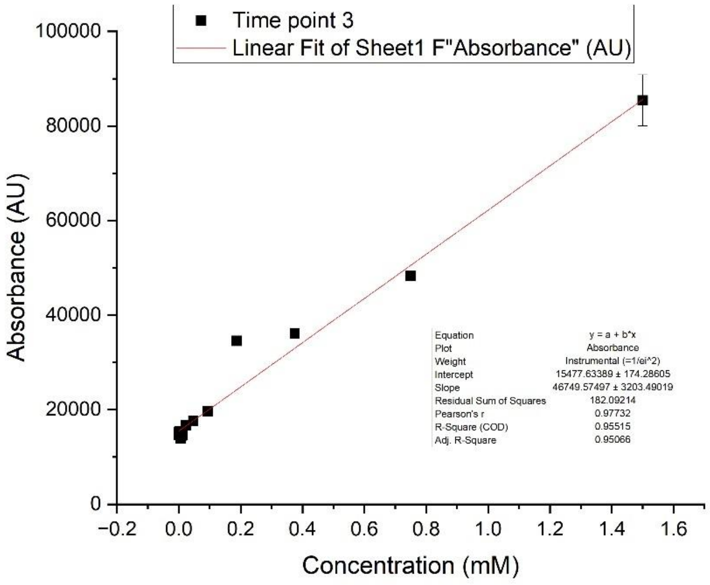

Figure S331 – Graph showing the lysis of calcein loaded PE:PG 1:1 vesicles at 10 minutes, with respect to increasing concentration of **3**. Data was then fitted to a linear line of best fit. Average of n= 3, error = one standard deviation of the mean.

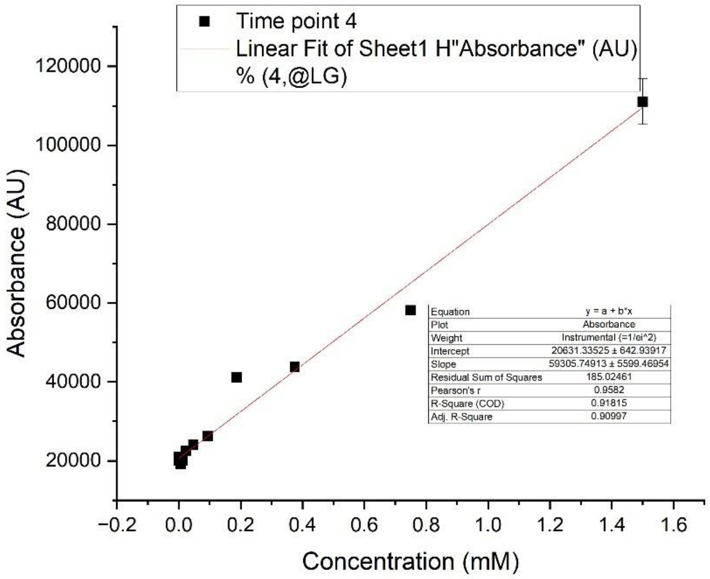

Figure S332 – Graph showing the lysis of calcein loaded PE:PG 1:1 vesicles at 15 minutes, with respect to increasing concentration of **3**. Data was then fitted to a linear line of best fit. Average of n= 3, error = one standard deviation of the mean.

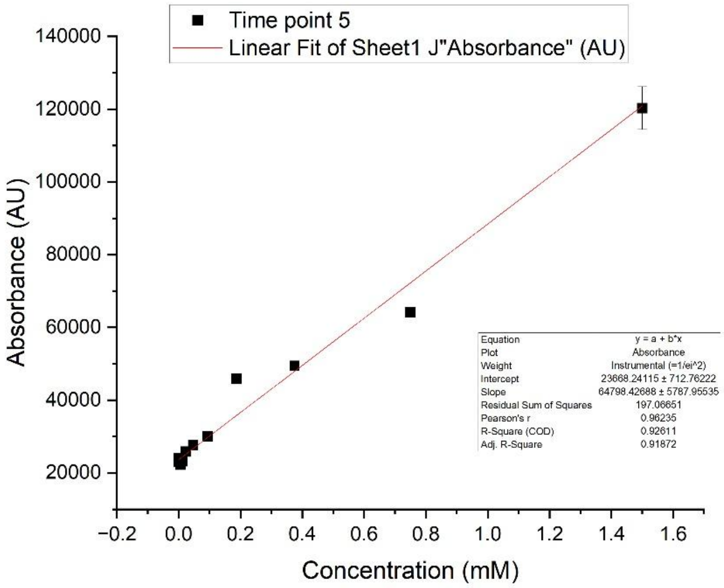

Figure S333 – Graph showing the lysis of calcein loaded PE:PG 1:1 vesicles at 20 minutes, with respect to increasing concentration of **3**. Data was then fitted to a linear line of best fit. Average of n= 3, error = one standard deviation of the mean.

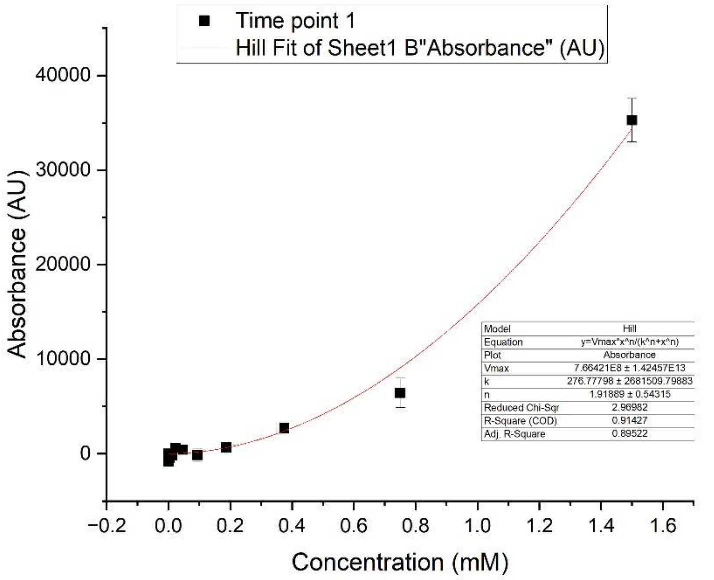

Figure S334 – Graph showing the lysis of calcein loaded PE:PG 1:1 vesicles at 1 minute, with respect to increasing concentration of **4**. Data was then fitted to the Hill equation. Average of n= 3, error = one standard deviation of the mean.

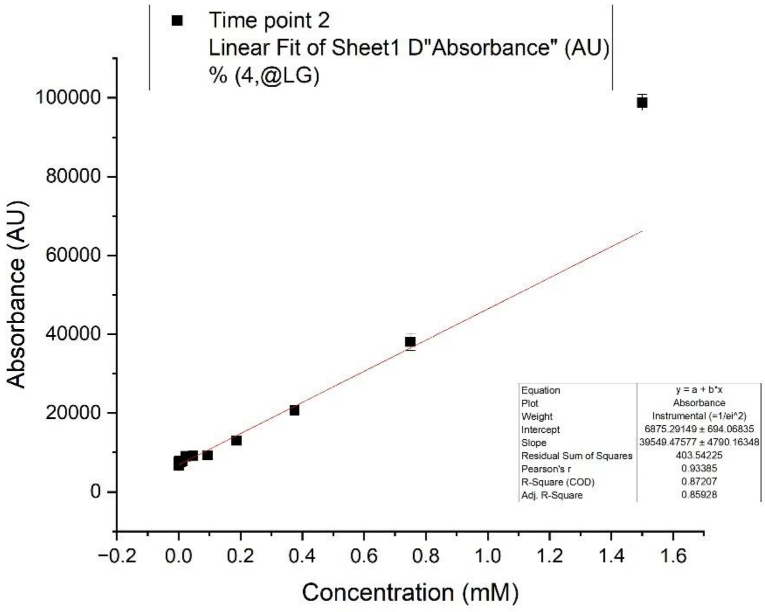

Figure S335 – Graph showing the lysis of calcein loaded PE:PG 1:1 vesicles at 5 minutes, with respect to increasing concentration of **4**. Data was then fitted to a linear line of best fit. Average of n= 3, error = one standard deviation of the mean.

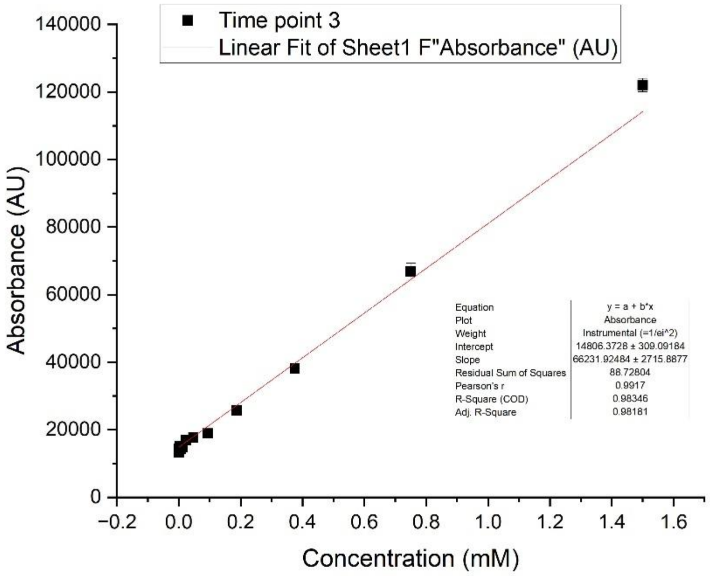

Figure S336 – Graph showing the lysis of calcein loaded PE:PG 1:1 vesicles at 10 minutes, with respect to increasing concentration of **4**. Data was then fitted to a linear line of best fit. Average of n= 3, error = one standard deviation of the mean.

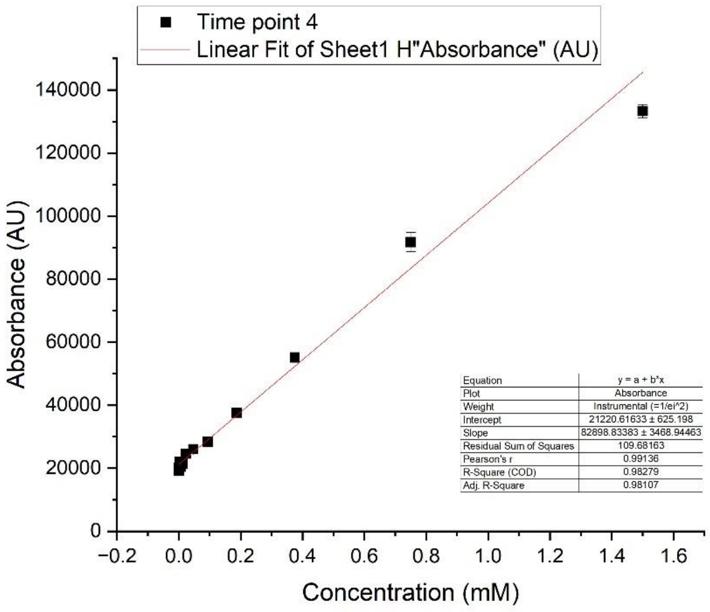

Figure S337 – Graph showing the lysis of calcein loaded PE:PG 1:1 vesicles at 15 minutes, with respect to increasing concentration of **4**. Data was then fitted to a linear line of best fit. Average of n= 3, error = one standard deviation of the mean.

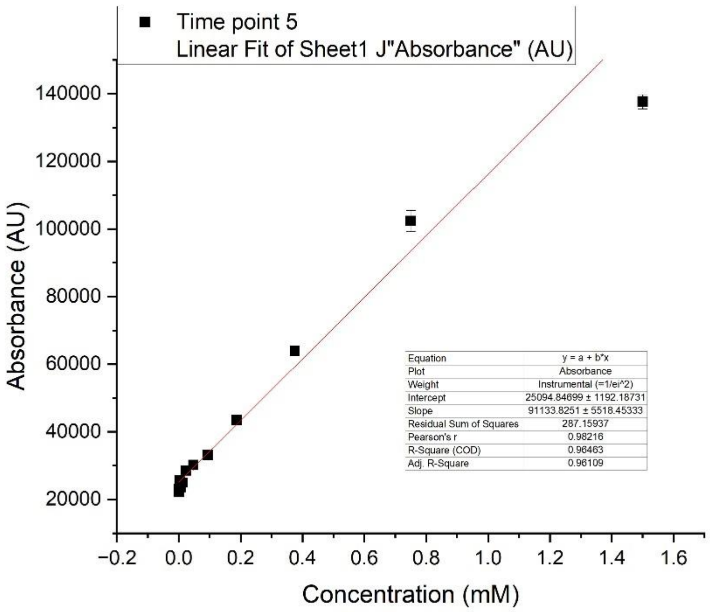

Figure S338 – Graph showing the lysis of calcein loaded PE:PG 1:1 vesicles at 20 minutes, with respect to increasing concentration of **4**. Data was then fitted to a linear line of best fit. Average of n= 3, error = one standard deviation of the mean.

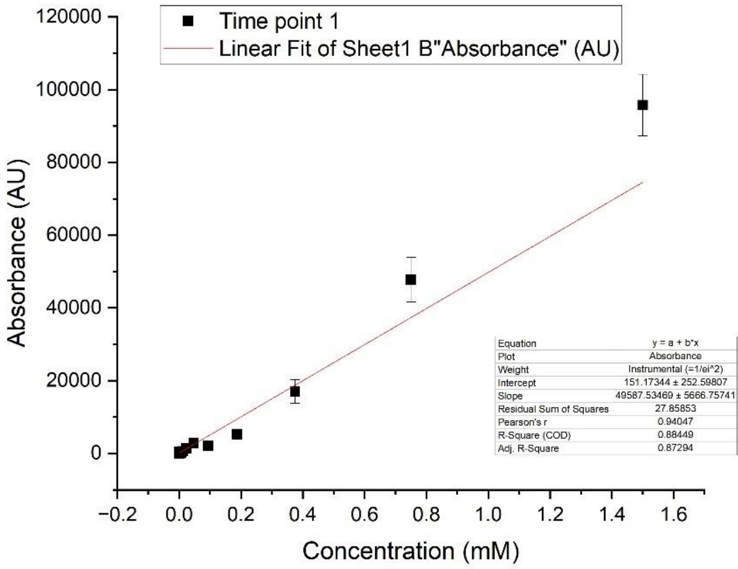

Figure S339 – Graph showing the lysis of calcein loaded PE:PG 1:1 vesicles at 1 minute, with respect to increasing concentration of **5**. Data was then fitted to a linear line of best fit. Average of n= 3, error = one standard deviation of the mean.

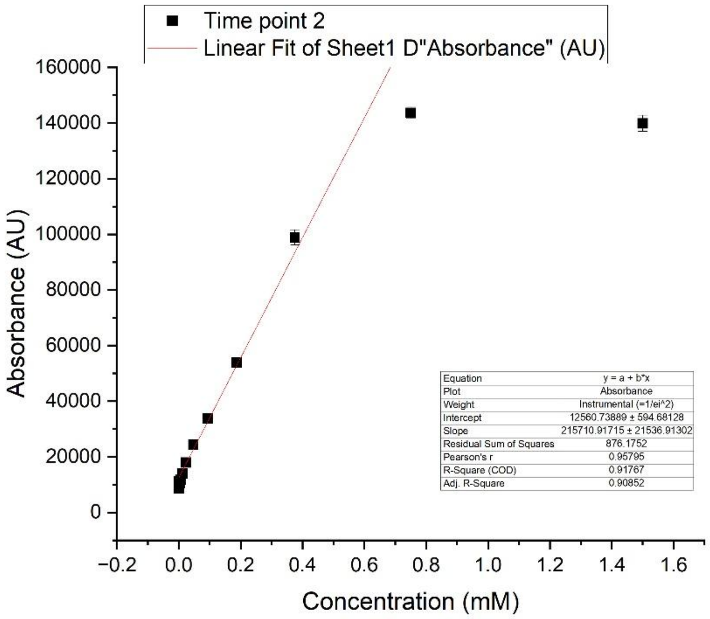

Figure S340 – Graph showing the lysis of calcein loaded PE:PG 1:1 vesicles at 5 minutes, with respect to increasing concentration of **5**. Data was then fitted to a linear line of best fit. Average of n= 3, error = one standard deviation of the mean.

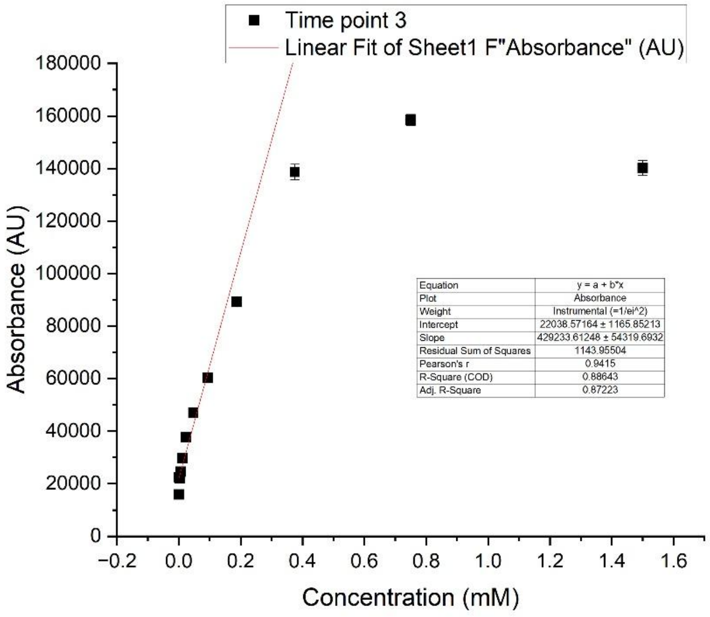

Figure S341 – Graph showing the lysis of calcein loaded PE:PG 1:1 vesicles at 10 minutes, with respect to increasing concentration of **5**. Data was then fitted to a linear line of best fit. Average of n= 3, error = one standard deviation of the mean.

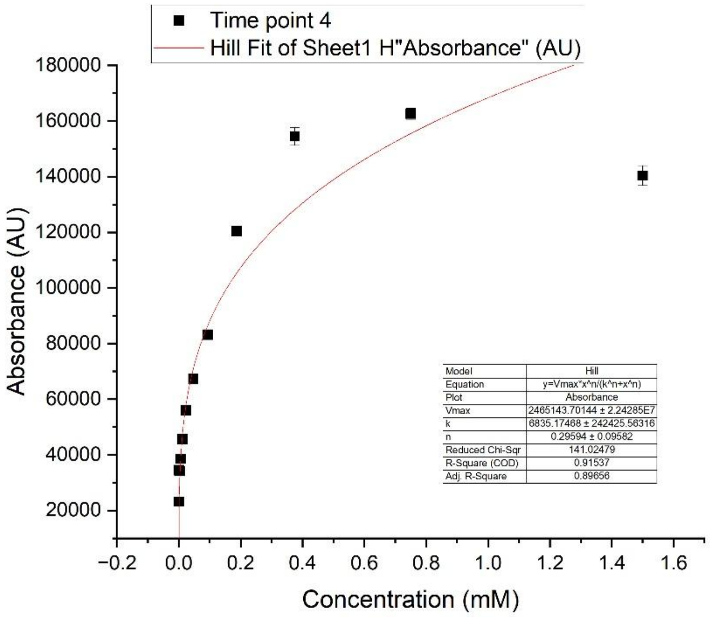

Figure S342 – Graph showing the lysis of calcein loaded PE:PG 1:1 vesicles at 15 minutes, with respect to increasing concentration of **5**. Data was then fitted to the Hill equation. Average of n= 3, error = one standard deviation of the mean.

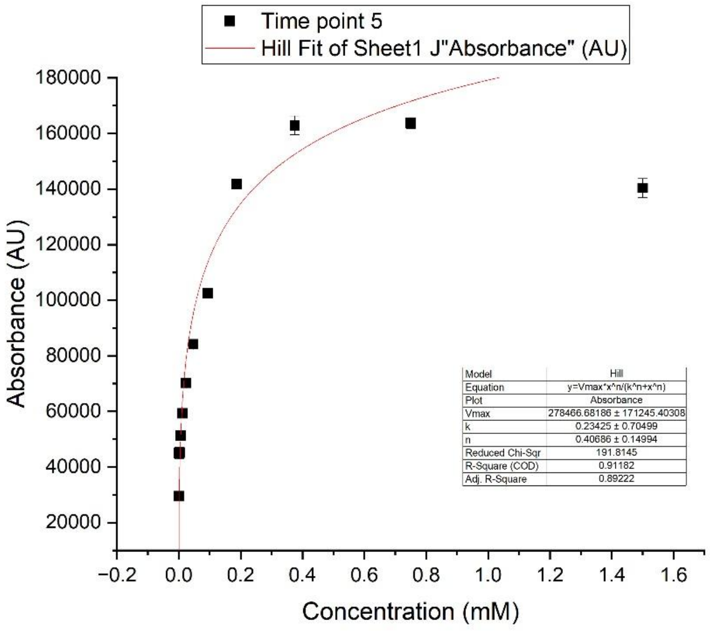

Figure S343 – Graph showing the lysis of calcein loaded PE:PG 1:1 vesicles at 20 minutes, with respect to increasing concentration of **5**. Data was then fitted to the Hill equation. Average of n= 3, error = one standard deviation of the mean.

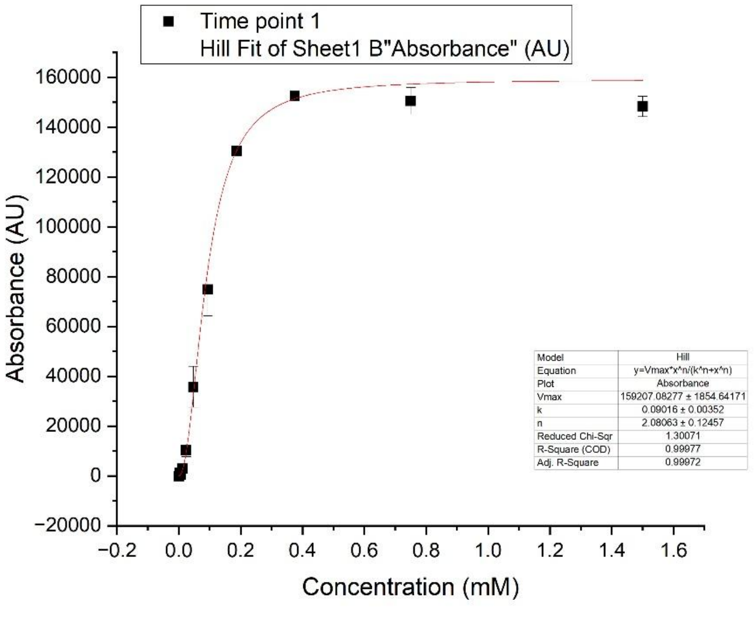

Figure S344 – Graph showing the lysis of calcein loaded PE:PG 1:1 vesicles at 1 minute, with respect to increasing concentration of **a**. Data was then fitted to the Hill equation. Average of n= 3, error = one standard deviation of the mean.

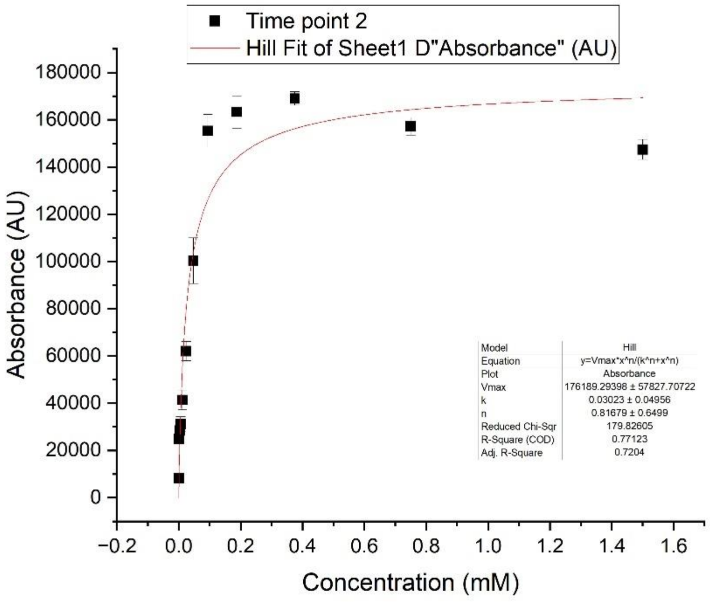

Figure S345 – Graph showing the lysis of calcein loaded PE:PG 1:1 vesicles at 5 minutes, with respect to increasing concentration of **a**. Data was then fitted to the Hill equation. Average of n= 3, error = one standard deviation of the mean.

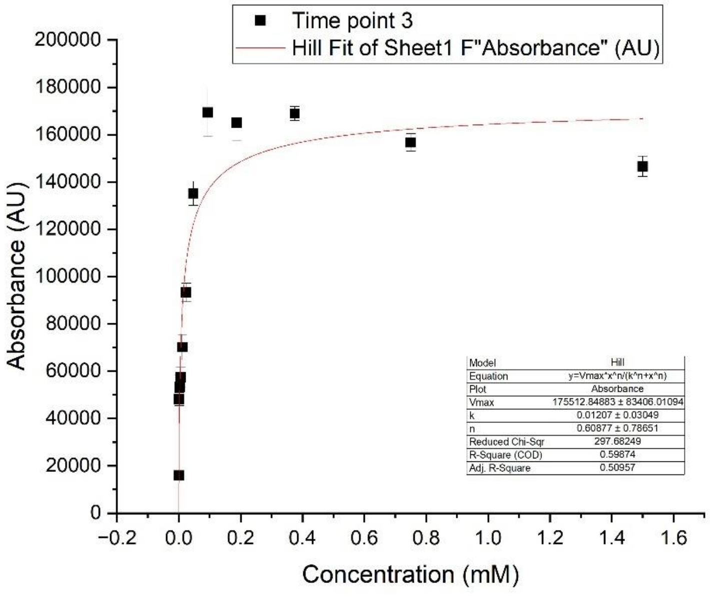

Figure S346 – Graph showing the lysis of calcein loaded PE:PG 1:1 vesicles at 10 minutes, with respect to increasing concentration of **a**. Data was then fitted to the Hill equation. Average of n= 3, error = one standard deviation of the mean.

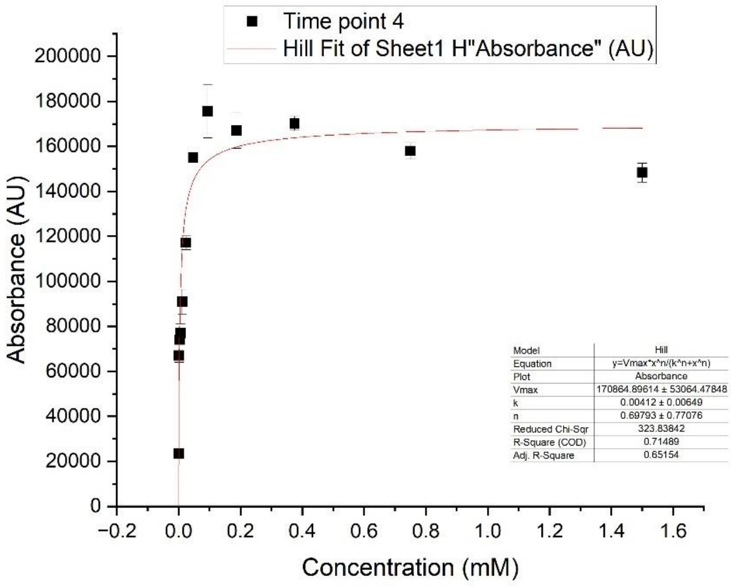

Figure S347 – Graph showing the lysis of calcein loaded PE:PG 1:1 vesicles at 15 minutes, with respect to increasing concentration of **a**. Data was then fitted to the Hill equation. Average of n= 3, error = one standard deviation of the mean.

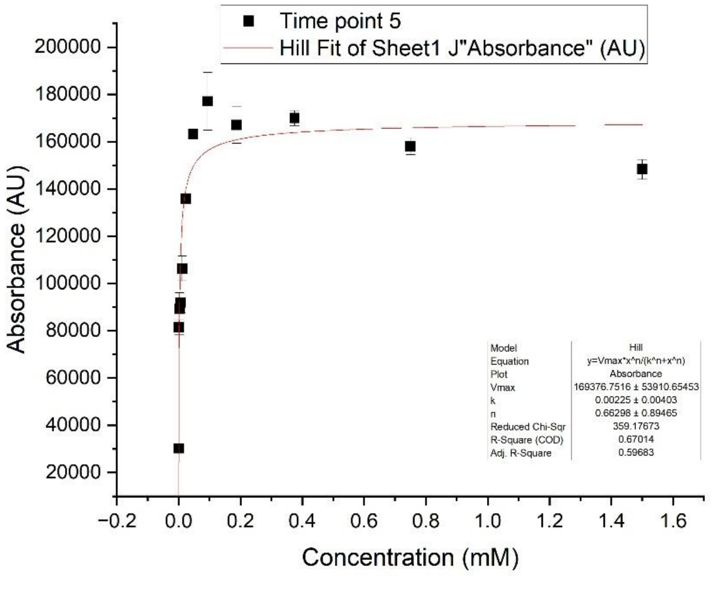

Figure S348 – Graph showing the lysis of calcein loaded PE:PG 1:1 vesicles at 20 minutes, with respect to increasing concentration of **a**. Data was then fitted to the Hill equation. Average of n= 3, error = one standard deviation of the mean.

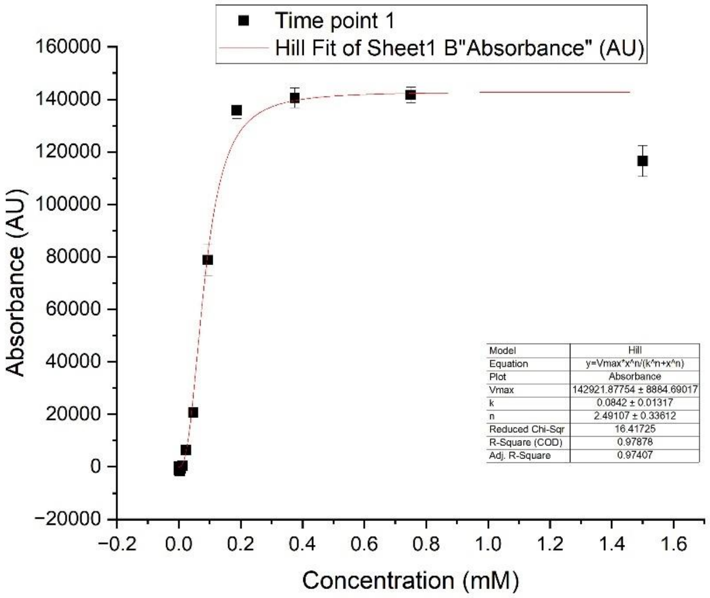

Figure S349 – Graph showing the lysis of calcein loaded PE:PG 1:1 vesicles at 1 minute, with respect to increasing concentration of **b**. Data was then fitted to the Hill equation. Average of n= 3, error = one standard deviation of the mean.

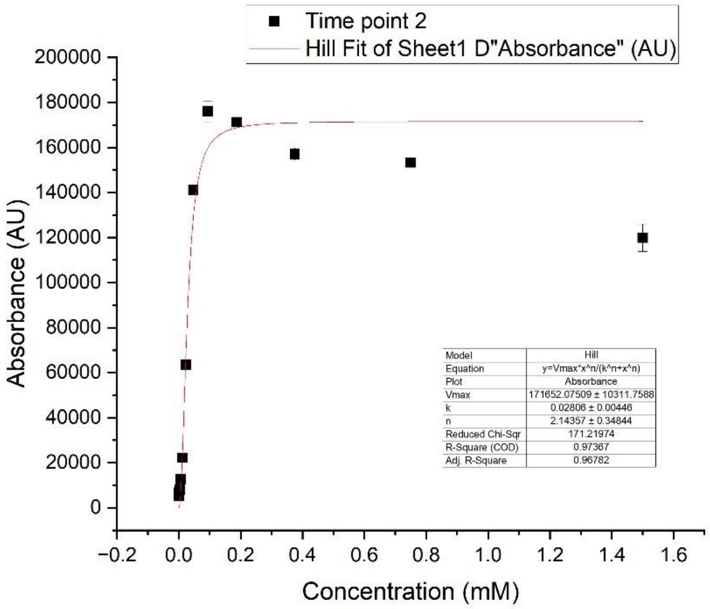

Figure S350 – Graph showing the lysis of calcein loaded PE:PG 1:1 vesicles at 5 minutes, with respect to increasing concentration of **b**. Data was then fitted to the Hill equation. Average of n= 3, error = one standard deviation of the mean.

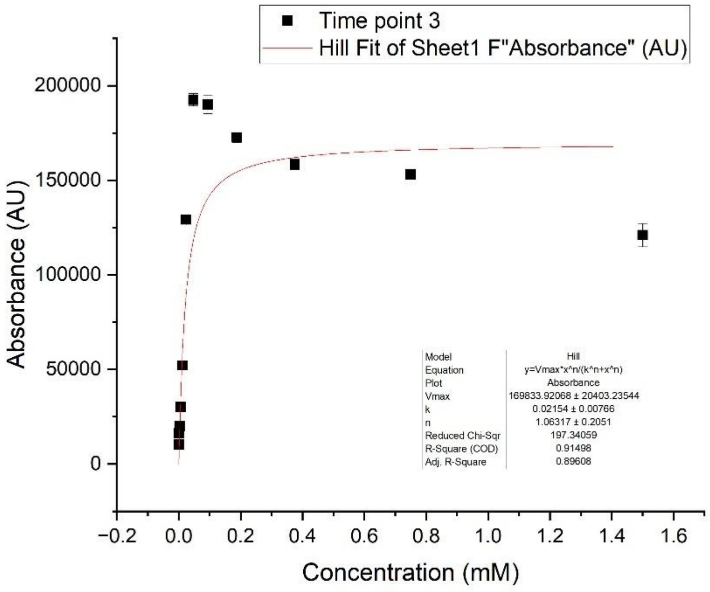

Figure S351 – Graph showing the lysis of calcein loaded PE:PG 1:1 vesicles at 10 minutes, with respect to increasing concentration of **b**. Data was then fitted to the Hill equation. Average of n= 3, error = one standard deviation of the mean.

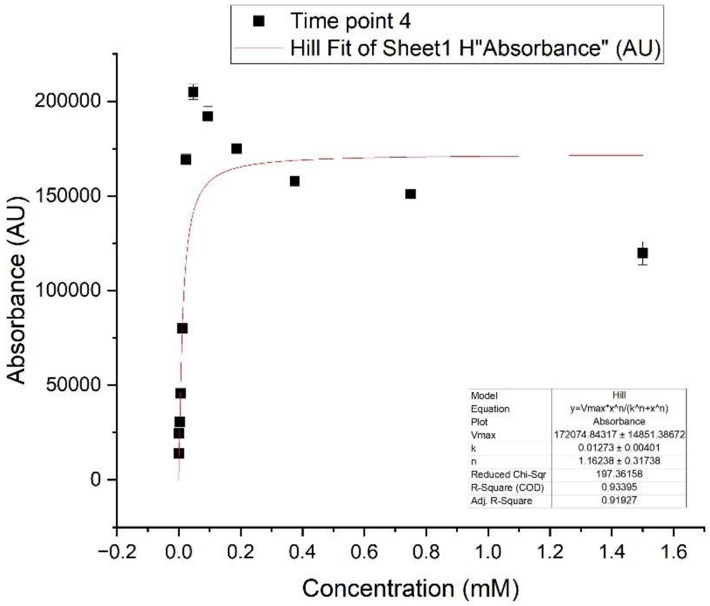

Figure S352 – Graph showing the lysis of calcein loaded PE:PG 1:1 vesicles at 15 minutes, with respect to increasing concentration of **b**. Data was then fitted to the Hill equation. Average of n= 3, error = one standard deviation of the mean.

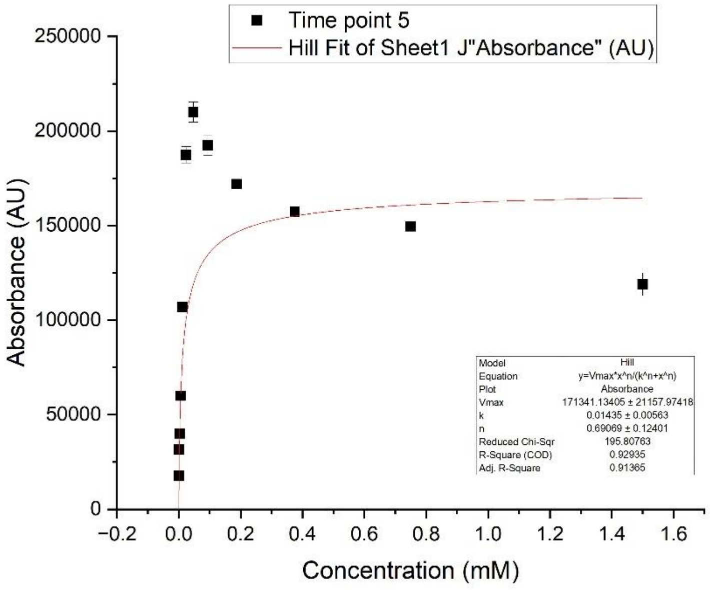

Figure S353 – Graph showing the lysis of calcein loaded PE:PG 1:1 vesicles at 20 minutes, with respect to increasing concentration of **b**. Data was then fitted to the Hill equation. Average of n= 3, error = one standard deviation of the mean.

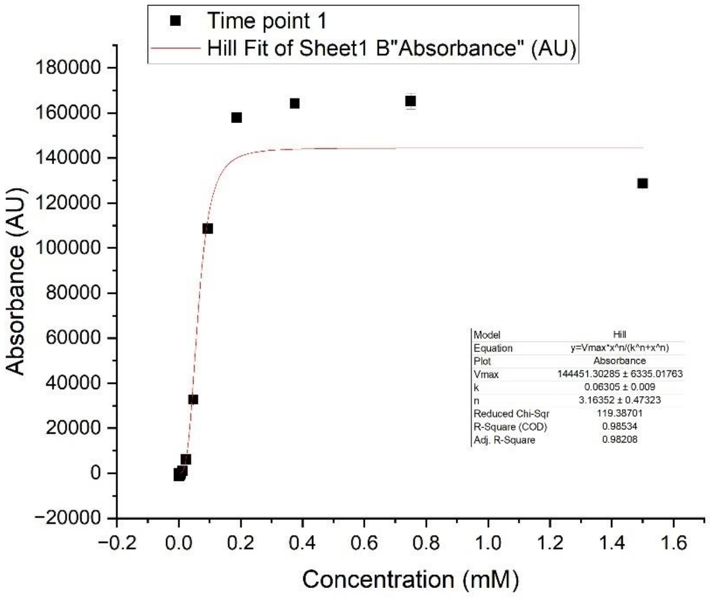

Figure S354 – Graph showing the lysis of calcein loaded PE:PG 1:1 vesicles at 1 minute, with respect to increasing concentration of **c**. Data was then fitted to the Hill equation. Average of n= 3, error = one standard deviation of the mean.

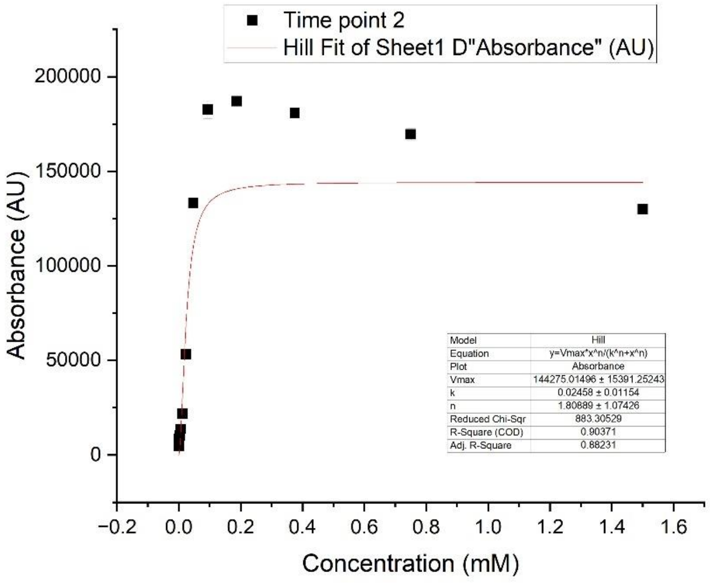

Figure S355 – Graph showing the lysis of calcein loaded PE:PG 1:1 vesicles at 5 minutes, with respect to increasing concentration of **c**. Data was then fitted to the Hill equation. Average of n= 3, error = one standard deviation of the mean.

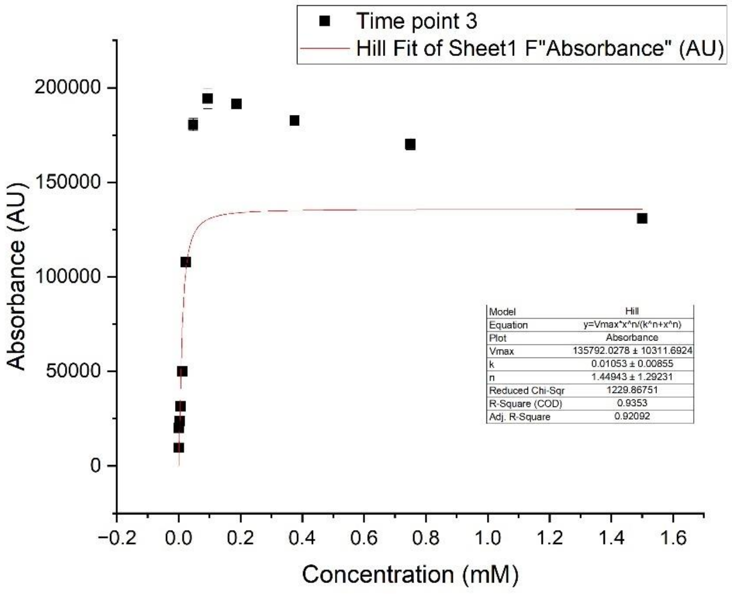

Figure S356 – Graph showing the lysis of calcein loaded PE:PG 1:1 vesicles at 10 minutes, with respect to increasing concentration of **c**. Data was then fitted to the Hill equation. Average of n= 3, error = one standard deviation of the mean.

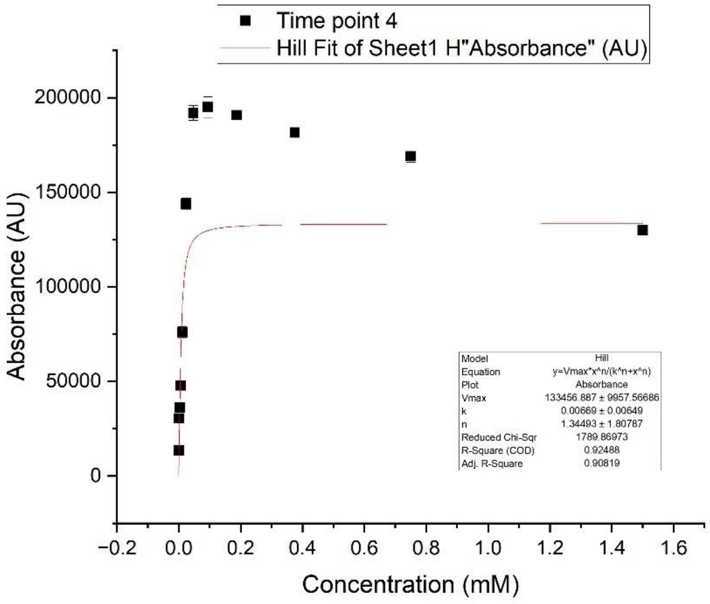

Figure S357 – Graph showing the lysis of calcein loaded PE:PG 1:1 vesicles at 15 minutes, with respect to increasing concentration of **c**. Data was then fitted to the Hill equation. Average of n= 3, error = one standard deviation of the mean.

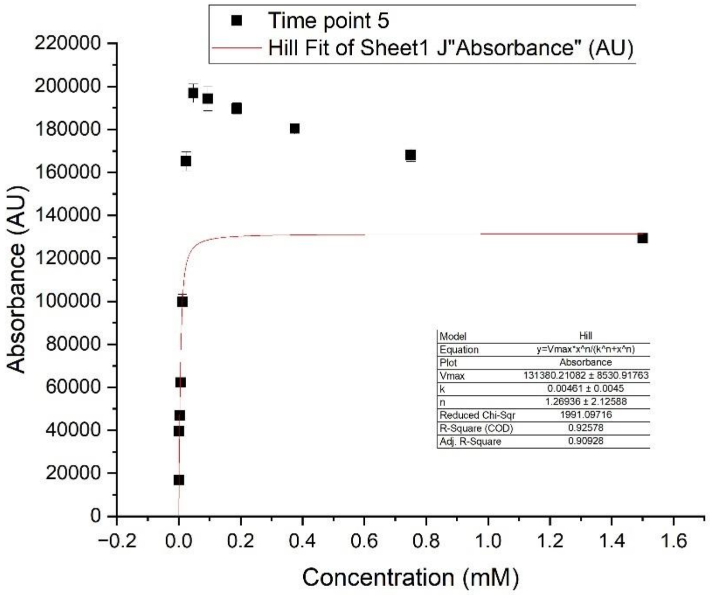

Figure S358 – Graph showing the lysis of calcein loaded PE:PG 1:1 vesicles at 20 minutes, with respect to increasing concentration of **c**. Data was then fitted to the Hill equation. Average of n= 3, error = one standard deviation of the mean.

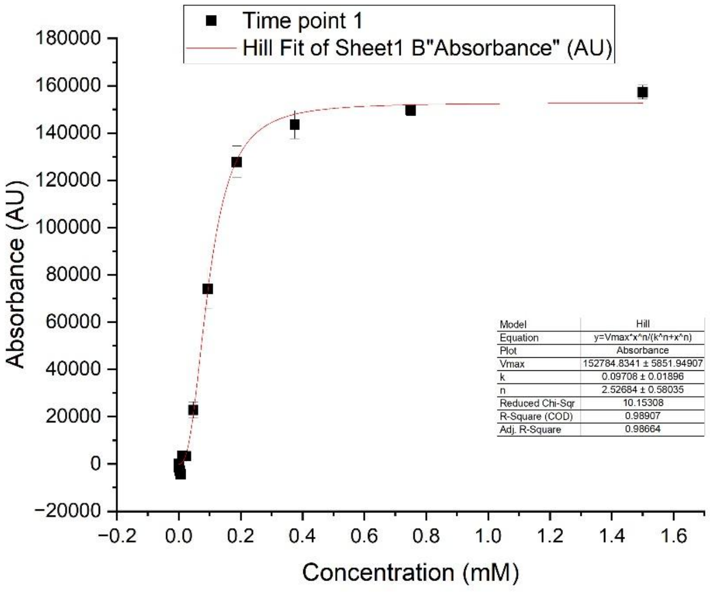

Figure S359 – Graph showing the lysis of calcein loaded PE:PG 1:1 vesicles at 1 minute, with respect to increasing concentration of **d**. Data was then fitted to the Hill equation. Average of n= 3, error = one standard deviation of the mean.

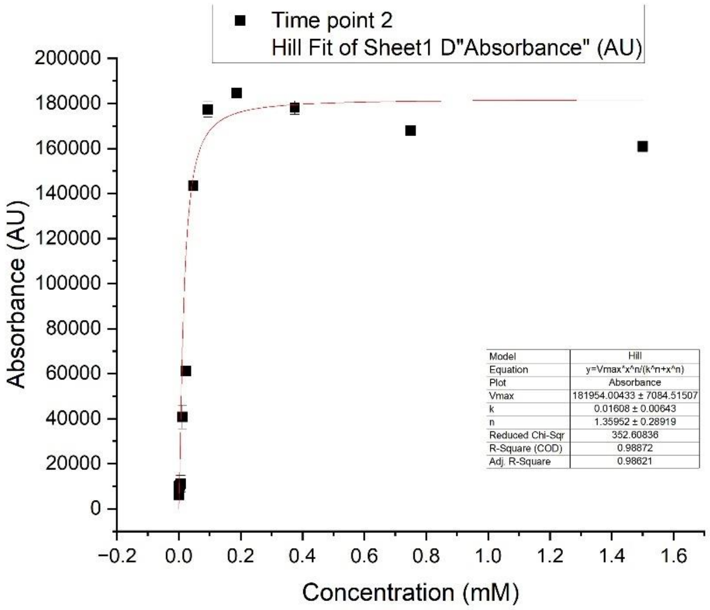

Figure S360 – Graph showing the lysis of calcein loaded PE:PG 1:1 vesicles at 5 minutes, with respect to increasing concentration of **d**. Data was then fitted to the Hill equation. Average of n= 3, error = one standard deviation of the mean.

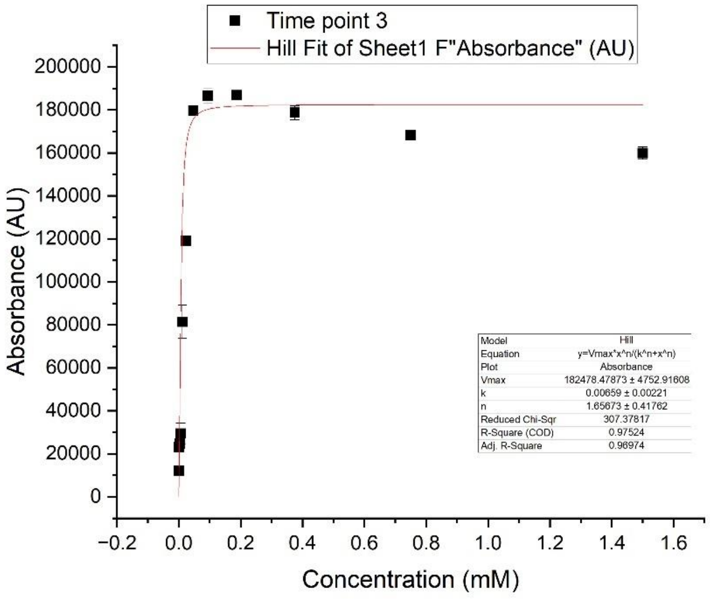

Figure S361 – Graph showing the lysis of calcein loaded PE:PG 1:1 vesicles at 10 minutes, with respect to increasing concentration of **d**. Data was then fitted to the Hill equation. Average of n= 3, error = one standard deviation of the mean.

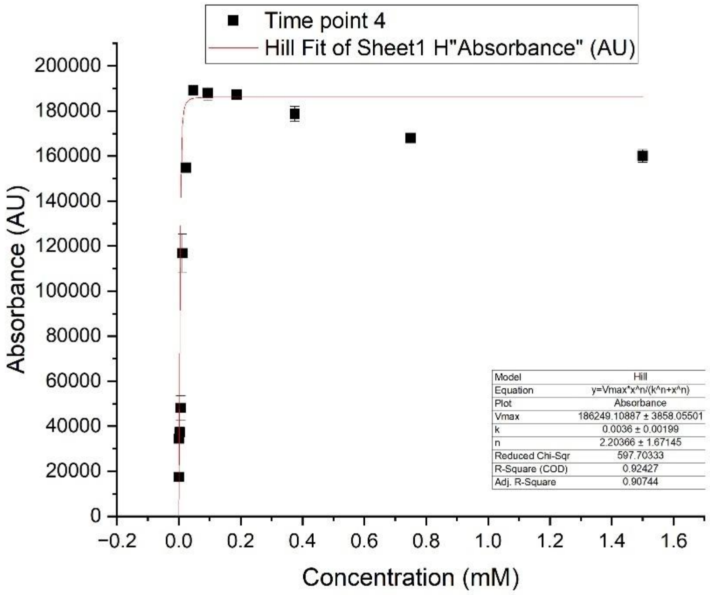

Figure S362 – Graph showing the lysis of calcein loaded PE:PG 1:1 vesicles at 15 minutes, with respect to increasing concentration of **d**. Data was then fitted to the Hill equation. Average of n= 3, error = one standard deviation of the mean.

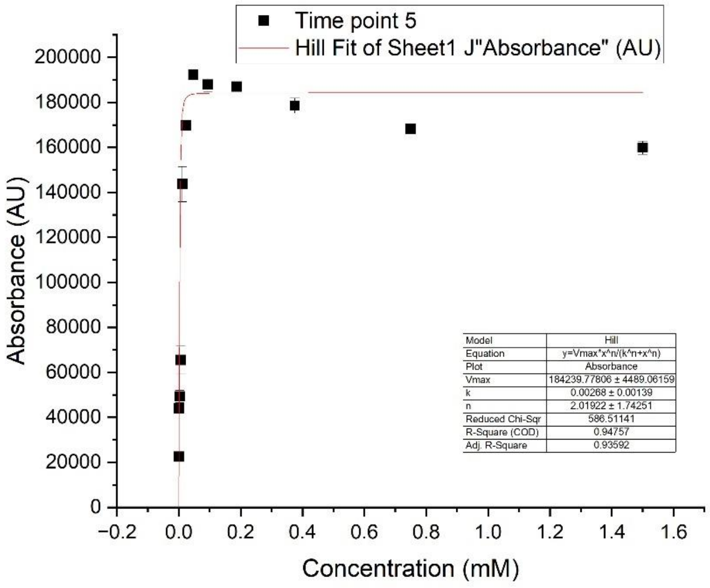

Figure S363 – Graph showing the lysis of calcein loaded PE:PG 1:1 vesicles at 20 minutes, with respect to increasing concentration of **d**. Data was then fitted to the Hill equation. Average of n= 3, error = one standard deviation of the mean.

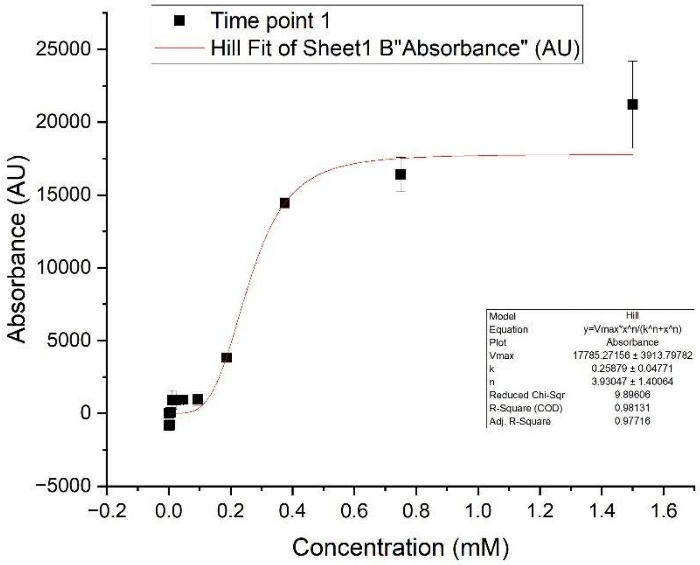

Figure S364 – Graph showing the lysis of calcein loaded PE:PG 1:1 vesicles at 1 minute, with respect to increasing concentration of **e**. Data was then fitted to the Hill equation. Average of n= 3, error = one standard deviation of the mean.

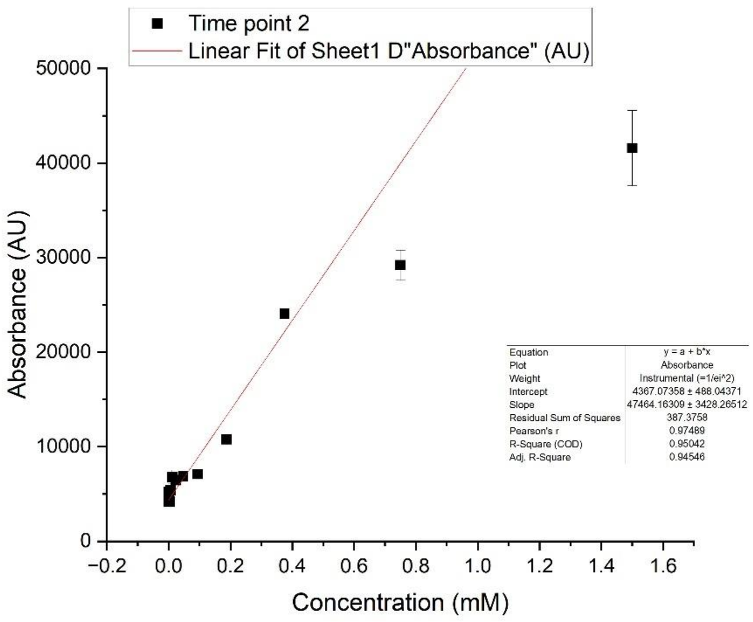

Figure S365 – Graph showing the lysis of calcein loaded PE:PG 1:1 vesicles at 5 minutes, with respect to increasing concentration of **e**. Data was then fitted to a linear line of best fit. Average of n= 3, error = one standard deviation of the mean.

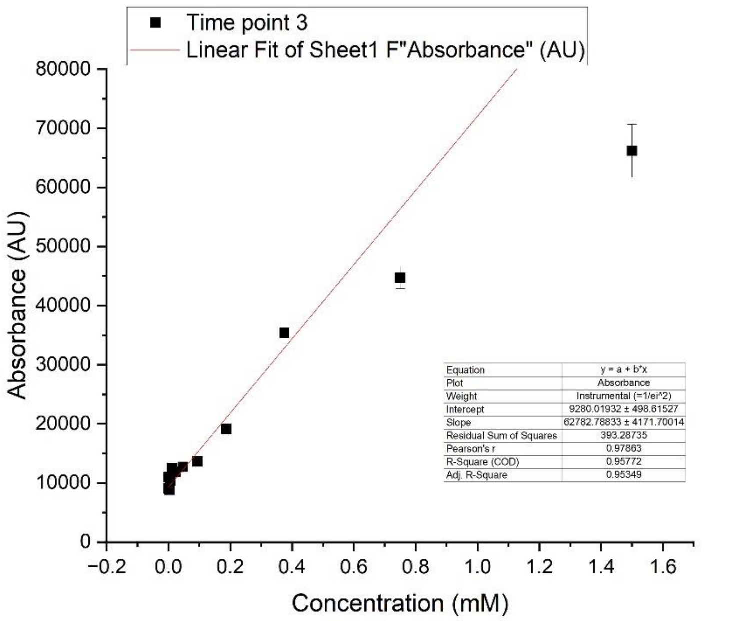

Figure S366 – Graph showing the lysis of calcein loaded PE:PG 1:1 vesicles at 10 minutes, with respect to increasing concentration of **e**. Data was then fitted to a linear line of best fit. Average of n= 3, error = one standard deviation of the mean.

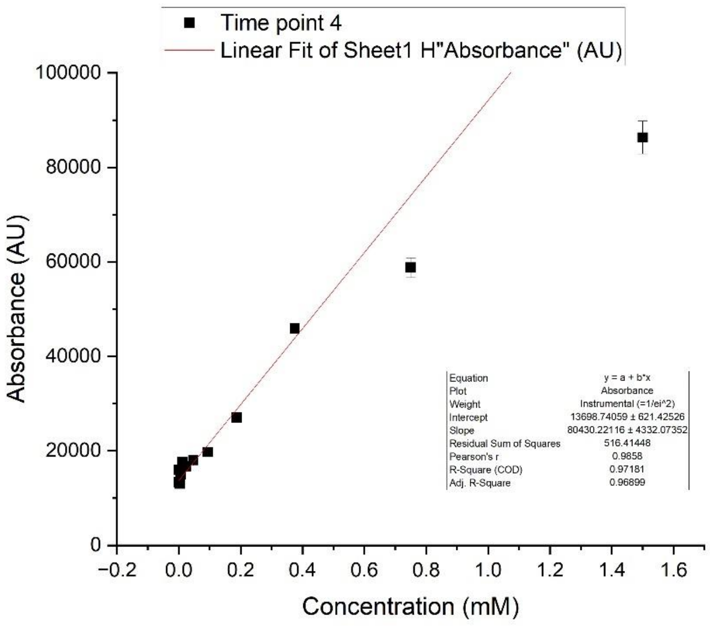

Figure S367 – Graph showing the lysis of calcein loaded PE:PG 1:1 vesicles at 15 minutes, with respect to increasing concentration of **e**. Data was then fitted to The Hill equation . Average of n= 3, error = one standard deviation of the mean.

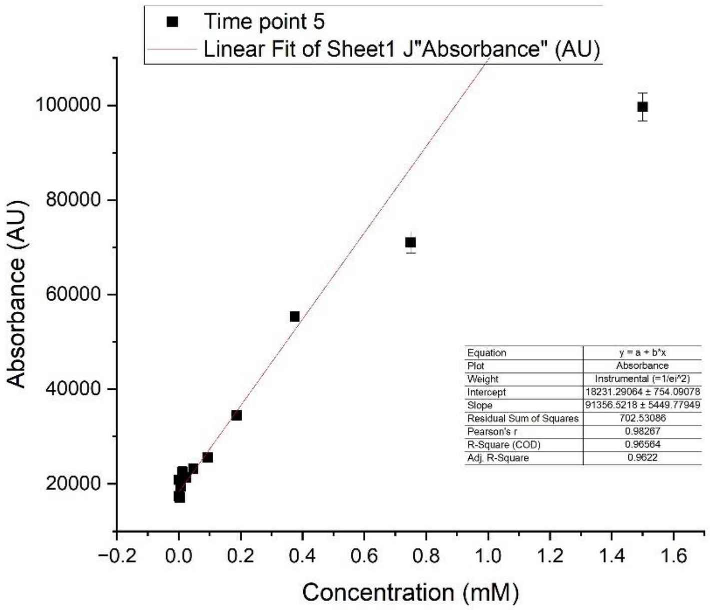

Figure S368 – Graph showing the lysis of calcein loaded PE:PG 1:1 vesicles at 20 minutes, with respect to increasing concentration of **e**. Data was then fitted to a linear line of best fit. Average of n= 3, error = one standard deviation of the mean.

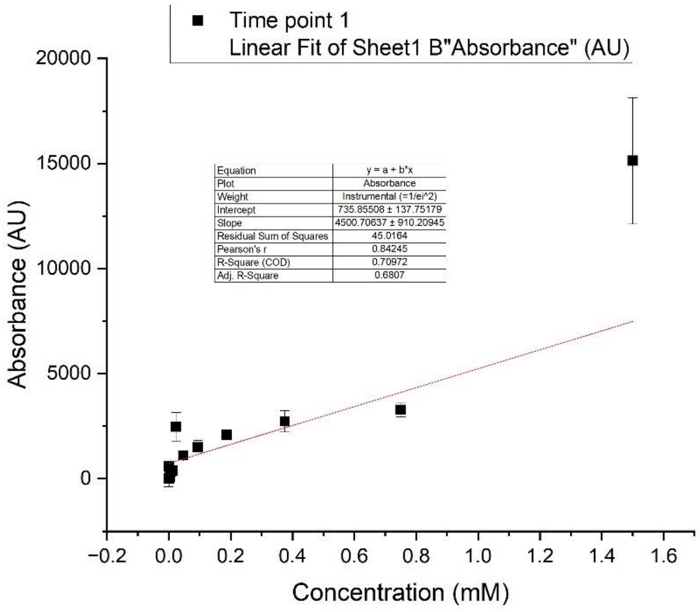

Figure S369 – Graph showing the lysis of calcein loaded PE:PG 1:1 vesicles at 1 minute, with respect to increasing concentration of **f**. Data was then fitted to a linear line of best fit. Average of n= 3, error = one standard deviation of the mean.

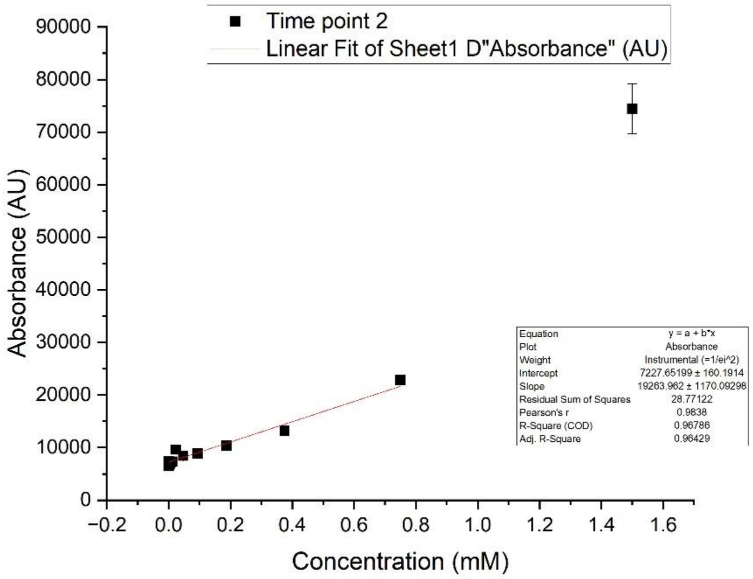

Figure S370 – Graph showing the lysis of calcein loaded PE:PG 1:1 vesicles at 5 minutes, with respect to increasing **f** concentration. Data was then fitted to a linear line of best fit. Average of n= 3, error = one standard deviation of the mean.

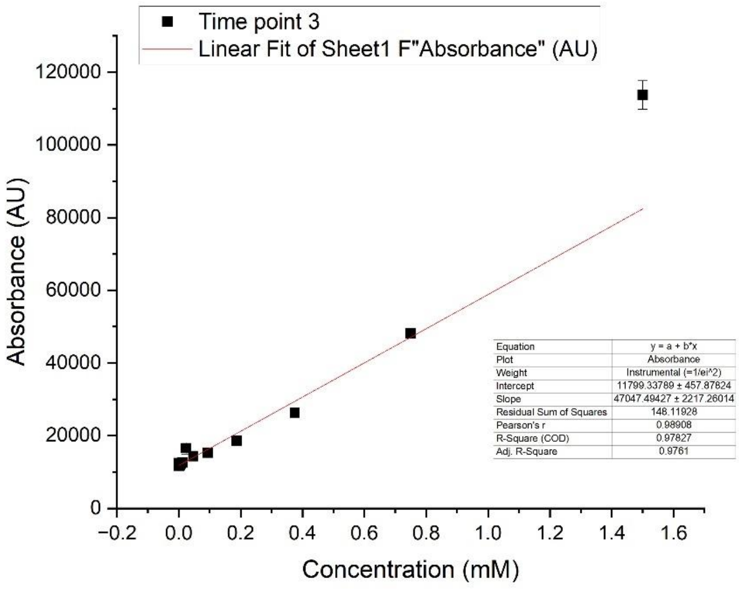

Figure S371 – Graph showing the lysis of calcein loaded PE:PG 1:1 vesicles at 10 minutes, with respect to increasing concentration of **f**. Data was then fitted to a linear line of best fit. Average of n= 3, error = one standard deviation of the mean.

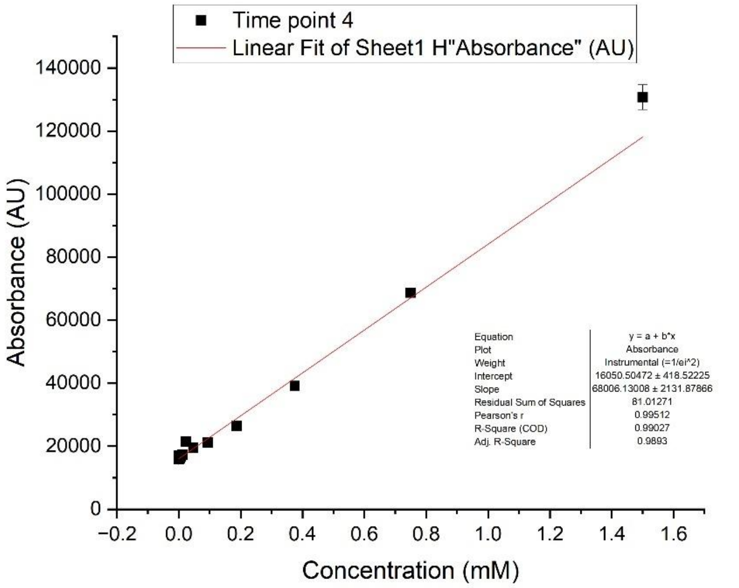

Figure S372 – Graph showing the lysis of calcein loaded PE:PG 1:1 vesicles at 15 minutes, with respect to increasing concentration of **f**. Data was then fitted to a linear line of best fit. Average of n= 3, error = one standard deviation of the mean.

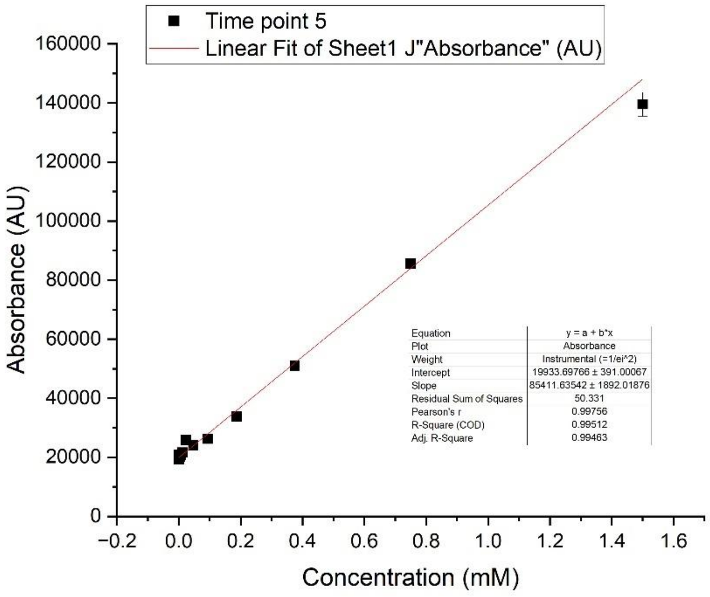

Figure S373 – Graph showing the lysis of calcein loaded PE:PG 1:1 vesicles at 20 minutes, with respect to increasing concentration of **f**. Data was then fitted to a linear line of best fit. Average of n= 3, error = one standard deviation of the mean.

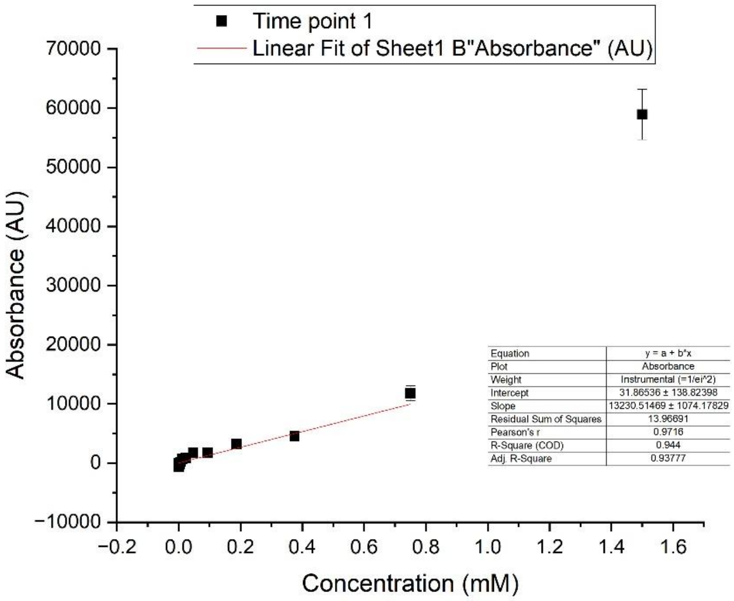

Figure S374 – Graph showing the lysis of calcein loaded PE:PG 1:1 vesicles at 1 minute, with respect to increasing concentration of **g**. Data was then fitted to a linear line of best fit. Average of n= 3, error = one standard deviation of the mean.

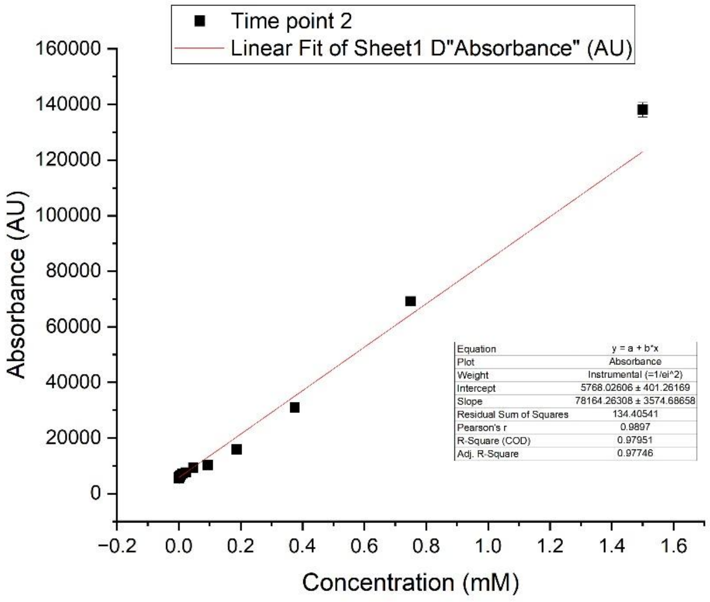

Figure S375 – Graph showing the lysis of calcein loaded PE:PG 1:1 vesicles at 5 minutes, with respect to increasing concentration of **g**. Data was then fitted to a linear line of best fit. Average of n= 3, error = one standard deviation of the mean.

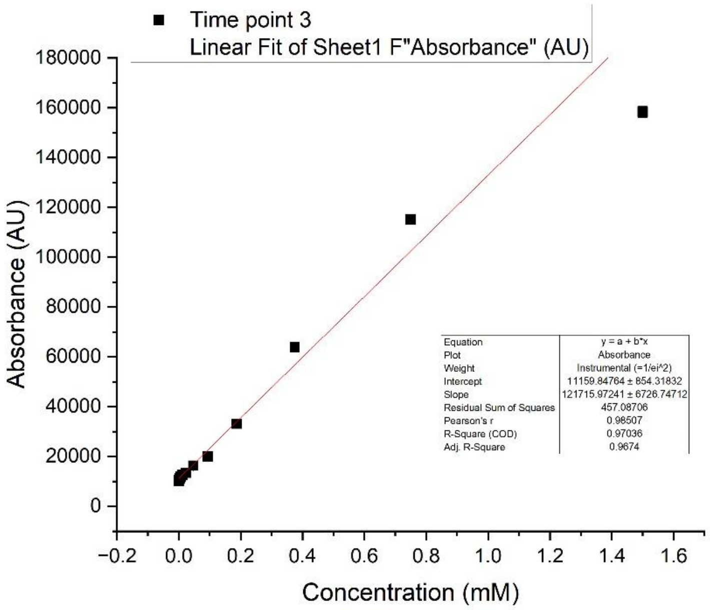

Figure S376 – Graph showing the lysis of calcein loaded PE:PG 1:1 vesicles at 10 minutes, with respect to increasing concentration of **g**. Data was then fitted to a linear line of best fit. Average of n= 3, error = one standard deviation of the mean.

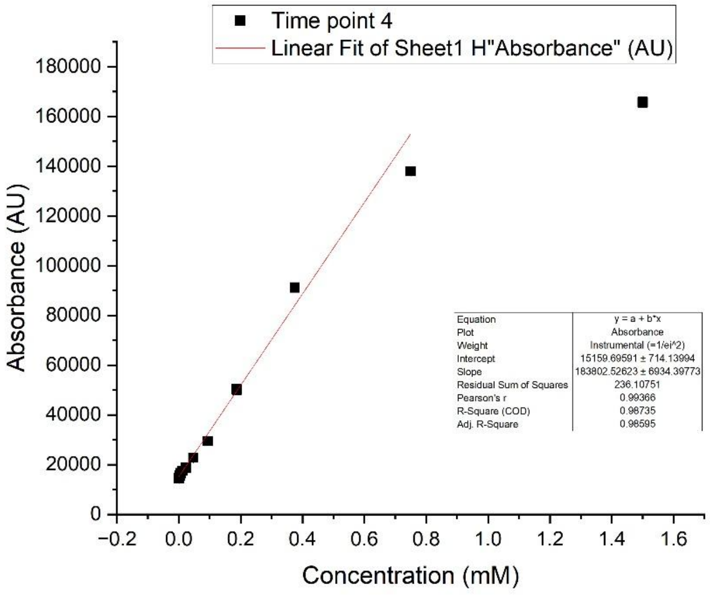

Figure S377 – Graph showing the lysis of calcein loaded PE:PG 1:1 vesicles at 15 minutes, with respect to increasing concentration of **g**. Data was then fitted to a linear line of best fit. Average of n= 3, error = one standard deviation of the mean.

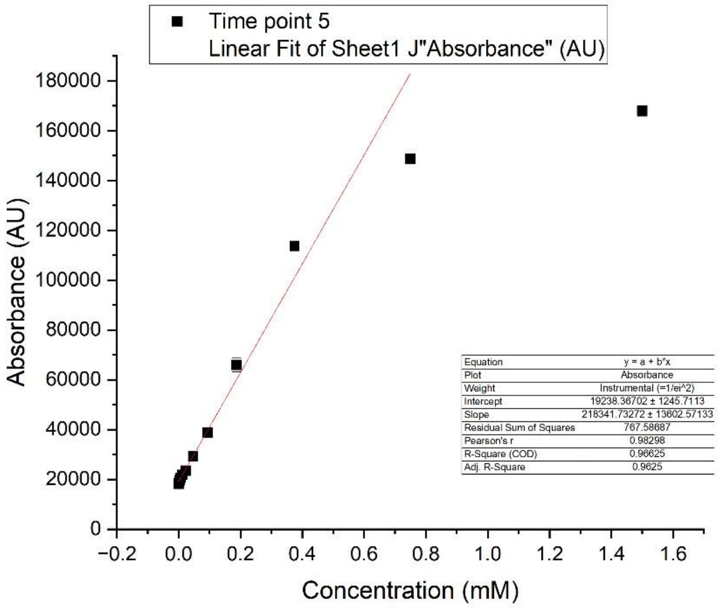

Figure S378 – Graph showing the lysis of calcein loaded PE:PG 1:1 vesicles at 20 minutes, with respect to increasing concentration of **g**. Data was then fitted to a linear line of best fit. Average of n= 3, error = one standard deviation of the mean.

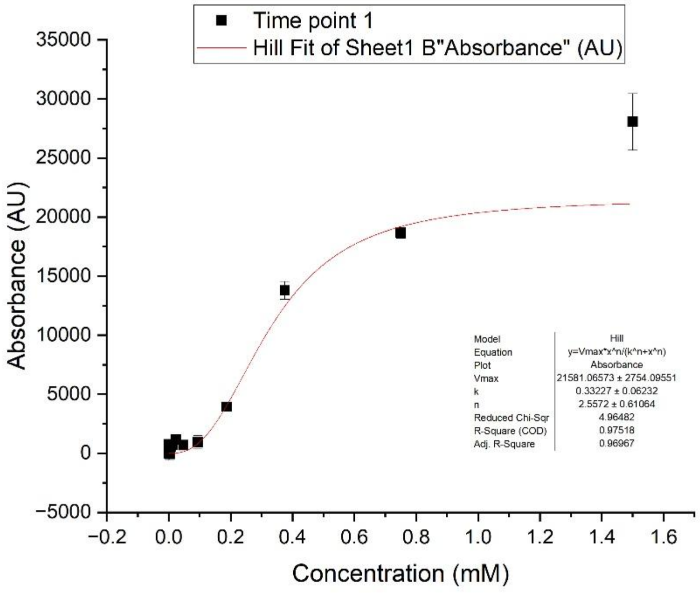

Figure S379 – Graph showing the lysis of calcein loaded PE:PG 1:1 vesicles at 1 minute, with respect to increasing concentration of **h**. Data was then fitted to the Hill equation. Average of n= 3, error = one standard deviation of the mean.

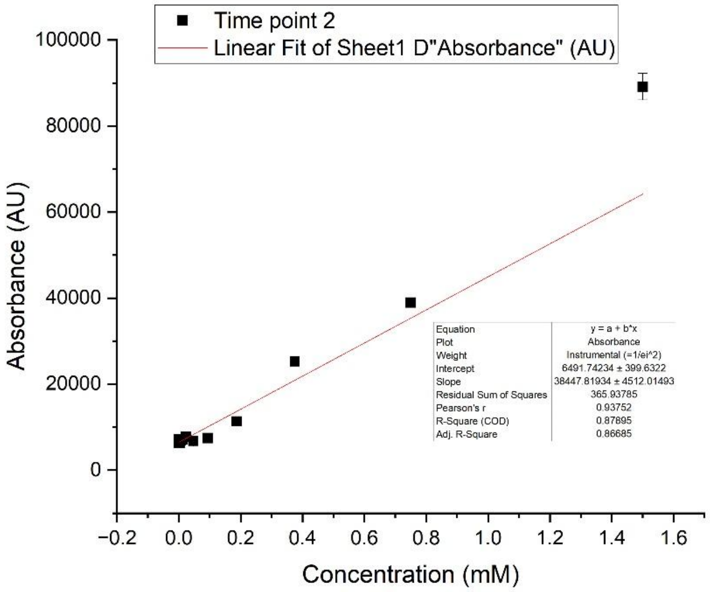

Figure S380 – Graph showing the lysis of calcein loaded PE:PG 1:1 vesicles at 5 minutes, with respect to increasing concentration of **h**. Data was then fitted to the linear line of best fit. Average of n= 3, error = one standard deviation of the mean.

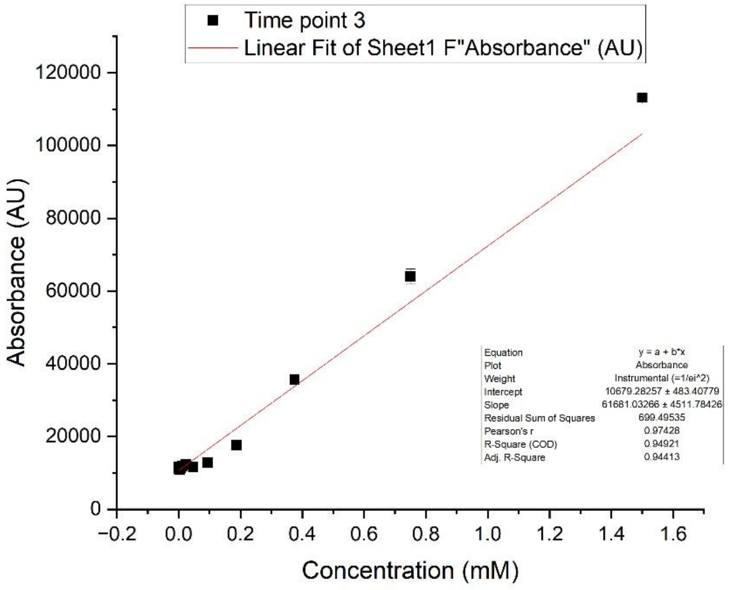

Figure S381 – Graph showing the lysis of calcein loaded PE:PG 1:1 vesicles at 10 minutes, with respect to increasing concentration of **h**. Data was then fitted to the linear line of best fit. Average of n= 3, error = one standard deviation of the mean.

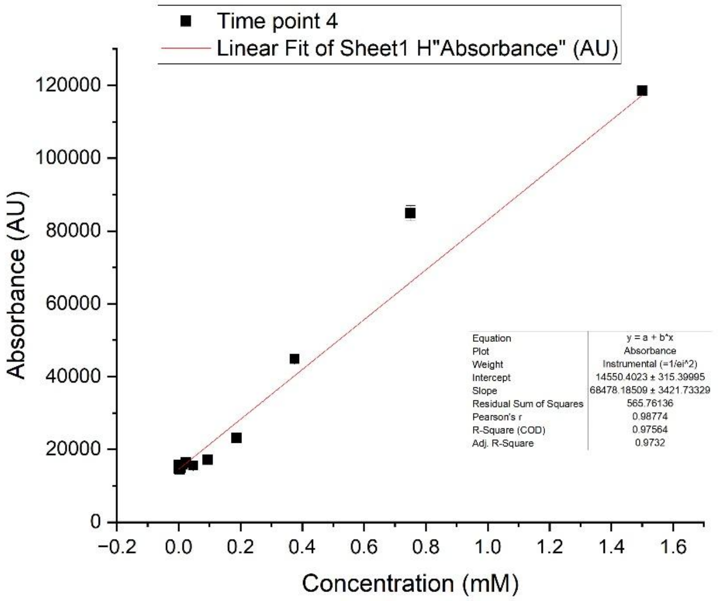

Figure S382 – Graph showing the lysis of calcein loaded PE:PG 1:1 vesicles at 15 minutes, with respect to increasing concentration of **h**. Data was then fitted to the linear line of best fit. Average of n= 3, error = one standard deviation of the mean.

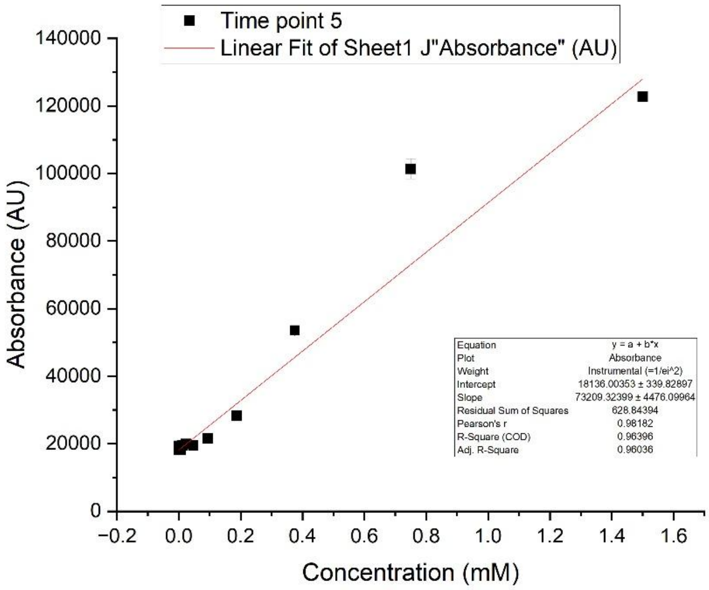

Figure S383 – Graph showing the lysis of calcein loaded PE:PG 1:1 vesicles at 20 minutes, with respect to increasing concentration of **h**. Data was then fitted to the linear line of best fit. Average of n= 3, error = one standard deviation of the mean.

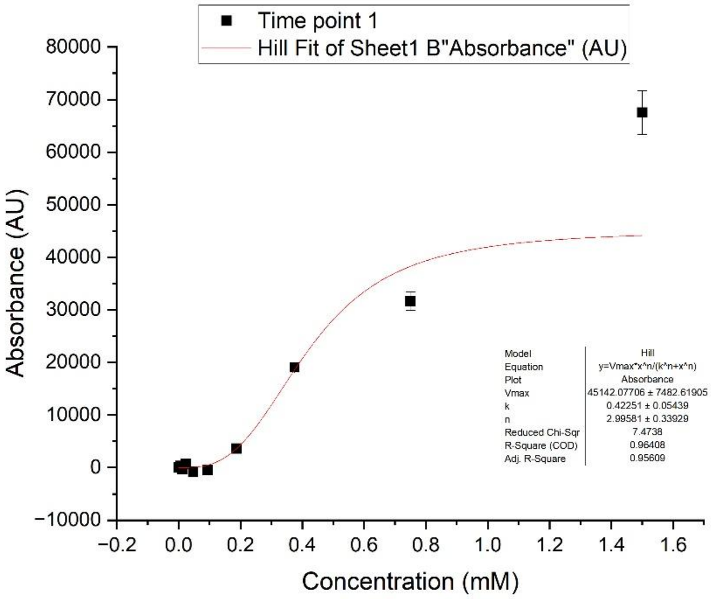

Figure S384 – Graph showing the lysis of calcein loaded PE:PG 1:1 vesicles at 1 minute, with respect to increasing concentration of **i**. Data was then fitted to the Hill equation. Average of n= 3, error = one standard deviation of the mean.

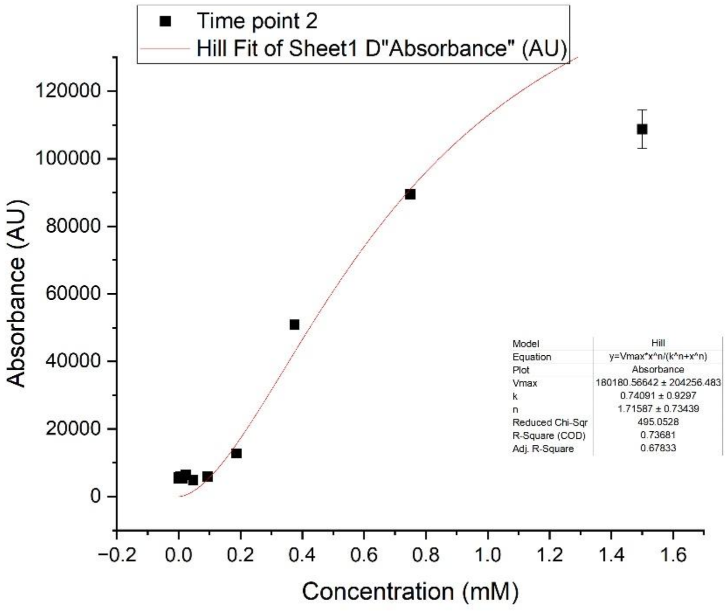

Figure S385 – Graph showing the lysis of calcein loaded PE:PG 1:1 vesicles at 5 minutes, with respect to increasing concentration of **i**. Data was then fitted to the Hill equation. Average of n= 3, error = one standard deviation of the mean.

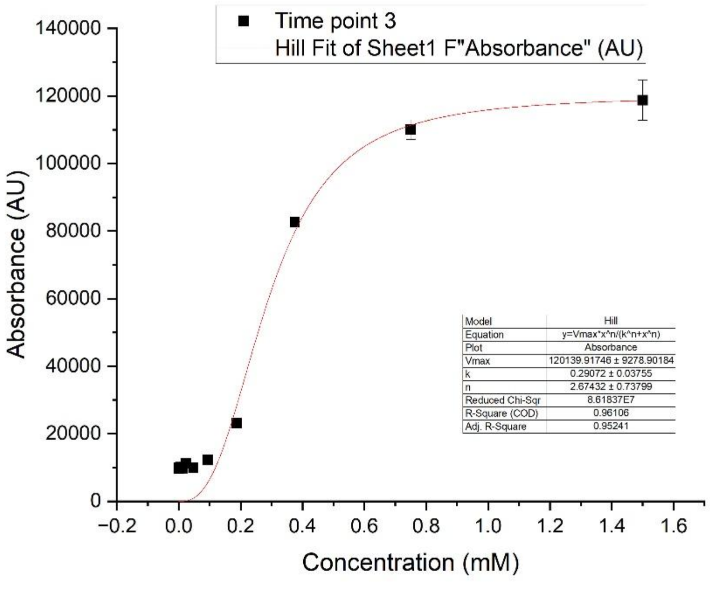

Figure S386 – Graph showing the lysis of calcein loaded PE:PG 1:1 vesicles at 10 minutes, with respect to increasing concentration of **i**. Data was then fitted to the Hill equation. Average of n= 3, error = one standard deviation of the mean.

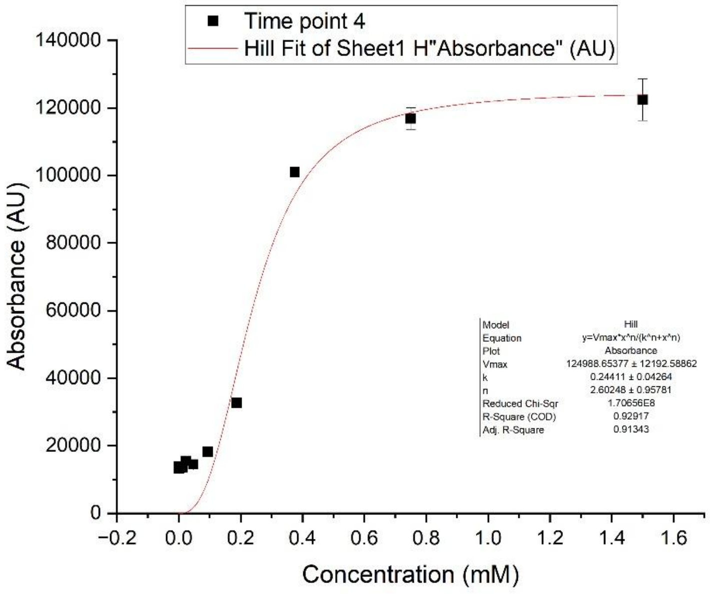

Figure S387 – Graph showing the lysis of calcein loaded PE:PG 1:1 vesicles at 15 minutes, with respect to increasing concentration of **i**. Data was then fitted to the Hill equation. Average of n= 3, error = one standard deviation of the mean.

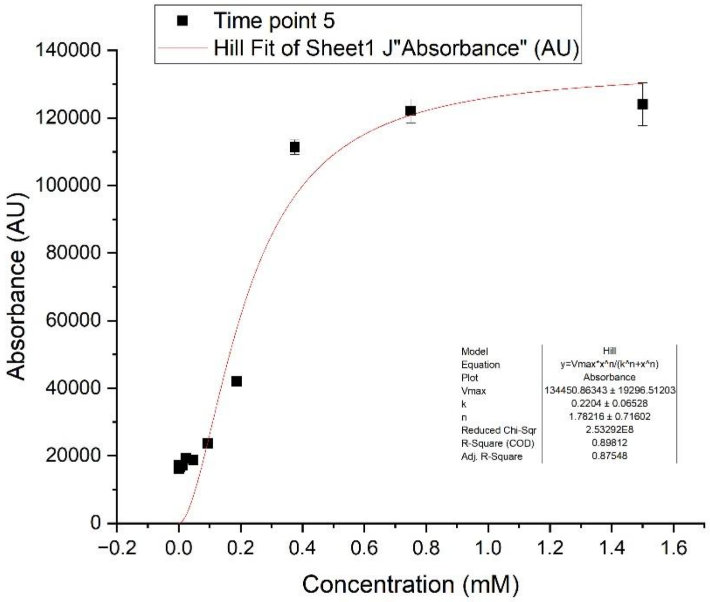

Figure S388 – Graph showing the lysis of calcein loaded PE:PG 1:1 vesicles at 20 minutes, with respect to increasing concentration of **i**. Data was then fitted to the Hill equation. Average of n= 3, error = one standard deviation of the mean.

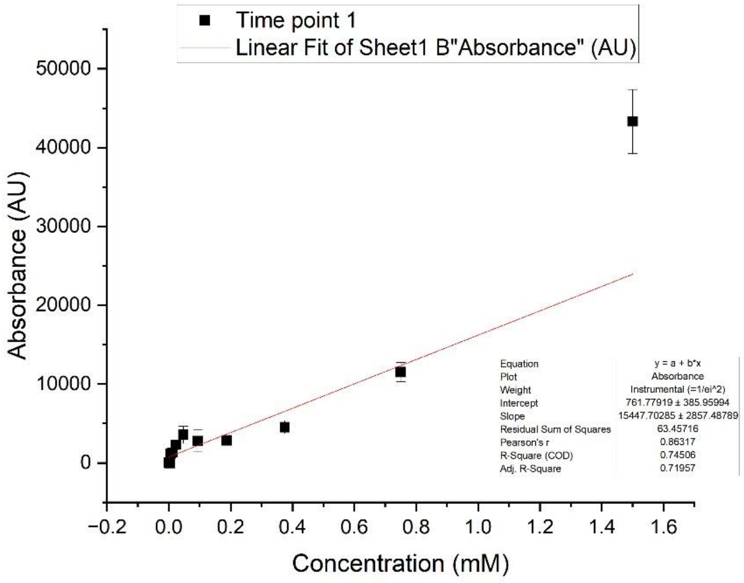

Figure S389 – Graph showing the lysis of calcein loaded PE:PG 1:1 vesicles at 1 minute, with respect to increasing concentration of **j**. Data was then fitted to a linear line of best fit. Average of n= 3, error = one standard deviation of the mean.

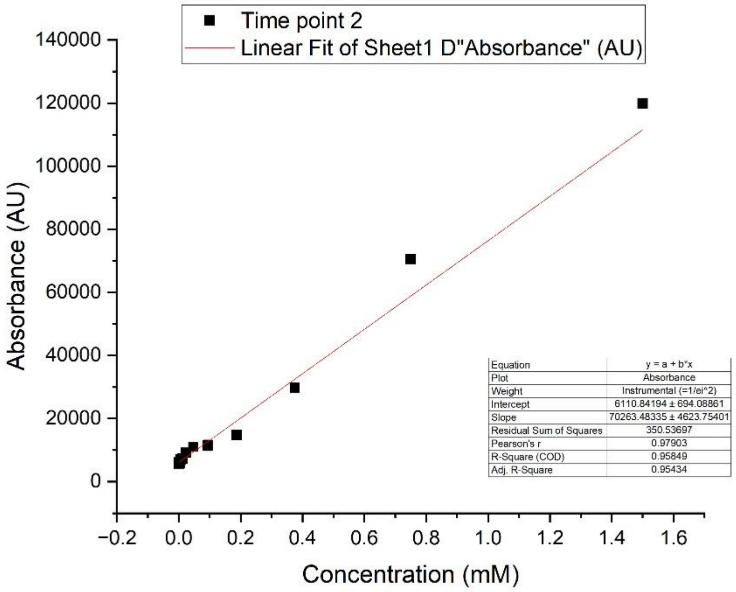

Figure S390 – Graph showing the lysis of calcein loaded PE:PG 1:1 vesicles at 5 minutes, with respect to increasing concentration of **j**. Data was then fitted to a linear line of best fit. Average of n= 3, error = one standard deviation of the mean.

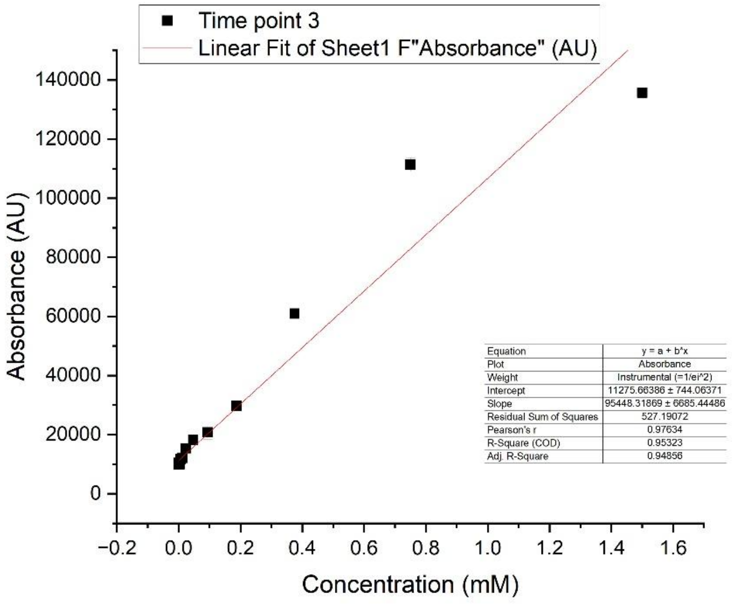

Figure S391 – Graph showing the lysis of calcein loaded PE:PG 1:1 vesicles at 10 minutes, with respect to increasing concentration of **j**. Data was then fitted to a linear line of best fit. Average of n= 3, error = one standard deviation of the mean.

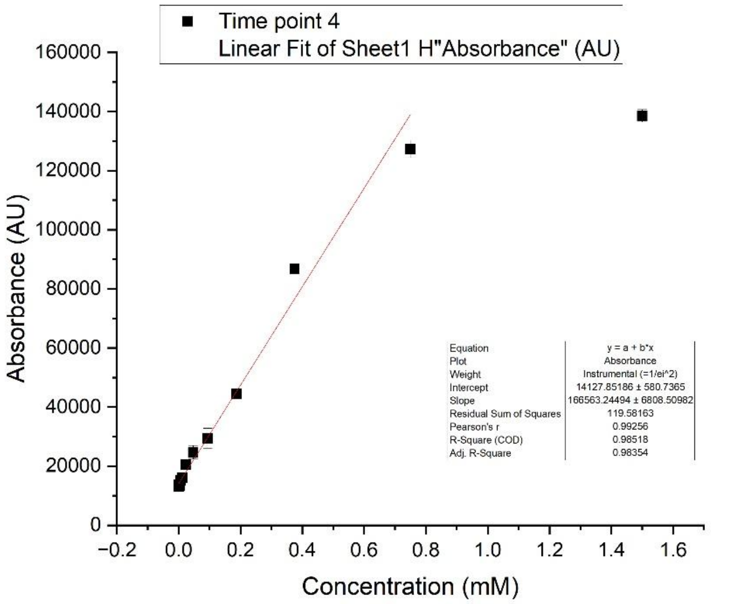

Figure S392 – Graph showing the lysis of calcein loaded PE:PG 1:1 vesicles at 15 minutes, with respect to increasing concentration of **j**. Data was then fitted to a linear line of best fit. Average of n= 3, error = one standard deviation of the mean.

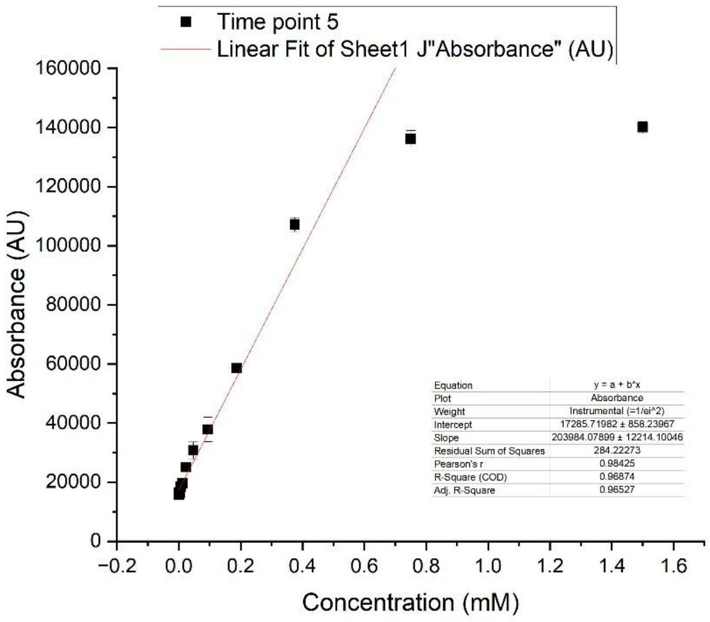

Figure S393 – Graph showing the lysis of calcein loaded PE:PG 1:1 vesicles at 20 minutes, with respect to increasing concentration of **j**. Data was then fitted to a linear line of best fit. Average of n= 3, error = one standard deviation of the mean.

PE:PG 1:1 vesicle titration summary

Table S14 – Summary of EC_{50}, R^2 and *n* values determined through fitting calcein filled PE:PG 1:1 vesicles to the Hill equation. If a fit to the Hill equation could not be obtained these data were fitted to a linear line of best fit to estimate a EC_{50} value. Where '-' is shown, the data was fitted to a linear line of best fit.

**Compound**	**Timepoint**	**EC_{50} (mM)**	**R^2**	***n***	**Compound**	**Timepoint**	**EC_{50} (mM)**	**R^2**	***n***
	1	0.055	0.995	1.181		1	0.097	0.989	2.527
	2	0.010	0.985	1.316		2	0.016	0.989	1.36
**1**	3	0.004	0.95	1.311	**d**	3	0.007	0.975	1.657
	4	0.002	0.969	1.183		4	0.004	0.924	2.204
	5	0.002	0.945	1.235		5	0.003	0.948	2.019
	1	> 1.5	0.937	-		1	> 1.5	0.981	-
	2	1.430	0.979	-		2	> 1.5	0.95	-
**2**	3	0.830	0.993	-	**e**	3	1.090	0.958	-
	4	0.661	0.884	-		4	0.789	0.972	-
	5	0.585	0.869	-		5	0.648	0.966	-
	1	> 1.5	0.873	-		1	> 1.5	0.71	-
	2	> 1.5	0.847	-		2	> 1.5	0.968	-
**3**	3	1.329	0.955	-	**f**	3	1.398	0.978	-
	4	0.953	0.918	-		4	0.898	0.99	-
	5	0.829	0.926	-		5	0.673	0.995	-
	1	> 1.5	0.914	-		1	> 1.5	0.944	-
	2	> 1.5	0.872	-		2	0.920	0.98	-
**4**	3	0.948	0.983	-	**g**	3	0.546	0.97	-
	4	0.675	0.983	-		4	0.337	0.987	-
	5	0.574	0.964	-		5	0.266	0.966	-
	1	> 1.5	0.884	-		1	> 1.5	0.975	-
	2	0.302	0.918	-		2	> 1.5	0.879	-
**5**	3	0.129	0.886	-	**h**	3	1.085	0.949	-
	4	0.100	0.915	-		4	0.914	0.976	-
	5	0.234	0.912	0.407		5	0.810	0.964	-
	1	0.902	0.999	2.081		1	0.423	0.964	2.996
	2	0.030	0.771	0.817		2	0.741	0.737	1.716
**a**	3	0.012	0.599	0.609	**i**	3	0.291	0.961	2.602
	4	0.004	0.715	0.698		4	0.244	0.929	1.782
	5	0.002	0.67	0.663		5	0.220	0.898	
	1	0.084	0.979	2.491		1	> 1.5	0.745	-
	2	0.028	0.974	2.144		2	1.019	0.958	-
**b**	3	0.022	0.915	1.063	**j**	3	0.695	0.953	-
	4	0.013	0.934	1.162		4	0.378	0.958	-
	5	0.014	0.929	0.691		5	0.295	0.969	-
	1	0.063	0.985	3.164					
	2	0.025	0.904	1.809					
**c**	3	0.011	0.935	1.449					
	4	0.007	0.925	1.345					
	5	0.005	0.926	1.269					

Summary

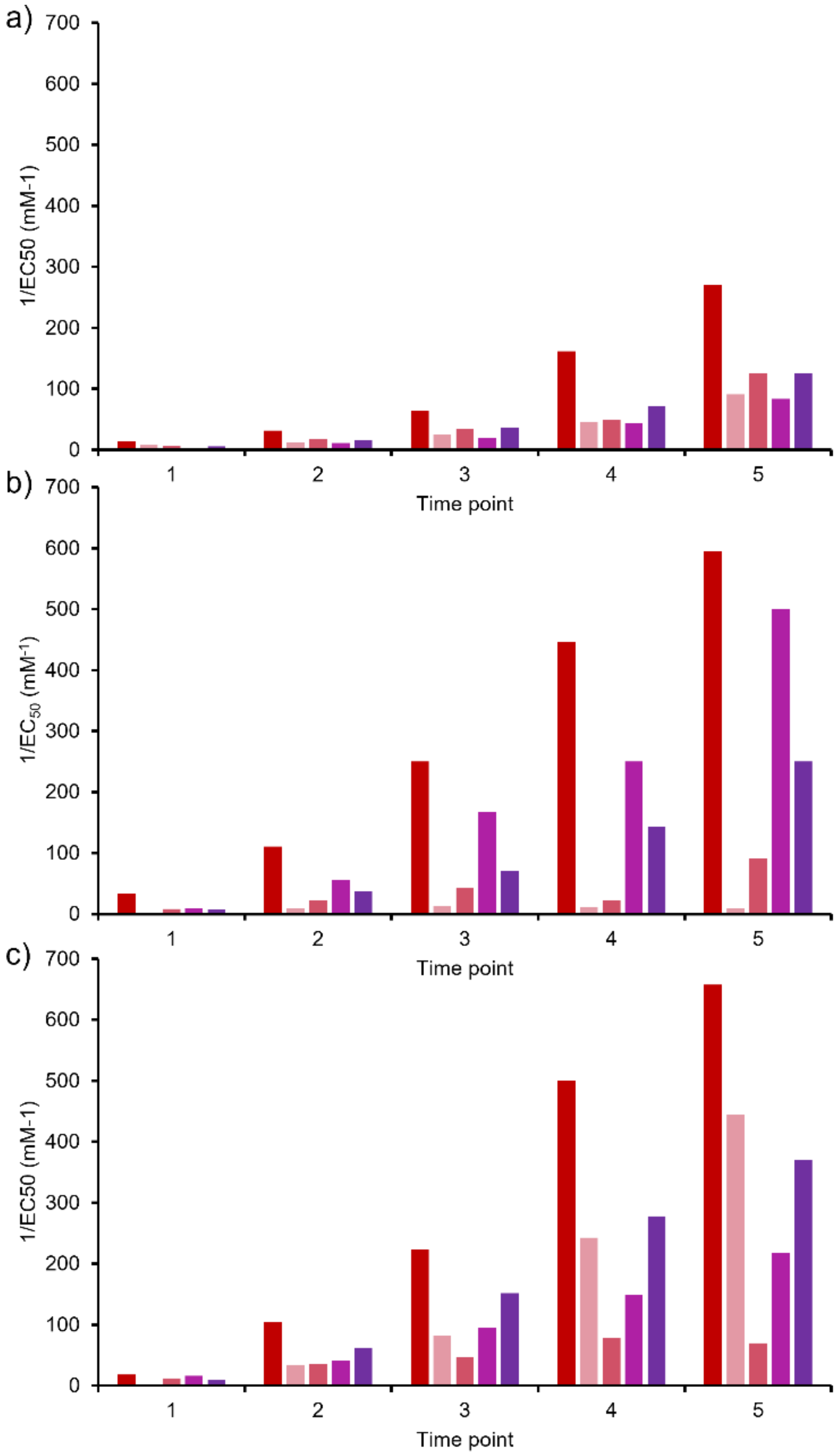

Figure S394 – $1/EC_{50}$ (mM^{-1}) values obtained from the fitting of **1** and **a** – **d** to calcein loaded a) PG vesicles, b) PE:PG 3:1 vesicles and c) PE:PG 1:1 vesicles. Dark red = **1**, light pink = **a**, dark pink = **b**, light purple = **c**, dark purple **d**.

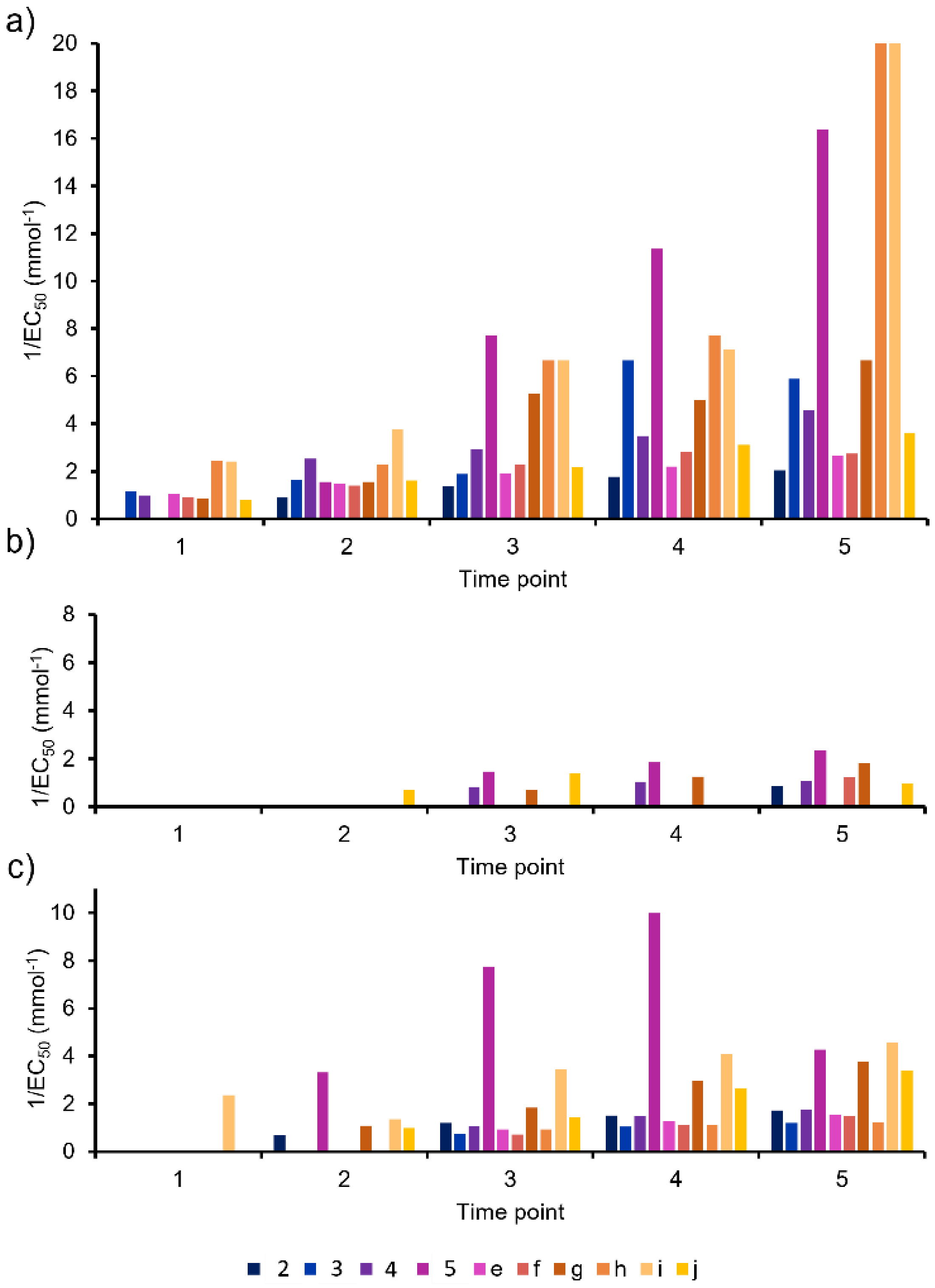

Figure S395 – 1/EC$_{50}$ (mM^{-1}) values obtained from the fitting of **2** – **5** and **j** – to calcein loaded a) PG vesicles, b) PE:PG 3:1 vesicles and c) PE:PG 1:1 vesicles. Red = **2**, light red = **3**, orange = **4**, yellow = **5**, dark blue = **e**, blue = **f**, light blue = **g**, turquoise = **h**, light green = **i**, dark green = **j**.

Section S15: FP membrane fluidity data

PG FP titrations

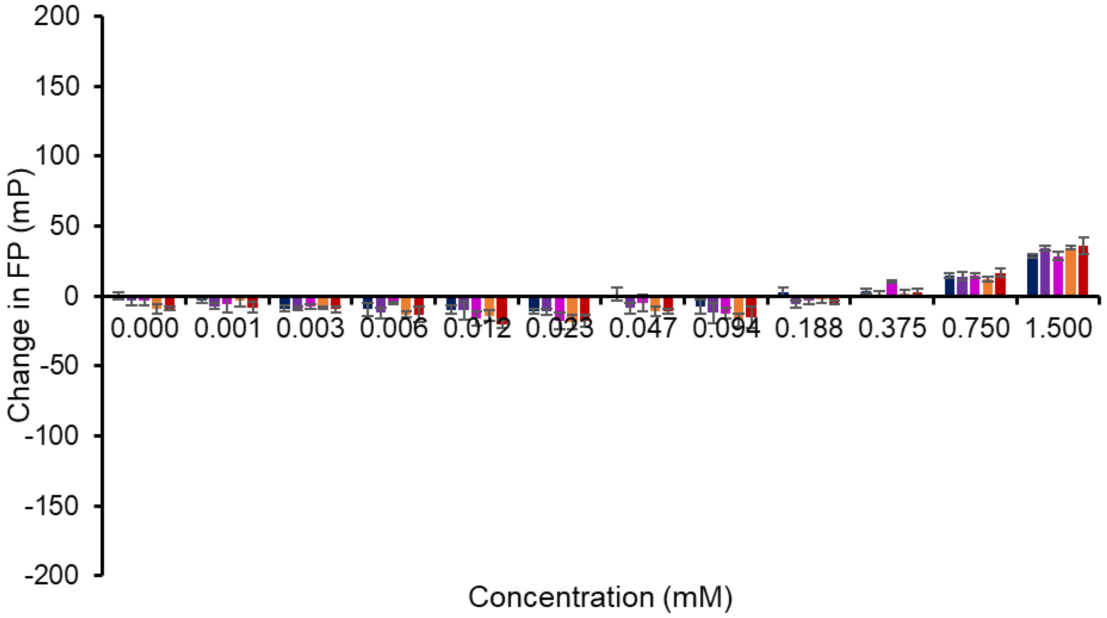

Figure S396 – Change in FP of PG vesicles incubated with DPH (30 μM) following addition of **1**. Blue = 1 minute, purple = 5 minutes, pink = 10 minutes, orange = 15 minutes, red = 20 minutes. Error = standard deviation of the mean. Average of n = 3 repeats, error = one standard deviation of the mean.

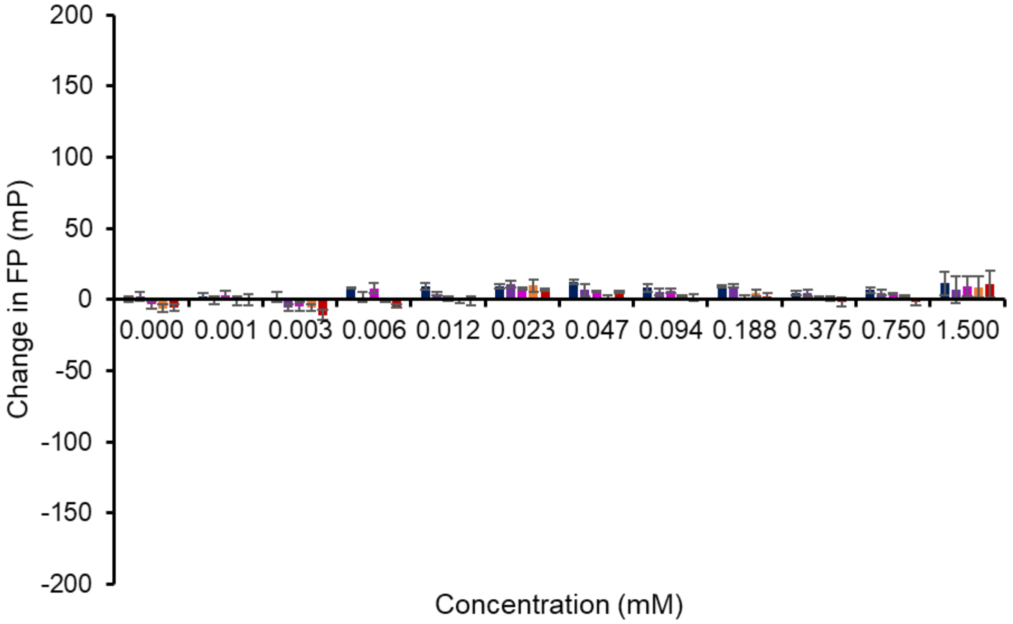

Figure S397 – Change in FP of PG vesicles incubated with DPH (30 μM)following addition of **2**. Blue = 1 minute, purple = 5 minutes, pink = 10 minutes, orange = 15 minutes, red = 20 minutes. Error = standard deviation of the mean. Average of n = 3 repeats, error = one standard deviation of the mean.

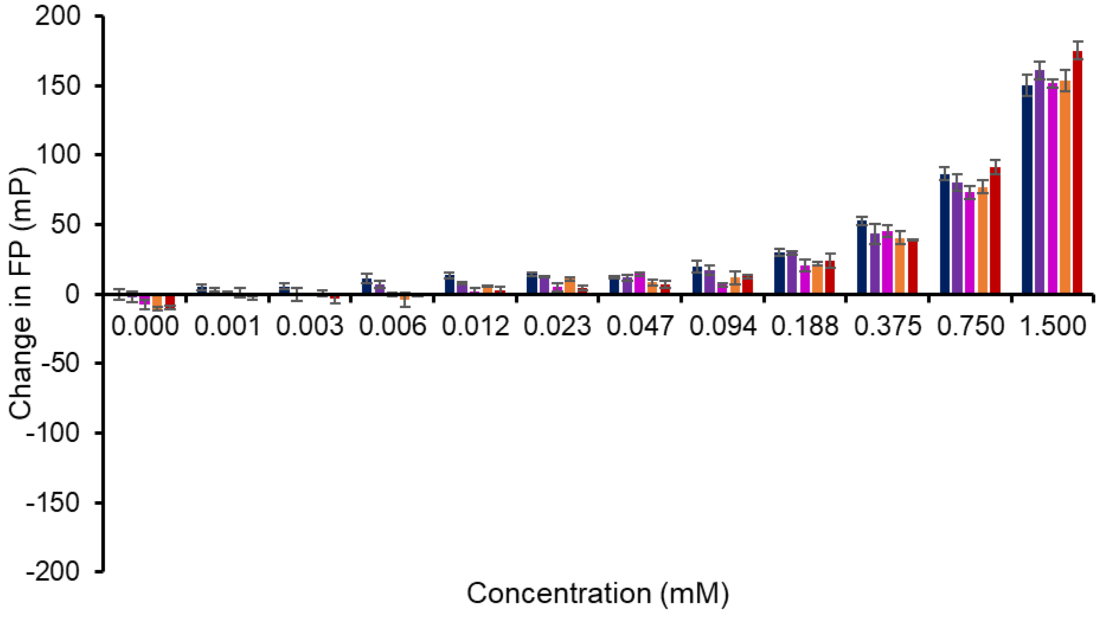

Figure S398 – Change in FP of PG vesicles incubated with DPH (30 µM) following addition of **4**. Blue = 1 minute, purple = 5 minutes, pink = 10 minutes, orange = 15 minutes, red = 20 minutes. Error = standard deviation of the mean. Average of n = 3 repeats, error = one standard deviation of the mean.

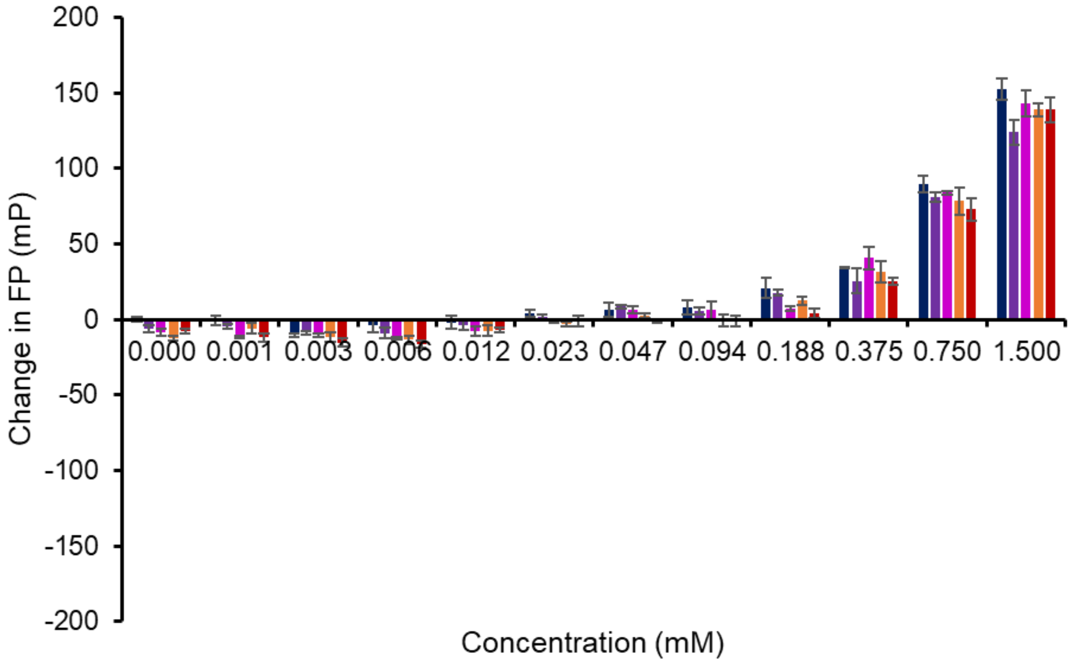

Figure S399 – Change in FP of PG vesicles incubated with DPH (30 µM) following addition of **5**. Blue = 1 minute, purple = 5 minutes, pink = 10 minutes, orange = 15 minutes, red = 20 minutes. Error = standard deviation of the mean. Average of n = 3 repeats, error = one standard deviation of the mean.

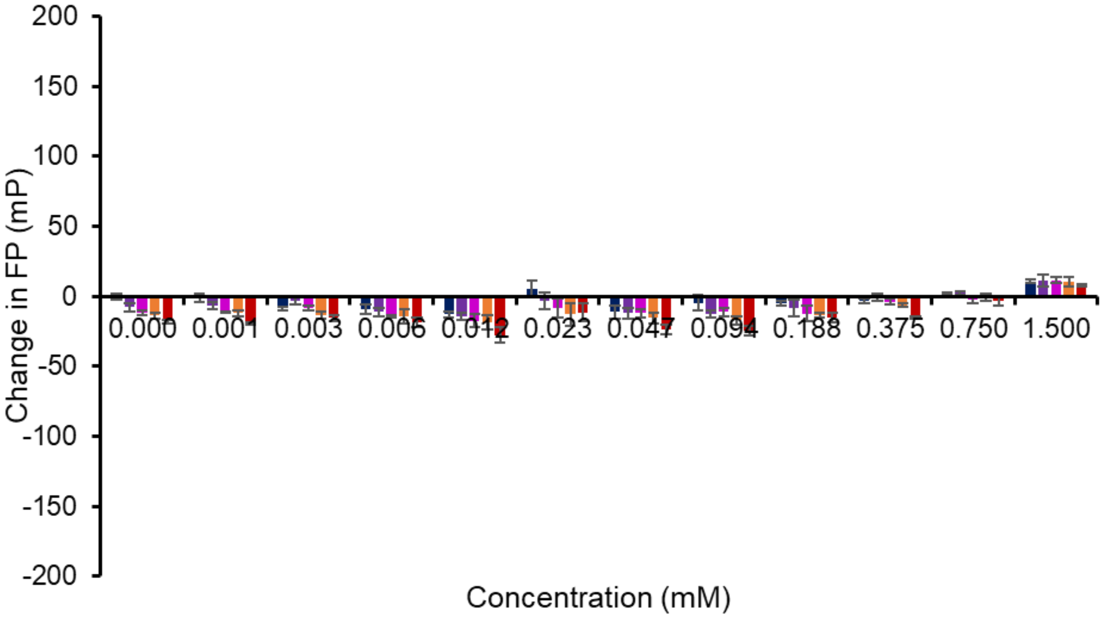

Figure S400 – Change in FP of PG vesicles incubated with DPH (30 µM) following addition of **a**. Blue = 1 minute, purple = 5 minutes, pink = 10 minutes, orange = 15 minutes, red = 20 minutes. Error = standard deviation of the mean. Average of n = 3 repeats, error = one standard deviation of the mean.

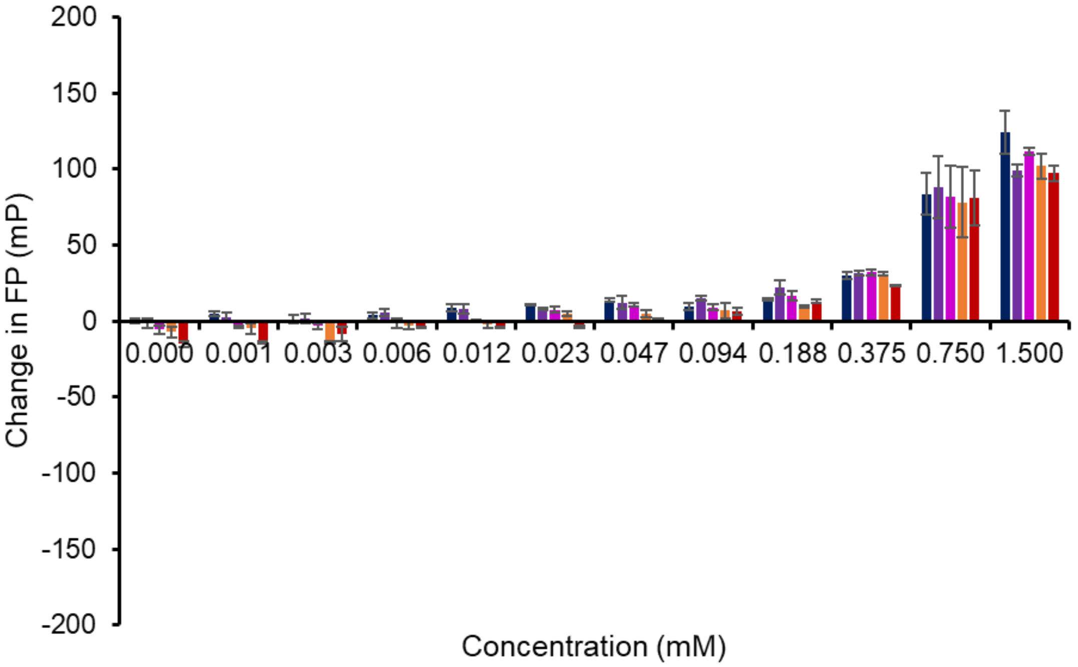

Figure S401 – Change in FP of PG vesicles incubated with DPH (30 µM) following addition of **c**. Blue = 1 minute, purple = 5 minutes, pink = 10 minutes, orange = 15 minutes, red = 20 minutes. Error = standard deviation of the mean. Average of n = 3 repeats, error = one standard deviation of the mean.

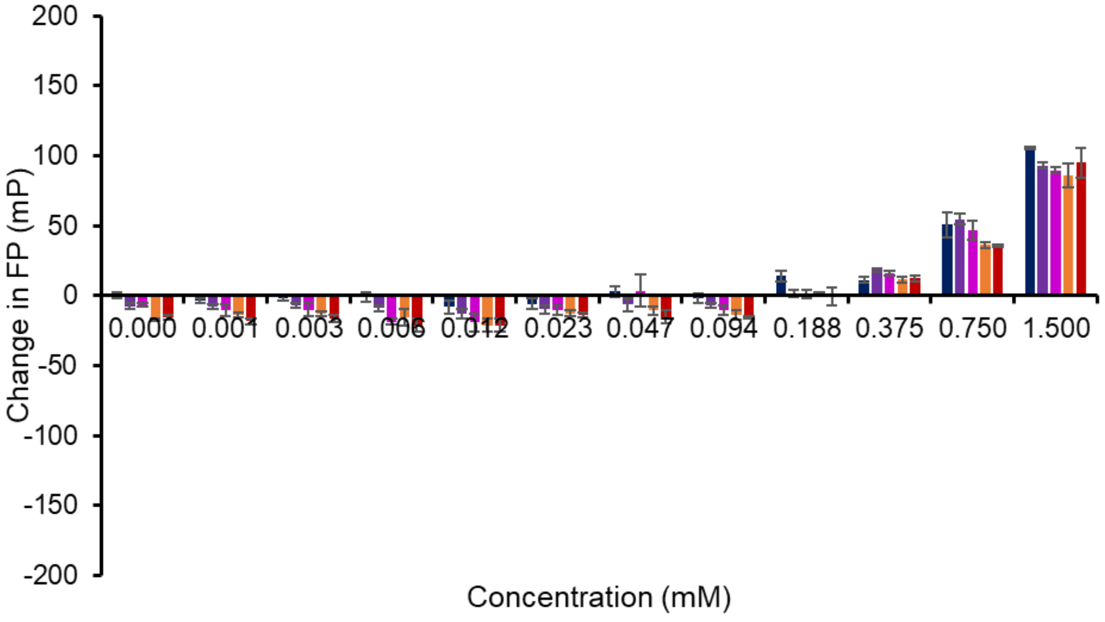

Figure S402 – Change in FP of PG vesicles incubated with DPH (30 µM) following addition of **d**. Blue = 1 minute, purple = 5 minutes, pink = 10 minutes, orange = 15 minutes, red = 20 minutes. Error = standard deviation of the mean. Average of n = 3 repeats, error = one standard deviation of the mean.

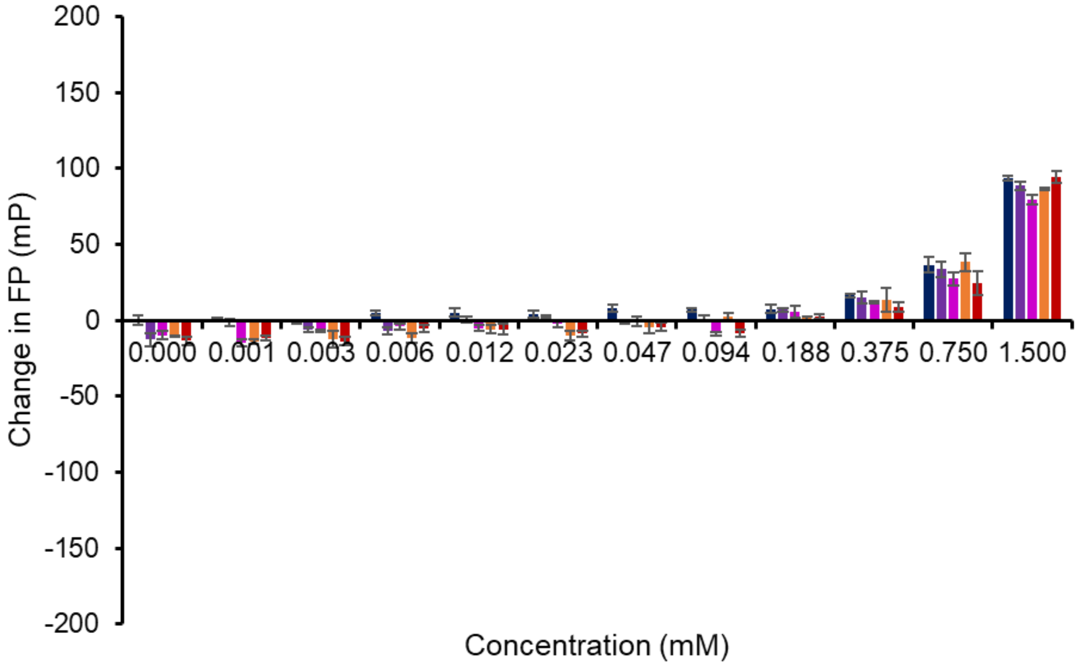

Figure S403 – Change in FP of PG vesicles incubated with DPH (30 µM) following addition of **f**. Blue = 1 minute, purple = 5 minutes, pink = 10 minutes, orange = 15 minutes, red = 20 minutes. Error = standard deviation of the mean. Average of n = 3 repeats, error = one standard deviation of the mean.

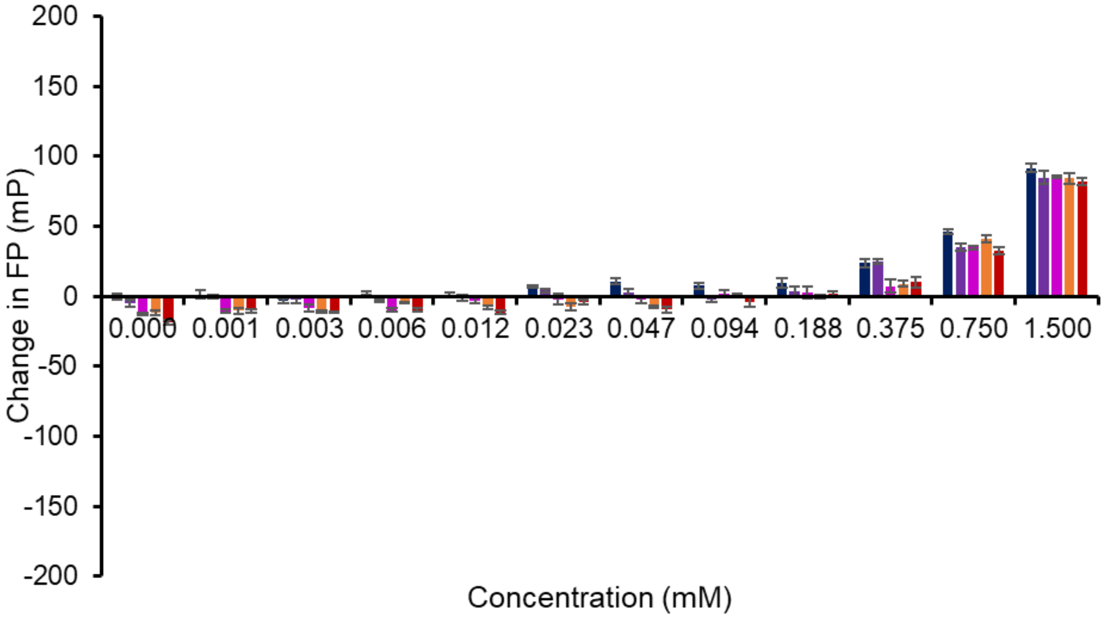

Figure S404 – Change in FP of PG vesicles incubated with DPH (30 µM) following addition of **g**. Blue = 1 minute, purple = 5 minutes, pink = 10 minutes, orange = 15 minutes, red = 20 minutes. Error = standard deviation of the mean. Average of n = 3 repeats, error = one standard deviation of the mean.

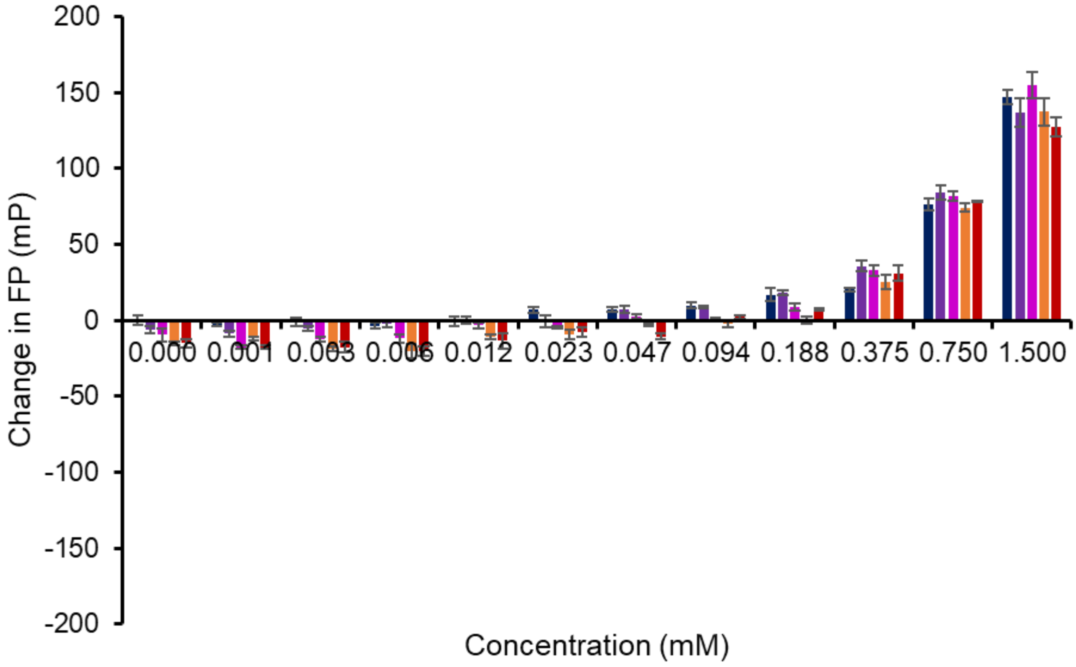

Figure S405 – Change in FP of PG vesicles incubated with DPH (30 µM) following addition of **j**. Blue = 1 minute, purple = 5 minutes, pink = 10 minutes, orange = 15 minutes, red = 20 minutes. Error = standard deviation of the mean. Average of n = 3 repeats, error = one standard deviation of the mean.

PE:PG 3:1 FP titrations

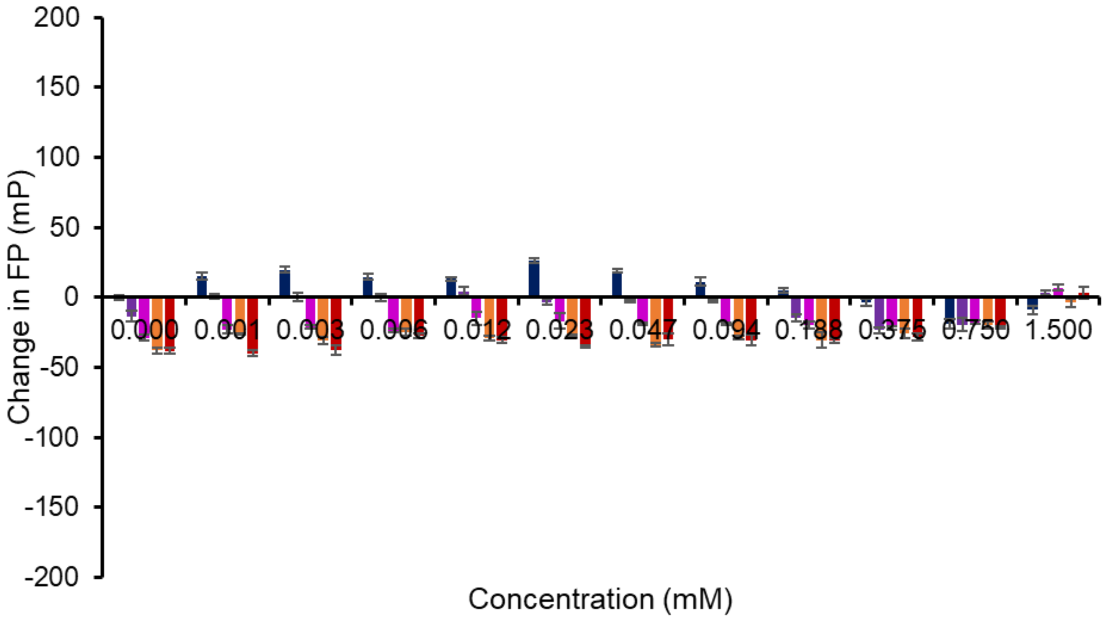

Figure S406 – Change in FP of PE:PG 3:1 vesicles incubated with DPH (30 µM) following addition of **1**. Blue = 1 minute, purple = 5 minutes, pink = 10 minutes, orange = 15 minutes, red = 20 minutes. Error = standard deviation of the mean. Average of n = 3 repeats, error = one standard deviation of the mean.

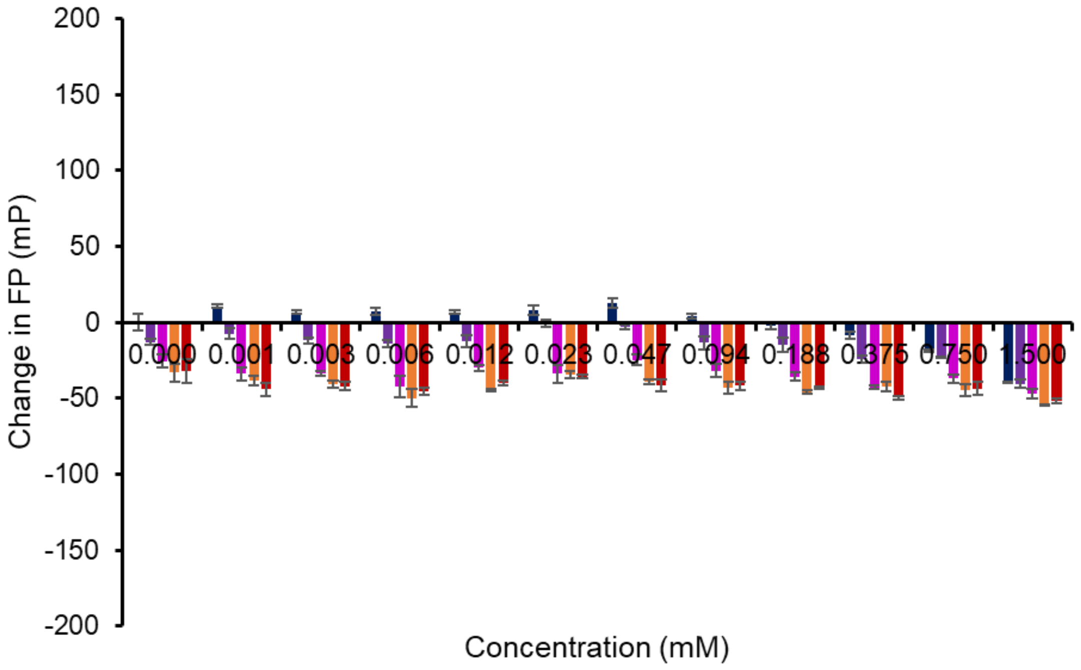

Figure S407 – Change in FP of PE:PG 3:1 vesicles incubated with DPH (30 µM) following addition of **2**. Blue = 1 minute, purple = 5 minutes, pink = 10 minutes, orange = 15 minutes, red = 20 minutes. Error = standard deviation of the mean. Average of n = 3 repeats, error = one standard deviation of the mean.

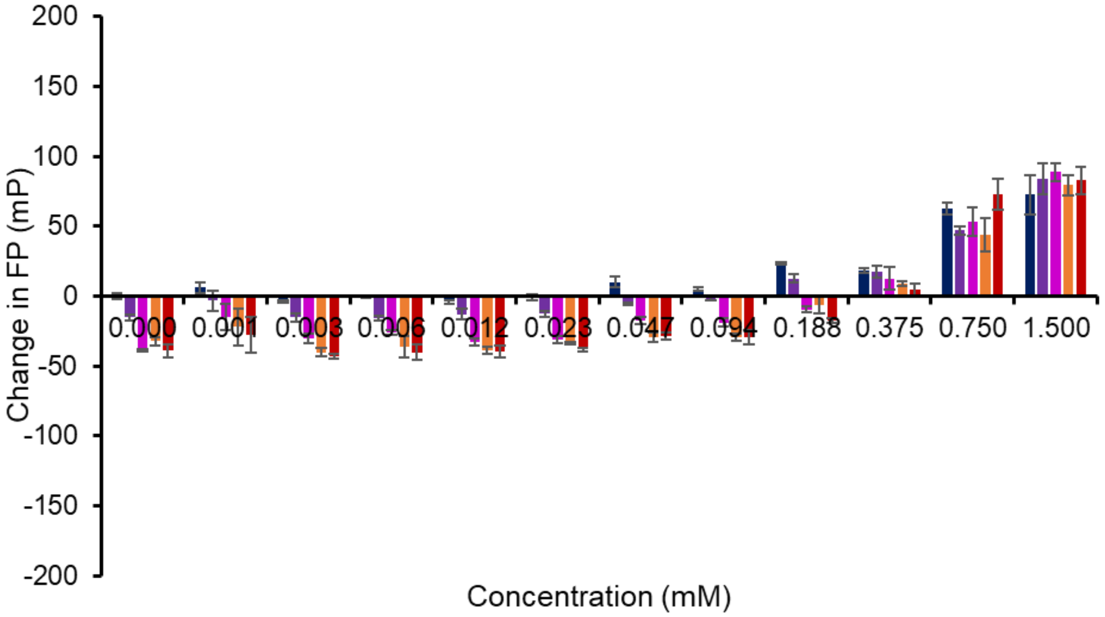

Figure S408 – Change in FP of PE:PG 3:1 vesicles incubated with DPH (30 µM) following addition of **4**. Blue = 1 minute, purple = 5 minutes, pink = 10 minutes, orange = 15 minutes, red = 20 minutes. Error = standard deviation of the mean. Average of n = 3 repeats, error = one standard deviation of the mean.

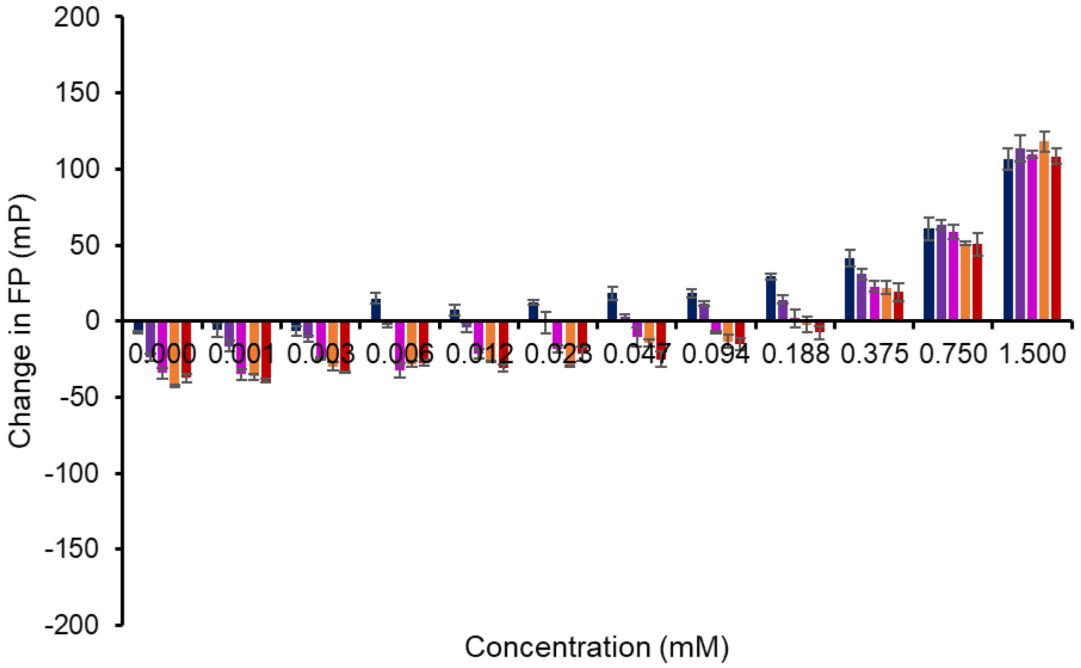

Figure S409 – Change in FP of PE:PG 3:1 vesicles incubated with DPH (30 µM) following addition of **5**. Blue = 1 minute, purple = 5 minutes, pink = 10 minutes, orange = 15 minutes, red = 20 minutes. Error = standard deviation of the mean. Average of n = 3 repeats, error = one standard deviation of the mean.

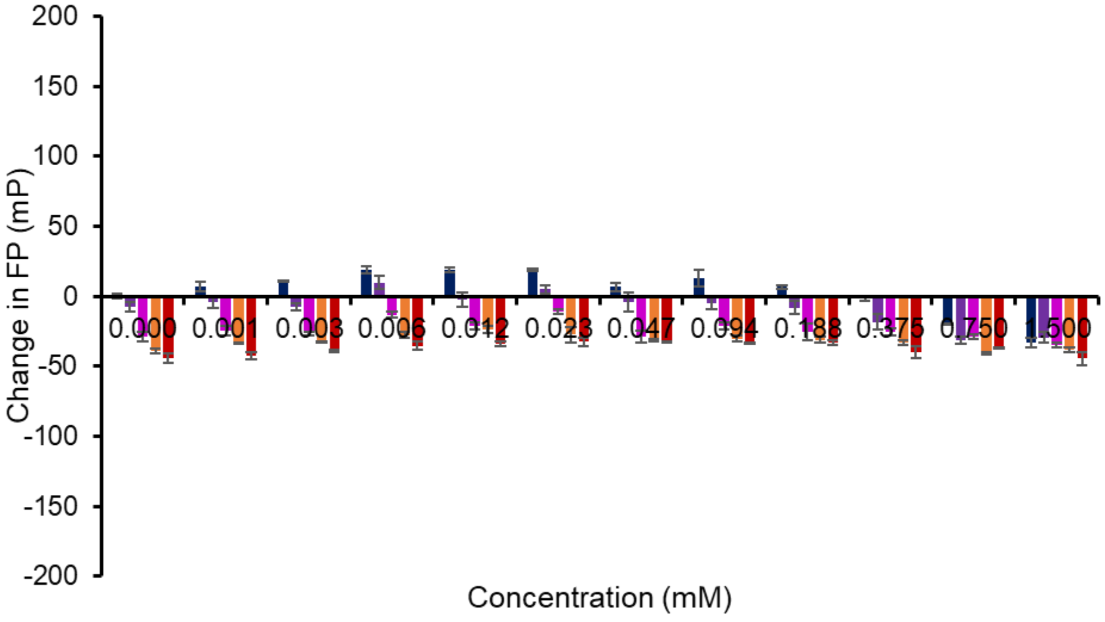

Figure S410 – Change in FP of PE:PG 3:1 vesicles incubated with DPH (30 µM) following addition of **a**. Blue = 1 minute, purple = 5 minutes, pink = 10 minutes, orange = 15 minutes, red = 20 minutes. Error = standard deviation of the mean. Average of n = 3 repeats, error = one standard deviation of the mean.

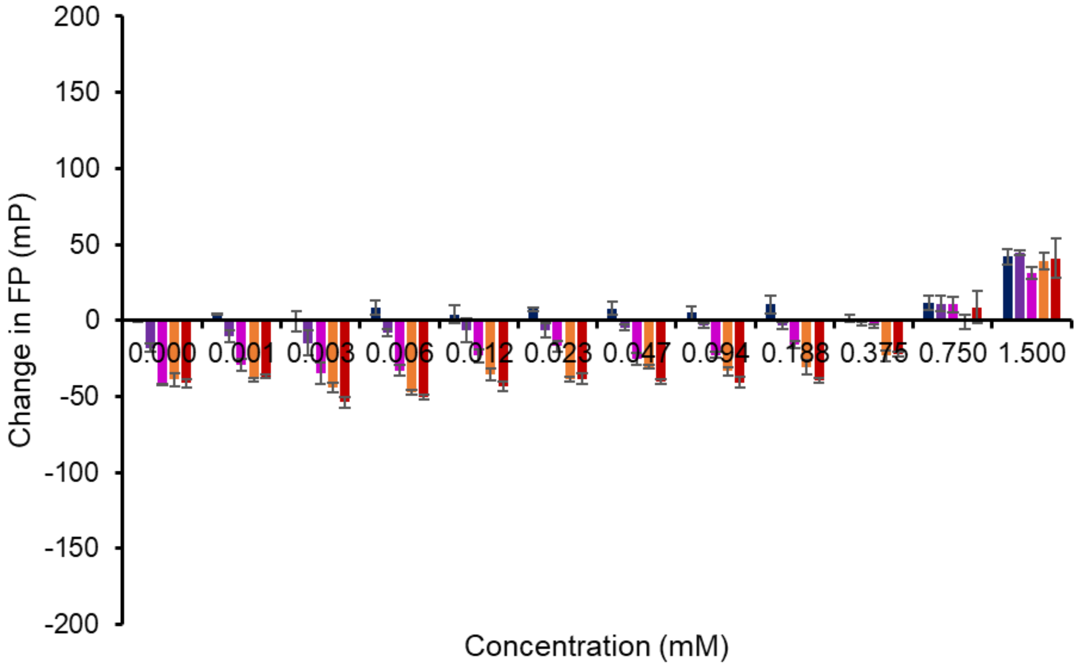

Figure S411 – Change in FP of PE:PG 3:1 vesicles incubated with DPH (30 µM) following addition of **c**. Blue = 1 minute, purple = 5 minutes, pink = 10 minutes, orange = 15 minutes, red = 20 minutes. Error = standard deviation of the mean. Average of n = 3 repeats, error = one standard deviation of the mean.

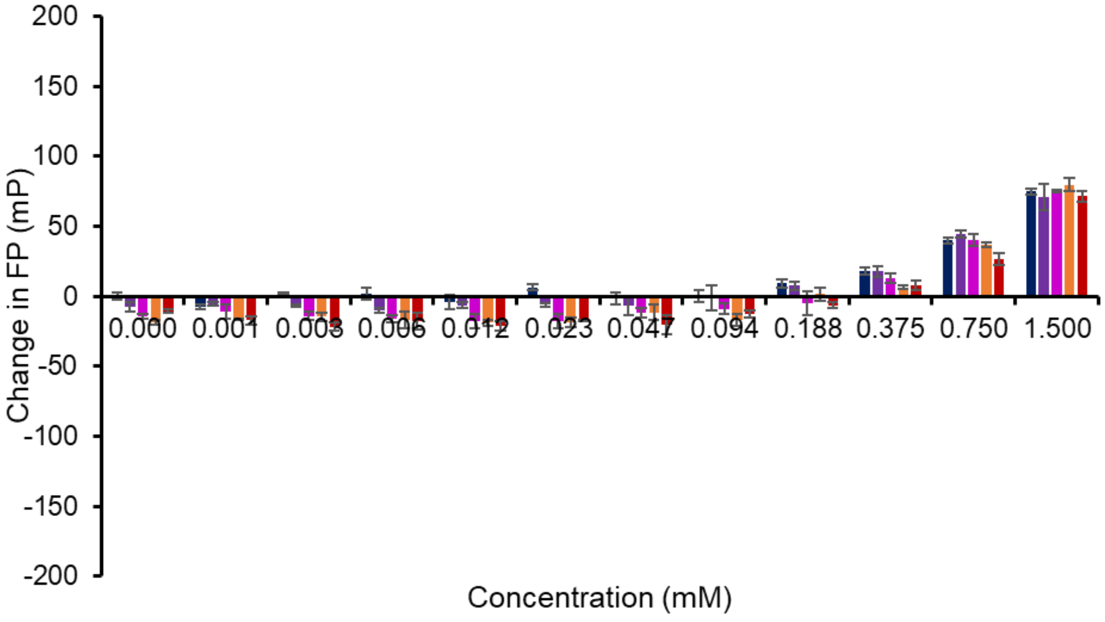

Figure S412 – Change in FP of PE:PG 3:1 vesicles incubated with DPH (30 μM) following addition of **d**. Blue = 1 minute, purple = 5 minutes, pink = 10 minutes, orange = 15 minutes, red = 20 minutes. Error = standard deviation of the mean. Average of n = 3 repeats, error = one standard deviation of the mean.

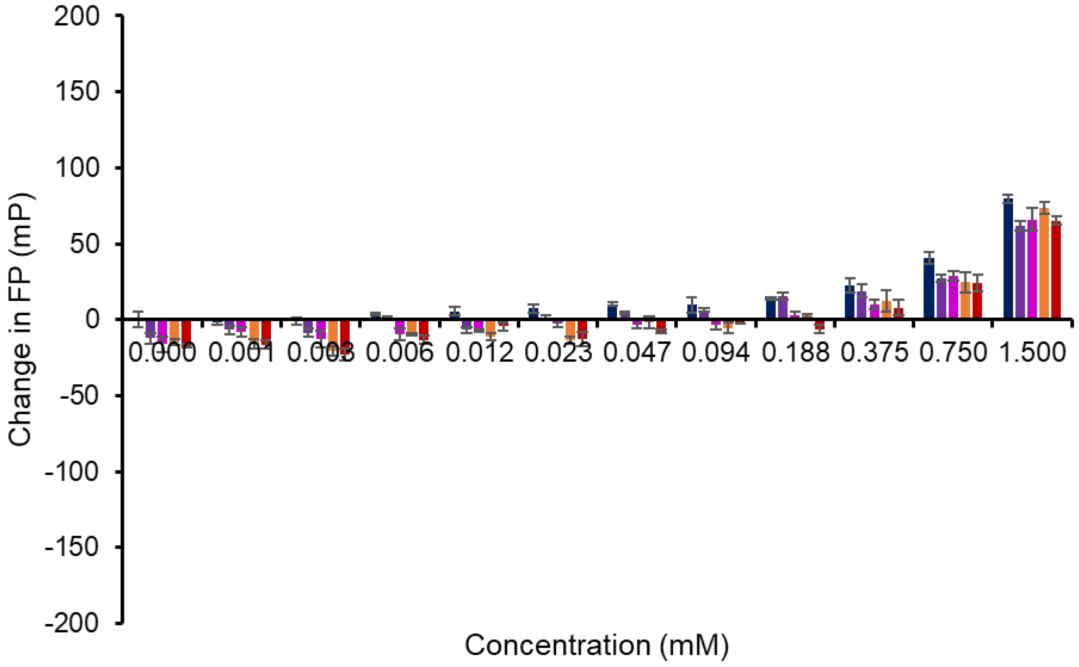

Figure S413 – Change in FP of PE:PG 3:1 vesicles incubated with DPH (30 μM) following addition of **f**. Blue = 1 minute, purple = 5 minutes, pink = 10 minutes, orange = 15 minutes, red = 20 minutes. Error = standard deviation of the mean. Average of n = 3 repeats, error = one standard deviation of the mean.

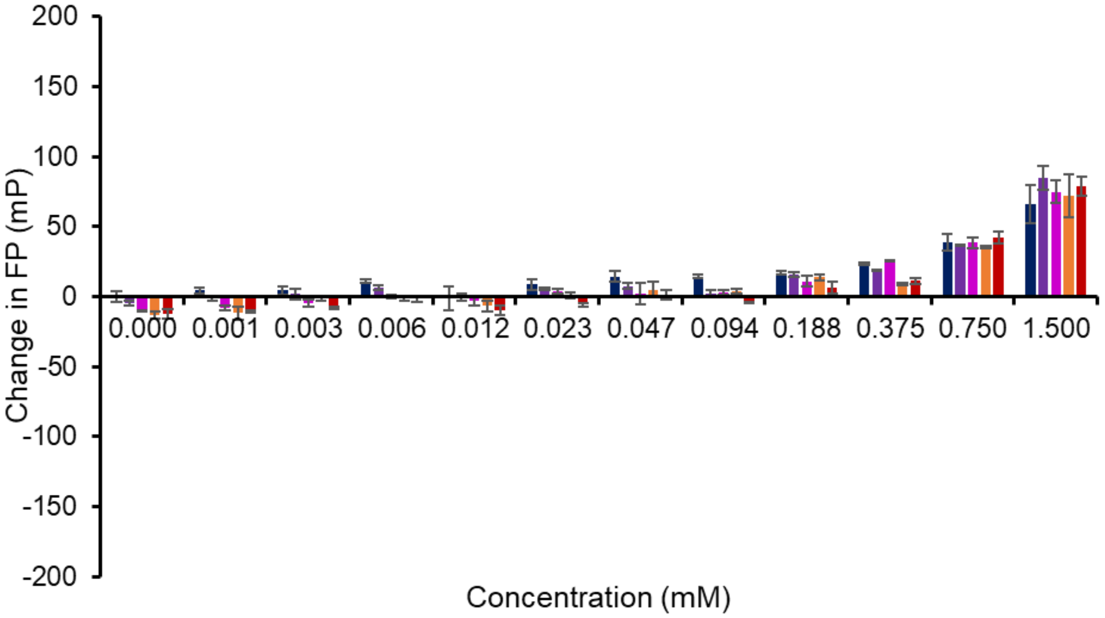

Figure S414 – Change in FP of PE:PG 3:1 vesicles incubated with DPH (30 µM) following addition of **g**. Blue = 1 minute, purple = 5 minutes, pink = 10 minutes, orange = 15 minutes, red = 20 minutes. Error = standard deviation of the mean. Average of n = 3 repeats, error = one standard deviation of the mean.

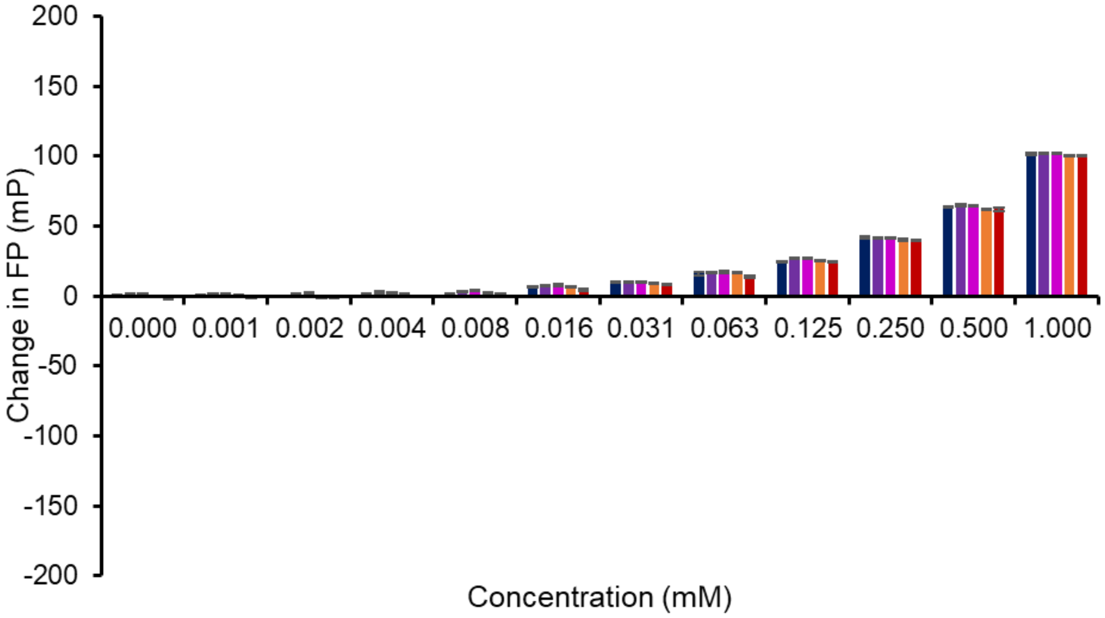

Figure S415 – Change in FP of PE:PG 3:1 vesicles incubated with DPH (30 µM) following addition of **j**. Blue = 1 minute, purple = 5 minutes, pink = 10 minutes, orange = 15 minutes, red = 20 minutes. Error = standard deviation of the mean. Average of n = 3 repeats, error = one standard deviation of the mean.

PE:PG 1:1 FP titrations

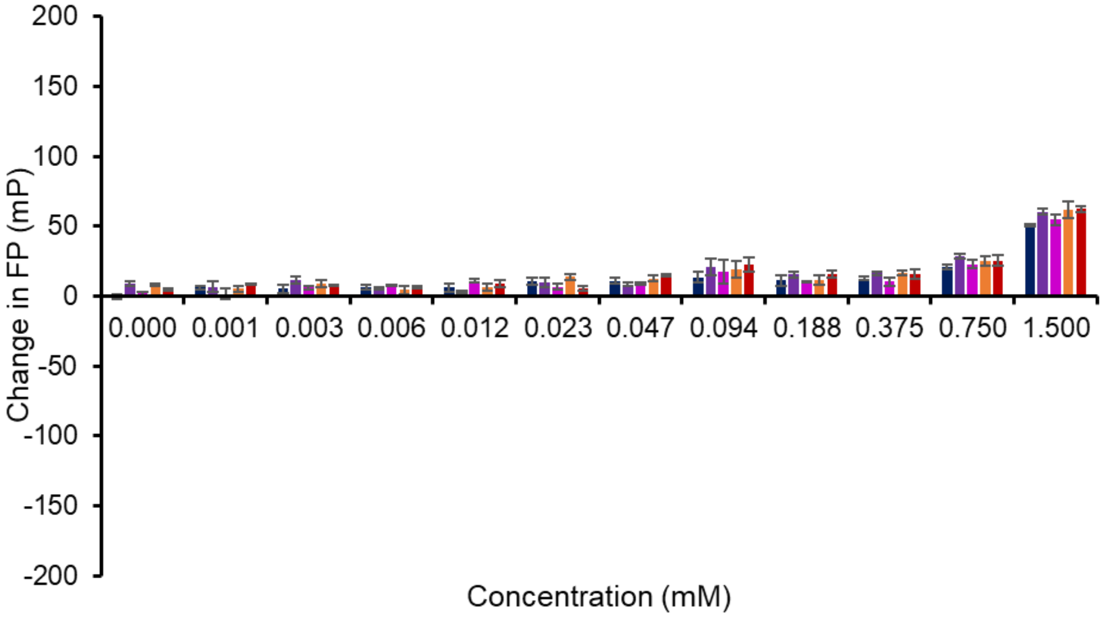

Figure S416 – Change in FP of PE:PG 1:1 vesicles incubated with DPH (30 µM) following addition of **1**. Blue = 1 minute, purple = 5 minutes, pink = 10 minutes, orange = 15 minutes, red = 20 minutes. Error = standard deviation of the mean. Average of n = 3 repeats, error = one standard deviation of the mean.

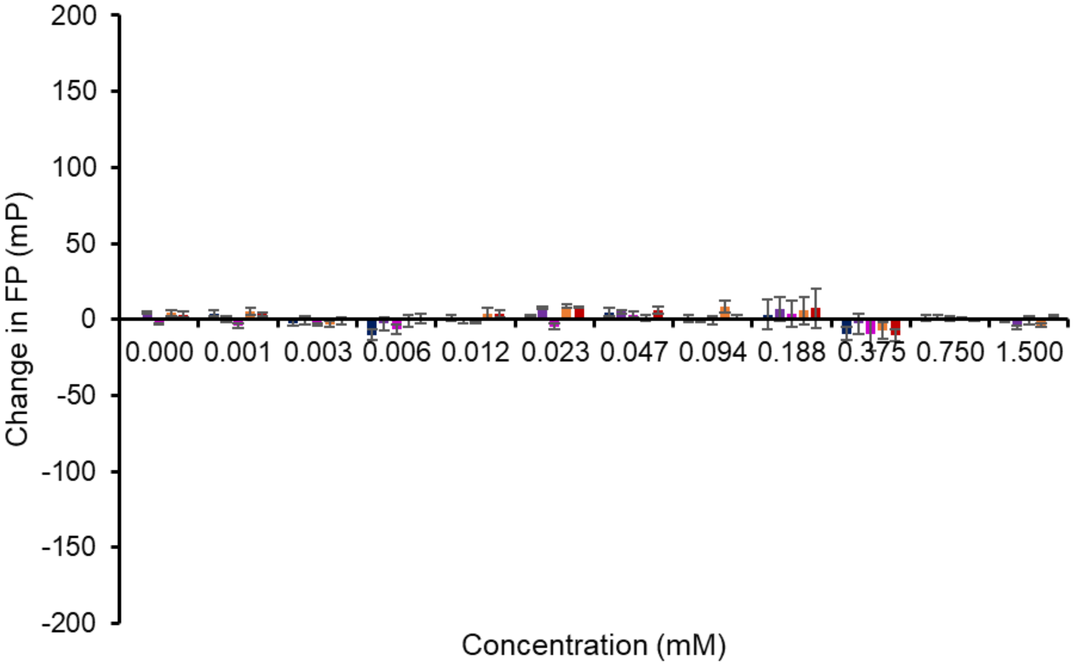

Figure S417 – Change in FP of PE:PG 1:1 vesicles incubated with DPH (30 µM) following addition of **2**. Blue = 1 minute, purple = 5 minutes, pink = 10 minutes, orange = 15 minutes, red = 20 minutes. Error = standard deviation of the mean. Average of n = 3 repeats, error = one standard deviation of the mean.

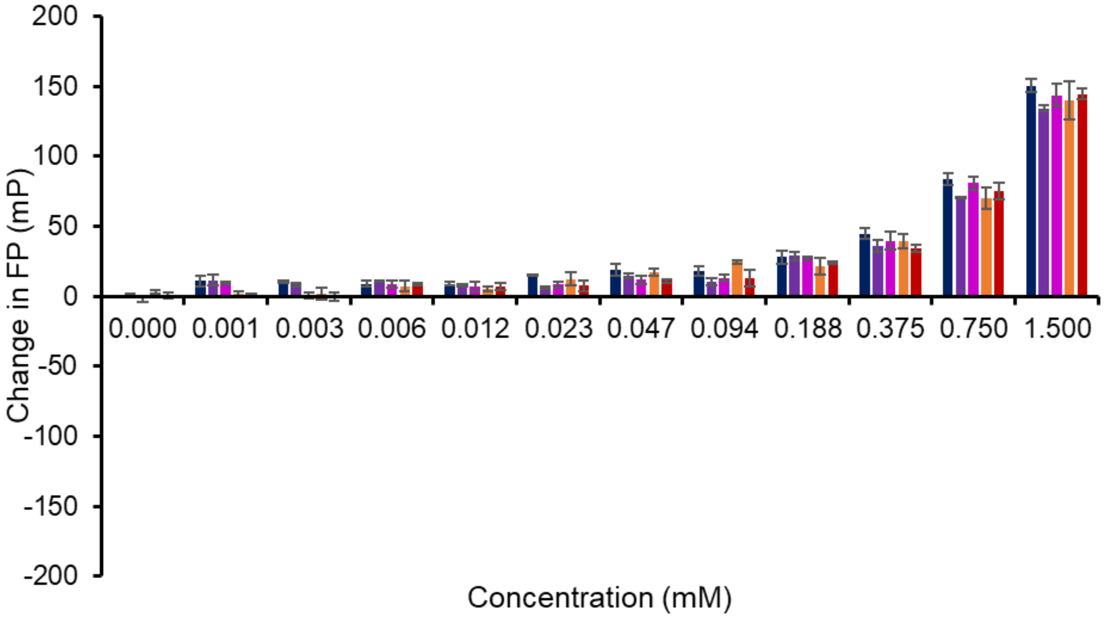

Figure S418 – Change in FP of PE:PG 1:1 vesicles incubated with DPH (30 µM) following addition of **4**. Blue = 1 minute, purple = 5 minutes, pink = 10 minutes, orange = 15 minutes, red = 20 minutes. Error = standard deviation of the mean. Average of n = 3 repeats, error = one standard deviation of the mean.

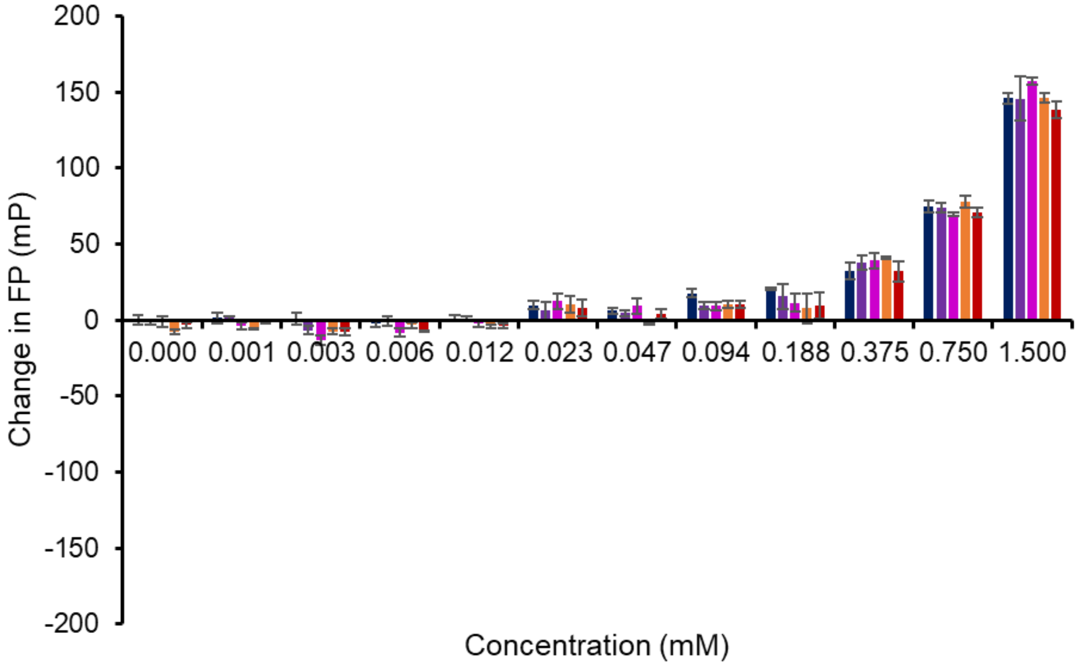

Figure S419 – Change in FP of PE:PG 1:1 vesicles incubated with DPH (30 µM) following addition of **5**. Blue = 1 minute, purple = 5 minutes, pink = 10 minutes, orange = 15 minutes, red = 20 minutes. Error = standard deviation of the mean. Average of n = 3 repeats, error = one standard deviation of the mean.

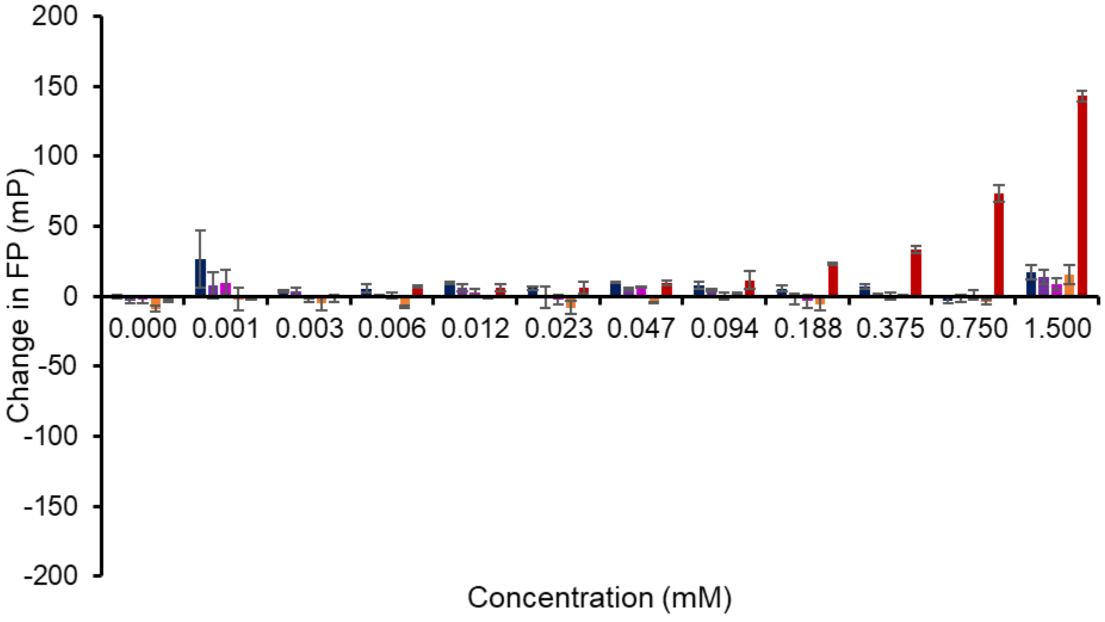

Figure S420 – Change in FP of PE:PG 1:1 vesicles incubated with DPH (30 µM) following addition of **a**. Blue = 1 minute, purple = 5 minutes, pink = 10 minutes, orange = 15 minutes, red = 20 minutes. Error = standard deviation of the mean. Average of n = 3 repeats, error = one standard deviation of the mean.

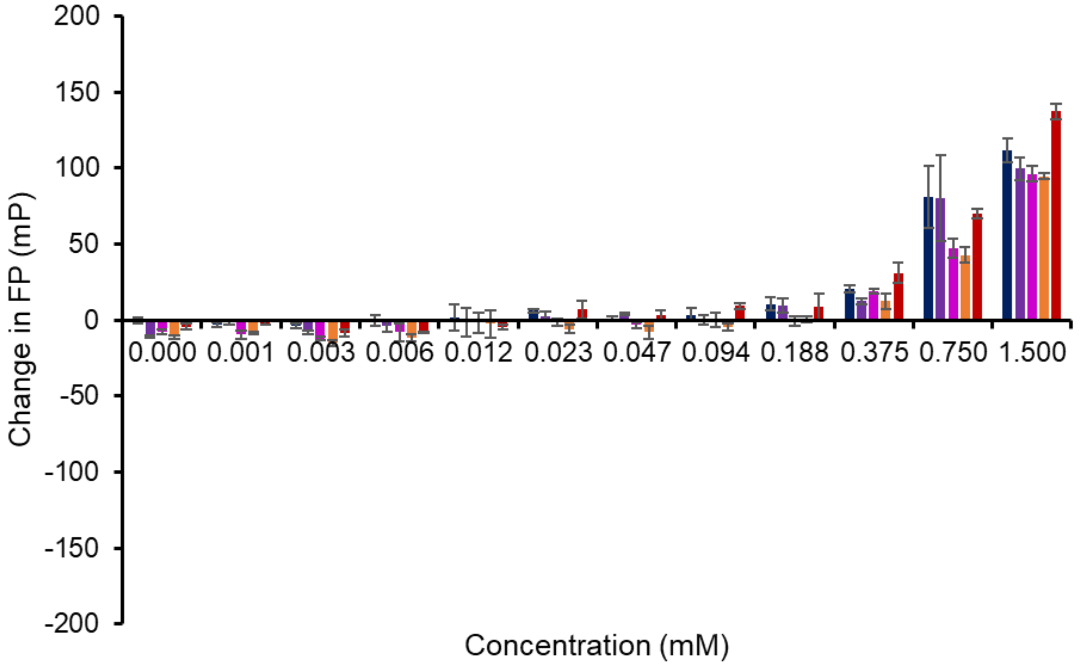

Figure S421 – Change in FP of PE:PG 1:1 vesicles incubated with DPH (30 µM) following addition of **c**. Blue = 1 minute, purple = 5 minutes, pink = 10 minutes, orange = 15 minutes, red = 20 minutes. Error = standard deviation of the mean. Average of n = 3 repeats, error = one standard deviation of the mean.

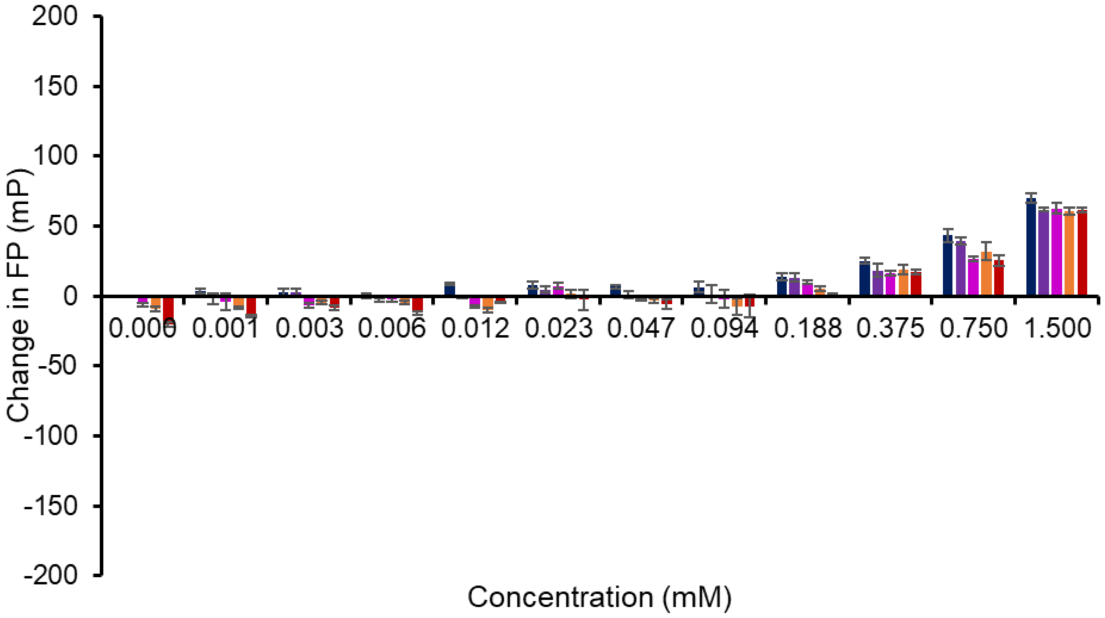

Figure S422 – Change in FP of PE:PG 1:1 vesicles incubated with DPH (30 µM) following addition of **d**. Blue = 1 minute, purple = 5 minutes, pink = 10 minutes, orange = 15 minutes, red = 20 minutes. Error = standard deviation of the mean. Average of n = 3 repeats, error = one standard deviation of the mean.

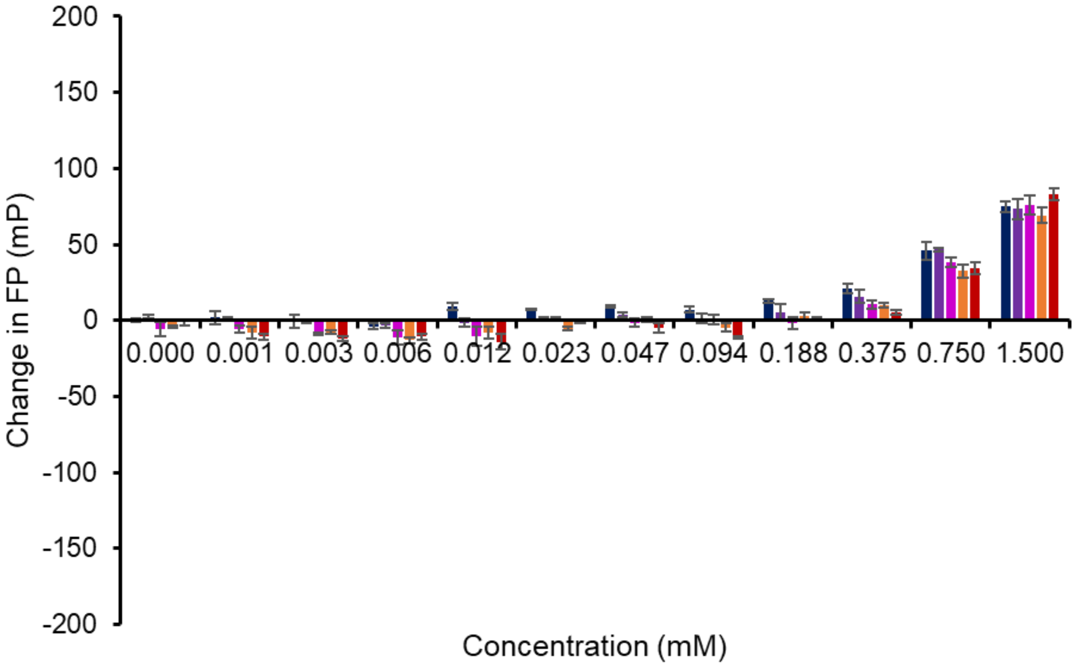

Figure S423 – Change in FP of PE:PG 1:1 vesicles incubated with DPH (30 µM) following addition of **f**. Blue = 1 minute, purple = 5 minutes, pink = 10 minutes, orange = 15 minutes, red = 20 minutes. Error = standard deviation of the mean. Average of n = 3 repeats, error = one standard deviation of the mean.

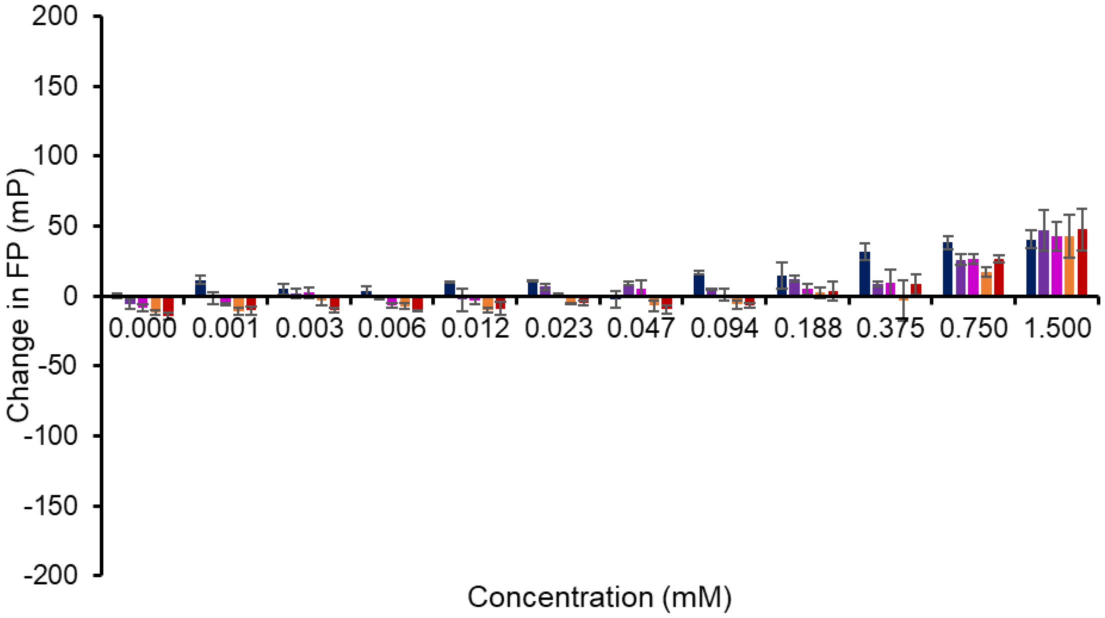

Figure S424 – Change in FP of PE:PG 1:1 vesicles incubated with DPH (30 µM) following addition of **g**. Blue = 1 minute, purple = 5 minutes, pink = 10 minutes, orange = 15 minutes, red = 20 minutes. Error = standard deviation of the mean. Average of n = 3 repeats, error = one standard deviation of the mean.

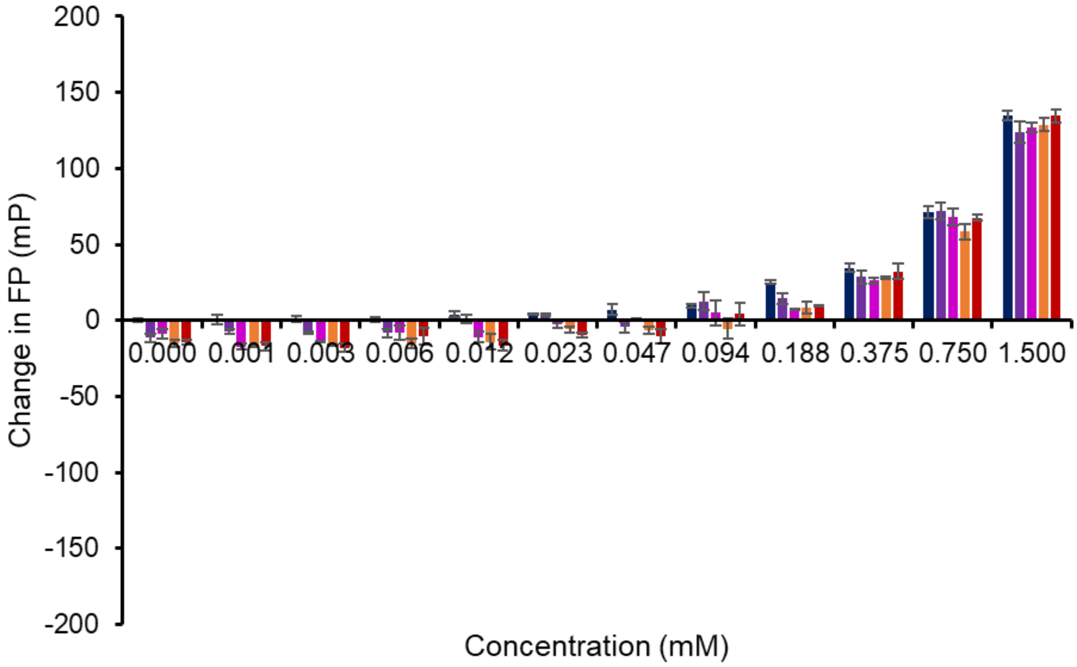

Figure S425 – Change in FP of PE:PG 1:1 vesicles incubated with DPH (30 µM) following addition of **j**. Blue = 1 minute, purple = 5 minutes, pink = 10 minutes, orange = 15 minutes, red = 20 minutes. Error = standard deviation of the mean. Average of n = 3 repeats, error = one standard deviation of the mean.

Comparison of membrane fluidity FP data

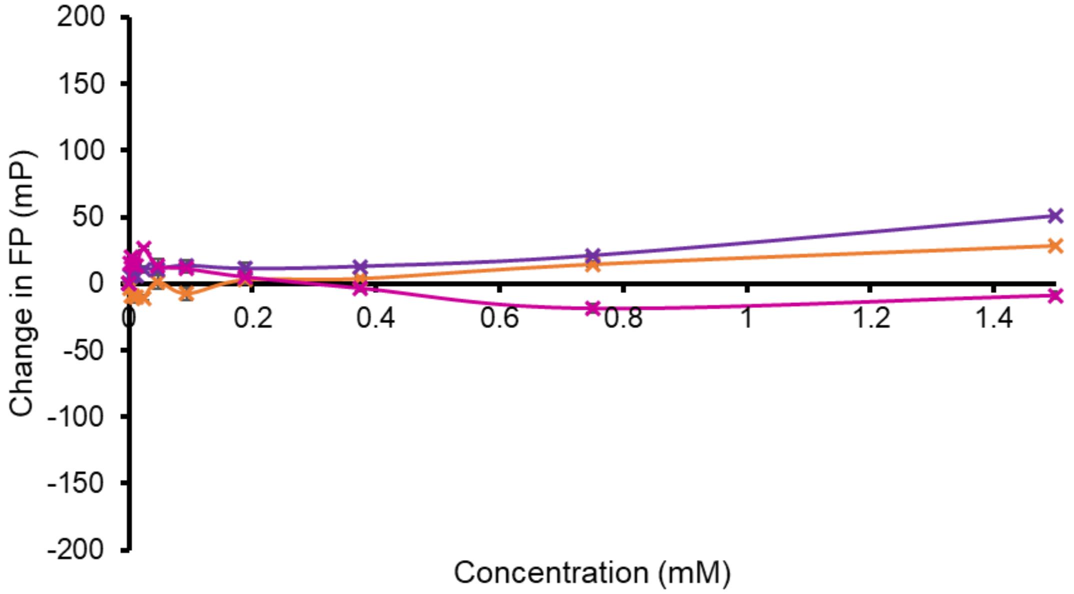

Figure S426 – Change in FP of different ratios of lipids incubated with DPH (30 µM) following the addition of **1** at 1 minute. Orange = PG, pink = PE:PG 3:1, purple = PE:PG 1:1. Average of n = 3 repeats. Error = one standard deviation of the mean.

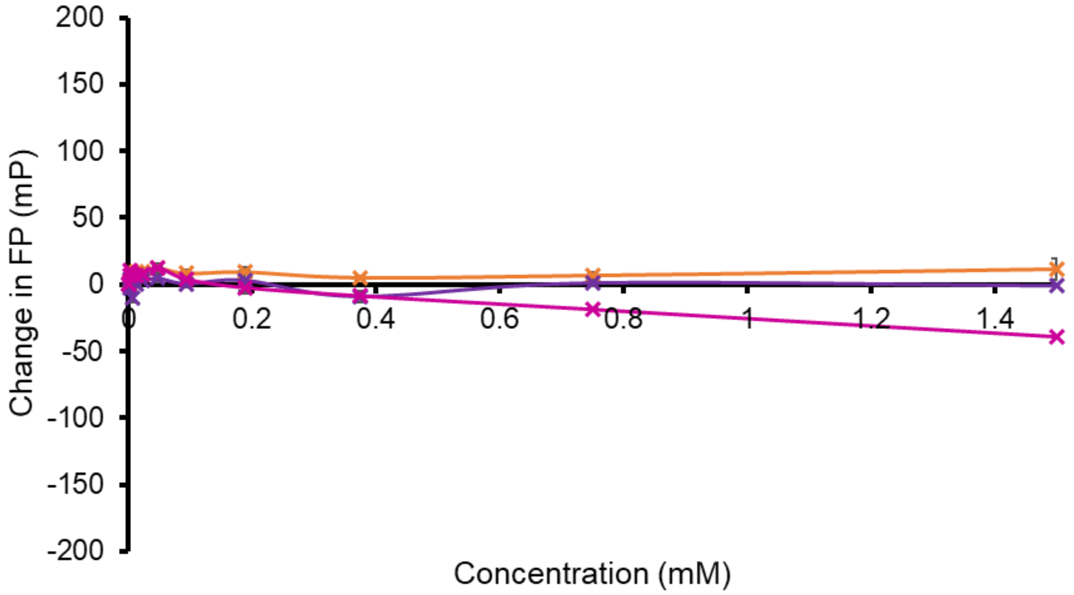

Figure S427 – Change in FP of different ratios of lipids incubated with DPH (30 µM) following the addition of **2** at 1 minute. Orange = PG, pink = PE:PG 3:1, purple = PE:PG 1:1. Average of n = 3 repeats. Error = one standard deviation of the mean.

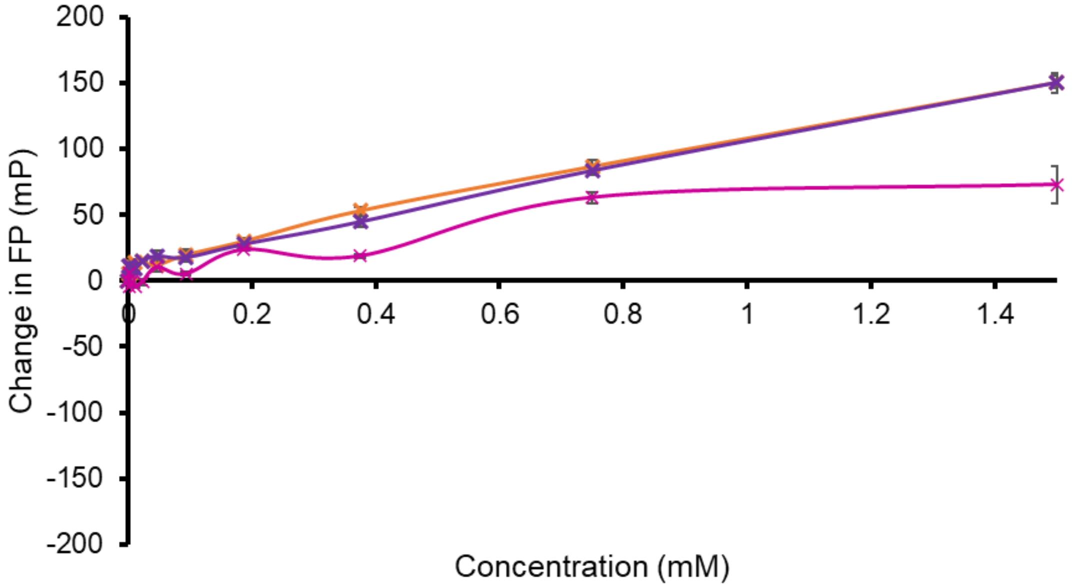

Figure S428 – Change in FP of different ratios of lipids incubated with DPH (30 µM) following the addition of **4** at 1 minute. Orange = PG, pink = PE:PG 3:1, purple = PE:PG 1:1. Average of n = 3 repeats. Error = one standard deviation of the mean.

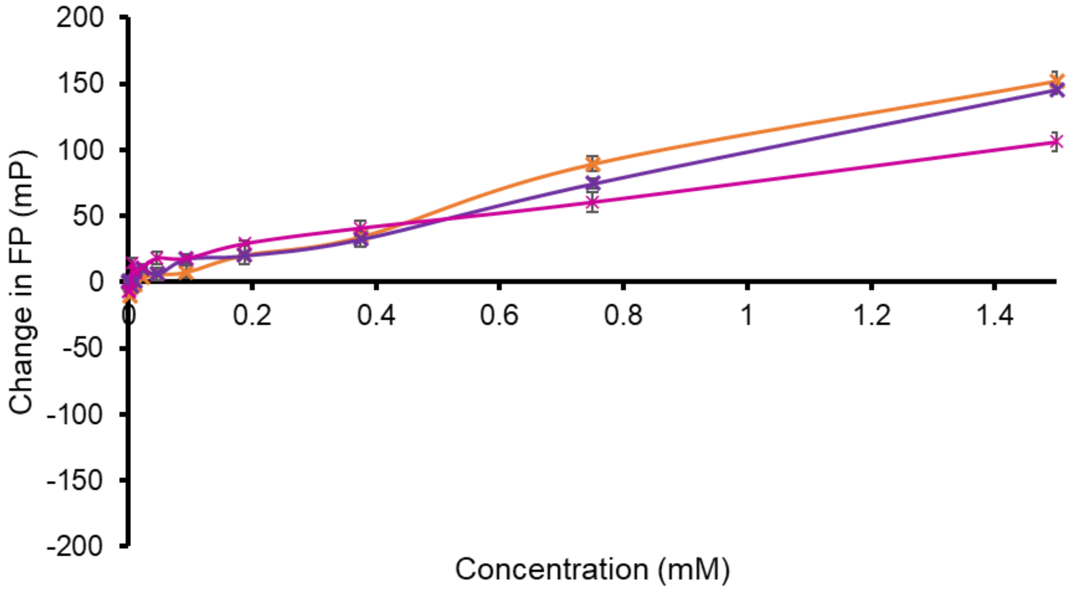

Figure S429 – Change in FP of different ratios of lipids incubated with DPH (30 µM) following the addition of **5** at 1 minute. Orange = PG, pink = PE:PG 3:1, purple = PE:PG 1:1. Average of n = 3 repeats. Error = one standard deviation of the mean.

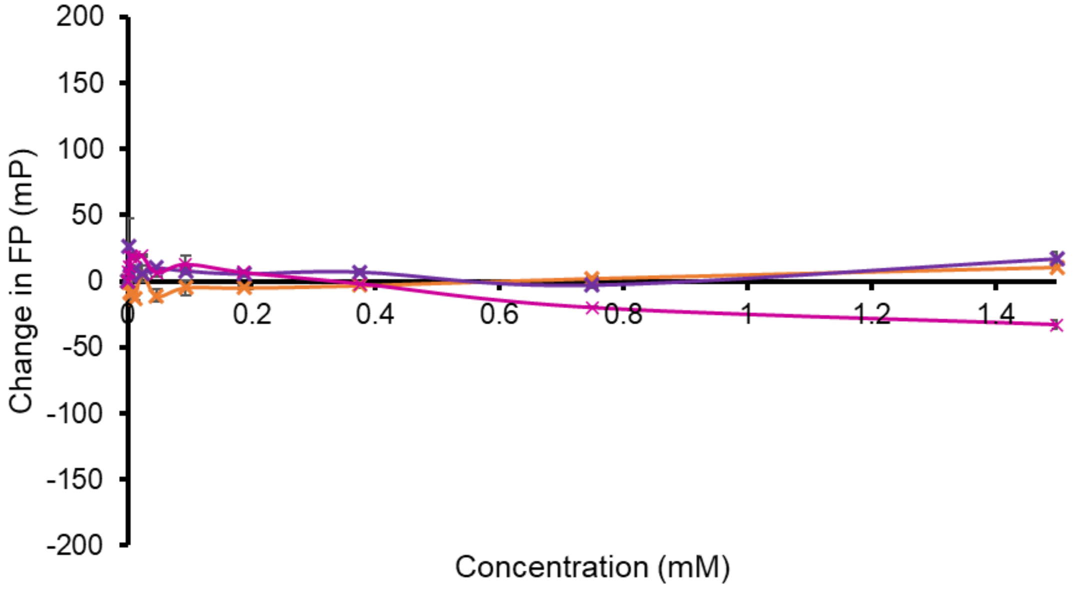

Figure S430 – Change in FP of different ratios of lipids incubated with DPH (30 μM) following the addition of **a** at 1 minute. Orange = PG, pink = PE:PG 3:1, purple = PE:PG 1:1. Average of n = 3 repeats. Error = one standard deviation of the mean.

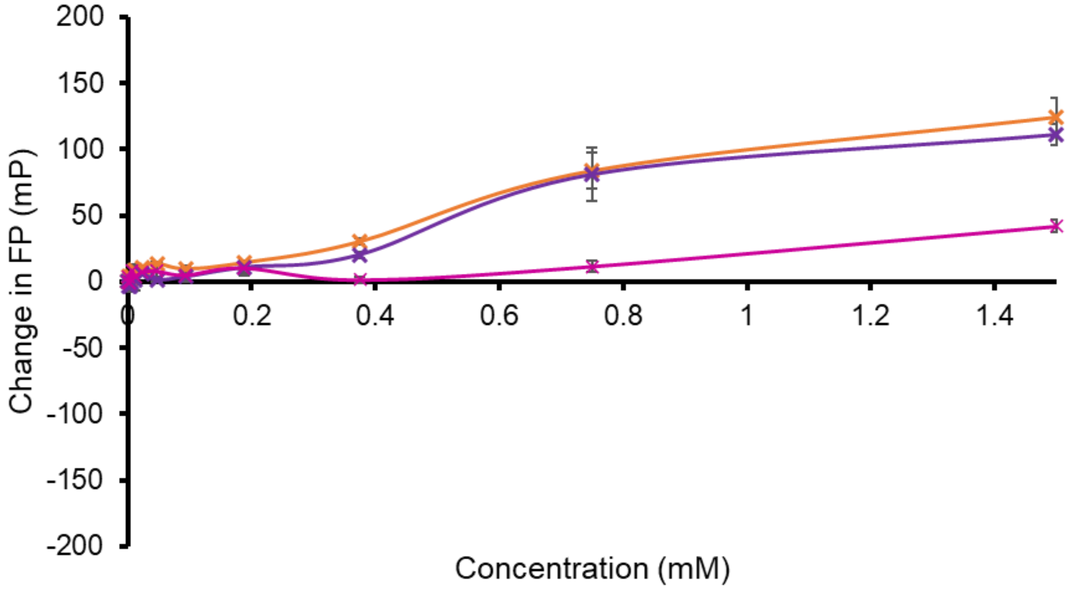

Figure S431 – Change in FP of different ratios of lipids incubated with DPH (30 μM) following the addition of **c** at 1 minute. Orange = PG, pink = PE:PG 3:1, purple = PE:PG 1:1. Average of n = 3 repeats. Error = one standard deviation of the mean.

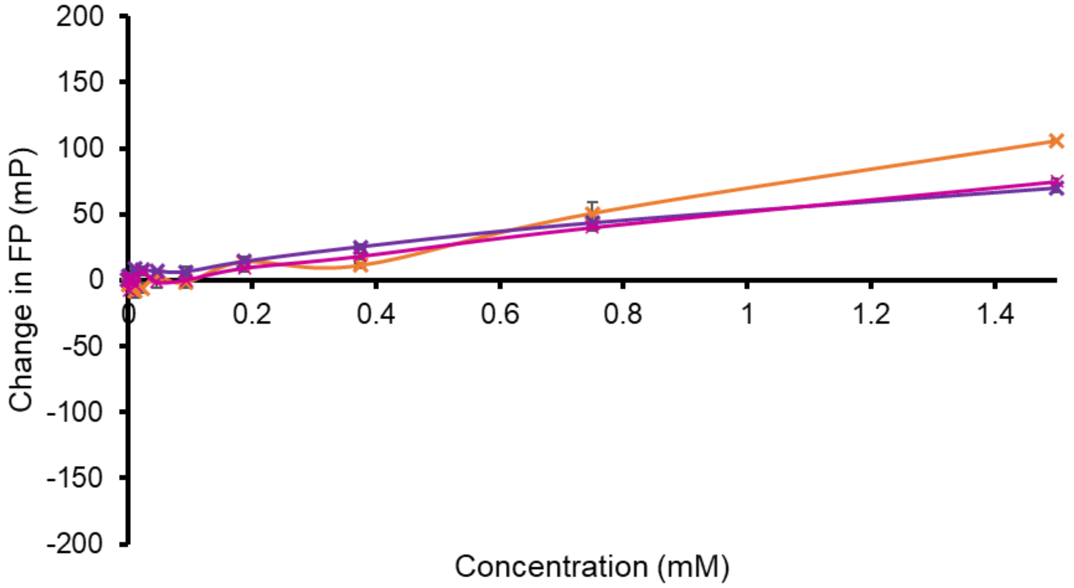

Figure S432 – Change in FP of different ratios of lipids incubated with DPH (30 µM) following the addition of **d** at 1 minute. Orange = PG, pink = PE:PG 3:1, purple = PE:PG 1:1. Average of n = 3 repeats. Error = one standard deviation of the mean.

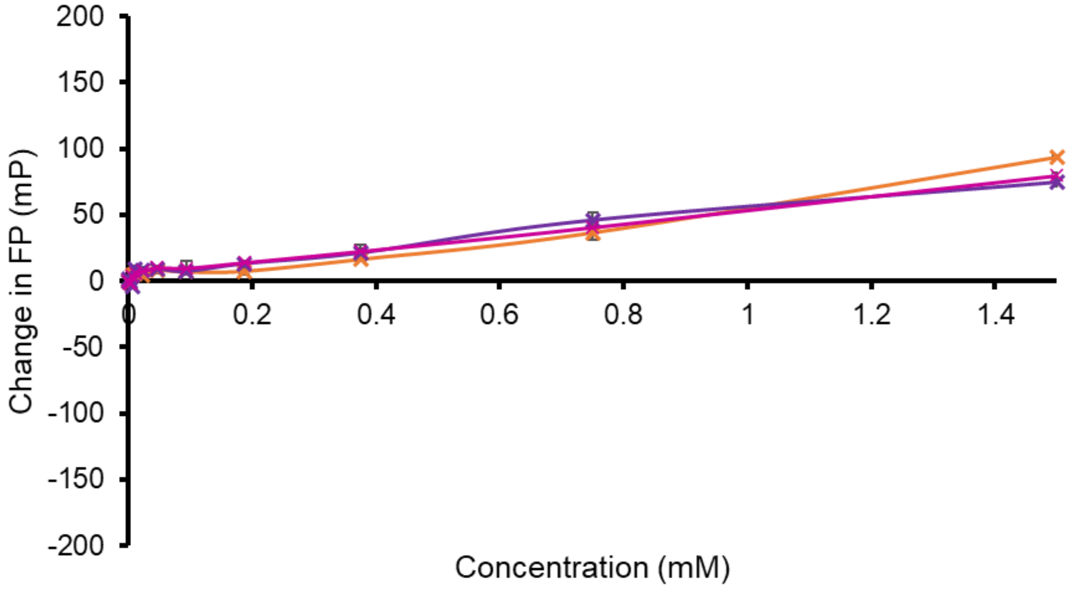

Figure S433 – Change in FP of different ratios of lipids incubated with DPH (30 µM) following the addition of **f** at 1 minute. Orange = PG, pink = PE:PG 3:1, purple = PE:PG 1:1. Average of n = 3 repeats. Error = one standard deviation of the mean.

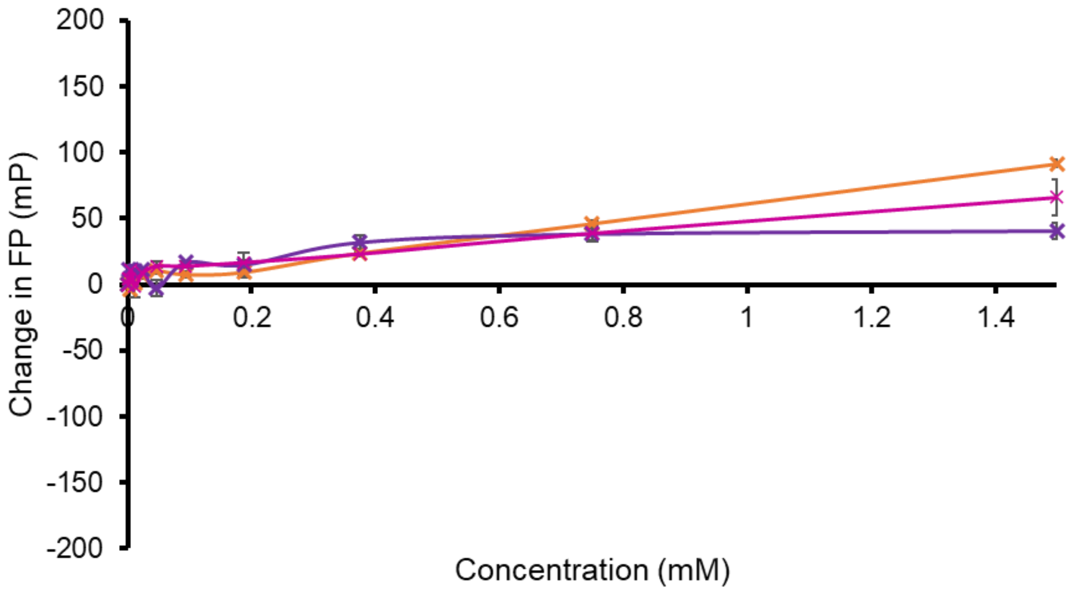

Figure S434 – Change in FP of different ratios of lipids incubated with DPH (30 µM) following the addition of **g** at 1 minute. Orange = PG, pink = PE:PG 3:1, purple = PE:PG 1:1. Average of n = 3 repeats. Error = one standard deviation of the mean.

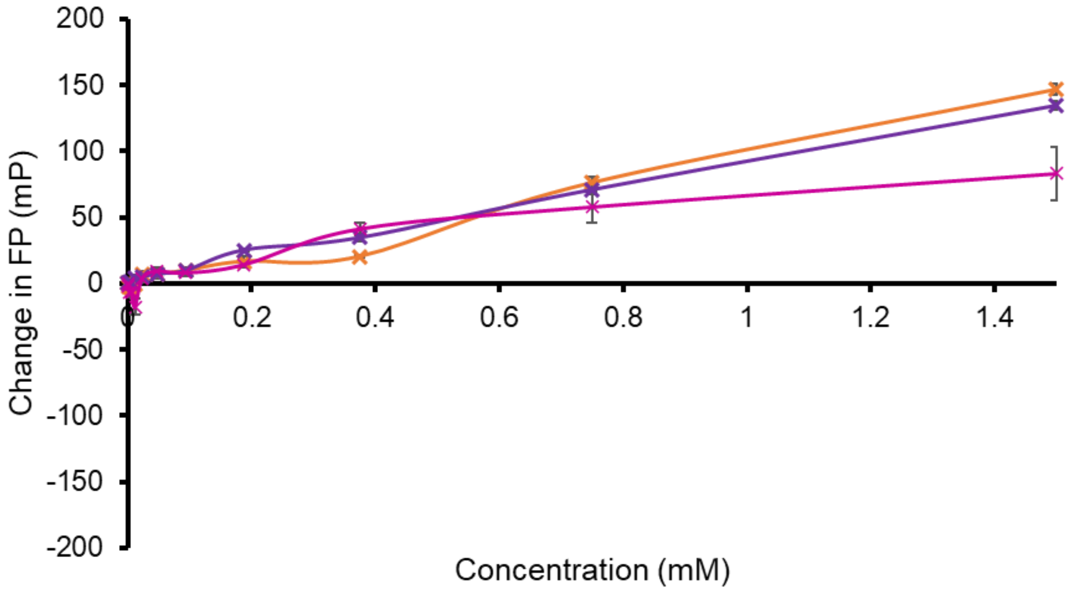

Figure S435 – Change in FP of different ratios of lipids incubated with DPH (30 µM) following the addition of **j** at 1 minute. Orange = PG, pink = PE:PG 3:1, purple = PE:PG 1:1. Average of n = 3 repeats. Error = one standard deviation of the mean.

Section S16: Vesicle adhesion data

PG FP titrations

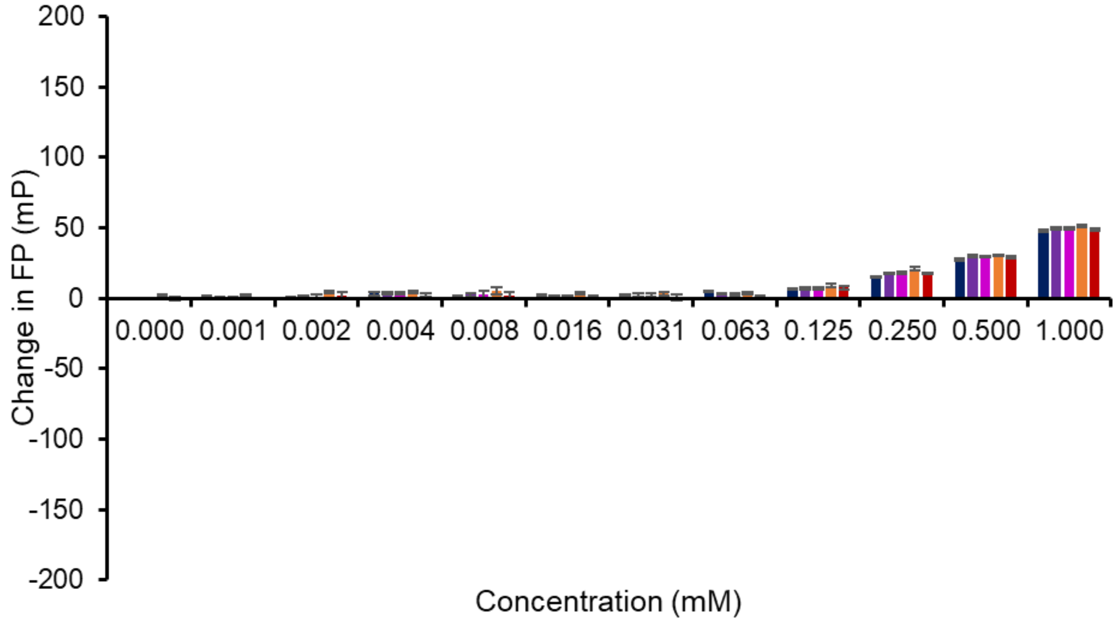

Figure S436 – Change in FP of **3** (0.15 mM) following addition of PG vesicles. Blue = 1 minute, purple = 5 minutes, pink = 10 minutes, orange = 15 minutes, red = 20 minutes. Average of n = 3 repeats. Error = one standard deviation of the mean.

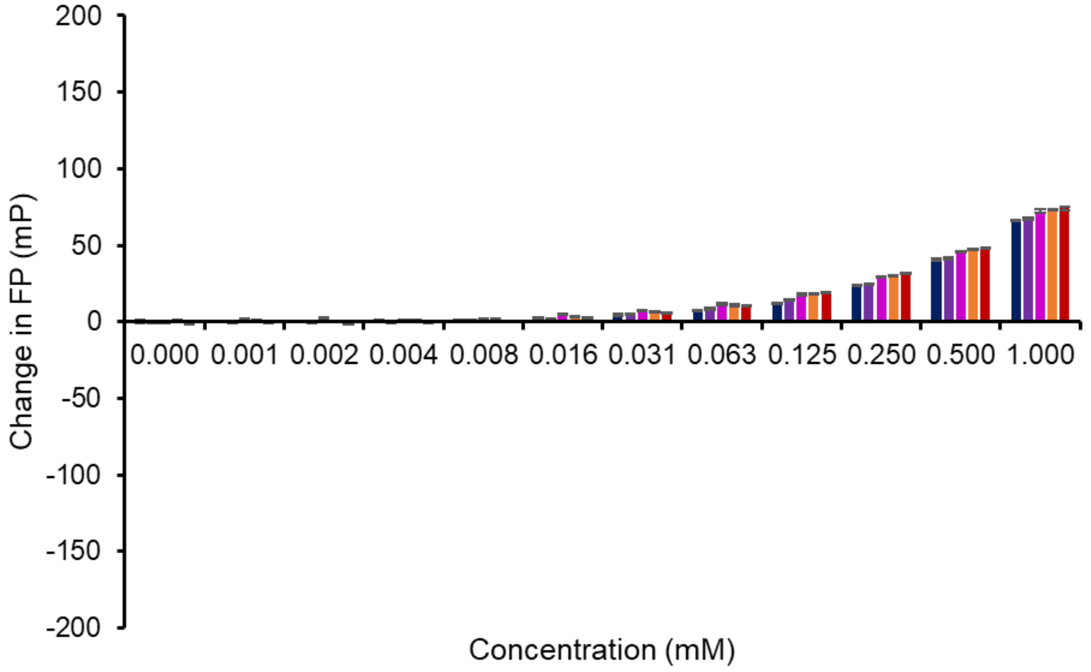

Figure S437 – Change in FP of **b** (0.15 mM) following addition of PG vesicles. Blue = 1 minute, purple = 5 minutes, pink = 10 minutes, orange = 15 minutes, red = 20 minutes. Average of n = 3 repeats. Error = one standard deviation of the mean.

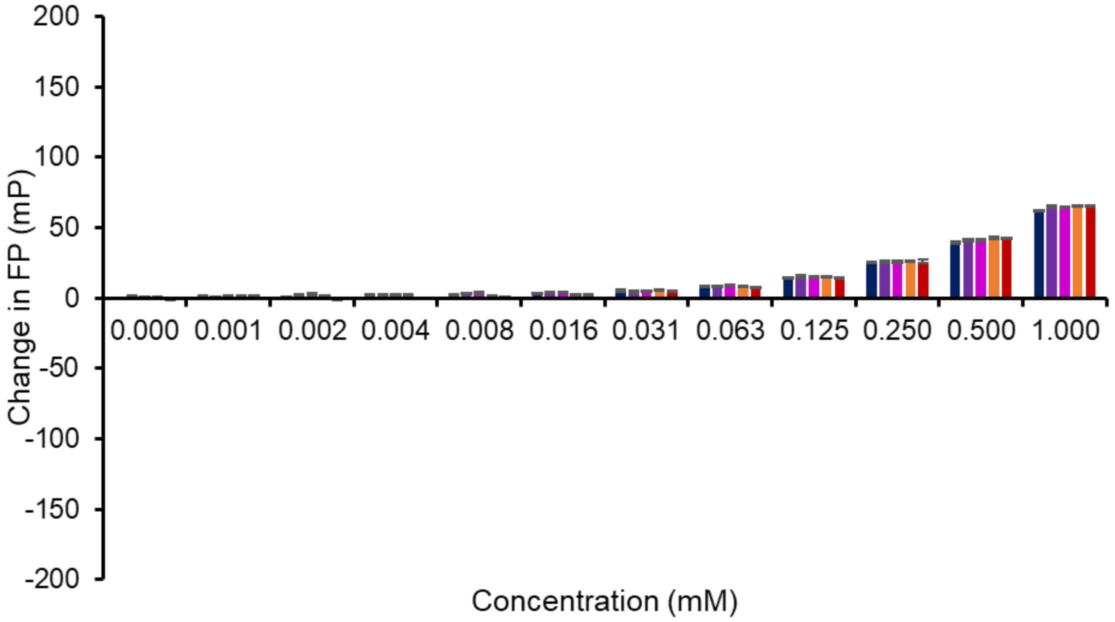

Figure S438 – Change in FP of **e** (0.15 mM) following addition of PG vesicles. Blue = 1 minute, purple = 5 minutes, pink = 10 minutes, orange = 15 minutes, red = 20 minutes. Average of n = 3 repeats. Error = one standard deviation of the mean.

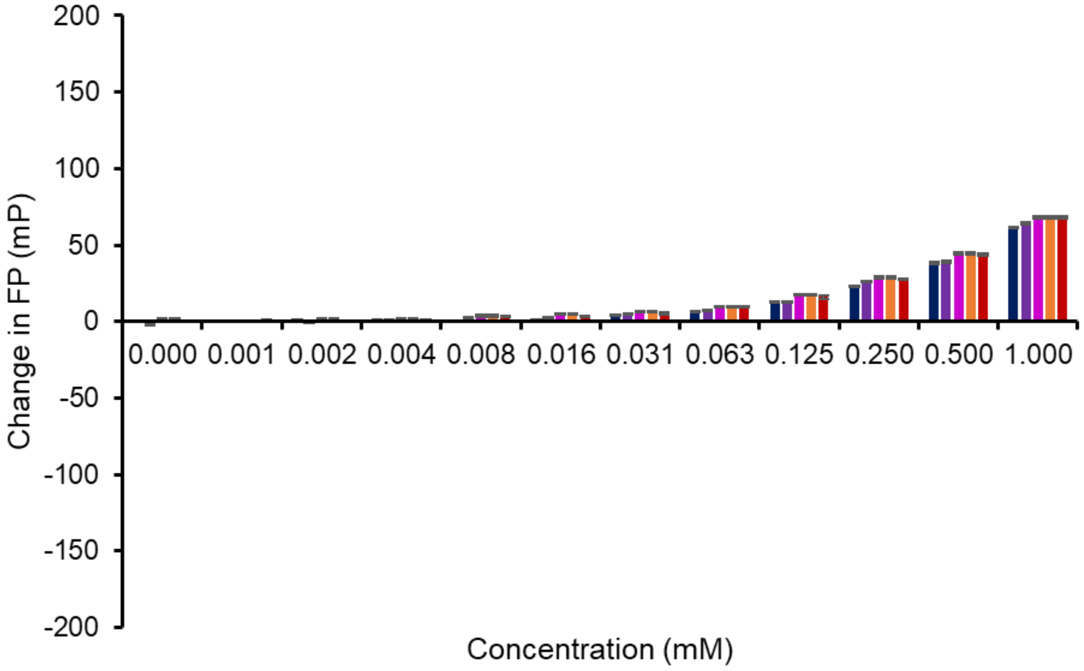

Figure S439 – Change in FP of **h** (0.15 mM) following addition of PG vesicles. Blue = 1 minute, purple = 5 minutes, pink = 10 minutes, orange = 15 minutes, red = 20 minutes. Average of n = 3 repeats. Error = one standard deviation of the mean.

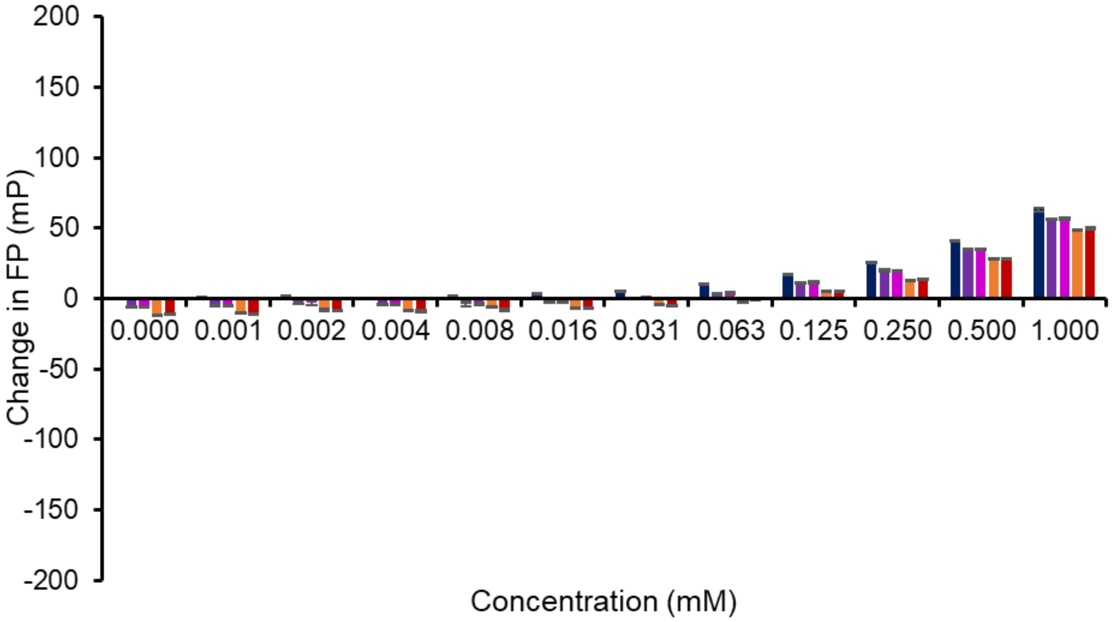

Figure S440 – Change in FP of **i** (0.15 mM) following addition of PG vesicles. Blue = 1 minute, purple = 5 minutes, pink = 10 minutes, orange = 15 minutes, red = 20 minutes. Average of n = 3 repeats. Error = one standard deviation of the mean.

PE:PG 3:1 FP titrations

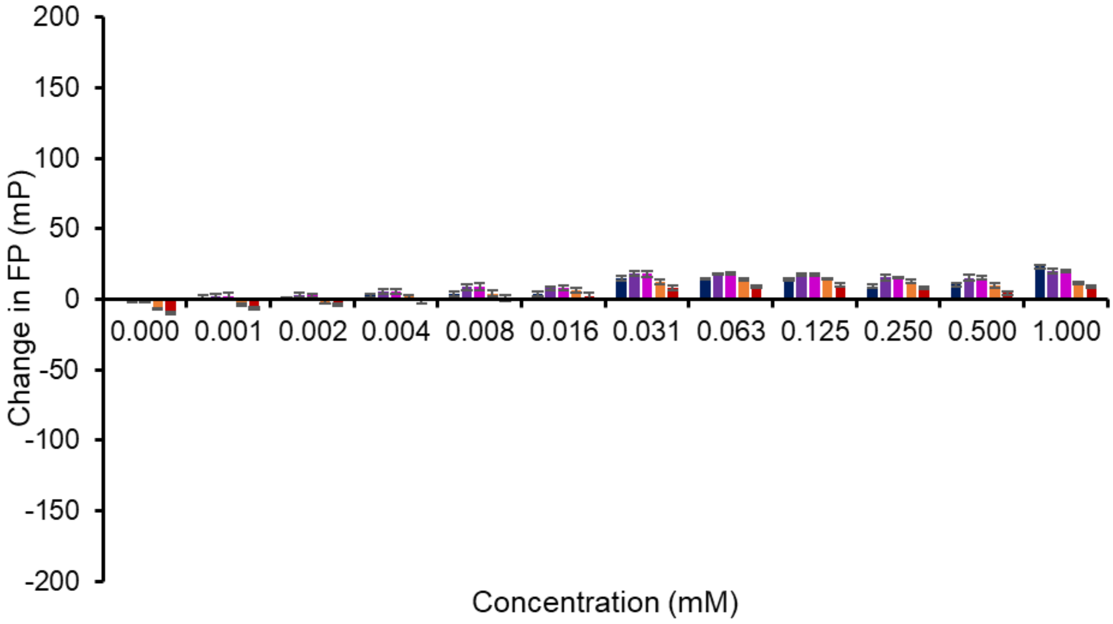

Figure S441 – Change in FP of **3** (0.15 mM) following addition of PE:PG 3:1 vesicles. Blue = 1 minute, purple = 5 minutes, pink = 10 minutes, orange = 15 minutes, red = 20 minutes. Average of n = 3 repeats. Error = one standard deviation of the mean.

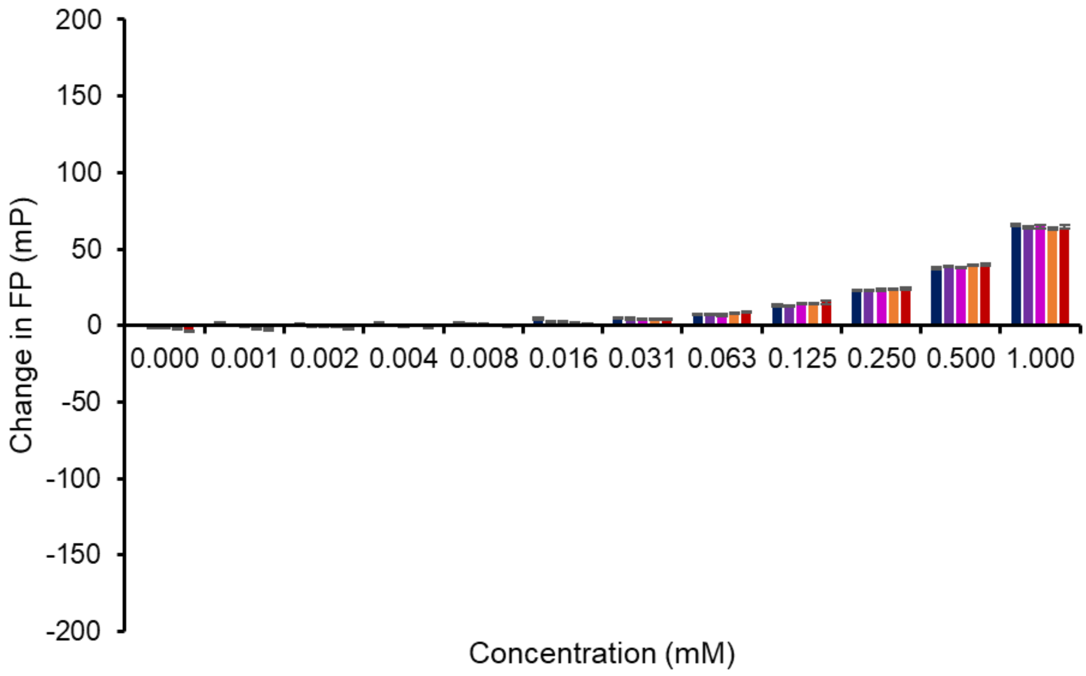

Figure S442 – Change in FP of **b** (0.15 mM) following addition of PE:PG 3:1 vesicles. Blue = 1 minute, purple = 5 minutes, pink = 10 minutes, orange = 15 minutes, red = 20 minutes. Average of n = 3 repeats. Error = one standard deviation of the mean.

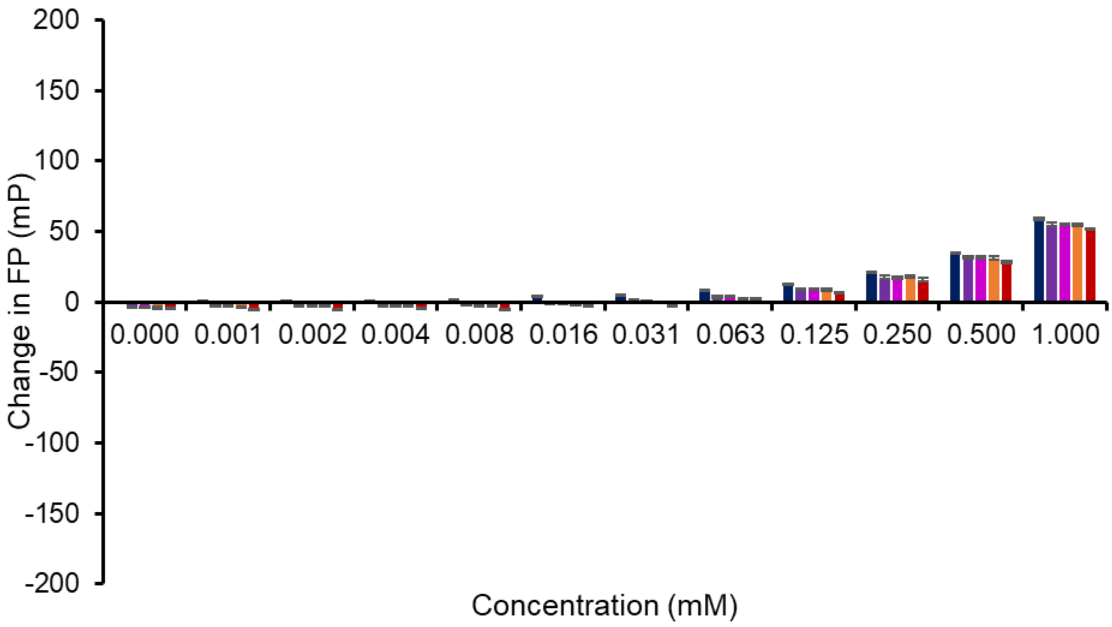

Figure S443 – Change in FP of **e** (0.15 mM) following addition of PE:PG 3:1 vesicles. Blue = 1 minute, purple = 5 minutes, pink = 10 minutes, orange = 15 minutes, red = 20 minutes. Average of n = 3 repeats. Error = one standard deviation of the mean.

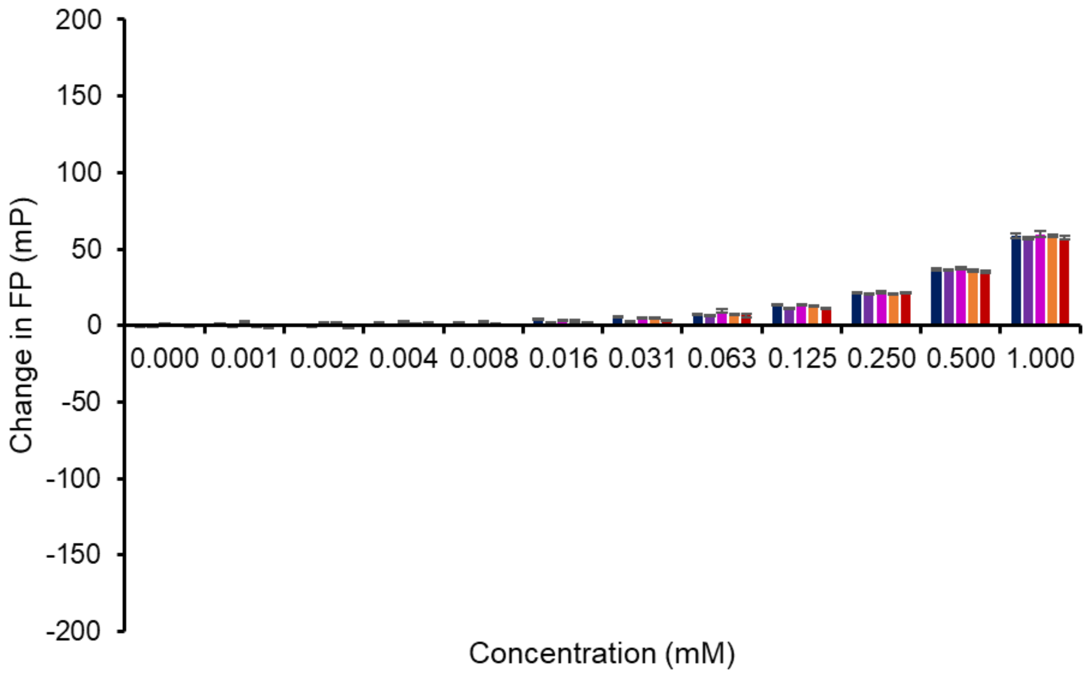

Figure S444 – Change in FP of **h** (0.15 mM) following addition of PE:PG 3:1 vesicles. Blue = 1 minute, purple = 5 minutes, pink = 10 minutes, orange = 15 minutes, red = 20 minutes. Average of n = 3 repeats. Error = one standard deviation of the mean.

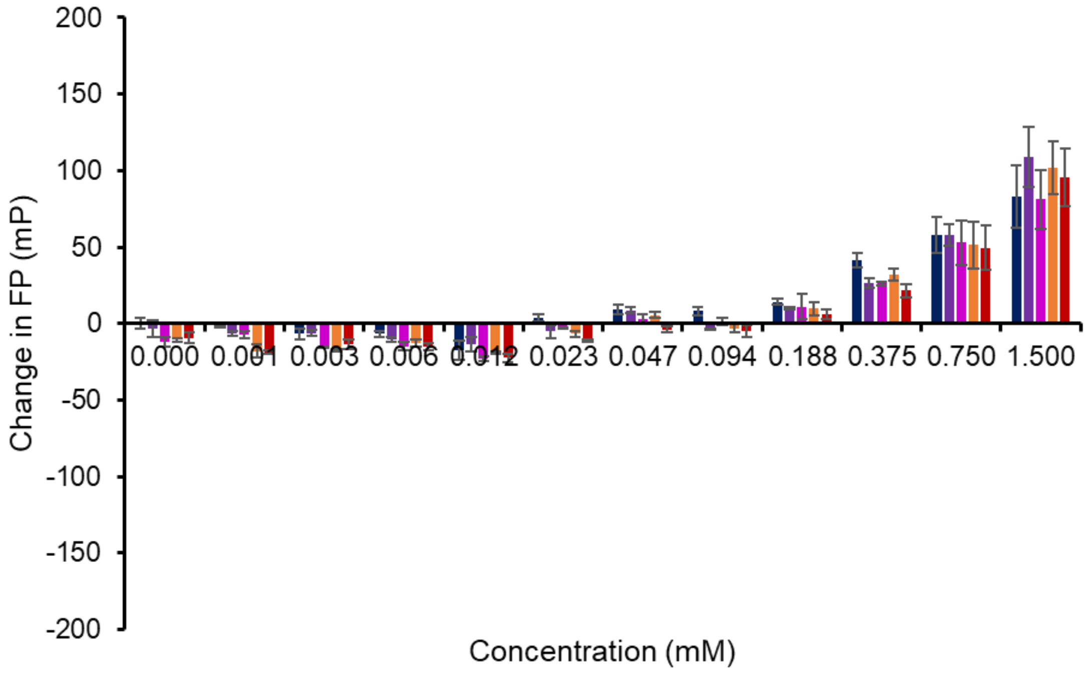

Figure S445 – Change in FP of **i** (0.15 mM) following addition of PE:PG 3:1 vesicles. Blue = 1 minute, purple = 5 minutes, pink = 10 minutes, orange = 15 minutes, red = 20 minutes. Average of n = 3 repeats. Error = one standard deviation of the mean.

PE:PG 1:1 FP titrations

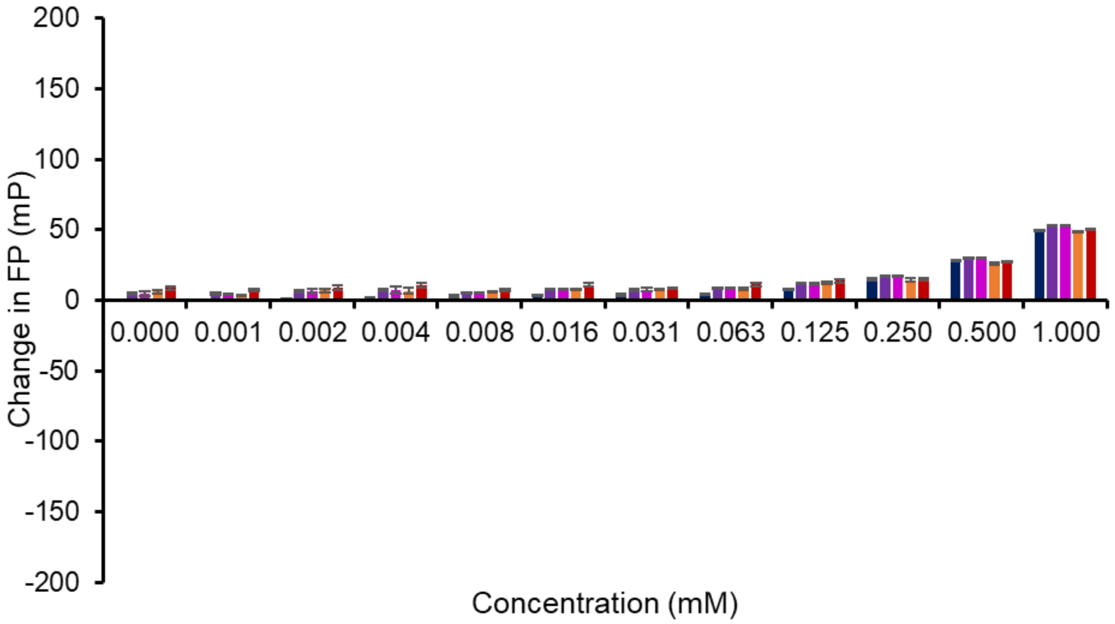

Figure S446 – Change in FP of **3** (0.15 mM) following addition of PE:PG 1:1 vesicles. Blue = 1 minute, purple = 5 minutes, pink = 10 minutes, orange = 15 minutes, red = 20 minutes. Average of n = 3 repeats. Error = one standard deviation of the mean.

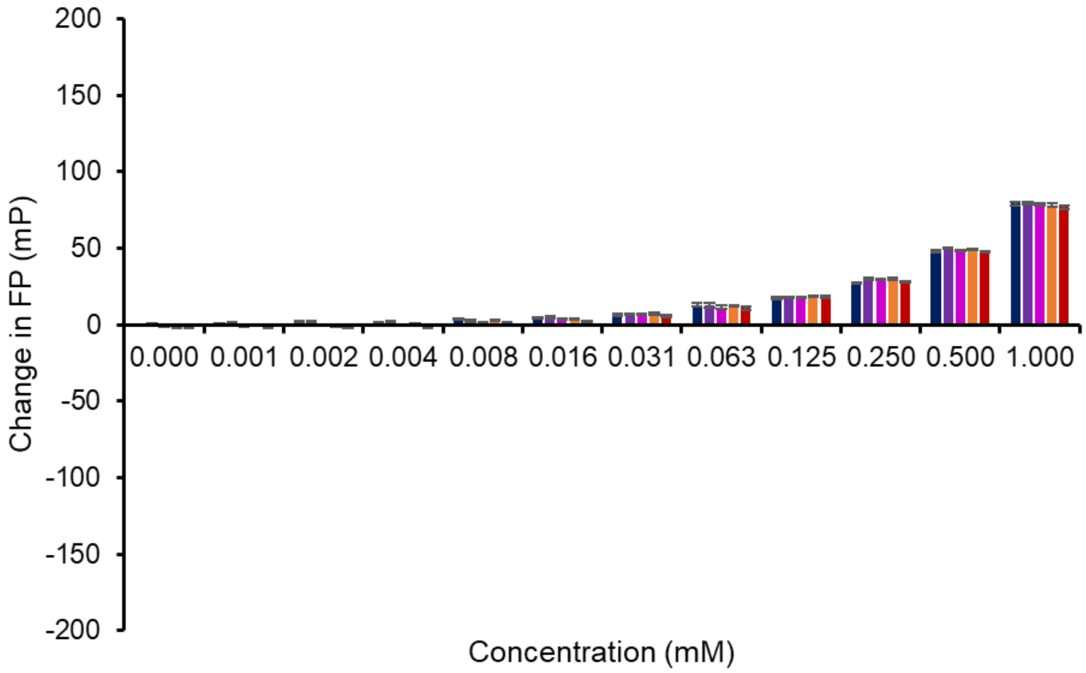

Figure S447 – Change in FP of **b** (0.15 mM) following addition of PE:PG 1:1 vesicles. Blue = 1 minute, purple = 5 minutes, pink = 10 minutes, orange = 15 minutes, red = 20 minutes. Average of n = 3 repeats. Error = one standard deviation of the mean.

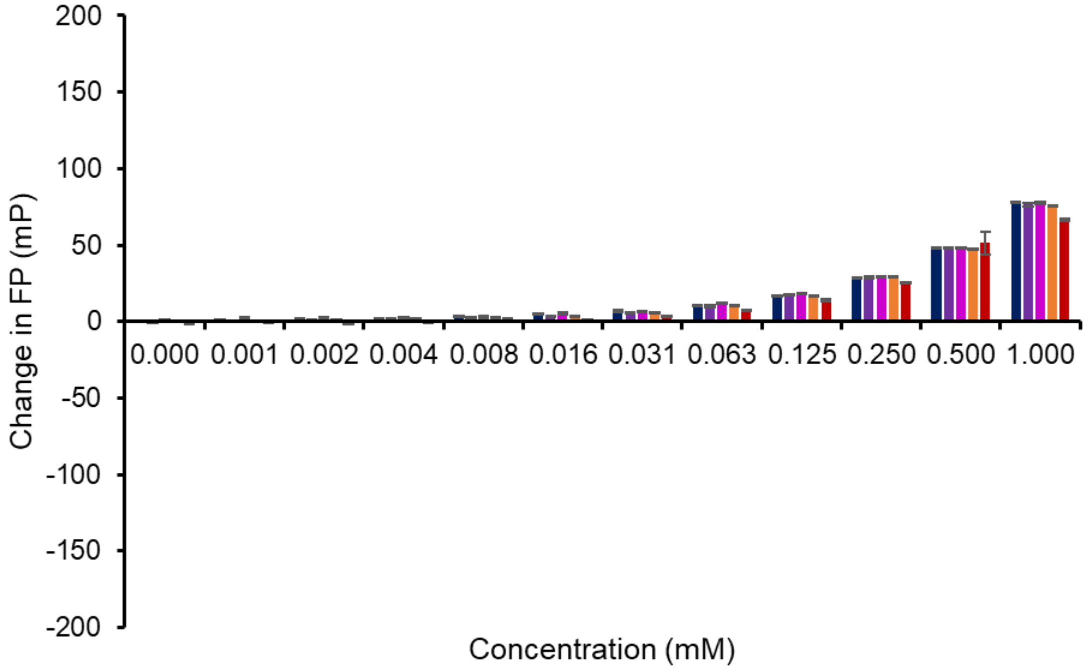

Figure S448 – Change in FP of **e** (0.15 mM) following addition of PE:PG 1:1 vesicles. Blue = 1 minute, purple = 5 minutes, pink = 10 minutes, orange = 15 minutes, red = 20 minutes. Average of n = 3 repeats. Error = one standard deviation of the mean.

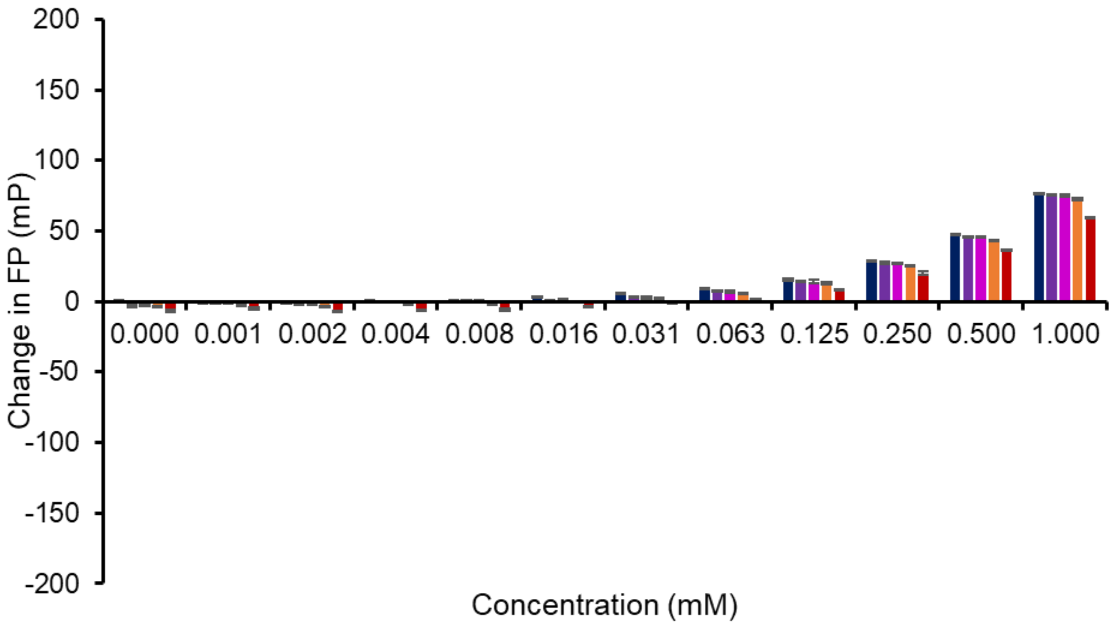

Figure S449 – Change in FP of **h** (0.15 mM) following addition of PE:PG 1:1 vesicles. Blue = 1 minute, purple = 5 minutes, pink = 10 minutes, orange = 15 minutes, red = 20 minutes. Average of n = 3 repeats. Error = one standard deviation of the mean.

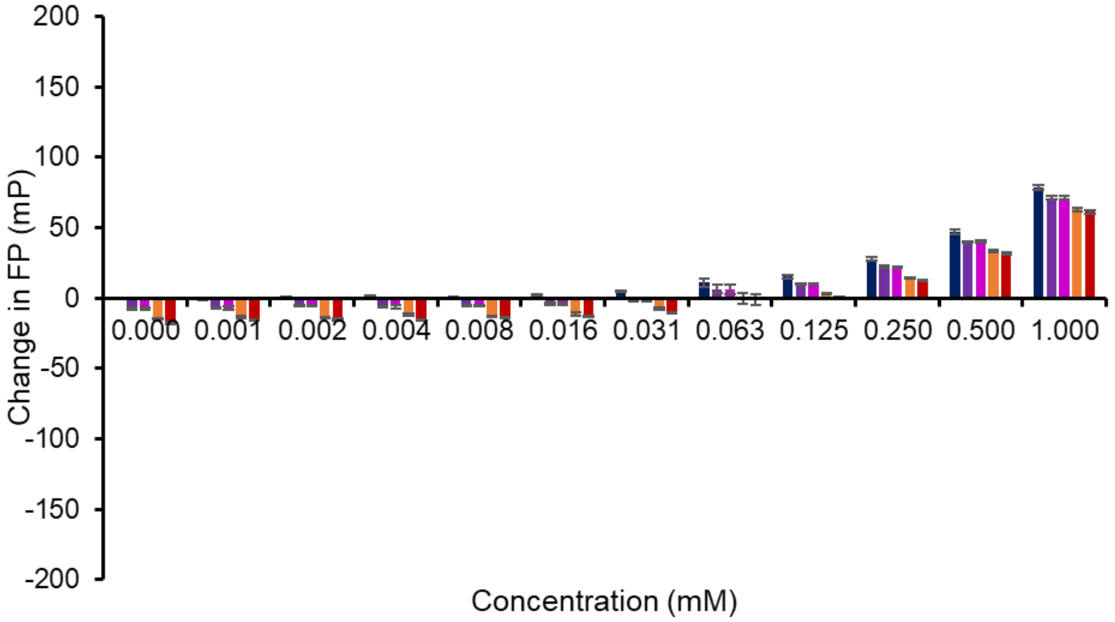

Figure S450 – Change in FP of **i** (0.15 mM) following addition of PE:PG 1:1 vesicles. Blue = 1 minute, purple = 5 minutes, pink = 10 minutes, orange = 15 minutes, red = 20 minutes. Average of n = 3 repeats. Error = one standard deviation of the mean.

Comparison of membrane adhesion FP data

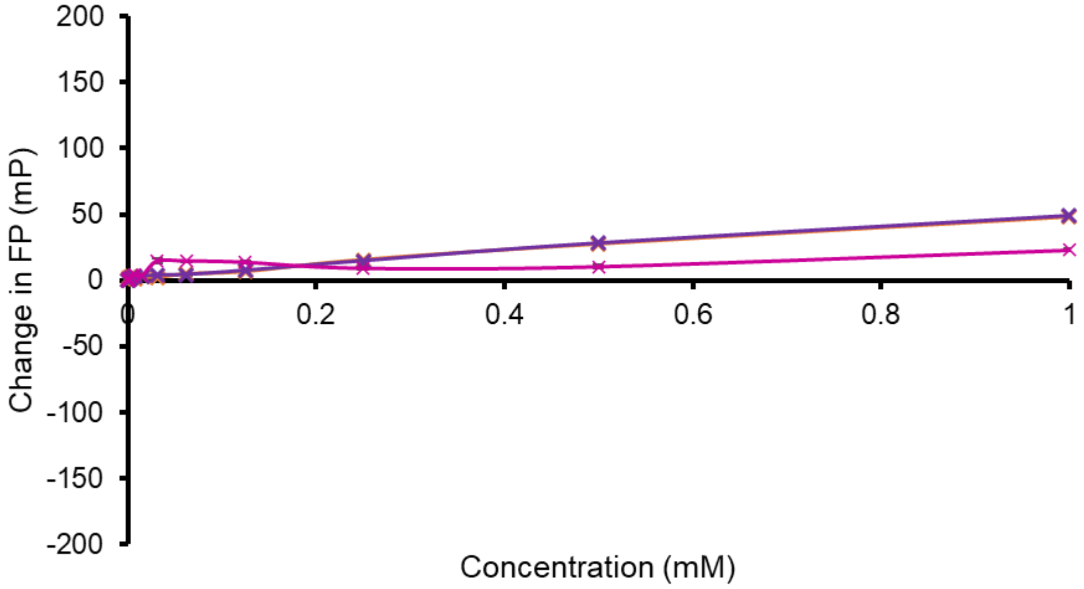

Figure S451 – Change in FP of **3** (0.15 mM) following the addition of different ratios of lipids at 1 minute. Orange = PG, pink = PE:PG 3:1, purple = PE:PG 1:1. Average of n = 3 repeats. Error = one standard deviation of the mean.

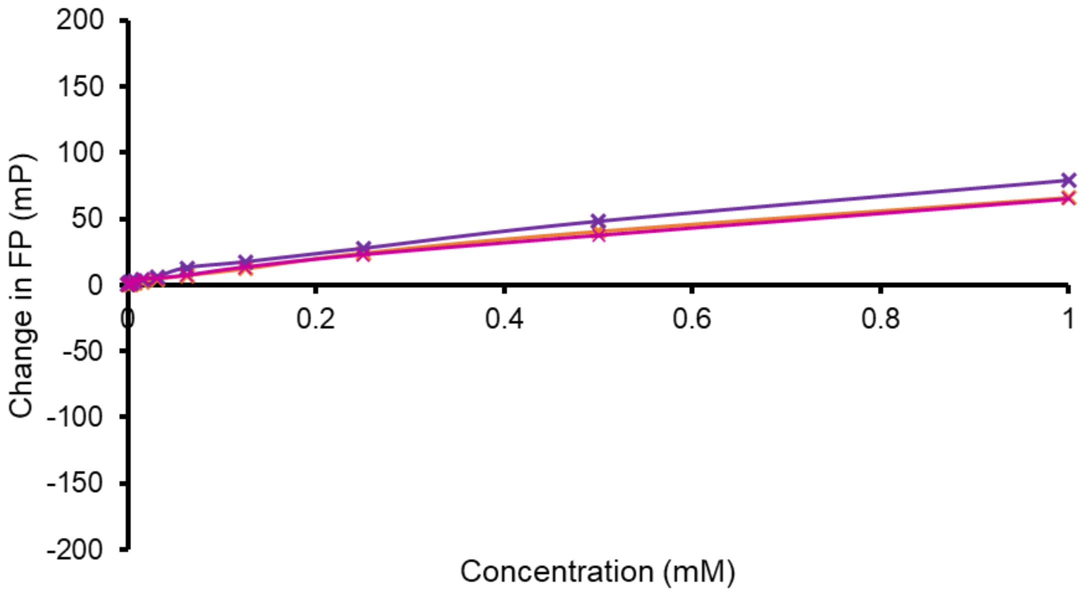

Figure S452 – Change in FP of **b** (0.15 mM) following the addition of different ratios of lipids at 1 minute. Orange = PG, pink = PE:PG 3:1, purple = PE:PG 1:1. Average of n = 3 repeats. Error = one standard deviation of the mean.

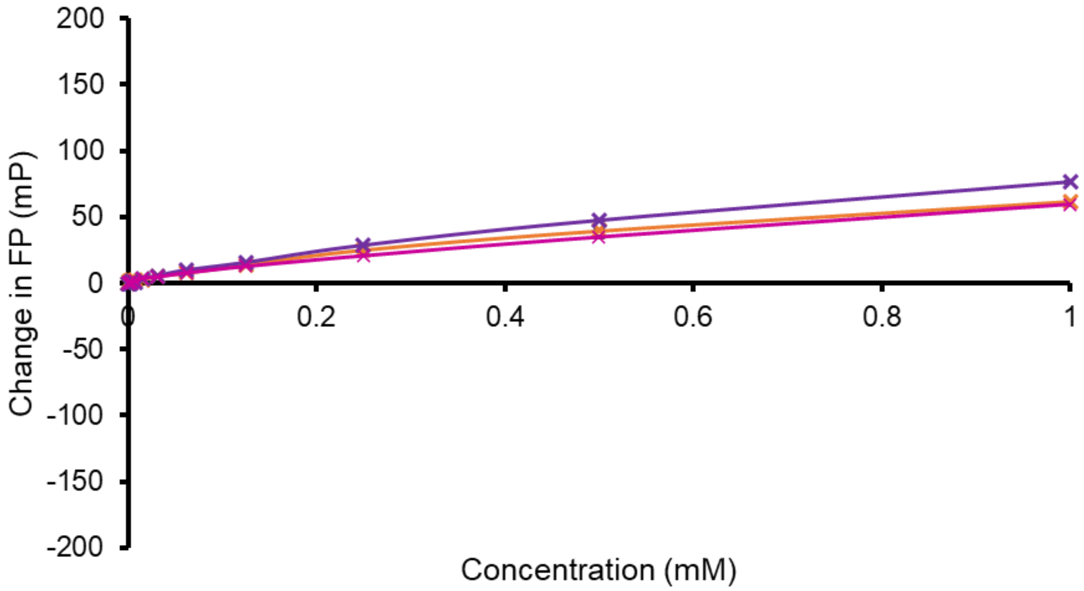

Figure S453 – Change in FP of **e** (0.15 mM) following the addition of different ratios of lipids at 1 minute. Orange = PG, pink = PE:PG 3:1, purple = PE:PG 1:1. Average of n = 3 repeats. Error = one standard deviation of the mean.

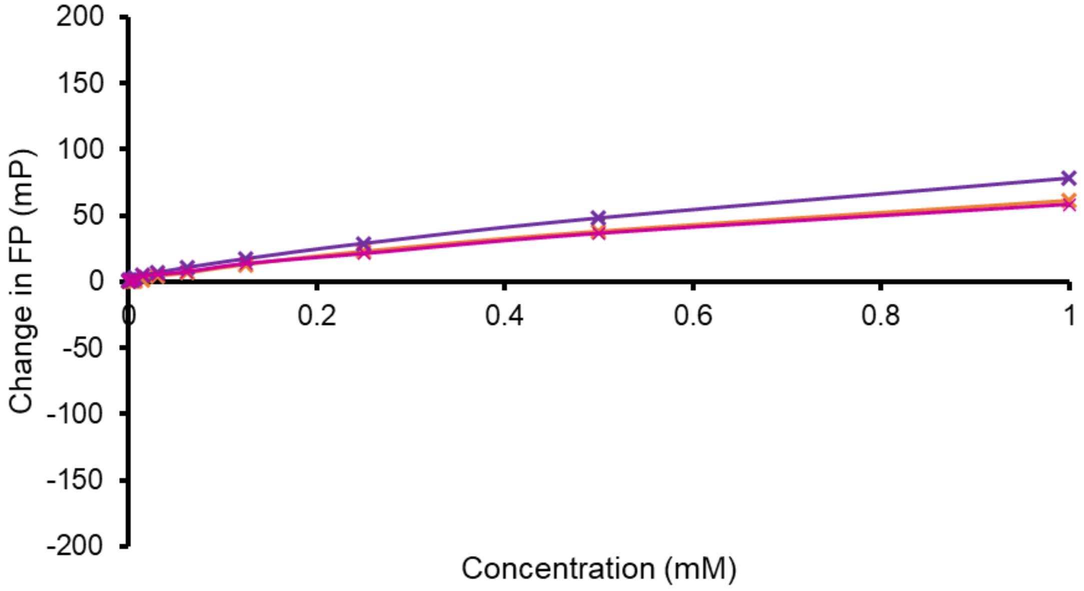

Figure S454 – Change in FP of **h** (0.15 mM) following the addition of different ratios of lipids at 1 minute. Orange = PG, pink = PE:PG 3:1, purple = PE:PG 1:1. Average of n = 3 repeats. Error = one standard deviation of the mean.

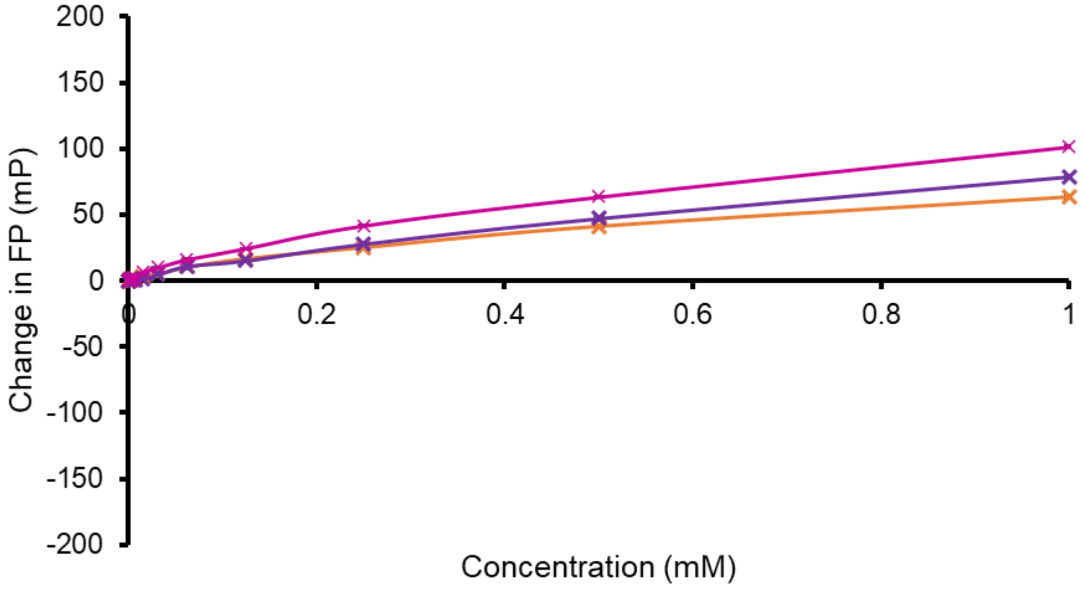

Figure S455 – Change in FP of **i** (0.15 mM) following the addition of different ratios of lipids at 1 minute. Orange = PG, pink = PE:PG 3:1, purple = PE:PG 1:1. Average of n = 3 repeats. Error = one standard deviation of the mean.

Section S17: MAF and PF determination from NMR spectroscopy data

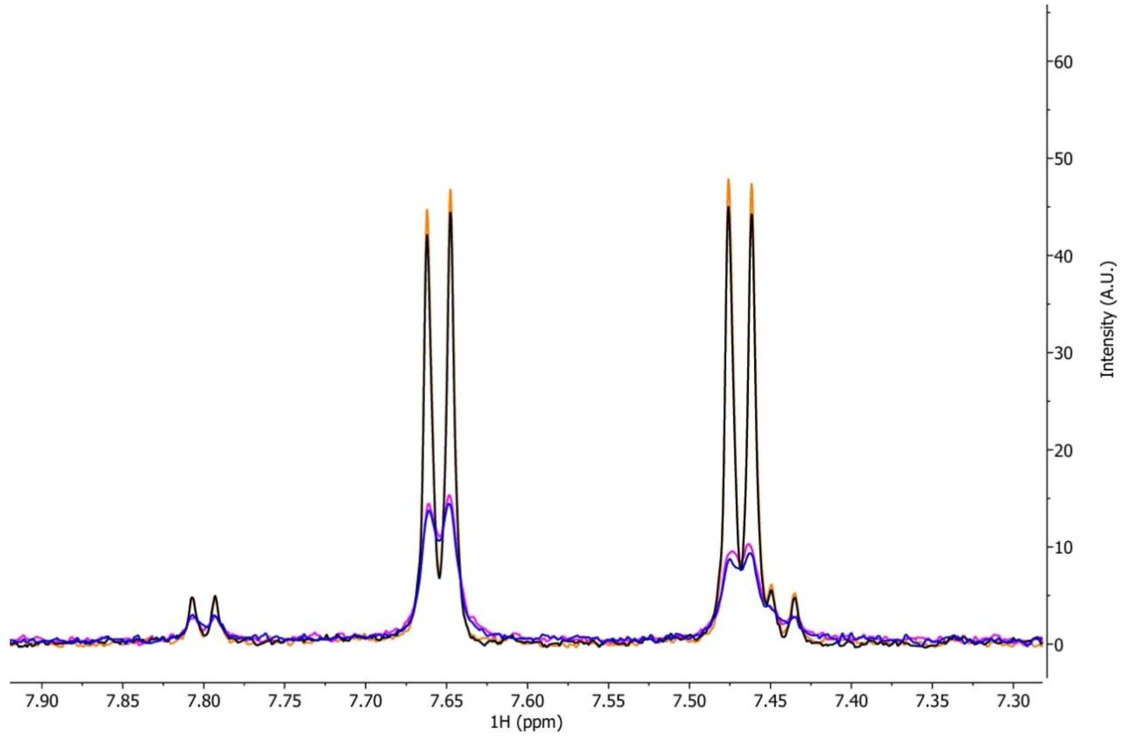

Figure S456 – An overlay of four *1*H CPMG NMR spectra collected with a spin-lock time of 150 mS of **1** (200 µM) in the absence of lipid vesicles (orange), in the presence of gadodiamide (pink), in the presence of lipid vesicles (black) and in the presence of both lipid vesicles and gadodiamide (blue).

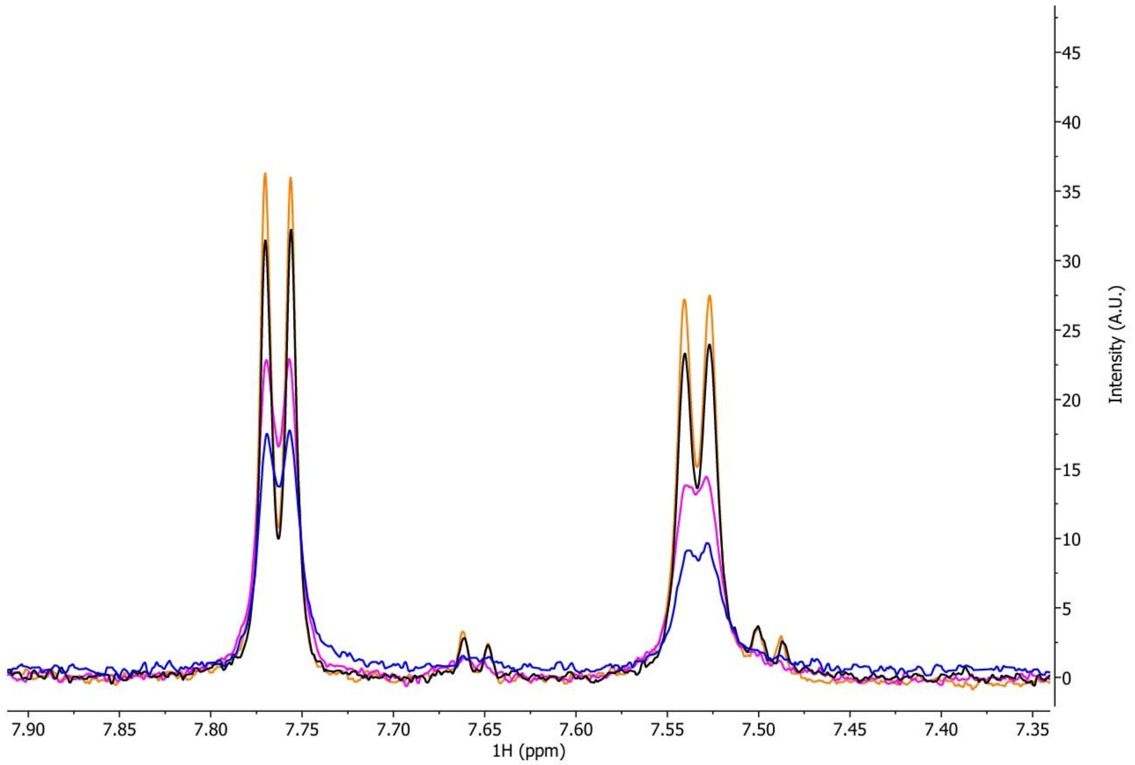

Figure S457 – An overlay of four ^{1}H CPMG NMR spectra collected with a spin-lock time of 150 mS of **2** (200 µM) in the absence of lipid vesicles (orange), in the presence of gadodiamide (pink), in the presence of lipid vesicles (black) and in the presence of both lipid vesicles and gadodiamide (blue).

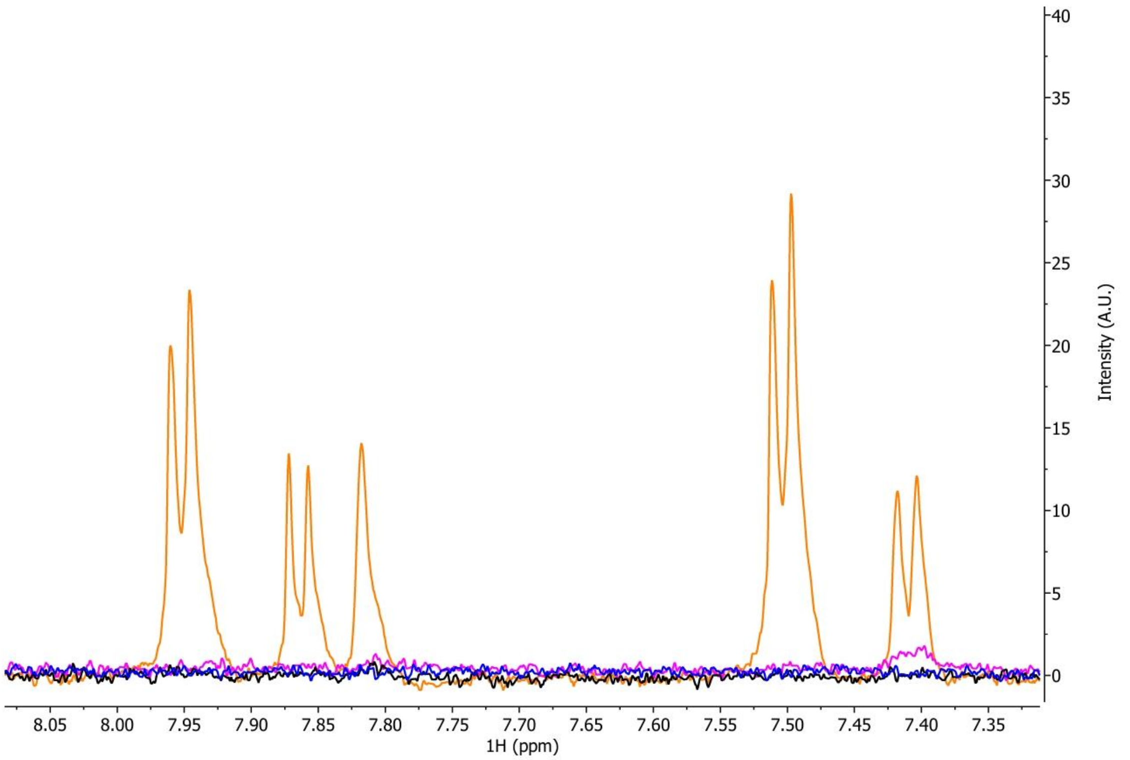

Figure S458 – An overlay of four ^{1}H CPMG NMR spectra collected with a spin-lock time of 150 mS of **3** (200 µM) in the absence of lipid vesicles (orange), in the presence of gadodiamide (pink), in the presence of lipid vesicles (black) and in the presence of both lipid vesicles and gadodiamide (blue).

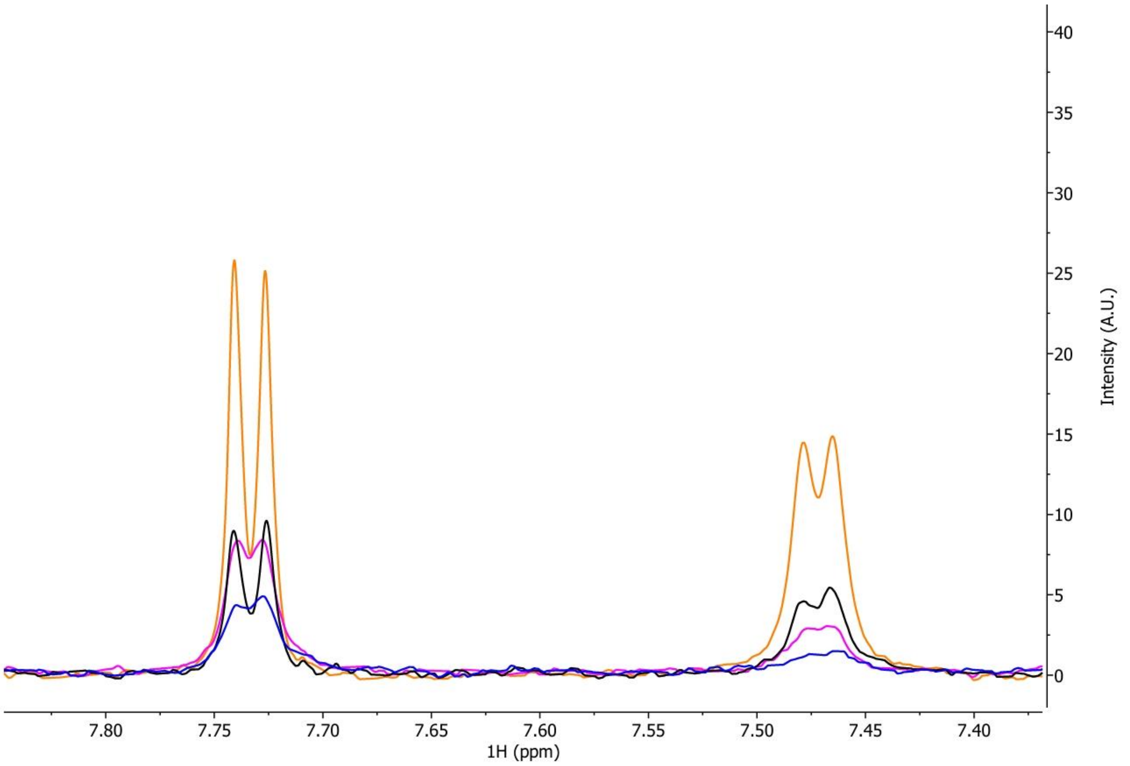

Figure S459 – An overlay of four ^{1}H CPMG NMR spectra collected with a spin-lock time of 150 mS of **4** (200 µM) in the absence of lipid vesicles (orange), in the presence of gadodiamide (pink), in the presence of lipid vesicles (black) and in the presence of both lipid vesicles and gadodiamide (blue).

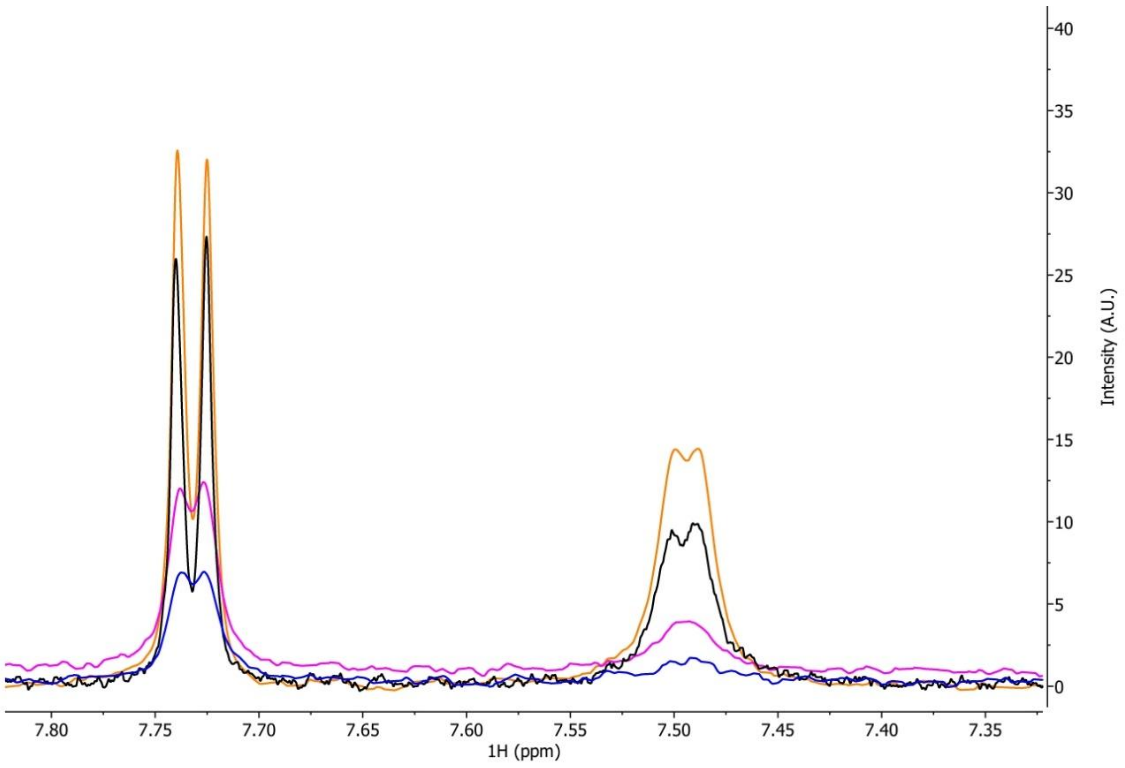

Figure S460 – An overlay of four ^{1}H CPMG NMR spectra collected with a spin-lock time of 150 mS of **5** (200 µM) in the absence of lipid vesicles (orange), in the presence of gadodiamide (pink), in the presence of lipid vesicles (black) and in the presence of both lipid vesicles and gadodiamide (blue).

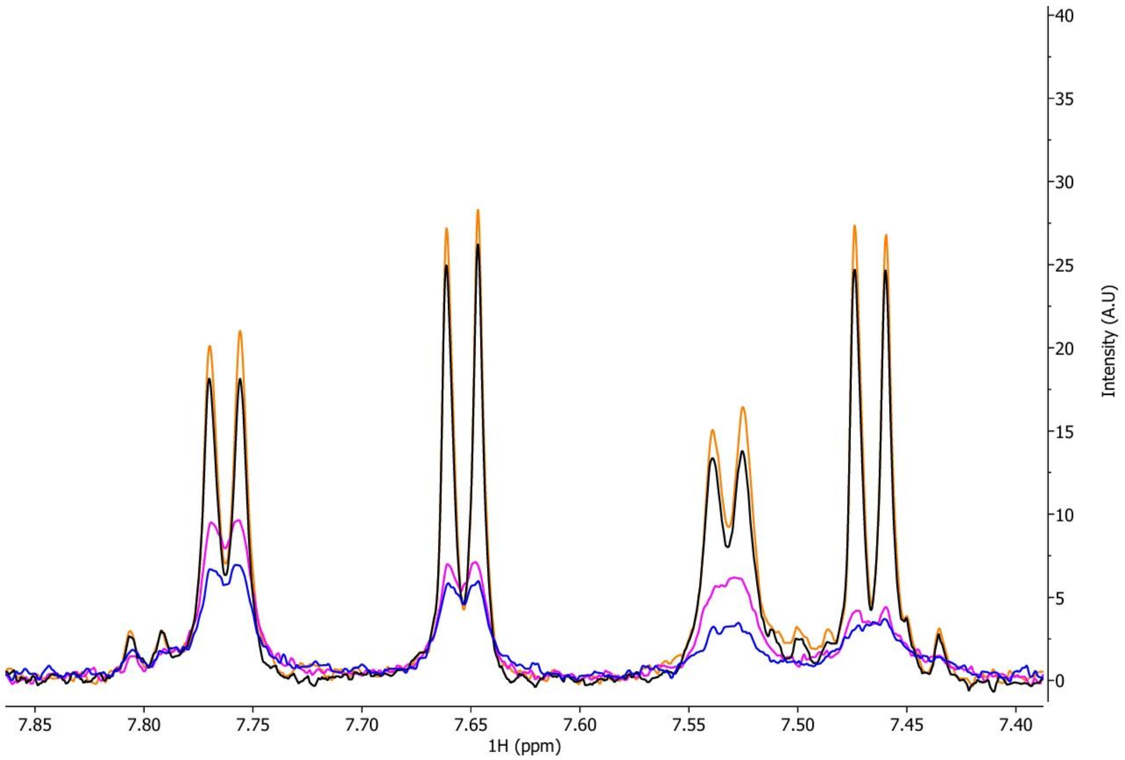

Figure S461 – An overlay of four ^{1}H CPMG NMR spectra collected with a spin-lock time of 150 mS of co-formulation **a** (200 µM) in the absence of lipid vesicles (orange), in the presence of gadodiamide (pink), in the presence of lipid vesicles (black) and in the presence of both lipid vesicles and gadodiamide (blue).

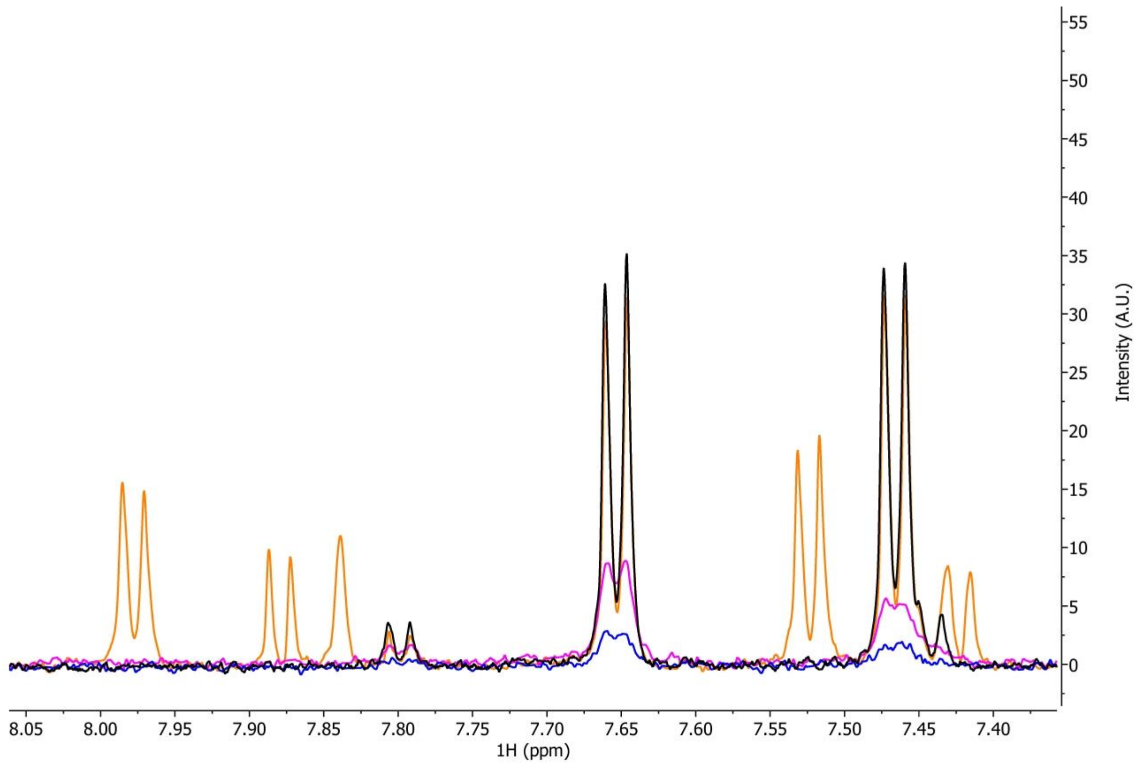

Figure S462 – An overlay of four ^{1}H CPMG NMR spectra collected with a spin-lock time of 150 mS of co-formulation **b** (200 µM) in the absence of lipid vesicles (orange), in the presence of gadodiamide (pink), in the presence of lipid vesicles (black) and in the presence of both lipid vesicles and gadodiamide (blue).

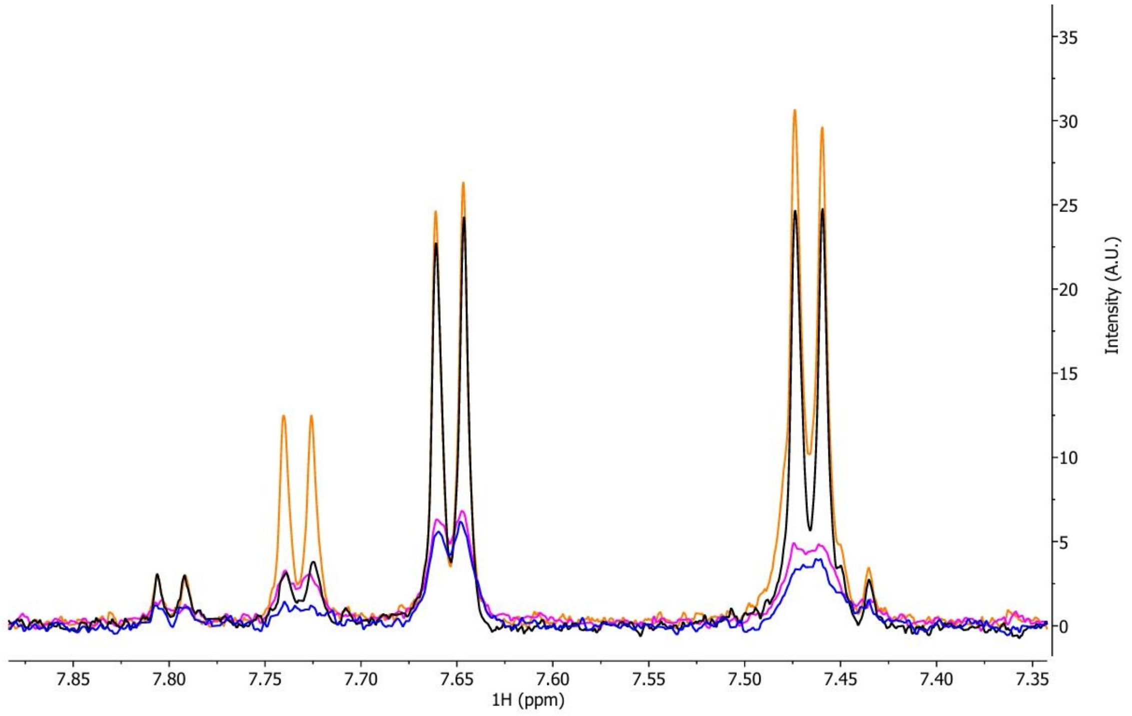

Figure S463 – An overlay of four ^{1}H CPMG NMR spectra collected with a spin-lock time of 150 mS of co-formulation **c** (200 µM) in the absence of lipid vesicles (orange), in the presence of gadodiamide (pink), in the presence of lipid vesicles (black) and in the presence of both lipid vesicles and gadodiamide (blue).

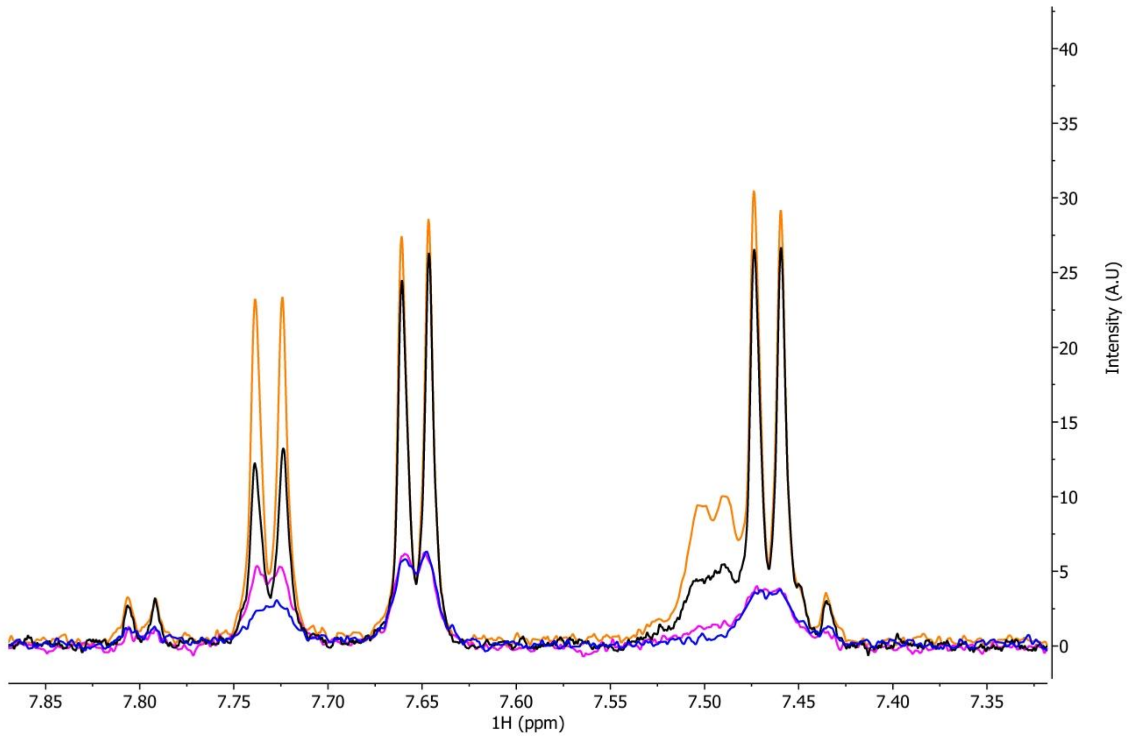

Figure S464 – An overlay of four ^{1}H CPMG NMR spectra collected with a spin-lock time of 150 mS of co-formulation **d** (200 µM) in the absence of lipid vesicles (orange), in the presence of gadodiamide (pink), in the presence of lipid vesicles (black) and in the presence of both lipid vesicles and gadodiamide (blue).

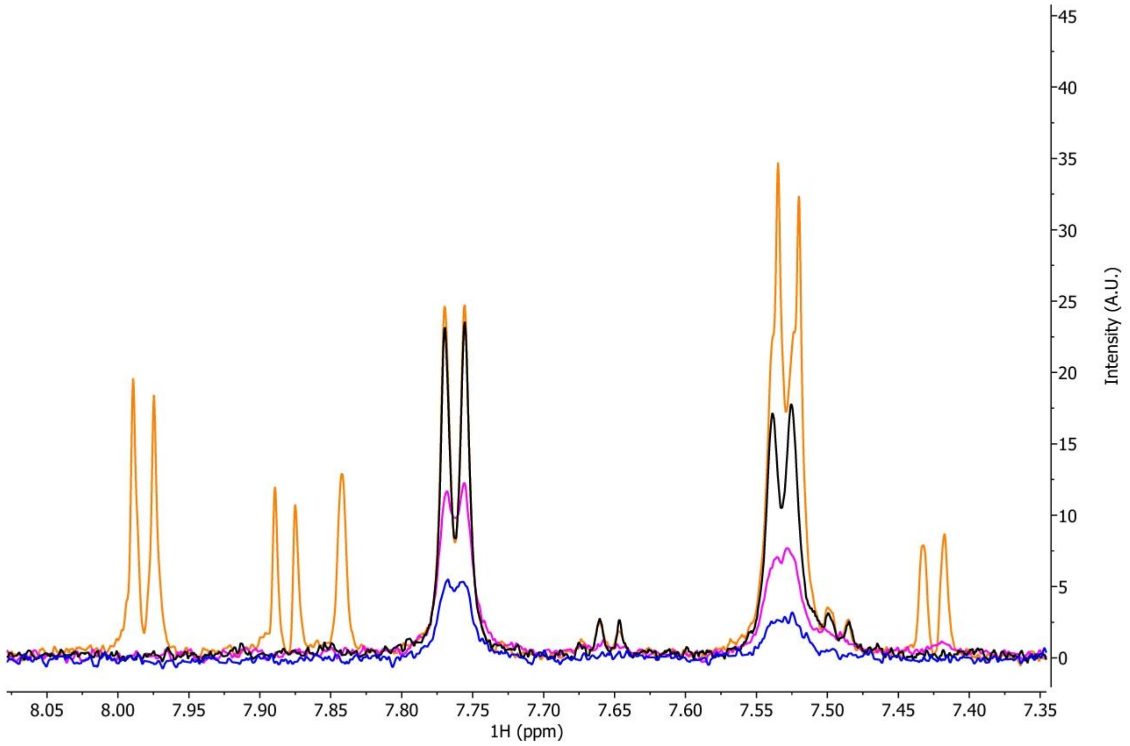

Figure S465 – An overlay of four ^{1}H CPMG NMR spectra collected with a spin-lock time of 150 mS of co-formulation **e** (200 µM) in the absence of lipid vesicles (orange), in the presence of gadodiamide (pink), in the presence of lipid vesicles (black) and in the presence of both lipid vesicles and gadodiamide (blue).

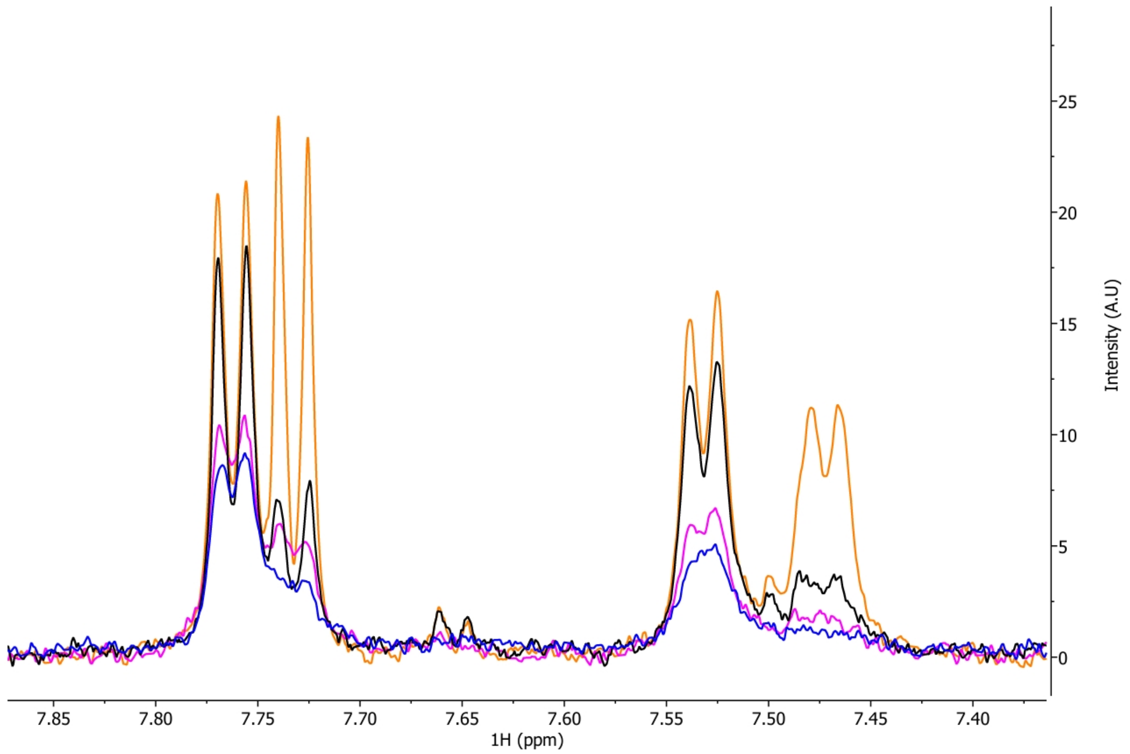

Figure S466 – An overlay of four ^{1}H CPMG NMR spectra collected with a spin-lock time of 150 mS of co-formulation **f** (200 µM) in the absence of lipid vesicles (orange), in the presence of gadodiamide (pink), in the presence of lipid vesicles (black) and in the presence of both lipid vesicles and gadodiamide (blue).

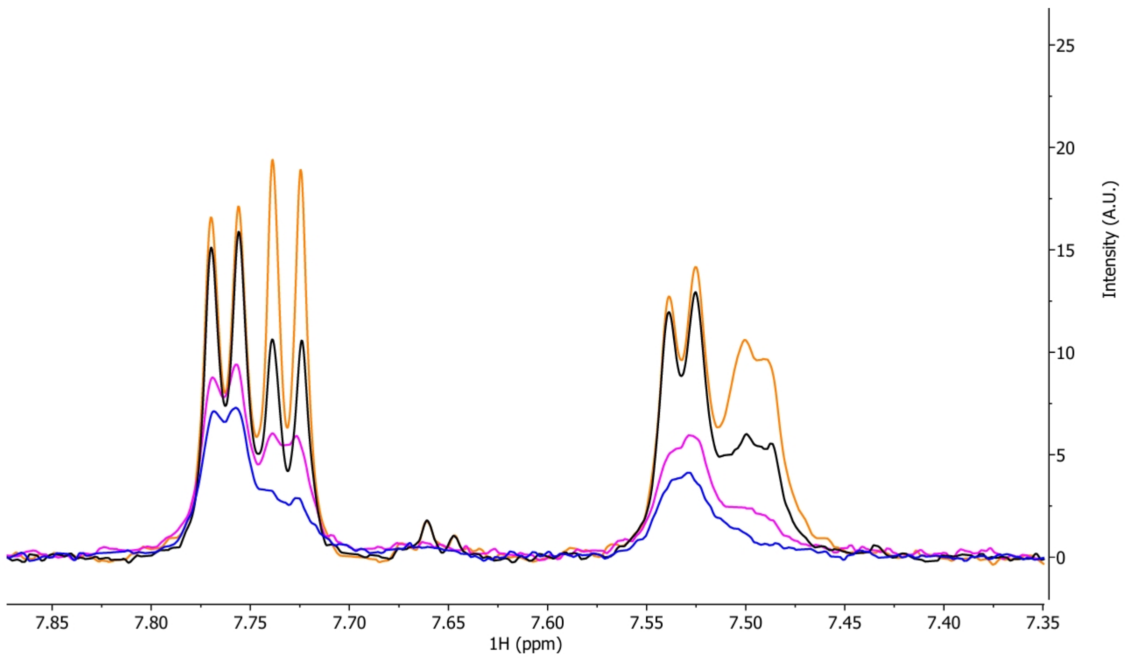

Figure S467 – An overlay of 4 proton 1D CPMG NMR spectra collected with a spin-lock time of 150 mS of co-formulation **g** (200 µM) in the absence of lipid vesicles (orange), in the presence of gadodiamide (pink), in the presence of lipid vesicles (black) and in the presence of both lipid vesicles and gadodiamide (blue).

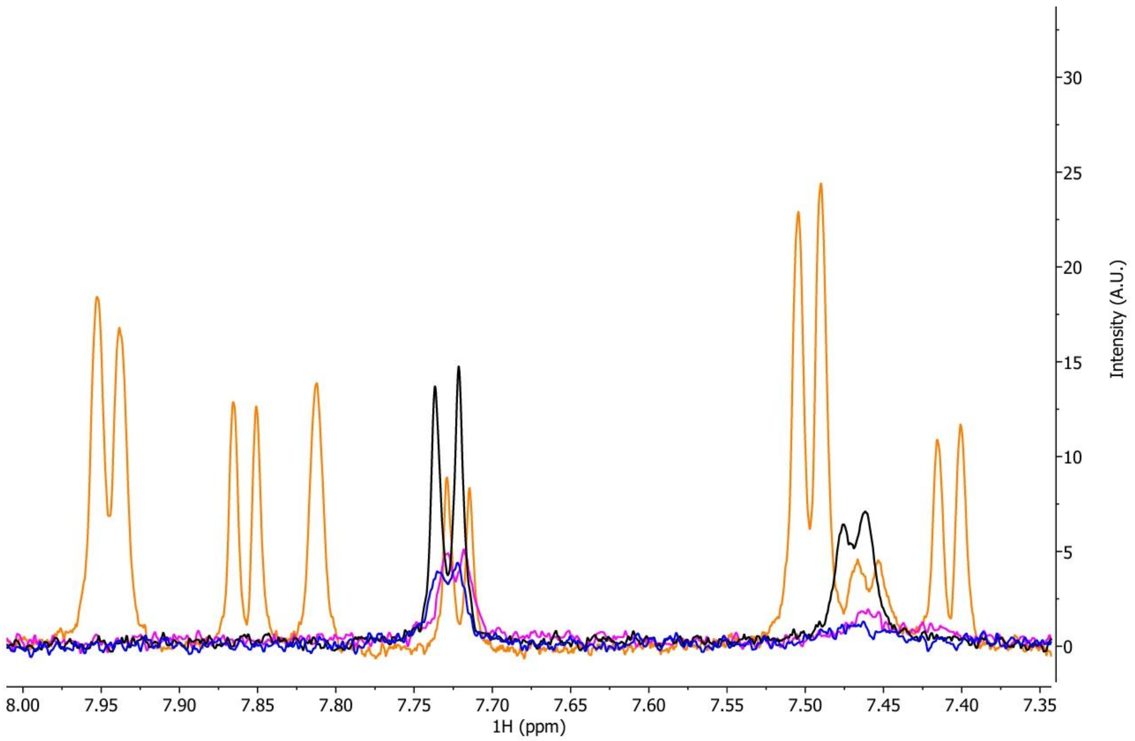

Figure S468 – An overlay of four ^{1}H CPMG NMR spectra collected with a spin-lock time of 150 mS of co-formulation **h** (200 µM) in the absence of lipid vesicles (orange), in the presence of gadodiamide (pink), in the presence of lipid vesicles (black) and in the presence of both lipid vesicles and gadodiamide (blue).

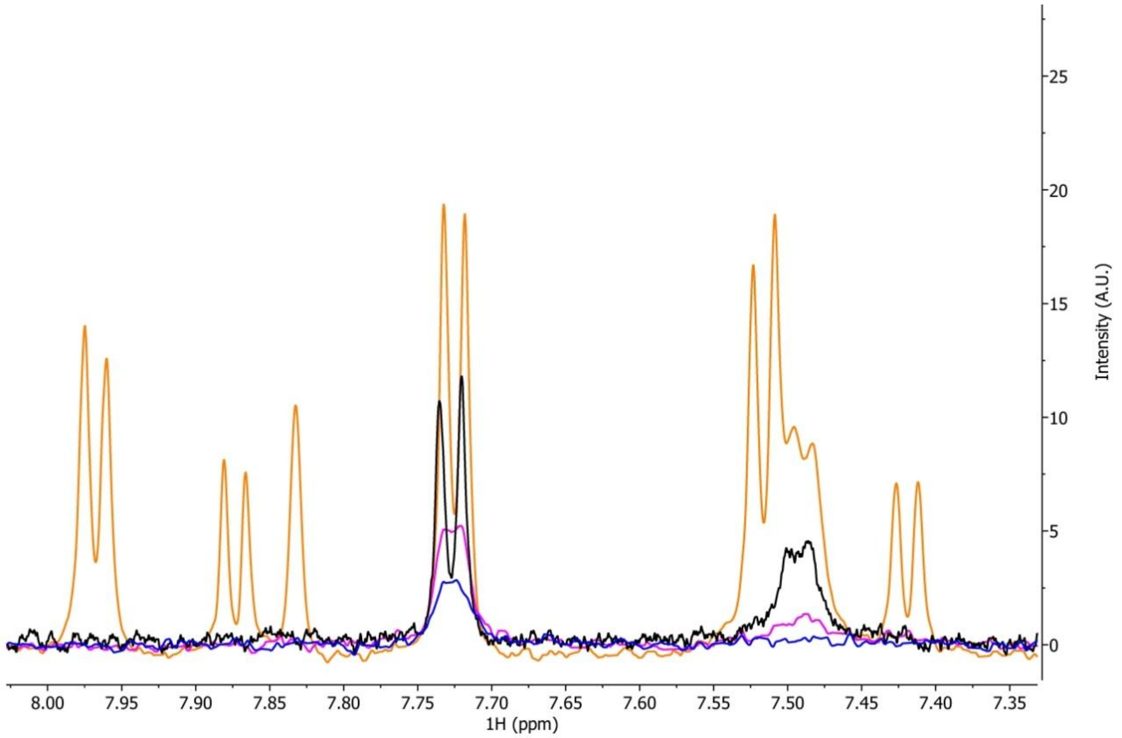

Figure S469 – An overlay of four ^{1}H CPMG NMR spectra collected with a spin-lock time of 150 mS of co-formulation **i** (200 µM) in the absence of lipid vesicles (orange), in the presence of gadodiamide (pink), in the presence of lipid vesicles (black) and in the presence of both lipid vesicles and gadodiamide (blue).

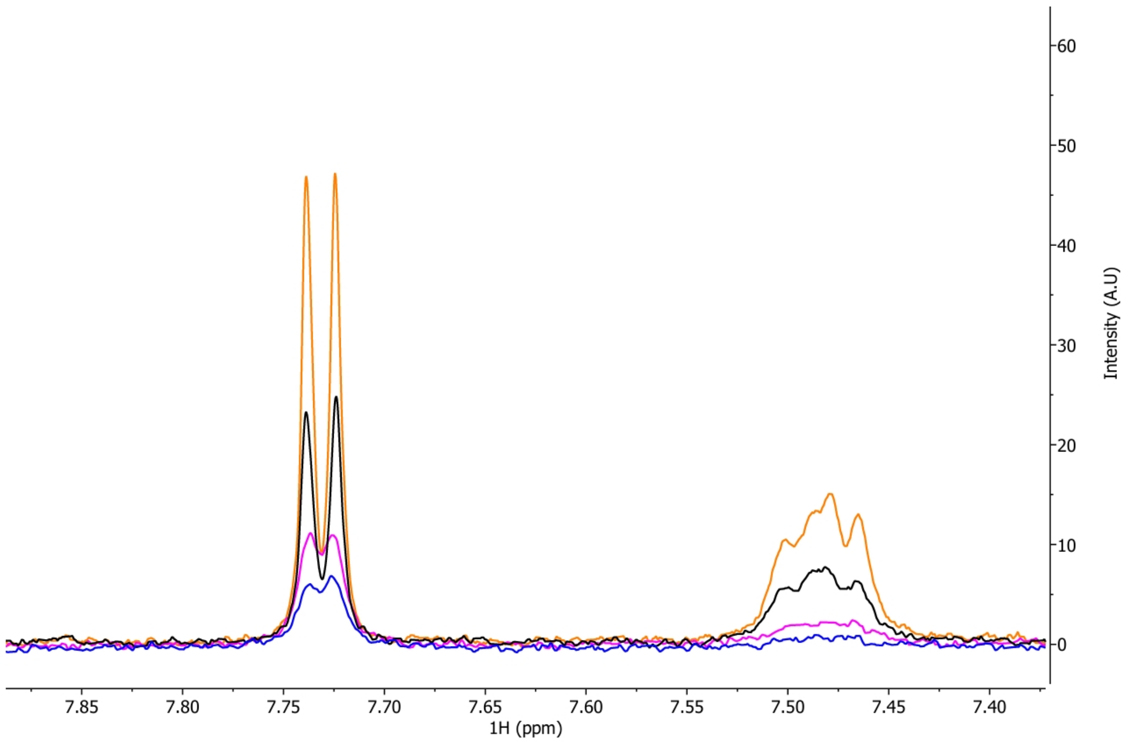

Figure S470 – An overlay of four ^{1}H CPMG NMR spectra collected with a spin-lock time of 150 mS of co-formulation **j** (200 µM) in the absence of lipid vesicles (orange), in the presence of gadodiamide (pink), in the presence of lipid vesicles (black) and in the presence of both lipid vesicles and gadodiamide (blue).

Section S18: Antimicrobial data

Table S15 – Summary of antimicrobial activity for **4**, **5** and **a** – **j** against planktonic bacteria. *P. aeruginosa* – PAO1, *A. baumannii* – 17978, *E. coli* – 12923, *S. aureus* – 9144, *E. faecalis* – 775, *E. faecium* – 12204. '-' Indicates MIC is greater than compound solubility, modal MIC values given. Average of n = 2 biological repeats.

Compound	*P. aeruginosa* (mM)	*A. baumannii* (mM)	*E. coli* (mM)	*S. aureus* (mM)	*E. faecalis* (mM)	*E. faecium* (mM)
**4**	-	3.75 - >3.75	-	1.88	-	3.75 - >3.75
**5**	-	-	-	0.70	-	-
**a**	-	5.00 - >5.00	-	5.00	5.00 - >5.00	5.00
**b**	5.00	5.00 - >5.00	5 - >5	5.00	5.00 - >5.00	5.00
**c**	-	3.75 - >3.75	-	1.88	3.75	3.75
**d**	-	-	-	0.70	-	1.39
**e**	5.00 - >5.00	-	5 - >5	5.00 - >5.00	5.00 - >5.00	5.00 - >5.00
**f**	-	3.75 - >3.75	-	3.75	3.75 - >3.75	3.75
**g**	-	-	-	-	-	2.78 - >2.78
**h**	-	-	-	0.94	3.75	3.75
**i**	-	-	-	0.70	-	-
**j**	-	-	-	0.70	-	-

>5 mM <1 mM

Section S19: Mass spectrometry data

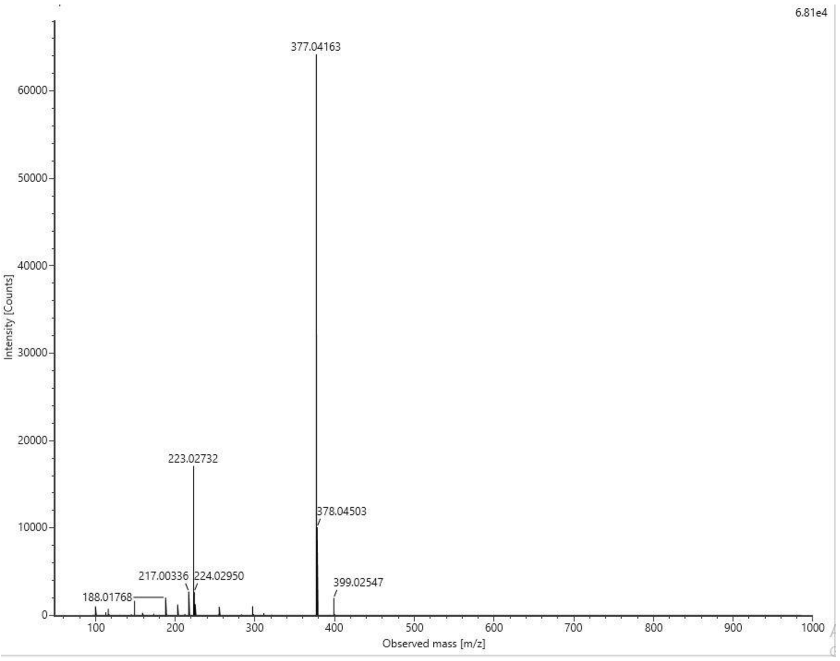

Figure S471 - A high-resolution mass spectrum (ESI$^-$) obtained for **4**, *m/z* [M]$^-$.

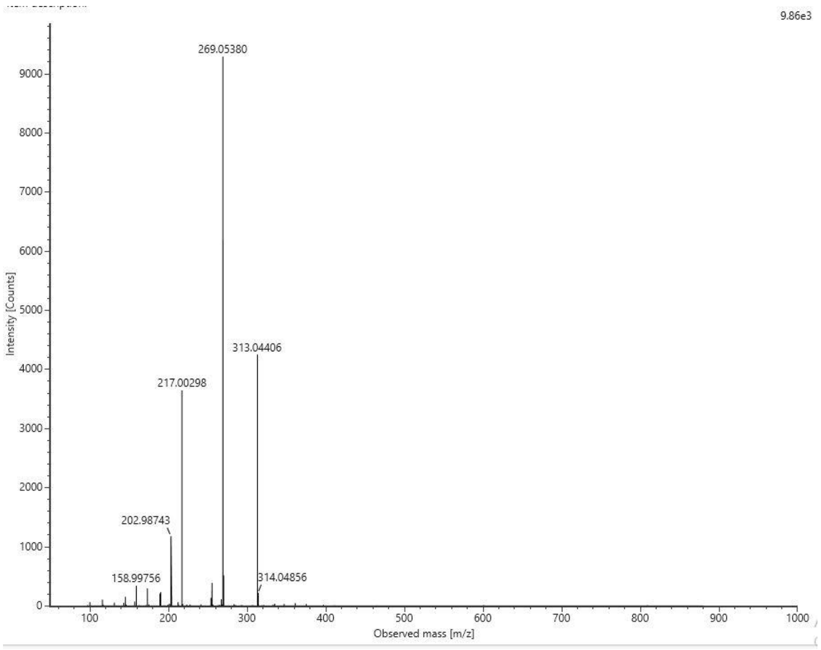

Figure S472 - A high-resolution mass spectrum (ESI$^-$) obtained for **5**, *m/z* [M]$^-$.

Section S20: References

1 P. Thordarson, K. Sewell and V. Efremova, Bindfit v0.5. http://app.supramolecular.org/bindfit/ Accessed (03/03/2025), .
2 S. Dutta, B. Watson, S. Mattoo and J.-C. Rochet, *Bio-protocol*, 2020, **10(14)**, e3690.
3 A. Serrano-Sanchez, M. Rice, J. Cassar, L. J. White, P. I. A. Popoola, G. S. Thompson, J. R. Hiscock and J. L. Ortega-Roldan, *Chem. Commun.*, 2024, **60**, 11160–11163.
4 R. Fourie, R. Ells, G. Kemp, O. M. Sebolai, J. Albertyn and C. H. Pohl, *Prostaglandins Leukot. Essent. Fatty Acids*, 2017, **117**, 36–46.
5 S. Stepanović, D. Vuković, I. Dakić, B. Savić and M. Švabić-Vlahović, *J. Microbiol. Methods*, 2000, **40**, 175–179.
6 G. A. O'Toole, *JoVE*, 2011, 2437.
7 L. J. White, S. N. Tyuleva, B. Wilson, H. J. Shepherd, K. K. L. Ng, S. J. Holder, E. R. Clark and J. R. Hiscock, *Chem. Eur. J.l*, 2018, **24**, 7761–7773.
8 L. J. White, N. J. Wells, L. R. Blackholly, H. J. Shepherd, B. Wilson, G. P. Bustone, T. J. Runacres and J. R. Hiscock, *Chem. Sci.*, 2017, **8**, 7620–7630.
9 Y. Zhu, J. P. Malerich and V. H. Rawal, *Angwe. Chem. Int. Ed.*, 2010, **49**, 153–156.
